# Concerted gene recruitment in early plant evolution

**DOI:** 10.1186/gb-2008-9-7-r109

**Published:** 2008-07-08

**Authors:** Jinling Huang, J Peter Gogarten

**Affiliations:** 1 Department of Biology, Howell Science Complex, East Carolina University, Greenville, NC 27858, USA; 2 Department of Molecular and Cell Biology, University of Connecticut, 91 North Eagleville Road, Storrs, CT 06269, USA

## Abstract

**Background:**

Horizontal gene transfer occurs frequently in prokaryotes and unicellular eukaryotes. Anciently acquired genes, if retained among descendants, might significantly affect the long-term evolution of the recipient lineage. However, no systematic studies on the scope of anciently acquired genes and their impact on macroevolution are currently available in eukaryotes.

**Results:**

Analyses of the genome of the red alga *Cyanidioschyzon *identified 37 genes that were acquired from non-organellar sources prior to the split of red algae and green plants. Ten of these genes are rarely found in cyanobacteria or have additional plastid-derived homologs in plants. These genes most likely provided new functions, often essential for plant growth and development, to the ancestral plant. Many remaining genes may represent replacements of endogenous homologs with a similar function. Furthermore, over 78% of the anciently acquired genes are related to the biogenesis and functionality of plastids, the defining character of plants.

**Conclusion:**

Our data suggest that, although ancient horizontal gene transfer events did occur in eukaryotic evolution, the number of acquired genes does not predict the role of horizontal gene transfer in the adaptation of the recipient organism. Our data also show that multiple independently acquired genes are able to generate and optimize key evolutionary novelties in major eukaryotic groups. In light of these findings, we propose and discuss a general mechanism of horizontal gene transfer in the macroevolution of eukaryotes.

## Abstract 2

Analyses of the red algal *Cyanidioschyzon* genome identified 37 genes that were acquired from non-organellar sources prior to the split of red algae and green plants.

## Background

The role of horizontal gene transfer (HGT) in prokaryotic evolution has long been documented in numerous studies, from bacterial pathogenesis to the spread of antibiotic resistance and nitrogen fixation [1-3]. The proportion of genes affected by HGT has been estimated from an average of 7% to over 65% in prokaryotic genomes [4-8]. The pervasive occurrence of gene transfer has revolutionized our view of microbial evolution - microbial evolution must be considered reticulate and cooperative by sharing genes and resources among organisms in the community [9,10].

Reticulate evolution and gene transfer have long been known in eukaryotes. Hybridization, which occurs frequently in seed plants [11], can be viewed as a form of HGT. However, since eukaryotic genomes are relatively stable, hybridization between closely related taxa rarely involves acquisition of novel genes and its impact is mainly limited to lower taxonomic levels. Symbioses that generate new phenotypes can also be considered a form of reticulate evolution. Primary endosymbioses with an α-proteobacterium and a cyanobacterium gave rise to mitochondria and plastids, respectively [12], whereas secondary endosymbioses contributed greatly to the evolution of several major eukaryotic groups [13-15]. Such endosymbiotic events are often accompanied by gene transfer from the endosymbiont to the nucleus, a process termed intracellular gene transfer (IGT) [16,17] or endosymbiotic gene transfer [18]. However, the distinction between IGT and HGT is fluid - once an endosymbiont becomes obsolete, the IGTs have to be considered a form of HGT [19].

Apparently, the residence of mitochondria and plastids in eukaryotic cells provides ample opportunities for IGT and this has been supported by several genome analyses [20-23]. On the other hand, the role of HGT in eukaryotic evolution was poorly appreciated until recently. Thus far, an increasing amount of data shows that HGT events do exist in eukaryotes - HGT from prokaryotes to eukaryotes not only is frequent in unicellular eukaryotes of various habitats and lifestyles [24-32], but occurred multiple times in multicellular eukaryotes as well [33-35]. In many cases, acquisition of foreign genes has significantly impacted the evolution of the biochemical system of the recipient organism [24,36].

A critical question regarding the role of HGT is whether and how HGT contributed to the evolution of major eukaryotic groups. Given the scope of HGT in unicellular eukaryotes and that multicellularity is derived from unicellularity, the unicellular ancestors of modern multicellular eukaryotes might have been subject to frequent HGT [37]. Most importantly, the anciently acquired genes, if retained among descendants, are likely to shape the long-term evolution of recipients [37,38]. In this study, we provide an analysis for genes that were introduced to the ancestor of plants (we use the term to denote the taxonomic group Plantae that includes glaucophytes, red algae, and green plants [39,40]). Such an analysis is possible because of the availability of sequence data of *Cyanidioschyzon*, the only red algal species whose nuclear genome has been completely sequenced. Our data indicate that ancient HGT events indeed occurred during early plant evolution and that the vast majority of the acquired genes are related to the biogenesis and functionality of plastids. In light of these findings, we also discuss the implications of concerted gene recruitment as a mechanism for the origin and optimization of key evolutionary novelties in eukaryotes.

## Results

To better understand the scope of HGT, one would like to eliminate complications arising from cases of IGT, in particular those from mitochondria. The ancient origin of mitochondria may translate into difficulties to uncover the α-proteobacterial nature of mitochondrion-derived genes and, therefore, identification of cases of HGT. Because of the ubiquitous distribution of mitochondria in eukaryotes, it is also often difficult to distinguish mitochondrion-derived genes from those transmitted from the ancestral eukaryotic nucleocytoplasm or anciently acquired from other prokaryotes. In this study, we removed genes that potentially are of organellar origin based on sequence comparison, phylogenetic analyses and statistical tests on alternative tree topologies. With only a few exceptions (for example, 2-methylthioadenine synthetase and isoleucyl-tRNA synthetase), anciently acquired genes identified in this study are predominantly found in prokaryotes and photosynthetic eukaryotes, suggesting a likely prokaryotic origin of these genes.

Using PhyloGenie [41], 2,605 trees were generated in the analyses of the *Cyanidioschyzon *genome [42], which were subject to further screening and detailed phylogenetic analyses (see Materials and methods). We previously reported 14 genes anciently acquired from the obligate intracellular bacterial chlamydiae (mostly the environmental *Protochlamydia*) [19] and two other genes, one each from crenarchaeotes and δ-proteobacteria [37]. In this study, an additional 21 anciently acquired genes are reported. Therefore, a total of 37 genes (Table 1; Additional data file 1) have been identified as likely acquired from non-organellar sources prior to the split of red algae and green plants (genome sequences of glaucophytes are not currently available) or earlier. For all these newly reported genes, approximately unbiased (AU) tests [43] for alternative tree topologies representing an organellar origin were performed, and an organellar origin of the subject gene was rejected (*p*-value < 0.05) if no scenario of secondary HGT was invoked. For only a few genes, the scenario of an IGT event in plants followed by secondary HGT to other organismal groups cannot be confidently rejected (Additional data file 1); in these cases, we prefer the simpler scenario of straightforward HGT rather than secondary HGT, based on an assumption that the chance is increasingly rare for the same acquired gene being repeatedly transferred to other organisms. Notably among the newly reported genes, six are related to proteobacteria and two to chloroflexi. The multiplicity of HGT from the same donor groups (for example, proteobacteria) may, in part, have resulted from the over-representation of their genomes in current sequence databases or past physical associations between the donors and the ancestral plant.

**Table 1:** Genes acquired from non-organellar sources prior to the split of red algae and green plants

Gene name	Putative donor	Localization	Putative functions
GCN5-related N-acetyltransferase*	β,γ-Proteobacteria	Cytosol	Arginine biosynthesis
*Glycyl-tRNA synthetase*	Bacteria	Plastid/mitochondria	Translation
*Dihydrodipicolinate synthase *(*dapA*)	γ-Proteobacteria	Plastid	Lysine biosynthesis
*ThiC family protein*	Bacteria	Plastid	Thiamine biosynthesis
*2-C-methyl-D-erythritol 4-phosphate cytidylyltransferase*	Chlamydiae	Plastid	Isoprenoid biosynthesis
*Polynucleotide phosphorylase*	Chlamydiae	Plastid	RNA degradation
*ATP/ADP translocase*^†^	Chlamydiae	Plastid	ATP/ADP transport
*MGDG synthase*^†^	Bacteria	Plastid	Lipid biosynthesis
*Glycerol-3-phosphate acyltransferase*^†^	Chlamydiae	Plastid	Phospholipid biosynthesis
*Alpha amylase*	Chlamydiae	Plastid	Carbohydrate metabolism
*Sodium:hydrogen antiporter*^†^	Chlamydiae	Plastid	Ion transport
*3-Dehydroquinate synthase*	β,γ-Proteobacteria	Plastid	Amino acid biosynthesis
*2-Methylthioadenine synthetase*	Bacteroidetes	Plastid	tRNA modification
*Uroporphyrinogen-III synthase*	Bacteria	Plastid	Porphyrin biosynthesis
*ACT domain-containing protein*^†^	γ-Proteobacteria	Plastid	Amino acid binding
*4-Hydroxy-3-methylbut-2-en-1-yl diphosphate synthase*	Chlamydiae	Plastid	Isoprenoid biosynthesis
*Queuine tRNA-ribosyltransferase*	Chlamydiae	Plastid	tRNA modification
*SAM-dependent methyltransferase*^†^	Bacteria	Cytosol	RNA binding
*Beta-ketoacyl-ACP synthase *(*fabF*)	Chlamydiae	Plastid	Fatty acid biosynthesis
*Semialdehyde dehydrogenase*	α-Proteobacteria	Cytosol	Amino acid metabolism
*Diaminopimelate decarboxylase *(*lysA*)	Bacteria	Plastid	Lysine biosynthesis
*Dihydrodipicolinate reductase *(*dapB*)	Bacteria	Plastid	Lysine biosynthesis
*Aspartate aminotransferase*	Chlamydiae	Plastid	Lysine biosynthesis
*Leucyl-tRNA synthetase*	Bacteria	Plastid/mitochondria	Translation
*Tyrosyl-tRNA synthetase*	Chlamydiae	Plastid/mitochondria	Translation
*Ribosomal protein L11 methyltransferase*	β,γ-Proteobacteria	Cytosol	Amino acid methylation
2-Methylthioadenine synthetase*	Bacteria	Cytosol	tRNA modification
*GTP binding protein, typA*	Chloroflexi	Plastid	Translation elongation
*Cu-ATPase*	Chlamydiae	Plastid	Ion transport
*4-Diphosphocytidyl-2-C-methyl-D-erythritol kinase*	Chlamydiae	Plastid	Isoprenoid biosynthesis
*Enoyl-ACP reductase *(*fabI*)	Chlamydiae	Plastid	Fatty acid biosynthesis
*Histidinol-phosphate transaminase*	Chloroflexi	Plastid	Histidine biosynthesis
Florfenicol resistance protein*	δ-Proteobacteria	Cytosol	Fe-S-cluster binding
*23S rRNA *(*Uracil-5-)-methyltransferase*	Chlamydiae	Plastid	RNA modification
*Topoisomerase 6 subunit B*^†^	Crenarchaea	Cytosol	Protein binding
*tRNA methyltransferase*	Bacteria	Plastid/cytosol	RNA processing
*Isoleucyl-tRNA synthetase*	Bacteria	Cytosol	Translation

*Genes for which plastid-derived homologs already exist in plants. ^†^Genes that likely possessed novel functions and whose homologs are rarely found in cyanobacteria. For all other genes, the possibility of them resulting from displacement of an endogenous homolog cannot be excluded. The putative donors of these genes are determined without invoking secondary HGT events. Alternative explanations for each gene are discussed in the text and Additional data file 1.

**Figure 1:**
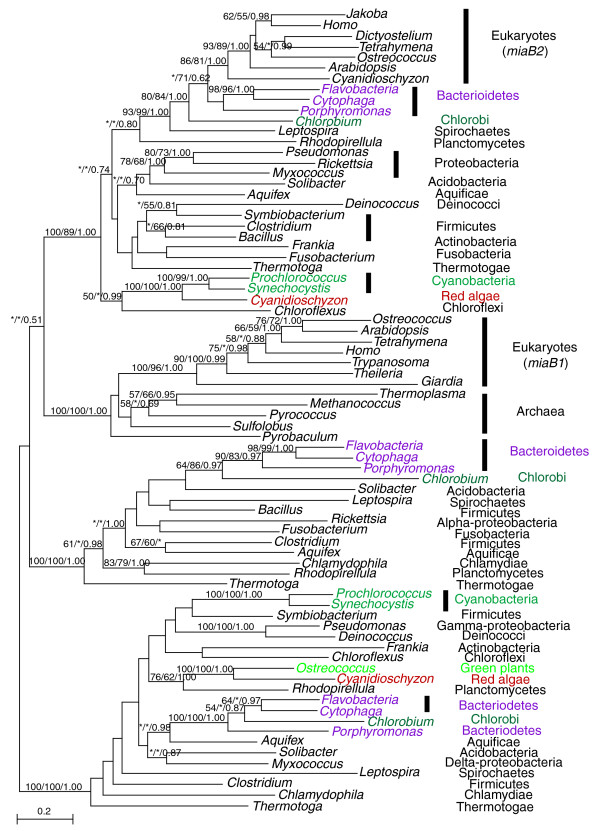
Phylogenetic analyses of 2-methylthioadenine synthetase. The numbers above the branch show bootstrap values for maximum likelihood and distance analyses, and posterior probabilities from Bayesian analyses, respectively. Asterisks indicate values lower than 50%. Colors show taxonomic affiliations.

The dynamics of ancient HGT may be illustrated with the gene encoding 2-methylthioadenine synthetase (*miaB*), a tRNA modification enzyme involved in translation (Figure 1). The evolution of this gene involves gene duplication, transfer, and differential losses. Three versions of this gene exist in bacteria, likely resulting from ancient duplications. Likewise, at least two gene copies (*miaB1*, *miaB2*) are distributed among several major eukaryotic lineages. The eukaryotic *miaB1 *sequences form a monophyletic group with archaeal homologs as expected [44,45]. On the other hand, eukaryotic *miaB2 *sequences and their homologs from bacteroidetes and chlorobi share the highest percent identity (42-45%; using Flavobacteria: ZP_01734273 and *Arabidopsis*: NP_195357 as queries). These sequences cluster together with high support within the otherwise bacterial group. To investigate if *miaB2 *is derived from mitochondria, we performed an AU test on a constraint tree enforcing a monophyly of proteobacterial and *miaB2 *sequences. Results of the AU test suggest that *miaB2 *is not very likely of mitochondrial origin (*p*-value < 0.001). Although the molecular phylogeny of this gene (Figure 1) is theoretically compatible with the scenario of a eukaryotic origin through genome fusion, no current data suggest a bacteriodete or chlorobi partner in the putative ancient fusion event. Therefore, it is more likely that eukaryotic *miaB2 *resulted from an ancient HGT from a bacteroidetes or chlorobi-related organism prior to the divergence of most major eukaryotic lineages. In addition to *miaB1 *and *miaB2*, two other *miaB *copies are also found in plants, one of which is related to cyanobacterial homologs, likely resulting from IGT from plastids, whereas the other copy is related to planctomycete homologs with modest support. Therefore, a total of four copies of the 2-methylthioadenine synthetase gene are found in plants, three of which were likely acquired via independent IGT and ancient HGT events.

**Figure 2:**
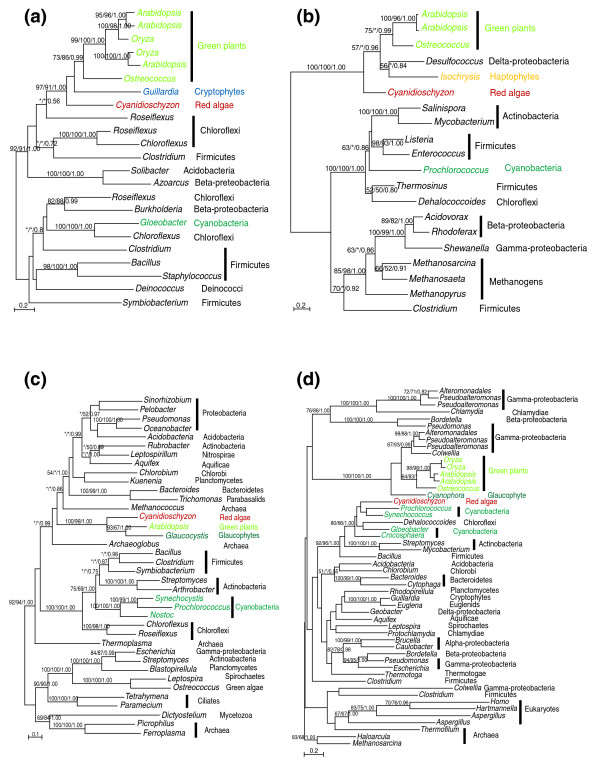
Phylogenetic analyses of anciently acquired genes. Numbers above the branch show bootstrap values from maximum likelihood and distance analyses, and posterior probabilities from Bayesian analyses, respectively. Asterisks indicate values lower than 50%. Colors show taxonomic affiliations. **(a) **MGDG synthase; **(b) **dihydrodipicolinate reductase (*dapB*); **(c) **diaminopimelate decarboxylase (*lysA*); **(d) **dihydrodipicolinate synthase (*dapA*). *DapA*, *dapB *and *lysA *are related to lysine biosynthesis in plants. Please note in (d) that green plant and glaucophyte sequences are of γ-proteobacterial origin whereas the red alga *Cyanidioschyzon *retains the cyanobacterial (plastidic) copy. The *Dehalococcoides *sequence in the cyanobacterial cluster in (d) was likely acquired from cyanobacteria. Another gene (aspartate aminotransferase) related to lysine biosynthesis in plants was likely acquired from chlamydiae [19]. Also see the text and Additional data file 1 for more discussion.

An anciently acquired gene might possess novel functions or merely displace existing homologs (either of eukaryotic or organellar origin) in the recipient. Among the 37 anciently acquired genes identified in our analyses, seven are largely absent from cyanobacteria and other eukaryotes and three already have cyanobacteria-related (or plastid-derived) homologs in plants (Table 1); these genes likely are not derived from homolog displacement. The gene encoding glycerol-3-phosphate acyltransferase (ATS1 and ATS2) has identifiable homologs only in chlamydiae and plastid-containing eukaryotes [19]. Similarly, the gene encoding monogalactosyldiacylglycerol (MGDG) synthases is predominantly found in chloroflexi and firmicutes, with sporadic occurrence in other bacterial groups (including the cyanobacterium *Gloeobacter*). Phylogenetic analyses suggest that plant MGDG synthases are derived from a single HGT event from bacteria, followed by subsequent spread to other photosynthetic eukaryotes (for example, cryptophytes) as well as gene duplication and functional differentiation in flowering plants (Figure 2a).

For the remaining genes, the possibility of them resulting from displacement of existing homologs, especially those that were previously acquired from plastids, cannot be excluded. Notably, at least four of these genes are essential to lysine biosynthesis in plants. The gene encoding aspartate aminotransferase was acquired from a *Protochlamydia*-related organism whereas donors of two other acquired genes, dihydrodipicolinate reductase (*dapB*) and diaminopimelate decarboxylase (*lysA*), cannot be unambiguously determined (Figure 2b,c; Additional data file 1). For another essential gene in lysine biosynthesis, dihydrodipicolinate synthase (*dapA*), sequences from green plants and glaucophytes cluster with γ-proteobacterial homologs, but the cyanobacterial (plastidic) copy is still retained in red algae (Figure 2d). The different evolutionary origins of *dapA *among primary photosynthetic eukaryotes may be explained by a HGT event in the ancestral plant, followed by differential gene losses (that is, displacements of a plastid-derived gene copy in green plants and glaucophytes, or displacement of an HGT-derived gene copy in *Cyanidioschyzon*). It is also theoretically possible that green plants and glaucophytes acquired the gene through independent HGT events, though the chance for closely related taxa acquiring the same gene from the same donor is conceivably lower. A similar scenario has also been observed for several other chlamydiae-related genes involved in isoprenoid and type II fatty acid biosyntheses [19,46].

## Discussion

### Scope of ancient HGT

We use the term HGT loosely in this study for any transfer events from non-organellar sources. Although the timing of HGT cannot be accurately calibrated in most cases, it can be inferred based on gene distribution in the recipient lineage. If the acquired gene is found in most taxa of a major lineage, it is likely that the gene was acquired prior to the divergence of the lineage. Given the paucity of sequence data from representatives of many major eukaryotic groups and the lack of consensus on eukaryotic phylogeny [47], identification of ancient HGT often becomes more difficult as phylogenetic depth increases.

A major issue related to the role of HGT in macroevolution is the scale of ancient HGT. Our analyses identified 37 anciently acquired genes in plants that account for 1.42% (37/2,605) of all generated gene trees (Table 1; Additional data file 1). It should be cautioned that HGT identification is affected by many factors, in particular taxonomic sampling, method of analysis, complications arising from IGT, and lineage-specific gains or losses (see [37,48,49] for more discussions). For studies based on phylogenetic approaches, long-branch attraction arising from biased sequence data is also a particular concern [50,51]. Additionally, if the α-proteobacterial or the cyanobacterial nature of IGT-derived genes has been erased, due to either frequent HGT among prokaryotes or the loss of phylogenetic signal over time, these genes will not be properly identified and may be mistaken as HGT-derived. It should also be noted that this study is based on the genome analyses of the red alga *Cyanidioschyzon*, which inhabits an extreme environment in acidic hot springs and maintains a streamlined genome [41]. Some anciently acquired genes might have been lost from the *Cyanidioschyzon *genome, but are retained in other red algal species. This could potentially underestimate the HGT frequency in plants. With the rapid accumulation of sequence data, in particular those from other red algae and under-represented eukaryotic groups, a broader taxonomic sampling will be possible and the number of anciently acquired genes identified in the plant lineage will likely change. Therefore, the data presented in this study should only be interpreted as our current understanding of the scale of ancient HGT, rather than an exhaustive list of all anciently acquired genes in plants.

Despite the difficulties in HGT identification, the multiple introductions of the same gene from various prokaryotic sources (for example, 2-methylthioadenine synthetase; Figure 1) suggest that HGT is a continuous and dynamic process. Given that phylogenetic signal tends to become obscure over time and that eukaryote-to-eukaryote transfer, which has been recorded in multiple studies [52,53], is largely not covered in this study, it is possible that the identified genes in our analyses represent only the tip of an iceberg for the overall scope of ancient HGT in eukaryotes. In particular, during early eukaryotic evolution when the ancestral nucleocytoplasmic lineage emerged from prokaryotes (either by a split from archaea or by fusion of archaeal and bacterial partners) and began to diverge into extant groups, these early eukaryotes might bear more biochemical and physiological similarities to their prokaryotic relatives. Because HGT tends to occur among organisms of similar biological and ecological characters [54], the barriers to interdomain gene transfer during early eukaryotic evolution might not be as significant as observed today. Therefore, although our data suggest that HGT indeed existed in early plant evolution, many other anciently acquired genes in plants might have escaped our detection because of the limitations of current phylogenetic approaches. These genes might have shaped the genome composition of the recipient lineages and may also be, in part, responsible for the lack of resolution of relationships among major eukaryotic groups [40,47].

### Functional recruitment and plant adaptation

A significant insight from prokaryotic genome analyses is the role of HGT in microbial adaptation. By acquiring ready-to-use genes from other sources, HGT avoids a slow process of gene generation and might confer to the recipient organisms immediate abilities to explore new resources and niches [55-57]. This may be crucial for organisms inhabiting shifting environments, where acquisition of beneficial genes from local communities is necessary for recipient organisms to avoid extinction or to optimize their adaptation. Therefore, lineage continuity and ecological stability can be achieved by increasing the genetic repertoire through recruitment of foreign genes.

An acquired gene may be novel to the recipient or homologous to an endogenous copy. In the latter case, the newly acquired homolog may be retained (for example, 2-methylthioadenine synthetase; Figure 1) and the acquisition of an additional gene copy will provide opportunities for functional differentiation and enriches the genetic repertoire of the recipient. Although all acquired genes affect genome composition and evolution, only those that potentially provide new functions will most likely induce biochemical or phenotypic changes, and consequently adaptation in recipient organisms. Some anciently acquired novel genes identified in our analyses appear to be critical for plant development or adaptation. For example, the gene encoding topoisomerase VI beta subunit (TOP6B) in plants was likely acquired from a crenarchaeote [37]. TOP6B in green plants is required for endoreplication, a process of DNA amplification without cell division and a mechanism to increase cell size in plants. *Top6b *mutants display extreme dwarf phenotypes (about 20% the height of wild types), chloroplast degradation, and early senescence [58-60].

Several other novel genes are functionally related to the biogenesis and development of plastids. These include genes acquired from different bacterial groups. For example, MGDG synthases are responsible for the generation of MGDG, a major lipid component of plant photosynthetic tissues. MGDG synthases appear to be encoded by a single-copy gene in red and green algae, but three copies exist in *Arabidopsis *and they are further classified into two types (type A, including MGD1, and type B, including MGD2 and MGD3). In *Arabidopsis*, MGD1 is localized in the inner membrane of chloroplasts and it is responsible for the majority of MGDG biosynthesis. No *mgd1 *null mutants are found in *Arabidopsis*, suggesting that MGD1 is essential for chloroplast development and plant growth [61]. In contrast, MGD2 and MGD3 are highly expressed in non-photosynthetic tissues and likely provide an alternative route for MGDG biosynthesis under phosphate starvation conditions [61-63]. Therefore, ancient HGT, gene duplication and subsequent functional differentiation provide a mechanism for specialized MGDG production in different tissues and growing conditions. As another example, knocking down the expression of the chlamydiae-related ATS1 and ATS2 in *Arabidopsis *will lead to small, pale-yellow plants, suggesting that the chloroplast development has been seriously impeded [64].

### Homolog displacement

Not all acquired genes may bring new biochemical functions to the recipient organism. The acquired gene may displace the existing homolog and, if they are functionally equivalent, the impact of gene transfer on the adaptation of the recipient may be limited. Such homolog displacement may be considered selectively neutral [65,66], though their contributions to genome evolution should not be ignored.

Although the role of HGT in eukaryotic evolution is gaining increasing appreciation, there are very few studies available on the number of acquired genes resulting from homolog displacement without introducing new functions. According to the gene transfer ratchet mechanism proposed by Doolittle [67], homolog displacement might be pervasive in unicellular eukaryotes and bacterial genes, either intracellularly or horizontally derived, may gradually replace all endogenous copies over time. Although our analyses only address anciently acquired genes prior to the split of red algae and green plants, homolog displacement indeed appears to be frequent compared to the acquisition of genes with novel functions. For example, at least three genes encoding organellar aminoacyl-tRNA synthetases (that is, leuRS, tyrRS, and ileRS) were likely acquired from other prokaryotic sources (Table 1; Additional data file 1). These aminoacyl-tRNA synthetases are often shared by both mitochondria and plastids [68], suggesting that both plastidic and mitochondrial aminoacyl-tRNA synthetases might have been frequently displaced in plant evolution.

It should be noted that the displacement of aminoacyl-tRNA synthetases is relatively easy to identify because these genes have low substitution rates and they are universally present in all organisms [38,69-72]. Many other cases of homolog displacement may not be as easily detected because of complications arising from possible independent gene losses/gains or lack of phylogenetic information retained in the acquired gene [37,65]. In our analyses, homologs for most identified genes can be found in multiple extant cyanobacteria. Given the cyanobacterial origin of plastids, a cyanobacterial copy of these genes might have existed when the plastids were first established; therefore, an IGT event and subsequent displacement of the original plastidic genes by later non-cyanobacterial homologs cannot be excluded, though such a scenario is highly unlikely to have occurred to all these genes. Overall, our data show that many acquired genes may have resulted from homolog displacement without introducing new functions, suggesting that the number of acquired genes does not predict the role of HGT in the adaptation of recipient organisms. It is unclear whether such a gene displacement pattern also exists in non-photosynthetic eukaryotes.

### Concerted gene recruitment and the origin of evolutionary novelties

Plastids are the key evolutionary novelty that defines photosynthetic eukaryotes. Aside from photosynthesis, some other important biochemical activities, including biosyntheses of fatty acids and isoprenoids, are also carried out in plastids. Intriguingly, over 78% (29/37) of the anciently acquired genes identified in our analyses are either predicted or experimentally determined to be related to the biogenesis and functionality of plastids (Table 1); these include genes possessing novel functions and those resulting from homolog displacement. Because of the extremophilic lifestyle of *Cyanidioschyzon *and its streamlined genome, some acquired genes related to non-photosynthetic activities might have been eliminated from the genome. It remains to be investigated whether such a high density of acquired genes that are functionally related to plastids also exists in other photosynthetic eukaryotes, including mixotrophs and those inhabiting broader niches. Nevertheless, given the total number of these plastid-related genes identified in our analyses, it appears that concerted gene recruitment from multiple sources or selective retention of the acquired genes occurred to optimize the functionality of plastids during early plant evolution. The observation that some independently acquired bacterial genes are functionally related to plastids has also been reported in the chlorarachniophyte *Bigelowiella natans*, which contains plastids derived from a secondary endosymbiont [21].

This phenomenon of concerted gene recruitment for the origin and optimization of key evolutionary novelties of the recipient also exists in other eukaryotic groups. In the protozoan group diplomonads, about half (7/15) of the acquired genes are related to the anaerobic lifestyle of the organisms. These genes were interpreted to have been acquired from various organisms, including other eukaryotes, and might be responsible for the lifestyle transition from aerobes to anaerobes in diplomonads [24]. Another example is related to ciliates that live in the rumen of herbivorous animals. In this case, over 140 genes were transferred from diverse bacterial groups to rumen ciliates, the vast majority of which are related to degradation of carbohydrates derived from plant cell walls [30]. A third example is the evolution of nucleotide biosynthesis in the apicomplexan parasite *Cryptosporidium*, where two independently acquired genes, one each from γ- and ε-proteobacteria, and likely two other plant-like genes facilitated the establishment of salvage nucleotide biosynthetic pathways [36,73], allowing the parasite to obtain nucleotides from their hosts. Therefore, concerted recruitment or selective retention of foreign genes apparently is not a unique phenomenon in the origin and optimization of evolutionary novelties of unicellular eukaryotes. In the case of plants, ancient endosymbioses and HGT events in concert drove the establishment of plastids. In the cases of diplomonads, rumen ciliates and *Cryptosporidium *parasites, multiple independent HGTs from other organisms contributed to the major lifestyle transitions in the recipient organisms. In all these cases, the origin of evolutionary novelties may be viewed as a result of gene sharing with other organisms.

Although the current data suggest that HGT events are frequent in unicellular eukaryotes [21,24,26,30], how and to what degree they have affected the evolution of the recipients remain largely unclear. An interesting observation from the studies of HGT in eukaryotes is that the vast majority of well-documented cases involve prokaryotes as donors [26,30,31]. Given the ubiquitous distribution of prokaryotes and their greater species and metabolic diversity, the gene pool of prokaryotes conceivably was significantly larger than that of eukaryotes, in particular during early eukaryotic evolution. Therefore, it is interesting to speculate whether early eukaryotes continuously obtained genes from a larger prokaryotic gene pool [67], either individually or occasionally in large chunks, through HGT events in response to the environment, as we have now observed in many prokaryotes and unicellular eukaryotes. Such changes in genetic background and biochemical system would likely induce shifts in ecology, physiology, morphology or other traits of the recipient lineage. Concerted gene recruitment in plants, diplomonads, rumen ciliates, *Cryptosporidium *parasites and possibly many other organisms suggests that independently acquired genes are able to generate and optimize key evolutionary novelties in recipient organisms. Whether such ancient gene recruitment events and the novelties they generated were ultimately responsible for the emergence and adaptive radiation of some major eukaryotic groups warrants further investigations.

## Conclusion

Phylogenetic analyses, sequence comparisons, and statistical tests indicate that at least 1.42% of the genome of the red alga *Cyanidioschyzon *is derived from ancient HGT events prior to the split of red algae and green plants. Although many acquired genes may represent displacement of existing homologs, other genes introduced novel functions essential to the ancestor of red algae and green plants. The vast majority of the anciently acquired genes identified in our analyses are functionally related to plastids, suggesting an important role of concerted gene recruitment in the generation and optimization of major evolutionary novelties in some eukaryotic groups.

## Materials and methods

### Data sources

Protein sequences for the red alga *Cyanidioschyzon merolae *were obtained from the *Cyanidioschyzon *Genome Project [42,74]. Expressed sequence tag (EST) sequences were obtained from TBestDB [75] and the NCBI EST database. All other sequences were from the NCBI protein sequence database.

### Identification of ancient HGT

Anciently acquired genes in this study include those horizontally acquired prior to the split of red algae and green plants. A list of ancient HGT candidates was first generated based on phylogenomic screening of the *Cyanidioschyzon *genome using PhyloGenie [41] and the NCBI non-redundant protein sequence database. The vast majority of the genes on this list are predominantly identified in bacteria and archaea, and therefore are likely of prokaryotic origin. To reduce the complications arising from potential cases of IGT, we adopted an approach combining sequence comparison, phylogenetic analyses, and statistical tests. Each gene on the list was first used to search the NCBI protein sequence database. Because of the cyanobacterial origin of plastids and the α-proteobacterial origin of mitochondria, genes with cyanobacterial and plastid-containing eukaryotic homologs as top hits were considered as likely plastid-derived; those with α-proteobacterial and other eukaryotic homologs as top hits were considered as likely mitochondrion-derived. These potentially organelle-derived genes were removed from the candidate list and the remaining genes were subject to detailed phylogenetic analyses. Gene tree topologies generated through detailed phylogenetic analyses were subject to careful inspections; any genes that formed a monophyly with cyanobacterial and plastid-containing eukaryotic homologs or with proteobacterial and other eukaryotic sequences were also eliminated from further consideration. Additionally, alternative topologies representing various evolutionary scenarios for each gene were statistically evaluated based on AU tests [43]. Genes for which a straightforward IGT scenario (versus IGT followed by secondary transfers) could not be rejected (*p*-value > 0.05) were also removed from the HGT candidate list. For a few genes, the gene tree topology may be explained by either a straightforward HGT or an IGT followed by secondary HGT events to other organisms; we prefer the scenario of straightforward HGT in these cases to that of secondary HGT, based on an assumption that chances for the same gene being repeatedly transferred among different organismal groups are relatively rare. In several other cases (for example, Figures 1 and 2d), the distribution of the subject gene may also be explained by either multiple independent HGT events or a single HGT followed by differential gene losses. In such cases, we prefer the gene loss scenario based on an assumption that independent acquisitions of the same gene, by closely related taxa, from the same donor are rare. Because identification of HGT heavily relies on an accurate organismal phylogeny and because the relationships among many major eukaryotic lineages remain unsolved [40,47], HGT events among eukaryotes were not included in our analyses in most cases, except for those between photosynthetic eukaryotes where secondary or tertiary endosymbioses and subsequent gene transfer to host cells have been frequently documented [21,26,76].

### Detailed phylogenetic analyses

Sequences were sampled from representative groups (including major phyla of bacteria and major groups of eukaryotes) within each domain of life (bacteria, archaea, and eukaryotes). Because of the potential for sequence contaminations, eukaryotic EST sequences whose authenticity is suspicious (for example, high nucleotide sequence percent identity with bacterial homologs and/or absence of homologs from genomes of closely related taxa) were not included in the analyses. Multiple protein sequence alignments were performed using MUSCLE [77] and clustalx [78], and only unambiguously aligned sequence portions were used. Such unambiguously aligned positions were identified by cross-comparison of alignments generated using MUSCLE and clustalx, followed by manual refinement. The alignments are available in Additional data file 1. Phylogenetic analyses were performed with a maximum likelihood method using PHYML [79], a Bayesian inference method using MrBayes [80], and a distance method using the program *neighbor *of PHYLIP version 3.65 [81] with maximum likelihood distances calculated using TREE-PUZZLE [82]. All maximum likelihood calculations were based on a substitution matrix determined using ProtTest [83] and a mixed model of four gamma-distributed rate classes plus invariable sites. Maximum likelihood distances for bootstrap analyses were calculated using TREE-PUZZLE [82] and PUZZLEBOOT v1.03 (by Michael E Holder and Andrew J Roger, available on the web [84]). Branch lengths and topologies of the trees depicted in all figures (Figures 1 and 2; Additional data file 1) were calculated with PHYML. For the convenience of presentation, gene trees were rooted using archaeal (or archaeal plus eukaryotic) sequences, or paralogous gene copies if ancient gene families were involved, as outgroups; otherwise, trees were rooted in a way that no top hits of the sequence similarity search were used as an outgroup. Nevertheless, all gene trees should be strictly interpreted as unrooted.

### AU tests on alternative tree topologies

Following detailed phylogenetic analyses, alternative tree topologies for each remaining HGT candidate were assessed for their statistical confidence using Treefinder [85]. In most cases, multiple constraint trees for each HGT candidate were generated using Treefinder by enforcing: monophyly of all eukaryotic sequences; monophyly of cyanobacterial, plant and other plastid-containing eukaryotic sequences; and monophyly of cyanobacterial, plant, and closely related bacterial sequences. These alternative topologies assumed that the subject gene in plants is not HGT-derived; they served as null hypotheses that all eukaryotic sequences have the same eukaryotic or mitochondrial origin or that plants acquired the subject gene from plastids, sometimes followed by secondary HGT to other bacterial groups. AU tests, which have been recommended for general tree tests [43], were performed on alternative tree topologies (non-HGT hypotheses) and the tree generated from detailed phylogenetic analyses (HGT hypothesis). In this study, topologies with a *p*-value < 0.05 were rejected.

### Prediction of protein localization

Targeting signal of identified protein sequences was predicted using ChloroP [86] and TargetP [87]. Additional information about protein localization in green plants was obtained from The *Arabidopsis *Information Resource (TAIR).

## Abbreviations

ATS, glycerol-3-phosphate acyltransferase; AU, approximately unbiased; EST, expressed sequence tag; HGT, horizontal gene transfer; IGT, intracellular gene transfer; MGDG, monogalactosyldiacylglycerol; TOP6B, topoisomerase VI beta subunit.

## Authors' contributions

JH conceived the study, performed the data analyses, and drafted the manuscript. JPG participated in data interpretation and manuscript writing. Both authors read and approved the final manuscript.

## Additional data files

The following additional data are available. Additional data file 1 contains protein sequence alignments used for phylogenetic analyses, resulting gene trees, tree interpretations, and AU tests on alternative topologies.

## Supplementary Material

Additional data file 1 Each sequence name includes a GenBank GI number followed by the species name. Click here for file
