# Supplementary material for: Concerted gene recruitment in early plant evolution
Source: Genome Biol. 2008 Jul 8;9(7):R109. doi: 10.1186/gb-2008-9-7-r109 (PMC2530860; doi:10.1186/gb-2008-9-7-r109)
Supplement: Additional data file 1 — Each sequence name includes a GenBank GI number followed by the species name. [file gb-2008-9-7-r109-S1.pdf]

Concerted gene recruitment in early plant evolution

Jinling Huang J. Peter Gogarten

1. Materials and methods

Data sources: Protein sequences for the red alga *Cyanidioschyzon merolae* were obtained from the *Cyanidioschyzon* Genome Project [41, 73]. EST sequences of several protists were obtained from TBestDB [74]. All other sequences were from the NCBI protein sequence database.

Identification of ancient HGT: Anciently acquired genes in this study include those horizontally acquired prior to the split of red algae and green plants. A list of ancient HGT candidates was first identified based on phylogenomic screening of the *Cyanidioschyzon* genome using PhyloGenie and the NCBI non-redundant protein sequence database. The vast majority of the genes on this list are predominantly identified in bacteria and archaea, and therefore are likely of prokaryotic origin. To reduce the complications arising from potential cases of IGT, we adopted an approach combining sequence comparison, phylogenetic analyses, and statistical tests. Each gene on the list was first used to search the NCBI protein sequence database. Because of the cyanobacterial origin of plastids and the α-proteobacterial origin of mitochondria, genes with cyanobacterial and plastid-containing eukaryotic homologs as top hits were considered as likely plastid-derived; those with proteobacterial and other eukaryotic homologs were considered as likely mitochondrion-derived. These potentially organelle-derived genes were removed from the candidate list and the remaining genes were subject to detailed phylogenetic analyses (see below). Gene tree topologies generated through detailed phylogenetic analyses were subject to careful inspections; any genes that formed a monophyly with cyanobacterial homologs or with proteobacterial and other eukaryotic sequences were also eliminated from further consideration. Additionally, alternative topologies representing various evolutionary scenarios for each gene were statistically evaluated based on AU tests [43] (see below); genes for which a straightforward IGT scenario (versus IGT followed by secondary transfers) could not be rejected (p-value > 0.05) were also removed from the HGT candidate list.

Detailed phylogenetic analyses: Sequences were sampled from representative groups (including major phyla of bacteria and major groups of eukaryotes) within each domain of life (bacteria, archaea, and eukaryotes). Because of the potential for sequence contaminations, eukaryotic EST sequences whose authenticity is suspicious (e.g., high nucleotide sequence percent identity with bacterial homologs and/or absence of homologs in genomes of closely related taxa) were not included in the analyses. Multiple protein sequence alignments were performed using MUSCLE [77] and clustalx [78], followed by cross-comparisons and manual refinement. Only unambiguously aligned sequence portions were used. Phylogenetic analyses were performed with a maximum likelihood method using PHYML [79], a Bayesian inference method using MrBayes [80], and a distance method using the program *neighbor* of PHYLIP version 3.65 [81] with maximum likelihood distances calculated using TREE-PUZZLE [82]. All maximum likelihood calculations were based on a substitution matrix determined using ProtTest [83] and a mixed model of 4 gamma-distributed rate classes plus invariable sites.

Maximum likelihood distances for bootstrap analyses were calculated using TREE-PUZZLE and PUZZLEBOOT v1.03 (by Michael E. Holder and Andrew J. Roger, available on the web [84]). Branch lengths and topologies of the trees depicted in all figures were calculated with PHYML. For the convenience of presentation, gene trees were rooted using archaeal (or archaeal + eukaryotic) sequences, or paralogous gene copies if ancient gene families were involved, as outgroups; otherwise, trees were rooted in a way that no top hits of the sequence similarity search were used as an outgroup. Nevertheless, all gene trees should be strictly interpreted as unrooted.

AU tests on alternative tree topologies: Following detailed phylogenetic analyses, alternative tree topologies for each remaining HGT candidates were assessed for their statistical confidence using Treefinder [85]. In most cases, multiple constraint trees were generated using Treefinder for each HGT candidate by enforcing **a**) monophyly of all eukaryotic sequences, **b**) monophyly of cyanobacterial, plant and other plastid-containing eukaryotic sequences, and **c**) monophyly of cyanobacterial, plant, and closely related bacterial sequences. These alternative topologies assumed that the subject gene in plants are not HGT-derived; they served as null hypotheses that all eukaryotic sequences have the same eukaryotic or mitochondrial origin or that plants acquired the subject gene from plastids, sometimes followed by secondary HGT to other bacterial groups. AU tests, which have been recommended for general tree tests, were performed on alternative tree topologies (non-HGT hypotheses) and the tree generated from detailed phylogenetic analyses (HGT hypothesis). In this study, topologies with a p-value < 0.05 were rejected.

Prediction of protein localization: Targeting signal of identified protein sequences was predicted using ChloroP [86] and TargetP [87]. Additional information about protein localization in green plants was obtained from The *Arabidopsis* Information Resource (TAIR).

2. Protein sequence alignment used for phylogenetic analyses and resulting phylogenetic trees. Each sequence name includes a GI number from GenBank (or ID number from other databases) followed by species name. Numbers above the branches of the gene tree show bootstrap support values for maximum-likelihood analyses and distance analyses, and posterior probability from Bayesian analyses, respectively. Asterisks indicate support values below 50%. N denotes genes whose homologs are rarely found in cyanobacteria and that likely possessed novel functions; E denotes genes for which plastid-derived homologs already exist in plants; D denotes genes for which a possible replacement of an endogenous homolog cannot be excluded.

---

1. GCN5-related N-acetyltransferase (E)

```
CLUSTAL X (1.83.1) multiple sequence alignment


32444545_Rhodopirellula_baltic      KADTLIEAMGWIRRFRGKTTVIKLGGSLLEDREALQHLLLDVIFMETVGL
21541979_Mycobacterium_leprae       KAEVLAEALPWLKQLHGKVVVVKYSGNAMTDDMLRRAFAADMAFLRNCGI
48834901_Thermobifida_fusca         KAKTLIEALPWLSALHGKTVVVKYGGNAMTDPDLRARFAENIVFLRYAGL
7106696_Streptomyces_coelic         KAQILIEALPWLTRHHGKTVVIKFGGNAMIDEDLKAAFAQDVVFLRHAGL
30409279_Cyanidioschyzon_merol      RVQVLTEALPYIQKWRNEIMVIKYGGAVVKQD---ADIIKDILFLTCCGF
```

```
21673937_Chlorobium_tepidu          ---MLVEALPYIRKFEGKTFVIKYGGAAMKDEVLKNIFAENVTLLRKVGI
11498879_Archaeoglobus_fulgid       NVELLIEALPYIKDFHSTTMVIKIGGHAMVNDRILEDTIKDIVLLYFVGI
48841339_Methanosarcina_barker      RENVLIEALPYMQEFYDSIMVIKVGGNAMVSAQIMEDIIKDIVLLRYVGI
45655673_Leptospira_interr          RVNYILEALPYITQYSGKTVVIKYGGAAMAKADLKESFAKDIVLLKYVGI
33237476_Prochlorococcus_marin      RVSVLSEALPYIQKFAGRRIVIKYGGSAMSKESLKEAVFRDIALLSSVGA
6706427_Arabidopsis_thalia          RVEILSESLPFIQKFRGKTIVVKYGGAAMTSPELKSSVVSDLVLLACVGL
50911447_Oryza_sativa               RVDVLSEALPFIQRFRGKTVVVKYGGAAMKSPELQSSVIRDLVLLSCVGL
50927665_Oryza_sativa               RVDVLSEALPFIQRFKGKTVVVKYGGAAMKSPELQASVIRDLVLLSCVGL
35214504_Gloeobacter_viola          RVQVLAEALPYIQRFAGKTIVVKYGGAAMVKENLRDLVIRDIVFLATVGI
67920085_Crocosphaera_watsonii      RVRVLSEALPYIQQFAGRTFVIKYGGAAMKDSSLKDKVIRDIIFLACVGV
48764058_Rhodospirillum_rubrum      KAKTLSEALPYMRRFAGLTIVIKYGGHAMGDPELAKTFARDVVLLKQVGM
28867322_Pseudomonas_syring         VAKVLSEALPYIRRFVGKTLVIKYGGNAMESEELKTGFARDIVLMKAVGI
23102640_Azotobacter_vinela         VAQVLSEALPYIRRFVGKTLVIKYGGNAMESDELKTGFARDIVLMKAVGI
46311045_Burkholderia_cepaci        KAEILAEALPYIRRYHGKTVVIKYGGNAMTEERLKQGFARDVILLKLVGI
17427041_Ralstonia_solana           KAEILAQALPYIRKFHGKTIVIKYGGNAMTEEKLKHGFARDVILLKLVGM
52007208_Thiobacillus_denitr        KAYILSEALPYIQRFYDKTIVIKYGGNAMTERHLMEAFAKDVVLLKLVGM
30249008_Nitrosomonas_europa        KVNILAEALPYIRRFHDKTIVIKYGGNAMIEEALKQGFARDVVLLKLVGM
53759351_Methylobacillus_flage      KAQTLAEALPYIKRFFDKTIVIKYGGNAMTDPHLKECFASDVVLLKLVGM
15607035_Aquifex_aeolic             ---------------------MIKYGGSAMHDEELRESFARDVVLLKYVGI
39995261_Geobacter_sulfur           KANTLMEALPYIRRFSGKTIVIKYGGHAMADEALKESFALDVIMLKSLGI
50875328_Desulfotalea_psychr        RAKVLIESLPYMQEFRHKTIVIKYGGHAMVDEALKKQFALDVILLKQIGI
CM204_Cyanidioschyzon_merolae       FVAFLRNCSPYIYAHRNKTFVIHIPGQMLLDRALFESFMQDVALIKSLGV
42573209_Arabidopsis_thalia         FVRWFREAWPYLWAHRGCTFVVIISGEIIAGSSC-DAILKDIAFLHHLGI
18420097_Arabidopsis_thalia         FVRWFREAWPYLWAHRGCTFVVIISGEIIAGSSC-DAILKDIAFLHHLGI
CDO00012939_Cyanophora_paradox      --------------------------------------------------
28867555_Pseudomonas_syring         YVNWLRHASPYINAHRDCTFVVMLPGDGVAHPNF-GNIVHDLVLLHSLGV
23103255_Azotobacter_vinela         YVNWLRHASPYINAHRDCTFVVMLPGEGIEHPNF-GNIVHDLVLLHSLGV
27361298_Vibrio_vulnif              LVKGFRQSTPYVNAHRGKTMVILLGGEAIADKNF-SNIINDIALMHSLGV
26249247_Escherichia_coli           LVEGFRHSVPYINTHRGKTFVIMLGGEAIEHENF-SSIVNDIGLLHSLGI
46312172_Burkholderia_cepaci        FVDWMRSVAPYIHKFRNSTFVVGFGGEVVQQGLL-NALVSDIALLQAMGI
17428264_Ralstonia_solana           FVDWLRAVAPYIHAFRDKTFVIGFGGELVKAGML-GALVNDIALLHAMGM
30248782_Nitrosomonas_europa        FVTWFRSVTPHIHASHGKVFVIAFGGEVVENGKF-VELVQDFNLLASLGI
53759353_Methylobacillus_flage      FVRWFRSAAPYIHAFGGRTFVIAFGGEVVDDGQF-VALSHDLNLLASLDV
52007496_Thiobacillus_denitr        FVHWFRSAAPYIHGFRGKTFVIAFGGELVAEGGF-VQLAHDVNLLNSLGV
```

```
32444545_Rhodopirellula_baltic      RPVVVHGGGKAITEAMAKAGIEAQFIRGRRVTDEKSLEVVEQVLAGELNV
21541979_Mycobacterium_leprae       HPVVVHGGGPQITAMLRRLGIADDFKGGFRVTTPEVLDVARMVLFGQVGR
48834901_Thermobifida_fusca         RPVVVHGGGPQINAHLERLGVESTFTAGLRVTTPETMEIVRMVLVGQVNR
7106696_Streptomyces_coelic         KPVVVHGGGPQISAALDKHGIVSEFKAGLRVTTEDAMDVVRMVLAGQVQR
30409279_Cyanidioschyzon_merol      QIVVVHGGGPLINEWLKQLNKSPQYWEGIRVTDKVTMEIVEMVLAGKVNK
21673937_Chlorobium_tepidu          KVVIVHGGGDAITKTSAKLGLETTFVHGKRVTDRQTVDVIQMTLAGKVNQ
11498879_Archaeoglobus_fulgid       KPVVVHGGGPEISEKMEKFGLKPKFVEGLRVTDKETMEVVEMVLDGKVNS
48841339_Methanosarcina_barker      KPVIVHGGGPEITEKMGRMGKKAEFFQGLRITDDETMEIAKMVLVGNINT
45655673_Leptospira_interr          HPVIVHGGGPEINRLLDNLKIPTEFVHGHRVTDNQTMEIVEMVLTGKVNK
33237476_Prochlorococcus_marin      QPVIIHGGGPEINHWLTKLEIKSEFRDGLRITDSNTMDIVEMVLIGRVNK
6706427_Arabidopsis_thalia          RPILVHGGGPDINRYLKQLNIPAEFRDGLRVTDATTMEIVSMVLVGKVNK
50911447_Oryza_sativa               RPVLVHGGGPEINSWLGRVGVEPQFRNGLRVTDALTMEVVEMVLVGKVNK
50927665_Oryza_sativa               HPVLVHGGGPEINSWLLRVGVEPQFRNGLRVTDALTMEVVEMVLVGKVNK
35214504_Gloeobacter_viola          KPVVVHGGGPEINSWLERLNIPVQFVGGLRVTDKITMEVVEMVLAGKVNK
67920085_Crocosphaera_watsonii      HPVVVHGGGPEINSWLGKLGIEPQFKDGLRVTDAATMDVVEMVLVGRVNK
48764058_Rhodospirillum_rubrum      NPVVVHGGGPQIGRMLDTLRIQSTFIDGLRVTDAATVDVVEMVLAGSINK
28867322_Pseudomonas_syring         NPVVVHGGGPQIGDLLKRLSIESHFIDGMRVTDAQTMDVVEMVLGGQVNK
23102640_Azotobacter_vinela         HPVVVHGGGPQIGDLLKRLSIESRFVDGMRVTDAQTMDVVEMVLGGQVNK
46311045_Burkholderia_cepaci        NPVIVHGGGPQIDHALKKIGKAGTFIQGMRVTDEETMEVVEWVLGGEVQQ
17427041_Ralstonia_solana           NPVVVHGGGPQIDDALKKVGKTGTFIQGMRVTDEETMEVVEWVLGGEVQQ
52007208_Thiobacillus_denitr        NPVVVHGGGPQIAGLLQRIGKQSEFIQGMRVTDEETLDVVEMVLGGLVNQ
30249008_Nitrosomonas_europa        NPVIIHGGGPQIDHMLKRVGKEGVFIQGMRVTDAETMDIVEMVLGGLINK
53759351_Methylobacillus_flage      NPVVVHGGGPQINELLDKLGKKGEFIQGMRVTDEETMDIVEMVLGGQVNK
15607035_Aquifex_aeolic             NPVIVHGGGPQISKTLEKFGIKPKFVGGMRKTDEETMHVVEMVLSGDINK
39995261_Geobacter_sulfur           NPVVVHGGGPQINETLKRYGIVSEFVKGMRVTDAATMQVVEMVLTGQVNK
50875328_Desulfotalea_psychr        NPIIVHGGGPQINNLLDRLDIKPSYVQGMRVTDGETMDVVEMVLVGKVNK
CM204_Cyanidioschyzon_merolae       RVILVGGCRPQVDRALERYGIQQRFVRGTRISSPEVMQAVLEA-SGYVRF
42573209_Arabidopsis_thalia         RFVLVPGTQEQIDQLLAERGREATYVGRYRVTDAASLQAAKEA-AGAISV
18420097_Arabidopsis_thalia         RFVLVPGTQEQIDQLLAERGREATYVGRYRVTDAASLQAAKEA-AGAISV
CDO00012939_Cyanophora_paradox      --------------------------------------------------
28867555_Pseudomonas_syring         RLVLVHGSRPQIESRLAQRGITPRYHRDMRITDTETLECVIDA-VGQLRI
23103255_Azotobacter_vinela         RLVLVHGSRPQIEARLAARGLTPRFHQNLRITDVPTLECVIDA-VGSLRL
27361298_Vibrio_vulnif              KVVLVYGARPQINQLLDKQSSQTPYHKHIRVTDENSLSIVMQA-AGQLQL
26249247_Escherichia_coli           RLVVVYGARPQIDANLAAHHHEPLYHKNIRVTDAKTLELVKQA-AGTLQL
46312172_Burkholderia_cepaci        QIVLVHGSRPQVEEQLSLHGVESEFSHGLRITDARALESAKEA-AGEVRL
17428264_Ralstonia_solana           HIVLVHGSRPQVEEQLALRHVQTQFVDGIRVTDNAALESAKEA-SGELRL
30248782_Nitrosomonas_europa        QLVLVHGARPQIESRLRANQQEMSYVQGMRVTDAATLQCVKEA-VGKVRV
```

```
53759353_Methylobacillus_flage      RLVLVHGARPQIERRLRRAKLQTKLVNGLRVTDDEAMEAVKEA-NGAVRV
52007496_Thiobacillus_denitr        RLVLVHGVRPQVEARLTENGTDIRYVSGLRVTDDAALACVKEA-AGTVRV


32444545_Rhodopirellula_baltic      ELTEMMERAVNLSPRTTCVLKGKKDDLGFVGEVTEVDRDVIESLAYTDQV
21541979_Mycobacterium_leprae       ELVNLINYAVGITGEDAQLFTAGRTDIGLVGDVDQVNIAAVLDLISAHRI
48834901_Thermobifida_fusca         EIVGLINFAVGMSGEDAHLFTAERVDIGLVGEVVDVQPGAVRSLLDDGRI
7106696_Streptomyces_coelic         ELVGLLNLAVGLTGEDAHTLTATKVDIGRVGEITEIDTGAIEALLADGRI
30409279_Cyanidioschyzon_merol      QLVGSINKAIGLCGKDANLIVAKAKELGLVGEIEQIHPQVIDMLLEKHYI
21673937_Chlorobium_tepidu          DIVQLINNAVGVSGLDADTILAKPSTLGLVGEVAEINTRYIDLLCDAGLI
11498879_Archaeoglobus_fulgid       KIVTTFIKAVGLSGKDGLLIVARKIDLGFVGETEFVNPEIIRILLDNGFI
48841339_Methanosarcina_barker      KIVSLIGKGIGLTGYDGRMILGHKVDIGWVGECEVINPDILHIVLENGYI
45655673_Leptospira_interr          QIVSLINKAVGISGKDGNLAKATKFDVGLVGKIESVNPEILHNLQKAGFI
33237476_Prochlorococcus_marin      QIVNGINSAVGLCGIDGKLIEARPGSYGLVGEVARVNADVIEPLIANGYI
6706427_Arabidopsis_thalia          NLVSLINTAVGLSGHDGRLLTARPAQLGFVGEVARVDPSVLRPLVDYGYI
50911447_Oryza_sativa               QLVSLISTAVGLCGKDARLLTARPAALGFVGEVTRVNPSVLHPIIESGHI
50927665_Oryza_sativa               HLVSLINTAVGLCGKDARLLTARPAALGFVGEVSRVDATVLHPIIASGHI
35214504_Gloeobacter_viola          SIVQMINSAVGLCGRDGSIIEARPPDIGFVGDIQSVNPKLIQSLLKEGHI
67920085_Crocosphaera_watsonii      ELVSLIN-GVGLCGKDGNLVKARREDIGFVGEVSKVDVKIVDALVNNNYI
48764058_Rhodospirillum_rubrum      AIVTEINCAVGLSGKDGRLIQARKLDLGFVGEPVVIDPHVLIQFRDSDII
28867322_Pseudomonas_syring         DIVNLINSAIGLTGKDAELIRAKKIDIGQVGEVVGVNTGLLNMLVKGDFI
23102640_Azotobacter_vinela         DIVNLINSAIGLTGKDAGLIRAKKIDIGQVGEVESVNTELLNMLVQGDFI
46311045_Burkholderia_cepaci        DIVMLINHAVGLTGKDGGLIHARKIDIGQVGEVEAINPAVVKALQDDAFI
17427041_Ralstonia_solana           DIVMLINQAVGLTGKDGGLIRAKKIDIGFVGDIETINPAVVRALQDDAFI
52007208_Thiobacillus_denitr        DIVTLINRAVGLTGKDGNFIHAKKLDIGQVGEITGIDPEIIQVLDARDFI
30249008_Nitrosomonas_europa        EIVNLINQAVGLTGKDGMFIRAKRINIGQVGEIEYIDPSLIALLDTRDFI
53759351_Methylobacillus_flage      EIVNLINKAVGLTGQDGNFIHARKIDIGQVGEISGIDPSLIQFLDSGDFI
15607035_Aquifex_aeolic             DIVALINYAVGLSGRDGRLLKAKKEDIGFVGEIVDVNEELIFTLLSHNFI
39995261_Geobacter_sulfur           EVVGYLNRAVGLSGKDGNLLLCRKVDIGFVGDVVKVNQELIQTLEHGKFI
50875328_Desulfotalea_psychr        EIVGLINKAVGLSGRDGDLVCAEQIDVGRVGQVTKINSHVLETLSQDDFI
CM204_Cyanidioschyzon_merolae       SIESALQNSVSLRVVSGNFFTAQPVDFGYTGKVRKIDVQKIMKRLDEDEI
42573209_Arabidopsis_thalia         MLEAKLSHDIGVRVDTGNFFAAKRVDFGATGEVKKIDVDRICERLDGGSV
18420097_Arabidopsis_thalia         MLEAKLSHDIGVRVDTGNFFAAKRVDFGATGEVKKIDVDRICERLDGGSV
CDO00012939_Cyanophora_paradox      --------------------------------------------------
28867555_Pseudomonas_syring         SIEARLSQGSRLRVTSGNVVTARPIDYQHTGEVRRVDRKGINRLLDERHI
23103255_Azotobacter_vinela         AIEARLSQGARLRVVGGNFVTARPIDYLHTGEVRRIDRKGIGRQLDERAI
27361298_Vibrio_vulnif              AITASLSAGTHLNVVSGNFVIAQPVDYCHSGRIRRIDTEAINRSLDQGSI
26249247_Escherichia_coli           DITARLSQGAHINVVSGNFIIAQPVDYCHSGRIRRIDEDAIHRQLDSGAI
46312172_Burkholderia_cepaci        DIEAAISAHAHISVVSGNFVTARPVDFAHTGIVRKIDAESIRHSLSSRKL
17428264_Ralstonia_solana           DIEATFSAGARISVVSGNFVTARPVDFQHTGLVRKIDAESIQHSLSNRKI
30248782_Nitrosomonas_europa        EIEALLSANAAIRVASGNFVTARPVDLQYTGEVRRINITAILDQLEQGTV
53759353_Methylobacillus_flage      EIESLLSAGADIRVASGNFVTAKPIDLQHTGEVRKIDAIGIQKRLDDGEL
52007496_Thiobacillus_denitr        EIEALLSAGADIRVASGNFVTAQPVDLLHTGEVRKIDAAAIRRRLDQHDI


32444545_Rhodopirellula_baltic      AVIPSLCTDDKGQLYNVNADTAAMAVAQSLGADKLVFLSDVNGVRRDPEI
21541979_Mycobacterium_leprae       PVVSTLAPDRDGVVHNINADTAAAALAETLGAEKLLMLTNVEGLYTRWPL
48834901_Thermobifida_fusca         PIISSIARSAEGGVYNVNADTAAAALAVALGAAKLIVLTDVEGLYADYPL
7106696_Streptomyces_coelic         PVVSSIARSQDDHVYNVNADTAAAALAAALGAETLMVLTDVEGLYEDWPV
30409279_Cyanidioschyzon_merol      PVIASVAASHDGTTYNLNADVVAGELAIKLKAKKLIFLTDTKGILADINV
21673937_Chlorobium_tepidu          PVIAPIGYDMEGNIYNINADDAAAAIAVALKAEKLIYVSDVEGVRVGNRI
11498879_Archaeoglobus_fulgid       PVVSPVATDLAGNTYNLNADVVAGDIAAALKAKKLIMLTDVPGILENPDL
48841339_Methanosarcina_barker      PVISPIAVDAKGNALNINADIVAGDIAAALHAKKLILMTDVSGLLRDMNH
45655673_Leptospira_interr          PVISPVAENSEGESLNINADTFAGEIAGALKAEKLILLTDTQGILIDNQL
33237476_Prochlorococcus_marin      PVISSVASSVEGINYNINADTVAGEIAAAIGAEKLILLTDTSGILKNKSL
6706427_Arabidopsis_thalia          PVIASVAADDSGQAYNINADTVAGELAAALGAEKLILLTDVAGILENKEL
50911447_Oryza_sativa               PVIATVAADETGQAYNINADTAAGEIAAALGAEKLLLLTDVSGILADRNL
50927665_Oryza_sativa               PVIATVAADETGQAYNINADTAAGEIAAAVGAEKLLLLTDVSGILADRNL
35214504_Gloeobacter_viola          PVLSSVASDENGQAYNINADTAAGELAAALDAEKLILLTDTPGILLDKDL
67920085_Crocosphaera_watsonii      PVISSVAADETGQAHNINADTVAGEIAAALGAEKLILLTDTAGILENYQL
48764058_Rhodospirillum_rubrum      PVIAPIGIGEAGETFNINADTAAGALAAQMKAARLLMLTDVKGVLDKDKL
28867322_Pseudomonas_syring         PVIAPIGVGPDGESYNINADLVAGKVAEALKAEKLILLTNIAGLMNKQGV
23102640_Azotobacter_vinela         PVIAPIGVGADGESYNINADLVAGKVAEALKAEKLMLLTNIAGLMDKEGV
46311045_Burkholderia_cepaci        PVISPIGFGEDGLSYNINADLVAGKLATVLNAEKLLMMTNIPGVMDKDGL
17427041_Ralstonia_solana           PVISPIGFSDDGQAYNINADVVAGKMAEILKAEKLVMMTNIPGVMDKQGL
52007208_Thiobacillus_denitr        PVVAPLGTDAEGNAYNINADVAAGKIAGVLQAEKVIFMTNTPGVLDKDKL
30249008_Nitrosomonas_europa        PVIAPIGVGEDGESYNINADLVAGRLAETLKAEKLILMTNTPGVLDKNGL
53759351_Methylobacillus_flage      PVIAPIGVGIDGQTYNINADVVAGKLAEVLGAEKLILLTNTPGVLDKTGL
15607035_Aquifex_aeolic             PVIAPVGVGEEGEAYNVNADLAASEIAGEIKAEKLIYLTDTKGVLDEKGL
39995261_Geobacter_sulfur           PVIAPVGVGEQGESYNVNADLVAGRVAGALRAEKLILLTDVAGVKDKAGL
50875328_Desulfotalea_psychr        PIIAPVGVGEDGRAFNINADLVASAIAAELSAEKLILLTDVPGVKNKAGL
CM204_Cyanidioschyzon_merolae       VILSNVGFAPSGALFNCQSEEVASSCASQLQADKLIFMTDGEMLADLRTI
42573209_Arabidopsis_thalia         VLLRNLGHSSSGEVLNCNTYEVATACALAIGADKLICIMDGP-ILDESGL
```

```
18420097_Arabidopsis_thalia         VLLRNLGHSSSGEVLNCNTYEVATACALAIGADKLICIMDGP-ILDESGL
CDO00012939_Cyanophora_paradox      ---------ARG--------------------------------------
28867555_Pseudomonas_syring         VLLSPLGYSPTGEIFNLACEDVATRAAIDLAADKLLLFGAETGLLDEQGL
23103255_Azotobacter_vinela         VLLSPLGYSPTGEIFNLACEDVATRAAIDLKADKLLLFGAEPGLLDGQGL
27361298_Vibrio_vulnif              VLLGPIASSVTGECFNLLSEEVATQVAIKLKADKLIGFCSEQGVIDEEGA
26249247_Escherichia_coli           VLMGPVAVSVTGESFNLTSEEIATQLAIKLKAEKMIGFCSSQGVTNDDGI
46312172_Burkholderia_cepaci        VLLSPLGFSPTGEAFNLSMEDVASAAAIALRADKIVFLTEGPGIVDDEGL
17428264_Ralstonia_solana           VLLSPLGFSPTGQAFNLSMEDVATNTATALKADKLIFITEVPGIMDRVGL
30248782_Nitrosomonas_europa        VLLSPLGYSPTGEIFNLTLENIAAEVAAALQADKLIFLVDTPGIRQQTEL
53759353_Methylobacillus_flage      VLLSPLGYSPTGEVFNLTLEDVATSTAIALDADKLIFLMDSSGVHNIRGL
52007496_Thiobacillus_denitr        VLLSPIGYSPTGEIFNLSLEDVATQAAVELAADKLIFLMNTEGVPGKGRI
                                               .

32444545_Rhodopirellula_baltic      IPALSAEEARQLIAGMIPKVEACLETLGRGVQKVHIIDGRLRHSLLLEIF
21541979_Mycobacterium_leprae       VNEIDSAALAQLLPGMIPKVEACLRAVTGGVPSAHVIDGRVKHCVLVELL
48834901_Thermobifida_fusca         ISHMTASELEEMLPGMVPKMEACLRAVRGGVPQAHVLDGRIPNAMLLEIF
7106696_Streptomyces_coelic         ISRLTASELEKLLPGMVPKMEGCLHAVRGGVTTARVIDGRVQHSILLEIF
30409279_Cyanidioschyzon_merol      ISTLNLKEAKNLANGMIPKVNACICAVENGVEAAHIIGGKEKHQLLLELL
21673937_Chlorobium_tepidu          LKTICKADAAELIEGMIPKVVSAYQTLDGGVGKVHLIDGQITHSLLLEVF
11498879_Archaeoglobus_fulgid       ISRIRLSELENMRSGMIPKVDAVIKALKSGVERAHIIDGSRPHSILIELF
48841339_Methanosarcina_barker      ISRVTLEDIDHLIAGMIPKLKGAAVAVKNGVEKAHIINGSVSHSMLLELF
45655673_Leptospira_interr          VTGLNRNKVKDYIRGMIPKVECCLTAIDQGVRRTHIIDGRVSHSILIEIF
33237476_Prochlorococcus_marin      IQNIRLSDMRELIDGMTPKAECCIRALAQGVNAAHIIDGRIPHALLLEVF
6706427_Arabidopsis_thalia          IKEIDIKGVKKMIEGMIPKVKCCIRSLAQGVKTASIIDGRRQHSLLHEIM
50911447_Oryza_sativa               VKEIDIAGVRQMVAGMIPKVECCVRALAQGVHTASIIDGRVPHSLLLEIL
50927665_Oryza_sativa               VKEIDIAGVRQMVAGMIPKVECCVRALAQGVHTASIIDGRVPHSLLLEIL
35214504_Gloeobacter_viola          IRKLDIYQARKLIAGMIPKVQCCVRALAQGVRAAHIVDGRQMHALLLEVL
67920085_Crocosphaera_watsonii      LTKLDIKQARDLIAGMIPKVNCCVRSLAQGVRAAHIIDGRIPHSILLEIF
48764058_Rhodospirillum_rubrum      IQELSVDRARLLKQGMIPKVETCIDAVERGVEAAVIVDGRVPHAVLLEIF
28867322_Pseudomonas_syring         LTGLTTEQVDGLIAGMLPKIRCALEAVQGGVNSSHIIDGRVPNAVLLEIF
23102640_Azotobacter_vinela         LTGLSTEQVDALIAGMLPKIRCALDAVQGGVTSAHIIDGRVPNAVLLEIF
46311045_Burkholderia_cepaci        LTDLSAREIDALFEGMLPKISSALDAAKSGVKSVHIVDGRIEHSVLLEIL
17427041_Ralstonia_solana           LTDLSAREIDELFAGMLPKISSALDAAKSGVNSVHIIDGRIEHSLLLEIL
52007208_Thiobacillus_denitr        LTGLTPREISDLIAGMIPKVGYAVDAVNSGVKTAHIIDGRVEHALLLEVL
30249008_Nitrosomonas_europa        LTGLTAGRVDELFAGMLPKIKSALDAVKNGVKSCHIIDGRVQHALLLEIL
53759351_Methylobacillus_flage      LTGLTPKQIDDLVAGMLPKISSALDAARSGVKSVHIIDGRVEHALLLEVL
15607035_Aquifex_aeolic             ISSLSKDKAEELIKGMIPKVRSALRALEKGVKKVHIIDGRVKHSILLEVF
39995261_Geobacter_sulfur           LSSIRLDTVPGLIDGMIPKVTCCVDAIEEGVRKASIIDGRVLHAVLLEIF
50875328_Desulfotalea_psychr        LTTLEWQELNGLIEGMIPKVRCCEDAVKGGVAKTYIVDGRVEHAILLEIF
CM204_Cyanidioschyzon_merolae       MHNVPLRAAMEFLQRFHVCLQESVRALRGGVTRAHLINMFVDGAILIELY
42573209_Arabidopsis_thalia         IHFLTLQEADMLVRGYLSELAAAAFVCRGGVKRVHLLDGTISGVLLLELF
18420097_Arabidopsis_thalia         IHFLTLQEADMLVRGYLSELAAAAFVCRGGVKRVHLLDGTISGVLLLELF
CDO00012939_Cyanophora_paradox      ---------------------------RDVRRLHLVGRTQDGALLVELF
28867555_Pseudomonas_syring         VRELRPQQVPAHLQ--AELLDAAAEACRGGVARSHIVSYAEDGALLTELF
23103255_Azotobacter_vinela         IRELRPQQVAAHLEEL---LDAAAQACRAGVPRSHMVSYAEDGALLTELF
27361298_Vibrio_vulnif              VAELFPSDAEKFIQGTLRFLKGAVAACRAGVPRSHLISYKIDGALIQELF
26249247_Escherichia_coli           VSELFPNEAQARVEGTVRFLRGAVKACRSGVRRCHLISYQEDGALLQELF
46312172_Burkholderia_cepaci        IREMSLDAAAELLDDDAFFLKHSIRACRGGVTRAHLIPQSLDGSMLLELF
17428264_Ralstonia_solana           QQELSMESAIERLRDTAYYLQHIVKAMRGGVRRAHLIPFALDGSILLELF
30248782_Nitrosomonas_europa        LPELTVRQGKTLLADTRLYLPWALHACEKGVKRVHLVSRHIDGALLLELF
53759353_Methylobacillus_flage      LREMTTDKARNLLRDELYYLPAAVRACGQGVARTHLISRHVDGAILQELF
52007496_Thiobacillus_denitr        HNALTVNEARQVLDDVTYYLPCAVTACTQGVKRAHLISRHRDGALLSELF
                                                                 .*    ::       :: *:

32444545_Rhodopirellula_baltic      TTDGVGTEIH
21541979_Mycobacterium_leprae       TNEGTGTKVV
48834901_Thermobifida_fusca         TDEGVGTMVL
7106696_Streptomyces_coelic         TDEGIGTMVV
30409279_Cyanidioschyzon_merol      TEKGRGSMIV
21673937_Chlorobium_tepidu          TNEGVGTQFV
11498879_Archaeoglobus_fulgid       TKEGIGTMVE
48841339_Methanosarcina_barker      TDRGIGTMVY
45655673_Leptospira_interr          TDQGIGSLIE
33237476_Prochlorococcus_marin      TDKGIGTMIV
6706427_Arabidopsis_thalia          SDEGAGTMIT
50911447_Oryza_sativa               TDEGTGTMIT
50927665_Oryza_sativa               TDEGTGTMIT
35214504_Gloeobacter_viola          TDQGIGSMLV
67920085_Crocosphaera_watsonii      TDRGIGSMIV
48764058_Rhodospirillum_rubrum      TPRGAGTLIR
28867322_Pseudomonas_syring         TDSGVGTQIT
23102640_Azotobacter_vinela         TDTGVGTLIT
46311045_Burkholderia_cepaci        TEQPFGTMIR
```

```
17427041_Ralstonia_solana          TEQAFGTMIR
52007208_Thiobacillus_denitr       TPEGVGTLIK
30249008_Nitrosomonas_europa       TDEGVGTLIK
53759351_Methylobacillus_flage     TDAGVGTLIT
15607035_Aquifex_aeolic            TKEGVGTEIT
39995261_Geobacter_sulfur          TDVGVGTEIH
50875328_Desulfotalea_psychr       TRDGVGTEIY
CM204_Cyanidioschyzon_merolae      TRDGVGLMIS
42573209_Arabidopsis_thalia        KRDGMGTMVA
18420097_Arabidopsis_thalia        KRDGMGTMVA
CDO00012939_Cyanophora_paradox     TRNGSGLMVV
28867555_Pseudomonas_syring        TRDGGGTLVA
23103255_Azotobacter_vinela        TRDGGGTLVT
27361298_Vibrio_vulnif             SFDGIGTQVV
26249247_Escherichia_coli          SRDGIGTQIV
46312172_Burkholderia_cepaci       LHDGVGTMIS
17428264_Ralstonia_solana          LHDGVGTMVS
30248782_Nitrosomonas_europa       THSGIGSMIT
53759353_Methylobacillus_flage     THDGIGTMVT
52007496_Thiobacillus_denitr       TRDGVGTLIT
                                        •   .
```

Note: All top hits of GenBank Searches (using *Cyanidioschyzon* sequence and *Arabidopsis* accession number NP_974701 as queries) are from gamma and beta-proteobacteria. Multiple copies of this gene exist in plants and in bacteria. One of these copies in red algae and green plants forms a group with homologs from gamma and beta-proteobacteria with strong support, whereas the other copy groups with cyanobacterial sequences with modest support (the *Cyanidioschyzon* sequence in that group is encoded in the plastidic genome). In *Arabidopsis*, the protein product of the gamma and beta-proteobacteria-related gene copy is annotated as located in cytoplasm (GenBank accession number NP_974701 and TAIR locus AT4G37670). The EST sequence of glaucophyte *Cyanophora* was obtained from tbESTdb; this sequences groups with homologs of red algae and green plants in preliminary analyses, but was removed from the detailed phylogenetic analyses because of its very short length (only 32 aa)

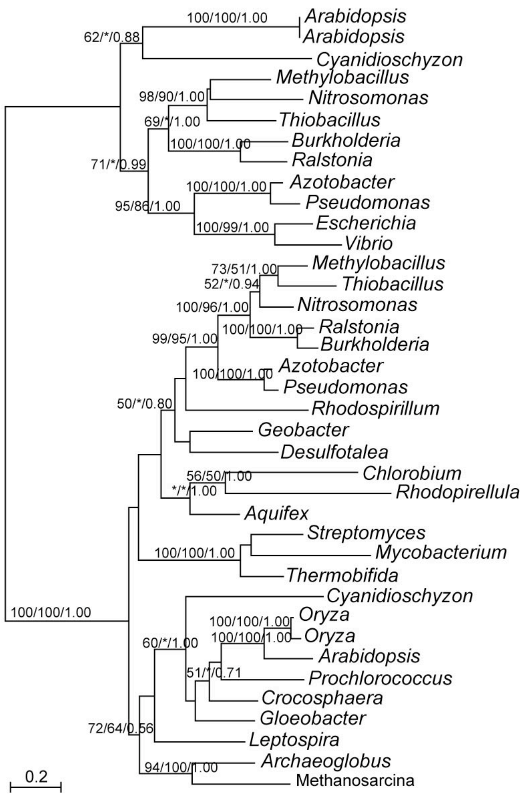

Figure 1. Molecular phylogeny of GCN5-related N-acetyltransferase. P-value = 0.235 from AU test on the presented tree. AU tests were also performed on alternative topologies including (**A**) monophyly of all red algal and green plant sequences, and (**B**) monophyly of cyanobacterial and all red algal, green plant sequences. These alternative topologies investigate if both copies of this gene in red algae and green plants have the same plastidic origin. P-values < 0.001 from AU tests on both alternative topologies.

---

2. Glycyl-tRNA synthetase (D)

Note: GlyRS in living organisms exists in forms of homodimer (α_2) and heterotetramer ($\alpha_2\beta_2$); the former is distributed in eukaryotes, archaea, and many bacteria whereas the latter is only found in bacteria, red algae and green plants. Few bacteria contain both glyRS types. The α- and β-subunits of tetrameric glyRS are usually encoded in separate genes. **In a few groups, the two subunits are encoded in a single fused gene; these include actinomycetes, chlamydiae, red algae, and green plants.** Not only are sequences from actinomycetes, red algae and green plants similar in gene structure, they also have the highest percent identity and share several conserved amino acid residues. Phylogenetic analyses of each of the subunits strongly suggest a common origin of actinomycete, red algal and plant sequences (**Figures 2A-2B**). Based on

the gene structure and molecular phylogeny, it is likely that primary photosynthetic eukaryotes acquired this gene from either actinomycetes or chlamydiae (Chlamydiae are the only bacterial group aside from actinomycetes and plants that possess a fused gene in our database searches). The second scenario requires an independent HGT event from photosynthetic eukaryotes or chlamydiae to actinomycetes. The *Arabidopsis* sequence (GenBank accession number NP_190394, TAIR locus AT3G48110) is experimentally determined to be targeted to both chloroplasts and mitochondria.

2A. Alpha subunit

```
CLUSTAL X (1.83.1) multiple sequence alignment


67931183_Solibacter_usitat         FQEIILTLKRYWADRGCIIQEPYDTEVGAGTMCPETFLRVLGPKPYRTAY
4980714_Thermotoga_mariti          LQDVIMKLNDFWASKGCLLEQPYDMEVGAGTFHPATFFGSLRKGPWKVAY
34484120_Wolinella_succin          FSDLLLKLQTFWKEQGCLIVQPYDIPAGAGTFHPATLLRSLDSKPWSVAY
15611961_Helicobacter_pylori       FSSLLLKLQEYWKDQGCLVIQPYDIPAGAGTFHPATLLRSLDKKPWNVAY
50914779_Streptococcus_pyogen      FQEIILTLQQYWNDQGCMLMQAYDNEKGAGTMSPYTFLRAIGPEPWNAAY
23099404_Oceanobacillus_iheyen     IQEMILTLQNHWSEHNCILMQAYDTEKGAGTMSPMTLLRSLGPEPWNVAY
46907687_Listeria_monocy           LQTMIRTLQDYWSEQGCIMLQSYDVEKGAGTMSPYTFLKAIGPEPWKAGY
51891712_Symbiobacterium_therm     FQDLVLTLDKFWAEQGCIIRHPYDVEKGAGTFNPETFFRALGPEPWNVAY
15606265_Aquifex_aeolic            FQDIIMTLHKFWAEKGCLIWQPYDVEVGAGTMNPATFLKVLGKKPWNVAY
34763703_Fusobacterium_nuclea      FQEIIFSLQQYWSSKGCIIGNPYDIEKGAGTFNPNTFLMSLGPEPWNVAY
66855635_Anaeromyxobacter_deha     FQDLILKLQSYWASRNCILAQGYDQEVGAGTMNPSTFLRVLGPEPWNVAY
39934120_Rhodopseudomonas_palu     FQGLILTLQRFWADYGCVILQPYDMEVGAGTFHPATTLRALGPKRWNAAY
13476181_Mesorhizobium_loti        FQGLILTLHNYWAAYGCVILQPYDMEVGAGTFHPATTLRALGPKRWNAAY
23013834_Magnetospirillum_magn     FQSLILTLQAFWAEQGCVILQPYDMEVGAGTFHPATTLRALGPDHWKCAY
48766000_Rhodospirillum_rubrum     FQSLILRLQQFWAAQGCVLLQPYDMEVGAGTFHPATTLRALGPEPWKAAY
68213209_Methylobacillus_flage     FQQIILTLQQYWDKQGCALLQPYDMEVGAGTSHTATFLRALGPEPWKAAY
56476158_Azoarcus_sp.              FQQVILTLQQYWGERGCVLLQPYDLEVGAGTSHTATFLRAIGPEPWNAAY
26986806_Pseudomonas_putida        FQDLILALQNYWAAQGCVVLQPYDMEVGAGTFHTATFLRAVGPETWNAAY
1573948_Haemophilus_influe         FQGMILALQEYWANQGCTIVQPFDMEVGAGTSHPMTALRALGPEPMAFAY
46580307_Desulfovibrio_vulgar      FQNVILTLQNYWASRGCVITQPIDVECGAGTFNPSTFLRVIGPEPWNVAY
68179079_Desulfuromonas_acetox     FQDLILSLQNYWAQQGCVIQQPYDIEKGAGTFNPATFLRSLGPEPWNVAY
39982448_Geobacter_sulfur          FQDLILALQGYWAQQGCVIQQPYDTEKGAGTFNPATFLRVLGPEPWNVAY
68207211_Desulfitobacterium_ha     FQDMILSLNQFWGEQGCIIAQPYDMEKGAGTFNPNTFLRALGPEPWKVAY
72382617_Prochlorococcus_marin     FQDIISSLNNFWSEKGCLLLQPYDLEKGAGTMSPHSFLRAIGPEPWAVAY
17131075_Nostoc_sp.                FQSVIATLHQFWAERGCLIAQPYDIEKGAGTKNPHTFLRALGPEPWAVAY
46135082_Anabaena_variab           FQSVIATLHQFWAERGCLIAQPYDIEKGAGTKNPHTFLRALGPEPWAVAY
68230324_Frankia_alpha             MQDALLTLTRYWTERGCMIVQPFNTEVGAGTHNPATILRVLGGEPWRVAY
68172611_Frankia_sp.               MQDALLALTRYWTDRGCMIVQPFNTEVGAGTLNPATVLRVLGPEPWRVAY
28572421_Tropheryma_whippl         MQDTILILQTYWAHRGCLVFQPMNTEVGAGTANPATLLGVLGDKTWGVAY
46399968_Parachlamydia_sp.         FQEFLRTLSTFWEKQGCIIHQGYDLEVGAGTFNPATFLRSLGPEPYRAAY
C_130039_Chlamdydomonas_reinh      FQEAITKLQEYWASVGCAIWLPHNTEVGAGTMNPATFLRVLGPEPWNVAY
15836479_Chlamydophila_pneumo      LQSMIATILRFWSEQGCVIHQGYDLEVGAGTFNPATFLRALGPEPYKAAY
15605530_Chlamydia_tracho          LQTMMAAILNFWSEQGCIIHQGYDLEVGAGTFNPATFLQSLGPEPFRTAY
55296761_Oryza_sativa              FQQAIQRLQDYWASVGCAVMQCSNTEVGAGTMNPLTFLRVLGPEPWNVAY
30692978_Arabidopsis_thalia        FQQAIQRLQEYWASVGCAVMQPSNTEVGAGTMNPCTFLRVLGPEPWNVAY
CM269_Cyanidioschyzon_merolae      FQEAILRLQIYWAERGCLLWRPYNAEVGAGTMNPATFLRALGPEPWNVAY
                                   :.  :  :  .*   .* :    :   ****  . : :  :       .*

67931183_Solibacter_usitat         VQPSRRPADGRYGDNPNRLYKHSQLQVILKPAPEDVIELYLGSLEAIGID
4980714_Thermotoga_mariti          VQPSRRPTDGRYGENPNRLQRYFQYQVIIKPSPENSQELYLESLEYLGIN
34484120_Wolinella_succin          VAPSRRPTDGRYGENPNRLGSYYQFQVIIKPSPDNIQELYLKSLEYLGLN
15611961_Helicobacter_pylori       VAPSRRPTDGRYGENPNRLGSYYQFQVVIKPSPSNIQELYLKSLEVLGIN
50914779_Streptococcus_pyogen      VEPSRRPADGRYGENPNRLYQHHQFQVVMKPSPSNIQELYLASLEKLGIN
23099404_Oceanobacillus_iheyen     VEPSRRPDDGRYGENPNRLYQHHQFQVIMKPSPDNIQELYLESLKKLGIN
46907687_Listeria_monocy           VEPSRRPADGRYGENPNRLFQHHQFQVVMKPSPDNIQELYLGSLEKLGIN
51891712_Symbiobacterium_therm     IEPCRRPADGRYAQNPNRMQHYFQYQVIMKPSPENIQEIYLEMLERIGID
15606265_Aquifex_aeolic            VEPSRRPQDGRYGENPNRLQHYYQFQVILKPAPRNPQEIYLESLERLGIN
34763703_Fusobacterium_nuclea      VEPSRRPKDGRYGDNPNRVYQHHQFQVIMKPSPINIQELYLESLRVLGID
66855635_Anaeromyxobacter_deha     VEPSRRPADGRYGENPNRLYQHHQFQVILKPNPPDVQELYLGSLRAIGID
39934120_Rhodopseudomonas_palu     VQPSRRPKDGRYGENPNRLQHYYQFQVILKPSPPDIQDLYLKSLAAIGVD
13476181_Mesorhizobium_loti        VQPSRRPKDGRYGENPNRLQHYYQYQVILKPNPPNLQELYLGSLAAIGVD
23013834_Magnetospirillum_magn     VQPSRRPKDGRYGENPNRLQHYYQYQVLLKPSPPNAQELYLESLKRLGID
48766000_Rhodospirillum_rubrum     VQPSRRPTDGRYGENPNRLQHYYQFQVILKPSPADPQALYLDSLRALGID
68213209_Methylobacillus_flage     VQPSRRPKDGRYGDNPNRLQHYYQFQVVLKPAPGNILELYLGSLEALGFD
```

```
56476158_Azoarcus_sp.             VQPSRRPKDGRYGENPNRLQHYYQFQVVLKPSPLNIQELYLDSLRALGID
26986806_Pseudomonas_putida       VQPSRRPADGRYGENPNRLQHYYQFQVVLKPNPANFQELYLGSLKAIGLD
1573948_Haemophilus_influe        VQPSRRPTDGRYGENPNRLQHYYQFQVVIKPSPDNIQELYLGSLEMLGFD
46580307_Desulfovibrio_vulgar     VEPSRRPTDGRYGENPNRLQHYFQFQVILKPSPDNVQELYLGSLRALGID
68179079_Desulfuromonas_acetox    VEPSRRPTDGRYGENPNRLQHYYQFQVILKPAPLNIQELYLDSLKSFGID
39982448_Geobacter_sulfur         VEPSRRPTDGRYGENPNRLQHYYQFQVIMKPSPMNILDLYLDSLRAFGID
68207211_Desulfitobacterium_ha    IEPSRRPTDGRYGENPNRLQHYFQYQVIIKPSPDNIQELYLQSLERLGVN
72382617_Prochlorococcus_marin    PEPCRRPTDGRYGDNPNRAQHYFQYQVLIKPSLDGIQEIYLSSLEALGIS
17131075_Nostoc_sp.               IEPCRRPTDGRYGENPNRFQHYYQYQVLIKPSPDNIQDIYLDSLRALGIR
46135082_Anabaena_variab          VEPCRRPTDGRYGENPNRVQHYYQYQVLIKPSPDNIQDIYLDSLRALGIR
68230324_Frankia_alpha            VEPSVRPDDSRYGFNPNRLQTHTQFQVVLKPDPGNPQELYLGSLQALGID
68172611_Frankia_sp.              VEPSVRPDDSRYGHNPNRLQTHTQFQVILKPDPGNPQELYLGSLAALGID
28572421_Tropheryma_whippl        VEPCVRPDDSRYGINPSRLQCHTQFQVLLKPEPGNPQEMYLESLEAIGID
46399968_Parachlamydia_sp.        IEPCRRPADGRYGTNPNRLQHYFQYQVILKPSPLNMQELYLQSLEALGFN
C_130039_Chlamdydomonas_reinh     PEPSIRPDDSRYGDNPNRLQRHTQFQVILKPDPGNAQELYLGSLEALGID
15836479_Chlamydophila_pneumo     VEPSRRPQDGRYGVHPNRLQNYHQLQVILKPVPENFLSLYTESLRAIGLD
15605530_Chlamydia_tracho         IEPSRRPQDGRYGQHPNRLQKYHQLQVILKPVPENFLSLYLESLKVIGLN
55296761_Oryza_sativa             VEPSIRPDDSRYGDNPNRLQRHTQFQVILKPDPGNSQDLFLHSLSALGIN
30692978_Arabidopsis_thalia       VEPSIRPDDSRYGENPNRLQRHTQFQVILKPDPGNSQQLFINSLSALGID
CM269_Cyanidioschyzon_merolae     EEPSVRPDDSRYGENPNRIQQHTQFQVILKPAPPNSQELLLGSYAALGID
                                    *. ** *.**. :*.*    : * **::**    .   :       :*.

67931183_Solibacter_usitat        LRKHDLKLEEDNWEAPTLGAWGIGWQVMLDGLEISQFTYFQQCGGIDLDL
4980714_Thermotoga_mariti         LKEHDIRFVEDNWESPTLGAWGVGWEVWLDGMEITQFTYFQQIGGISLKD
34484120_Wolinella_succin         LKEHDIRFVEDNWESPTLGAWGLGWEVWLDGMEVTQFTYFQQVGGIACDP
15611961_Helicobacter_pylori      LNEHDIRFVEDNWESPTLGAWGLGWEVWLDGMEVTQFTYFQQVGGIPCSP
50914779_Streptococcus_pyogen     PLEHDIRFVEDNWENPSTGSAGLGWEVWLDGMEITQFTYFQQVGGLATSP
23099404_Oceanobacillus_iheyen    PLEHDIRFVEDNWENPTLGAAGLGWEVWLDGMEITQFTYFQQIGGLEANP
46907687_Listeria_monocy          PLEHDIRFVEDNWENPSLGCAGLGWEVWLDGMEITQFTYFQQVGGLECFP
51891712_Symbiobacterium_therm    PKKHDIRFVEDNWEAPSQGAWGLGWEVWCDGMEITQFTYFQQVGGFECKP
15606265_Aquifex_aeolic           PLEHDIRFVEDDWESPTLGAWGLGWEVWLDGMEITQFTYFQQAGGLDLDE
34763703_Fusobacterium_nuclea     PEKHDIRFVEDDWESPTLGAWGLGWEVWLDGMEITQFTYFQQVGGLELDV
66855635_Anaeromyxobacter_deha    PREHDIRFVEDDWESPTLGAWGLGWEVWCDGMEITQYTYFQQAGGFEVKP
39934120_Rhodopseudomonas_palu    TALHDVRFVEDDWESPTLGAWGLGWECWCDGMEVSQFTYFQQVAGVECAP
13476181_Mesorhizobium_loti       PLLHDIRFVEDDWESPTLGAWGLGWECWCDGMEVSQFTYFQQVCGIECAP
23013834_Magnetospirillum_magn    PLAHDIRFVEDDWESPTLGAWGLGWEVWCDGMEVTQFTYFQQVGGIECDP
48766000_Rhodospirillum_rubrum    PLKHDIRFVEDDWESPTLGAWGLGWEVWCDGMEVTQFTYFQQVGGFDCDP
68213209_Methylobacillus_flage    LQQNDIRFVEDDWENPTLGAWGLGWEVWLNGMEVTQFTYFQQVGGINCRP
56476158_Azoarcus_sp.             TNAHDIRFVEDDWENPTLGAWGLGWEVWLDGMEVTQFTYFQQVGGLDCKP
26986806_Pseudomonas_putida       PLVHDIRFVEDNWESPTLGAWGLGWEIWLNGMEVTQFTYFQQVGGIECYP
1573948_Haemophilus_influe        PTKNDIRFVEDNWENPTLGAWGLGWEVWLNGMEVTQFTYFQQVGGLECKP
46580307_Desulfovibrio_vulgar     PAAHDIRFVEDDWESPTLGAWGLGWEVWLNGMEVTQFTYFQQVGGIDLAP
68179079_Desulfuromonas_acetox    SASHDIRFVEDDWESPTLGAWGLGWEVWLDGMEITQFTYFQQVGGIDLKP
39982448_Geobacter_sulfur         PAKHDIRFVEDDWESPTLGAWGLGWEVWLDGMEITQFTYFQQAGGIDLKP
68207211_Desulfitobacterium_ha    PKEHDIRFVEDNWESPTLGAWGLGWEVWLDGMEVTQFTYFQQCGGIDCKP
72382617_Prochlorococcus_marin    AKDHDIRFVEDNWESPTLGAWGVGWEVWLDGMEVTQFTYFQQCGGLDCKP
17131075_Nostoc_sp.               PEDHDIRFVEDNWEDATVGAWGTGWEVWLDGMEITQFTYFQQCGGIDCRP
46135082_Anabaena_variab          PEDHDIRFVEDNWEDATVGAWGTGWEVWLDGMEITQFTYFQQCGGIDCRP
68230324_Frankia_alpha            TAAHDVRFVEDNWASPALGAWGLGWEVWLDGQEITQFTYFQQAGGQSLDP
68172611_Frankia_sp.              TAAHDVRFVEDNWASPAFGAWGLGWEVWLDGLEITQFTYFQQAGGQTLDT
28572421_Tropheryma_whippl        LKKHDIRFVEDNWESPALGAWGLGWEIWLDGLEITQFTYFQQVGGVPLKT
46399968_Parachlamydia_sp.        LQEHDIRFVHDDWEAPTQGAWGLGWEVWMDGMEITQFTYFQAIGGIELRP
C_130039_Chlamdydomonas_reinh     TKA---------------------------------------GGRVLDA
15836479_Chlamydophila_pneumo     LRDHDIRFIHDDWENPTIGAWGLGWEVWLNGMEITQLTYFQAIGSKPLDT
15605530_Chlamydia_tracho         LVDHDIRFVHDDWENPTIGAWGLGWEVWLNGMEITQLTYFQAVGSKPLDA
55296761_Oryza_sativa             VREHDIRFVEDNWESPVLGAWGLGWEVWMDGMEITQFTYFQQSGSLPLLP
30692978_Arabidopsis_thalia       VTAHDIRFVEDNWESPVLGAWGLGWEIWMDGMEITQFTYFQQAGSLPLSP
CM269_Cyanidioschyzon_merolae     IDAHDIRFVEDNWESPALGAWGLGYEVWMDGCEITQYTYFQQVGGFSLDP
                                                                              .

67931183_Solibacter_usitat        VPAELTYGLERLTAFLQVKDSVYDIDWTYGDVRYKDELQYSVYNFEMADI
4980714_Thermotoga_mariti         IPLEITYGLERIAMYLQGVDNVYEVQWKYGDVFLENEREFSVFNFEEANV
34484120_Wolinella_succin         VAVEITYGTERLAMYLQEVENVFDIEWSYADVHLEGEYEFSKYHFEVADT
15611961_Helicobacter_pylori      IPVEITYGLERLTMYVQKVENILEIEWRYAQVHLESEYEFSKYHFEVASV
50914779_Streptococcus_pyogen     VTAEVTYGLERLASYIQEVDSVYDIEWKYGEIFLQPEYEHSKYSFEMSDQ
23099404_Oceanobacillus_iheyen    VTVELTYGIERLASYIQDKENVFDLEWTIRDIFYQPEYEHSTYTFKESNT
46907687_Listeria_monocy          VTSEITYGVERLASYIQDKENVFDLEWSYRDIFFQAEFENSTYAFETSNT
51891712_Symbiobacterium_therm    VSVEITLGLERLASYIQGVDNVWDIMYRYGDVFERFEWEHSVYNFEQSDT
15606265_Aquifex_aeolic           ISVEITYGLERIAMYIQDKDSVFDIEWTYGEIFKRSEWEWSKYNFELADT
34763703_Fusobacterium_nuclea     IPVEITYGLERLALYIQNKENVYDLEWKYGDMRYQFEFENSKYSFELASL
66855635_Anaeromyxobacter_deha    VAAELTYGLERIAMYLQDVENVFDVEWKYREVFHRNEVEMSTYSFQASDP
39934120_Rhodopseudomonas_palu    VAGELTYGLERLAMYVQGIDRVYDLNFTYGDVFLQAEKEYSKHNFEVADT
13476181_Mesorhizobium_loti       VAGELTYGLERLAMYVQGVDNVYDLNFTYGDVFLQAEQEYSRHNFEYADT
```

```
23013834_Magnetospirillum_magn      VAVELTYGLERLAMYIQGVENVYDLDFTYGDVFLQAEREYSAHNFEHADT
48766000_Rhodospirillum_rubrum      VPVEMTYGLERLAMYIQGVENVYDLDYRYGDIFLQGEKEFSAHNFEYANT
68213209_Methylobacillus_flage      ITGEITYGLERLAMYLQGVENVYDLVYKYGDVFHQNEVEQSAYNFEHSDA
56476158_Azoarcus_sp.               VLGEITYGLERLAMYLQGVENVYDLVWTYGDVFHQNEVEQSTYNFEHSNV
26986806_Pseudomonas_putida         VTGEITYGLERLAMYIQGVDSVYDLVWTYGDVFHQNEVEQSTYNFEHANV
1573948_Haemophilus_influe          VTGEVTYGLERLAMYIQGVDSVYDLVWTYGDVFHQNEVEQSTYNFEHANT
46580307_Desulfovibrio_vulgar       TSVEITYGLERLCMYLQGKESVYDLDWTYGDIYHQNEVEQSKYNFERSDA
68179079_Desulfuromonas_acetox      IPGEITYGCERIAMYLQGVDNVYDLEWKYGDIHHQTEVEFSAYNFEEADT
39982448_Geobacter_sulfur           VSSEITYGCERIAMYLQGVDNVYDLEWSYGDIHHRTEVEFSTYNFEEADV
68207211_Desulfitobacterium_ha      VCAEITYGLERLAMYIQNKESVYDIEYTYGDIYLQNEIDYSHYNFEAADV
72382617_Prochlorococcus_marin      VSIEITYGLERLAMYLQNVESIWDLSWKYGDIWLPFETGQCKYNFEESNS
17131075_Nostoc_sp.                 VSIEITYGLERLAMYLQEVEAITKIHWTYGDVFLQNEIEQSTYNFEASNP
46135082_Anabaena_variab            VSIEITYGLERLAMYLQEVEAITKIHWTYGDVFLQNEIEQSTYNFEASNP
68230324_Frankia_alpha              VSVEITYGIERILMVLQKVRHFTEIAYSYGEVFGQAEYEMSRYYLDEADI
68172611_Frankia_sp.                VSVEITYGVERILMALQGVRHFSEIAYSYGEVFGQAEYEMSRYYLDDADI
28572421_Tropheryma_whippl          SAVEITYGIERIMMALQKVSHFKDIEYSYGELFAQNEREMSAYYLEKANI
46399968_Parachlamydia_sp.          ITGEITYGIERLAMYLQKVDSIFDLKWTYGDVYQRNEVEWSHYNFEKAST
C_130039_Chlamdydomonas_reinh       PAVEITYGLERILMALQGVTNFKDIRYTYGEMFYQNEYEMSCYNLDEADV
15836479_Chlamydophila_pneumo       ISGEITYGIERIAMYLQKKTSIYDVLWTYGQITQASEKAWSEYNFDYANT
15605530_Chlamydia_tracho           ISGEITYGVERIAMYLQKKNSVYDVMWTYGDITQYAEQAWSQYNFETANT
55296761_Oryza_sativa               VSVEITYGLERILMSLQGVDHFKNIQYTYGELFLENEKEMSAYYLEHANV
30692978_Arabidopsis_thalia         VSVEITYGLERIIMLLQEVDHFKKILYTYGELFLENEKEMSSYYLEHASV
CM269_Cyanidioschyzon_merolae       IALEITYGMERILMTVQGKSHFKDILYTYGEVFLQNEIEMSRYNLEEADV
                                       *:* * **:   :*    . .: :   ::    *   . . :. :.

67931183_Solibacter_usitat          PTLWKLFDLYEGEAIRLLGLYDPLPAYDQVLKCSNLFNTLDARGAISVTE
4980714_Thermotoga_mariti           GLLFRHFDEYEKEFYRLVEKNLYLPAYDYILKCSHTFNLLDARGAISVSQ
34484120_Wolinella_succin           QRLLTLFEEAQGESKRCLEALLPLPAYDWTMLSSHMFNVLDARKAISATE
15611961_Helicobacter_pylori        ERLLEMFKNAQAEALHCLENKLPLPAYDLVMLCSHFFNILDARKAISVAE
50914779_Streptococcus_pyogen       DMLLENFEKFEKEASRALEEGLVHPAYDYVLKCSHTFNLLDARGAVSVTE
23099404_Oceanobacillus_iheyen      DMLFDLFSMYEQEAKQTMEKGLVFPAYDYVLKCSHTFNLLDAKGVISVTE
46907687_Listeria_monocy            DMLLTLFDTYEREATRQMQDGLVFPAYDYVLKCSHTFNLLDARGVVSVTE
51891712_Symbiobacterium_therm      QMLFQLYNMYEAEAKRLLSLDLVYPAYDYILKCSHTFNLLDARGAIAVSE
15606265_Aquifex_aeolic             DMLFQVYEMFEKESKRMVEEGLIFPAYDYLLKCSHVFNILDARGAISVQE
34763703_Fusobacterium_nuclea       DKHFKWFDDYEEEAKKILDQGLVLPAYDYVLKCSHVFNVLDSRGAISTTE
66855635_Anaeromyxobacter_deha      KMLFGLFDTYEAECKRLIGLKLPLPAYDYCLKCSHAFNNLDARGAISVTE
39934120_Rhodopseudomonas_palu      DMLFEQFKMAEAACKKYLDAGWSLPAYDQCIKASHVFNLLDARGVISVTE
13476181_Mesorhizobium_loti         AMLLRHFEDAEAECKALLDAGAPFPAYDQCIKASHVFNLLDARGVISVTE
23013834_Magnetospirillum_magn      DMLLQHFLDAETECKNLLDKGVALPAYDQCIKASHRFNLLDARGVISVTE
48766000_Rhodospirillum_rubrum      EALFRHFKDAEEECMALLAKGVPLPAYDQCIKASHRFNLLDARGVISVTE
68213209_Methylobacillus_flage      DFLFTAFNAHEKKAQELMEHKLALPAYEQVLKAAHTFNLLDARGAISVTE
56476158_Azoarcus_sp.               DFFFSLFGNYEAEAKRLMEAGLALPAYEMVLKAAHSFNMLDARGAISVTE
26986806_Pseudomonas_putida         DKLFELFDFYESEANRLIKLELPLPTYEMVLKASHTFNLLDARRAISVTE
1573948_Haemophilus_influe          DFLFYCFDQYEKEAQELLALELPLPAYERILKAAHSFNLLDARKAISVTE
46580307_Desulfovibrio_vulgar       AMLLHAFNAYETECRRLTEEGLLWPAYDYCLKCSHTFNLLDARGAISITE
68179079_Desulfuromonas_acetox      GMLFQLFDMYEKECTKLAQKDLVFPAYDFVMKASHAFNLLDARGAISVTE
39982448_Geobacter_sulfur           DMLLTLFTMYEKECVRLVERGLVLPAYDFVMKCSHTFNLLDARGAISVTE
68207211_Desulfitobacterium_ha      EALQTWFEMYEKEAIRIVEKGLVLPAYDYVLKCSHTFNLLDARGAISVTE
72382617_Prochlorococcus_marin      ENLRKLFEIYEEEAHQLIAKKLPAPCLDYVLKCSHTFNLLEARGVISVTE
17131075_Nostoc_sp.                 ELLLTLFNLYEQEASQLTERGLVLPSLDYVMKCSHTFNLLDARGVISVTE
46135082_Anabaena_variab            ELLLTLFSLYEQEATQLTEKGLVLPSLDYVMKCSHTFNLLDARGVISVTE
68230324_Frankia_alpha              DTVRRMYADCAAEARRLIDARLPVPAHIYVLKCSHAFNILDSRGAVSTTE
68172611_Frankia_sp.                DTVRRLYADYAAEARRLIDARLPVPAHSYVLKCSHAFNILDSRGAVSTTE
28572421_Tropheryma_whippl          ERNKQLFEIYSDEAKMLLRAGLPLPAYTFVLKCSHVFNILDARGAISTAE
46399968_Parachlamydia_sp.          SMWFRHFDDYENEAKQLIANNLPIPAYDFVMKASHAFNLLDARGVISVTE
C_130039_Chlamdydomonas_reinh       AGQRARFDLYDKEARRLLAKRLPIPAYDHLLKLSHTFNLMDARGAVGVTE
15836479_Chlamydophila_pneumo       EMWFKHFEDFAEEALRTLKNGLSVPAYDFVIKASHAFNILDARGTISVTE
15605530_Chlamydia_tracho           TMWLKHFDDFSAEALATLDQGLPLPAYDFVIKASHAFNMLDSRGVISVTE
55296761_Oryza_sativa               DNIQKHFDDFEEEARSLLSLWLPIPAYDHVLKASHAFNILDSRGFVGVTE
30692978_Arabidopsis_thalia         DRLQKHFDYFDEEARSLLALGLPIPAYDQLLKTSHAFNILDARGFIGVTE
CM269_Cyanidioschyzon_merolae       ERQRHLFDLSEAEARELLQKRLPIPAYHYVLRTSHTFNILDARGAVGVAE
                                          :                 *     :  :: ** ::::  :.  :

67931183_Solibacter_usitat          RVGVIQRVRKMAVAVAHAWVDQG
4980714_Thermotoga_mariti           RQTYVKRIQAMARKAARVFLEVQ
34484120_Wolinella_succin           RQNYILKIRELAKGCAELYKGQE
15611961_Helicobacter_pylori        RQNYILQIRDLAKGCAVLYKEQE
50914779_Streptococcus_pyogen       RAGYIARIRNLARVVAKTFVAER
23099404_Oceanobacillus_iheyen      RTGYIARIRNLARSISKIYVSER
46907687_Listeria_monocy            RAQYIGRIRNLARRIAKTFYESR
51891712_Symbiobacterium_therm      RQGYILRVRNLARAAAKAYIEMR
15606265_Aquifex_aeolic             RARYIRRMNNLAREIAKLYLQVF
34763703_Fusobacterium_nuclea       RMGYILRVRNLARRCAEVFVENR
```

```
66855635_Anaeromyxobacter_deha      RAAYIGRVRALAHDCAKGYLDSR
39934120_Rhodopseudomonas_palu      RQSYILRVRELAKACGEAWVHTE
13476181_Mesorhizobium_loti         RQSYILRVRNLAKACGEAFLKTQ
23013834_Magnetospirillum_magn      RQAYIGRVRALAKACCEGWLASR
48766000_Rhodospirillum_rubrum      RAAYIGRVRALAKGCCEGWLRAR
68213209_Methylobacillus_flage      RATYIGRIRNLARAVAASYLDSR
56476158_Azoarcus_sp.               RAAYIGRIRNLSRAVAQAYSASR
26986806_Pseudomonas_putida         RQRYILRVRTLARDVAQSYLQAR
1573948_Haemophilus_influe          RQRYILRIRALTKGVAEAYYASR
46580307_Desulfovibrio_vulgar       RTGYIGRVRYLASGVARLYAAQR
68179079_Desulfuromonas_acetox      RAHYIGRVRNLSRLCAEGYVAQR
39982448_Geobacter_sulfur           RASYIGRVRNVARLCAEGYLKLR
68207211_Desulfitobacterium_ha      RTGYIARVRNLARLCAQAYVEQR
72382617_Prochlorococcus_marin      RTKIIARIRSLARKVAEAWLEER
17131075_Nostoc_sp.                 RTRYIARIRHLARKVANLYVEQR
46135082_Anabaena_variab            RTRYIARIRHLARKVANLYVEQR
68230324_Frankia_alpha              RATSFAQMRGMSREVAQLWRDRR
68172611_Frankia_sp.                RATSFAQMRGLSREVATLWRDRR
28572421_Tropheryma_whippl          RAKAFSLMRTLTKEISATWVESR
46399968_Parachlamydia_sp.          RTGYIGRIRELAKQVAESYIKSR
C_130039_Chlamdydomonas_reinh       RAECFAVLRGLAREITGLWVSRR
15836479_Chlamydophila_pneumo       RTRYIARIRQLTRLVADSYVEWR
15605530_Chlamydia_tracho           RTRYIAKIRQLARAAADKYVAWR
55296761_Oryza_sativa               RARYFGRMRSLARQCAQLWVKTR
30692978_Arabidopsis_thalia         RARYFGRMRSLARQCAQLWLATR
CM269_Cyanidioschyzon_merolae       RAAYFRRMRSLAREVAALWLERR
                                    *   .  :. ::      :
```

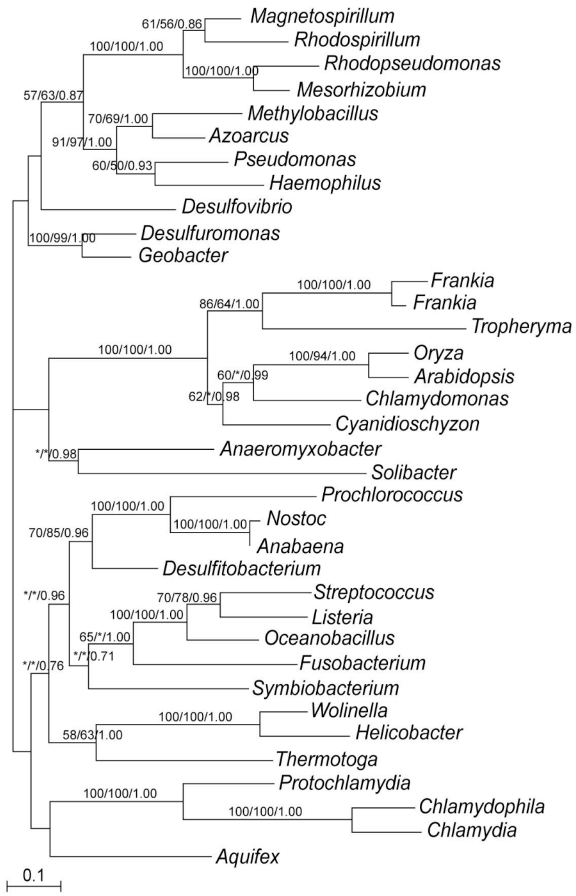

Figure 2A. Molecular phylogeny of alpha subunit of glycyl-tRNA synthetase. P-value = 0.838 from AU test on the presented tree. AU tests were also performed for alternative topologies, including (**A**) monophyly of cyanobacterial and photosynthetic eukaryotic sequences, (**B**) monophyly of cyanobacterial, *Frankia*, *Tropheryma*, and photosynthetic eukaryotic sequences, and (**C**) monophyly of chlamydial, *Frankia*, *Tropheryma*, and photosynthetic eukaryotic sequences. These tests investigate different evolutionary scenarios, including a) plastdic origin of photosynthetic eukaryotic sequences, b) photosynthetic eukaryotes acquired this gene from plastids and subsequently spread to *Frankia* and *Tropheryma* by a secondary HGT event, c) photosynthetic eukaryotes acquired this gene from chlamydiae and subsequently spread to *Frankia* and *Tropheryma*. P-value < 0.001 from AU test for topology **A** whereas P-values = 0.247 and 0.287 for topologies **B** and **C**, respectively. Therefore, the scenario of a plastidic or chlamydial origin of this gene in red algae and green plants, though less parsimonious, cannot be confidently rejected.

2B. Beta subunit

```
CLUSTAL X (1.83.1) multiple sequence alignment


68172611_Frankia_sp.                LFEIGTEELPAAEVTRTVEAVRAGLVERLAATRLTLGTPRRIVAIVDEVA
68230323_Frankia                    -------------MTRQTAAVRAALVERLAATRLTQGTPRRIVAIVDDVA
28572421_Tropheryma_whippl          LFEIGTEEMPS--TQEIITTVENTLREKLGRSQLKDATPRRIVIRIYKMH
46399968_Parachlamydia_sp.          VIEIGSQELPASFVSIGGQNLERAIRALLEKEGISYGTPRRIAVYIYQLS
C_130039_Chlamdydomonas_reinh       VLEVGGEELPPDDVVSAVHQLRERVPALLAKLRLSEGTPRRLAVVVEALA
15836479_Chlamydophila_pneumo       LLEIGSEELPATFVPIGIQQLESLARQVLTDHNIVLGSPRRLALLVKNVA
15605530_Chlamydia_tracho           LLEIGSEELPATFVPTGIQQLESLAKKLLADHGIALGTPRRLALCIEGLS
55296761_Oryza_sativa               VLEIGTEELPPHDVIEATKQLEKSLIQILEKRRLSYGTPRRLAVVVENLN
30692978_Arabidopsis_thalia         IIEIGTEEMPPQDVINASEQLRVLVLELLENQRLRFGTPRRLVVLVDAMS
CM269_Cyanidioschyzon_merolae       LIEIGTEELPPADLDAAVEQLREHLLQLLHSSRLLRATPRRHAILVEGVA
34482366_Wolinella_succin           LIEIGIEELPAIPFLRELPHLLSKWNTLLQKYRMEFYTPRRLVLLSQAFP
15611973_Helicobacter_pylori        LVEVLVEELPAQALLNEYKEMPKKLHALFQKRALEFYTPRRLCLFVKDFP
15607085_Aquifex_aeolic             LIEIGTEELPAGVINPALDYLKDKINSLLNARQVKYGTPRRLTLYFKDFE
66855634_Anaeromyxobacter_deha      LFEIGAEEIPAGFVPGALRQLEDDLAKALADARLAVGTPRRLAVWARDVA
46580306_Desulfovibrio_vulgar       VLEIGTEELPARFLPALERELAERFTRALADAGIEMSTPRRAVVRMDAVN
4980715_Thermotoga_mariti           LLEVGLEELPASEFHSILKQLEEKSAELLKAYRVSFVGSRRFGVILKNLP
68207212_Desulfitobacterium_ha      LLEIGTEEIPAKFAPGVLNQLREQAQKYCQELRLDYTTPRRFAVLIQGLA
50914778_Streptococcus_pyogen       LIELGLEELPAYVVTPSEKQLGERLATFLTENRLSFSTPRRLAVRVSGLA
17133246_Nostoc_sp.                 LLEVGTEELPASFLGGAIAQWRSRIPQSLAANSLTYGTPRRLAVLITGLP
45508785_Anabaena_variab            LLEVGTEELPASFLSGAIAQWRSRIPQSLAANSLTYGTPRRLAVLITGLP
72382003_Prochlorococcus_marin      LLEIGTEELPADLAESVISQLELSVNNDLNSAQIKTTTPRRIALTIEGIA
46907686_Listeria_monocy            LLEIGLEEMPAKYVTSSVLQLEKRVTDWLKDNQIEYSTPRRLTVLVEEMA
23099403_Oceanobacillus_iheyen      LIEIGLEELPARFIDDAELQLYTKTKQWLEENRISYSTPRRLAVFVKNMA
68179078_Desulfuromonas_acetox      FLEIGTEEIPAGMLPVAMRDLERMMRKELTNARVAYATPRRLVLSVADVA
39982449_Geobacter_sulfur           FLEIGTEEIPAGFLPKAMADMEAIVTKELENARLAFATPRRLALVVKGLP
51891713_Symbiobacterium_therm      ILEIGTEEIPARFCAPALEQLKENAAKALAEARLDFGTPRRLVLYVRNLA
67931182_Solibacter_usitat          LLEIGTEEIPDWMITTALENLRMS----FEKLEITDATPRRLVLRVEGVP
1573946_Haemophilus_influe          LVEIGTEELPPKALKTLATSFADNVEAELNQAGLSFAAPRRLAVKVLNLA
26986805_Pseudomonas_putida         LVELGTEELPPKALASLGDAFLAGIEKGLQAAGLNYAAPRRLAVLIRQLD
13476182_Mesorhizobium_loti         LLELRSEEIPARMQRKAAGDLKKMLTDGLVEAGLTYWTPRRLTLDIRGLT
39934122_Rhodopseudomonas_palu      LLELFSEEIPARMQGKAADDLKRLVTDKLVAEGLVFATPRRLTLTVHGIP
23013833_Magnetospirillum_magn      LLEIFSEEIPARMQARAADDLRGMVTDGLAKNGITYVTPRRLVLVIEGLP
68213210_Methylobacillus_flage      LVELFVEELPPKALQNIGISFADQIYHSLVTQDLIFASPRRIAVWIRNVT
                                                                     :    .**       .

68172611_Frankia_sp.                PREPDVERVVRGPPTKAAVGFARGQGHVALVRTEAGRDAARVLAGVGELV
68230323_Frankia                    PREPDVERVVRGPPTKAATGFVRGQGHVALVRTDTGRPATEVLAEVGAVL
28572421_Tropheryma_whippl          PRQSDSSVRIRGPPTDALTGFLKANGVFITK-NIPGQSAAKLLEQIAEIV
46399968_Parachlamydia_sp.          KGKPSQTIEKKGPIKVAGEGFFRSLGYLFGSVKQEGRATTSILAEKPLLI
C_130039_Chlamdydomonas_reinh       AAQTSEESKVRGPPTKALEGFCRKNGYVWAVVKDAGRPAVEVLTDEPALV
15836479_Chlamydophila_pneumo       PEVVQKAFEKKGPVSPQGQQFFASQGYLFLLHPEIRLRTADILMQEPLLI
15605530_Chlamydia_tracho           HVTIRPESEKKGPVSPQGEQFFSSHGYLFLVIPEERKETAAILVNEPQLI
```

```
55296761_Oryza_sativa               MKQMEEEIELRGPPTKAAEGFCRKNNYIYARVKESARFADEVLTEDPTII
30692978_Arabidopsis_thalia         SKQLEEEVEVRGPPTKAAEGFSRRYGYVHARVTEPARLALEVLSEDPGIL
CM269_Cyanidioschyzon_merolae       HKQPDQWTQIRGPLTAVGQAFAQRHQYIFLNRTIQGQRALPLLHERDSVL
34482366_Wolinella_succin           LEQPELKEALFGPPTKAAESFAAKCGVLFYSKTSAGESAKRLLPLMEEWI
15611973_Helicobacter_pylori        LLTQETKEEFFGPLNALGLGFYQKLGVLYHAKIHAKQPTKDLIMPILEFL
15607085_Aquifex_aeolic             NERKEKKEVIWGPPTKALEGFLKKNNVAIVRKVIEKSPIEK-LQEEEEIL
66855634_Anaeromyxobacter_deha      PKQTDARTEAFGPPTPAATGFARSQGRVAVTKVEKGRKAEQVLPALERLV
46580306_Desulfovibrio_vulgar       PVQSESEEVVTGPLTKAAEGFARTQGYIAVRKHMGGARSIDLLRDIPAII
4980715_Thermotoga_mariti           ERQEDFTEEKKGPPTRALEGFLRNNNVYLSR-VVEGKPVEEVLPDLRDLV
68207212_Desulfitobacterium_ha      EKQTDFTAEVKGPPTKAAQGFARGQGYVYARKFELGQPTLQLLPKLTDLI
50914778_Streptococcus_pyogen       AQQTDLTEDFKGPFSKAAQGFVRGKGYVYVTKHEAGKPAKEVLLGVTEVL
17133246_Nostoc_sp.                 SQQPDREEEIKGPPTPAAVGFAKKQGFVFIQKKTPGRPVADILTELPQWI
45508785_Anabaena_variab            SQQPDREEEIKGPPTPAAVGFAKKQGFVFVQKKTPGRPVAEILTELPQWI
72382003_Prochlorococcus_marin      PFSEDNIAERKGPPTKAAIGFAKRYEFVFAKSIEKGKPVTTVLSDYPRWI
46907686_Listeria_monocy            EEQANRVEEAKGPWSKAALGFAKSQKYIYIKKEVIGEKTTALLPSLEKVV
23099403_Oceanobacillus_iheyen      EKQSSIEEDVKGPWTKAAQGFTKGQGYIYVTKHIEGRPIQELLPEFKSII
68179078_Desulfuromonas_acetox      EEQERQELNLSGPPTKAALGFARSNGYLFLSKVIEGQPIKEQLPEIERIV
39982449_Geobacter_sulfur           TVQPDAEITALGPPSKAAEGFARGQGYVAAVRKESGRPVPDLLAEIPRLV
51891713_Symbiobacterium_therm      LRQRDVEVEVKGPFTVPARKFAEGQGYLWARKRIQGESVETVLPPLAGLV
67931182_Solibacter_usitat          AKQQDSEERILGPPPKAVEGFARKQGYYVYQKKVPGRFIKDILAEAPGII
1573946_Haemophilus_influe          TQQPSKEIEKRGPPTKAAEGWARGCGWLIHRAKIEGQPTKNLLNDIANAL
26986805_Pseudomonas_putida         VQQPDRSINIDGPPTQAALGFAKKCG-LRFSQHIPGKATVGLLPTIEDSL
13476182_Mesorhizobium_loti         ARSKDIREEIKGPPEQAVQGFLRKAGFYVAHISKPGRAAEEIIAQLPGII
39934122_Rhodopseudomonas_palu      ARQPDLKDERKGPPDAAVQGFLKATGFYVALIEKPGKPAIDVIAQFPVIV
23013833_Magnetospirillum_magn      LAGPDVSEEKRGPPAQAMEGFLKSTGFYFAVINRKGRPTAEVLSEVEATM
68213210_Methylobacillus_flage      QKAPDKQLAQKLMATPALLKKLAALGVLLLDRTVRGATLAEGLQKADEAI
                                                                              :      :

68172611_Frankia_sp.                VGLRAERNMRWNPTLSFSRPVRWLLALLGETVVPVTVSTLAAGRTTRGHR
68230323_Frankia                    GGLRAERNMRWNPELSFSRPVRWVVALLGGAVVPVAASTLAAGSTTRGHR
28572421_Tropheryma_whippl          LEIRSSRNMRWNPKLVFSRPIRWLVALYGQRQLNVRVSLLESGMHTRVLR
46399968_Parachlamydia_sp.          GSLDFPKKMRWALEITYARPLEWVLALFGKDIIPFAIGNLFSDRITYGHR
C_130039_Chlamdydomonas_reinh       GGLSFKKSMRWSASPAFSRPLRWLLALHGDTVLPFVYGTLQAGATTRVLR
15836479_Chlamydophila_pneumo       QRMKFPKKMVWDSGVEYARPIRWLVALYGEHILPITLGTIIASRNSFGHR
15605530_Chlamydia_tracho           RSIRFPQKMTWDGGVEYARPIRWLVALYGDQILPISLGFVSSGNTSWGHR
55296761_Oryza_sativa               SGISFPKSMRWNSNIVFSRPIRWIFALHGDLIVPFCFAGISSGNQSCGLR
30692978_Arabidopsis_thalia         AKISFPKSMRWNSSVMFSRPIRWVMALHGDLVVPFSFAGISSGNVSCGLR
CM269_Cyanidioschyzon_merolae       AKIQFRRSMRWNSGVLYSRPVRWLLAMLDDQVIPVAFGGVVSAPVTWGLR
34482366_Wolinella_succin           ASLNFGKSMRWGLHENFIRPVRWLGIMHGENLIPATLFGISSQKLTYVHR
15611973_Helicobacter_pylori        EGLNFGKSMRWGVEKSFIRPIHNICVLFNFNDIEIKEYGFKTKQATKAHR
15607085_Aquifex_aeolic             LSVPFPKRMRWTKRITFSRPVRWILALFNGQVLKLRFGELESSNKTYGHR
66855634_Anaeromyxobacter_deha      AGLRFRKAMRSRDEVTFARPVRWMVALLGGRPLKVRHGEVASGKVTYGHR
46580306_Desulfovibrio_vulgar       GALPFPKRMRWGGDFTYARPMRWLLALFDESVVDFEVGGVRSGNITYGHR
4980715_Thermotoga_mariti           LGLNFRKPMRWGE-HEYIRPVHWIVAMVDGRVLDLEIFGLRSSRISYGKR
68207212_Desulfitobacterium_ha      TGLHFPKPMRWALEFRFARPIRWIVALFGSEVIPFEFVGLASGKASRGHR
50914778_Streptococcus_pyogen       SAMTFPVSMHWANSFEYIRPVHTLTVLLDDEALDLDFLDIHSGRVSRGHR
17133246_Nostoc_sp.                 YSLEGKRLMRWGGDVRFSRPIRWLVALLDEAILPLELVNVKSDRISYGHR
45508785_Anabaena_variab            YHLEGKRLMRWGGDVRFSRPIRWLVTLLDEAILPLELVNVKSDRISYGHR
72382003_Prochlorococcus_marin      SKIQGRRFMRWGGDFRFSRPIRWIVSLLDSEVLPFKISGIQIGNISRPHR
46907686_Listeria_monocy            TSMTFPVSMHWGNDLRYIRPIKWLIAMFGEEIIPFEITGVSTSNTSRGHR
23099403_Oceanobacillus_iheyen      ESITFGKNMRWGETIRYARPIRWLVAMMGNEVIPFEIAHVATNNVTYGHR
68179078_Desulfuromonas_acetox      SSLSFKKSMRWKLDVRFARPVHWLIALFGGEVIPFSWGNLTSGNTSYGHR
39982449_Geobacter_sulfur           GGIPFRKSMRWALDVRFARPIHWIVALFDGVVVPFAFGNVQSGNVSRGHR
51891713_Symbiobacterium_therm      TSIHWPKAMRWARELRYARPIKWILALLGDWRVPFDVDGIETVSTTRGHR
67931182_Solibacter_usitat          LGLYFPKTMYWTGGPRFIRPIRWIVALLGEEIIPFELAGVRSGALTSGHR
1573946_Haemophilus_influe          AKLPIPKPMRWAKTVQFIRPVHTVTMLLGDELIEGEILGVASARTIRGHR
26986805_Pseudomonas_putida         NDLPIPKRMRWA-REEFVRPTQWLVMLLGDQVVDCTILSQKAGRESRGHR
13476182_Mesorhizobium_loti         RNFPWPKSMRWGGSLRWVRPLQSVLCTFGPVVVDFEIDGIRSGNITYGHR
39934122_Rhodopseudomonas_palu      RTFPWPKSMRWGGALQWVRPLHSIIATFGPDVVHFNVDGIEAGQTTRGHR
23013833_Magnetospirillum_magn      AAFPWPKSQRWGNSIRWVRPIQSILALFEGHVVPVEFGPAKSGDMTCGHR
68213210_Methylobacillus_flage      AKLPIPKVMTYQSNVNFVRPAHGLVALHGNEVVPIRALGLTAGNRTQGHR
                                      .             : ** . :        :                *

68172611_Frankia_sp.                RAGPLDVPSASGYPELLAARILLDPVVRRELVVVLTEVANLVEFPCPILG
68230323_Frankia                    RAGPIEVTGAVGYPELLAENVLLDPAARRELIVVLDEVTNLVEFPRPVLG
28572421_Tropheryma_whippl          NSKIVPVKAADNYEEFLTRNVILSRSKRREIIFPIEEITDLVEAPSLVIC
46399968_Parachlamydia_sp.          QLSAIHLDHAKNYLKSLHQHVMADPNEREESILVIPQVLNLVEWPYLTIS
C_130039_Chlamdydomonas_reinh       NAATLPVPSAEAYLPALAAAISLPGAGRAEAIWLLEEVTNLVEAPSVVMG
15836479_Chlamydophila_pneumo       QLDKISISSPQDYVETLRQAVVVSQKERRMIIELIEEATFLSEHPFVSCG
15605530_Chlamydia_tracho           QLDQLTIPSSNMYVDTLRSAVIVSQKERRAIIKLIDETVFLTEHPFVISA
55296761_Oryza_sativa               NSSNFKVEAAELYLHTLEKAILIDMQERKQRILLVQEVINLVEAPMPIIG
30692978_Arabidopsis_thalia         NTASLLVQNAESYEDTMRNSINIEIEERKKIILLLNEVANLVEAPVPLIG
CM269_Cyanidioschyzon_merolae       ASGPQLPVSHAQLYDRVLERNIVAEVSAREQSIMLHEVARLIESPQVLLG
```

```
34482366_Wolinella_succin           IDSPQPYEDIASFLALLAQKVILDPKVRREKILLLEEVVAITEYPTALLG
15611973_Helicobacter_pylori        QEGFIEVDSPKAYFEVLEKNVILDPKKREAKILLLDEVVAITEYPTALLG
15607085_Aquifex_aeolic             FLSEVTINNPADYEKTLKEHVIPDFNERKEIILLVEEVTNLVEYPFPVLG
66855634_Anaeromyxobacter_deha      FLAAIALKGTPDDYLAKLRRVLADPEERRAALLLVEQVLYLVEEPSAVVG
46580306_Desulfovibrio_vulgar       IHGPLTVARAGDYERVIREQVTPVGEERRNAVVLLDEVQGLVEHPVPCLG
4980715_Thermotoga_mariti           YHASIEIPDSERYYESLKKGVISSHLERKKFVLLIEEIVAITEYPRIVVG
68207212_Desulfitobacterium_ha      TLGPVTLDSPADYEKQMLQAVMVDPEQRRQSVWLLDEVTHIIEYPTALLG
50914778_Streptococcus_pyogen       FLGETTITSADSYEDDLRSQVIADAKERQEMIVLLNEVLNLVEFPTAFMG
17133246_Nostoc_sp.                 VLHPITIQKAGDYVNTLRDGVTVNAKERTTTIKLLEEVTNLVEWPSPVVG
45508785_Anabaena_variab            VLHPITIQQASDYVNTLRSGVTVDAKERAAIIKLLEEVTNLVEWPSPVVG
72382003_Prochlorococcus_marin      LYGKLEINDAKTYFDQLQDVVTVDRDRRLSCINLLNELTDLIESPLLVTG
46907686_Listeria_monocy            FLGTATINQPSDYPNALLEQVVVNASERKQAIVLLEEVTNLVEYPTVLAG
23099403_Oceanobacillus_iheyen      FLGEVTIQEPAEYEKTLKDNVIVKAKDRETLIVLLEEVRNLVEFPTAFVG
68179078_Desulfuromonas_acetox      FMAGFDVTG-LDDFVAKAREVIVDPEERKQIIALLEEVAYLVEDPTPLCG
39982449_Geobacter_sulfur           FMAPFPVRD-FAHYLDECERVIPDPERRKETIRLLDEVAYLVEYPSVVHG
51891713_Symbiobacterium_therm      VLGPIAVSDADDYFAKVAGGVMVDQAVRKQVIWLLEELTWLVEQPTAFAG
67931182_Solibacter_usitat          RLGEIAITT-SDYEQRLRDHVILSAQERRNQIRLLETLVYLTEFPRPIRG
1573946_Haemophilus_influe          FLGEFYIQHADQYPQLLREKVVADFNERKAEILLLEEVTSLVEYPNVLAA
26986805_Pseudomonas_putida         FHHNVLITTPANYVEDLR-KVLADFAERRELISLLDEVTALVEWPVPLVC
13476182_Mesorhizobium_loti         FLAEITVRRFDDYVSKLEAAVVLDADRRKEIILLLEEVSGLVEWPVVLMG
39934122_Rhodopseudomonas_palu      FMAEFSVRRFEDYEAKLFAAVVLDSARRKDIILLLDEVSGLVEWPVALMG
23013833_Magnetospirillum_magn      FLASFRVKDFADYLARLRHAVMLDPVERRHAILLLAEVTGLVEWPVPLVG
68213210_Methylobacillus_flage      FEAPIVLRNADSYAEQLQQDVIPGFKERRDEIALLEEVTALVERPNVLVG
                                                                      :     : * *

68172611_Frankia_sp.                SFEERFLDLPAEILTTVMRKHQRYLPVLLPSFVAVADGKVVRAGNEDVIR
68230323_Frankia                    SFEPRYLDLPAEILTTVMHKHQRYLAVLLPCFVTVADGAVVRAGNEAVVR
28572421_Tropheryma_whippl          RFEKKYLELPEVVLNAIMKKQKYITLSISQHFITFANGLCVRSGNEAVIR
46399968_Parachlamydia_sp.          TFREEFLKVPKEVLISEMVEHQKYFPVLKNLFVITANIIPIKQGNQKVLS
C_130039_Chlamdydomonas_reinh       SFSPDFLALPSEVLVMVMRKHQRYFPVLLPHFITVANG-PVAAGNEAVLR
15836479_Chlamydophila_pneumo       QFSEQFCALPKELLIAEMVNHQKYFPTISNFFIVVCDNSPIIEGNEKALT
15605530_Chlamydia_tracho           QFDPAFCSLPKELLIAEMIQHQRYFPTITNRFLIVCDNSPIVEGNEKALA
55296761_Oryza_sativa               RYDVSFLALPKDVLITVMQKHQKYFPVLLPCFITVANGAIVRKGNEAVLR
30692978_Arabidopsis_thalia         KFKESFLELPEELLTIVMQKHQKYFSILLPYFIAVANGAIVKKGNEAVLR
CM269_Cyanidioschyzon_merolae       SFDEAYLALPSPVLITVMQKHQRYFPILRNAFVIVANGRLVRRGNEAVIQ
34482366_Wolinella_succin           HFEARFLELPPEVIITSMKENQRYFALLYNGFIVVSNARSIVQGNEKVLR
15611973_Helicobacter_pylori        EFDKAFLKLPSEIITTSMKENQRYFAILHNGFIVVSNAISIILGNQKVLK
15607085_Aquifex_aeolic             KFDEKYLELPPLVITTVAAHHQRFFCFLLNYFLGISNNKPIKEGYEKVLR
66855634_Anaeromyxobacter_deha      EFERSNLELPPEVVISEMRNHQRYFAVLKNRFVAVSATRVARHGYERVLR
46580306_Desulfovibrio_vulgar       NIDPSFLELPREVLLTSMESHQKSFGVLMPHFLTVLNLTPVRKGWERVLR
4980715_Thermotoga_mariti           QFDRKYLELPEEIIVTAVKHHQRSFIALTNTFVAFQDGPQVVKGYERVIN
68207212_Desulfitobacterium_ha      EVAPNYMHLPEPVITTPMKEHQRYFPVLLPYFITVRNGDDVKAGNEKVLK
50914778_Streptococcus_pyogen       SFEAKYLDVPEEVLVTSMKNHQRYFVVLMPNFVSVRNGNDVIKGNEKVLV
17133246_Nostoc_sp.                 KFEEEFLNLPTEVTTTVMVSHQRYFPVLLPYFITVSNGDPIAVGNERVIR
45508785_Anabaena_variab            KFEEEFLNLPTEVTTTVMVSHQRYFPVLLPYFITVSNGDPIAVGNERVIR
72382003_Prochlorococcus_marin      CFDESFLELPPEVLSTVMKVHQRYIPLLLPTFLCISNGLPIIKGNEKVLK
46907686_Listeria_monocy            NFEKEYLELPEEVLITTMKEHQRYFPVLLPHFVTVRNGNHVARGNEKVLR
23099403_Oceanobacillus_iheyen      KFEEAYLEIPPEVLITSMKEHQRYFPVLQPYFVGVRNGDNVAKGNEKVLR
68179078_Desulfuromonas_acetox      SFEEEFLQLPDELLITSMKEHQRYFTLLLPKFITISNTRPVVKGNERVLR
39982449_Geobacter_sulfur           TFSPDFLKVPREVLITSMRSHQRYFSILMPGFITINNTLTVVKGNERVLR
51891713_Symbiobacterium_therm      SFDPAFLEVPAEVLVTTMKDNQRYFPVLLPYFIGVRNGGHVRAGNEKVLA
67931182_Solibacter_usitat          SFDPQFLELPEEVLITVMRHHQKYFSVLAPEFVAVMNIDSVRRGNERVLR
1573946_Haemophilus_influe          KFEEHFLAVPAEALVYTMKGDQKYFPILLPHFIFVSNINPIIEGNEKVVR
26986805_Pseudomonas_putida         SFEERFLEVPQEALITTMQDNQKYFCLLLPRFITVANVESIVQGNEKVVR
13476182_Mesorhizobium_loti         EFEEAFLAIPAEVIRLTIRANQKCFVTLSNRFILTSNIEAIAHGNGKVVR
39934122_Rhodopseudomonas_palu      SFDKDFLSIPDEVIRATIRANQKCFVVLANKFILTANIEAITAGNERVIR
23013833_Magnetospirillum_magn      SIDDKFMDVPAEVLITSMRAHQKYFSLLASRFVVISNMETVVAGNQRVLR
68213210_Methylobacillus_flage      QFEQAFLAVPQECLILTMKANQKYFPLLTNKFLIVSNISPVVGGNERVVR
                                            :*          .:     :   *:          *   .:

68172611_Frankia_sp.                ARFTDAAFFYDADITVPLETFRAELAKLTFEQRLGSVADRADRIAALLDR
68230323_Frankia                    ARFEDAAFFFDADLKVPLETFRAGLAKLTFEQRLGSVADRAERIARLLDR
28572421_Tropheryma_whippl          TRFEDALFFQEADSKLSPENLYKMLDGLVYEEKLGSYLQKSQRIQRIARQ
46399968_Parachlamydia_sp.          ARLSDGVFLYEEDLKVPLASFNEKLKKVTFQKELGTVYQKVERIIQHVER
C_130039_Chlamdydomonas_reinh       ARFEDAAFFYREDLKQPLQDFRPKLSGTTFQKELGSLLAKSDRVEQLAVQ
15836479_Chlamydophila_pneumo       PRLTDGEFLFKQDLQTPLTTFIEKLKSVTYFEALGSLYDKVERLKAHLDI
15605530_Chlamydia_tracho           PRLTDGNFLFKQDLLTPLSSFVEKLKSVTYFESLGSLADKTSRLKLHIDT
55296761_Oryza_sativa               ARYEDAKFFYKMDTQKKLSEFRDQLSSILFHERLGTMLDKMKRVENTIKD
30692978_Arabidopsis_thalia         ARYEDAKFFYEVDTRKRFSEFRDQLQGILFHEKLGTMLDKMNRLKKMVED
CM269_Cyanidioschyzon_merolae       ARFADAKFFYEADLKRPLASFVPKLEGLIFEERLGSMLDKTRRIEALPAP
34482366_Wolinella_succin           ARLSDALFFYQNDLKNGLLT--ERLQEVTFVEGLGSMAQKSERETKIMQE
15611973_Helicobacter_pylori        ARLSDAVFFYENDLKKPLDN--APLESVVFVQGLGTLKDKMERESIIISR
15607085_Aquifex_aeolic             ARLEDALFFYREDLKKDLKSLIPELKKVLFHPKVGSMYEKEERMEKIVKE
```

```
66855634_Anaeromyxobacter_deha             ARLADARFFFEEDRKRKLHERIEDLGRRTFQAKLGSELDRAQRIGAVLLE
46580306_Desulfovibrio_vulgar              ARLEDARFFWKTDLASSFDAWLASLDNVIFLGPLGSMGDKTRRLEQLAAR
4980715_Thermotoga_mariti                  ARLEDARYYFQKDLETPLEKMNEKLKEIVFQEKLGTLYDKVERIKKIVLE
68207212_Desulfitobacterium_ha             ARLEDAAFYYREDQKTPLAELVEKLDKVTYHEKLGSVRQRVERIRTLVER
50914778_Streptococcus_pyogen              ARLEDGEFFWREDQKLQIADLVAKLTNVTFHEKIGSLAEHMDRTRVIVDR
17133246_Nostoc_sp.                        ARLADGQFFYKTDLSKSLESYLPQLETVTFQEDLGSLRGKVERIVKVNQR
45508785_Anabaena_variab                   ARLADGQFFYKTDLSKSLESYLPQLETVTFQEDLGSLRGKVERIVKVIQR
72382003_Prochlorococcus_marin             ARFADASFFIKSDLLISSSSRIDKLKDVTFAEGLGSLYERVNRIEWLSVK
46907686_Listeria_monocy                   ARLSDADFFYQEDLKITIDEAVAKLQNIVFHEKLGTLTEKMKRVQKVIIR
23099403_Oceanobacillus_iheyen             ARLADAEFFYNEDLHQSIDFFQEKLTKVVFQEKLGTYSDKVERMKQIIER
68179078_Desulfuromonas_acetox             ARLSDAMFFWKEDQKTPLENRLEALKNVVYQAQLGTSYAKVQRFTALTRR
39982449_Geobacter_sulfur                  ARLSDARFFFEEDQKVKLETRVESLKNVVYQQKLGTSYEKMERFRALTSR
51891713_Symbiobacterium_therm             ARLSDARFFWDEDRRQPLESFNARLKEAVFQEKLGTQFERVERLVSLAAR
67931182_Solibacter_usitat                 ARFNDARFFWDADQKKPLAARKQDLANVTFQAKLGSYLVKTERMMALAVR
1573946_Haemophilus_influe                 PRLTDAEFFFKTDLKQKLVDRLPRLETVLFQQQLGTLKDKTDRIEQLAKR
26986805_Pseudomonas_putida                PRLTDAEFFFKQDKKQPLETFNERLKNVVFQAQLGTVYDKAERVSRLAGR
13476182_Mesorhizobium_loti                ARLSDALYFWTTDQGDLPDLGQLEASAETFHAKLGTQGERVERIRRLAAR
39934122_Rhodopseudomonas_palu             ARLSDAKFFYDTDLKTKLEDRLPKFDQIVFHEKLGTQAERIARIERLTKR
23013833_Magnetospirillum_magn             ARLSDAKFFWDTDRKQRLESRLPKLAERTFYASLGTVADKVERIAGLAER
68213210_Methylobacillus_flage             PRLADAKFFFEQDRKKTLESRIPGLSKVVYHNKLGSQGERIARVSEIASQ
                                           .*   *.  :     *                   :    :*:    :   *

68172611_Frankia_sp.                       AAALAKFDLATQMVIELSSLAGTMAREYARRAGEPEAVAVALFEMDQLPD
68230323_Frankia                           AGELAKFDLATQMVIELSSLAGTMAREYARRAGEPEAVAVALHEMGTLPA
28572421_Tropheryma_whippl                 SAAIAKFDLASQMVTEMPELSGFMAKIYAQRAGKPPEVAQALEDMKSLPV
46399968_Parachlamydia_sp.                 AALLSKADLASNMVYEFPELQGTIGRYYALANREEAEIAQAIDEHAPLPE
C_130039_Chlamdydomonas_reinh              AAHLCKADLATSTVTEMTALAGTMGRHYAAKQGLPAAVSEAVFEADLLPS
15836479_Chlamydophila_pneumo              AIQYCKADLVSAVVNEFPELQGIMGEYYLKHANLPTASAVAVGEHQKL--
15605530_Chlamydia_tracho                  AIHYCKADLVSSVVNEFPELQGIMGRYYLQNASLSRAAALAIGEHSSI--
55296761_Oryza_sativa                      AAALAMSDLATNIVTEFTSLAGIMARHYALRDGLSEQIAEALFEIDVFPK
30692978_Arabidopsis_thalia                AASLAMSDLATAVVTEFTALSGIMARHYALRDGYSEQIAEALLEIDVIPK
CM269_Cyanidioschyzon_merolae              VPSPCDSPVDTASPRMRAQSPTPDWEQYARKQGYRDDVARALFEIDQVPR
34482366_Wolinella_succin                  AATLAKADLMSETVYEFTELQGTIGYYLGKALGKDPLVYEALKEQSELPS
15611973_Helicobacter_pylori               AIQIAKADLLSEVVYEFSELQGIMGYYYALKQNENELVALSVKEQAPLPS
15607085_Aquifex_aeolic                    AVWLSKVDLLTEMVKELDELQGYMGYVYAKAQGYDEEVAKALWEQDKVPE
66855634_Anaeromyxobacter_deha             ASRLAKVDLNTGMVGEFPELQGTMGAHYARLEGLKPEIADAIEDHEEMPR
46580306_Desulfovibrio_vulgar              AGRLSKGDLVSGMVGEFDTLQGIMGGIYARRMGEAEAVAAAIAEQSPVPS
4980715_Thermotoga_mariti                  AASICKADIASKVVYEFPELQGVMGRIYALREGINEEIATAIEDHSEEPQ
68207212_Desulfitobacterium_ha             TALLAKADLVTLMVYDFPELQGIMGADYARMVGEKPEVCTGILEHDELPQ
50914778_Streptococcus_pyogen              AAQIYKFDLLTGMVGEFDELQGIMGEKYALLSGEDAAVATAIREHGALPE
17133246_Nostoc_sp.                        AALLCKADLVTQMVFEFPELQGIMGQKYAVASGESPEVSTAIVEHDILPQ
45508785_Anabaena_variab                   ATLLCKADLVTQMVFEFPELQGIMGQKYAVASGESPEVATAIFEHDILPQ
72382003_Prochlorococcus_marin             VAHFSKHDLVSNMVDEFPELQGIIGSKYLLHEGESRDVCLGVLEHDSLPS
46907686_Listeria_monocy                   LTNIYKFDLVTNIVGEFPELQGLMGEKYALLQGEKPAIATAIREHGDLPQ
23099403_Oceanobacillus_iheyen             AAEISKFDLMTSMVNEFTELQGIIGEKYANHFGENSATSQAIKEHDDLPQ
68179078_Desulfuromonas_acetox             AAQLCKCDLETGMVYEFPELQGVMGREYAAIEGEDPRVAKAIFEHGDLPS
39982449_Geobacter_sulfur                  AAFLCKADLVSGMVGEFPEVQGIMGREYALLQGEDAEVAAAIAEHGDLPA
51891713_Symbiobacterium_therm             AAWLCKADLMTRMVFEFPEVQGYMGKQYLLHQGGDPAVAEAIYEHDDLPR
67931182_Solibacter_usitat                 AAELCKCDLTTELVKEFTELQGVVGGLYARVQGEPEAVWQAIYDHDALPR
1573946_Haemophilus_influe                 AGLLSKCDLMTNMVFEFTDTQGVMGMHYARHDGEDEEVAVALNEQDELPK
26986805_Pseudomonas_putida                AGLLSKCDLATEMVGEFPEMQGVAGYYYALNDGEPEDVALALNEQAELPQ
13476182_Mesorhizobium_loti                AAVLAKADLQTEVVGEFPELQGAMGRKYALLQGEHPSVAAAIEEHDYVPS
39934122_Rhodopseudomonas_palu             AAKLCKADLLTEVVGEFPELQGLMGKYYALAQGEDASVAAACEEHDRVPT
23013833_Magnetospirillum_magn             AARLAKADLSSELVGEFPELQGLMGRYYALNDGENPLVADAIAAHDSCPT
68213210_Methylobacillus_flage             AAQLAKADLVTDMVGEFPELQGIMGRYYAQHEGLSDDIAYAIEDHDALPR
                                                     : :                                   .

68172611_Frankia_sp.                       TAPGGLLALADRLDLLVGLFALDAAPTGSSDPFGLRRAALGVAAIAGFAA
68230323_Frankia                           TVPGALLALADRIDLLAGLFALDAAPTGSSDPFGLRRAALGVTAIASFAR
28572421_Tropheryma_whippl                 TQAGAILSIADRLDQLVAMTALGITSTGSSDLFGVRRTASGLVDISQFLE
46399968_Parachlamydia_sp.                 TETGTIISLADKLDNLIGCFSIGLKPTSSSDPYALRRQVLGVIKMSQFHS
C_130039_Chlamdydomonas_reinh              SPAGVLVAVADRLDSLVGLFAAGCAPSAATDPFALRRAAYGMLQTGRLRK
15836479_Chlamydophila_pneumo              STIGTLLSLLDRLDNLLACFILGLKPTSSHDPYALRRQSLEVLTLEKLAV
15605530_Chlamydia_tracho                  STTGALLSILDRIDNLLSCFILGLLPTSSHDPYALRRQSLEILTLQILKT
55296761_Oryza_sativa                      TDPGIVLAVTDRLDSLVGLFGAGCQPSSTNDPFGLRRISYGLVQIEDFPK
30692978_Arabidopsis_thalia                TDAGMVLAIGDRLDSLVGLFAAGCQPSSTNDPFGLRRISYGLVQIEMFPK
CM269_Cyanidioschyzon_merolae              TPAGVALALSDRMDSLVGLFGIGLAPKANSDPFGLRRAALGVVQIERFRD
34482366_Wolinella_succin                  SPFSAILALAHRLDNLMGLFSLGRIPTGSKDPFALRRAASGVLRISEGKS
15611973_Helicobacter_pylori               SVFSSIVALSLKIDSLFSLFSVGKIPSGSKDPFALRRLSFGLLKIQKKEL
15607085_Aquifex_aeolic                    TTTGTILSLSDKIDNLYSFFKAGEIPKGSSDPYGLRRSAFGIIKIPEFPS
66855634_Anaeromyxobacter_deha             SDLGALVAVADRLHSLVGIIGVGEKATGAADPFGLRRAAIGILRIPDFGS
46580306_Desulfovibrio_vulgar              SMCGALLSIADKADTLAGCFGLGMIPTGAADPYALRRCVLGIARIDDFAS
4980715_Thermotoga_mariti                  TVIGSILGIADRIDTIVGNFAIGNVPTSSKDPYGLKSKADTIFRIPEFQD
```

```
68207212_Desulfitobacterium_ha      SYTGQIVSVADKLDAIVGAFGIGIQPTGSQDPYALRRQAQGVVGITEFVP
50914778_Streptococcus_pyogen       TKVGAVLALADKLDTLLSFFSVGLIPSGSNDPYALRRATQGIVRIENYKP
17133246_Nostoc_sp.                 TLTGQVVGLADRLDTLVSIFGLGLIPTGSSDPFALRRAANAVVNIGTLDK
45508785_Anabaena_variab            TLTGQVAGLADRLDTLVSIFGLGLIPTGSSDPFALRRAANAVINIGTLDK
72382003_Prochlorococcus_marin      NNLGNAVSLAERFELLISIYAKGERPSGSSDPYALRRAANGILLINTLNL
46907686_Listeria_monocy            TDLGSLIAIADKLETLIGFFCVNIAPTGSADPFGLRRSAFGAVRIEWFRP
23099403_Oceanobacillus_iheyen      TVIGSVVSVADKLDTIAGCIAVGLVPTGSQDPYGLRRQASAILRIETFKP
68179078_Desulfuromonas_acetox      DNVGAFVSIADKIDTICGCFGVGLIPTGTADPYALRRCAIGVLNIPEFEA
39982449_Geobacter_sulfur           SDIGAFVSIADKLDTICGCFGVGLIPTGSADPYALRRSALGIINIPDFDS
51891713_Symbiobacterium_therm      TGPGIVVALADKLDTLAGYFSIGLIPTGSQDPFALRRAAQGVVQTPEFAA
67931182_Solibacter_usitat          NATALIVSLADKLDTLRGCFGVGMVPTGSKDPFALRRAAQGVVRIPDFEP
1573946_Haemophilus_influe          SLVASAVALADKFDTLTGIFGIGQAPKGSADPFALRRAALGALRIDSAEA
26986805_Pseudomonas_putida         TLTGAAVAIADKLDTLVGIFGIGMLPTGSKDPYALRRAALGVLRIPEAEA
13476182_Mesorhizobium_loti         DPVSVAVALADKLDTLVGFWAIDEKPTGSKDPYALRRAALGVVRIEDGKN
39934122_Rhodopseudomonas_palu      DPVSIAVALADKLDTLVGFWAIDEKPTGSKDPYALRRAALGVIRLDDGKN
23013833_Magnetospirillum_magn      APVSVAVALADKIDSLVGFFAINERPTGSKDPFALRRAALGIIRLDDGAN
68213210_Methylobacillus_flage      NKVGMIVALADKLETLAGLFSIGEKPTGEKDPFALRRHAIGILRIPEAAS
                                       .   .: : . : .    .  ...  * :.::

68172611_Frankia_sp.                LAAALQRVRRIVPDLPAAVDAFFDAVMVMADDPAVRAARLGLLAWIRDLT
68230323_Frankia                    LAAALGRVRRIVPQLPDAVDGFFDAVLVMADDPALRAARLGLLAEIRDVA
28572421_Tropheryma_whippl          IVQCLKRIDRICKNLPEAVNNFFDNTLVMTPEEEIRTARLGLLCAIRSYS
46399968_Parachlamydia_sp.          LYEVYKRAKGQLNTMQPALAVLFEQVKILADEPQIRDNRLALLKQVFNFF
C_130039_Chlamdydomonas_reinh       VMAAFSRPTRIVREPLLSPIDKFFEVFVMCEDQAVRANRLALLRDLAALP
15836479_Chlamydophila_pneumo       ITTTHNRLKKILSDLSNDIQDFLNTVHIANDDGAIRNLRISLLLTAMDKF
15605530_Chlamydia_tracho           IAAMYNRLKKILAELAQSTELFLDSVRVASDDESTRNQRIALLIAAQKCF
55296761_Oryza_sativa               IVEAYSRPARIIRELLQPLEDFFTNVFVMAEDERVRNNRLALLTKVASLP
30692978_Arabidopsis_thalia         IVEAYSRPTRIVRQLVEPLEDFFNNVFVMVEEERVRKNRLALLNNIANLP
CM269_Cyanidioschyzon_merolae       AVTCYLRPARLVRTLRPSVDAFLDGVMVMVDEPALRRNRLNLCARIARIP
34482366_Wolinella_succin           LSTTFKRVANITKKLKEPLDQFFDHVLINCDNTLLKKNRQALIAHIYQAF
15611973_Helicobacter_pylori        LFSAFKRLANINKGLHAPLEEYFKSVLVMDKDIEIQKNRKNFLWGVYQSF
15607085_Aquifex_aeolic             VVEAAKRVIRIIPPLKEYIDKFFDNVKVMAEDEKIRNNRISLLKRVENLF
66855634_Anaeromyxobacter_deha      LAVAFKRVANIQETLEPAVARFFDDVLVMAEDPALRANRLGLMRRVGALF
46580306_Desulfovibrio_vulgar       AVLTFKRAANIIRELRPVVDGFFEGVMVMSDDAALRTNRLNLLQALVGRL
4980715_Thermotoga_mariti           LFVGFERVHNIT-ELKPYIDEYFDNVFVMVKRDDLRVNRLSFLKNIDELF
68207212_Desulfitobacterium_ha      YSQAYIRCLNLSKELIPMIEALFNAVMIMVEDEILKQARLALLGECVAIL
50914778_Streptococcus_pyogen       AVESLSRAFNLAEVLSPVINDFFDNTMVMAEDQALKNNRLAILSDLVSKA
17133246_Nostoc_sp.                 IYETVNRSTRLAAEIAPTVSNFFDGVLVIDPDPEIKRNRLNILGLLRNHA
45508785_Anabaena_variab            IYETVNRSTRLAAEIAPTVSNFFDGVLVIDPDPEIKRNRLNILGLLRNHA
72382003_Prochlorococcus_marin      LQAVVTRASRLAASSTETLSNFFDGVMVMTEDINLRNNRLNLLTLLRNQS
46907686_Listeria_monocy            TIEALTRVVNISKDLRTTIDAYFDNTLVMTDNDELKNNRLALLFELASFI
23099403_Oceanobacillus_iheyen      IQEALVRVLNLSNQLAAPIHAFFDNNMVMADDLEIRNNRLALIQALTSLI
68179078_Desulfuromonas_acetox      LAATFKRVGNIIKSLRPAVDAFFDGVMVMAEDEAVKRNRLALLTQVAGLF
39982449_Geobacter_sulfur           LAVAFKRVCNIVKALKGAVDDFFDKVMVMAEDERVRTNRLALLTGIARLF
51891713_Symbiobacterium_therm      VTGAFKRVANLAGRLKAPVDAFLDTVRVNVEDEKVRANRYALLQALGGLL
67931182_Solibacter_usitat          LAASFKRIRNILERLRPRVDLFFDKVLVNAPDPVVRQNRLTLLSTLLAEF
1573946_Haemophilus_influe          LAAANKRVSNILANLRAPVDSFFDNVMVNAEDPALRQNRLAILNTLQGLF
26986805_Pseudomonas_putida         LAAVNKRVSNLLSALRDPVDAFFEAVMVNAEDAKVRANRYALLSRLRGLF
13476182_Mesorhizobium_loti         LLAGTKRAANILAALREPVDSFFEGVLVNDEDLEVRANRLALLTRIRAAT
39934122_Rhodopseudomonas_palu      LLAGTKRAANILATLRSAVDSFFAKIKVNADDKAVRENRLKLLNEIRAAT
23013833_Magnetospirillum_magn      LLIAYRRAANIVRRLRRPVDAFFDKVTVNADEADLRVNRLKLLAMIGSAM
68213210_Methylobacillus_flage      LAAANKRVGNILKALKAPVDAFFDHVMVNAEDPALKTNRLALLATLHQAM
                                          *               :    :       :  *  :
```

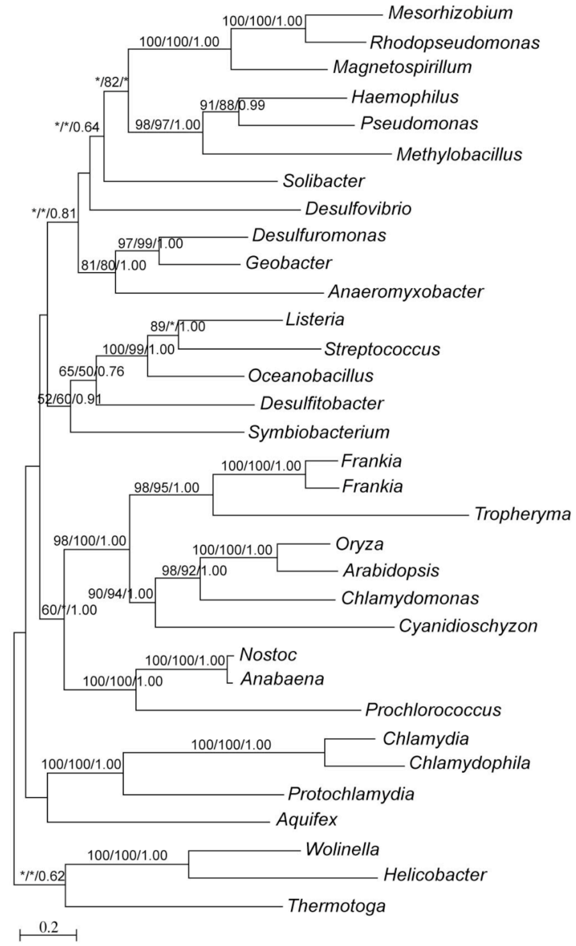

Figure 2B. Molecular phylogeny of beta subunit of glycyl-tRNA synthetase. P-value = 0.731 from AU test for the presented tree. AU tests were also performed on alternative topologies, including (**A**) monophyly of cyanobacterial and photosynthetic eukaryotic sequences, and (**B**) monophyly of chlamydial, *Frankia*, *Tropheryma*, and photosynthetic eukaryotic sequences. These tests investigate different scenarios, including a) plastdic origin of photosynthetic eukaryotic sequences, and b) photosynthetic eukaryotes acquired this gene from chlamydiae and subsequently spread to *Frankia* and *Tropheryma*. P-values = 0.085 and 0.446 from AU tests for these alternative topologies respectively. Therefore, the scenario of a plastidic or chlamydial origin of this gene in red algae and green plants cannot be confidently rejected based on these statistical tests. However, given that the two glycl-tRNA subunits likely acquired from a single event, we have chosen to present this gene here as a likely case of HGT.

---

3. Dihydrodipicolinate synthase (dapA) (D)

CLUSTAL X (1.83.1) multiple sequence alignment

```
15605085_Chlamydia_tracho         SVLGACITPFKIDFAALESVVRSQEHAG-NGIILFGSTGEGLSLTYEEKL
88858626_Pseudoalteromonas_tun    PLWTALITPFTVDFDALAKLVAEQVAAN-NGILLLGSTGEGLALTETEQK
119468235_Alteromonadales_bact    ALWTALVTPFDVDYDTLTTLVSEQEAAH-NGILLLGSTGEGLALTLKEQQ
77359151_Pseudoalteromonas_hal    SVWTALVTPFNIDYGTLTTLVAEQAAAN-NAILLLGSTGEGLALTLKEQQ
83765535_Aspergillus_oryzae       GFYVPTVAFFAVDVSTVEKHAAYLAQSGITGLVVQGSNGEAVHLDRDERK
71281575_Colwellia_psychr         GVYPAVTTQYNINFEATTQMVDTIIKEGVNGIIALGTVGENASHTREEKV
31543060_Homo_sapien              GIYPPVTTPFTVDYGKLEENLHKLGTFPFRGFVVQGSNGEFPFLTSSERL
119720569_Thermofilum_penden      GVISPFITPFRLDREAVAWLARYQAEKGVHGIFPNSTTGEFVHLSREEAV
83770010_Aspergillus_oryzae       GVMVALITPFTIDEGRLKSHIDRLLQAGVHGLVPGGSTGEFTVLSLAERK
33594549_Bordetella_pertus        GIWIPLVTPFAVDLPALRRLVRDYKAAGVDGLVACGSTGEAAALDEAEQL
84321353_Pseudomonas_aerugi       GIWVPLVTPFRIDFAALPRLLDHLLNAGVAGVVVCGTTGEAAALEHGEQL
126701253_Clostridium_diffic      GIICAMITPFDINSQATCQLIDYLIEKGIYGLFILGTNGECHVLTDDEKV
CDL00000863_Cyanophora_paradox    ALVTALKTPFLIDIDTFDGLVKEQMKAGVEGLVVGGTTGEGHLMNESELL
71279937_Colwellia_psychr         SLMTAIKTPYTVCLKTYDFLIEQQISAGVDGIIVGGTTGEGHLLSWEEHL
88857450_Pseudoalteromonas_tun    SLITAIKTPYQIDLNAYDQLVLAQIDADVDGIVVGGTTGEGQLMNWEEHL
119468748_Alteromonadales_bact    SLITAIKTPYLIDLAKYDKLVEIQIAAGVDGIVVGGTTGEGQLMNWEEHL
77362300_Pseudoalteromonas_hal    SLITAIKTPYLIDLATYDRLVELQIAAGVDGIVVGGTTGEGQLMNWEEHL
116057686_Ostreococcus_tauri]     RLITAIKTPYLVDLYAYDALVEAQIEGGVEGLIVGGTTGEGQLMSWDEHV
115457444_Oryza_sativa            RVITAVKTPYLFDLEAYDSLINMQIDGGAEGVIVGGTTGEGHLMSWDEHI
115460042_Oryza_sativa            ----------------------MQIEGGAEGVIVGGTTGEGHLMSWDEHI
15225477_Arabidopsis_thalia       RVITAIKTPYLFDLEAYDDLVNIQIQNGAEGVIVGGTTGEGQLMSWDEHI
30695296_Arabidopsis_thalia       RVITAIKTPYLFDLQAYDDLVNTQIENGAEGVIVGGTTGEGQLMSWDEHI
HVL00003027_Hartmanella_vermif    GVFAPIPTCFDLAIKDFKHNISRFNKTKLDGLVIMGSYGEFPYLSMSEKK
46446320_Candidatus_Protoc        GLYTALITPFTLDEEGLKKLIQIQLHHQVDGVVVLGTTGESPTLTQIEKR
118464138_Mycobacterium_avium     TVLTAMVTPFALDTAAAARLANHLVDSGCDGLVVSGTTGESPTTTDDEKR
21224090_Streptomyces_coelic      RVLTAMVTPFTLDLDGAQRLAAHLVDAGNDGLIINGTTGESPTTSDAEKA
15614962_Bacillus_halodu          HVLTAMVTPFDVDLEKVERLVEFLLANGTEGLVVAGTTGESPTLREEEKL
88933169_Dehalococcoides_sp.      RLITAMVTPFKVDYAQAQKLALGLLDSGSDGLVVVGTTGESPTVTWEEEH
CM654_Cyanidioschyzon_merolae     RVITALVTPFKVDYGVAESLAAHLAENGSDAIIVAGTTGESATLTWSEEY
67923140_Crocosphaera_watson      KVITAMVTPFDVNYEMAEKLAVHLVNNGSDSLLVSGTTGESPTLSWSEKC
72383089_Prochlorococcus_marin    RLLTAMVTPFDVDYGLAADLANYLVDQGSDGIVVCGTTGESPTLSWQEQQ
56752256_Synechococcus_elonga     RVITAMITPFNVAYDIAQSLAVHLVEQGSDGLVICGTTGESPTLTWEEEL
37520587_Gloeobacter_violac       RVLTAMITPFDVHYAEAEKLADYLAAHGSDTLVVCGTTGESPTLSWEEEY
110637717_Cytophaga_hutchi        GTGTALITPFKVDYDAFKNLIEFNISSGVDYIVVNGTTAESATTSKEEKA
53713695_Bacteroides_fragil       GMGVALITPFKVDYDALMRLVDYLLQNNADFLCVLGTTAETPTLSEEEKK
15644269_Thermotoga_mariti        GVGTAIVTPFKLDLESYERLVRYQLENGVNALIVLGTTGESPTVNEDERE
55377124_Haloarcula_marism        GVFPAMCTPFHIDFETLRDDAQRLESAGVDGLVPVGSTGESATLSHDEHI
20093258_Methanosarcina_acetiv    GAMPALITPFTIDREGLRRNIAFVEEGGVSGIVPCGTTGESATLSALEHE
67918442_Chlorobium_limico        GSAVALVTPFRVDTVALRQLVRFHIAAGTDIIIPCGTTGESPTLSMEEQS
94969520_Acidobacteria_bacter     GCGTALVTPFKVDFAAQRALVEWQIESGIDFLVPCGTTGETPTLSHDEWL
32472136_Rhodopirellula_baltic    GLSTAIVTPFRVDEKRLAEQIDFQIDSGVTCIVPCGTTGESPTLTHDEHE
23501533_Brucella_suis            GSITALVTPFDFDEKAFRAFVNWQIEEGTKGLVPVGTTGETPTLSHDEHK
113932881_Caulobacter_sp.         GVFPALVTPFRVDEKTFVALVERQIAGGVHGLVPVGTTGETATLSHDEHK
24216005_Leptospira_interr        GVYTAIITPFKIDYDSYFKLLEKQIKAGVSGVVPCGTTGESPTLSHSEHA
126700845_Clostridium_diffic      GSAVALVTPFTVDFDKLGELVEYQIANGTDAIVSCGTTGEANTMTDEEQL
GTL00002039_Guillardia_theta      GSYVALITPFTVDYAKIEELVEWHIAEGTAGIVPCGTTGESPTLTEEENK
ELL00008008_Euglena_gracilis      ----------------------------------------------EETK
16130403_Escherichia_coli         GSIVAIVTPMDVCRASLKKLIDYHVASGTSAIVSVGTTGESATLNHDEHA
84316486_Pseudomonas_aerugi       --MVALVTPFDLDWDSLAKLVDFHLQEGTNAIVAVGTTGESATLDVEEHI
33592660_Bordetella_pertus        GSLVALVTPMQLDYDAYRSLIDWHVAEGTDGLVVVGTTGESPSVSMEEHA
15606400_Aquifex_aeolic           GSIVALITPFKVDYEALGNLIEFHVDNGTDAILVCGTTGESPTLTFEEHE
88935908_Geobacter_uraniu         GSIVAIVTPFKVDEEKLRELVEFQIENGTDAIVPCGTTGESSTLDYEEHD
                                                                              *

15605085_Chlamydia_tracho         SILSFVSTLVPIFVGVTATSVQETMSWIDFAQQWPIDGFLVPTPLYTRPG
88858626_Pseudoalteromonas_tun    EIVTFVCQLVPLMVAVGGFNLKQQVQWLEFCNDLPIHAYLLGSPLYAKPG
119468235_Alteromonadales_bact    AIVEHVCQLVPLMVAVGGSNLAQQKEWVSYCNQLPIHSYLLGSPLYAKPG
77359151_Pseudoalteromonas_hal    AIVEHVCQLVPLMVAVGGMNLKQQLAWVEYCNHLPIHSFLLGSPLYAKPG
83765535_Aspergillus_oryzae       TITAATLDAMPVIVGCGGSSTRETIQLCKDAGESGGDYALVLPPCYSLVS
71281575_Colwellia_psychr         AILKAVKEVVPVLSGVAETSTQFAIEFSQACEAIGIDGLMVLPGMIYKSD
31543060_Homo_sapien              EVVSRVRQARLLLAGSGCESTQATVEMTVSMAQVGADAAMVVTPCYGRMS
119720569_Thermofilum_penden      EVTRLVLEAVWVIPGISANYTEDSVALGRTFKDLGVDGAVVTPPYFFKVS
83770010_Aspergillus_oryzae       QLTELCVKYVPVVAGTGATSTQEAVELAKHAGEVGAAAVMVVPPFYDPVN
33594549_Bordetella_pertus        AVLDAVLEELPVAMGLAGNHQDHVLRRLARLGERPLAGVLAPA-------
84321353_Pseudomonas_aerugi       QLLDAVLERERVAMGLAGNNQAQLLAFQREVLERPLAGLLVPAPYYIRPS
126701253_Clostridium_diffic      EFAKIVINHVPVFVGTGGNSTREVVDLSKRMEAIGASALSIITPYFVTPT
CDL00000863_Cyanophora_paradox    DLIQHSVEKLVIIGNTGGNNTSKALKLTKQGFAKGMHCALQINPYYGKTS
71279937_Colwellia_psychr         MLIAHCVNNLIIVGNTGSNNTREAIKATENGFAMGMDAALQINPYYGRTS
88857450_Pseudoalteromonas_tun    MLIAHSVAKLIIVGNTGSNNTREAIKATQYGFATGMDAALQINPYYGRSS
119468748_Alteromonadales_bact    MLIAHSANKLLIVGNTGSNNTREAIKASKYGFASGMHAALQINPYYGRTS
77362300_Pseudoalteromonas_hal    MLIAHSVNKLVIVGNTGSNNTREAIKATKYGFASGMDAALQINPYYGRTS
```

```
116057686_Ostreococcus_tauri]       MLIAHTAQKVLVIGNTGSNSTREAVHATSQGFAVGMDASLQINPYYGKTS
115457444_Oryza_sativa              MLIGHTVNCVKVVGNTGSNSTREAIHATEQGFAVGMHAALHINPYYGKTS
115460042_Oryza_sativa              MLIGHTVNCIKVVGNTGSNSTREAIHATEQGFAVGMHAALHINPYYGKTS
15225477_Arabidopsis_thalia         MLIGHTVNCIKVIGNTGSNSTREAIHATEQGFAVGMHAALHINPYYGKTS
30695296_Arabidopsis_thalia         MLIGHTVNCIKVIGNTGSNSTREAIHATEQGFAMGMHGALHINPYYGKTS
HVL00003027_Hartmanella_vermif      QLLKAAKEAM-LIAGTGSTNFRETLELNHYAKELGYDAVMVITPYYSKMN
46446320_Candidatus_Protoc          RIIEIALEEIKVIVGTGSYSTQQAIEQTLQAKQMGADAALIVTPYYNKPT
118464138_Mycobacterium_avium       ELLRVVLEAARVIAGAGTYDTAHSVRLAKACAAEGAHGLLVVTPYYSKPP
21224090_Streptomyces_coelic        DLVRAVVEAAHVVAGVGTNNTQHSIELARAAERVGAHGLLLVTPYYNKPP
15614962_Bacillus_halodu            ALFEKVVTVVPVIAGTGSNNTYVSAQLTKKATALGVDGIMAVTPYYNKPS
88933169_Dehalococcoides_sp.        ALFAAVKSAGKVIAGTGANSTQEALENTLKAEKIGVDACLLVVPYYNKPT
CM654_Cyanidioschyzon_merolae       ELFRVVKSACRVIAGAGSNSTEEAIEATKKSAKLGLDGTLQVVPYYNKPP
67923140_Crocosphaera_watson        QLFKVVKKAAKVLAGTGGNSTQHAIEMTQEVAKMGLDGSLQVVPYYNKPP
72383089_Prochlorococcus_marin      KLLETVRNSAKVLAGTGSNSTSEAIEATKEAANSGADGALVVVPYYNKPP
56752256_Synechococcus_elonga       QLFRAVKEAAAVIAGTGSNCTREAIAATQSAATLGLNGSLQVVPYYNKPP
37520587_Gloeobacter_violac         ELFDVVRRAAKVVAGTGSNATREAVVATRKAAALGLDGTLQVCPYYNKPT
110637717_Cytophaga_hutchi          KLLEVALDVVPVMYGIGGNDTAEVIQRIGSADFKGVDALLSVCPYYNKPS
53713695_Bacteroides_fragil         KIKKMVIDRIPILLGVGSNNTRAVVETLKNDDFTGVDAILSVVPYYNKPS
15644269_Thermotoga_mariti          KLVSRTLEIIPVIVGAGTNSTEKTLKLVKQAEKLGANGVLVVTPYYNKPT
55377124_Haloarcula_marism          EVVEAVIDAVPVIAGSGSNNTKEALELSRRSAEAGADALLLISPYYNKPE
20093258_Methanosarcina_acetiv      EVIDIAVECVPVVAGTGSNNTGEALQFTKHAADAGVDGVLLISPYYNKPN
67918442_Chlorobium_limico          RIIRTVKEEIMVAAGAGTNATDHAVELAKNAEKAGASAILSVAPYYNKPS
94969520_Acidobacteria_bacter       KVIAQTVEVVPIVAGATSNNTAEAVEKAKEVAAIGVDAILTASPYYNKPT
32472136_Rhodopirellula_baltic      RVISLTIQHVKVMAGTGSNSTAEALRLTRRAADEGADATLQVAPYYNKPT
23501533_Brucella_suis              RVIEVCIEVVPVIAGAGSNNTVEAIELAQHAEKAGADAVLVVTPYYNKPN
113932881_Caulobacter_sp.           RVVELCVQTVPVIAGAGSNSTAEAIELVAHAKAVGADAALVVSPYYVRPS
24216005_Leptospira_interr          ELIRETVKAIQVVAGTGSNSTKEAIELTEAACKDGVDGILSVNPYYNKPT
126700845_Clostridium_diffic        ATIKYVVEKVPVIAGSGSNDTMHSVNLSQEAEKLGVDALLIITPYYNKAN
GTL00002039_Guillardia_theta        KVIETVVKKCHVVAGAGGNCTKKVVAFTKWCKEIGADCVLSVIPYYNKPT
ELL00008008_Euglena_gracilis        KVIETVVKKCKVVGGGGSNSTAKAVKAAQMCKDLGCDVVLSVIPYYNKPT
16130403_Escherichia_coli           DVVMMTLDLIPVIAGTGANATAEAISLTQRFNDSGIVGCLTVTPYYNRPS
84316486_Pseudomonas_aerugi         QVIRRVVDQIPVIAGTGANSTREAVALTEAAKSGGADACLLVTPYYNKPT
33592660_Bordetella_pertus          ELIRVAVEHIPVIAGVGANSTDEAIHLARHAKAVGAQAGLSVVPYYNKPN
15606400_Aquifex_aeolic             KVIEFAVKRIKVIAGTGGNATHEAVHLTAHAKEVGADGALVVVPYYNKPT
88935908_Geobacter_uraniu           RVIEIVVEQVPVIAGTGSNSTKEAIEMTSHAKTLGADGALLVTPYYNKPT
                                               :

15605085_Chlamydia_tracho           LNGQKAWFDRILSVSPIILYNNPIRTGVSLYPEVVKSFHPLCIGVKDSGG
88858626_Pseudoalteromonas_tun      IQGQTQWFDTLLNHSPCMLYNVPGRSAVAIAPEVLTNLHTRLWAMKEASG
119468235_Alteromonadales_bact      AVGQTLWYESLLNESPCMLYNVPGRSGVSIPIETIENLHPKLWALKEASG
77359151_Pseudoalteromonas_hal      VIGQTHWFESLLNISPCMLYNVPGRSAVDIPVATIQNLHKNLWALKEASG
83765535_Aspergillus_oryzae         TEALRDHFRAVASASPVLIYNFPGASGLDLTSDDILALHPNIVGVKLTCG
71281575_Colwellia_psychr           ESEAVNHYQQIARNCPIMIYNNPVTYGVDVGIEGMKILEDNIVSIKEATE
31543060_Homo_sapien                SAALIHHYTKVADLSPVVLYSVPANTGLDLPVDAVVTLHPNIVGMKDSGG
119720569_Thermofilum_penden        PERLKVHFSTILEKVPIIVYNIPATTGINIPVGLYLELHSNLAGAKATVE
83770010_Aspergillus_oryzae         YEQLTEMMSEIHTESPIMYYNIPSASGLTLTPQQIADLKVGVKYLKDTSG
33594549_Bordetella_pertus          ---------------PLILYDIPYRTGAQLDTATLLALHPNIAAVKDCGG
84321353_Pseudomonas_aerugi         QEALVRFFHGVADASPLIVYDIPYRTGVRMELETLRRIHPRIAAIKDCGG
126701253_Clostridium_diffic        QQELILHYKTIANSVPIIMYNMPGKTGINIEPESVRELIKNIVGIKDSSG
CDL00000863_Cyanophora_paradox      DAGVLEHLGRLLAEGPGIVYNVPGRTGQDIKPAVVEKL------------
71279937_Colwellia_psychr           IRGVSEHFKRVLDIGPAFIYNVPGRTGQDLTPEIIEPLHKNFIGVKECSG
88857450_Pseudoalteromonas_tun      IAGVKEHLSRVLALGPAFIYNVPGRTGQDLTPEILIPFHPNFIGIKECAG
119468748_Alteromonadales_bact      IAGVKEHFKHVLDIGPAFIYNVAGRTGQDLTPDIIEPLHPNLIGVKECGG
77362300_Pseudoalteromonas_hal      IAGVKEHLKRVLDIGPAFIYNVAGRTGQDLTPDIIEPLHEHFIGVKECGG
116057686_Ostreococcus_tauri]       RRGLIEHFGAVMDLGPAIVYNVPARTSQDIAPEVMFELHKNFAGVKECEG
115457444_Oryza_sativa              IEGLISHFEAVLPMGPTIIYNVPSRTGQDIPPAVIEAVFTNLAGVKECVG
115460042_Oryza_sativa              VEGLISHFEAVLPMGPTIIYNVPSRTGQDIPPAVIEAVFSNMAGVKECVG
15225477_Arabidopsis_thalia         IEGLIAHFQSVLHMGPTIIYNVPGRTGQDIPPRAIFKLNPNLAGVKECVG
30695296_Arabidopsis_thalia         IEGMNAHFQTVLHMGPTIIYNVPGRTCQDIPPQVIFKLNPNMAGVKECVG
HVL00003027_Hartmanella_vermif      DATLEAYYSKLADAVPLILYNMPSYSGINMSVSVVSKLHKNI--------
46446320_Candidatus_Protoc          QEGIFKHFEAINQAVPICLYNIQGRTGQNIQTHTLKRILSSIIGVKETSG
118464138_Mycobacterium_avium       QRGLIAHFTAVADATPVLLYDIPGRSVVPIESSTIRALHPNIVGVKDAKA
21224090_Streptomyces_coelic        QEGLYLHFTAIADAAPVMLYDIPGRSGVPINTETLVRLHPRIVANKDAKG
15614962_Bacillus_halodu            QEGMYAHFAAIAEATPVMLYNIPSRSVVNLSAETIVKLIPNITSLKEANG
88933169_Dehalococcoides_sp.        QEGLYLHFKTIAEATPCILYNVPSRTITHMNPETVIRLIPNIVGIKEASG
CM654_Cyanidioschyzon_merolae       QQGIMAHFRAIANAAPMMLYNIPGRTGINMTAETSIKLCPNIVALKEASG
67923140_Crocosphaera_watson        QEGLYEHFKKIAETVPIMLYNIPGRTGTNMSPETTAKLIDNIVAIKEASG
72383089_Prochlorococcus_marin      QEGLEVHFRAIANAAPLMLYNIPGRTGCSISPSIVSKLCSNVVSFKAASG
56752256_Synechococcus_elonga       QEGLYAHFRAIAEACPLMLYNIPGRTGQSLQPETVARLLPNVVAIKAASG
37520587_Gloeobacter_violac         QDGLFEHFRAVAQAGPVILYNVPGRTGVHLAPETVARLVPGIVAIKEASG
110637717_Cytophaga_hutchi          QEGVFQHYKAIAEKCPVLAYNVPGRTGINISAKTSIRLVKNIFGIKEASG
53713695_Bacteroides_fragil         QEGIYQHYKAIASATPIVLYNVPGRTGVNMTAETTLRIFQNVIAIKEASG
15644269_Thermotoga_mariti          QEGLYQHYKYISERTGIVVYNVPGRTGVNVLPETAARILKNVVGIKEANP
```

```
55377124_Haloarcula_marism          QQGFIDHYTTLADAVPQIVYNVPSRTGQNIEPDTAAELHPNIRAYKAASG
20093258_Methanosarcina_acetiv      PAGLLAHFKKIAEAVPMILYNVPSRTGQDMPVDVITKLVENIVGIKEASG
67918442_Chlorobium_limico          QEGIYQHYARIAEAVPIIIYNVPGRTGSNVAASTILRLFGNIAAVKEASD
94969520_Acidobacteria_bacter       QEGQYLHFKAIAEAVPLVLYNVPGRTAANIETATLLRLIPNIIAVKEASG
32472136_Rhodopirellula_baltic      QEGMYQHFRAVAEAVPVCVYNIPGRSAKNIEVDTIVRLLPNITMVKEATG
23501533_Brucella_suis              QRGLYEHFSRVVRSIPLVIYNIPGRSIIDMTPETMGALCKNIVGVKDATG
113932881_Caulobacter_sp.           QEGIYQHYKAINDAVPVLVYNVPGRTGSDVANETLARLLPNIVGIKDATG
24216005_Leptospira_interr          QEGLFQHFKSIAEHSPVMLYNIPGRTSVNLLPETVLRLVKQIRSMKEATG
126700845_Clostridium_diffic        KAGLKRHFETIANSVPIILYNVPGRTCVNISPSLIVELIDNIVAVKEASG
GTL00002039_Guillardia_theta        QDGLYGHFKAVADVGPVMLYNIPGRCGGGLTSATIAKLCPTICAVKEATG
ELL00008008_Euglena_gracilis        QDGLYAHFSEVAKVGPIMLYNIPGRCGGGLTSATIAKLLPMVCSIKEATG
16130403_Escherichia_coli           QEGLYQHFKAIAEHTPQILYNVPSRTGCDLLPETVGRLVKNIIGIKEATG
84316486_Pseudomonas_aerugi         QEGMYQHFRHIAEAVPQILYNVPGRTSCDMLPETVERLVPNIIGIKEATG
33592660_Bordetella_pertus          QEGIYRHFRAVAEAVPTVLYNVPGRTVADMSNDTVLRLVPGIIGIKEATG
15606400_Aquifex_aeolic             QRGLYEHFKTVAQEVPIIIYNIPSRTCVEISVDTMFKLCENIVASKESTP
88935908_Geobacter_uraniu           QEGLFLHYKAVADAVPQILYNVPGRTGVNLLPETVARLHKNIVAIKEATG
                                                       *.        :       .

15605085_Chlamydia_tracho           SLRVFCGDDNLWPDMRLSGASGVISVLANVWPELARDYVAQGEAWKKVCS
88858626_Pseudoalteromonas_tun      DLAIYSGDDALMPYFAQAGAKGLVSVAANAWPNQTAEFVRRSRQWSSAIN
119468235_Alteromonadales_bact      NLALYSGDDALMPYFAQAGAKGLVSVAANAWPKQTHEFVKRSAQWTRAIN
77359151_Pseudoalteromonas_hal      NLAIFSGDDALMPYFAQAGAKGLVSVAANAWPLQTHEFVQRSTQWSSAIS
83765535_Aspergillus_oryzae         NFLTFGGSADFTLQTLVAGGAGIIGGLGNLIPRSCVYVMKLYAVVARA-D
71281575_Colwellia_psychr           DFVIFGGVDDIALESLMLGCTGWISGLTNVFPKESVAIYKLARWFLPLLR
31543060_Homo_sapien                DFQVLAGSAGFLMASYALGAVGGVCALANVLGAQVCQLERLCHRLIEPNA
119720569_Thermofilum_penden        SFAVLTGLDDLLLPVLMMGGDGGIMALANAAPQIHREVYDAYHKLLRLVR
83770010_Aspergillus_oryzae         NITAFNGWDTLTFYGMAAGAPGCVWGAANVIPELAVQLWEAIAKAFPICK
33594549_Bordetella_pertus          SMAVLAGEDLQGLGTLCLGGTGMIAAAAHVRPDLFVAMHRAVLALAPVIR
84321353_Pseudomonas_aerugi         SVAVLAGEDLQMFNAFCLGASGAIAASAHLHPQRFVALHRQVFALLPLIR
126701253_Clostridium_diffic        KFSVFSGSDSLILDSLKAGSQGAVAATTNFLTEIGISIYENFSSIEELRR
CDL00000863_Cyanophora_paradox      --------------------------------------------------
71279937_Colwellia_psychr           NISCWSGNDDECYVGRVHGSHGVISVASNIIPGFMRQLMDKDDKLQGLMK
88857450_Pseudoalteromonas_tun      NIACWSGNDDEAHDARLFGSHGVISVTSNLVPNIMRQLMDKSTHTQPLMN
119468748_Alteromonadales_bact      NIACWSGNDDEAHDARVHKAHGVISVTSNLIPGLFRQLMDTKSSLQPLMN
77362300_Pseudoalteromonas_hal      NIACWSGNDDESHDARTHGAHGVISVSSNLMPELFRQLMDAPTSLQPLIN
116057686_Ostreococcus_tauri]       NVTCWTGNDDEVHEAREAGAVGVISVTSNLVPELMRELLFDGDRLLPLMG
115457444_Oryza_sativa              HITIWSGNDDECHDSRKYGATGVISVASNLIPGLMHDLMYEGEKLFPLMK
115460042_Oryza_sativa              HITIWSGNDDECHDSRKYGATGVISVASNLIPGLMRKLMYEGDKLLPLMK
15225477_Arabidopsis_thalia         NVVVWSGNDDECHDSRDYGATGVISVTSNLVPGLMRKLMFEGSKLLPLMA
30695296_Arabidopsis_thalia         NIVVWSGNDDQCHDSRDHGATGVISVTSNLVPGLMRKLMFEGAKLLPLMD
HVL00003027_Hartmanella_vermif      --------------------------------------------------
46446320_Candidatus_Protoc          DFAILSGDDALTLPMIALGGHGIISVVSNLVPAAMKSLVNAANQLYSFIK
118464138_Mycobacterium_avium       DLAYYSGDDALNLPWLAMGATGFISVISHVAANQLRELLSAFATVAPLCD
21224090_Streptomyces_coelic        DLAWYSGDDMLNLPLLAVGAVGFVSVVGHVVTPELRAMVDAHQKLLPVFT
15614962_Bacillus_halodu            DFHLYSGDDSLTLPALSIGAKGVVSVASHIIGPEMQQMMMAFRKLLPIMK
88933169_Dehalococcoides_sp.        KFTVWSGNDSDTLPMLAMGSYGVISVASHLVGNQIKDMITSFRHLTPLIR
CM654_Cyanidioschyzon_merolae       NFALYSGDDALTLPLLSLGGNGVVSVASHFIGPEIQRMIEHFCRYMDLFE
67923140_Crocosphaera_watson        NFLIYAGDDFLTLPLLTVGGVGVVSVASHLVGLQMQEMIAAYVKLFPLFK
72383089_Prochlorococcus_marin      TLAIYSGDDALVLPMLSVGAVGVVSVASHLVAPNLKKLIESFETLQPLFK
56752256_Synechococcus_elonga       NFAIYSGDDSLTLPMLAVGAQGVVSVASHLVGPQLQALIQAFHQLYPLFK
37520587_Gloeobacter_violac         SFVIYCGDDSLTLPMLAVGATGVVSIASHLVGEPIQQMIRAFLHLLPLFR
110637717_Cytophaga_hutchi          DFLVISGDDLLAVPTISLGAVGAISVLSNTYPAKFSEMIKAAARFTEMNP
53713695_Bacteroides_fragil         NFDVISGDDGITFPLITLGAVGVISVIGNAFPREFSRMTRLAHKFTELFN
15644269_Thermotoga_mariti          DFMVWSGNDDRTFYLLCAGGDGVISVVSNVAPKQMVELCAEYRKLRPLMK
55377124_Haloarcula_marism          DFAVLSGDDGMTLPMLSVGGTGCISVSANIEPERTCAMVGAAHELGPLFR
20093258_Methanosarcina_acetiv      SFVVLSGEDGLTLPIISVGGSGVISVAANIVPDKMSGMVSAAFEIAPLIR
67918442_Chlorobium_limico          NFSVLTGEDMLILPFMAMGGDGVISVAANQVPSAVKRLVEAATRYRRLFR
94969520_Acidobacteria_bacter       SFTVLSGDDALTLPILAVGGVGLVSVASNQIPKELSEMVRAANHFLALMN
32472136_Rhodopirellula_baltic      SLTVLSGDDSLTLPMMSVGAEGVISVAANLVPSVLLEMVQTAHRLYSLCS
23501533_Brucella_suis              KFIQLSGEDATALGFNAHGGVGCISVTSNIAPRLCAEFQEACDRLMPLHK
113932881_Caulobacter_sp.           DWVMLSGDDPTALGYVAHGGHGVISVTSNVAPDACATFINACDRLVRLHK
24216005_Leptospira_interr          DMTVLSGDDNLTLPLLAIGGVGVVSVISNLFPKALVQLVESFYDFIEVFA
126700845_Clostridium_diffic        DFAIYSGNDDTILPLLSLGGQGVISVLANVCPQETHDLVAKFLGMDALIA
GTL00002039_Guillardia_theta        SIQIFSGDDSLTVPLMSIGAKGVVSVAANLTPRKVADLCKAMSELYKLCK
ELL00008008_Euglena_gracilis        SIEVFSGDDSLTVPLMSVGAKGVVSVAANLVPKKMADLCKSMAELYKLCK
16130403_Escherichia_coli           NFVLLSGDDASALDFMQLGGHGVISVTANVAARDMAQMCKLAQRLMPLHN
84316486_Pseudomonas_aerugi         DFLVYSGDDATAVELMLLGGKGNISVTANVAPRAMSDLCAAADRLMPLHK
33592660_Bordetella_pertus          DFQVFSGDDPTAAALILLGARGNISVTANVAPRLMHELCTAAARLARLNK
15606400_Aquifex_aeolic             NFSVLSGDDSLTLPMMALGAKGVISVANNVMPREVKELIRAAYYLHDLFK
88935908_Geobacter_uraniu           SIDVFSGDDFITFPMMACGAKGVISVTANIMPKEVAALVDAFLKLLKISN

15605085_Chlamydia_tracho           WLNLSTNPLGIKALMAAQKMIECDVRPPLSIR
```

```
88858626_Pseudoalteromonas_tun      SLFEVANPIPVKVLMHKQGRIQFPLRPPLTHL
119468235_Alteromonadales_bact      SLFAVANPIPVKVLMHQQGRLSSPLRPPLTHL
77359151_Pseudoalteromonas_hal      SLFTVANPIPVKVLMHLQGRLTSSLRPPLTHL
83765535_Aspergillus_oryzae         WHAIKGGFVAVKSALQSYRGYGAQPRRPCVEP
71281575_Colwellia_psychr           LDTVPKLVQCIKLCEQLAGRGSELTRAPRMPL
31543060_Homo_sapien                AVTRRFGIPGLKKIMDWFGYYGGPCRAPLQEL
119720569_Thermofilum_penden        VYDATSFPTSVKTLLKVMGAPVKPARTPLTPE
83770010_Aspergillus_oryzae         FLESHNYAAAVKTGVELTGQPTGGLRKPFALL
33594549_Bordetella_pertus          LLFAEPNPAPLKALLASQGRLRNEMRAPMLPA
84321353_Pseudomonas_aerugi         AAFAEPNPAPVKAGLARQGLIGDELRAPLLGC
126701253_Clostridium_diffic        ILKLGTIPSVMKKTIVLNGINVGTARLPVTEP
CDL00000863_Cyanophora_paradox      --------------------------------
71279937_Colwellia_psychr           WLFCEPNPIAINTAMIMTDAVPNNFRLPYIAL
88857450_Pseudoalteromonas_tun      WLFCEPNPIAINTALMMTGAVKPVFRLPYVPL
119468748_Alteromonadales_bact      WLFCEPNPIAINTALMMTGAVNPVFRLPYLPL
77362300_Pseudoalteromonas_hal      WLFCEPNPIAINTALMMTEAVKPVFRLPYLPL
116057686_Ostreococcus_tauri]       WLFREPNPIGVNTATAMLGVAKPVFRLPYVPY
115457444_Oryza_sativa              WLFCQPNPIALNTALAQLGVVRPVFRLPYVPL
115460042_Oryza_sativa              WLFCQPNPIALNTALAQLGVARPVFRLPYVPL
15225477_Arabidopsis_thalia         WLFHEPNPIGINTALAQLGVSRPVFRLPYVPL
30695296_Arabidopsis_thalia         WLFQEPNPIGVNTALAQLGVARPVFRLPYVPL
HVL00003027_Hartmanella_vermif      --------------------------------
46446320_Candidatus_Protoc          AAFIETNPIPIKAALSLSKLPAGSCRLPLCDL
118464138_Mycobacterium_avium       AMARLGGVTMSKAGLRLQGIDVGDPRLPQVPA
21224090_Streptomyces_coelic        GMFRTQGVMTTKGALALQGLPAGPLRAPMVGL
15614962_Bacillus_halodu            GLFTAPNPTCVKAALQIKGFDTGGVRLPLVPP
88933169_Dehalococcoides_sp.        SLFVVSNPIPIKYALNYLGVEVGGLRLPMTEA
CM654_Cyanidioschyzon_merolae       ALFVMANPIPAKAALRLLGWPVGPTRLPLTDI
67923140_Crocosphaera_watson        VLFCTSNPLPIKAALNLQGWDVGGVRLPLVSI
72383089_Prochlorococcus_marin      SLFATTNPIPVKAALQLIGWSVGPPRSPLVSL
56752256_Synechococcus_elonga       ALFLTTNPIPVRAAMELLGWSIGLPRLPLVPA
37520587_Gloeobacter_violac         GLFWETNPIPVKLALALQGWDVNHLRLPLVAG
110637717_Cytophaga_hutchi          LMYEEGNPVGVKSLLELMGICSSEVRLPLVKA
53713695_Bacteroides_fragil         LLFVDGNPAGVKSMLNAMGMIENKLRLPLVPT
15644269_Thermotoga_mariti          ALFVETNPIPVKAALNLMGFIENELRLPLVPA
55377124_Haloarcula_marism          AMFVETNPIPVKEAMRIRGYGPAHLRSPLTRL
20093258_Methanosarcina_acetiv      ALFLETNPIPAKKAAELIGLASGHLRLPLAPM
67918442_Chlorobium_limico          LNFIESNPVPVKYALALMGMIEEVYRLPLVPL
94969520_Acidobacteria_bacter       ANFLESNPGPVKAVLAMMGRIEENYRLPMVPM
32472136_Rhodopirellula_baltic      KMLLATNPIPVKAAMQMVGRDTGEMRLPMVPL
23501533_Brucella_suis              ALFLEPNPSGPKYALSRLGRIENVLRSPMVTI
113932881_Caulobacter_sp.           ALFLDASPSPTKFAMAHLGLCEATTRLPITPC
24216005_Leptospira_interr          LAFMETNPIPIKAAMCWFGHCGPEIRLPLTPL
126700845_Clostridium_diffic        ALFIEVNPIPVKTAMNLLGFNVGDLRLPLAEM
GTL00002039_Guillardia_theta        AMFCETNPIPCKAAGAMMGKWKNNTRLPMTPM
ELL00008008_Euglena_gracilis        AMFCETNPIPC---------------------
16130403_Escherichia_coli           KLFVEPNPIPVKWACKELGLVATDLRLPMTPI
84316486_Pseudomonas_aerugi         ALFIESNPIPVKWALHEMGLIPEGIRLPLTWL
33592660_Bordetella_pertus          ALFIEANPIPVKWALAQMGHTALGYRLPMVEL
15606400_Aquifex_aeolic             VLFIETNPIPVKTACWMLGMCEKEFRLPLTEM
88935908_Geobacter_uraniu           AMFIESNPVPVKTAVALMGKCSAEVRLPLAPL
```

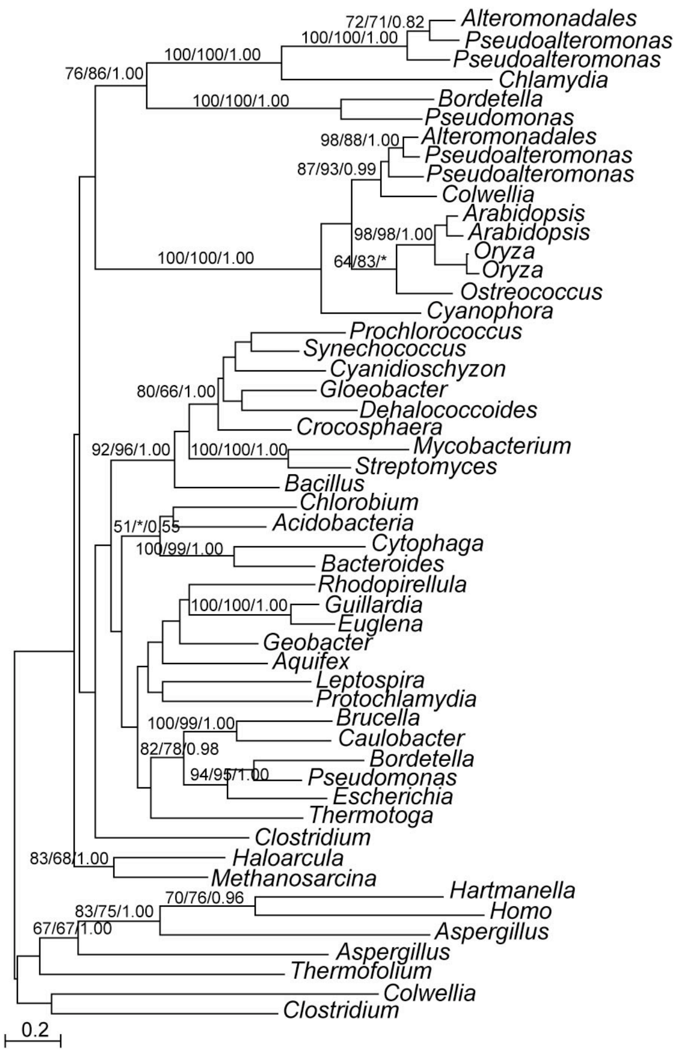

Figure 3. Molecular phylogeny of dihydrodipicolinate synthase (dapA). The red algal *Cyanidioschyzon* sequence forms a group with cyanobacterial and *Dehalococcoides* homologs with strong support whereas green algal and glaucophyte sequences form another strongly supported group with gamma-proteobacterial homologs. This could be explained by an HGT from gamma-proteobacteria and subsequent gene losses among primary photosynthetic eukaryotes. See text for explanations. Sequences of *Cyanophora*, *Guillardia*, *Hartmanella*, and *Euglena* were obtained from TBestDB. P-value = 0.199 from the AU test for the presented tree.

AU tests were also performed on alternative topologies, including (**A**) monophyly of all eukaryotic sequences except *Cyanidioschyzon* that appears to be plastid-derived, (**B**) monophyly of *Cyanidioschyzon* and cyanobacterial sequences, and (**C**) monophyly of all plastid-containing eukaryotic and cyanobacterial sequences. P-values < 0.001 from AU test for topologies **A** and **C**, suggesting that green plant and glaucophyte sequences are unlikely of the same origin (mitochondrial or plastidic origin) with other eukaryotic sequences. P-value = 0.801 from AU test for topology **B**, confirming the likely plastidic origin of the *Cyanidioschyzon* sequence.

4. ThiC family protein (D)

```
CLUSTAL X (1.83.1) multiple sequence alignment


15922177_Sulfolobus_tokoda          MAIIDDARKGIITDEMKKISLIEKISPEKVRKRIVEGKIMLIRNEKYSKK
18311872_Pyrobaculum_aeroph         NTIIRRAREGRIDDEMRKIAEAEGVSPEKLRDRIAKGQVVYIRNVKWSEK
15789892_Halobacterium_sp.          STQLQHARDGTVTDAMRRVADREGRDPEVVREAVADGHAVIPANHHHALD
34541691_Porphyromonas_gingiv       ATQLYYARQGIVTPEMEFVAIRENITPEFVRDEIAAGRAILPANINHESE
48764428_Rhodospirillum_rubrum      FTQLEYARAGVITDEMIYVATRENVTPEFVRAEIAAGRAIIPANINHELE
71836834_Pelobacter_propio          VTQMHYARQGIVTPEMEFVAIRENITPEYVRSEVARGRAIIPLNINHEAE
67938921_Chlorobium_phaeob          VTQMHYARKGIITPEMEYVAIRENVTPEFVRDEIAKGRAIIPANINHELE
54027290_Nocardia_farcin            RTQLAWARAGIVTPEMRFIAAREGVEPELVRAEVAAGRAVIPANHNHELE
45658515_Leptospira_interr          HSQMYFAKKGIITEEMRYAAFRENMEPEFVRSEIACGRAILPSNRNHELE
66797754_Deinococcus_geothe         ITQMQAARRGEITPEMEFVALRENITPEFVRAEVARGRAVIPANVNHELE
73539911_Ralstonia_eutrop           VSQMHYARKGIITPEMEYVALRESITPEFVRNEIATGRAIIPANINHELE
149920854_Plesiocystis_pacific      WSQMHFARRGIITEEMRYCAIRENVEPEFVRDEIAAGRAIIPANIHHELE
21232748_Xanthomonas_campes         VTQMHYARRGIITPEMEFVAIRENITPEFVREEIARGRAILPNNINHESE
15141415_Sinorhizobium_melilo       VTQLAYARAGIVTPEMEFIAIRENVTPEFVRQEVAAGRAIIPANINHEAE
17936444_Agrobacterium_tumefa       VTQLAYARAGIITPEMEFIAIRENVTAEFVRQEVASGRAIIPANINHELE
CM937_Cyanidioschyzon_merolae       FTQLYYARKGIITEEMLFAAVRENVDPEFVRQEVAAGRAIIPSNKKAELE
26991600_Pseudomonas_putida         VSQMHYARQGIITAEMEYVAIRENITPEFVRQEIARGRAIIPANINHEVE
26250765_Escherichia_coli           VTQLHYARQGIITPEMEFIAIRENITAEFVRDEVAAGRAIIPANINHESE
48855688_Cytophaga_hutchi           VSQMHYARKGIITPEMEYIAIRENITPEFVREEVARGRAIIPSNINHESE
16077944_Bacillus_subtil            VTQMHYAKKGIITPEMEFIAIREHVSPEFVRDEVASGRAIIPSNINHESE
61100363_Erythrobacter_litora       VSQMHYARKGIITPEMEYVAERENITPEFVRDEVARGRAIIPNNINHETE
157028_Chlamydomonas                VTQMALARAGIISEEMRFAAEREGLDPEFVRSELARGRAIIPANKCHELE
22136156_Arabidopsis_thalia         YTQMYYAKQGIITEEMLYCATREKLDPEFVRSEVARGRAIIPSNKKHELE
CPO00022869_Cyanophora_paradox      --------------------------------------------------
32445237_Rhodopirellula_baltic      MTQLLSARAGEITPEMEYVAKREDLPVELIRDEVASGRMVIPANKVHALE
67930575_Solibacter_usitat          RTQMHYARKGIITEEMHYIAKRESISPELIRSEVARGRMIIPANVNHNLE
15606443_Aquifex_aeolic             KTQMHLARQGIITEEMEYVAKREGLHPEFVRQEVARGRMIIPANINHHLE
33241220_Prochlorococcus_marin      VSQMYFARREVITEEMSFVAKKENLPESLIMEEVARGRMIIPANINHNLE
16331858_Synechocystis_sp.          VSQMHYARKGVITEEMDYVAKRENLPVELIKDEVARGRMIIPANINHNLE
71675053_Trichodesmium_erythr       VSQMHYGRQGIITEEMNFVAQRENLPAELIRAEVARGRMIIPANINHNLE
45507375_Anabaena_variab            VTQMHYARQGVITEEMQYVAQRENLPADLIREEVARGRMIIPANINHNLE
4981317_Thermotoga_mariti           MTQMEMARKGVVSDEMKKVAEYEGVDVEIVRQKLAEGRAVLPKNKNLHIER
46198627_Thermus_thermo             MTQLEAARKGKITEEMAYVAEKEGVSPEFVREGVAAGRIVIPRNPNHLTD
71839731_Pelobacter_propio          LTQIESARQGILTPQMEAVAAAEELTPAYILQMVAEGKIVIPWNHNRKPK
20093117_Methanosarcina_acetiv      MTIVEDAKKGIITEEMKIVAKDEGLDPEFIRRGIAAGRIVIPTSPYRQVK
20090641_Methanosarcina_acetiv      MTLMEDAKKGIVTPSIEAVAKAEGIDAETVRSCVAKGLIAIPKNNRRETL
57158692_Thermococcus_kodaka        MTQLEEARKGVVTEEMKFIAEREGIDAEKLRRNVAKGYTVIFRNVRHWVK
18977903_Pyrococcus_furios          MTQMEEAKRGVITEEMKKIAKIEKIDPEKLRRSVAKGHTVIFRNVNHWVV
71838180_Pelobacter_propio          MTQLEYARRGVITEKMKIAAQSEGVAPEFIRDGIAAGTIVICHNIKHNGT
19712998_Fusobacterium_nuclea       KTQMEAAKKGILTKEMKSIAESESIDEKVLMERVASGEITIPANKKHSLL
67875148_Clostridium_thermo         TTQMDAAKKGIITDEMRIVAEKEGVHVEKLRELVASGKVVIPANKNHKLE


15922177_Sulfolobus_tokoda          IVAIGKGLTTKVNINIGTSSEVVNLDMELEKVKIANKWG-DTLMDLSTGG
18311872_Pyrobaculum_aeroph         VVAIGKGLSTKINVNLGTSTEVVDLDSELKKVEVANKWG-DTLMDLSVGG
15789892_Halobacterium_sp.          PMIIGRDFATKVNANIGNSDTTGDIDDELEKLHTAVHYGADTVMDLSTGE
34541691_Porphyromonas_gingiv       PMIIGRNFLVKINANIGNSPISSTIEEEVEKAVWAIRWGADTVMDLSTGD
48764428_Rhodospirillum_rubrum      PTIIGRNFLVKVNANIGNSALGSSIEDEVAKLVWAIRWGADTVMDLSTGK
71836834_Pelobacter_propio          PMIIGRNFLVKVNANIGNSALASSVMDEVEKMIWAIRWGADTVMDLSTGA
67938921_Chlorobium_phaeob          PMAIGRNFRVKINANIGNSALASSISEEVEKSVWACRWGADTVMDLSTGK
54027290_Nocardia_farcin            PTIIGKKFLVKINANIGNSAVSSSIAEEVEKMVWATRWGADTIMDLSTGK
45658515_Leptospira_interr          PMVIGKNFLVKINANIGNSTFSSSIEEEVEKLHWAIKWGADTVMDLSTGK
66797754_Deinococcus_geothe         PTIIGRNFRVKVNANLGTSIVTSSIEEEVEKMVWATRWGADTVMDLSTGR
73539911_Ralstonia_eutrop           PMAIGRNFRVKINGNLGNSAVTSSLAEEVEKMVWSIRWGADTIMDLSTGK
149920854_Plesiocystis_pacific      PMIIGRNFLVKINANIGNSAVSSSIEEEVEKLQWSTRWGADTVMDLSTGK
21232748_Xanthomonas_campes         PMIIGRNFLTKINANIGNSAVSSGIAEEVEKLVWSIRWGGDTVMDLSTGK
15141415_Sinorhizobium_melilo       PMIIGRNFLVKINANIGNSAVTSSMAEEVEKMVWATRWGADTVMDLSTGR
17936444_Agrobacterium_tumefa       PMIIGRNFLVKINANIGNSAVTSSMAEEVEKMVWAIRWGADTVMDLSTGR
CM937_Cyanidioschyzon_merolae       PMVVGPNFSVKVNANFGNSAVASNIEDEVEKLQWSVLWGADTVMDLSTGE
26991600_Pseudomonas_putida         PMIIGRNFLVKINGNIGNSALGSSIEEEVAKLTWGIRWGSDTVMDLSTGK
26250765_Escherichia_coli           PMIIGRNFLVKVNANIGNSAVTSSIEEEVEKLVWSTRWGADTVMDLSTGR
48855688_Cytophaga_hutchi           PMIIGRNFLVKINTNIGNSAVTSSIEEEVEKAVWSCRWGGDTLMDLSTGK
16077944_Bacillus_subtil            PMIIGRNFHVKINANIGNSAVTSSIEEEVEKMTWAIRWGADTMMDLSTGK
61100363_Erythrobacter_litora       PMAIGRNFLVKINANIGNSAVASDVAAEVDKMVWSIRWGADTVMDLSTGR
```

```
157028_Chlamydomonas                PCVIGRNFMTKVNSNFGNSAVTSSIEEEVEKLQWSTIWGADTVMDLSTGH
22136156_Arabidopsis_thalia         PMIVGRKFLVKVNANIGNSAVASSIEEEVYKVQWATMWGADTIMDLSTGR
CPO00022869_Cyanophora_paradox      --------------------------------------------------
32445237_Rhodopirellula_baltic      PMAIGIAAKCKINANIGNSAVTSNVGEELEKLHTAVHFGADTVMDLSTGK
67930575_Solibacter_usitat          PMCIGVASKCKINSNIGNSSVVSDIAGELEKLEYSVKYGADTVMDLSTGG
15606443_Aquifex_aeolic             PMCIGINSKVKVNANIGNSGLASDIPTEVEKAKVAIKYGADTIMDLSTGE
33241220_Prochlorococcus_marin      PMAIGIASSCKVNANIGASPNASDVNEELKKLHLAVKYGADTVMDLSTGG
16331858_Synechocystis_sp.          PMAIGIASKCKVNANIGASPNSSNIDEEVEKLLLSVKYGADTVMDLSTGG
71675053_Trichodesmium_erythr       PMCIGIASRCKVNANIGASPNTSDITKEVDKLKLSIKYGADTVMDLSTGG
45507375_Anabaena_variab            PMAIGIASKCKVNANIGASPNSSNLQEEVDKLNLAVKYGADTVMDLSTGG
4981317_Thermotoga_mariti           PMIVGEGFSVKVNANIGTSQGFSSLEEEKEKARVAIEYGADSLMVLSTWG
46198627_Thermus_thermo             FKGIGEGLSVKVNANLGTSYDYVNVEEEVEKARVAIQYGADTVMDLSTGG
71839731_Pelobacter_propio          VCGIGKGLRTKVNASIGTSSDIVDYAAEVRKAKVAQEAGADTLMELSVGG
20093117_Methanosarcina_acetiv      ICGLGEGLRTKVNASIGVSSDIVDVNMEVQKAIAAEKAGADTLMELGTGG
20090641_Methanosarcina_acetiv      PIGIGKYMSTKINANVGTSRDCIDIDAEVEKAKAAEAFGAHAVMDLSTGG
57158692_Thermococcus_kodaka        PVAVGAGVRVKVNANIGTSRDIVDVKAEIEKAKVAVKYGADTIMDLSTGG
18977903_Pyrococcus_furios          PVAVGQGVRVKVNANIGTSRDIVNVEEEIEKAKIAVKYGADTIMDLSTGG
71838180_Pelobacter_propio          PLPVGTGLRTKINANIGSSSDDTDMQKELEKARVAVKYGADAIMDLSTGG
19712998_Fusobacterium_nuclea       AKGVGTGLSTKINVNLGISKDCPNVDKELEKVKVAIDMKVDAIMDLSSFG
67875148_Clostridium_thermo         PQGIGEGLRTKINVNIGISKDCCNFEMELEKAKKAIELKAEAIMDLSSYG


15922177_Sulfolobus_tokoda          DLDLIRREIIKHSELPVGTVPVYQVFIEHKSGGAYFTEDDLLNTIEKHLK
18311872_Pyrobaculum_aeroph         DLDAIRRAVISKSKLPVGTVPVYQAFIEKRSGGAYFTIDDLFNTIERQLK
15789892_Halobacterium_sp.          NLDGIRTANIDNSPVPVGTVPIYEAVTRVE-DVTDITPDLLIDVVEKQAK
34541691_Porphyromonas_gingiv       HIHETREWIIRNSPVPIGTVPLYQTLEKVQGDVTKLNWEIFRDTLIEQAE
48764428_Rhodospirillum_rubrum      AIHATREWILRNSPVPIGTVPLYQALEKVGGDATRLDWAVFEDTLIEQCE
71836834_Pelobacter_propio          HIHETREWILRNSPVPIGTVPIYQALEKVAGVAEDLSWDVFRDTLVEQAE
67938921_Chlorobium_phaeob          NIHQTREWILRNSPVPIGTVPIYQALEKVGGKAEELNWNIYRDTLIEQAE
54027290_Nocardia_farcin            NIHETREWILRNSPVPVGTVPIYQALEKVNGDPAALTWEIYRDTVIEQAE
45658515_Leptospira_interr          NIHETREWILRNSPVPIGTVPIYQALEKVKGKTENLNIQIFLETLEEQAE
66797754_Deinococcus_geothe         HIHQTREWILRNSPVPVGTVPIYQALEKVGGVAEELTWEVYRDTLIEQAE
73539911_Ralstonia_eutrop           HIHETREWILRNSPVPIGTVPIYQALDKTGGIAEDLTWEMFRDTLIEQAE
149920854_Plesiocystis_pacific      NIHETREWIMRNAPVPIGTVPIYQALEKVKGKAEDLNIDIFMETLEEQAQ
21232748_Xanthomonas_campes         HIHETRDWIIRNSPVPIGTVPIYQALEKVDGRAEELTWEIFRDTLIEQAE
15141415_Sinorhizobium_melilo       NIHNIREWIIRNSPVPIGTVPLYQALEKVNGIAEDLSWEVFRDTLIEQAE
17936444_Agrobacterium_tumefa       NIHNIREWIIRNSPVPIGTVPLYQALEKVNGIAEDLNWEVFRDTLIEQAE
CM937_Cyanidioschyzon_merolae       HIPLVRNWILRNSPVPVGTVPIYEALDKVGGVVEDLNWEVFRDTVIEQAE
26991600_Pseudomonas_putida         HIHETREWIIRNSPVPIGTVPIYQALEKVNGVAEDLTWELFRDTLIEQAE
26250765_Escherichia_coli           YIHETREWILRNSPVPIGTVPIYQALEKVNGIAEDLTWEVFRDTLLEQAE
48855688_Cytophaga_hutchi           NIHETREWIIRNCPVPVGTVPIYQALEKVNGKAEDLTWEIFRDTLIEQAE
16077944_Bacillus_subtil            DIHTTREWIIRNCPVPVGTVPIYQALEKVNGVAEDLTWEIYRDTLIEQAE
61100363_Erythrobacter_litora       NIHDTREWIIRNSPVPIGTVPIYQALEKVGGIAEDLTWDIFRDTLIEQAE
157028_Chlamydomonas                NIFETREWVMRNSPVPVGTVPIYEALERADGQVEGITWELFRQVLLDQAE
22136156_Arabidopsis_thalia         HIHETREWILRNSAVPVGTVPIYQALEKVDGIAENLNWEVFRETLIEQAE
CPO00022869_Cyanophora_paradox      -----------NSPVPVGTVPIYQALEKVKGIAEDLTWEIFRETLIEQAE
32445237_Rhodopirellula_baltic      DIDNIRRQIIDKSPVPIGTVPIYQMLEELGGNIEDMNAQHFLDMVEHQAK
67930575_Solibacter_usitat          DIPAIRKAIIGASPIPIGTVPIYEALSRVR-RVEDLSAQVMLEVIEEQAE
15606443_Aquifex_aeolic             AIKETREAIINVSTVPVGTVPIYEAWKIAKGNVKELTVDLILDVIEEQAR
33241220_Prochlorococcus_marin      NLDEVRTAIIQASPVPIGTVPVYQALESVHGSIEKLSEEDFLHIIEKHCQ
16331858_Synechocystis_sp.          DLDVIRTAIINASPVPIGTVPIYQALESVHGSIENLTPDDFLHIIEKHAQ
71675053_Trichodesmium_erythr       NLDEIRTAIINASPVPIGTVPVYQAVESVHGRIENLTADDFLHVIEKHAQ
45507375_Anabaena_variab            NLDEIRTAIINASPVPIGTVPVYQALESVHGTIENLTPEDFLHIIEKHAQ
4981317_Thermotoga_mariti           DLREIRRAIVEMSPVPVGSVPIYDSAVRMKKNVVDFSEKDFFDMVIAHAE
46198627_Thermus_thermo             DLKAIRKRILEVATVPLGTVPIYEAEFRRRKNFFDMSADELFQVIEEHGK
71839731_Pelobacter_propio          DLDRVRREVIAAVDLPVGNVPLYQAFCEKYGNPNKLDEEMLFDLIEQQCA
20093117_Methanosarcina_acetiv      DFLGIRKKVIDSISLSVGSVPLYQAFIEKYGSIVHMTEDELFNATEAQAK
20090641_Methanosarcina_acetiv      DLNEIRTRILKAVNIPVGTVPIYQAAA-SQKVVVEMTSDDMFNAVRKHAE
57158692_Thermococcus_kodaka        DLDEIRKTIMHAVDVPVGTVPIYQAAEEKGKAIIEMSEEDMWRAVEKHFK
18977903_Pyrococcus_furios          DLDFIRRKIMKAVNVPVGTVPIYQAAEERGKAIIEMTEDDMWKAVEKHFK
71838180_Pelobacter_propio          PVDEIRRAVVAETNACIGSVPLYQAALDKKKAIVDMTVDDIFAGIIKHAE
19712998_Fusobacterium_nuclea       KTEEFRKKLITMSTAMVGTVPVYDAIGFYDKELKDIKAEEFLDVVRKHAE
67875148_Clostridium_thermo         KTREFRRKLVEMSPVMIGTVPVYDAVGFYEKDLKDISAEEFFEVVEKHAE
                                                    :*.**:*:           :           :

15922177_Sulfolobus_tokoda          DGVAFMTIHAGLTKDLAIRALKRIIPIVSRGGDMIAGWMIHNNSENPYRK
18311872_Pyrobaculum_aeroph         DGVAFMTIHAAVTKEAAIRVLKRVIPVVSRGGDMIIGWMLHNDAENPYLT
15789892_Halobacterium_sp.          QGVDYMTLHAGVLAEHLPLTDDRTTGIVSRGGSILSQWMTEHGEQNPLYT
34541691_Porphyromonas_gingiv       QGVDYFTIHAGLRWHHVPLTLRRLTGIVSRGGSIIANWCTTHKRESFIYE
48764428_Rhodospirillum_rubrum      QGVDYFTIHAGVRLAHIPLTASRTTGIVSRGGSILAKWCLSHHRENFLYE
71836834_Pelobacter_propio          QGVDYFTIHAGVRLEHLPLTSRRLTGIVSRGGSIMAKWCHAHRCESFLYT
67938921_Chlorobium_phaeob          QGVDYFTIHSGILLDFLPAAQRRTTGIVSRGGSIIAKWCRAHKQENFLYS
54027290_Nocardia_farcin            QGVDYMTVHAGVLLRYVPLTAKRVTGIVSRGGSIMAAWCLAHHRESFLYT
```

```
45658515_Leptospira_interr                 QGVDYFTIHAGVLLRYIPLTTNRITGIVSRGGSILAKWCQAHHKENFLYT
66797754_Deinococcus_geothe                QGVDYFTVHAGVRLAHIPLTARRRTGIVSRGGSILAKWCLAHHRENFLYT
73539911_Ralstonia_eutrop                  QGVDYFTIHAGVLLRYVPLTADRVTGIVSRGGSIMAKWCLAHHKENFLYT
149920854_Plesiocystis_pacific             QGVDYFTIHAGVLLRYVPLTAKRVTGIVSRGGSIMAKWCLAHHKENFLYT
21232748_Xanthomonas_campes                QGVDYFTIHAGVLLRYVPLTARRVTGIVSRGGSILAKWCLAHHKENFLYT
15141415_Sinorhizobium_melilo              QGVDYFTIHAGVRLHYIPLTVDRVTGIVSRGGSIMAKWCLHHHRESFLYE
17936444_Agrobacterium_tumefa              QGVDYFTIHAGVRLHYIPLTVNRVTGIVSRGGSIMAKWCLHHHKESFLYE
CM937_Cyanidioschyzon_merolae              AGVSYFTIHAGVRLAYIPLTARRLTGIVSRGGSIHAKWCIAHRKENFAYE
26991600_Pseudomonas_putida                QGVDYFTIHAGVLLRYVPLTAKRVTGIVSRGGSIMAKWCLAHHKENFLYT
26250765_Escherichia_coli                  QGVDYFTIHAGVLLRYVPMTAKRLTGIVSRGGSIMAKWCLSHHQENFLYQ
48855688_Cytophaga_hutchi                  QGVDYFTIHAGVLLRYIPSTAKRVTGIVSRGGSIMAKWCLSHHKENFLYT
16077944_Bacillus_subtil                   QGVDYFTIHAGVLLRYVPLTAKRTTGIVSRGGAIMAQWCLAHHQESFLYT
61100363_Erythrobacter_litora              QGVDYFTIHAGVRLPYVPMTAKRVTGIVSRGGSIMAKWCLAHHKESFLYE
157028_Chlamydomonas                       QGVDYWTIHAGVLLRHVPLTANRITGIVSRGGSIHAKLCLMEHKENFAYE
22136156_Arabidopsis_thalia                QGVDYFTIHAGVLLRYIPLTAKRLTGIVSRGGSIHAKWCLAYHKENFAYE
CPO00022869_Cyanophora_paradox             QGVDYFTIHAGVLLRFIPMTAKRLTGIVSRGGSIHAKWCLAHHKENFAYE
32445237_Rhodopirellula_baltic             QGVDYMTIHCGVKLEHLHLTVNRVTGIVSRGGSLIAKWMMAHNKQNPLLE
67930575_Solibacter_usitat                 QGVDYMTIHAGILVQHIPLTTKRVTGIVSRGGSILAEWMVKNHKQNFLYE
15606443_Aquifex_aeolic                    QGVSYMTIHAGILREHLPLVQHRVMGIVSRGGAILAQWMAEHGKQNPLYE
33241220_Prochlorococcus_marin             QGVDYQTIHAGLLIEHLPKVKGRLTGIVSRGGGILAQWMLYHHKQNPLFT
16331858_Synechocystis_sp.                 QGVDYMTIHAGLLIEYLPLVKSRITGIVSRGGGIIAKWMLHHHKQNPLYT
71675053_Trichodesmium_erythr              QGVDYQTIHAGILIEHLPLVRSRITGIVSRGGGIIAKWMLHHHKQNPLYT
45507375_Anabaena_variab                   QGVDYQTIHAGILIEHLPLVRSRITGIVSRGGGILARWMLHHHKQNPLYT
4981317_Thermotoga_mariti                  DGIDFMTIHVGVTRRVLDRIKSRVLKIVSRGGAIIAGWMIKNNKENPFYE
46198627_Thermus_thermo                    EGVDYITVHVGVTLKNLEVYRNRTTGIVSRGGGLMAAWMLHRGEENPLYA
71839731_Pelobacter_propio                 DGMAFMAVHCGINLYTIERLRKRYGGLVSKGGVSMVAWMVANNRENPLYE
20093117_Methanosarcina_acetiv             LGTNFMAIHTGINNITLDRLKARYGGLCSRGGAFMSSWMLHNEKENPLYA
20090641_Methanosarcina_acetiv             QGVDFVTVHAGVNLNSLERLRQRIMNVVSRGGSFTLAWMLHNGEDNPFYA
57158692_Thermococcus_kodaka               DGVDYTTIHVGVTKEVVEKMKRRAVGMVSRGGTFLAAWILHWGEENPFYK
18977903_Pyrococcus_furios                 DGVDFVTIHAGVTKEVVEKLKRRVVGMVSRGGTFLAAWILHWGEENPFYK
71838180_Pelobacter_propio                 DGVDFITVHCGVTRSTVERMKNRIMDVVSRGGAFTIEWMAYNNQDNPLFE
19712998_Fusobacterium_nuclea              DGVDFVTIHAGLNREAVNLFKRRITNIVSRGGSLMYAWMELNNAENPFYE
67875148_Clostridium_thermo                DGVDFMTIHAGINRETAKRFKERLTNIVSRGGSLIFAWMELTGNENPFYE
                                            *   : ::* .:             *    : *:**               :.

15922177_Sulfolobus_tokoda                 NWDYILEMFKQYDAVISLGDALRPGATGDAHDEFQIGELLETARLVKSAL
18311872_Pyrobaculum_aeroph                HWDYLLELFAQYDAVISIGDALRPGAVADAHDEFHVGELVEAARLAKRAI
15789892_Halobacterium_sp.                 HYDELCEILQAHDVTISLGDGLRPGSVADASDDAQFAELDTLGELTRRAW
34541691_Porphyromonas_gingiv              HFEEICQILARYDVAISLGDGLRPGCIHDANDAAQIAELKTLGELTEIAW
48764428_Rhodospirillum_rubrum             RFADICAILRRYDVAFSLGDGLRPGSVADANDAAQFAELDTLGALTAVAW
71836834_Pelobacter_propio                 RFEEICEIMKAYDVSFSLGDGMRPGSLHDANDEAQFAELKTLGELTRLAW
67938921_Chlorobium_phaeob                 HFDDICDILRSYDIAISIGDALRPGSIADANDEAQFSELKTLGELTLKAW
54027290_Nocardia_farcin                   HFEELCEILARYDVTFSLGDGLRPGSVADANDEAQFAELRTLGELTKIAK
45658515_Leptospira_interr                 HFDEILKVMKKYGVSISLGDGLRPGSIADANDKAQFSELETLGELTQLAW
66797754_Deinococcus_geothe                HFREICEILSAYDITFSLGDGLRPGSIEDANDAAQFAELDTLGELTRVAW
73539911_Ralstonia_eutrop                  HFDEICEIMKAYDVSFSLGDGLRPGCIADSNDDAQFGELRTLGELTAKAW
149920854_Plesiocystis_pacific             EFERICALMKKYDVSFSLGDGLRPGCIADANDEAQFAELHTLGELTKIAW
21232748_Xanthomonas_campes                HFEDICEIMKAYDVAFSLGDGLRPGCIADANDAAQFGELETLGELTKLAW
15141415_Sinorhizobium_melilo              HFEDICDICRAYDVSFSLGDGLRPGSIADANDRAQFAELETLGELTKIAW
17936444_Agrobacterium_tumefa              HFEEICDICRAYDVSFSLGDGLRPGSIADANDEAQFAELETLGELTQIAW
CM937_Cyanidioschyzon_merolae              HWDELCEIMRQYDVAFSIGDGLRPGCIHDANDEAQFAELKTQGELTRRAW
26991600_Pseudomonas_putida                HFDEICEIMKAYDVSFSLGDGLRPGSIADANDAAQFGELETLGELTKIAW
26250765_Escherichia_coli                  HFREICEICAAYDVSLSLGDGLRPGSIQDANDEAQFAELHTLGELTKIAW
48855688_Cytophaga_hutchi                  HFEEICEIMKAYDVAFSLGDGLRPGCLADANDEAQFSELKTLGELTEIAW
16077944_Bacillus_subtil                   HFEEICEIMKMYDIAFSLGDGLRPGSIADANDEAQFAELETLGELTQIAW
61100363_Erythrobacter_litora              RFDEITEIMKAYDIAYSLGDGLRPGSIADANDEAQFAELYTLGELTKRAW
157028_Chlamydomonas                       HWDEILDICAKYDITLSIGDGLRPGCIADANDAAQFAELKTQGELTRRAW
22136156_Arabidopsis_thalia                HWDDILDICNQYDVALSIGDGLRPGSIYDANDTAQFAELLTQGELTRRAW
CPO00022869_Cyanophora_paradox             HWDDILDICNQYDISLSIGDGLRPGSIHDANDEAQFAELKLQGELTKRAW
32445237_Rhodopirellula_baltic             AFDDLCDIMREYDVTWSLGDGLRPGSIADASDDAQFAELDVLGECTKRGW
67930575_Solibacter_usitat                 CFEDICKILQKHDVSFSLGDGLRPGSIADASDAAQFAELKTLGELTQVAW
15606443_Aquifex_aeolic                    HFDKICEIFKKYDVSFSLGDALRPGCIEDATDDAQLAELKVLGELVEKAW
33241220_Prochlorococcus_marin             RFDDICEIFKRYDCSFSLGDSLRPGCLHDASDEAQLAELKTLGELTQRAW
16331858_Synechocystis_sp.                 HFDEIIEIFKKYDVSFSLGDSLRPGCTHDASDDAQLSELKTLGQLTRRAW
71675053_Trichodesmium_erythr              HFDDIIEIFKKYDVSFSLGDSLRPGCTHDASDEAQLAELKTLGQLTRRAW
45507375_Anabaena_variab                   HFRDIIEIFKKRYDVSFSLGDSLRPGCTHDASDDAQLAELKTLGQLTRKAW
4981317_Thermotoga_mariti                  HFDELLDIAKDYDITLSLGDGMRPGAVVDASDAQQFEELFVMGELVERAR
46198627_Thermus_thermo                    RFDDLLDIARTYDMTLSLGDGLRPGSLADSTDRAQIAELLTIGELVERAR
71839731_Pelobacter_propio                 KFDRVCSILKKYDTVLSLGNGLRAGAIHDSSDRAQIQELLINCELAELGR
20093117_Methanosarcina_acetiv             NFDYLVEILKEHEVVLSTGNGMRAGAVHDATDRAQIQELIINSEVAEKAH
20090641_Methanosarcina_acetiv             EFDYLLEIAKEYDMTLSLGDGMRPGCIADASDRPKFMEFITLGELVKRSR
57158692_Thermococcus_kodaka               DYDYLLELAREYDVVLSLGDGLRPGGLPDAGDELQIAELYTLGRLVRRAR
18977903_Pyrococcus_furios                 NYEYLLELAKEYDVVLSLGDGLRPGGLPDAGDELQIAELYTIGRLVKRAR
```

```
71838180_Pelobacter_propio        HFDKLLEITKEYDMTLSLGDGFRPGCLADATDRAQIHELILLGELTQRAQ
19712998_Fusobacterium_nuclea     NFDKLLDICEEYDMTISLGDALRPGCLNDATDACQIKELITLGELTKRAW
67875148_Clostridium_thermo       QYDRLLQIFEKYDVTISLGDALRPGSINDSTDASQIQELIVLGELTKRAW
                                   :  :  :   :    * *:.:*.*   *: *  :. *:       .  .

15922177_Sulfolobus_tokoda        EKGVQVMVEGPGHVPLNEIAWDVKLMKKLTGGVPYYVLGPLPIDVGAPYD
18311872_Pyrobaculum_aeroph       KAGVQVMIEGPGHVPLNDVIWTIKLEKRLTGGVPYYVLGPLPTDVAAPYD
15789892_Halobacterium_sp.        DHGVQAMVEGPGHVPMDQIRANVDRQQEVCDGAPFYVLGPLVTDIAPGYD
34541691_Porphyromonas_gingiv     KYNVQTIIEGPGHVPMHKIRENMEIQLEACHGAPFYTLGPLVSDVASGYD
48764428_Rhodospirillum_rubrum    EHGCQVMVEGPGHVPMHKIKANMDRQLATCGEAPFYTLGPLTTDIAPGHD
71836834_Pelobacter_propio        KHDVQTMIEGPGHVPLHLIRENMELQLEQCHEAPFYTLGPLVTDVAPGYD
67938921_Chlorobium_phaeob        KYDVQVMIEGPGHVPLNLVEENMRKQLEYCHEAPFYTLGPLVTDIAAGYD
54027290_Nocardia_farcin          SHGVQVMIEGPGHVPMHKIVENVRLEEELCEEAPFYTLGPLATDIAPAYD
45658515_Leptospira_interr        KEDIQVMIEGPGHVPMNLIKENVDLQTKICQEAPFYTLGPIVTDIAPGYD
66797754_Deinococcus_geothe       EHGVQTMIEGPGHVPMQLIRENMTRQLQVCQEAPFYTLGPLTTDIAPGYD
73539911_Ralstonia_eutrop         KHDVQVMIEGPGHVPLQRIQANMDEELKHCYEAPFYTLGPLVTDIAPGYD
149920854_Plesiocystis_pacific    KHDVQVMIEGPGHVPMHKIKENMTEQLEHCHEAPFYTLGPLTTDIAPGYD
21232748_Xanthomonas_campes       KHDVQTMIEGPGHVPMQLIKENMDKQLRECGEAPFYTLGPLTTDIAPGYD
15141415_Sinorhizobium_melilo     AKDCQVMIEGPGHVPMHKIKENMDKQLAVCGEAPFYTLGPLTTDIAPGYD
17936444_Agrobacterium_tumefa     ARDCQVMIEGPGHVPMHKIKENMDKQLKTCGEAPFYTLGPLTTDIAPGYD
CM937_Cyanidioschyzon_merolae     AFDVQVMNEGPGHVPLHKIPENMEKQKKWCSSAPFYTLGPLVIDHAAGRD
26991600_Pseudomonas_putida       KHDVQCMIEGPGHVPMQLIKENMDKQLECCDEAPFYTLGPLTTDIAPGYD
26250765_Escherichia_coli         EYDVQVMIEGPGHVPMQMIRRNMTEELEHCHEAPFYTLGPLTTDIAPGYD
48855688_Cytophaga_hutchi         KHDVQVMVEGPGHVPMHMIKENMDKQLKECHEAPFYTLGPLTTDIAPGYD
16077944_Bacillus_subtil          KHDVQVMIEGPGHVPMHKIKENVDKQMDICKEAPFYTLGPLTTDIAPGYD
61100363_Erythrobacter_litora     EQDVQVMIEGPGHVPMHKIKENMDKQLEACGEAPFYTLGPLVTDIAPGYD
157028_Chlamydomonas              EKNVQVMNEGPGHVPLNKIPENMAKQLEWCSEAPFYTLGPLTTDIAPGYD
22136156_Arabidopsis_thalia       EKDVQVMNEGPGHVPMHKIPENMQKQLEWCNEAPFYTLGPLTTDIAPGYD
CPO00022869_Cyanophora_paradox    EKDVQVMNEGPGHIPLHKVQENVDKQMEWCHEAPFYTLGPLTTDIAPGYD
32445237_Rhodopirellula_baltic    EKGTQVMVEGPGHIPLDQIQMNIERQIEVCHGAPFYVLGPLVTDIAPGYD
67930575_Solibacter_usitat        KHDVQTMIEGPGHIPMDQIEMQVIKEKELCFEAPFYTLGPLVTDIAPGYD
15606443_Aquifex_aeolic           KHDVQAMVEGPGHVPLHQVEFNMKIQQEWCHEAPFYVLGPLVLDVAPGYD
33241220_Prochlorococcus_marin    KHDVQVMVEGPGHVPMDQIEFNVRKQMEDCLEAPFYVLGPLVTDIAPGYD
16331858_Synechocystis_sp.        EHDVQVMVEGPGHVPIDQIEFNVKKQMEECSEAPFYVLGPLVTDIAPGYD
71675053_Trichodesmium_erythr     GHEVQVMVEGPGHVPMDQIEFNVKKQMEECSSAPFYVLGPLVTDIAPGYD
45507375_Anabaena_variab          EHDVQVMVEGPGHVPMDQIEFNVKKQMEECSEAPFYVLGPLVTDIAPGYD
4981317_Thermotoga_mariti         EKGVQVMLEGPGHVPLNEVEMNVRLMKKIGKGAPIFLLGPLPTDRAMGYD
46198627_Thermus_thermo           RAGVQAMVEGPGHIPLNEVAANVQIQKKLTGHAPFYILGMLPVDTAAGFD
71839731_Pelobacter_propio        DMGCQMLVEGPGHVPLDEIEGNIQLQKRMSGDAPYYMLGPIATDVAPGYD
20093117_Methanosarcina_acetiv    QQGLQVIVEGPGHVPLDQIATNVKLMKEMSGHKPFYMLGPLVTDIAPGYD
20090641_Methanosarcina_acetiv    AANVQTFVEGPGHVPLNEIELSVRGMKELCDGAPLYLLGPLVTDIAPGFD
57158692_Thermococcus_kodaka      EAGVQTMVEGPGHVPIDQIAAQVKLAKVATDNAPFYVLGPIVTDIFPGYD
18977903_Pyrococcus_furios        EFGVQTMVEGPGHVPIDQIPTHIRLMKIASDNAPVYVLGPIVTDIFPGYD
71838180_Pelobacter_propio        AAGVQVMIEGPGHVPLQQIQANILLQKRLCHGAPFYVLGPLVTDIAPGYD
19712998_Fusobacterium_nuclea     ERNVQIIIEGPGHMAIDEIEANVKLEKKLCHNAPFYVLGPLVTDIAPGYD
67875148_Clostridium_thermo       EKNVQVMIEGPGHMAINEIAPNMVLEKKLCHGAPFYVLGPIVTDIAPGYD
                                      * : *****:.:. :   :          * : ** :  *     *

15922177_Sulfolobus_tokoda        HIASAIGAAIASASGADLLCYLTPAEHLSLPTVEQVEEGAIAYRIAAHAG
18311872_Pyrobaculum_aeroph       HIASAVGAALAAAAGADLLCYITPAEHLSLPTVEQVEQGAIAYRIAAHIG
15789892_Halobacterium_sp.        HITSAIGATEAARAGAAMLCYVTPKEHLGLPDAEDVRDGMAAYRIAAHAG
34541691_Porphyromonas_gingiv     HITSAIGAAQIGWFGTAMLCYVTQKEHLGLPNREDVREGVVTYRLAAHAA
48764428_Rhodospirillum_rubrum    HITSAIGAAMIGWFGTAMLCYVTPKEHLGLPDRADVKAGVIAYKLAAHAA
71836834_Pelobacter_propio        HITSAIGGAMIGWLGTSMLCYVTRKEHLGLPDKDDVKEGIVTFKIAAHAA
67938921_Chlorobium_phaeob        HVNSAIGGTLLASLGCAMLCYVTPKEHLGLPDKNDVREGVIVHKLAAHAA
54027290_Nocardia_farcin          HITSAIGAAIIAQAGTAMLCYVTPKEHLGLPNRDDVKTGVITYKIAAHAA
45658515_Leptospira_interr        HITSAIGAAMIGWYGTAMLCYVTPKEHLGLPNKEDVKQGVIAYKIAAHAA
66797754_Deinococcus_geothe       HITSAIGAAQIAWYGTAMLCYVTPKEHLGLPDRQDVRDGVIAYRIAAHAA
73539911_Ralstonia_eutrop         HITSGIGAANIGWMGTAMLCYVTPKEHLGLPDKEDVREGIITYKIAAHAA
149920854_Plesiocystis_pacific    HITSAIGAAMIGSFGCAMLCYVTPKEHLGLPNREDVKAGVIAYKIAAHAA
21232748_Xanthomonas_campes       HITSAIGAAMIGWFGTAMLCYVTPKEHLGLPNRQDVRDGIMAYKIAAHAA
15141415_Sinorhizobium_melilo     HITSGIGAAMIGWFGTAMLCYVTPKEHLGLPDRNDVKTGVITYKIAAHAA
17936444_Agrobacterium_tumefa     HITSGIGAAMIGWFGTAMLCYVTPKEHLGLPDRNDVKVGVITYKIAAHAA
CM937_Cyanidioschyzon_merolae     HISSCIGAANIAMHGTALLCYVTPKEHLGLPNADDVKEGLLAYRIAAHAA
26991600_Pseudomonas_putida       HITSGIGAAMIGWFGCAMLCYVTPKEHLGLPNKDDVKTGIITYKIAAHAA
26250765_Escherichia_coli         HFTSGIGAAMIGWFGCAMLCYVTPKEHLGLPNKEDVKQGLITYKIAAHAA
48855688_Cytophaga_hutchi         HITSAIGAAMIGWFGTAMLCYVTPKEHLGLPNKKDVKDGVMAYKIAAHAA
16077944_Bacillus_subtil          HITSAIGAAMIGWYGTAMLCYVTPKEHLGLPNRDDVREGVITYKIAAHAA
61100363_Erythrobacter_litora     HITSGIGAAQIGWYGTAMLCYVTPKEHLGLPDRDDVKVGVVTYKLAAHAA
157028_Chlamydomonas              HITSAIGAATIGALGTALLCYVTPKEHLGLPNRDDVKAGVIAYKIAAHAA
22136156_Arabidopsis_thalia       HITSAIGAANIGALGTALLCYVTPKEHLGLPNRDDVKAGVIAYKIAAHAA
CPO00022869_Cyanophora_paradox    HITSAIGAANIGMGGTALLCYV----------------------------
32445237_Rhodopirellula_baltic    HITSCIGAANAGMHGAAMLCYVTPKEHLGLPNEEDVKQGVIAYKIAAHTA
```

```
67930575_Solibacter_usitat          HITSAIGAAMIGWYGASMLCYVTPKEHLGLPNREDVKAGIIAYKIAAHAA
15606443_Aquifex_aeolic             HIGSAIGGALAGWAGAAMLCYITPKEHLGLPNVEDVKQGVIAYKIAAHAA
33241220_Prochlorococcus_marin      HITSAIGAAMAGWYGTAMLCYVTPKEHLGLPNPEDVREGLIAYKIAAHAA
16331858_Synechocystis_sp.          HITSAIGAAIAGWHGTAMLCYVTPKEHLGLPNAEDVRNGLIAYKIAAHAA
71675053_Trichodesmium_erythr       HITSAIGAAMAGWYGTAMLCYVTPKEHLGLPNAEDVRNGLIAYKIAAHAA
45507375_Anabaena_variab            HITSAIGAAMAGWYGTAMLCYVTPKEHLGLPNAEDVRNGLIAYKIAAHAA
4981317_Thermotoga_mariti           HIACAIGGALAGYYGADFLCYVTPSEHISLPDVEDVREGVIASKIAAIVA
46198627_Thermus_thermo             HIAGAIGGALAGWWGADMLCYLTPAEHLGLPTPEHVKQGVIAFKIAAHAA
71839731_Pelobacter_propio          HITAAIGAAQSSRFGADLICYITPAEHLALPNEEDVRMGVKAAKVAAYIG
20093117_Methanosarcina_acetiv      HIVTAIGASVSASYGCDFLCYVTPAEHLALPNLEDVITGVKTSRIAAHVG
20090641_Methanosarcina_acetiv      HITGAIGGAVAGMYGTDFLCMVTPSEHLALPSLEDIKEGLFVTKLAAHTI
57158692_Thermococcus_kodaka        HITAAIGGAIAALNGADFLCYVTPAEHLGLPDVEHVRQGVIAAKIAAHAV
18977903_Pyrococcus_furios          HISAAIGGAIAALNGADFLCYVTPAEHLGLPTIEHVKEGVIATKIAAHAV
71838180_Pelobacter_propio          HITCAIGGAIAAAAGADFLCYVTPSEHLRLPDVQDVRDGVIASRIAAHAG
19712998_Fusobacterium_nuclea       HITSAIGGAIAAAAGVDFLCYVTPAEHLRLPDLDDMKEGIIASRIAAHAA
67875148_Clostridium_thermo         HITSAIGGAIAAANGADFLCYVTPAEHLRLPDIDDMKEGIIAARIAAHAA
                                    *.    :*.:   .   *   ::* :

15922177_Sulfolobus_tokoda          DIVKLGKKVRNWDDVVSYYRGKLEWEKMISSLIDPERAMKVYTQFGKPKV
18311872_Pyrobaculum_aeroph         DVVKLGRKARRWDDEVSYYRGRLMWDEMIKRLVDPERAYKVYTQYGPPKV
15789892_Halobacterium_sp.          DVAAGKPGARDWDDALSEARYNFDWNRQFDLALDPERAQAFHDQTLPGDN
34541691_Porphyromonas_gingiv       DLAKGHPTAYWRDYMMSKARFEFRWKDQFHLSLDPEKAIQFHDATLPDEG
48764428_Rhodospirillum_rubrum      DIAKGHPAAQLRDDAISRARFDFRWSDQFNLGLDPEGARAFHDETLPHAA
71836834_Pelobacter_propio          DLAKGHPGARLRDDALSKARFEFRWKDQFNLALDPEMPQRLHDLTLPAEA
67938921_Chlorobium_phaeob          DIAKGSPSALLHDKLMSSARYSFAWNDQFNLSLDPVKTRQVHAESSQQNT
54027290_Nocardia_farcin            DLAKGHPHAQQRDDALSKARFEFRWRDQFALSLDPDTAREYHDETMPAEP
45658515_Leptospira_interr          DLAKGHPGAIDRDNLLSKARFEFRWEDQFSLSLDPETAKTFHDEMLPQDR
66797754_Deinococcus_geothe         DLAKGHPGAQARDNALSQARFEFRWEDQFNLALDPERARALHDATLPADA
73539911_Ralstonia_eutrop           DLAKGWPGAQLRDNALSKARFEFRWEDQFNLGLDPERARSYHDATLPAEG
149920854_Plesiocystis_pacific      DLAKGHPGAQVRDDALSKARFEFRWEDQFNLSLDPETAREFHDETLPAEP
21232748_Xanthomonas_campes         DLAKGHPGVQVRDNALSKARFEFRWDDQFHLGLDPEKAKEFHDETLPKDA
15141415_Sinorhizobium_melilo       DLAKGHPAARTRDDALSRARFEFRWEDQFNLSLDPETARNFHDETLPKEA
17936444_Agrobacterium_tumefa       DLAKGHPAAQVRDDALSRARFEFRWEDQFNLSLDPDTARSFHDETLPKEA
CM937_Cyanidioschyzon_merolae       NLAKGLPGAQARDDAISKARFEFRWSDQFNLSLAPLDAKRHHDEALPAEA
26991600_Pseudomonas_putida         DLAKGHPGAQIRDNALSKARFEFRWEDQFNLGLDPDTARAFHDETLPKES
26250765_Escherichia_coli           DLAKGHPGAQIRDNAMSKARFEFRWEDQFNLALDPFTARAYHDETLPQES
48855688_Cytophaga_hutchi           DLAKGHPGAQNRDNALSKARFEFRWADQFNLSLDPDTAREFHDETLPAEG
16077944_Bacillus_subtil            DLAKGHPGAQIRDDALSKARFEFRWRDQFNLSLDPERALEYHDETLPAEG
61100363_Erythrobacter_litora       DLAKGHPAAKVRDDALSKARFEFRWRDQFNLSLDPDTAEQYHDQTLPAEG
157028_Chlamydomonas                DLAKGHPAAASWDLELSKARFEFRWRDQFALSLDPVTAQAYHDATLPQEP
22136156_Arabidopsis_thalia         DLAKQHPHAQAWDDALSKARFEFRWMDQFALSLDPMTAMSFHDETLPADG
CPO00022869_Cyanophora_paradox      --------------------------------------------------
32445237_Rhodopirellula_baltic      DVARKRKGAQDRDDALSKARFAFDWKEQFRLSLDPETAQRYHDETLPQDT
67930575_Solibacter_usitat          DIARHRPGVRDRDDALSRARYRFDWKEQFALSLDPETARSMHDETLPEDG
15606443_Aquifex_aeolic             DIAKHWPGARDWDLAMSIARYNFDWNKQFELAMDPETARAYHDETLPQEG
33241220_Prochlorococcus_marin      DIARHRSGARDRDDELSRARYAFDWNKQFELSLDPERAREYHDETLPADI
16331858_Synechocystis_sp.          DIARHRPGARDRDDELSKARYNFDWNRQFELSLDPERAKEYHDETLPADI
71675053_Trichodesmium_erythr       DIGRHRQGARDRDDELSAARYNFDWNRQFELSLDPERAKEYHDETLPADI
45507375_Anabaena_variab            DIARHRPGARDRDDELSKARYNFDWNRQFELSLDPERAKEYHDETLPADI
4981317_Thermotoga_mariti           DVARGNKKAWELEKKMALARKNFDWETMFSLSLGKDVAKKKYEERPYPDK
46198627_Thermus_thermo             DVARGNKRALERNRRMSEARYRLDWEGQFALALFPEEARRLKEERGSKTK
71839731_Pelobacter_propio          DMNKYPEKGRQRDKEISKARRDLNWDKQYELALFPEDAKAIRDSRTPEDE
20093117_Methanosarcina_acetiv      DMIKYPERARQWDLDMGRARRELDWEKMYSLAIDPEHARAVRNSRAPEDT
20090641_Methanosarcina_acetiv      DLIKEGPRERAWDTAMAYARRDLDWEKQFELAIDGDRARKIRDARKTESD
57158692_Thermococcus_kodaka        NLTRFE-ADFKKDYLMALARGKLNWARQFELSMDKERFVEIRKERPTKTE
18977903_Pyrococcus_furios          NLTRFEEDFKI-DYQMSLARGKLNWKEQFKIAFDKEKFIEIRKERPTKSE
71838180_Pelobacter_propio          DIAKGVKGAMNKDIQMAKCRKKLDWEGQYSLALDPERARTLRESSPVSDH
19712998_Fusobacterium_nuclea       DISKKVPKAIDWDNRMAKYRTDIDWEGMFTEAIDEEKARRYRKESTPENE
67875148_Clostridium_thermo         DIAKGIKGAREWDYQMSEARRNLDWNRMFELAIDREKAERYRKSSMPEDE


15922177_Sulfolobus_tokoda          RTMCGGYCPMMWAMEQ
18311872_Pyrobaculum_aeroph         KTMCGGYCPMNMVIQQ
15789892_Halobacterium_sp.          YSMCGDFCSMRIDQDA
34541691_Porphyromonas_gingiv       HSMCGHFCSMRANKNF
48764428_Rhodospirillum_rubrum      HSMCGKFCSMKISHDI
71836834_Pelobacter_propio          DSMCGDFCAMRITRNI
67938921_Chlorobium_phaeob          GTMCGDFCSMKKSQEV
54027290_Nocardia_farcin            ASMCGKFCSMRISADV
45658515_Leptospira_interr          MSMCGHFCSMNLTQEL
66797754_Deinococcus_geothe         ASMCGHFCSMKLSHDL
73539911_Ralstonia_eutrop           ASMCGKFCSMKITQEV
149920854_Plesiocystis_pacific      ASMCGHFCSMKLTQDV
```

```
21232748_Xanthomonas_campes         HSMCGHFCSMKITQDV
15141415_Sinorhizobium_melilo       HSMCGKFCSMRISHDI
17936444_Agrobacterium_tumefa       HSMCGKFCSMRISHDI
CM937_Cyanidioschyzon_merolae       ASMCSDFCSMRITQDI
26991600_Pseudomonas_putida         ASMCGKFCSMKITQEV
26250765_Escherichia_coli           GSMCGKFCSMKISQEV
48855688_Cytophaga_hutchi           ASMCGHFCSMKITQEV
16077944_Bacillus_subtil            ASMCGKFCSMRISQDI
61100363_Erythrobacter_litora       ASMCGKFCSMKITQEV
157028_Chlamydomonas                ASMCGKFCSMNITQEL
22136156_Arabidopsis_thalia         ASMCGKFCSMKITEDI
CPO00022869_Cyanophora_paradox      ----------------
32445237_Rhodopirellula_baltic      FSMCGKYCSMKITEDI
67930575_Solibacter_usitat          FSMCGKFCSMNISAKV
15606443_Aquifex_aeolic             YSMCGEFCSYKISQKV
33241220_Prochlorococcus_marin      YSMCGKHCPMQTKITD
16331858_Synechocystis_sp.          YSMCGKFCPMQTKVDA
71675053_Trichodesmium_erythr       YSMCGKFCPMQTKVDA
45507375_Anabaena_variab            YSMCGKFCPMQTKVDA
4981317_Thermotoga_mariti           GSMCGPFCAIKIAEEF
46198627_Thermus_thermo             ASMCGPFCPMNLVEAV
71839731_Pelobacter_propio          NTMCGDFCASRGAGRL
20093117_Methanosarcina_acetiv      DTMCGNFCALKIVNQN
20090641_Methanosarcina_acetiv      ASMCGELCAVKIVKEA
57158692_Thermococcus_kodaka        ASMCGDLCAIKLINDM
18977903_Pyrococcus_furios          ASMCGDLCAIKIVREM
71838180_Pelobacter_propio          GTMCGEFCAYKVMDDA
19712998_Fusobacterium_nuclea       DTMCGKMCSMRTMKKM
67875148_Clostridium_thermo         DTMCGRMCAVKNTNKA
```

Note: All top hits in GenBank searches (using *Cyanidioschyzon* sequence and *Arabidopsis* GI 22136156 as queries) are from proteobacteria, firmicutes and spirochaetes. Primary photosynthetic eukaryotic sequences share many conserved residues with non-cyanobacterial sequences, and likely are not of cyanobacterial origin. The same is also supported by the phylogenetic analyses. The *Arabidopsis* sequence (GenBank accession number NP_180524 and TAIR locus AT2G29630) is annotated as a chloroplast precursor. The glaucophyte *Cyanophora* sequence was obtained from TBestDB.

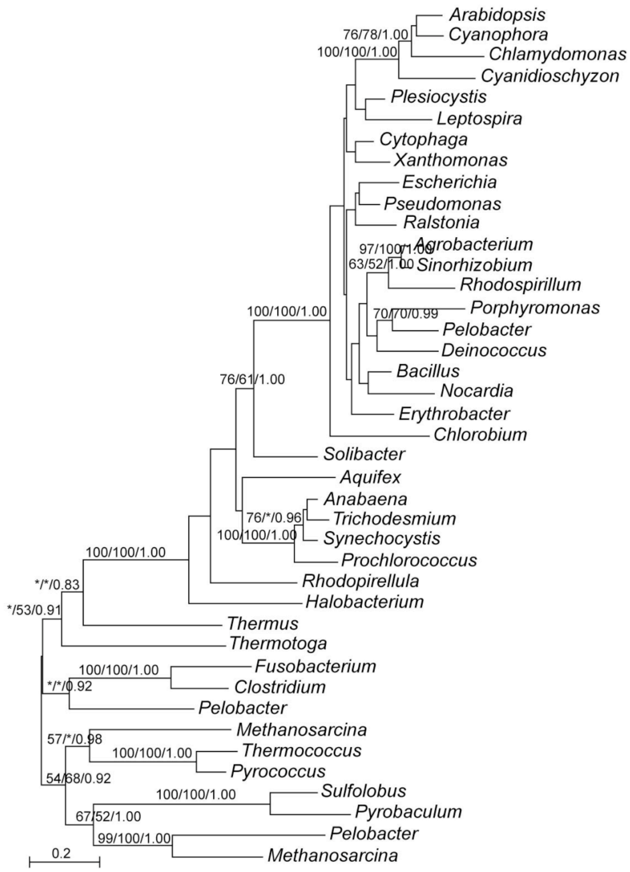

Figure 4. Molecular phylogeny of ThiC. P-value = 0.539 from AU test for the presented tree. AU tests were also performed on alternative topologies, including (**A**) monophyly of red algal, green plant and cyanobacterial sequences, and (**B**) monophyly of red alga, green plant and archaeal sequences. These tests investigate if red algal and green plant sequences has a plastidic or an archaeal (or eukaryotic) origin. P-values < 0.001 from AU tests for both alternative topologies.

---

5. Diaminopimelate decarboxylase (lysA) (D)

```
CLUSTAL X (1.83.1) multiple sequence alignment


66825991_Dictyostelium_discoi       TPFHIYNGDKIKENGELLMNYFAVKATPNPSILKLLKEIGMGVDCSSLAE
24213747_Leptospira_interr          TPVFVYSRERIEKSCEVALVRYAMKANPNRTVLEIMKRKGIQIDASSEYE
116056027_Ostreococcus_tauri]       TPTYVYDAPTLEARAAVAKVRYAMKASPNAAILKIFKKAGLHVDASSGYE
87306324_Blastopirellula_marin      TPTFVYDAAKIVERIEDLRIRYAQKACSNLAILDLMRKNGVMVDAVSAWE
21224744_Streptomyces_coelic        TPLWLYDAATIRAQIDRLRIRYAQKACSNLHILRLMREEGVHVDAVSEGE
75239202_Escherichia_coli           CPVWVYDAQIIRRQIAALKVRFAQKACSNIHILRLMREQGVKVDSVSLGE
118385090_Tetrahymena_thermo        TPLYVVYNSQVIKERCQQLLALYACKANTNIAIIKLMKEQGIGIDAVSIQE
145525334_Paramecium_tetrau         SPLYVVYDEEIIKRQCQTITVLYACKANTNPNVLKIMKSNGVGIDAVSPNE
48477088_Picrophilus_torrid         TPLIVLSEKRINNNYLKIKVHYALKANSNPAVISILRRLGAGADAANPNE
126007985_Ferroplasma_acidar        TPVIVYNMARVRENIRRIRVHFAVKSNYNPYIVSQIIKEGTGIDAANYNE
60682355_Bacteroides_fragil         TPFYYYDTKVLRDTLACVNVHYAVKANANPKVLTIIRESGLGADCVSGGE
123416580_Trichomonas_vagina        --------------------------------------------------
45358763_Methanococcus_maripa       TPLYVMSETQTVKNFTRYVISFAYKANTNLAVTRLLSKLGCGADIVSAGE
```

```
15644265_Thermotoga_mariti         TPTYVYFEETLRKRSRLVKPTFAVKANNNPVLLKILREEGFGMDVVTKGE
CM1350_Cyanidioschyzon_merolae     SPFYLYSQSRIEANVRAYQIGYAVKANNNLMIMQLVRERGCGAVLVSGNE
15231844_Arabidopsis_thalia        RPFYLYSKPQITRNLEAYKIGYAIKANNNLKILEHLRSLGCGAVLVSGNE
88601236_Glaucocystis_nostoc       --------------------------------------------------
8134392_Archaaeoglobus_fulgi       TPVYVTSRALLERNLEAYKLLYAVKANNNLALMRIIASHGFGADVFSDGE
67919461_Chlorobium_limico         TPLFVTSKRSLEGSLREFETCYSVKANFNLSVISTLAGMGCGCDVNSGGE
91202264_Candidatus_Kuenen         TPVYIYSKNAILTRFNELKICFSVKSNSNLSVCKILADEGSGFDVVSGGE
94970467_Acidobacteria_bacter      TPLYTYSATTIRERFDIFNICYSVKANSNLTILKMLVKLGSGFDVVSGGE
15606446_Aquifex_aeolic            TPLYVYSSNFIKERFEAYRICYAVKANFNPHLVKLLGELGAGADIVSGGE
108802931_Rubrobacter_xylano       TPAYVYSYAALERAYREVEVCFAVKANGNLAVLRALASLGAGADIVSGGE
124516709_Leptospirillum_sp.       SPVYIYSEKALREAYSSYQIAYAMKANGNLQILSMLGKMGSGADVVSGGE
94417748_Pseudomonas_aerugi        TPTYVYSRAHIEAQYRAYAVCFAVKANSNLGVLNVLARLGAGFDIVSRGE
94499872_Oceanobacter_sp.          SPLYIYSRKAFSEHYLEYAICYAVKANSNIAVLNVLAKLGAGFDIVSVGE
77920014_Pelobacter_carbin         TPFYVYSHATLSRHLEAFSICYSIKANSNLAVLKTFVSHNAGFDIVSGGE
15075673_Sinorhizobium_melilo      TPFYCYSTATLERHYKVFSVCYAMKANSNQAVLKTLGRLGAGLDVVSEGE
76258104_Chloroflexus_aurant       TPLYLLDDLTLRGAMRAYRVHYAGKALLNTALAQIVAQEGLGLDVVSATE
118062084_Roseiflexus_casten       TPLYLFDEETIRSACRTWRVHYASKALLNTALTHLIADEGLGLDVVSGGE
23128550_Nostoc_puncti             SPLYILDEETLRSACQQYRVLYASKAWNCLAVCAIAASEGLGIDVVSGGE
33240555_Prochlorococcus_marin     TPLYILDELTLRTSCREYKPLYASKANSSLIISNIVASEGMGVDVVSEGE
87303422_Synechococcus_sp.         TPLYVLDEATLRASCRAYQALYASKANSSLVLSALVASEGLGLDAVSAGE
21223714_Streptomyces_coelic       TPAYLLDEADFRERARAWRVFYAGKAFLSRAVVRWLDEEGLNLDVCSGGE
116671170_Arthrobacter_sp.         TPLFVMSENDFRARARAFSVYYAGKSFLCTAVVRWVEEEGLRLDTASGGE
118443019_Clostridium_novyi        TPLYVMDEELIRENCRRYRVAYAGKAFLTIAMCQLINEEGMCLDVVSGGE
51892948_Symbiobacterium_therm     TPLYVMDEELIRQNCRAYVVAYASKAFLCTAMAALVAQEGLSLDVVSGGE
16079395_Bacillus_subtil           TPLYVYDVALIRERAKSFKVAYASKAFSSVAMIQLAEEEGLSLDVVSGGE

66825991_Dictyostelium_discoi      LILSEKVGFKNEEIMFTSNNTKSEEYKKAYELGSINLDDYTQIEYLYEIS
24213747_Leptospira_interr         VLRAIHYGFKPEQIMLTSQELAKG-LKELVEKGVFNACSLRQLETFGNVS
116056027_Ostreococcus_tauri]      CERAMRAGFAGDEISLSTQEL-PSFFPELVEKGVINACSLDQLERFGAIG
87306324_Blastopirellula_marin     IRRALAAGYEPAPLVYTADIFDHEALALCVEHGLVNCGSPDMIAQLGEIT
21224744_Streptomyces_coelic       IERALAAGYDDEPIVFTADLLNRSTLRRVVELGIVNAGSPQMLDQVGRVW
75239202_Escherichia_coli          IERALAAGYHPDDIVFTADVIDQATLERVSELQIVNAGSVDMLDQLGQVW
118385090_Tetrahymena_thermo       VLLAKKAGFEGRDIQFTENFIGQKEVEDSLKEGILCIGELDTLETLGQVA
145525334_Paramecium_tetrau        VEVALQAGFLNNQITFTANYMTKEEIDYAIQKGVLNVGEIDTLKKL--VI
48477088_Picrophilus_torrid        AEIAMYSGISPDNIIMTGNNLSYNDLKRALEMNIINFDDVNQMNILKNIS
126007985_Ferroplasma_acidar       VKLAIMCGLGSRDIIATPNNLSGTELRTIKEEGVINFDHEGQMGLLKGVS
60682355_Bacteroides_fragil        IRAAIKAGFPAGKIVFAGVGKADWEIDLGLDYDIFNVESVPELEVINEVA
123416580_Trichomonas_vagina       --------------------------------------------------
45358763_Methanococcus_maripa      LYIAKLSDVPSEKIVFNGNCKLKEEIKMGIEAEIFNVDSISELVLINEVA
15644265_Thermotoga_mariti         LLAAKLAGVPSHTVVWNGNGKSRDQMEHFLREDVVNVDSFEEMEIWREYF
CM1350_Cyanidioschyzon_merolae     LRLARLAGFDMRRTVFNGNGKTPQEIALAVRAECINVDSEFDLENILAVL
15231844_Arabidopsis_thalia        LRLALRAGFDPTKCIFNGNGKSLEDLVLAAQEGVVNVDSEFDLNNIVEVL
88601236_Glaucocystis_nostoc       --------------------------------------------------
8134392_Archaaeoglobus_fulgi       LYLASLAGFRKDMVLFNGNSKSRKEIEMGVTAGVFSVDSLDELRTISKIA
67919461_Chlorobium_limico         LFRALKAGVSPEKIIFAGVGKKPEEIAFALEAGVLKVESLSELEAIERVA
91202264_Candidatus_Kuenen         LFRALKAGGKPSKIVFAGVGKTDKEIQYALENDIFNVESIAEIEHINAVA
94970467_Acidobacteria_bacter      LERVLVVDKAAKKVVFSGVGKTAPEMDLAIKSDIFNIESESELELLASIA
15606446_Aquifex_aeolic            LYLAKKAGIPPERIVYAGVGKTEKELTDAVDSEIFNVESRQELDVLNEIA
108802931_Rubrobacter_xylano       LYRAMRAGFDPKKVVFAGVGKTEQELKAGLGERIFNVESAGELEHLERVA
124516709_Leptospirillum_sp.       LFRARRAGIPSDRIVFAGVGKTDGEMREAIDAGIFNVESSMELDRLSRVA
94417748_Pseudomonas_aerugi        LERVLAAGGDPAKVVFSGVGKSRDDMRRALQVGVFNVESSEELERLQRVS
94499872_Oceanobacter_sp.          LERVLKAGGDPSKIVFSGVAKTADEMRRALAVGVFNVESESELERLNALS
77920014_Pelobacter_carbin         LFRAQQVGCDPAKIVYSGVGKTEAEIADALQAGIFNVESRQELDTINAIA
15075673_Sinorhizobium_melilo      LRRALAAGIPAGRIMFSGVGKTAREMDFALEAGIFNVESEPELEVLNQVS
76258104_Chloroflexus_aurant       LLVAQRAGLPMDRVHLHGNAKSDQELERAIAWRVIVVDNLDELQRLCAVL
118062084_Roseiflexus_casten       LYVALRAGFPPQRIHMHGNAKTRAELEQALASGIIIVDNLDELAMLANIA
23128550_Nostoc_puncti             LYTALQAGVSPEKIYLHGNNKSREELILAIESGVIVADNWYELRTLVEIM
33240555_Prochlorococcus_marin     LITALKGGVESSNIVFHGNNKSKSELIMAYKNDVIVLDNQYDIDQLRELM
87303422_Synechococcus_sp.         LLTALQGGMPPERIVLHGNNKSDEELSLAVAHGVVVVDNDHDLERLAELM
21223714_Streptomyces_coelic       LATALSAGMPAERIAFHGNNKSPEEIERAVRAGVIVLDSFQEIVRVAHVQ
116671170_Arthrobacter_sp.         LAVAARAGIPGADVALHGNNKSDAEIHRALDMKLIVVDSLAELERIAAVM
118443019_Clostridium_novyi        LYTAYKSEFPMEKIYFHGNNKTLEEIDMGVRLGVFVADNFYEIENINKIL
51892948_Symbiobacterium_therm     IATALQAGFPMDRTYFHGNNKTAEEIRLALEAGVFVVDSLHELELLNRAL
16079395_Bacillus_subtil           LYTAVAAGFPAERIHFHGNNKSREELRMALEHRIIVVDNFYEIALLEDVL

66825991_Dictyostelium_discoi      FRYNPGPLKKGNDIIGN-PKDAKFGLTSVKRFGLHCMVASNELDTFEMVN
24213747_Leptospira_interr         VRFNPGLGSGQTKKTDVGGKTSSFGIWYLKIFKVHTHIGSGS-DECKILN
116056027_Ostreococcus_tauri]      VRFNPGLGSGGTGKTNVGGPSSSFGIWYLKVVRVHTHIGSGS-DDVTTLN
87306324_Blastopirellula_marin     LRINPGFGHGHSQKTNTGGQQSKHGIWHVRVTGLHMHIGSGT-DTITTIS
21224744_Streptomyces_coelic       IRINPGFGHGHSRKTNTGGEHSKHGIWHLDLVGLHMHIGSGV-DDIRAIS
75239202_Escherichia_coli          LRVNPGFGHGHSQKTNTGGENSKHGIWYLQLVGIHMHIGSGV-DDLQAIS
```

```
118385090_Tetrahymena_thermo        VRINPDVGVGECEHTITGGPKSKFGIYCIEIEGIHQHIGSNMVQGLKFIN
145525334_Paramecium_tetrau         IRLNLDVGIGHHNHVNTGGNDSKFGINLVKIIGLHQHMGSKIMKYLKYID
48477088_Picrophilus_torrid         FRINPGYGNGEFTGIKTAGPSSKFGIPVVKKFGIHMMAGSNNLDSFDFID
126007985_Ferroplasma_acidar        FRINPGIGKGEFPGITTGGKNVKFGISVVKHFGIHMMTGSNVLEDFDFID
60682355_Bacteroides_fragil         FRINPNVGAHTHANITTGLAENKFGISMVKFIGLHFHIGSQILDLVEHIN
123416580_Trichomonas_vagina        ----------------------------------------MLDNVKILD
45358763_Methanococcus_maripa       FRVNPNVDAKTHPKISTGMKKNKFGLDIVKIVGIHCHIGSQLTYKIRDVN
15644265_Thermotoga_mariti          IRVNPEVDAKTHPHISTGLKKHKFGIPLMNIRGLHVHIGSQITRGFEEIN
CM1350_Cyanidioschyzon_merolae      LRINPDIDPQVHPYVSTGLATSKFGIRTVELVGVHSHLGSTIKKSIRFLN
15231844_Arabidopsis_thalia         LRINPDVDPQVHPYVATGNKNSKFGIRNLKLVGAHCHLGSTITKEVSYLN
88601236_Glaucocystis_nostoc        --------------------------------------------------
8134392_Archaaeoglobus_fulgi        FRVNPDVDPKTHPKIATGLRESKFGIPHVVPVGIHCHIGSQILDELSFVD
67919461_Chlorobium_limico          LRINPNVTAETHPYITTGDSKEKFGIDEVRLVCLDMHIGSQIFDSIEYLD
91202264_Candidatus_Kuenen          LRINPDIDAKTHAKTTTGKKENKFGIDLVTICGIHVHLGSPIYSNIQYLN
94970467_Acidobacteria_bacter       FRVNPDVPAETHPYISTGLREHKFGVPILEPAGVSVHIGSQITDNIRLID
15606446_Aquifex_aeolic             IRVNPDVDPKTHPYIATGMQKSKFGVDILEIVGIHCHIGSQILDDIKYLD
108802931_Rubrobacter_xylano        LRINPGVDPGTHEHISTGNVESKFGIPAVDLIGVHQHIGSQIVKDIRYLN
124516709_Leptospirillum_sp.        LRVNPDVDPKTHPYISTGMKKSKFGIPVIRIVGIHQHIGSQLTEEVTWLD
94417748_Pseudomonas_aerugi         LRVNPDVDAQTHPYISTGLKENKFGIAILEVIGVDCHIGSQLTQAIRHLD
94499872_Oceanobacter_sp.           LRVNPDVDAQTHPYISTGLKENKFGVDIIEIKGVDCHIGSQLTQELEHID
77920014_Pelobacter_carbin          IRVNPDVDPQTHPYISTGMKKAKFGINILEVIGVDCHIGSQLTKGIKYLD
15075673_Sinorhizobium_melilo       FRINPDVDARTHAKISTGKKENKFGISWIEVTGIDMHIGSQITEVIEHVD
76258104_Chloroflexus_aurant        LRVAPAIEADTHAHIATGSATAKFGLPLLRWLGLHAHIGSQLFAAPVELS
118062084_Roseiflexus_casten        LRIAPDIVTNTHAHIQTGHATSKFGLPLLCLIGLHAHLGSQLFDIIQQIN
23128550_Nostoc_puncti              LRLTPGIECHTHEYIRTGHLDSKFGFDPLDCVGVHAHIGSQIFENITELN
33240555_Prochlorococcus_marin      LRFTPGIECHTHEYIKTGHIDSKFGFDPGELIGLHAHIGSQIFESLNALN
87303422_Synechococcus_sp.          LRFTPGIECHTHEYIRTGHLDSKFGFDPARLTGLHAHIGSQIFEPVSDLN
21223714_Streptomyces_coelic        IRITVGVEAHTHEFIATAHEDQKFGIPLLEVIGIHSHIGSQIFDELPEID
116671170_Arthrobacter_sp.          LRLTPGVHAHTHEFIATAHEDQKFGLSMIELLGLHCHIGSQIFEVLPELD
118443019_Clostridium_novyi         LRITPGIEAHTHDYIKTGQIDSKFGFTLIEFVGFHCHIGSQIFETIKELD
51892948_Symbiobacterium_therm      LRVTPGVEAHTHEYISTGQLDSKFGIPIVELLGFHCHIGSQIFDTPSELS
16079395_Bacillus_subtil            LRITPGVEAHTHDYITTGQEDSKFGFDLIQLLGVHCHIGSQIFDVSKVLN


66825991_Dictyostelium_discoi       LGGGIGIPYRNVVTECGRVITGPYGVLVTKAIHSKKEYIGVDACMVNLMR
24213747_Leptospira_interr          LGGGFKVGRMRLEIEPGSFLMVNNGSILTTVDDVVYTFVKLDAGMDVNTR
116056027_Ostreococcus_tauri]       LGGGYKVGRMKLEIEPGTFLLANSCAVVTRVQDKVHTFLKLDCGMTEVLR
87306324_Blastopirellula_marin      AGGGLPIPYNSLEIEPGRYLSAESGFLLAEIRAIKNKFYVVDAGFNNLAR
21224744_Streptomyces_coelic        AGGGLSVPYTRLEIEPGRFLVAGAGVLAAEVRAQKNYFVLVDAGFNDLMR
75239202_Escherichia_coli           AGGGLSIPYQKLEIEPGRFLVAQAGVLITQVRSVKRHFVLVDAGFNDLMR
118385090_Tetrahymena_thermo        VGGGIGVPYEEIRVEPGRFLVAESGHLLTTVTAIKSTWIGTDTGMNHLIR
145525334_Paramecium_tetrau         IGGGFGVKYQMLFIEPGRYLIAESGYLLCTVQAINRTFVGTDSGMHHLIR
48477088_Picrophilus_torrid         IGGGFGVPYKEIIIEPGRYLVADAGIALATVTDLKRSIAGTDLGMNILIR
126007985_Ferroplasma_acidar        IGGGFGVPYTRLIIEPGRYIVADSAILLSKVTSIKRELIGIDASMNTLIR
60682355_Bacteroides_fragil         VGGGLGIDYDTLHFELGRAITGQCGSLISKVLYVKKQFAILDAGMTDLIR
123416580_Trichomonas_vagina        VGGGLGVSYTEIHFELGRSIICQCGSLVTKLLYIKRNFAIVDGGMTELIR
45358763_Methanococcus_maripa       LGGGLGIPYDNLILEPGRSLVATAGVLLGTVEHVKAKWVMIDAGMNDMMR
15644265_Thermotoga_mariti          IGGGWGINYSRVIVEIGRYIVAPSGYLLLRVVLVKKAFVVVDGGMNVLIR
CM1350_Cyanidioschyzon_merolae      IGGGLGIDYETLIIEPGRSLIGNAGILVCRVIGVKKRFIVVDGSMAELIR
15231844_Arabidopsis_thalia         IGGGLGIDYYNLIIEPGRSLIANTCCFVNHVTGVKKNFIVIDGSMAELIR
88601236_Glaucocystis_nostoc        ----------GIMVEPGRSIVGNTGMLVNKVTGVKKNFIVTDGSMAELIR
8134392_Archaaeoglobus_fulgi        MGGGLGIDYEQLWLEPGRSIVGNTTVLITRVNAVKKNFVAVDAGFNVLIR
67919461_Chlorobium_limico          IGGGFPVTYDPVIFEPGRFLVANASVLLSRILYKKKQFYIVDAGMTELIR
91202264_Candidatus_Kuenen          IGGGYCISYTRVIMEPGRFITGNSGILVTSVIYNKKKFVICDAAMNDLLR
94970467_Acidobacteria_bacter       GGGGLGIAYRHLLLEPGRAIIGPAGALLTRVVYSKKTFVIVDAGMNDLIR
15606446_Aquifex_aeolic             IGGGLGIKYKKIILEPGRSIMGNAGILITQVQFLKKHFIIVDAGMNDLIR
108802931_Rubrobacter_xylano        IGGGLGIRYRKILCEMGRYIAGPAGALLTGVLYRKRSFIVADAGMNDLIR
124516709_Leptospirillum_sp.        VGGGLGIRYGGIILEPGRSIVGNAGILVTEVQYLKKMFYIADAGMNDLIR
94417748_Pseudomonas_aerugi         LGGGLGVRYRTLVFEPGRSIVANAGVLLTRVEYLKKDFAIVDAAMNDLIR
94499872_Oceanobacter_sp.           LGGGLGVTYDKLVLEPGRSIAANAGIFVTQVEFLKKHFAIIDGGMNDLIR
77920014_Pelobacter_carbin          LGGGLGITYETLVFEPGRVMVGNAGMLVSKVLYVKKNFVIVDAAMNDLTR
15075673_Sinorhizobium_melilo       IGGGLGIPYRKIVTEPGRLIVGNAGILVTEVVYVKKTFVIVDGAMNDLIR
76258104_Chloroflexus_aurant        PGGGLGVPYTRLTVEPGRSIVARAGVALYRVVGRKEHWVHIDGGMADNIR
118062084_Roseiflexus_casten        IGGGAGVPYTHLVIEPGRSIIARAGVALYTIIATKMRFLHIDGGMGDNIR
23128550_Nostoc_puncti              VGGGLGIKYTKLLSEPGRSLIATACVTAYTVGSSKRTYVAIDGGMSDNPR
33240555_Prochlorococcus_marin      LGGGLGIRYILLMCEPGRSIVGSAGLTLYKIGSRKKTYLSVDGGMSDNPR
87303422_Synechococcus_sp.          VGGGLGIRYVRLLCEPGRSLVATAGLTVYTIGSRKRTYLSVDGGMSDNPR
21223714_Streptomyces_coelic        LGGGLGIAYTRISVEPGRAIVGPTAFTLYEVGTVKRTYVSVDGGMSDNIR
116671170_Arthrobacter_sp.          LGGGYGIAYTRISIEPGRAIVGTSTFTLYEVGTLKRRYVSVDGGMSDNAR
118443019_Clostridium_novyi         LGGGFGIYYGSLVIEPGRSIVGNAGTTLYTVGSIKRKYVSVDGGMTDNIR
51892948_Symbiobacterium_therm      LGGGLGVRYVKLIVEPGRSIVAEAGTTLYTVGTIKRTYLSVDGGMGDNIR
16079395_Bacillus_subtil            LGGGFGIRYTEIWIEPGRSLVGDAGTTLYTVGSQKRQYVAVDGGMNDNIR
                                               :  * *  :                     *  :    *
```

```
66825991_Dictyostelium_discoi       PGMYGAYHHITVLYDVVGSLCENNDKFCIDRSLKIHRGDTLVIHDTGAHG
24213747_Leptospira_interr          PSLYAAKHPLVVVYVYVGHCCESGDLFTQARITEAKLGDYVVMEGAGAYC
116056027_Ostreococcus_tauri]       PSLYGSQHPLVSVYVVVGHCCESGDLMTPARTMEVNIGDFIVIEGSGAYC
87306324_Blastopirellula_marin      PILYGSYHPMSIAVVVGGPLCESGDIFTQERSLAASIGDFLVIECAGAYG
21224744_Streptomyces_coelic        PAMYGSNHRVSVLTVLAGPLCESGDVFTQVVPVRTDVGDLVVFHDTGAYG
75239202_Escherichia_coli           PAMYGSYHHISALTVVAGPLCESGDVFTQQRALEVKAGDYLVLHDTGAYG
118385090_Tetrahymena_thermo        PALYESYHKIENAVNVCGNICESGDVFGQNRLITVKDDDILAIYNGGAYG
145525334_Paramecium_tetrau         PALYESYHEIINSVTVCGNICESGDLLGVDRNLKVEVNDVLVIQNGGAYG
48477088_Picrophilus_torrid         PALYGAVHEIICIYDITGQICENTDFTGLNVKLGLEPGDILGIMNAGAYV
126007985_Ferroplasma_acidar        IPLYGARHGILLSFDIVGQVCENTDYIAKDISIEPAVGDIVVIADAGAYV
60682355_Bacteroides_fragil         PALYQAHHKMENLYDVVGPICESSDVFGKAVDLKVKRGDLIALRSAGAYG
123416580_Trichomonas_vagina        PALYNAFHLVENVFDVVGPVCESSDVIAHSVKMDAKRGDYVIIRSAGAYG
45358763_Methanococcus_maripa       PAIYESYHEITPCVSVAGGLCESSDVFGKDREIKMEVKDTVAILDVGAYG
15644265_Thermotoga_mariti          PALYSAYHRIFVLADVVGPLCESGDVIAYDRELEVEPGDIIAVENAGAYG
CM1350_Cyanidioschyzon_merolae      PALYGAYQLITLTYDIVGPVCESSDFLGKARVLLPAPGTGLAVMDSGAYC
15231844_Arabidopsis_thalia         PSLYDAYQHIELVFDVVGPVCESADFLGKDRELTPPQGAGLVVHDAGAYC
88601236_Glaucocystis_nostoc        PSLYDAFMYIDFIFDIVGPVCESADFLGKDRMLTPPEGAGIAVFDAGAYS
8134392_Archaeoglobus_fulgi         PAMYGSYHRVAVAYTVVGPICESGDVLARDRKLKVEVGDLIAVFDAGAYG
67919461_Chlorobium_limico          PALYQSHHEVLTVADVVGPVCESSDFFARQRTIDAAEGELLAVMSSGAYA
91202264_Candidatus_Kuenen          PALYDAFHRIWPVVDIVGPVCESSDVFAKDRAIPVNEGDLLAIFSAGAYG
94970467_Acidobacteria_bacter       PALYGAYHEIVAAVDVVGPVCETGDFFARDRELHLEEGDLVAILDAGAYG
15606446_Aquifex_aeolic             PSIYNAYHHIIPVADIVGPICETGDFLALDREIEVQRGEYLAVLSAGAYG
108802931_Rubrobacter_xylano        PSLYGAYHEVLPLADLVGPVCESGDFLARDRDLDAREGDVLAVMNAGAYG
124516709_Leptospirillum_sp.        PSLYGAFHDLWPVGDLVGPVCETGDFLVQDRELPAEPGDLLAVMSAGAYG
94417748_Pseudomonas_aerugi         PALYQAWMDVQAVYDLVGPICETGDFLAKDRELALAEGDLLAIRSAGAYG
94499872_Oceanobacter_sp.           PSLYSAWQRIVEVYDVVGPVCETGDFLGKDRELAILPGDLLAVKSAGAYG
77920014_Pelobacter_carbin          PALYGSYQGIQAVADVVGPICESGDFLAQDREIAFSQGDLLAVASAGAYG
15075673_Sinorhizobium_melilo       PTLYEAYHEIRPVADVVGPVCETGDYLALDREMMPKPGDLFAIGSAGAYG
76258104_Chloroflexus_aurant        PALYGARYSAVLAVNVGGRYCESGDVLLRDIALTVQPDDLLAVATAGAYT
118062084_Roseiflexus_casten        PALYGARYSAVLAVAITGRYCESGDVLIHAAPLRASVGDILAVPVAGAYT
23128550_Nostoc_puncti              PITYQSVYRAVVAVTIAGKHCESGDILIKNALLKTEPGDILVVMGTGAYN
33240555_Prochlorococcus_marin      PITYQSEYSACLVVTIAGKHCESGDVLLKDFRLRSNSGDVLGVFSTGAYN
87303422_Synechococcus_sp.          PITYQSSYTAVLAVTVAGKHCESGDVLLKDLALPSSSGDLLVVFATGAYN
21223714_Streptomyces_coelic        TALYDAEYSVALAVRVVGKHCESGDIVVKDAFLDLAPGDLIAVPATGAYC
116671170_Arthrobacter_sp.          PVLYDADYSAIVASRVVGKHCESGDIVVRDVYLDVAAGDLLAVPGTGAYC
118443019_Clostridium_novyi         PALYRASYECLVAVTISGKCCESGDILLENVKVHAESGDILAVLTTGAYG
51892948_Symbiobacterium_therm      PALYHARYECAVAVTVVGRYCESGDVLLTDAAVDVAPGDLLAVFATGAYN
16079395_Bacillus_subtil            PALYQAKYEAAAAVSIAGKCCESGDMLIWDIDLEVKEGDLLAVFCTGAYG
                                       * :           *  **. *               . .   **:

66825991_Dictyostelium_discoi       HSMGFNYNGKLRSAEYLLLNNSINCIRRAETFDDLFS
24213747_Leptospira_interr          SSMSTNYNSFPETAEVLVDSDSFQLIRRRQNWEQIFQ
116056027_Ostreococcus_tauri]       AGMASNYNSFPEAPEVMRRGADMVCIRRKQTLDQILS
87306324_Blastopirellula_marin      FVMGSNYNSKPLAAEVLIRDGQTHLIRERQTFENLIA
21224744_Streptomyces_coelic        ASMSSTYNSRPLIPEVLVDGAETRLIRRRQTVAELLA
75239202_Escherichia_coli           ASMSSNYNSRPLLPEVLFDNGQARLIRRRQTIEELLA
118385090_Tetrahymena_thermo        YCMASNYNSRNRPAEILVDGSVDKLIRRRETLEDQLA
145525334_Paramecium_tetrau         FSMSSNYNLRERPAEILVRDSKPYIIRKRETLDYLLA
48477088_Picrophilus_torrid         SSMSSNYNSLSRAAEVLISDDNVHLIKKRDDIRDIIN
126007985_Ferroplasma_acidar        ASMASNYNLLSRPAELVLEGDREILTKGHDGLESMLA
60682355_Bacteroides_fragil         EIMASGYNCRELPKGYI-----------SEELV----
123416580_Trichomonas_vagina        ESMASSYLCRDIPLGYI-----------SEEFQDN--
45358763_Methanococcus_maripa       ISMANNYNSRGKPAMILTNEKEVSLIRARETLADLIS
15644265_Thermotoga_mariti          YTMSNNYNSTTRPAEVLVRENRISLIRRRETEMDIFK
CM1350_Cyanidioschyzon_merolae      MSMASNYNMKVLPGELMVQGNRWRIIRQRQRFADLLS
15231844_Arabidopsis_thalia         MSMASTYNLKMRPPEYWVEEDSITKIRHAETFDDHLR
88601236_Glaucocystis_nostoc        YCMSSNYNMKMRPPEYLVDGDKVTLIRKGESLDEWMK
8134392_Archaeoglobus_fulgi         FVMSSQYNGRPRCAEVLVSGDRWDVIREKESYGDLIE
67919461_Chlorobium_limico          AVMSSNYNGRLRPAEVMVDGQEVTVIRRRETYEQLIA
91202264_Candidatus_Kuenen          FTMSSSYNSRPRSCEILVDRDKYIVIRKRETYEDLIA
94970467_Acidobacteria_bacter       MSQTSNYNTRPRPAEVLVEGSRAKVVRRRETIKDLLG
15606446_Aquifex_aeolic             FAMSSHYNMRPRAAEVLVENGSVKLIRKRENYDYIVE
108802931_Rubrobacter_xylano        FSMASNYNSRPRPAEVLVREDRWAVVREREHYPDLIR
124516709_Leptospirillum_sp.        FAMASNYNARPRPAEVLVSGDRFSVIRARETYEDLIR
94417748_Pseudomonas_aerugi         FVMSSNYNTRGRAAEVLVDGERAHEVRRRETVPELYA
94499872_Oceanobacter_sp.           FVMASNYNTRNKPAEVMVDGDQSHLIRRRETISDQLM
77920014_Pelobacter_carbin          FTMSSNYNSRRRVPEIMVKGDQVMVIRKRETYDDLIK
15075673_Sinorhizobium_melilo       AVQAGTYNSRLLVPEVLVKGDRFHVIRPRRDYDELIG
76258104_Chloroflexus_aurant        LSMSSTYNLVPRPALVLVADGQARLIQRRETEADVLA
118062084_Roseiflexus_casten        LSMASTYNLTPRPAVVMVNGGSVRLIQRRETYEDMIA
23128550_Nostoc_puncti              YSMASNYNRLPRPAAVVVANGEANLILQRETYQDLIR
33240555_Prochlorococcus_marin      FSMSSNYNRIPKPAAIIVGNGESELIQKRELPEDLLR
```

```
87303422_Synechococcus_sp.         ASMASNYNRIPRPAAVLVHGGQAELVQRRERPDELLL
21223714_Streptomyces_coelic       RSMASNYNHALRPPVVAVRDGAARVVVRRETEEDLLR
116671170_Arthrobacter_sp.         WALSSNYNYLARPGVVAVRDGSARLIVRGETEQDLLN
118443019_Clostridium_novyi        YSMSSNYNKIPRPAVVFVNRGQSKLICKRQSYEDLVS
51892948_Symbiobacterium_therm     YSMASRYNRFPTPAVVFVRDGRADLVVRRETFADMAA
16079395_Bacillus_subtil           YSMANNYNRIPRPAVVFVENGEAHLVVKRETYEDIVK
                                         *
```

Note: All top hits in GenBank searches (using *Cyanidioschyzon* sequence and *Arabidopsis* GI 15231844 as queries) are from various non-cyanobacterial groups. The donor of the acquired gene in primary photosynthetic eukaryotes (upper part of the tree) is difficult to pinpoint because of the lack of sufficient internal support on the gene tree, but it is unlikely from cyanobacteria based on AU tests. The *Arabidopsis* sequence (GenBank GI 15231844 and TAIR locus AT3G14390) is annotated as a chloroplast precursor.

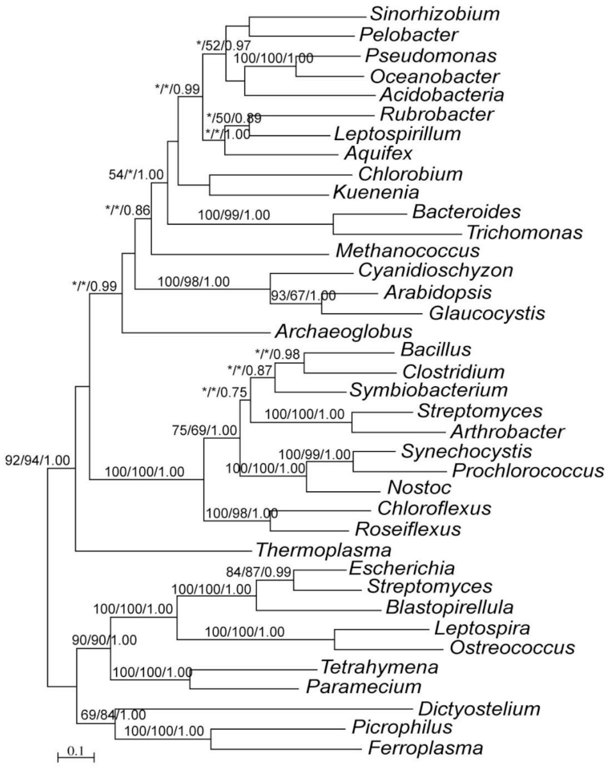

Figure 5. Molecular phylogeny of lysA. See text for detailed explanation. P-value = 0.235 from AU tests for the presented tree. AU tests were also performed on alternative topologies, including (**A**) monophyly of all eukaryotic sequences, (**B**) monophyly of cyanobacterial and primary photosynthetic eukaryotic sequences (except for the *Ostreococcus* sequence that appears to be distant). These tests investigate if red algal, green plant and glaucophyte sequences have the same origin (mitochondrial or eukaryotic) with other eukaryotic sequences and if red algae, green plants and glaucophytes acquired this gene from plastids. P-values < 0.001 from AU tests for both alternative topologies.

---

6. MGDG synthase (N)

CLUSTAL X (1.83.1) multiple sequence alignment

```
125975655_Clostridium_thermo       NILILSSNTGHGHKSITESLLEQFSH-YPDV-NVHVIDYGSVTRNAKLWK
89202509_Bacillus_cereus           KVLILTAHYGNGHVQVAKTLEQAFHQKGIE--DVIVCDYLKSYTIGKLYR
49483181_Staphylococcus_aureus     KILIITGSFGNGHMQVTQSIVNQLNDMNLDHLSVIEHDYINSFKYFRMYK
56478620_Azoarcus_sp.              IVDLIYFNAGGGHRATAVALGAVLRQRKPE-WQVRLVNYRSITGSAPDWY
116623425_Solibacter_usitat        KLDFIYFDAGGGHRAAANALRQVMEQ-QGRPCEIRMVNRKFTGLRLQIYN
125974899_Clostridium_thermo       NVLFLSISLGSGHIRAAEALQKFVVQKYPKS-KTLIVDYLNIVKYVPIYG
76259735_Chloroflexus_aurant       DVLFAISDTGGGHRSAAVAISAALEQMSGASITWAIEDYDQLSTRWLLYN
118063998_Roseiflexus_casten       RILFAISDTGGGHRSGAQAIAAAIEQRVGDAVETYIIDYDKLSTRWLLYD
118062512_Roseiflexus_casten       RILILTTDAGSGHRSAAQAVEAALLHVYRHNVQVTIANYLSTIQHAPRYD
145343579_Ostreococcus_lucima      RVMCLMSDTGGGHRASAQALKDCFELIYGDEFSVHIVDYFFLVKNPWLWR
18397057_Arabidopsis_thalia        TVLILMSDTGGGHRASAEAIRDAFKIEFGDDYRIIIKDYKFMVKHVGLWS
115475782_Oryza_sativa             NVLILMSDTGGGHRASAEALRDAFRLEFGDAYQVFVRDYKFMIRHVRLWK
30687800_Arabidopsis_thalia        NVLILMSDTGGGHRASAEAIRDAFKIEFGDKYRVIVKDYKFMVKHVQLWK
115479239_Oryza_sativa             KVLILMSDTGGGHRASAEAIKAAFIQEFGDDYQVFVTDYSFLVKHGPLWK
30689152_Arabidopsis_thalia        KVLILMSDTGGGHRASAEAIRAAFNQEFGDEYQVFITDYNFLVKHGTLWK
CM1391_Cyanidioschyzon_merolae     RALLLISDTGGGHRASAYAIRDAMNELYPGAFEWFIVDYQFLAKNPPLWR
GTO00086114_Guillardia_theta       ---ILQS--------------------APRATSSHMKD------------
94985286_Deinococcus_geothe        RALILSASFGSGHHQANDALDRALRA-AGVNLRARHADYAAWLRYAPMYK
51891890_Symbiobacterium_therm     RILLLSADFGSGHLAAATAIAAACRSLSADCEAVVVQCYLWQIRHTPLYR
37522798_Gloeobacter_violac        RILIFHASLGSGHIHAANALGEAFSR-YPDV-EVRIEDYEQLSEKAPLYR
76259734_Chloroflexus_aurant       RVLILHASVGTGHKRAAEALAAAFSRRQPG--EVRVEDYLELTDRAPVWG
78061400_Burkholderia_sp.          KILLLSVSAGAGHTRAAEAIR-AFADHHPAGIEATHLDYIKLVSSQPLWG
118063689_Roseiflexus_casten       RILILSTSAGSGHKAAAAALEKVFRQ-SPQVAELVNLDYLRLVQERPLVG


125975655_Clostridium_thermo       LVWELSFLKLICNIKPDLILSVHPNFNGSVLNIKVPFVTLLADIVSITPL
89202509_Bacillus_cereus           LFYYGVLKALLHTEKPDIVINTFPIIAVPELKKSIPVYNVLTDF-CLHKI
49483181_Staphylococcus_aureus     GFYYSRLINLLIKEKPDLILLTFPTPVMSVLTENIPVATVMTDY-RLHKN
56478620_Azoarcus_sp.              NKRLARFQQHWAQTEPDLVVSLVPNFNRALFESGVPHVTMLTDLADVPPF
116623425_Solibacter_usitat        LLLKKGLEAFWQKSRPDMVVSLVPNFDRALCESGVPFVTILTDIADYPPF
125974899_Clostridium_thermo       GLYRMSIHRLIQSFKPSIIVCTHPFPLQMIAHLDVPSIAIVTDF-VNHPF
76259735_Chloroflexus_aurant       FSFQLTLNRLLTERRPRLVVVTHPLVHRLVCAASFRVLTVVTDLVTLHAS
118063998_Roseiflexus_casten       TLYRLTILRVLEAVQPTLVVSVHPLLNRLIGNASFRFITVVTDLVSLHAS
118062512_Roseiflexus_casten       RAHTLTLHRLLVRHAPDVVISVYPLFTALVADARPGLMTVVTDLGHVHHT
145343579_Ostreococcus_lucima      LNFRCSFARAFKEIDPHLIVSVHPLMQHVPMRVRVPFATVVTDLTRCHAT
18397057_Arabidopsis_thalia        VAFHGTIEAGLMEYKPDIIISVHPLMQHIPLWVKVIFVTVITDLNTCHRT
115475782_Oryza_sativa             VAFHGTVVAGIMRYNPDIIISVHPLMQHIPLWVKVPFVTVITDLNTCHPT
30687800_Arabidopsis_thalia        VAFHSTVEAGLMEYKPEIIISVHPLMQHIPLWVRVLFVTVITDLNTCHPT
115479239_Oryza_sativa             MTYYGTVAKGLMKYQPDVIISVHPLMQHVPLRIKIPFTTVVTDLSTCHPT
30689152_Arabidopsis_thalia        MTYYGTIAQGLMKYQPDIIISVHPLMQHVPLRVKIVFTTVITDLSTCHPT
CM1391_Cyanidioschyzon_merolae     ATYCYGIRAAINCIRPDLVISLHPLTQMLPLRVRIPFVTVVTDLGGAHAT
GTO00086114_Guillardia_theta       ---------------------------------RIPFVTVVTDLGEAHPW
94985286_Deinococcus_geothe        AFYEWTMLRDLRAVRPEVVVSSFPTPVALAHTADFLNALVVTDYRVHHH-
51891890_Symbiobacterium_therm     QLYRLPVRRALQQYNPDVVVGTHPFFPAGAALHLRVPVVMALTDF-APHGF
37522798_Gloeobacter_violac        LIYEGSFEQLIKQTAPDVFICVQQIPSRLVQLF--PHYVVITDA-VAHST
76259734_Chloroflexus_aurant       YFYTQTLKEVLRAFQPEVIICTHFLPMELLVSYTEPIYCVITDY-AAHTF
78061400_Burkholderia_sp.          YLYQKTLLAEIERQRPDAIICTHFLPAELLSREDTPVWVQVTDF-DLHSM
118063689_Roseiflexus_casten       WWYQASLTRFIREFKPTITVCTHFMPAGIVAQLDTRLAIVTTDY-DFHSM
                                                                            :*

125975655_Clostridium_thermo       WADPRADYIICPSKESKFKCLEFGVSESKLIETGFPVRQKFENNTQNIKK
89202509_Bacillus_cereus           WIHREVDRYFVATDHVKQVMIEIGVPAERIVETGIPIRKNFSELIYNKYQ
49483181_Staphylococcus_aureus     WITPYSTRYYVATKETKQDFIDVGIDPSTVKVTGIPIDNKFQKQWLIDNN
56478620_Azoarcus_sp.              WIEPDQDQHFCGTPKAVEQARALGHAEERIATSGMIIRPDFRARERHRYG
116623425_Solibacter_usitat        WIEEKQDQHFCGSAKAVEQARAMGHPESKVRVSGMILNPRFRDAARRGLG
125974899_Clostridium_thermo       WFQNNIEAYIVAHDYIKRDMIECGISEDRIFTYGLPVAPEFKEQARKELS
76259735_Chloroflexus_aurant       WSYPGVDLALTPTDEAYRLMHKRGMKPSQLQRCGFPVHPKFAPTARRDLG
118063998_Roseiflexus_casten       WADPDAELCIVPTNEAYERMLRLGMPESKLVRTGFPVHPKFRDAAQANLG
118062512_Roseiflexus_casten       WFSPVDDLCIVPNAQVRTRALSCGLNPRQVQIVGIPVHPRFPATVRRDLG
145343579_Ostreococcus_lucima      WFHRSVDRCFVATNLVAAQAMRLGLKSEQIACHGLPIRPSFKHELRTKLA
18397057_Arabidopsis_thalia        WFHHGVSRCYCPSKEVAKRALVDGLDDSQIRVFGLPVRPSFKNELRKELE
115475782_Oryza_sativa             WFHHGVTRCYCPSAEVAKRALLRGLEPSQIRVYGLPIRPSFKDELRKELD
30687800_Arabidopsis_thalia        WFHPGVNRCYCPSQEVAKRALFDGLDESQVRVFGLPVRPSFKDDLRKELE
115479239_Oryza_sativa             WFHKLVTRCYCPSAEVSKRALKAGLQPSQIKVYGLPVRPSFEDELRRELG
30689152_Arabidopsis_thalia        WFHKLVTRCYCPSTEVAKRAQKAGLETSQIKVYGLPVRPSFKVELRRELG
CM1391_Cyanidioschyzon_merolae     WFDPDADLVFVASDAVRQLAIRCGVHPERIRQHGLPVRPEFRRAWRRRLG
GTO00086114_Guillardia_theta       WFNKGLDKMFVPIEEMKAQALEFGLKEEQVSVCGLPLRKGFKELMRDKLE
94985286_Deinococcus_geothe        WARPEAELLMVANEEAREQMGRWRIPDANVAVTGIPIAPVYRTALREQHG
51891890_Symbiobacterium_therm     WIWPGVARYFTASEQAARELVRRGADAAAVRATGIPVRMALRTAGPTAGS
37522798_Gloeobacter_violac        WINYEVDGYFLASDLTANILTKQGVDPELLHVTGIPVKLEIARQMRERHD
76259734_Chloroflexus_aurant       WTYTEIDGYFVGDEQTRAQLIERGVSPQQIVVSGIPIDPAFCREARQRRE
78061400_Burkholderia_sp.          WVVPNMRGYFAANDEIAWRMHARGMAPDTVHVSGIPIMPAFRAACAAEFG
```

```
118063689_Roseiflexus_casten        WLSPRFNRYFVALEETKVHLMALGLPEDRITISGIPVDPAFRDAALARYH
                                    *                            :   *: :

125975655_Clostridium_thermo        YTGDRPLCLIMSGGEGSGNMSRIASILLKRVKIVTGRNRLLKRRLFGDRV
89202509_Bacillus_cereus            LSREKKILLIVAGAHGVGNVKDLCASFMSQVAVVCGKNDALKQELNSEAL
49483181_Staphylococcus_aureus      LDPDKQTILMSAGAFGVKGFDTMITDILAQVVMICGKSKELKRSLSNENV
56478620_Azoarcus_sp.               LDPLRPTGVVLFGGHGSKAMLGIAKKLAEQLILFCGHNKALARQLQRAPR
116623425_Solibacter_usitat         FAPAQPVGLVMFGGEGSAVMRQIARSLEDQLILICGKNTKLRERLHRAPI
125974899_Clostridium_thermo        LENTLT-VLLMGGSLGIGDIENTFKSFAKQIIAVAGKNTALKKRLFPMPV
76259735_Chloroflexus_aurant        LEPDRFTVLLTAGGVGSGRLGELVQTLEQQFLVVTGKNRALYEELRLPHT
118063998_Roseiflexus_casten        LAPELFTVLVTSGGVGSGNMEQLVRNIHTQVLVVTGRNTALRERLFGPNV
118062512_Roseiflexus_casten        WRTDLVTVLISGGGAGVGPLAELAIAADEQIAVIAGRNSDLAARLWKNPV
145343579_Ostreococcus_lucima       LDTDAPTVMLVGGGEGMGKLQETAEALAQQVIVVCGRNLRLSDFLWPLKM
18397057_Arabidopsis_thalia         IDLNLPAVLLMGGGEGMGPVQKTALALGDQLIVICGRNKVLASTLWKIPV
115475782_Oryza_sativa              MDPDLPAVLLMGGGEGMGPVEETARALSDQIVVICGRNQVLRSTLWNVPV
30687800_Arabidopsis_thalia         MDQDLRAVLLMGGGEGMGPVKETAKALEEQMVVICGRNKKLASALWKIPV
115479239_Oryza_sativa              MDEYLPAVLLMGGGEGMGPIEATARALGDQILVICGRNKKLTSRLWKVPV
30689152_Arabidopsis_thalia         MDENLPAVLLMGGGEGMGPIEATARALADQVLIICGRNKKLQSKLWKIPV
CM1391_Cyanidioschyzon_merolae      FLAEHRRVLIVGGGDGVGGLTRIARAVVEQVIVVCGRNERARNELTRTNL
GTO0086114_Guillardia_theta         LKQDWRVVILIGGGDGMGKLKECAMSFFAQIVIICGKNEKLQKQLTECDI
94985286_Deinococcus_geothe         LKPGEPLILVSGGGTGTRALNRVLNELANQVLVLAGAQGRGVTRV-GATI
51891890_Symbiobacterium_therm      SSSGVRRVLVIGGGLGLGPIAAAVDALLSRVTVICGRNEALLDQISDARL
37522798_Gloeobacter_violac         LPLDAPVIALFGGGLQPRRVRLMVAGLLETLAVVAGRNHALGTALPAMRL
76259734_Chloroflexus_aurant        LPLDGTVITLFGGGVDDEHVRLIVSGLMQTLIVVAGRNTTLVESLPNIDL
78061400_Burkholderia_sp.           LDPARPTFLMMSGGAGLGGLDVLAARLLEQLIALAGKNKAMLASLHPGRL
118063689_Roseiflexus_casten        LRSDLPTLLLSAGAAGIGPAREAVQQILQQVIVVCGRNEELRAELQAKRF
                                             :  *.                .  . * .      :

125975655_Clostridium_thermo        EIYGFTENIQDLMLSSDIAFTRGSPNVMMEAVACNVPLIITGNLPGQEEG
89202509_Bacillus_cereus            KVFGYIENIDELFRVTSCMITKPGGITLSEAAALQVPVILYKPVPGQENE
49483181_Staphylococcus_aureus      LILGYTKHMNEWMASSQLMITKPGGITITEGFARCIPMIFLNPAPGQELE
56478620_Azoarcus_sp.               LVIEFSLDIPCYMRLADFFIGKPGPGSISEAVQQGLPVIVNAWTMPQERY
116623425_Solibacter_usitat         FVEGFTKEVPRYMQLADYFIGKPGPGSVSEALAMKLPVIVNAWTLPQERY
125974899_Clostridium_thermo        KIFGYTDSIPMLMDASDFIVTKPGAMTISEALVKRLPALIISPIPGQEER
76259735_Chloroflexus_aurant        HVFGFVNNMEELMAASDVVVTKAGPGTLMEALVMRKPVIVTEAVGLQEHG
118063998_Roseiflexus_casten        RIFGFVTNMEELMAASDIVISKAGPGTLMEALVMRRPVIVTQAVGMQERG
118062512_Roseiflexus_casten        HIYGFVP-LADMMYAADIIATKAGGLSVSEALAVGRPLLIYGSAPGQEAG
145343579_Ostreococcus_lucima       VVKGFVHNMAEYMSACDCVISKAGPGTIAEAMICGVPIVLNGCIPCQEEG
18397057_Arabidopsis_thalia         KVRGFETQMEKWMGACDCIITKAGPGTIAEALICGLPIILNDYIPGQEKG
115475782_Oryza_sativa              KIRGFEKQMEKWMGACDCIITKAGPGTIAEALIRGLPIILNDFIPGQEVG
30687800_Arabidopsis_thalia         KVRGFETQMEKWMGACDCIITKAGPGTIAESLIRSLPIILNDYIPGQEKG
115479239_Oryza_sativa              QVKGFVTKMEECMGACDCIITKAGPGTIAEAMIRGLPIILNGYIAGQ---
30689152_Arabidopsis_thalia         QVKGFITKMEECMGACDCIITKAGPGTIAEAMIRGLPIILNGYIAGQVSR
CM1391_Cyanidioschyzon_merolae      TVFGFVSNMGELMAAADAIVTKAGPGTIAEALIRGVPIMLSGFLPGQEEG
GTO0086114_Guillardia_theta         RILGFVSNMEEWMIAADVLVTKAGPGTIAEACCCGLPVLLFDFLPGQEEG
94985286_Deinococcus_geothe         HALGFTTAFPELLAASDLVVGKAGGLTVAEATALGVPLVIFEPIPGQEEH
51891890_Symbiobacterium_therm      TALGFTDQVLDHMREADLLVTKPGGITCAEALALGLPMLLLDPLPGPEEE
37522798_Gloeobacter_violac         RSLGQIDFVDDLIAASDLVITKAGGLIASEVMARGTPMVIVDPIPGQEEW
76259734_Chloroflexus_aurant        RVLGFVDYVDDLITASDLVITKAGGLIVSEVLARGTPMVIIDPIPGQEEW
78061400_Burkholderia_sp.           FPQGFTQQVERLMACADLVITKPGGLTTSECLAMQLPMIVNSPIPGQEER
118063689_Roseiflexus_casten        RVLGFTDDMHVLMRIATILIGKPGGLTSSEAMAAGLPMVIIAPIPGQEER
                                            .   :        : .     *      *  :.

125975655_Clostridium_thermo        NPAYMQKYNLGVVCKDVRKLRHTVNELLIKQSQKEFLNPNVAKEIVSFL
89202509_Bacillus_cereus            NAIYFESKGAAVVIREDAEIFEKTKALLMKEAMGSIYRPEPAAHIVDVI
49483181_Staphylococcus_aureus      NALYFEEKGFGKIADTPEEAIKIVASLTMISTMEQDKIKYATQTICQDL
56478620_Azoarcus_sp.               NAAWVRDNHLGIVHGSFRTIDRAVAQLLFRVSVRSIDNR-ALFEVPEIL
116623425_Solibacter_usitat         NGDWLREMGAGMVLPDFRGIARAVEELLYREAAERYNNR-AIFEIPDIL
125974899_Clostridium_thermo        NEQFLVNSGTAVRIYKNTKIDSVLCQVYMKEIAGNLANPDSGRNILSLI
76259735_Chloroflexus_aurant        NIDFVLNYELGFFCPTNERIVAAISQLETVERLKHAVPRDGAIQIARVI
118063998_Roseiflexus_casten        NIDFVLNHELGLFCPTIDRIVPVLAELMVTARLVDAVPRDGAAQIAAIL
118062512_Roseiflexus_casten        NLEYVMRRGAAQYTPDAAQFVAALQRWIAADAARSAGRPQAAFEIASMV
145343579_Ostreococcus_lucima       NIPFVTDNKVGAYSENPRTIAQIVAEWLMSSRARALGRPEATFDIIRDL
18397057_Arabidopsis_thalia         NVPYVVDNGAGVFTRSPKETAKIVADWFMSENALKLSQPEAVFDIVKDI
115475782_Oryza_sativa              NVPYVVDNGAGVFSKDPREAARQVARWFYSLNALKLAQPEAVFDIVKDI
30687800_Arabidopsis_thalia         NVPYVVENGAGVFTRSPKETARIVGEWFTSDNARKLAQPEAVFDIVKDI
115479239_Oryza_sativa              ----------------------------------------VILQSVNVV
30689152_Arabidopsis_thalia         ECTVRGGKRMWEILKITERDIEDCSGLV---------------RVGDNV
CM1391_Cyanidioschyzon_merolae      NVPFVVNHGVGEYHQGPVAIANRLAEWLMAIRARDLGKPLATYEICQDI
GTO0086114_Guillardia_theta         NVTFVESSGMGEFVKDTTK------------------------------
94985286_Deinococcus_geothe         NADYLLRHGAGLWARELTDVRPAVLRALLSAHARAISVPDAADRVAAAL
51891890_Symbiobacterium_therm      NAAYLAGSGAARVVGVKRLAGAADDLLFMAAAARQAGHPASALAIAAEV
37522798_Gloeobacter_violac         NADAIAAYGAGIQLRLPEMVAPTVQFLLMRFQARKYGRPTAALAVVESI
```

```
76259734_Chloroflexus_aurant        NADYVVSTGSGIQLRMCESTPRAVVNLLMRRCAQAAAHPRAALDIAEKV
78061400_Burkholderia_sp.           NADFLLEQGVALKAIDDDALVYRIHALLMRRRLAPLGRPLAGRFVLDRV
118063689_Roseiflexus_casten        NSDHLLEEGVALRCNQMTTLAYKIDRLLMRENTRNIGRPDAARVIVETL
```

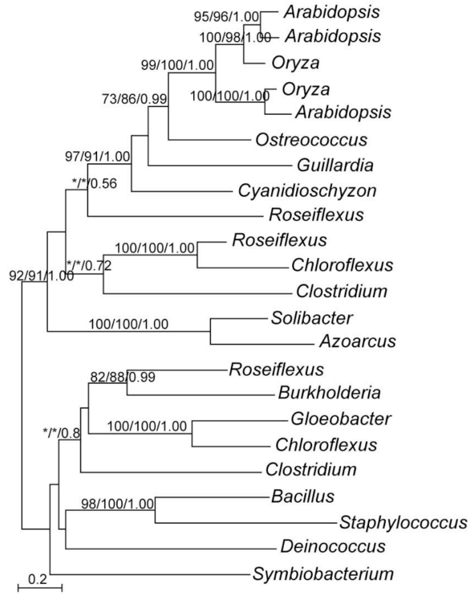

Figure 6. Molecular phylogeny of MGDG synthase. See text for detailed explanation. P-value = 0.235 from AU test on the presented tree. AU tests were also performed an alternative topology enforcing a monophyly of photosynthetic eukaryotic sequences with the rare cyanobacterial homolog (i.e., *Gloeobacter* in this case). P-value < 0.001 from AU test for the alternative topology.

---

7. Alpha amylase (D)

```
CLUSTAL X (1.83.1) multiple sequence alignment


CM1405_Cyanidioschyzon_merolae      YGCHPTVNFAIYSTEQRHMSLIIYNRMGKVWHVAVQAGYRYAWRVRGKG-
33635247_Prochlorococcus_marin      LGSTIGVNFSVAAPTAKRVELLLFHRSGDYWHVEVETGCCYGYRIFGPHH
149177467_Planctomyces_maris        LGETWAYNFAIYSKHAERVSLLFFNKTAEIWHCRIPPATYYAYQIEGPSD
32398065_Rhodopirellula_baltic      LGASWSFNFSLYSRHATAVHLLLYNKSGPVWHCRVREWAYYAYRVDGPAD
33236242_Chlamydophila_pneumo       LGASKRYRFALYASQATEVILALTHRTGAIWHIEIEDQSSYAFRVHGP-S
42525620_Treponema_dentic           LGAKLGVNFSVFSRNAKEIVLHLFNKTGDVWHVFVSSWAFYLYTADGEFD
19714344_Fusobacterium_nuclea       LGANLGCSFAIYAKNVNSLSLNIFHKLGDIWSIFLEEGTLYNWEING---
150017328_Clostridium_beijer        NGAVVGVNFTIHSQSATSCKLLLFYRIGNVYSMIVLEEFEYAYSVDGPYN
48894542_Trichodesmium_erythr       FGATMGVNFSIFSSYATSCTLVLFFRIGNVYCMIVFENIEYGYRMDGPND
16330244_Synechocystis_sp.          FGATIGVNFSIYSSHSTACTLVLFFRIGNVYCMVVFENLEYGYRMEGPND
CM4050_Cyanidioschyzon_merolae      YGVSLGTNFAIFTDEEEHVALILYHRTGHVWHVQIAEHYAYAWRIDRPTG
22328517_Arabidopsis_thalia         LGVSQGINFALFSQNATSVTLCLSNKTGDTWHICVENNVLYGYRVDGPGD
15225595_Arabidopsis_thalia         FGPTVGVNFSVYSTNSVSATICLINRTGHVWHVFLRKDMLYGYRFDGKFD
46446740_Parachlamydia_sp.          FGANIKVNFALYAKNIEKISLCLFNKTGNVWHIAIEPYTLYAFRV-----
116626210_Solibacter_usitatus       LGATLGVNFAIYSKQAAGVFLLLFARDKLVWHACVKAGQLYGYKVSGEYN
48765636_Rhodospirillum_rubrum      LGAMAGVNIAVFSEAATQIDLCLFARTGDIWHGFLPPGLRYGLRAHGPYN
24373069_Shewanella_oneide          LGATVGVNFALFSAHATGVELCLFEQTQQIWHLYVHAGQLYGYRVYGPYN
1707700_Sulfolobus_solfat           LGSNWGVNFSLFSENAEKVELLLYNKTGDIWHVFVPPGQLYAYRVYGPYN
48784580_Burkholderia_fungor        LGASWGVNFAVFSANAQKIELCLFECTDEVWHGYLPPGTAYGFRAHGPYN
17548459_Ralstonia_solana           LGAQWGINFAVFSANATRIDLCLFECTDEVWHGYLPSGLVYGYRAYGPWN
118048461_Chloroflexus_aggrega      LGATWGVNFAIFSAHATRVELCLFERSADIWHGYIPPGQLYGYRVYGPYN
```

```
48763460_Rhodospirillum_rubrum      LGATWGTNFALFSAHAEKVELCLFEYTDEIWHGYLPPGQMYGYRVYGPYN
58582972_Xanthomonas_oryzae         RGAVFGTNFALFSAHATRVEVCLFEYTNEIWHGYLPPGQRYGYRVHGPYN
58579740_Xanthomonas_oryzae         LGATWGVNFALYSRNATRVELCLFEYTDEVWHGYLPPGQLYGYRVHGPYN
26990756_Pseudomonas_putida         LGATWGVNFALFSANATKVELCLFEYTDEIYHGYLPPGLVYGYRVYGPYN
48785916_Burkholderia_fungor        LGATWGVNFALFSAHATKVELCLFEYTDEVWHVFVPPGAVYGYRVHGPYN
21223814_Streptomyces_coelic        LGARFGTNFALWAGGAEAVELCLFELTHEIWHGFVPPGQRYGYRVHGRWN
45916433_Mesorhizobium_sp.          LGATWGVNVAVFSAHAEKIELCLFEYTHEVWHGYFPPGQVYGLRAYGPYN
6457937_Deinococcus_radiod          LGATWGTNFALYSENATGVELCLFEQTAFVWHGYLPPGQRYGYRVHGEYN
48894700_Trichodesmium_erythr       LGSSWGTNFAIFSENATSVELCLFEVSNFIWHGYLPPGQKYGFRVHGPYN
108803168_Rubrobacter_xylanoph      LGATWGVNFALFSENAERVELCLFEQTDQIWHCYLPLGQLYGYRVYGPYN
25028566_Corynebacterium_effic      LGSKYGTNFALFSDVAESVELCLVEKDNNVWHCYLPPGQRYGYRVHGPWD
21224411_Streptomyces_coelic        LGATYGTNFAVFTEAADRVELCLLESDAFVRHAYVPPGQRYGYRVHGPYN
21225613_Streptomyces_coelic        LGAAYGTNFALFSEVAERVDLVLVDVDGFVWHCYLPPGQRYGYRVHGPWN
149179477_Planctomyces_maris        YGAVPGVLFSVYSRSATSMWLLLYGRLGDIWTAFISPGQLYHFQADGPFD
32446352_Rhodopirellula_baltic      FGATLGVQFSVFSRSATEMRLLLYDRWGDVWSLHVPAGQLYHFQASGPWD
150020446_Thermosipho_melanesi      LGATPGVNFALFSRHAERVILELYNKTGDIWHIYVYHGQYYGWRVDGPYN
48837599_Methanosarcina_barker      LGATPGVNFSIYSEHADYVELLLFNKTFHFWHVYVRPGVHYAYRIGGPFD
116622006_Solibacter_usitatus       LGATVGVNFSVFSRSASAIELLLFNRTYHYWHTFVPPGQLYGYRVHGPFD
48893487_Trichodesmium_erythr       IGATVGVNFSIYSKNATAIELLLFHRTHHYWHIFISAGQIYAYRVYGPFD
16329290_Synechocystis_sp.          LGATVGVNFCLFSKHAERVTLLLFNRTFYYWHVFVKAGQVYAYRVDGPHD
                                     *      . : :       : :           .     *

CM1405_Cyanidioschyzon_merolae      -AQIVLDPFARIIDSPLYYTPFAVIPPDMVIYEVHVRSFTAMRGTFTAVI
33635247_Prochlorococcus_marin      PSKVLLDPCTRAISGWDIYCLKGVVCERSVIYELHVGGFSSGQGTFLGLI
149177467_Planctomyces_maris        SEKLLFDPYSRNIYFPPDFAPLSVLQSNLIIYEMHVRGFTKRRGTFAGVI
32398065_Rhodopirellula_baltic      FQKILLDPFARGVFFPESFAPLGILPNDLVIYEMHVRGFTKNQGTFLGVV
33236242_Chlamydophila_pneumo       FKEYLADPYAKNIHSPQSFYAFCYLKEEMIIYEMHVRSFTQSPGTFLGII
42525620_Treponema_dentic           ENNYLLDPYARLISSHSVFFPKCVVIDKCVIYEAHVKGFSFLRGKYSGLV
19714344_Fusobacterium_nuclea       --MAILDPYALAYTGNETIIVLARVGTDMIIYESHIGLFTKSGATYSAFE
150017328_Clostridium_beijer        SSKYLLDPYSKAIAGQSVWQYKSRVVSDLIIYELHVRGFTKHPGTFAGLA
48894542_Trichodesmium_erythr       KSKILMDPYAKIIGGRDVWQHRGRIPYDQIIYEMHVRSFTAHPGTFAAIR
16330244_Synechocystis_sp.          PSKVLLDPYAKVVSGRDVWQHRGRLSFDMVIYEMHVRGFTKDRGTFAGIL
CM4050_Cyanidioschyzon_merolae      SN-IVLDPYAKCVFSPPSARPICGVPADLIIYELHVRGLTKQAGTYLGVI
22328517_Arabidopsis_thalia         RSILLLDPYAKLVKGHSSFFAQFYGTYDLVIYEMNVRAFTADGGSYLGFI
15225595_Arabidopsis_thalia         SSNILLDPYAKAIISRDEFQMACMVPTDLVIYEMHVRGFTRHPGTYQGVA
46446740_Parachlamydia_sp.          ---LLIDPYAKSIYSSPDWSPLGRIIPDLIIYEMHIRGLTQDPGTYLGVI
116626210_Solibacter_usitatus       EAKLLLDPYARAVTGKFRNVPKGIVIDQLVIYEVHVKGFTAHPGTYLGFI
48765636_Rhodospirillum_rubrum      PHKLLIDPAARRLDGPVVWVPKAVVEARLLIYEAHVKGLTRTRGTFDGVA
24373069_Shewanella_oneide          PHKLLLDPYARQLVGRYHHMPKCKVVDQCIIYEMHLKGFTALRGTFAGLA
1707700_Sulfolobus_solfat           PNKVLIDPYAKAINGSVIWVPKSVVINDTVIYEVHVKGFTKLRGTYEGLA
48784580_Burkholderia_fungor        PHKLLLDPYARKLVGQFRWMPKCVVIDETIVYETHVRGASMLRGTFAALA
17548459_Ralstonia_solana           PHKLLLDPYARRLVGALRWCPKAVVTDRTVIYEMHVRGASMLRGTFAALA
118048461_Chloroflexus_aggrega      PYKLLIDPYARALAGQLRWVPRSVVIDDSVIYELHVKGFTKLRGTYAGLA
48763460_Rhodospirillum_rubrum      ANKLLIDPYAKSLKGEILWMPKCEVVDETVIYEMHPRGFTMLRGTFDGLA
58582972_Xanthomonas_oryzae         HNKLLLDPYARELEGDLIWMPKCVVVEEMVIYETHVRGYTMGRGTFAGLA
58579740_Xanthomonas_oryzae         HNKLLLDPYAKQIVGELKWMPKSAVIDRTVIYEAHVRGLSMLRGTFSALK
26990756_Pseudomonas_putida         PNKLLIDPYAKQLVGSLKWVPKCKVIDRTIIYEAHARGISMRRGTFAGLA
48785916_Burkholderia_fungor        PNKLLLDPYAKAHIGELKWVPKCKVVDRVIFYEAHVRGFTKRRGTFAGLG
21223814_Streptomyces_coelic        PAKLLLDPYARAVDGDFGLVPKGVVVHDSVIYELHVRGFTKLRGTYAGLA
45916433_Mesorhizobium_sp.          PNKLLIDPYAKQLLGDIRWMPKCVVTDETIIYEAHVKGMTALRGTFGGLA
6457937_Deinococcus_radiod          PNVVLLDPYAKALDGTEQFAPLGLVVDQSVIYEAHVKGLTMTRGTYAGVA
48894700_Trichodesmium_erythr       PNKLLIDPYAKAIDGNILYMPKSVVIDESVIYEINLKGFTKLRGTYAGLA
108803168_Rubrobacter_xylanoph      PNKLLLDPYAKSIAGSLDWALKCRVVDDTIIYELHVKGFTRLRGTYAGLA
25028566_Corynebacterium_effic      ASKFLVDPYARAFVGEYDGSMKSVVVSETVIYEAHVKGMTMTRGTYAGLA
21224411_Streptomyces_coelic        SAKLLLDPYARAISGEVQWTMTSVVVNHTVIYEAHVKGLTMRRGTYAALA
21225613_Streptomyces_coelic        PAKLLLDPYTRAVDGLVDNTMLGVVTDESVIYEAHVRGLSRTRGTYAGLA
149179477_Planctomyces_maris        KRARLIDPYAKALAGNFQPPPKCVVVDDTVIYEMHVRGFTNSPGTYLGVI
32446352_Rhodopirellula_baltic      STARLIDPYAQALAGTYQKPPKCVVVDESIIYEMHVRGFTKSPGSYLGVI
150020446_Thermosipho_melanesi      LNKLLVDPYAKAISSSFDWPTKSIVIDDTVIYEMHVRLFTISRGTFLGII
48837599_Methanosarcina_barker      GDKVLIDPYSKG-NNKTLWSMRSVVIDETIIYELHVGGFTRSSGTFSGVI
116622006_Solibacter_usitatus       PRKLLLDPYGRGIVVPAGYAMKSVVIDRTIIYELHVRGFTRHRGTYAGLI
48893487_Trichodesmium_erythr       GTKVLLDPYARIVVGQEKYALKGVVVDKTVIYEMHVGGFTRHRGTFAGLI
16329290_Synechocystis_sp.          PDKVLLDPYAKAIVGKDIYALRSVVVDASIIYELHVGGFTRNRGTYAGLI
                                        : **                     :.** :    :   ..: ..

CM1405_Cyanidioschyzon_merolae      EKIPYLKALGINCVEFLPIFEFNELCQYWGYSTVAFFAPMNRFSLDEFRT
33635247_Prochlorococcus_marin      DKLPYLRKLGITAIELLPIFAFDPRDNYWGYSPLNWFTPHPKYVRQQVRE
149177467_Planctomyces_maris        EKIPYLQELGITAVELLPVYQFDT--NYWGYMPLGFFAPHDGYCHIEFCE
32398065_Rhodopirellula_baltic      DKIPYLVELGVTAVELMPVFQFDP--DYWGYMPLNFFAPHHAYARDEFCT
33236242_Chlamydophila_pneumo       EKIDHLHKLGINAVELLPIFEFDELCNYWGYAPLNFFSPCRRYASREFKT
42525620_Treponema_dentic           ELIPYLKDLGITSLELLPVFDFDELKNYWGYSTIAFFAPKALYAVNEFKF
19714344_Fusobacterium_nuclea       EKIPYLKNLGINVVEFLPIFEWDDLKNVWGYNPINFFALTKKYSINEFKK
150017328_Clostridium_beijer        EKIPYLKELGINAVELMPIFEFDELCDYWGYNPVCFFAPNTSYTGDELKS
```

```
48894542_Trichodesmium_erythr       EKIPYLKELGVNAVELMPIYEFDELYNYWGYSTVGFFAPKAGYAMDELKT
16330244_Synechocystis_sp.          SKIPYLQELGVNTIELMPIFEFDELVNYWGYSTVNFFAPKAGYAIDELKN
CM4050_Cyanidioschyzon_merolae      EKIPYLKQLGINAVELMPCAEFNELCQFWGYSPISFFAPMNRYAVREFKT
22328517_Arabidopsis_thalia         EKIPHLQDLGINAVELLPVFEFDEMVNTWGYSTVNFFAPMSRYASKEFKE
15225595_Arabidopsis_thalia         EKLDHLKELGINCIELMPCHEFNERVNFWGYSTIGFFSPMIRYAINEFKI
46446740_Parachlamydia_sp.          EKIPYLKELGINAVELMPIYEFNELVNYFGYSTVNFFSPMNRYALVEFKT
116626210_Solibacter_usitatus       EKIPHLTRLGVNAVELMPVHEHYVLTNYWGYNTIGFFAPEVSYGVAEFKT
48765636_Rhodospirillum_rubrum      GSIAHLKHMGVTALELLPVWAFADLTNYWGYNPVCLTAPHAAYGPDSFRR
24373069_Shewanella_oneide          SKIDYLVKLGVNCVELLPIQAFFSLSNYWGYNSIGFFAPEPSYLIIEFRT
1707700_Sulfolobus_solfat           SEISYLKDLGITTVELMPVFHFIDLTNYWGYDPINFFSPECRYSVLSFKK
48784580_Burkholderia_fungor        SPIDHLLKLGVTAVELLPVHAFLNLRNYWGYNSAAFFAPEPSYLLDEMRI
17548459_Ralstonia_solana           DPIEHLRALGVTAVELLPVHAFLQLRNYWGYSTLAFFAPEPAYLLHEMKV
118048461_Chloroflexus_aggrega      TPIAYLKELGVTAVELLPVQAFITLTNYWGYQPINYFSPEPRYAVNEFKA
48763460_Rhodospirillum_rubrum      HRTDYLTDLGVTAIELLPVHAYVQLTNYWGYNSIGFLAPHATYLASAFKA
58582972_Xanthomonas_oryzae         QPLQYIKDLGITAVELLPVHAYLDLRNYWGYNTIGFFALKSRYLRDEFRD
58579740_Xanthomonas_oryzae         TDIDHISSLGVTAVELLPVHAFVDLRNYWGYNTLGFFAPQARYMVAEFKQ
26990756_Pseudomonas_putida         NDLKHIKDLGVSSIELLPIHAFVNLNNYWGYNSIAFFAPHPRYLIAEFKE
48785916_Burkholderia_fungor        QQLDYIKNLGVTSVELMPIQTFVNLTNYWGYNTIGFFAADPRFFVGEFKE
21223814_Streptomyces_coelic        HPIAHLTRLGVTAVELLPVHQFAHLRNYWGYNSVGYFAPHAGYTVGEFKR
45916433_Mesorhizobium_sp.          DSIDYLVKLGITAIELLPVQAFFDLTNYWGYNTIGFFAPTPRYILHEFKR
6457937_Deinococcus_radiod          TPLDYLRDLGITAIEFLPVHQHVDLTNYWGYSTLNFFAPDVRYSVPEFKN
48894700_Trichodesmium_erythr       HPIAYLQSLGITAVELMPIHHFLRLSNYWGYDSINYFAPYSGYSVREFKQ
108803168_Rubrobacter_xylanoph      SAIEHLKRLGVTAVELMPVHFFVDLRNYWGYNSIGFFAPDARYSINEFKT
25028566_Corynebacterium_effic      HPIEYLVDLGITAIELMPVHQFMQLRNYWGYNTFGFFAPQNDYSVAEFKA
21224411_Streptomyces_coelic        HPIEHLTGLGVTALELMPVHQFVNLNNYWGYNTVGFFAPHNAYAVLEFKS
21225613_Streptomyces_coelic        HPVDHLTSLGVTAVELMPVHQFVHLSNYWGYNTIGFFAPHNGYAVSEFKS
149179477_Planctomyces_maris        EKIPYLIDLGVTAVELMPIHEFPMHQNYWGYETLAFFAPHRGFAVREFKE
32446352_Rhodopirellula_baltic      EKIPYLKSLGVTSVELMPVHEFPIRPNYWGYDPMAFFAPHRGYAVNEFKQ
150020446_Thermosipho_melanesi      EKLDHLKELGVTTIELMPVFEFNVLKDVWGYNPLGFFAVTGNYSVFLFKD
48837599_Methanosarcina_barker      DKIPYLKELGITAVELMPVFDFDDRKQYWGYDPICFFAPHSGYCMEEFRD
116622006_Solibacter_usitatus       EKIPYLHDLGITAVELMPVFQFDPHCNYWGYAPVSFFAPHQQYSLNEFRD
48893487_Trichodesmium_erythr       EKIPYLKELGITAVELLPVQQFDELINYWGYSQIAFFAPHHSYSMDEFRD
16329290_Synechocystis_sp.          EKIPYLKELGITAVELLPVHYFDPLTNYWGYSTIGFFAPHQGYSVDEFRD
                                        ::  :*:. :*::*        : :**      :    :    .

CM1405_Cyanidioschyzon_merolae      MVRELHRAGIAVILDIVFNHTAETWK--TYHFRALANREYYLHDHGNDVN
33635247_Prochlorococcus_marin      LVAACHDEGIEVILDVVYNHTTEGSIDGTLSWRGFSDALYYHQNDGEYLD
149177467_Planctomyces_maris        MVKALHRAGIEVIIDVVYNHTGEGNENGTYSFKGIDNTTYYSLTGSQFAN
32398065_Rhodopirellula_baltic      MVKALHAAGIEVILDVVYNHTCEGGHRGTYCWKGIDSSTAYMMTGAPYAN
33236242_Chlamydophila_pneumo       LVKTLHQEGIEVILDVVFNHTG---LQGTCSLPWIDTPSYYILDAGHFTN
42525620_Treponema_dentic           MVREFHKAGIEIILDVVFNHTAEGNENGVFSFKGFDNSIYYHLEDFYYKN
19714344_Fusobacterium_nuclea       LIFSLHKNGIEVILDVVYNHTAEGGTGGVYNFKAMGENIFYTKDRNYFTN
150017328_Clostridium_beijer        LIKKFHEDGIEVILDVVFNHTAEGNEYGYISFKGFDNNVYYMLTPGKYYN
48894542_Trichodesmium_erythr       LVKDLHKNGIEVILDVVFNHTAEGNENGTISFRGIDNKTYYMLTPGYYFN
16330244_Synechocystis_sp.          LVKELHKVGISVILDVVFNHTAEGNERGTISFRGLDNKTYYMLTPGYYFN
CM4050_Cyanidioschyzon_merolae      MVRELHRHGIEVILDVVFNHTGEFGDDGFYHFKGLALSTYYILDRNQFAN
22328517_Arabidopsis_thalia         MVKALHSAGIEVILDVVYNHTNEADDKYTTSFRGIDNKVYYMLDPNQLLN
15225595_Arabidopsis_thalia         LVKEAHKRGIEVIMDVVLNHTAEGNEKGIFSFRGVDNSVYYMLAPGEFYN
46446740_Parachlamydia_sp.          MVKELHRHGIEVILDVVYNHTFEGNQMGIQSFRGLDKHAYYMIDEGNYLN
116626210_Solibacter_usitatus       LVRELHRAGIQVILDVVYNHTAEGNEMGSMSFRGLDNVSYYCLTGRYYMN
48765636_Rhodospirillum_rubrum      MIDCLHGADIAVILDVVLNHSGEGDEYGTLSFRGLDNAAYYRLDPRRYLN
24373069_Shewanella_oneide          MVDALHGAGIEVILDVVYNHSAEGSRLGTFSFRGIDNLSYYRLHPRFYIN
1707700_Sulfolobus_solfat           MVNELHNAGIEVIIDVVYNHTAEGNHLGTLSFRGIDNTAYYMLQPRYYLD
48784580_Burkholderia_fungor        AVRQLHAAGIEVILDVVYNHTCEGNEMGTVSWRGLDNASYYRLIPRHHIN
17548459_Ralstonia_solana           AIRRLHAAGIEVLLDVVFNHTCEGNELGTLSFRGLDNASYYRLVPRYYIN
118048461_Chloroflexus_aggrega      MVKALHAAGIEVIIDVVYNHTGEGNHLGTLSFRGIDNASYYRLVPRFYLD
48763460_Rhodospirillum_rubrum      FVQKMHDAGLEVILDVVYNHTAEGNHLGTLSFKGIDNRSYYYLMGRHYNN
58582972_Xanthomonas_oryzae         MVKAMHKQGLEVILDVVYNHTAEGSELGTLSFKGIDNASYYRLAERYYIN
58579740_Xanthomonas_oryzae         MVARLHHAGLEVLLDVVYNHTAEGNELGTLSFKGIDNASYYRLADRFYIN
26990756_Pseudomonas_putida         MVAHLHDAGLEVILDVVYNHTAEGNERGTLSMRGIDNASYYRLMPRYYIN
48785916_Burkholderia_fungor        MVDRFHNANLEVILDVVYNHTAEGNERGTISFKGIDNASYYRLMPRYYIN
21223814_Streptomyces_coelic        MVRALHAAGIEVILDVVYNHTAEAGELGTLSLKGIDNRGYYRLQPRRYAD
45916433_Mesorhizobium_sp.          MVRKLHEAGIEVILDVVYNHTAEGNELGTLSFRGLDNASYYILGDRYYYD
6457937_Deinococcus_radiod          MVRALHDAGIEVILDVVYNHTAEGNHMGTMSFKGIDNPTYYRLVARFYFD
48894700_Trichodesmium_erythr       MVKALHNGGIEVILDVVYNHTGEGNHLGTLSFKGIDNAAYYRLVERYYMD
108803168_Rubrobacter_xylanoph      MVKRLHSAGLEVILDVVYNHTAEGNHLGTLSFRGIDNAAYYRLVPRYYMD
25028566_Corynebacterium_effic      MVRSFHEAGIEVILDVVYNHTAEGNHMGTISFRGIDNEAYYRLVDRHYMD
21224411_Streptomyces_coelic        AVKALHEAGIEVILDVVYNHTAEGNHLGTLSFKGLDNPSYYRLADRYYMD
21225613_Streptomyces_coelic        MVKTLHEAGLEVILDVVYNHTAEGNERGTLSFRGIDNASYYRLVDQHYYD
149179477_Planctomyces_maris        MVRALHKAGIEVILDVVFNHTAEGNENGTLSFRGLENQVYYHLDQKYYKN
32446352_Rhodopirellula_baltic      MVKALHSAGIEVILDVVFNHTCEGNEQGTLSFKGLENQVYYILSEQHYCN
150020446_Thermosipho_melanesi      FVKELHKNGFEVILDVVYNHTGEGNELGTLNFRGIDNEIYYMLNPRYYLN
48837599_Methanosarcina_barker      MVRALHKAGIEVVLDVVFNHTAEGDNLGVFSFKGIDNSIYYLLEPQYYSN
```

```
116622006_Solibacter_usitatus       MVKALHRAGIEVILDVVFNHTAEGDHRGTLSFRGFDNTAYYLLEPRSYSN
48893487_Trichodesmium_erythr       MVKALHKEGIEVILDVVFNHTAEGNENGTVSFRGLENSTYYILEKGYYSN
16329290_Synechocystis_sp.          MVKALHKAGIEVILDVVFNHTAEGNEKGTLSFRGIDNRTYYILDESSYSN
                                     :   *  .: :::*:* **:              .     *        :

CM1405_Cyanidioschyzon_merolae      FSGCGHTFACNSLAGIEVIHDCVRFWANDMQVDGFRFDLAACLLRSPAVI
33635247_Prochlorococcus_marin      VSGCGNSIAANRPIVRQLILESMRCWAIELGVDGFRFDLGIALSRGPPLF
149177467_Planctomyces_maris        YSGAGNTLHTANRAVRKMIVDSLQYWVKEMHVDGFRFDLASVLTRKPTTL
32398065_Rhodopirellula_baltic      HSGTGNTLHTANRAVRRMIVDSLRFWDSQMHVDGFRFDLASVFTRNPPIV
33236242_Chlamydophila_pneumo       YSGCGNSLKTSEIPVIKFILDCLRYWVTEMHVDGFRFDLAPVLARDSFMI
42525620_Treponema_dentic           FSGCGNTLNCNHKVVKDMIIQSLLYWYLEVGVDGFRFDLAPVLGRD-SLL
19714344_Fusobacterium_nuclea       FSGCGNTLNCNHKVVKDMIIQSLLYWYLEVGVDGFRFDLAPVLGRD-SLL
150017328_Clostridium_beijer        SSGCGNTLNCNHPIVHRMILDCLRYWVTEYRVDGFRFDLASILGRNPPLL
48894542_Trichodesmium_erythr       FSGCGNTINCNNPIVRNVVLDCLRYWASEYHIDGFRFDLASILGRDPPLL
16330244_Synechocystis_sp.          FSGTGNTLNCNNPIVRGMVLDCLRYWTAEFHIDGFRFDLASILGRDPPLL
CM4050_Cyanidioschyzon_merolae      YSGCGNSLSANHVVTAEFIHECVRYWALEMGVDGFRFDLAAVMCRDPPVI
22328517_Arabidopsis_thalia         FSGCGNTLNCNHPVVMELILDSLRHWVTEYHVDGFRFDLASVLCRAPPLI
15225595_Arabidopsis_thalia         YSGCGNTFNCNHPVVRQFILDCLRYWVTEMHVDGFRFDLGSIMSRSPPVI
46446740_Parachlamydia_sp.          FSGCGNTFNANHPIVKEFIIQSLRYWVTEMRVDGFRFDLASILCRSSPLI
116626210_Solibacter_usitatus       YTGCGNSMNFDSPAVIRLVMDSLRYWVEAMHVDGFRFDLASVLGRGSGFF
48765636_Rhodospirillum_rubrum      DSACGNTLRMEHPQVLRLAMDALRYWVGVMGVDGFRFDLAVTPARHGPFL
24373069_Shewanella_oneide          DTGCGNTLNLNHPRMLQLVLDSLRYWVKVMGVDGFRFDLAASLGRESGFF
1707700_Sulfolobus_solfat           FTGTGNTLNLSHPRVIQMVLDSLRYWVTEMHVDGFRFDLAAALARENTFF
48784580_Burkholderia_fungor        DTGCGNTVNLPHPRVLQMVMDSLRYWSTAFNMDGFRFDLGVTLGRESGFF
17548459_Ralstonia_solana           DTGCGNTLNLSHPRVLQMVMDALRYWVNACHIDGFRFDLGVTLGRESGFF
118048461_Chloroflexus_aggrega      DTGTGNSLNMNNPRALQIVLDSLRYWVTEMHVDGFRFDLARTLVRGSPFL
48763460_Rhodospirillum_rubrum      FTGTGNALELRHPRVLGMVMDSLRYWATAMGVDGFRFDLATTLARVAGFL
58582972_Xanthomonas_oryzae         DTGTGNTLNLSNSRVIQFVNDSLRYWAGEMHVDGFRFDLATILGREGGFL
58579740_Xanthomonas_oryzae         DTGTGNTFDLTNVGALRMVMDSLRYWVQEMHVDGFRFDLASILGREGSFL
26990756_Pseudomonas_putida         DSGTGNTLDLSHPCVLQLVTDSLRYWAGEMHVDGFRFDLATILGRYHGFL
48785916_Burkholderia_fungor        DTGTGNTLNLSHPRVLQMVTDSLRYWVTEMKVDGFRFDLATILGREGGFL
21223814_Streptomyces_coelic        YTGCGNTLHVVQPHVLRLITDSLRYWVTEMGVDGFRFDLAAALARSSPFL
45916433_Mesorhizobium_sp.          TTGCGNTLNLKHPRVLQMVMDSLRYWVEECHVDGFRFDLATALGRESVFF
6457937_Deinococcus_radiod          YTGTGNSLNVRHPQTLQLIMDSLRYWVTEMHVDGFRFDLASTLARGSGFF
48894700_Trichodesmium_erythr       FTGCGNSLNVRHPQILKLIMDSLRYWVTEMHVDGFRFDLASALAREAAFF
108803168_Rubrobacter_xylanoph      YTGTGNTLNMMHPRVLQLIMDSLRYWILEMHVDGFRFDLASALARESAFF
25028566_Corynebacterium_effic      YTGTGNSLNVRDPHSLQLIMDSLRYWVTEMHVDGFRFDLASTLAREATFF
21224411_Streptomyces_coelic        TTGTGNSLLMRSPHVLQMIMDSLRYWVTEMHVDGFRFDLAATLARQSSFF
21225613_Streptomyces_coelic        TTGTGNSLLMRHPYVLQLIMDSLRYWVTEMHVDGFRFDLAATLARQSAFF
149179477_Planctomyces_maris        YSGCGNAINGNHPVVREMIFHCLRHWTCNYHIDGFRFDLASILSRDPPLV
32446352_Rhodopirellula_baltic      YSGCGNTINGNHPVVREMIFHCLRHWVHNYHIDGFRFDLASILSRDPPMV
150020446_Thermosipho_melanesi      YSGCGNTLNCNHPVVKQLIIDSLRYWVTEMHVDGFRFDLAAVLGRTFSLL
48837599_Methanosarcina_barker      YSGCGNTVSCNHPISQKLIVDCLKYWAKEMHVDGFRFDEGSILSLDPPVI
116622006_Solibacter_usitatus       YSGTGNTLNANHPIVRRMIVDSLRYWVEEMHIDGFRFDLASILSRDPPVL
48893487_Trichodesmium_erythr       YTGCGNTFNTNNPFVHRLIVDCLCYWVREMHVDGFRFDLASVMSRSPPVL
16329290_Synechocystis_sp.          YSGCGNSVKANHPVVGGLILDSLRYWVSEMHVDGFRFDLASVLVRDANII
                                     :. *::.            . :   *     :****** .         .

CM1405_Cyanidioschyzon_merolae      QALKNDSALSDVLFIAEPWDLS-AYVVGHFAPSWSDWNGKYRDCVRKFIK
33635247_Prochlorococcus_marin      EEIEADPELSDLKLFSEPWDCGGLYRLSDFPHRIGTWNGHYRDDLRAFWK
149177467_Planctomyces_maris        GQIGSDSSLAGRRFIAEPWDAGGAFQLGSFPHRWMQWNAAYRDTLQKFVR
32398065_Rhodopirellula_baltic      SEIGTDSDLSDDRLIAEPWDADGQFQLGKFPQRWMQWNAHYRDTMQRFVR
33236242_Chlamydophila_pneumo       EAISFDPLLASTKIIAEPWDAGGLYQVGYFPPRWSEWNGPYRDNVKAFLN
42525620_Treponema_dentic           QAIADDSVLRSTKIIAEAWDAAGAYMVGKFPGRWAEWNDLFRNSVREFWL
19714344_Fusobacterium_nuclea       HELIEHPILSHAKLIAESWDLG-GYFVGAMPSGWCEWNGAYRDTVRQFIR
150017328_Clostridium_beijer        QSLAFDPILANTKLIAEAWDAGGLYQVGSFPKRWCEWNGKYRDDIRRFLK
48894542_Trichodesmium_erythr       ETLAFDPILANCKLIAEAWDAGGLYQVGSFPGRWAEWNGKYRDAIRKFIK
16330244_Synechocystis_sp.          ETLAFDPILARSKLIAEAWDAGGLYQVGSFPGRWAEWNGKYRDTVRKFIK
CM4050_Cyanidioschyzon_merolae      ERLSKDPCLRHLKLIAEPWDLG-QYLVGHFPGCWAEWNGRFRDTVRRFIK
22328517_Arabidopsis_thalia         RAIAKDSVLSRCKIIAEPWDCGGLYLVGKFPDRWAEWNGMYRDDVRRFIK
15225595_Arabidopsis_thalia         DMISNDPILRGVKLIAEAWDAGGLYQVGMFPGIWSEWNGKFRDVVRQFIK
46446740_Parachlamydia_sp.          EAISHDPILSQTKLIAEAWDAGGLYQVGGYPQRWAEWNGHYRDIVRRFIK
116626210_Solibacter_usitatus       DAVAQDPVLSRAILIAEPWDTG-TYQLGNFPIDWSEWNGKFRDTVRCFAK
48765636_Rhodospirillum_rubrum      SAIAQDPLLSRALVIAEPWDVGGGYQAGRFPKSWMEWNDLARDGLRRFWL
24373069_Shewanella_oneide          DALLQDPVLCRVKLIAEPWDIGGGYQVGNFPVAFSEWNDRYRDTMRRFWR
1707700_Sulfolobus_solfat           IALQQDPILSQVKLIAEPWDVGGGYQVGNFPYQWAEWNGKYRDSIRRFWR
48784580_Burkholderia_fungor        DALRQDPILSQRKLISEPWDIGGGYQLGNHPPGFGEWNDRFRDTVRRFWR
17548459_Ralstonia_solana           DAVRQDPVLATVKTIAEPWDVGDGYQLGNHPPGFAEWNDRFRDGVRRFWR
118048461_Chloroflexus_aggrega      TMVQQDPVLQRVKLIAEPWDVGDGYWVGRFPPPWAEWNGRYRDTVRRFWK
48763460_Rhodospirillum_rubrum      DAVAQDPVLSHVKLIAEPWDTGGGYQVGAFPPGWAEWNDQYRNTIRKFWK
58582972_Xanthomonas_oryzae         DACNQDPLLSEVKLIAEPWDCGGGYQVGHFPPGWSEWNDKFRDNAREFWK
58579740_Xanthomonas_oryzae         DAVRQDPVLSQTKLIAEPWDIGGGYQVGNFPPGWVEWNDKFRDNVRAFWR
26990756_Pseudomonas_putida         VACRQDPMLSQVKLIAEPWDCGGGYQVGNFAPGWAEWNDRFRDTARAFWK
```

```
48785916_Burkholderia_fungor        DSCRQDPVLSSVRLVAEPWDCGGGYQVGGFPPGWAEWNDRFRDTVREYWK
21223814_Streptomyces_coelic        AVIAQDPVLRRVKLIAEPWDVGGGYQVGAFPPLWTEWNDRYRNAVRDFWR
45916433_Mesorhizobium_sp.          DTVRQDPVLSRVKLIAEPWDLGDGYQLGNFPPGWGEWNGAYRDWVRSFWR
6457937_Deinococcus_radiod          TIIHQDPIISQVKLIAEPWDVGGGYQVGNFPVNWAEWNGIYRDDMRSFWK
48894700_Trichodesmium_erythr       DIVHQDPVISNVKLIAEPWDVGGGYQVGNFPLLWSEWNGKYRDTVRDFWR
108803168_Rubrobacter_xylanoph      DIIRQDPVISQVKLIAEPWDVGGGYQVGNFPVGWTEWNGKYRDAVRSYWK
25028566_Corynebacterium_effic      DLVQQDPVVSQVKLIAEPWDIGGGYQVGNFPPLWTEWNGKYRDTVRDFWR
21224411_Streptomyces_coelic        DLVQQDPVVSQVKLIAEPWDVGGGYQVGNFPPLWTEWNGKYRDTVRDLWR
21225613_Streptomyces_coelic        DLIQQDPVISRVKLIAEPWDVGGGYQVGNFPQLWSEWNGKYRDAVRDFWR
149179477_Planctomyces_maris        EAIAEDPLLADTKLIAEAWDAAGAYQVGSFSIRWAEWNGRYRDDIRRFWR
32446352_Rhodopirellula_baltic      ELIAEDPMLADTKIIAEAWDAAGAYQVGSFGHRWAEWNGRYRDDVRGFWR
150020446_Thermosipho_melanesi      KDISEDPILHNLKLIAEGWDAAGGYFLGEFPQGWAEWNGKYRDIVRKFVR
48837599_Methanosarcina_barker      WQIELDDALGYIKVIAEAWDAAALNQVGYFPPRWAEWNGYYRDEIRRFVR
116622006_Solibacter_usitatus       WDIECDAVLAGTKLIAEAWDAAGLYQVGSFIDSWREWNGRFRDDIRAFVR
48893487_Trichodesmium_erythr       WAIESDPVLAGTKIIAEAWDAAGLYQVGSFIDRFAEWNGHYRDDVRQFVK
16329290_Synechocystis_sp.          WAIESDPILAGTKLIAEAWDAAGLYSVGKFVDWFAEWNGPFRDDVRRFVK
                                         .  :     .:* **       .        **   *:  :

CM1405_Cyanidioschyzon_merolae      GDTGMIQEMATRICGSEDIYAYGINFITCHDGMSLRDLVSYNDAQNWANG
33635247_Prochlorococcus_marin      GDEDSAWRMGQRLSGSADLYGRSLNFITAHDGFTLNDLVSFNRKHNLANG
149177467_Planctomyces_maris        GDEGQIADLMTRLYGSSDLFYQSINYITSHDGFSLYDMVSFNQKRNWENG
32398065_Rhodopirellula_baltic      GDCGMVSDLMTRLYGSCDLFQLSVNYITSHDGSTLYDLTAYNEKRNWANG
33236242_Chlamydophila_pneumo       GDQNLIGTFASRISGSQDIYTNSINYVSCHDGFTLCDTVTYNHKHNEANG
42525620_Treponema_dentic           QPNPDIRHLATRVTGSADLYYQSINFVCCHDGFTLCDLLSYSEKHNEENG
19714344_Fusobacterium_nuclea       GDFGQVPELIKRIFGSVDIFQSSINFICCHDGFTMWDLVSYNLKHNILNG
150017328_Clostridium_beijer        GDSDLAATVAERITGSYDLYNASVNFITCHDGFTLYDLYSYNDKHNEENG
48894542_Trichodesmium_erythr       GDC-TVGEMAQRIQGSPDLYATSINFITAHDGFTLADLVSYNDKHNEANG
16330244_Synechocystis_sp.          GDAGVIGEMAQRLQGSPDLYSTSINFVTAHDGFTLADLVAYNGKHNYANG
CM4050_Cyanidioschyzon_merolae      GDPHLIGDFATRLCGSEDLYWHSINFVTAHDGFTLYDLVSYAEKHNWDNG
22328517_Arabidopsis_thalia         GDSGMKGSFATRVSGSSDLYYHGVNFVIAHDGFTLRDLVSYNFKHNEANG
15225595_Arabidopsis_thalia         GTDGFSGAFAECLCGSPNLYWHSINFICAHDGFTLADLVTYNNKNNLANG
46446740_Parachlamydia_sp.          GTSGHKTAFATALSGSQDLYCCSINFITAHDGFSLADLVTYNDKHNLDNG
116626210_Solibacter_usitatus       SDSGQLSQMGWRLTGSADLYYNSVNFVTCHDGFTLHDLVSYNGKHNEGNG
48765636_Rhodospirillum_rubrum      AN-GRSGDLAAVLAGCAARYRAGVSYISAHDGFTLADMVSYGHRHNNANG
24373069_Shewanella_oneide          GDHGMLPEFARRFHGSGDFFAASINFLTSHDGFTLKDLVSYCERHNWANG
1707700_Sulfolobus_solfat           GEALPYSEIANRLLGSPDIYFASINYVTSHDGFTLEDLVSYNQKHNEANG
48784580_Burkholderia_fungor        GDAGLRPDLAARLTGSADLFWASVNFVTSHDGFTLADVTAYEQKHNEANR
17548459_Ralstonia_solana           GDAGQRPELAARLTGSGDLFWASINYAASHDGFTLRDVVSYTHRHNEANG
118048461_Chloroflexus_aggrega      GDPGQAAEFASRFMGSMDLYYHSINFVTAHDGFTLRDLVSYNEKHNEANG
48763460_Rhodospirillum_rubrum      GDDGLLPTMAGRFSASSDVFWASVNFITAHDGFTLADLVSYNGKHNEANG
58582972_Xanthomonas_oryzae         GEDGKLAEFATRFTGSADLFWASVNFITAHDGFTLRDLVSYNEKHNIDNG
58579740_Xanthomonas_oryzae         GDGGQLAELATRLTGSADLFTASVNFVTAHDGFTLRDLVSYEGKHNLANG
26990756_Pseudomonas_putida         GDEGQLADFAARLTASGDMFYSSVNFITAHDGFTLRDLVSYNHKHNEDND
48785916_Burkholderia_fungor        GDEGKVPDLATRLTGSGDKFWASVNFIAAHDGFTLNDLVSYNDKHNEANG
21223814_Streptomyces_coelic        HALPDVREMGYRLSGSSDLYYASVNFVTAHDGFTLRDLVSYERKHNEANG
45916433_Mesorhizobium_sp.          GDERVAPDLASGLLGSAAMFWASVNFITAHDGFTLHDLYAYNERHNEANL
6457937_Deinococcus_radiod          GEGGLASEIGYRITGSSDLYYASINFVTAHDGFTLRDSVTYEQKHNEANG
48894700_Trichodesmium_erythr       GEKETLAEFAYRFTGSSDLYHASINFVTAHDGFTLNDLVSYNEKHNEANG
108803168_Rubrobacter_xylanoph      GDGGLVDELAYRLTGSSDLYYASINFVTAHDGFTLQDLVSYNEKHNEANG
25028566_Corynebacterium_effic      GEPATLGEFASRLTGSSDLYTASINFVTAHDGFTLNDLVSYNEKHNMANG
21224411_Streptomyces_coelic        GEPRTLAEFASRLTGSSDLYLASINFVTCHDGFTLHDMVAYNDKHNHANG
21225613_Streptomyces_coelic        AEDHSLGEFASRLTGSSDLYRASVNFVTAHDGFTLRDLVSYNDKHNEANG
149179477_Planctomyces_maris        GDVPTLGDYATRISGSSDLYFHGVNFITAHDGFTLNDLVSYEHKHNYANR
32446352_Rhodopirellula_baltic      GDAGTLGPLATRLAGSSDLYSCSVNLVTTHDGFTMNDLVSYKDKHNEANG
150020446_Thermosipho_melanesi      GDEGVIIELAKRITGSEDLYQASINFITCHDGFTMRDLVSYNEKHNEENG
48837599_Methanosarcina_barker      GDPGIVRRVASRIAGSPDLYINSVNFVTCHDGFTLNDLVSYNHKHNEANG
116622006_Solibacter_usitatus       GDDDTAARFADRWLGSQEIYEQSINFVTCHDGFTLNDLVSYNDKHNQQNG
48893487_Trichodesmium_erythr       SDPQIVEKLAARIMGSPDIYNHSINFITCHDGFTMNDLVSYNEKHNEANC
16329290_Synechocystis_sp.          GDNGAVPALASRLLGSPDIYNRSINFVTCHDGFTLVDLVSYNEKHNEANG
                                                  ..   :  .:.    *** :: *  ::   .*  *

CM1405_Cyanidioschyzon_merolae      EDNRDGEKHNLSWNCGVEGETDDENVLRLRRRQERNFFLILLCSARVPMI
33635247_Prochlorococcus_marin      ECNRDGENHNNSWNNGIEGPCSDHAVQALRHRQMRNLIATLLLSRGVPML
149177467_Planctomyces_maris        QHNEDGT-HDYSWNCGWEGSDAPEDILKLRKQQVKNFFTLLMVSNGTPMF
32398065_Rhodopirellula_baltic      NNNTDGA-LEYSWDCGWEGDGTPSEILQLRKQQVRNFFCLLMLSNGTPMF
33236242_Chlamydophila_pneumo       EDNRDGTDANYSYNFGTEGKTEDPGILEVRERQLRNFFLTLMVSQGIPMI
42525620_Treponema_dentic           ENNRDGSNENLSYNHGIEG-SASVEIERMRMRSAKNILTTLILSAGTPMI
19714344_Fusobacterium_nuclea       ENNQDGENNNHSYNHGEEGFTENPHIISLRKQQIKNMILILYISQGIPML
150017328_Clostridium_beijer        WNNTDGENNNNSWNCGAEGETDDENIIKLRRKLIKNACAVLLSSQGAPML
48894542_Trichodesmium_erythr       ENNNDGANDNESWNCGAEGWSDDPGINALRSRQMRNAIAIMMVSQGVPML
16330244_Synechocystis_sp.          ENGNDGANDNYSWNCGVEGPTDNPDILRLRARQMRNAIAILLVSQGVPML
CM4050_Cyanidioschyzon_merolae      EENQDGESHNNSWNCGFEGDTPNPNIFFLRQKQMRNFLVALLISTGTPMV
22328517_Arabidopsis_thalia         EGGNDGCNDNHSWNCGFEGETGDAHIKSLRTRQMKNFHLALMISQGTPMM
```

```
15225595_Arabidopsis_thalia         EENNDGENHNYSWNCGEEGDFASISVKRLRKRQMRNFFVSLMVSQGVPMI
46446740_Parachlamydia_sp.          EENRDGFDHNDSWNCGIEGHSNNKKIVALRERQIRNFLLALLVSQGIPMI
116626210_Solibacter_usitatus       ENNQDGTNDNNSWNCGAEGDTTDSGILSLRRQLMRNHACYLMFACGTPMM
48765636_Rhodospirillum_rubrum      ESNRDGSAIELSANHGVEGPTNDLAIRSLRDRQRRNLLACLLLSLGVPMI
24373069_Shewanella_oneide          EENRDGHHANFSYHYGVEGASNDVSVLVLRARQQRNLLTTLFLSQGVPML
1707700_Sulfolobus_solfat           FNNQDGMNENYSWNCGAEGPTNDQNVVICREKQKRNFMITLLVSQGTPMI
48784580_Burkholderia_fungor        EDNNDGHNENCSRNWGVEGESDDPAILATRKRVARSLIATLLMALGTPMV
17548459_Ralstonia_solana           EDNRDGHHDNCSANWGVEGPTDDAAILAQRARVARALLATVLLSNGTPML
118048461_Chloroflexus_aggrega      EENRDGDSHNNSWNCGVEGPTDDPDIRALRLRQMMNFIATLFLSQGTPMV
48763460_Rhodospirillum_rubrum      EDNRDGSDDNNSWNCGAEGPTDDEEINTLRRRQMRNMLATLLLSQGTPML
58582972_Xanthomonas_oryzae         EDNRDGSSNDGSCNYGEEGETDNAEILQIRERQMKNLLATLLLSQGAPMM
58579740_Xanthomonas_oryzae         EDGRDGSDHNISCNYGVEGETNDPTIKQLRRQQMRNLLATLLLSQGTPML
26990756_Pseudomonas_putida         ENNQDGTDNNLSWNCGVEGPTDDPAINALRMRQMRNYFATLLLAQGTPMI
48785916_Burkholderia_fungor        EDNKDGHSDNKSWNMGVEGPTDDPDIRQQRERQKRNLLATLLLSQGTPMI
21223814_Streptomyces_coelic        EGNRDGTDDNRSWNCGTEGETDDAQVRALRRRQLRNLLTTLLLSTGVPML
45916433_Mesorhizobium_sp.          EDNRDGHSDNRSWNCGAEGPTDDETIKALRAKMRRNAIATLLLSQGTPMV
6457937_Deinococcus_radiod          EGNNDGHNHNITWNCGVEGPTDDPEINRLRGQQMRNFLATLLLGQGTPMI
48894700_Trichodesmium_erythr       EDNNDGEKHNRSWNSGEEGETNDPEILSLRNRQRRNFLVTLMLSQGVPML
108803168_Rubrobacter_xylanoph      EDNRDGHDDNRSWNCGVEGPTGDRGIRRLRARQKRNLMATLLLSQGVPMI
25028566_Corynebacterium_effic      EDNRDGESHNRSWNCGVEGPTDDPEILQLRAQQRRNFLTTLLLSQGTPMI
21224411_Streptomyces_coelic        EDNRDGESHNRSWNCGVEGDTDDPAVLELRARQMRNFIATLLLSQGVPML
21225613_Streptomyces_coelic        EDNRDGESHNRSWNCGAEGGTKDPAVRELRGRQQRNFLATLLLSQGIPMI
149179477_Planctomyces_maris        EDNQDGENNNISMNFGVEGPTDDPAIIGMRERQIKNMLATLFLSQGVPML
32446352_Rhodopirellula_baltic      EDNNDGDNHNISDNYGVEGPTRKKAISTIRSQQVRNMLATLLTSQGVPMI
150020446_Thermosipho_melanesi      EDNRDGTNENFSYNHGVEGETDEPKIIKIRKQQVKNFITILMISHGTPMI
48837599_Methanosarcina_barker      ENNRDGIENNLSWNCGVEGETEDPEVETLRERQIKNFAAILLLSIGVPMI
116622006_Solibacter_usitatus       ESNRDGCNDNRSWNCGVEGPSNDPEIEALRNRQIKNFLTITLLSLGVPMI
48893487_Trichodesmium_erythr       EDNRDGANHNFSWNCGIEGTTDQEEIEMLRLRQIKNFFTILFFSQGTPMI
16329290_Synechocystis_sp.          EKNRDGTNDNFSWNCGVEGETDDPKINQLRLRQIKNFLTILFFSQGTPML
                                      . **   : : . * **      :   * :           .   **.

CM1405_Cyanidioschyzon_merolae      RSGDEIGHTQRGNSNVWCQDNELNYLHWLFRFVSTLVHFRKLTAPIWRRF
33635247_Prochlorococcus_marin      LMGDEVGRSQGGNNNSWCQDNPLGWMLWLHLFVSRLLMIRHQLSELFSPV
149177467_Planctomyces_maris        RMGDEFLQTQGGNNNPYNQDNETSWLDWMFRFVKLLIAFRKTHSSLCRSH
32398065_Rhodopirellula_baltic      RMGDEFLQTQRGNNNPYNQDNETSWLDWIFNFVREVISFRKSHPSISRSR
33236242_Chlamydophila_pneumo       QSGDEYAHTAEGNNNRWALDSNANYFLWLMHFLCDLIAFRKKYKTLFNRG
42525620_Treponema_dentic           NMGDEVFRTQNGNNNAYCQDNEMSWFDWLLEFTKKLINLRKTHFSFLRKH
19714344_Fusobacterium_nuclea       LMGDEMGRTQLGNNNAYCQDNPTTWVDWVFLFTKNMINLRKSYSIFKKET
150017328_Clostridium_beijer        LSGDEFGNTQFGNNNPYCQDNEISWLNWLFTFFKNMINFRKRHPAIKDRI
48894542_Trichodesmium_erythr       LMGDEIGRSKNGNNNTYCHDSELNWLNWLFRFVKNCVAFRKAHPILKNQN
16330244_Synechocystis_sp.          LMGDEMGKTQDGNNNTYCHDSPFNWLNWWFRFVKHCIAFRLAHPVLRNSE
CM4050_Cyanidioschyzon_merolae      LMGDEYGHTRRGNNNAWCQDNELNYMDWLVRFTRLMILFRRVNTFLCRQN
22328517_Arabidopsis_thalia         LMGDEYGHTRYGNNNSYGHDTSLNNFQWHFRFFSEVIKFRHSHHVLKHEN
15225595_Arabidopsis_thalia         YMGDEYGHTKGGNNNTYCHDNYMNYFRWFFRFCRILIKFRDECESLGLND
46446740_Parachlamydia_sp.          LMGDEYAHTRDGNNNTWCQDNKLNWFLWVFRFFKSLITFRKNYPLLKRDT
116626210_Solibacter_usitatus       LGGDEFARSQQGNNNAYCQDNAIGWFDWLVEFFRKAIAFTRRFPVLQRRK
48765636_Rhodospirillum_rubrum      RGGDELGQTQQGNNNVYCQDNPLNWLDWFAAFAARAGALRARLPQLGRAS
24373069_Shewanella_oneide          LSGDETGRTQGGNNNAYCQDNPMNWFDWLLSFTQQLIALRKRFPLLCAKR
1707700_Sulfolobus_solfat           LGGDELSRTQRGNNNAFCQDNEITWFDWFLEFVKKMIQFYRAHPAFRRER
48784580_Burkholderia_fungor        LAGDESLRTQHGNNNAYCQDNELSWLDWMTAFVARVIALRKQHPLLRETR
17548459_Ralstonia_solana           LGGDEFGRTQQGNNNAYCQDNAISWLDWLFAYTARLIALRKTSPALRWPH
118048461_Chloroflexus_aggrega      LAGDERGRTQQGNNNAYCQDNPISWVDWLLAFTRRLIAFRRQHPVLRRRR
48763460_Rhodospirillum_rubrum      LAGDEFANSQNGNNNAYCQDNALSWLDWQIAFVTSLLRLRREHVVFHRTR
58582972_Xanthomonas_oryzae         LSGDERGQTQGGNNNTYCQDNAITWLDWLTDFVKALTKLRKRYPILSRGR
58579740_Xanthomonas_oryzae         LAGDEFGHSQNGNNNAYCQDNELTWIDWQAAFVRRLIRIRQRYPLLHRAR
26990756_Pseudomonas_putida         VAGDEFSRTQHGNNNAYCQDSEIGWVNWLLAFVKRLTRLRLAYPVLRRSR
48785916_Burkholderia_fungor        LAGDEFGRTQKGNNNAYCQDNEISWVDWLIEFVRNLTTLRHRLPVLRRGR
21223814_Streptomyces_coelic        VAGDEMGRTQRGSNNAYCQDNETGWVDWLFALASRLIGLRHRHPVLRRRA
45916433_Mesorhizobium_sp.          LMGDEVGRTQNGNNNAYCQDNEINWLAWFLQFVRRLIAYRRAHPRLRLKN
6457937_Deinococcus_radiod          LGGDEFGRTQGGNNNAYCQDNDISWYDWLLAFTRKLIALRKAHPSLHRRK
48894700_Trichodesmium_erythr       LGGDEFGRTQKGNNNAYCQDNEISWVNWLLDFTRELIYFRFQHPVFYRRK
108803168_Rubrobacter_xylanoph      LHGDEMGRTQNGNNNAYCQDNETSWVSWFLAFVRRMIRLRREHPIFRRRS
25028566_Corynebacterium_effic      SHGDEMARTQNGNNNVYCQDNELAWINWLLSFTRRLLRIRAEHPVFRRRR
21224411_Streptomyces_coelic        SHGDEFARTQRGNNNAYCQDNELAWVAWLLEFTRAMVWLRKDHPVLRRRR
21225613_Streptomyces_coelic        CHGDELGRTQRGNNNAYCQDNEISWIDWLLDFARRLIALRADHPVLRRRR
149179477_Planctomyces_maris        LSGDECRRTQRGNNNTYCQDNAISWFDWLYRFCKELIHFRLCEPTLRQKN
32446352_Rhodopirellula_baltic      VSGDEARRTQKGNNNAYCQDTDISWFDWLVRFVSALIDFRKNQPTIRRRE
150020446_Thermosipho_melanesi      LMGDEIYRTQYGNNNAYCQDNEKTWLDWIFRFFKKMIEFRKKHHALRRRH
48837599_Methanosarcina_barker      CMGDEVRRTQKGNNNAYCQDNETSWFDWMFRFWKLMIDFRKRHTTILRPR
116622006_Solibacter_usitatus       VMGDEVRRTQQGNNNAYSQDNETSWFDWLHRFVKLLIARRLLRDIDSDRT
48893487_Trichodesmium_erythr       LMGDEVRRTQLGNNNSYCQNNKSSWFDWLLRFVKKIIHFTQELQITQLDK
16329290_Synechocystis_sp.          LMGDPVGRTQQGNNNGYCQDNELSWFDWETHFLRGIIALTQSLSLFLEES
                                      **   .:  *..* :  :.      *
```

```
CM1405_Cyanidioschyzon_merolae      LLYIAFNASHEACDITAPWAVLFNTAHMEPYSAVLMV
33635247_Prochlorococcus_marin      NMWMGFNAYSKAMHFDLPWHRILDTADLQNHSMLILL
149177467_Planctomyces_maris        FIYVMINAAANTCCFGIHWKCVVDTADVQARSVVVLI
32398065_Rhodopirellula_baltic      FIYVMINAGRDSVEFGIQWRLAIDTSNVNARSICVLV
33236242_Chlamydophila_pneumo       FVYVAFHVGAQDQLATLPYQIVAESQSLQPHTTLIAI
42525620_Treponema_dentic           FFYVMTNSYNNDITVRLPWHRLIDTSVVLSRTTVVLI
19714344_Fusobacterium_nuclea       PFYIAFNSYTEQLCFELPWYILTDTSCVLPKSSVILI
150017328_Clostridium_beijer        EVYLCINAHWETQYVRLPWRIAVNTSEIEPRSVMILV
48894542_Trichodesmium_erythr       HIYVAMNMHWDSLWFNIPWHIFVNTALLGDRSIVILV
16330244_Synechocystis_sp.          HIYVAMNMHYESLWFELPWHVFANTGLIGDRSVVILL
CM4050_Cyanidioschyzon_merolae      FLLVGFNAGPERRTVTVPWLRIVDTNVMQPWSSIILQ
22328517_Arabidopsis_thalia         FIYVAFNAHDYFVKALIPWFRVADTNNVAPFSSILLQ
15225595_Arabidopsis_thalia         FIYVAFNTSHLATLVSLPWEPFVDTSPMLSYSSIILL
46446740_Parachlamydia_sp.          FLYVAFNASHVVLTVTIPWVWLVNTHKIPSYTSIALK
116626210_Solibacter_usitatus       FLFFILNSDFDSQWVKLPWFRAIDTSIANARSTVVLL
48765636_Rhodospirillum_rubrum      FLLVLINGQPHEQSFRLPWTIALRSAVVPEMSLVALT
24373069_Shewanella_oneide          FLLLMVNADEHPLAFTPPWQCLLHTQLLQDRSLMLFH
1707700_Sulfolobus_solfat           YFLIIILNANPNNVKVKFPWELVISSYEIEGRTALVYR
48784580_Burkholderia_fungor        FLLMMLNANEKTLRFTPPWHVLLDTAKPGPQHGPRLL
17548459_Ralstonia_solana           FALTLINGAAADLIFTLPWLLAIDSARVQGRSVVLLL
118048461_Chloroflexus_aggrega      FLLVLVNAGNEEVPFILPWHVVIDTAHIKPRALAVLI
48763460_Rhodospirillum_rubrum      FFLLVLNASHTEVVFTLPWETVIDTMALPGRSLQVLR
58582972_Xanthomonas_oryzae         FLLIVINAYHEGVSFTLPWKLVLSTDLAPPRSVSVFE
58579740_Xanthomonas_oryzae         FLLMLINAASTSVSFTLPWRVLVDTAKASAHSLTLLA
26990756_Pseudomonas_putida         FVLLIVNAHHDIVPFKLPWSCLVDTDEVKGRSMLLMV
48785916_Burkholderia_fungor        FLLLVLNAHHDVVNFTLPWTCLLDTNQVTARSLLLLA
21223814_Streptomyces_coelic        FFLAVLHAGGEPAGFVLPYEVVVDTGTVPARTVLLLR
45916433_Mesorhizobium_sp.          FILILINGFHEPLGFSLPWKMIMRTDELADRSMVVLE
6457937_Deinococcus_radiod          FLLLLLSSSYVDLPFKMPWDLLLDTSTLRGRSVKLYR
48894700_Trichodesmium_erythr       WFMIFFNAHYELIEFTIPWVIIIDTSPVMEHSIVVLQ
108803168_Rubrobacter_xylanoph      FFLLLLNAHHEDLSFVLPWEAVVDTAPLKARSMAVLA
25028566_Corynebacterium_effic      FFILMFNAHHEAIDFTLPWKLLVDTTTVPARSSMLLR
21224411_Streptomyces_coelic        FFLLMFNAAPRPLDFVVPWEVVVDTVTLLDRSLTVLQ
21225613_Streptomyces_coelic        FFLLLLNSHWEPADFRLPWTALVDTATVEQRSLVLLS
149179477_Planctomyces_maris        FLMILVNSSPDPQAFELPWNLTIDTSVLPPRSLRCYV
32446352_Rhodopirellula_baltic      YVILMFNSTGDHREFHFPWNLFVDTAEVGRHSMRIYV
150020446_Thermosipho_melanesi      FIFVILNQWIEPLQFTLPWYRVVDTSIAQPKSSVVLI
48837599_Methanosarcina_barker      YIHVLMNMYWEPLEFEIPWYRAVDTFIVQGRSVVVLI
116622006_Solibacter_usitatus       TFHMIFNSWWQPLDFELPWRRWIDTAHAAPRSVVVLI
48893487_Trichodesmium_erythr       VFHIMLNAYWKPLMFELPWYLIVNTARLEPRAAVILM
16329290_Synechocystis_sp.          LLHLIFNAYWEPLNFELPWHRLVDTYWVQPRSSVVLM
                                                        :     :
```

Note: Top hits in GenBank searches (using *Cyanidioschyzon* and *Arabidopsis* sequences) are from chlamydiae and cyanobacteria. The *Arabidopsis* sequences are annotated as chloroplast precursors.

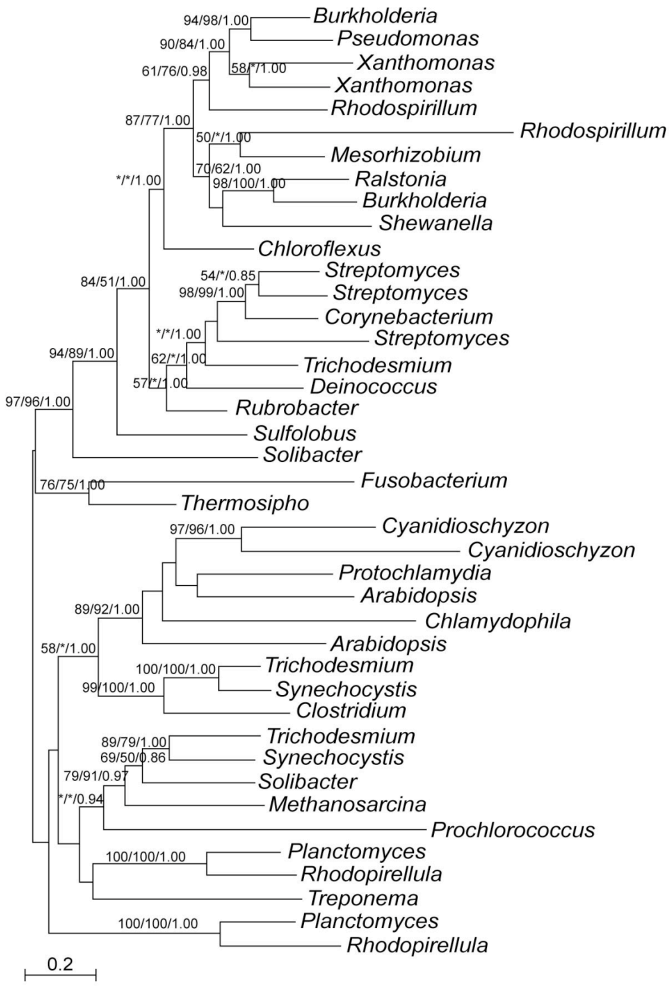

Figure 7. Molecular phylogeny of alpha amylase. P-value = 0.983 from AU test for the presented gene tree. AU tests were also performed on alternative topologies, including (**A**) monophyly of red algal and green plant sequences with different copies of cyanobacterial homologs, and (**B**) monophyly of red algal, green plants, cyanobacterial, and chlamydial sequences. These tests investigate if red algal and green plant sequences are plastid-derived and if red algae and green plants acquired the gene from plastids and subsequently spread to chlamydiae. P-values < 0.001 from AU tests for scenarios **A** whereas the P-value = 0.017 for scenario **B.**

---

8. 3-dehydroquinate synthase (D)

```
CLUSTAL X (1.83.1) multiple sequence alignment


45508724_Anabaena_variab           IELRLFPITITEPDKTIQTFERVIDVFADFKLVRKEPVLVVGGGLITDVV
KMO00293952_Karlodinium            IKFTKLVFSGNEADKDIRDVERILVALKEIGLARNEPLLVVGGGVIADIA
```

```
34498288_Chromobacterium_viola      IIGEIVPVPGGEACKDPQQLWRLLQALADRNIDRHSYALAIGGGAALDAV
ELO00198527_Euglena                 PSVMKYLVPCGDACKSWDVMSDLVQWLFASKATKRSVIVTWGGGAVLNVG
74023174_Rhodoferax_ferrir          NHPKVIVIEATEENKSIERVIPVIARLVANKARRGDTLVAIGGGIIQDIT
39937112_Rhodopseudomonas_palu      EAKRRIRIDALEANKSLEYAPHLISELRKAGADRSTHLVAIGGGIIQDVV
15836567_Chlamydophila_pneumo       YQVIVLTFPPGEPNKTWETFISLQYQLVDQNISPKSSIIGIGGGTVLDMT
60491665_Bacteroides_fragil         KDAVEITIGAEDVHKTLETLASVWQVLSEKGATRHSLLINLGGGMVTDLG
48430319_Picrophilus_torrid         FIKNRIIIDDGERAKSMEYLTLIINELLNKRVERGDSIIYIGGGTTGDLS
13542147_Thermoplasma_volcan        NSVVKISLNDGESLKSLRNYQKIVKVLLERKVDRRSLLVYIGGGTVGDLA
19714428_Fusobacterium_nuclea       DKVFYFAIKDGEEYKNIESILPVYDFMLENNFSRKSLIISLGGGVICDMG
57234788_Dehalococcoides_ethen      TETHLLAFPAGEASKTLSTLEDIGSKILDLRATKSSVIVCLGGGVVGNLG
18312973_Pyrobaculum_aeroph         PNAPALALKGGEGVKSLEALSKVYVFLQEAEADRGSTLVAVGGGALLDLA
65318882_Bacillus_anthra            QKVFSFVVPSGEKEKSFENFYAAHTSALENKLDRNSLIIALGGGMIGDLA
15922603_Sulfolobus_tokoda          KSFIEIPIIDGEGAKDIQYALKLVKLLFENGFDRGDYVIALGGGTVTDVV
18978063_Pyrococcus_furios          FKPQTIVVPDGEKYKDLDTVRYIWEKLLEMGFTRRSLLIGLGGGVITDIA
21674227_Chlorobium_tepidu          FRTIELVVPARETSKSVSTAWKLYGQMIEADVDRSWNLLCAGGGVVGDLG
57234699_Dehalococcoides_ethen      IGSLFLELPEGEIHKSLDMASHIYPQLINHFAERNTPILALGGGVIGDLS
45655651_Leptospira_interr          LPIKTIYIKGGEKSKHINRTGEVYNQLIEYGADRKSLILAFGGGVVGDFA
6458486_Deinococcus_radiod          QPHLTVTVPARDECKTLEVFADVLSQLAQAGLPRSGAVVGLGGGAATDLA
46399348_Parachlamydia_sp.          WNFSVLLIPEGETSKSLKQTTRCWRHFIKHQLDRYSLVVALGGGVICDLA
67928196_Solibacter_usitat          IPFEILNLPGGEDQKRLAPVEALAEEMVQRGADRSSMVIAYGGGIVTDMG
54025607_Nocardia_farcin            IDAHRVEIPDAEAGKDLAVAGFCWEVLGRIGLTRNDVVVSLGGGAATDLA
15827180_Mycobacterium_leprae       VDAHRIEIPDAEAGKDLSVMDFIWEVLGRIGIGRKDALVSFGGGAATDVA
67916807_Clostridium_thermo         YEVSKFVIPAGEENKNLDTTRDIYKYLLGLKLDRSATLMALGGGVVGDIT
39933580_Rhodopseudomonas_palu      IAHASVIVGEGESSKSYAGLEQVCEALIAAKIERNDLVIALGGGVIGDLA
48766569_Rhodospirillum_rubrum      IAHDHVVLPAGEATKSFSQLEELLDLLLAARFERSTTLLALGGGVIGDLV
CM1664_Cyanidioschyzon_merolae      DRVEECILPDGEEYKTMDSLRAIIDVCMQKRLDRKSTLFALGGGVIGDLT
74024228_Rhodoferax_ferrir          KHIHTVVLPDGEAFKTWQTLNLIFDALLSHGCDRKTILFALGGGVVGDMT
26249983_Escherichia_coli           VNVDSVILPDGEQYKSLAVLDTVFTALLQKPHGRDTTLVALGGGVVGDLT
22655384_Arabidopsis_thalia         VTVESVILPDGEKYKDMDTLMKVFDKAIESRLDRRCTFVALGGGVIGDMC
34496282_Chromobacterium_viola      VACSSVVLPDGEQHKDWQTLNLIFDALLSGNAERKTTLIALGGGVIGDMT
164848_Chlamydomonas_rein           LQVDEVVLPDGEEYKSMEVLGKVWDKALESRMDRGVTFLALGGGVVGDMT
67158019_Azotobacter_vinela         HELTPIVLPDGEAFKHWETLQKIFDGLLEARHDRRTTLIALGGGVVGDMA
15600231_Pseudomonas_aerugi         HEVTPVVLPDGEAYKQWETLQLIFDVLLKERHDRKTTLIALGGGVIGDMA
68179822_Desulfuromonas_acetox      YDVEQIVVEDGESYKNAETLNTIYTRLIELGCDRHSGIIALGGGVVGDMA
39984013_Geobacter_sulfur           FTVLRIDIPDGEEHKTSVTLTSVYDELIKGGLTRDSLLVALGGGVVGDLA
57223777_Cryptococcus_neofor        ARFLVYEVAPGEGAKSRKVKGEIEDWMLDNKCTRDTVILAFGGGVIGDLT
68466574_Candida_albica             SRLLTYCVSPGENNKNRATKAAVEDFLLQQGCTRDTVILAVGGGVIGDMI
BNO00088117_Bigelowiella            --------------------------------------------------
53796836_Chloroflexus_aurant        YRVHTTTVPSGEQSKSFGELNRLYDWMIEHGIERRDVVLALGGGVVGDLA
33240459_Prochlorococcus_marin      YDANLLILEAGENKKNYESIALIHNAAYEHQLDRGSLIIALGGGVIGDMA
46135112_Anabaena_variab            FQVASYCLPAGERYKTLNSIQKLYDIALENRLERSSTMVALGGGVIGDMT
16330660_Synechocystis_sp.          YEVFQHLIPAGETHKTLASINELYDVAFQANLERNSTLLSLGGGVIGDMT


45508724_Anabaena_variab            GFACSTYRRSSNYIRIPTTLIGLIDASVAIKVAVNHRKLKNRLGAYHASR
KMO00293952_Karlodinium             GFATSMYSRNTPYVMLCTSIVAGIDAGPSPRVCCNGFDYKNLYGSYHPPV
34498288_Chromobacterium_viola      GLAASLFHRGVRHIRLPSTVLSQADSGVGVKNAINWNGQKNLLGSFAVPW
ELO00198527_Euglena                 GLVASMVFRGMKYVQIPTTLLAMHDVTTSLKTSICFQGRKNNIGSYYAPI
74023174_Rhodoferax_ferrir          CFIASTLLRGMAWRFVPTTLLAQADSCIGSKSSINLGATKNILGTFNPPE
39937112_Rhodopseudomonas_palu      TFAASIFMRGLPWTYMPTTLLSMVDSCIGGKSSINAAGYKNLVGNFYPPK
15836567_Chlamydophila_pneumo       GFLAATYCRGLPLYLIPTTITAMVDTSIGGKNGINLRGIKNRLGTFYLPK
60491665_Bacteroides_fragil         GFAAATFKRGIAYINIPTTLLAMVDASVGGKTGINFNGLKNEIGAFAPAA
48430319_Picrophilus_torrid         GFAASIYKRGMGLIAVPTTLLAQVDSSIGGKNGINYMNIKNLIGTFYNPK
13542147_Thermoplasma_volcan        GFVASTYKRGVMMIAVPTTLLAQVDSSIGGKNGLDFSDVKNVIGTFYNPY
19714428_Fusobacterium_nuclea       GYISATYMRGIEFIQVPTSLLAQVDASVGGKVAINHPKCKNMIGSFKNPY
57234788_Dehalococcoides_ethen      GLTAALLFRGLRFFHIPTTMVAQIDSAIGQKQAVNYKMGKNLFGQYYAPE
18312973_Pyrobaculum_aeroph         TFAAGTYMRGIGLVQVPTTLLAMVDAALGGKGAVDWGLVKNLVGVFYQPK
65318882_Bacillus_anthra            GFVAASFMRGIRFVQVPTTLLAH-DSAVGGKVAINHPLGKNMIGAFHQPE
15922603_Sulfolobus_tokoda          GFVASIYMRGINLINIPTTLLGMVDAAIGGKTGVNFENVKNVLGTFYQPT
18978063_Pyrococcus_furios          GFVASTYMRGTYLGLVPTTLLAQVDAAIGGKTGINF-YGKNIIGTFYLPK
21674227_Chlorobium_tepidu          GYIAASYYRGIPVVQLPTTLLAMTDSSIGGKVAINHPLGKNLIGFFHMPE
57234699_Dehalococcoides_ethen      GFVAATYQRGVPLVHLPTSLLSQVDSSIGGKVAVNHGGIKNIVGSFYQPR
45655651_Leptospira_interr          GFIASTYLRGIRFVQIPTTLLACVDSSVGGKVAVNADFGKNMIGSFYQPE
6458486_Deinococcus_radiod          GFVAASYLRGVAFYTAPTTLLGMVDAAVGGKTGVNLPEGKNLVGAFWPPR
46399348_Parachlamydia_sp.          GFVASCYMRGIDTIYLPTTLLAMVDASIGGKTGINTSKSKNIIGSFHLPK
67928196_Solibacter_usitat          GFLAAIFMRGIPVLQIPTTLLAQVDAAIGGKTGVNLVSGKNLIGSFHQPL
54025607_Nocardia_farcin            GFVAATWMRGVRIVHVPTTLLAMVDAAVGGKTGINTEAGKNLVGCFHEPT
15827180_Mycobacterium_leprae       GFAAATWLRGVSIVHVPTTLLGMVDAAVGGKTGINTEAGKNLVGAFHQPL
67916807_Clostridium_thermo         GFAAATFLRGINFVQIPTTLLAQSDSSVGGKVGVDFEGTKNIIGAFYQPK
39933580_Rhodopseudomonas_palu      GFSASLLRRGVDFVQVPTSLLAQVDSSVGGKTGINSPQGKNLIGTFHQPV
48766569_Rhodospirillum_rubrum      GFAAAILLRGVDFIQIPTTLLAQVDSSVGGKTGINTAYGKNLVGAFHQPR
CM1664_Cyanidioschyzon_merolae      GFAASVFLRGIPFIQVPTTLLAVVDSAVGGKTGINHPLGKNMIGSFYQPH
74024228_Rhodoferax_ferrir          GFAAASYMRGVPFVQVPTTLLAQVDSSVGGKTGINHPLGKNMIGAFYQPL
```

```
26249983_Escherichia_coli           GFAAASYQRGVRFIQVPTTLLSQVDSSVGGKTAVNHPLGKNMIGAFYQPA
22655384_Arabidopsis_thalia         GYAAASYLRGVNFIQIPTTVMAQVDSSVGGKTGINHRLGKNLIGAFYQPQ
34496282_Chromobacterium_viola      GFAAACYQRGAPFIQIPTTLLAQVDSSVGGKTAINHPLGKNMIGAFYQPK
164848_Chlamydomonas_rein           GFAASCYQRGVHFVQVPTTVMAQVDSSVGGKTGVNHPLGKNMIGAFYQPR
67158019_Azotobacter_vinela         GFAAACYQRGVDFIQIPTTLLSQVDSSVGGKTGINHPLGKNMIGAFYQPK
15600231_Pseudomonas_aerugi         GFAAACYQRGVNFIQVPTTLLSQVDSSVGGKTGINHPLGKNMIGAFYQPQ
68179822_Desulfuromonas_acetox      GYAAATFLRGIPFVQIPTTVLAQVDSSVGGKTAINHPLGKNLIGAFYQPW
39984013_Geobacter_sulfur           GYAAATYLRGVPFVQVPTTLLAQVDSSVGGKTGINHPLGKNLIGAFHQPR
57223777_Cryptococcus_neofor        GFVAATFMRGVKFVQIPTTLLAMVDSSVGGKTAIDTPHGKNLIGAFWQPS
68466574_Candida_albica             GFVAATFMRGVRVVQVPTTLLAMVDSSVGGKTAIDTPLGKNFIGAFHQPE
BNO00088117_Bigelowiella            ----------------------MVDSSVGGKTAINVPAGKNLIGAFHQPR
53796836_Chloroflexus_aurant        GFAAATILRGVALVQIPSTLLAMVDAAVGGKTGINHPLGKNLIGAFHQPR
33240459_Prochlorococcus_marin      GFAAATWLRGIDFVQVPTTLLAMVDASVGGKTGVNHPKGKNLIGAFHQPK
46135112_Anabaena_variab            GFAAATWLRGINVVQVPTTLLAMVDSAIGGKTGVNHPHGKNLIGAFHQPR
16330660_Synechocystis_sp.          GFGAATWLRGINFVQVPTSLLAMVDASIGGKTGVNHPQGKNLIGAFYQPR
                                                            *   . :        **  * :  .

45508724_Anabaena_variab            KVFLDFSLLRTLPTDQVRNGMAELVKIAVVAHQEVFELLEAHRLTYKAIH
KMO00293952_Karlodinium             LTITDRGFWKSLHPGWLRHGVAEIIKMAVMKDLSLFNLMECDLIVGKAME
34498288_Chromobacterium_viola      AVINDAALIDGLPAREKRAGMAEAVKVALIRDASFFRWLELEQLIRRSAA
ELO00198527_Euglena                 LSLIDVAFCRTLPPAELFSGLGELAKFGAEHAEGFIDALTLEQLVKLGIK
74023174_Rhodoferax_ferrir          KIHLCSKFLDTLDQKDIYSGVGEILKVHAIAGKETFDLLALRRYIGSALR
39937112_Rhodopseudomonas_palu      KVLVDVSFISTLDAEMIAGGLFEAAKICYANYQAFLDYLAAVNVIRHSLL
15836567_Chlamydophila_pneumo       EVWMCPQFLSTLPREEWYHGIAEAIKHGFIADAYLWEFLNLHEFIKRNCQ
60491665_Bacteroides_fragil         SVLIETEFLRTLDAHNFFSGYAEMLKHGLISNTSHWAELLLKKLVGHSVQ
48430319_Picrophilus_torrid         LIIDDISFID--DKKLMMDGLAESLKMGITIEPELFNIIN--RIITLSIN
13542147_Thermoplasma_volcan        MVIDDTVFLKN-NSFIIREGMSEVIKYAIISGGDMYDTLN-TNIIKLSVK
19714428_Fusobacterium_nuclea       RVIIDVEFLKTLPKREFKSGMGELLKHSFLKDKSYLEYIELENIVEQSIR
57234788_Dehalococcoides_ethen      FVFIDFNFLRTLPERQIRAGLAESVKHGLCQESSFFDYIEIDYISRHTIE
18312973_Pyrobaculum_aeroph         AILCDLEWLRSLPPRVYRSAFAEVVKYGLALDEEFYSWLRLEEAVYRSLK
65318882_Bacillus_anthra            AVVYHTPFLQSLPEKEWRSGYAEVIKHALIGDVKLYHWLKLIHILMKAIP
15922603_Sulfolobus_tokoda          MIISDLNFLETLPLEEIKKGLAEVIKYGLVLDKDLYDYLALEEIIYKSSV
18978063_Pyrococcus_furios          FVLICRDFLKTLPMVEILNGLAEVVKYGIL-DKEVYNAIKREDIIKKSVA
21674227_Chlorobium_tepidu          LVLIDPAYLRTLPSREIYGGMSEVVKYGFIADREFFDLLTLSKAVSRSAF
57234699_Dehalococcoides_ethen      LVISDISCLKTLPEKEFACGMAEIIKSAAIGSSELFKQLEMEDIISQTAA
45655651_Leptospira_interr          FVFAPLSVLSTLPDREWKCGQAEIIKHSLLSGGEYWEKVKLPYLIAESVR
6458486_Deinococcus_radiod          AVWCDTATLTTLPDAVFREGAAEAFKHGLMADPSLLDRV-LEDTLADAIA
46399348_Parachlamydia_sp.          KILIDPFTLKTLSKKHYQAGFAEIIKYGMIASPSLFEFLELEIIIQQSCA
67928196_Solibacter_usitat          AVLTDPAILDSLPEREYRAGLWEIIKAGIIREVELFDYLSVDHIIAESVR
54025607_Nocardia_farcin            AVLVDLATLETVPRNEIVAGMAEIIKAGFIADPVILELVELPELIRRAIQ
15827180_Mycobacterium_leprae       AVLADLATLETLPRKEIASGMAEVVKAGFIADPIILDLIELPELIRRAVT
67916807_Clostridium_thermo         FVYINVNTLKTLPERELKAGLAEVVKHGVIMDEEFYEYIDLQYIAKRNCS
39933580_Rhodopseudomonas_palu      LVLADTAILDTLSPRQFRAGYAEVAKYGALGDEAFFAWLEREHAVATSCR
48766569_Rhodospirillum_rubrum      LVLADTTVLDTLPRRELLAGYGEVVKYGVIDDPAFFDWLERIHAVLTACR
CM1664_Cyanidioschyzon_merolae      AVIVDASTLKTLDPRQISAGIAEIIKYGLICDEEFFNWCEIQYAMERSCQ
74024228_Rhodoferax_ferrir          KVVCDLDTLKTLPARELSAGLAEVIKYGPIADLEFLAWIELAHAVKRSCE
26249983_Escherichia_coli           SVVVDLDCLKTLPPRELASGLAEVIKYGIILDGAFFNWLEMAYCIRRCCE
22655384_Arabidopsis_thalia         CVLVDTDTLNTLPDREMASGLAEVIKYGLIRDAEFFEWQELAFAIKRSCE
34496282_Chromobacterium_viola      AVIADMELLATLPDRELSAGLAEVIKYGLLGDAGFLAWLELQYAVKRSCE
164848_Chlamydomonas_rein           VVLVDTDTLATLPDRELASGISEVIKYGLIRDAPFFEWLEIAYAVERSCI
67158019_Azotobacter_vinela         AVVIDTATLATLPERELSAGLAEVIKYGLICDEPFLGWLELTEAIARSCA
15600231_Pseudomonas_aerugi         AVVIDTASLKTLPSRELSAGLAEVIKYGFICDEPFITWLEVTEAIERSCA
68179822_Desulfuromonas_acetox      AVFINVATLETLDQRNVLAGIAEVIKYGVMFDETFFSWLELAYAVRRSCE
39984013_Geobacter_sulfur           AVLIDVDTLATLPQREYLGGLAEVIKYGVVLDGKFFAFLELVRAITRCCA
57223777_Cryptococcus_neofor        YIFVDLAFLTTLPTREVSNGMAEVIKTAAIWKDDDFALLELLQVVSGSIY
68466574_Candida_albica             YVFCDVSFLETLPARQFINGMAEVVKTAAIWNEEEFTRLEKAELVKTSVR
BNO00088117_Bigelowiella            VVYADMLLLKTLTEKQVKEGLAEAVKMGCIRNAGLFDYMELAEVIYTAIK
53796836_Chloroflexus_aurant        LVLADTNLLATLPARELRAGWAEVIKHGVIRDAELFDALELTNIIARAVA
33240459_Prochlorococcus_marin      LVLIDINTLKTLPQREFRSGMAEIIKYGVIKDLELFNKLELLELIKISVS
46135112_Anabaena_variab            FVLIDPQVLKTLPVREFRAGMAEVIKYGVIWDAELFNQLEMDAILTRSCQ
16330660_Synechocystis_sp.          LVYIDPVVLKTLPEREFRAGMAEVIKYGVIWDSELFTALELTKIIQRSCQ
                                                       .  *  *

45508724_Anabaena_variab            KMLELEVPNLHELDLDRVIAYGHTWSPTLE--MFHGHAVNVDMAFSATIA
KMO00293952_Karlodinium             GYVRSEYGNLWETHQARPHAYGHTWCPGYE--MLHGQAVGTCMGYGAYLA
34498288_Chromobacterium_viola      LHLRQITQDPFEQGSARPLDYGHWIAHKLERLLNHGEAVAIGVACDARHS
ELO00198527_Euglena                 AKMTVLADDAYEKKFGMIFEYGHTVSHAIEKAIPHGLGVAYGMLCCSYVS
74023174_Rhodoferax_ferrir          IKQKYIELDEFDRGIRNIFNYGHSFGHAIESAVPHGIAVAMGMDMANAIA
39937112_Rhodopseudomonas_palu      TKKWFIEIDEFDQKERLLLNFGHTFGHALEAAISHGVGVGLGMLVAAQYA
15836567_Chlamydophila_pneumo       IKAAIVAEDPYDRSLRKILNFGHSIAHAIETLVNHGQAVSVGMMIETRIS
60491665_Bacteroides_fragil         VKEDIVEQDPFEHGIRKALNLGHTVGHAFESLVLHGYAVAWGIVCELYLS
48430319_Picrophilus_torrid         AKLSIVSKDFHDKHLRYVLNFGHTIGHALESYISHGEAVANGMIIEAYIS
13542147_Thermoplasma_volcan        IKSEIVNRDFYDRGIRSVLNLGHTIAHGIEGAISHGKAVATGMLVEAHIG
```

```
19714428_Fusobacterium_nuclea       IKKHYVDIDPFEKGERAFLNLGHTYAHALESFYTHGEAVSKGIIFDLELS
57234788_Dehalococcoides_ethen      LKLELLKIDPFEGKLDPQLELGHTIGHAVEIILLHGESIAIGMVAEAALS
18312973_Pyrobaculum_aeroph         LKASVVEADEFEEGIRQVLNVGHTVGHALERVLLHGEAVSLGIAAELRLS
65318882_Bacillus_anthra            VKANIVAQDETEKGVRAHLNFGHTLGHALEKEITHGDGVAVGMLFAIFLS
15922603_Sulfolobus_tokoda          DKFSVVKADERETGIRIVLNFGHTIGHAIEAGVPHGYAISVGMVCEAKMA
18978063_Pyrococcus_furios          VKLRIVEEDLKENGKRRVLNLGHTVGHAIEKIIKHGFAVSMGLVVEAKIG
21674227_Chlorobium_tepidu          IKANVVEKDFRETGLRATLNFGHTFAHGLEKMLRHGEAVTIGMVCALFLS
57234699_Dehalococcoides_ethen      IKAGIVCQDETDRGIRNILNFGHTLGHALESTQSHGAAVAIGMCFASRLS
45655651_Leptospira_interr          FKANVVSSDEKETGLRKILNLGHTTAHAIESVYSHGEAVAIGLVTALLLS
6458486_Deinococcus_radiod          VKAGVVTRDLTEQGERAFLNFGHTLAHALEAVIPHGEAVAYGMHYAARLS
46399348_Parachlamydia_sp.          IKKKYVEADFKDLGIRAQLNYGHTFGHVIEMMYLHGEAVSIGMSCAAYLS
67928196_Solibacter_usitat          MKAEVVSSDEREGDMRRILNFGHTFGHALEAELLHGEAVAWGMRAAIYLG
54025607_Nocardia_farcin            VKADVVAADLKESSLREILNYGHTLGHAIERRWRHGAAVAVGLVFAAELG
15827180_Mycobacterium_leprae       VKAGVVSADEKESELREILNYGHTLAHAIERRWRHGAAVSVGLVFAAELA
67916807_Clostridium_thermo         IKASVVEKDEKEGGLRAILNFGHTIGHAIETVLLHGECVSLGMVGAMRMA
39933580_Rhodopseudomonas_palu      AKAAIVARDERETGDRALLNLGHTFGHALEAALFHGEGVAIGMVLAAEFS
48766569_Rhodospirillum_rubrum      AKARVVAEDEREGGRRALLNLGHTFGHALEAELLHGEAVALGMVMALDLS
CM1664_Cyanidioschyzon_merolae      CKAAVVAADERESGYRAILNLGHTFGHAIEAAWLHGEAVAAGMVMAAEMS
74024228_Rhodoferax_ferrir          IKAWVVGQDEREAGLRAILNFGHTFGHAIEAGWLHGEAVGCGMVMAAHLS
26249983_Escherichia_coli           LKAEVVAADERETGLRALLNLGHTFGHAIEAEWLHGEAVAAGMVMAARTS
22655384_Arabidopsis_thalia         NKADVVSQDEKESGLRATLNLGHTFGHAIETGWLHGEAVAAGTVMAVDMS
34496282_Chromobacterium_viola      MKAAIVAEDEKENGVRALLNLGHTFGHAIEAGWLHGEAVAAGMVLAAAAS
164848_Chlamydomonas_rein           NKAEVVAADERESGVRATLNLGHTFGHAIETGWLHGEAVAAGTVMAADLS
67158019_Azotobacter_vinela         AKARVVGVDERESGVRATLNLGHTFGHAIETHWLHGEAVAAGSVMALEMS
15600231_Pseudomonas_aerugi         AKARVVGADERESGVRATLNLGHTFGHAIETQWLHGEAVGAGTVMALEMS
68179822_Desulfuromonas_acetox      LKAEVVAADERESSVRAVLNFGHTFGHAVEQLVLHGEAVAIGMLVAANVS
39984013_Geobacter_sulfur           LKAWVVEQDERETGLRAVLNYGHTFGHAVEALVLHGEAVAIGMVRAAVLA
57223777_Cryptococcus_neofor        VKAHIVTIDERETGLRNLVNFGHTIGHAIEAVL-HGECVSVGIVLEAEVA
68466574_Candida_albica             VKAGVVSSDEKEAGLRNLLNFGHTIGHAIEAVL-HGECVSIGMIKEAELS
BNO0088117_Bigelowiella             GKADVVAQDEKEKGLRSTLNFGHTIGHAVEALLYHGECVAIGMVHEMAVL
53796836_Chloroflexus_aurant        VKVDVVSRDEFERGERITLNYGHTIGHAIEQLLLHGECVAIGMDAAARIA
33240459_Prochlorococcus_marin      IKARIVEKDEKESGLRAILNYGHTFGHVIETLWLHGEAVSMGMVLIGQLA
46135112_Anabaena_variab            AKADVVGKDEKEGGLRAILNYGHTVGHAVESLLKHGEAVGIGMVAAGQIA
16330660_Synechocystis_sp.          AKVDVVSQDEKEAGLRAILNYGHTVGHGVESLINHGEAVAIGMEAAAKIA
                                            :  :         **      *    **  :

45508724_Anabaena_variab            ARRGYITIAERDRILGLMSRVGLDIDILWRGTESITLLRAAMPK
KMO00293952_Karlodinium             YVKGFIPKDEMHRILKVISDCEIMEDKWKSNLAIIEKLAAPVPK
34498288_Chromobacterium_viola      VLAGLLPEGGETRVWQLLRALGHGRLALLRGLDEFRELCVTLLA
ELO00198527_Euglena                 HRLGIMSAEDRHKHDALIQKWPPTANRILELGMRDSKVSEVLLK
74023174_Rhodoferax_ferrir          SGRGLTSIETYQRMHGVLKKN-IQTDALMTALMKDKKLVLILPI
39937112_Rhodopseudomonas_palu      KRETELTDAGKDHVAHLIEHVKVDLDLAMEKFEFDKKYRIVIPN
15836567_Chlamydophila_pneumo       LAEVMKTPQLIDQLERLLKRFNYSPENIIYTLGYDKKLKMIMIE
60491665_Bacteroides_fragil         HLKGFPKEKMRQTIQFIKDNYGFDCKQLYEFMQHDKKINFTLLK
48430319_Picrophilus_torrid         KCLGNA--DISNEIRSIIKRLGVDINRLLEYIKNDKKINIVAVN
13542147_Thermoplasma_volcan        EKYGNTNHEVIEAIRDLMKRYGIGPGNILRYISNDKKINMPVPS
19714428_Fusobacterium_nuclea       LLRGQIDKEYLERARNIFKLFDLPSDKFIPLMRKDKKIITILLD
57234788_Dehalococcoides_ethen      CEMGYMKPELAAKIERIFKKLGVPLEGVLEALRYDNKTDFFLLE
18312973_Pyrobaculum_aeroph         AELGYLREKYVEETKSLLKAFELSSEQAKGLIKYDKKVYLPLVI
65318882_Bacillus_anthra            EQV-YKVNLAYEEMKQWFLNYGLSVERLVGLMKQDKKIHMVLMQ
15922603_Sulfolobus_tokoda          EEVGYAEEGVVEDVTWILSQYEIDVKKAIDAITKDKKVMMPFPT
18978063_Pyrococcus_furios          EILGFDSGKVVE----ILTKFGFDPYTTLQAMKIDKKIVIVVPV
21674227_Chlorobium_tepidu          HRPGFLAEADLREGLALLARFRLDRDELVESMLSDKKLRFVLLD
57234699_Dehalococcoides_ethen      VKLGLCENETVLRLEKLIADFGIDPEKIIDAMHHDKKIRFILLK
45655651_Leptospira_interr          EQHSELDPVTTKETIESLKNYGLKSKQLAKHMLHDKKIRFVLLK
6458486_Deinococcus_radiod          HALGGADLTAHTR---AFLRWQLTFDALAPFIARDKKVRFVLLR
46399348_Parachlamydia_sp.          CQMGLTTQETMQRQDALCQQAQFPLTRFTYLMAKDKKINLILPE
67928196_Solibacter_usitat          ESTGYVSAEDSVDMLQMIEDYGIRAENLLARLVHDKKVHFVLPV
54025607_Nocardia_farcin            RLAGRLDDATADRHRTILEAVGYDADALLDAMQTDKKLRFVVLD
15827180_Mycobacterium_leprae       RVAGRLDDATAQRHHTILTSLGYDADALLEYMAGDKKLRFVILD
67916807_Clostridium_thermo         LYLEMIDEQSVNRVKNTLDKIGIDVDKVYNQMFYDKKLTFVLPR
39933580_Rhodopseudomonas_palu      AERGMMPATDAERLAKHLAEVGADADRLMALMAQDKKLTFILME
48766569_Rhodospirillum_rubrum      VRLGLCPPADAARLRAHLDHVGWNAERLLAAMDHDKKVTFVLAR
CM1664_Cyanidioschyzon_merolae      RRLGFIDEQVVRRIEAILTEAAMNVETFLTYMAMDKKLRLVLLE
74024228_Rhodoferax_ferrir          LRLGLIDAALVQRLTTLIQKAGSASDNYLELMRLDKKIKFVVID
26249983_Escherichia_coli           ERLGQFSSAETQRIITLLTRAGMSAQAYLPHMLRDKKMRLILPL
22655384_Arabidopsis_thalia         YRLGWIDESIVKRVNKILVRAKMTVSMFKSIMAVDKKLRLILLK
34496282_Chromobacterium_viola      AELGWIGRDEAERVRRLIAAAGMPTEQWLNLMSHDKKVRFVLLR
164848_Chlamydomonas_rein           YRLGWIDQSLHDRIVALMQRARMTTEQFRSLMAVDKKLRLILLR
67158019_Azotobacter_vinela         RRLGWLGEAERDRGIRLLQRAGMSADDFLGHMAVDKKLRLVLLR
15600231_Pseudomonas_aerugi         HRLGWLSAAERDRGIRLLRRAGMTAEDFMEHMAVDKKLRLVLLQ
68179822_Desulfuromonas_acetox      RLMELCQDSDVERLTRLLEAFGFSLEDYLAAMGRDKKLRFIVNV
39984013_Geobacter_sulfur           EARGHSSAGDTRRIRALVEALGFDADSYRDVLLRDKKLDFVLNR
```

```
57223777_Cryptococcus_neofor       RQLGILSQVAVGRLTRCLQAYGLSVDRLLDIMKIDKKKKIVLLS
68466574_Candida_albica            RYLGILPPVAVARLSKCLVAYGVEIDILLKKMAIDKKIRCVLLE
BNO00088117_Bigelowiella           ADMGHLDAKHVDRITECLKAYNLKVEDMMKKMKIDKK-------
53796836_Chloroflexus_aurant       VALGICPPALVERQRNLLAAYGLDRSAILTLIGRDKKVRWVLPT
33240459_Prochlorococcus_marin     LRKNLWNVDDALRQEKLLTKAGINNEDVLRTLKGDKKIRLIVPL
46135112_Anabaena_variab           VNLGLWQQADADRQNALIEKAGLDIEGIIEALQLDKKVRFVLPT
16330660_Synechocystis_sp.         HYLGLCDQSLGDRQRQLLLKTKLAVENLLASLLHDKKVRFILPT
```

Note: All top hits in GenBank searches (using *Cyanidioschyzon* and *Arabidopsis* sequences as queries) are from gamma-proteobacteria. Phylogenetic analyses show that red algal and green plant sequences group with beta and gamma-proteobacterial homologs with strong support. The *Arabidopsis* sequence (GenBank accession number AAM98284 and TAIR locus AT5G66120) is annotated as a chloroplast precursor. Sequences of *Euglena* and *Karlodinium* were obtained from TBestDB.

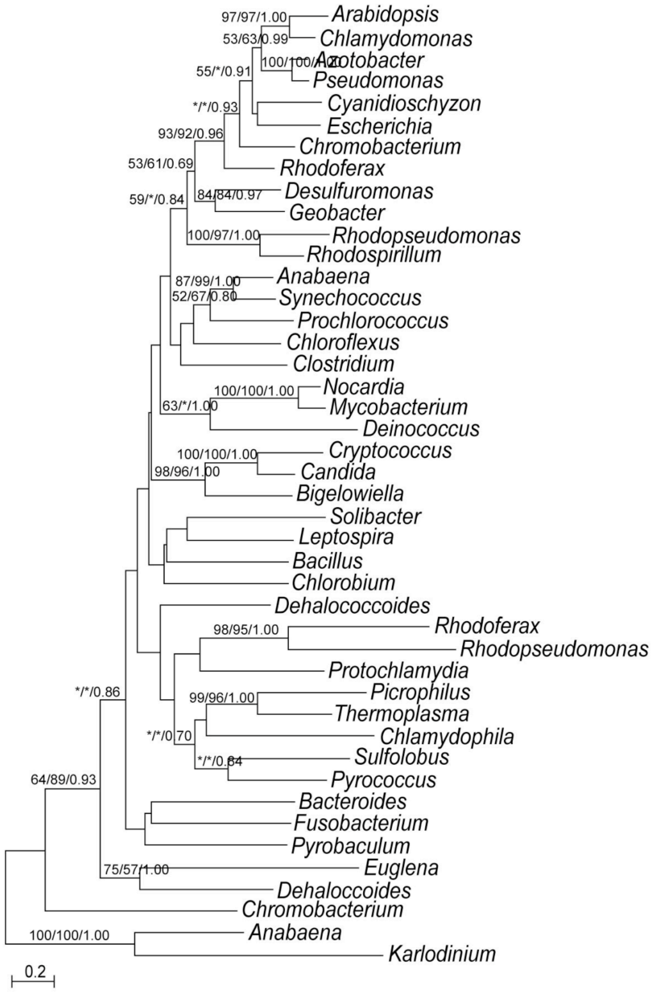

Figure 8. Molecular phylogeny of 3-dehydroquinate synthase. P-value = 0.997 from AU test on the presented tree. AU tests were also performed on alternative topologies, including (**A**) monophyly of all eukaryotic sequences (except for *Karlodinium*, which appears to be of cyanobacterial origin), (**B**) monophyly of cyanobacterial, green plant

and red algal sequences. These tests investigate if red algal and green plant sequences have the same origin (mitochondrial or eukaryotic) and if they acquired the gene from plastids. P-values < 0.001 from AU tests for both alternative topologies.

9-10. 2-methylthioadenine synthetase (E, D)

CLUSTAL X (1.83.1) multiple sequence alignment

```
24213838_Leptospira_interr         KFYITTLGCPKNIADSMSMHHSLLEEGFEESDFHFINTCTFIQSATEETI
111225093_Frankia_alni             RVALVTLGCSRNEVDSEELAGRLAAQGWADADAVLVNTCGFVDAAKKDSI
15835786_Chlamydophila_pneumo      KIHFISLGCSRNLVDSEVMLGILLKAGYEDADYLILNTCAFLKSARDEAK
76258351_Chloroflexus_aurant       KYHIVTLGCPKNAVDSEGMDGLLSTQGHEEADVIIVNTCSFIAAARAETL
116060729_Ostreococcus_tauri       RVSMVSLGCPKNTVDGEVILGDLHANGFEAADAIIINSCGFVEDAKNESV
CM3493_Cyanidioschyzon_merolae     RVAVVAMGCAKNTVDAEVFLGDLSAKGFEPADAIIVNTCTFIDEAKSESI
94417985_Pseudomonas_aerugi        KVGFVSLGCPKALVDSERILTQLRMEGYEDADVVVVNTCGFIDSAKAESL
94984483_Deinococcus_geothe        KVGFISLGCPKALVDSERILTQLRAEGYEDAQAVIVNTCGFITPAVEESL
119886282_Thermotoga_petrop        RVGIKVLGCPKNEADCEVLAGVLREGGHKDADVVVLDTCAFIEDAKRESI
116620691_Solibacter_usitat        KVGFISLGCPKNLVDSEVMMGQLVAKGHDQADVLVVNTCSFIDPAKKESV
118443956_Clostridium_novyi        KIGLISLGCDKNRIDSELLLGKLNEKN-NKADIIIVNTCGFIESSKQESI
32476670_Rhodopirellula_baltic     GRYVVSLGCPKNLVDTEQMLGRLDADGYDGADFVVVNTCGFIDSARDESM
108760706_Myxococcus_xanthu        SLYMMTLGCPKNRVDSEVMLGTLRHRGYSDAQVIVVNTCAFIGPAKQESV
51892771_Symbiobacterium_therm     KVGFISLGCAKNLVDTESMIGLLRNTGYEEADVLVVNTCGFIDAAKQESV
33862448_Prochlorococcus_marin     SVAFAHLGCEKNRVDTEHMLGLLTEAGYNDAAVVVVNTCSFIQDAREESV
16331757_Synechocystis_sp.         TIAINHLGCEKNRIDSEHMLGLLVEAGYELADYVIVNTCSFIQDARQESV
15606200_Aquifex_aeolic            KIGVVSLGCAKNLVDSEILLGKLKGAGVEEADVIIVNTCGFIEPAKLESI
21674219_Chlorobium_tepidu         AIFLLSLGCSKNTVDSERLTAQAVASGLDEADIILINTCGFIKDAKQESI
34540013_Porphyromonas_gingiv      RVDVITLGCSKNLVDSEVLMRQFLSNGYVCGEIVVVNTCGFIGDAQEESV
126662084_Flavobacteria_bacter     KINVITLGCSKNTYDSEVLMGQLKANG-EEGNIVVINTCGFIDNAKEESV
110637073_Cytophaga_hutchi         KVNIVTLGCSKNLVDSENLLTQLRGNGIDNSNVVVINTCGFIDNAKQESI
119885033_Thermotoga_petrop        TVRIETFGCKVNQYESEYMAEQLEKAGYGDVSYYIVNSCAVTKEVEKKVK
116622276_Solibacter_usitat        KFFVQNFGCRATQADGAALESLLAAKGLAGADLVVLNTCTVTASADDDVR
34580504_Rickettsia_sibiri         RQNVVTFGCRLNIYESEIIRKNLELSG--IDNVAIFNTCAVTKAAEKQAR
15836008_Chlamydophila_pneumo      GTFLVCLGCRVNQYEVQAYRDQLTILGYIPADLCIINTCAVTASAESSGR
24213088_Leptospira_interr         TVLFNTLGCRLNFFESDGLFASLSKHGYEHPEVVIINTCTVTNKADSKNR
66576257_Chlorobium_tepidu         SVAAVTLGCKVNYAETSSIVDALVSQGWDGADVLIIHTCAVTGEAERKSR
19704549_Fusobacterium_nuclea      KVAFHTLGCKVNQYETESIKNQLIKRGYDKSDIYIINSCTVTSIADRKTR
34541783_Porphyromonas_gingiv      KAAFLTLGCKLNFAETSTIGKALAEQGVEKADICVINTCSVTELADKKCR
126663485_Flavobacteria_bacter     KVAFYTLGCKLNFSETSTIARDFQNEGFEIADIYVINTCSVTDNADKQFK
110638137_Cytophaga_hutchi         KVAFYTLGCKLNFSETSTIGRMFTENGYEKADVYIINTCSVTDHADKKCK
32477342_Rhodopirellula_baltic     KLRTRTLGCKVNQYETELVRQGLQTIGYESADLCIVNTCTVTETGDAKSR
16079597_Bacillus_subtil           TVAFHTLGCKVNHYETEAIWQLFKEAGYQTADVYVINTCTVTNTGDKKSR
118444753_Clostridium_novyi        KVAFATLGCRVNMYESEAMAEKFIKNGYEMADVYVVNTCTVTNMSDKKSR
15605955_Aquifex_aeolic            KVAFETLGCRMNQFDTDLLKNKFIQKGYDMADVYVINTCTVTVGGDRSSR
32475869_Rhodopirellula_baltic     TVYIKTVGCQMNVLDSEMVIADLKRHGYGEADLLLYNTCSIREQAEEKTY
76261232_Chloroflexus_aurant       RYYVWTVGCQMNISDSERLEAALQGVGYEDASFIVLNSCSVRASAEERIL
94985469_Deinococcus_geothe        RAHLITYGCQMNEYDTHLVESQLVSFGADEADFVLINTCAVRGKPVDKVR
116625754_Solibacter_usitat        TFYIETFGCQMNAHDSEKVVGTLLAEGYEAAELVFYNTCSIRDKAEQKVF
66807387_Dictyostelium_discoi      KVWIETYGCQMNVSDEEVICSIMKSSGYNTADIVFLNTCSIRENAEAKIW
118402077_Tetrahymena_thermo       KYFIETYGCQMNESDTEIISGILQKAGFDNADIVFLNTCAIREGAENKIW
116061814_Ostreococcus_tauri       RVFVETYGCQMNANDSDVVRALLVEAKHSDATVVLVNTCAIRENAESRVW
CM1685_Cyanidioschyzon_merolae     KVFIETYGCAMNTSDSEIVASILAARPDDEADVVLINSCAIRDKPEVRVL
24213805_Leptospira_interr         KVYIETYGCQMNEYDSGIVSSLMRDAEYENSDIIFLNTCAIRENAHAKIY
Jakaba_bahamensis                  --------------------------------------------------
28872782_Homo_sapien               KVYLETYGCQMNVNDTEIAWSILQKSGYQEADVILLVTCSIREKAEQTIW
21674804_Chlorobium_tepidu         SFYIHTFGCQMNQADSEIVTALLRAEGFTNADIVLLNSCAVRENAEERLG
30690642_Arabidopsis_thalia        RIYHETYGCQMNINDMEIVLAIMKNSGYESAEVIFVNTCAIRENAEQRVW
34540755_Porphyromonas_gingiv      KLYIETYGCQMNVADSEVVASVMQMDGYDEADTILVNTCSVRDNAEQKVL
126663275_Flavobacteria_bacter     KLFIESYGCQMNFSDSEIVASILYENGYEDADLVLVNTCSIRDKAEQTIR
110638221_Cytophaga_hutchi         KLYIESYGCQMNFSDSEIVMSILTEEGFNNADVILLNTCSIRDNAEQKVR
119885210_Thermotoga_petrop        RFYIKTFGCQMNENDSETMAGLLMKEGFEEADVVIINTCAVRRKSEEKAY
108759982_Myxococcus_xanthu        RYFIHTFGCQMNVNDSLRMSEVLSQMSYDNADLIILNTCSIREKAEDKML
34581161_Rickettsia_sibiri         KLYIKTYGCQMNVYDSVKMQDLLYPFGYEEADVIILNTCHIREKAAEKTY
94417083_Pseudomonas_aerugi        KLFIETHGCQMNEYDSSRMADLLGEHQAAEADVILLNTCSIREKAQEKVF
CM4402_Cyanidioschyzon_merolae     RLYVQTFGCQMNLADSERMAGELERCGYYEADLILLNTCSIRDHAEQKVY
33862596_Prochlorococcus_marin     SFWIQTFGCQMNKADSERMAGILEAMGYLEADLVLYNTCTIRDNAEQKVY
16329745_Synechocystis_sp.         RYHIITFGCQMNKADSERMAGILENLGMNQADLVLYNTCSIRDNAEQKVY
111225071_Frankia_alni             RSYVRTFGCQMNVHDSERLSGLLESAGYSEADVVVFNTCAVRENADNRLY
```

```
19703810_Fusobacterium_nuclea       KASIITYGCQMNVNESAKIKKIFQNLGYDNADAVFLNTCTVREGAATQIF
15605821_Aquifex_aeolic             KFFIKTFGCQMNFNDSERIRGLLKTIGYEEADLIILNTCTIREKPDQKVL
118443818_Clostridium_novyi         RFFISTWGCQMNEEDSEKISGLLKGIGYDEADVVIFNTCCVRENAEQKVY
51892897_Symbiobacterium_therm      RVHIETFGCQMNEHDSEIMYGILAQMGYDDADLLLFNTCAVRESAVEHAF
16078764_Bacillus_subtil            KFYIRTYGCQMNEHDTEVMAGIFMALGYDDANVILLNTCAIRENAENKVF
71075619_Giardia_lambli             RVMMVTMGCGHNAAESDIIASALQTAGARDVDVLYINSCTVKNPSEDKAF
13542169_Thermoplasma_volcan        KIYFEAYGCTLNQGETALYVNKLLNEGNKEADLSIIGTCAVIKKTEDHMM
71029160_Theileria_parva            VVYLKNFGCSHNISDSEYMLGIISESGYDSCDLVIINSCTVKNPSEHGMI
71661088_Trypanosoma_cruzi          TVFIHTFGCSHNVSDGEYMAGLLAQAGYNDADVYLLNSCTVKNPSEEHFI
93277076_Homo_sapien                KIWIRTWGCSHNNSDGEYMAGQLAAYGYSDADLWLLNSCTVKNPAEDHFR
118373032_Tetrahymena_thermo        NVYVKTFGCSHNISDSEFMMGQLAEYGYKDAHLILVNSCTVKNPSQDAFM
116056662_Ostreococcus_tauri        KIFVHTFGCSHNHSDSEFMAGQLQSYGYSDADGWLVNTCTVKNPSQSAMN
18409989_Arabidopsis_thalia         TIYIKTFGCSHNQSDSEYMAGQLSAFGYEEADLWLINTCTVKSPSQSAMS
121522286_Methanococcus_maripa      KIYIEGYGCTLNTADTEIIKNSVNEFEDDDSDIIVINTCIVRQETEHRMI
18978284_Pyrococcus_furios          KVYIENYGCARNRADGEIMAGLLLSSGYENADIVVVNSCAVKDPTEVKIA
15920732_Sulfolobus_tokoda          KVYIETYGCALNKGDSYIMMTLLKDRGHNQADVIVLNTCAVRLETEERMK
119871859_Pyrobaculum_island        RIYVETYGCWLAKADAEILRQRL---GYDEADVILVYTCAVREDGEVRQL


24213838_Leptospira_interr          QTILSAAQQKLVVVGCFAERYPVDLFFGTGKYSQAGKILNYSKPYAYVKV
111225093_Frankia_alni              DALLAADDRAVVAVGCLAERYGADAVLGFDAYPSIGEHLLTAGPVAALKI
15835786_Chlamydophila_pneumo       DYLDHLIDAKIIVTGCMTSNHKIHYLLGSGDVENILSAISTPKHYAYLKV
76258351_Chloroflexus_aurant        GVLKELAGQRLIAAGCMAQSHPVDATLGTQQWTQINALVTHQTPSAYLKI
116060729_Ostreococcus_tauri        EAILEAAKTKIIVTGCLAQRYAADVIVGFENYGNLPRTVITPRHYAYLRV
CM3493_Cyanidioschyzon_merolae      SAILEAAARALVVTGCMAQRYAVDAVIGFAAYNQLAEQILGASHTAYLRV
94417985_Pseudomonas_aerugi         EVIGEAIAGKVIVTGCMGVEE-VLAVTGPQQYEQVVTAVLTPRHYAYLKI
94984483_Deinococcus_geothe         SAIGEALDGKVIVTGCLGER-PVAAITGSEAVDDVMAHVLTPRHYAYVKI
119886282_Thermotoga_petrop         DEIFSFVDYKLVVKGCLVQRYYVDQWIGVADPEEIANAILEERPYAYVKI
116620691_Solibacter_usitat         DTILEMAEKKLIVAGCLVERYRVDALIGTNELDSIVDICATPRHFAYMKI
118443956_Clostridium_novyi         DTILEMAKKMIIATGCLTQRYSIDIMLGVNDYANIQNYITTAKHMAYIRI
32476670_Rhodopirellula_baltic      AAIDEMLARNVVVTGCLAERQQIDALVGVFGRNDIVSVVVTPRHFAYLKI
108760706_Myxococcus_xanthu         DSILEMAEKTLVVTGCLSQRYGVDHFLGTSAYAQIGDLLSMPKYTAYLKI
51892771_Symbiobacterium_therm      DAILEAAQQALVVAGCMVPRYGIDALVGTADYPRIGEVVATPGYTAYLKI
33862448_Prochlorococcus_marin      RTLIGLAEKELIIAGCLAQHFQAKAIVGTGDYQHIVDVLTTDQAVAYLKV
16331757_Synechocystis_sp.          RTLVELAEKKIVISGCLAQHFQAVAVVGTGDYQNIVDIITTNEAIAYLRV
15606200_Aquifex_aeolic             ETILEFAEKEVIVMGCLVERYKVKAYFGTESWNEILNYLSTPRSYAYLKI
21674219_Chlorobium_tepidu          DETLAAIGREVYVMGCLVELYRIDGLFGTRELPEVLAAILTPPHYAFLKI
34540013_Porphyromonas_gingiv       NTILEMVEGSLYVMGCLSERFRVDAYYGKFDWKQLISHLTTPRHYAYLKI
126662084_Flavobacteria_bacter      NTILEYVDDKVFVTGCLSERYRVDQYFGTTELPLLLKALTTPKNYAYLKI
110637073_Cytophaga_hutchi          DTILRYIDDKIYVSGCLSQRYKVDAFFGSNELPAILKKFTTPSHYAYVKI
119885033_Thermotoga_petrop         RLIKSIRNAKIILTGCFAQLSPVDMVLGIDEKKHIVDHIFEDRTRSYIKV
116622276_Solibacter_usitat         QTIRRVHRARILVTGCYAQRAPVEWVVGNSHKTQIAEIVAGDRTRPNLKI
34580504_Rickettsia_sibiri          QAIRKAKKLKIIVTGCSAQTSPVDKVIGNEEKL-LPNYYFDCKSRAFIQV
15836008_Chlamydophila_pneumo       HAVRQLCRAHIVVTGCLGESDKQCTLVSNKEKSRLIEKIFEGKSRAFIKV
24213088_Leptospira_interr          NTIRNAIKSQIWVTGCYAETDRVAGVVGNTEKSKLPAMIPNGHTRAYLKI
66576257_Chlorobium_tepidu          QQIRKIIRSRVGVIGCYAQLDPVSFVLGTTDKFEIAWYDEKGRTRAFLKI
19704549_Fusobacterium_nuclea       NMLRRAKKAKVIVTGCYAQTNSVDFVIDNKNKSNIVNFVLREMTRAYVKI
34541783_Porphyromonas_gingiv       NAIRKLHKALMIVTGCYAQLKPVDIVLGADEKLDLVSILADDRTRHFLKV
126663485_Flavobacteria_bacter      QIVKKAMKAFVAAVGCYAQLKPVDLVLGATEKFKITDYIFGDRTRAFLKV
110638137_Cytophaga_hutchi          KVVKEALRAFVGIIGCYAQLKPVDAVLGASEKFKLFELLVNDRTRTFLKV
32477342_Rhodopirellula_baltic      QVVRRLNRARIVVMGCYATRAPVEVLTDKRELGDLMGRFFAGRKRAYVKV
16079597_Bacillus_subtil            QVIRRAIRGVICVTGCYAQTSPVDIVVGTQDREKMLGYIFTDRTRASLKI
118444753_Clostridium_novyi         QMLSRAKRSIIAAVGCYTQIAPVDVILGTRNKGDIVYWVYQDKTRAFLKI
15605955_Aquifex_aeolic             QAIYQAKRAIVVATGCYAQVNPVDLVVGNTHKSELLKILYFEGVRPFLKV
32475869_Rhodopirellula_baltic      SALGKLKEKTIGVMGCMAQKDQVDMVVGPGQLHAIPDMLRPTPFQAYLRI
76261232_Chloroflexus_aurant        GKLGELVRTRIVLWGCMVGPNNVDHFVSPSAVDEVVAL-SHPPVSVHVPI
94985469_Deinococcus_geothe         SLLGDLRKLVVGMMGCLAQLEEVDVLLGPGSLLDIGKALPQGKLQAHLTI
116625754_Solibacter_usitat         NRLQNFKRKIFGVLGCVAQQEGVSLVAGSASYTRLPEMLRDNPHRAYLTI
66807387_Dictyostelium_discoi       LRLTELRALIVGVLGCMAERLKVDIVVGPDAYRSLPSLLSDNQVSAYVSI
118402077_Tetrahymena_thermo        KRLENIRALITGVLGCMAERLKVDIIVGPDAYRDLPRLINPDSCQAFVSI
116061814_Ostreococcus_tauri        TRLRQLRARAVGVLGCMAERLKADMVVGPDAYRDVVRLLDPMSPQAYVSV
CM1685_Cyanidioschyzon_merolae      QRLSMLRARILGLLGCMAERLKVDLVVGPDSYRDLPLLLDHGLITAYVSI
24213805_Leptospira_interr          NRLQSLGYLVIGVLGCMAQNLGLDLVVGPDNYRSLPELIVVNGIQAFVTI
Jakaba_bahamensis                   --------------------------------------------------
28872782_Homo_sapien                NRLHQLKALRIGILGCMAERLKVDILAGPDAYRDLPRLLSASATSAFVSI
21674804_Chlorobium_tepidu          NILMHLKGLVIGVLGCVPQFERVDFIVGPDNYRELAGLVRAGSISAFLPV
30690642_Arabidopsis_thalia         QRLNYFWFPKVVVLGCMAERLKVDVVCGPDAYRDLPRLLSENSITAFVSV
34540755_Porphyromonas_gingiv       NRLAYYHSLVIGVLGCMAERVKVDVVAGPDSYLDLPNLVGGVHINGFVSI
126663275_Flavobacteria_bacter      KRLEKYNAMKVGVLGCMAERLKVDMVVGPDAYKDLPNLLNSNGVNAFVSI
110638221_Cytophaga_hutchi          HRLVHFKGMIVGVLGCMAERLKVDMVVGPDAYRDLPNLISGDGVSAFVSI
119885210_Thermotoga_petrop         SELGQMLKLVVGVAGCVAEKERADFVLGTRAVLKVTEAVRSSKHHAWVTI
108759982_Myxococcus_xanthu         SALGRYKPALIGVGGCVAQQEKLDFVFGPDNIARLPDIISRGKVTEFVTV
34581161_Rickettsia_sibiri          SELGRIKKAIIVVAGCVAQAEGVDIVVGPQSYYNLPELIYPQGTSAFISV
```

```
94417083_Pseudomonas_aerugi        SKLGMWRDLVIGVGGCVASQEGVDVVFGPQTLHRLPEMIRVDGPTAFVSV
CM4402_Cyanidioschyzon_merolae     SFLGPFVRLKLIVAGCVAQQEGLDLVMGPQYANRLADLLRQSQVCAWVNI
33862596_Prochlorococcus_marin     SYLGRQARLKLVVAGCVAQQEGLDLVMGPQHANRLEALLRDSTICAWVNV
16329745_Synechocystis_sp.         SYLGRQAKLTLVVAGCVAQQEGLDLVMGPQHANRLDQLLRESTVSAWVNI
111225071_Frankia_alni             GNLGQLVPMQIAVGGCLAQKDRVDVVFGTHNLHRLPVLLRASHHSAWVSI
19703810_Fusobacterium_nuclea      GKLGELKATIIGVTGCFAQEQGIDIVMGNQNIGRIPQAIFGSDQTASISI
15605821_Aquifex_aeolic            SHLGEYKKALIAVAGCLAQRTGIDIMFSSFNMHQLPELIRDNKYCAYVTI
118443818_Clostridium_novyi        GHLGELKALILIVTGCMMQQKGVDIIAGTYNSYKLPEYIRKSDIKAFVTI
51892897_Symbiobacterium_therm     GRIGQLKPLIIGVCGCVPQVEGLDLIFGTHNIHRLPELVREGDLKAWVTI
16078764_Bacillus_subtil           GELGHLKALILGVCGCMSQEESVDMIFGTHNIHRLPELLRNGKIKGWVNI
71075619_Giardia_lambli            VHVQKGLETVVVLGGCVPQSYGISGVLTQQWLAALPRLLRANPIIDIIST
13542169_Thermoplasma_volcan       ARIQNLSRGKVEVIGCLAPVKGNVSVIEKDRFRSFQEVIVNAEIVSGIPI
71029160_Theileria_parva           NYINQGLKKKIIVTGCIPQS--NISLLGIMQIEKIVYVIRKNKLIEIIPI
71661088_Trypanosoma_cruzi         SMMNRVRAKPLVVAGCVPQADPDVSVIGVRNIDRVGEVIRRNRFIEIIPI
93277076_Homo_sapien               NSIKKAQEKKIVLAGCVPQAQPGLSIIGVQQIDRVVEVVRKNPLIEIISI
118373032_Tetrahymena_thermo       TIVKTYKHKPIVVAGCVPQGDRDVSVIGISQIDRVVEVVKTLPSLDLPKI
116056662_Ostreococcus_tauri       TVLERGKAKALLVAGCVPQGDKDVSLLGVTQIDRVVEAMRRNEFVEILPL
18409989_Arabidopsis_thalia        TLITRGRSKPLVIAGCVPQGSRGVSVVGVQQIDRVVEIVRRNNFIEILPI
121522286_Methanococcus_maripa     SRIEYFKSKKVVVAGCMAKALPADVLVMPREAQYSGKILSSQGLITALPI
18978284_Pyrococcus_furios         RRIRELLDKKVIVTGCLPHVNPVSAVLGVKSIDRIVQAVSPRGVHFILPI
15920732_Sulfolobus_tokoda         QRIKELKKKKLIIAGCLVSSQPESSIVGAQSIDKIVEAVIFEGKISIIPI
119871859_Pyrobaculum_island       ARIRELVKKELIVAGCLARLRPAKLIYPSE---------YERGLIYTVPL


24213838_Leptospira_interr         SDGCNRGCSFCIIPSFRGKFVESPLDDILRDTNRAIRAGAKEICLVSQDT
111225093_Frankia_alni             SSGCDRRCAFCAIPSFRGSHVSRPADDVLAEAEWLAGEGARELVLVSENS
15835786_Chlamydophila_pneumo      AEGCRKRCAFCIIPSIKGKLRSKPLDQILKEFRILVNKSVKEIILIAQDL
76258351_Chloroflexus_aurant       SDGCNLRCAFCTIPSFKGDMRSKPVGAVLAEAQELVAGGVREIVLVAQHL
116060729_Ostreococcus_tauri       AEGCDHKCTFCAIPSFRGRFRSKPFGAIVDEAKALADSGVRELNLIAEDT
CM3493_Cyanidioschyzon_merolae     AEGCDHQCAFCAIPQWRGRFRSKPFDQLVEEAHHLVERGVRELCLIAEDT
94417985_Pseudomonas_aerugi        SEGCNHSCSFCIIPSMRGKLVSRPVGDVLSEAERLVKAGVKELLVISQDT
94984483_Deinococcus_geothe        AEGCNHTCSFCIIPKLRGRQVSRDAGAVLYEAYRLIAGGTKELMIISQDT
119886282_Thermotoga_petrop        SDGCDRGCTFCSIPSFKGSLRSRSIEDITREVEDLLKEGKKEIILVAQDT
116620691_Solibacter_usitat        AEGCDHPCTFCVIPQYRGAFRSRRFESVVSEATRLFQQGIREINLIGQDT
118443956_Clostridium_novyi        SEGCNNLCTYCIIPKIRGKYRSRSIESIINEAKELANMGVKELILVGQDT
32476670_Rhodopirellula_baltic     SEGCDRLCTFCAIPKMRGKHFSKPIEQIIDEAKRLGDSGVREVVIVAQDT
108760706_Myxococcus_xanthu        SEGCDNACAFCIIPTLRGGQRSRPIDDIVAEAKQLADSGVQELNLVAQDL
51892771_Symbiobacterium_therm     AEGCDCACAFCSIPLMRGRHRSRPIESIVDEARRLAGMGVRELVVISQDT
33862448_Prochlorococcus_marin     AEGCDYRCAFCIIPKLRGDQRSRPVESIVTEAHQLAEQGVQELILISQIT
16331757_Synechocystis_sp.         AEGCDYRCAFCIIPQLRGKQRSRPIESIVAEAEQLASQGVKELILISQIT
15606200_Aquifex_aeolic            AEGCNRLCSFCAIPKIRGRHRSRKIEEIVDEAKFLADQGVKEICVVSQDT
21674219_Chlorobium_tepidu         AEGCNRRCSFCSIPKIRGPYVSQPIEQLLREAALLQQQGVKELNLIAQDI
34540013_Porphyromonas_gingiv      SEGCDRSCSYCAIPIITGRHRSRPMEDLVEEVRMLVKHGTREFQLIAQDL
126662084_Flavobacteria_bacter     AEGCDRPCSFCAIPLMRGKHVSQPIEKLVKEAEGLAKKGVKELILIAQDL
110637073_Cytophaga_hutchi         AEGCDRPCSFCAIPVMRGKHVSTPMEDLVKQAKGMAAKGTKELILIAQDL
119885033_Thermotoga_petrop        EDGCDNTCTYCAIRLARGKIRSKPLEIFKDEFVEMVAKGYKEIVITGVNL
116622276_Solibacter_usitat        QDGCNNRCSFCIIPFVRGRSRSAPAAQVVEQVRALAAR-YHEVVLSGINL
34580504_Rickettsia_sibiri         QNGCDHFCTFCIIPYGRGKSRSVAIGAIAEQVKHLVLNGFKEVVFTGVDV
15836008_Chlamydophila_pneumo      QDGCNSFCSYCIIPYLRGRSVSRPAEKILAEIAGVVDQGYREVVIAGINV
24213088_Leptospira_interr         QDGCNRKCSYCKIPQARGLGVSRNYQDVLDQVRFLQDNGVGEIVLTGVNL
66576257_Chlorobium_tepidu         QDGCSFGCAYCSIPLARGRSRSVSLSTVLDRAQKIADAGYREIVLTGINI
19704549_Fusobacterium_nuclea      QDGCNHFCSYCKIPFARGKSRSRKKENILKEIEKLVEDGFKEIILIGIDL
34541783_Porphyromonas_gingiv      QDGCDYHCSYCTIPKARGRSRNGSIESLVRQAEAVAAEGGKEIVLTGVNI
126663485_Flavobacteria_bacter     QDGCDYKCTYCTIPLARGISRSDTMEGVLKNAKEISAKGIKEIVLTGVNI
110638137_Cytophaga_hutchi         QDGCDYSCSFCTIPLARGSSRSDTIANIVKTAKEIAAKDVKEIVLTGVNI
32477342_Rhodopirellula_baltic     QDGCLLRCSYCIIPMVRPKLHSRPSQEIVDEVTRLVDAGHREVILTGIHL
16079597_Bacillus_subtil           QEGCNNFCTFCIIPWARGLLRSRDPEEVIKQAQQLVDAGYKEIVLTGIHT
118444753_Clostridium_novyi        QDGCNNFCSYCLIPFARGAVCSKNPEIIIDEVKKLAAHGFKEIILSGIDI
15605955_Aquifex_aeolic            QEGCNKFCTFCVIPYARGKVRSVDLEKIVHQVKLLAQKGFKEVVLTGTQL
32475869_Rhodopirellula_baltic     QIGCDKFCTYCVVPNTRGPEQGRSPEEIVSEARVLAEQGALEITLLGQTV
76261232_Chloroflexus_aurant       QYGCNMTCSYCVIPLRRGRERSRPLAEIVEEVRRIVARGAKEITLLGQIV
94985469_Deinococcus_geothe        MRGCDHHCTYCIVPTTRGPQVSRHPDDILRELDLLLAAGVQEVTLLGQNV
116625754_Solibacter_usitat        IEGCDKACAYCVVPFTRGPERSRTSESVMAEARGLTEKGYTEVQLLGQNV
66807387_Dictyostelium_discoi      MRGCNNMCSYCIVPFTRGRERSRPIDSILREVKDLSDQGFKEITLLGQNV
118402077_Tetrahymena_thermo       MRGCNNMCSFCIVPFTRGRERSRDIQSIVEEVKMLANQGVKEITLLGQNV
116061814_Ostreococcus_tauri       TRGCDNMCAFCVVPFTRGRERSRPFESVLEECRKLIDQGVKEITLLGQNV
CM1685_Cyanidioschyzon_merolae     MRGCNNHCAFCVVPQTRGPERSREPASIWNEIESLGNEGFKEVVLLGQNV
24213805_Leptospira_interr         MRGCNNFCTFCVVPYTRGRERSRDPKSIVREVQDLVQKGIRQITLLGQNV
Jakaba_bahamensis                  --------------------------------------------------
28872782_Homo_sapien               MRGCDNMCSYCIVPFTRGRERSRPIASILEEVKKLSEQGLKEVTLLGQNV
21674804_Chlorobium_tepidu         MRGCNNHCAFCVVPVTRGRERSVGFERVVAEVVALEKAGFREVTLLGQNV
30690642_Arabidopsis_thalia        MRGCNNMCAFCIVPFTRGRERSRPVESIIREVGELWESGVKEVTLLGQNV
34540755_Porphyromonas_gingiv      MRGCNNFCSYCIVPYTRGRERSREIESILNEVRDLKAKNFREVTLLGQNV
```

```
126663275_Flavobacteria_bacter      TRGCDNMCTFCVVPFTRGRERSREPQSIIDEIQDLYDRGFKEVTLLGQNV
110638221_Cytophaga_hutchi          MRGCDNMCSFCVVPYTRGRERSRDAHSILNEVRELVANGYKEVTLLGQNV
119885210_Thermotoga_petrop         IFGCDRFCTYCIVPYTRGREKSRPMEDILEEVRELAKQGYREVTFLGQNV
108759982_Myxococcus_xanthu         MKGCDNVCSFCIVPHTRGREVSRAFPDVLVEVADLAKVGVREVTLIGQNV
34581161_Rickettsia_sibiri          QEGCDKFCTFCVVPYTRGAEFSRNVEQVFREALKVVSSGAKEIMLLGQNV
94417083_Pseudomonas_aerugi         MEGCSKYCSFCVVPYTRGEEVSRPFDDVIAEVIHLAENGVREVTLLGQNV
CM4402_Cyanidioschyzon_merolae      SYGCNERCTYCVVPYTRGLEQSRSVESIVNEVVQLKNEGYREVTLLGQNI
33862596_Prochlorococcus_marin      IYGCNERCTYCVVPSVRGKEQSRSPEAIRLEIEGLAARGFREITLLGQNI
16329745_Synechocystis_sp.          IYGCNERCSYCVVPNVRGVEQSRTPEAIYGEMEVLAQQGFKEVTLLGQNI
111225071_Frankia_alni              SVGCDNTCTFCIVPSLRGRERDRRPGDVLAEVEALVGEGALEITLLGQNV
19703810_Fusobacterium_nuclea       TYGCNNFCTFCIVPYVRGRERSVPLEEIVKDVEQYVKKGAKEIVLLGQNV
15605821_Aquifex_aeolic             IKGCDKNCTYCVVPRTRGKERSRALHSILDEVKRLVDDGVREIHLLGQNV
118443818_Clostridium_novyi         MYGCNNFCSYCIVPYVRGRERSRDPQNIIDEIKDLVSKGYKEITLLGQNV
51892897_Symbiobacterium_therm      MYGCDKHCTYCIVPTTRGKERSRPYEVILAEVQELARQGFKEITLLGQNV
16078764_Bacillus_subtil            MYGCDKFCTYCIVPYTRGKERSRRPEDIIQEVRRLASEGYKEITLLGQNV
71075619_Giardia_lambli             GSGCMGSCTYCKTCHSRGRLRSVPLDTLLARIRSSLADIIRELWLTGEDT
13542169_Thermoplasma_volcan        NQGCTGKCNFCVSHIARGKLVSRKPEKIAGQIKILLQRGIKEIKITSLDT
71029160_Theileria_parva            STGCLGSCTFCKTKHSRGVLNSYEIESILDRVESCISEGVKEIWLTSEDL
71661088_Trypanosoma_cruzi          SVGCLNHCTYCKTKQARGDLRSWPIESIVLRVRSVLKEGVKEIRITSEDV
93277076_Homo_sapien                NTGCLNACTYCKTKHARGNLASYPIDELVDRAKQSFQEGVCEIWLTSEDT
118373032_Tetrahymena_thermo        R--CLGSCTYCKTKHARGKLGSYQPEAIVNRVKTVCEEGVKEIWLTSEDT
116056662_Ostreococcus_tauri        STGCLGACTYCKTKHARGDLGSYEISALVSRVEQAISEGVSEVWLSSEDT
18409989_Arabidopsis_thalia         NVGCLGACTYCKTKHARGHLGSYTVDSLVERVRTVISEGVKEIWLSSEDT
121522286_Methanococcus_maripa      CEGCLGSCTYCIVKRARGNLASYDRDLIVKKAEELVKTGTKCLLVTAQDT
18978284_Pyrococcus_furios          AEGCLNGCTYCATRSARGVLKSYSPEKIVEWVKWAIRQGYKEIWLSAEDT
15920732_Sulfolobus_tokoda          ADGCAGDCNFCITKLARKKLRSYPPRNIVNAVKEAVQKGAVEIELTAQDT
119871859_Pyrobaculum_island        QVGCLGNCTFCATKYTRGYVKSADPDDVVRHIKEAVAKGAREIYLTGQDV


24213838_Leptospira_interr          VYYGRDSELLDMVRKVAEISLEILRLLYLYPDKKTEKLIRLMGETIAPYL
111225093_Frankia_alni              TSYGKDLGLEKLLPQLAAVGIVRVRTVYLQPAELRPSLLEVLLTTLAPYL
15835786_Chlamydophila_pneumo       GDYGKDLSLESLLHELLKEGDYWLRMLYLYPDEVSDGIIDLMQSNLLPYV
76258351_Chloroflexus_aurant        TDYGRDLGLATLLAELCQVPETWIRLMYAYPHGISERLITTMASYICHYL
116060729_Ostreococcus_tauri        NQWGMDLRLAELLYALAEVGIEWMRILYAYPSYFSDELIQAIRTX-----
CM3493_Cyanidioschyzon_merolae      NMYGMDRGLAELLRYFQREGLVWIRLLYCYPSYFSDDLIAAIAECVVRCI
94417985_Pseudomonas_aerugi         SAYGVDLKVKTRMKELCEAMGVWVRLHYVYPYPNVDDVIPLMAAGLLPYL
94984483_Deinococcus_geothe         SAYGVDLRLIDLAEKLGEM-GAWVRMHYVYPYPHVERIVELMSQGILPYL
119886282_Thermotoga_petrop         TSYGIDLYLPDLLRRLNSLGEFWIRVMYLHPDHLTEEIISAMLELVVKYF
116620691_Solibacter_usitat         TCYGEDLGLAELLARLAQIQEKWIRFLYAYPNKVTQKLLDTLAEHLAKYI
118443956_Clostridium_novyi         AIYGSDLYLSQLLRELSNIDIEWIRILYTYPEEITDELIEEIKNNVCKYL
32476670_Rhodopirellula_baltic      TYYGMDRYLNQLLKELDKISIDWIRLMYFYPMYIDDALIDTLASAIVPYI
108760706_Myxococcus_xanthu         TAYGHDLPLHDLLKALVQVDVKWIRLHYAYPRIFPDELIEVMASEIARYL
51892771_Symbiobacterium_therm      TYYGLDLYLARLLRELAQVGIRWIRIHYSYPTRITDELIEVIVTEVLNYL
33862448_Prochlorococcus_marin      TNYGLDLYFAELLQALGEVDIPWVRVHYAYPTGLTPEVLAAYREVVLRYL
16331757_Synechocystis_sp.          TNYGLDLYLAELLQALGKVDIPWIRIHYAYPTGLTPKVIEAIRDTVLPYL
15606200_Aquifex_aeolic             TYYGKDLYLVELLEGLEKVGIKWIRLLYLYPTEVHEDLIDYVANSVLPYF
21674219_Chlorobium_tepidu          SVYGYDLYLNDLTLRLSDMGFNWIRLLYAYPLNFPLEVISTMRERVCNYI
34540013_Porphyromonas_gingiv       TFYGLDLYLAELTARLSDIGVEWLRLHYAYPAQFPLDLLPVMRERVCKYL
126662084_Flavobacteria_bacter      TYYGLDLYLAELLENLAKVGIEWIRLHYAFPSGFPMDVLDLMKREICNYI
110637073_Cytophaga_hutchi          TYYGLDIYLSDLLKNLSDVGIDWIRLQYAYPSGFPLDVLDVMAERICKYI
119885033_Thermotoga_petrop         GKYGKDMGLAELLKVIEKVGDYRVRLSSINVEDVNDEIVEAFKRNLCPHL
116622276_Solibacter_usitat         GRWGREPGLAGLLRLLLAEDVARLRLSSVEPMDFSEDLLHLMAASIANHV
34580504_Rickettsia_sibiri          TAYGSDLPFAQMIKRVLNLELKRLRLSSIDVAEIDDELFELIAYSIMPHF
15836008_Chlamydophila_pneumo       GDYCDGERLASLIEQVDQIGIERIRISSIDPDDITEDLHRAITSSTCPSS
24213088_Leptospira_interr          GWYRDSENFNKILGEILNIEYSRIRISSIEPPDVGNELVELMTHPFTPFL
66576257_Chlorobium_tepidu          GDYQDGDTLSGLLRRLETIDVSRIRISSVEPQLLDDELIDIVAASIMPHF
19704549_Fusobacterium_nuclea       SAYGEDFEFESLLEDILRIDLKRVRIGSVYPDKITDRFIELFKNKLMPHL
34541783_Porphyromonas_gingiv       GDFGRSTGFLDLLRALDQVGIERYRIGSIEPNLLSDELIDFCASAIAPHF
126663485_Flavobacteria_bacter      GDYGKGEFFLDLVTELDKVGIERLRISSIEPNLLKNETIELVSKSFVPHF
110638137_Cytophaga_hutchi          GDYGIISGFLDLIKELDKVGIERFRISSIEPNLLTDEIISFVSTSFVPHF
32477342_Rhodopirellula_baltic      GHYGVDWNLAHLVKDLCQIGQFRIRMSSIEATEVTRELIGVMAEFVVPHL
16079597_Bacillus_subtil            GGYGEDMKFAKLLSELDTRGVKRIRISSIEASQITDEVIEVLDRSIVNHL
118444753_Clostridium_novyi         SSYGVDLELLTILKKIDEIGITRVRIGSIGPEFFNEDRIKEIGKLLCPHF
15605955_Aquifex_aeolic             SQYGWDKGLYTLLTELIKIGIELIRLSSMHIKEMDKELLKLIVSEIAPHF
32475869_Rhodopirellula_baltic      NSYRHRGPMAGLLERLHDIGLKRIKFVTNYPKDMTARLLETIRDLVSPYL
76261232_Chloroflexus_aurant        DSWGHDLPLADLLEAVDPTGLLRLRFLTSHPAWMTDRLIETVARLCQPEI
94985469_Deinococcus_geothe         NAYGVDQGFANLLRLVGRSGIRRVKFTTSHPMNFTEDVAAAMAETVCEYV
116625754_Solibacter_usitat         NSYRDPSPFATLLAKVAEIGMRRVRYTTSHPRDFVRPIVDAMDANICDHI
66807387_Dictyostelium_discoi       NSYEESNNFTKLMELVSKVPEIRIRFTSPHPKDFPDDLLELIKNQICKQL
118402077_Tetrahymena_thermo        NSYFDKEAFAHLLEEVAKAPEVRFRFTSPHPKDFPDPVLQVIKKYIAKNL
116061814_Ostreococcus_tauri        NSYADASEFADLVEAVAAIVECRVRFTSPHPKDFPDDLLRVIANTVCKQL
CM1685_Cyanidioschyzon_merolae      NSYCYQSDFADLLEGVCEIHSMRVRYTSPHPKDFPHEVLRLYTRYLCRQI
24213805_Leptospira_interr          NSYKEQDTFAGLIQMLLDESIERIRFTSPHPKDFPTHLLQLMSENFCPNI
```

```
Jakaba_bahamensis                    -------------------------FTSPHPKAFPDALLHLMRERVCKQI
28872782_Homo_sapien                 NSFRDNSEFAHLLDQVSRVPEMRIRFTSPHPKDFPDEVLQLIHERICKQI
21674804_Chlorobium_tepidu           NSWRDAEKFAGLLEGVSLAPSMRIRFTTSHPKDISEALVKVIAARLCNHI
30690642_Arabidopsis_thalia          NSYNDDSAFADLLDRLSVEPEMRFRFTSPHPKDYPDELLYLMRDRICNLI
34540755_Porphyromonas_gingiv        NSYRYEQNFPDLLAAVAEAPDMRIRFTSPHPKDMDDEAIAVMARYICNHI
126663275_Flavobacteria_bacter       DSYGGGLKFAQLLDKCATLPKMRFRFSTSNPQDMHEEVLHVIAKHICNYI
110638221_Cytophaga_hutchi           DSYKWSAEFAQLLERVALVPDLRVRFSTSHPKDITDEVLYTMKKYICNSI
119885210_Thermotoga_petrop          DAYGKDLKLAKLLEEASKIGIERIWFLTSYPTDFSDELIEVIARNVAKSV
108759982_Myxococcus_xanthu          NSYAGGISFAQLLLRTAEVGIERVRFTTSHPHDLSDELIEAFRVQITPHF
34581161_Rickettsia_sibiri           NAYHGKGPLADLLKHLAQINLERLRYTTSHPIDMNNDLIKLYGTELMPFL
94417083_Pseudomonas_aerugi          NGFRGQTHFAELLRVVAAVGIERIRYTTSHPLEFSDALIQAHAEVLVKFI
CM4402_Cyanidioschyzon_merolae       DAYGRDMRFAQLLRHVSATGIDRIRAITAHPRYWSPRVIQATAELIMPYF
33862596_Prochlorococcus_marin       DAYGRDLPLTDLLHHIHDVGIERIRFATSHPRYFTERLIEACFDLVCEHF
16329745_Synechocystis_sp.           DAYGRDLPLTDLLYHVHDIGIDRLRFATSHPRYFTERLIQACQELVCEHF
111225071_Frankia_alni               NSYGRSLGFAKLLLACGRVGLERVRFTSPHPRDFTDDVIEAMAATVCHQL
19703810_Fusobacterium_nuclea        NSYGKDFKFAKLLDEICKVGDYIVRFVSPHPRDFTDDVIEVIAKNISKCL
15605821_Aquifex_aeolic              TAWGKDFEFSELLYQVSKIGVERIRFTTGHPRDLTDDIIEAMADIVCNAL
118443818_Clostridium_novyi          NSYGKGLEFATLLRMVNKIGLERIRFMTSHPKDVSDELIKAMAECVCEQG
51892897_Symbiobacterium_therm       NAYGKDLYFGDLIELIDRNGIERIRFTTNHPKDFTRKMVEQIARAVCEWF
16078764_Bacillus_subtil             NAYGKDFELGDLMDELRKIDIPRIRFTTSHPRDFDDRLIEVLAKGLLDHI
71075619_Giardia_lambli              LAWGRESDFAVLLQEVQKLTHKMLKIGMTDPDNQEDSLISFMRCKVYKFL
13542169_Thermoplasma_volcan         AAYGKDIGLPHLIRTITEIDDFRLRVGMMEPKEIVDDLISAYRSEVFKFL
71029160_Theileria_parva             GAYGIDLGIITLLHSIIAVKDIMLRLGMCNPPKYISEVCEILRHEVFEFI
71661088_Trypanosoma_cruzi           GAYGIDINIICLLRAIVKETEVMMRVGMSNPPRHLDDFAALLRHPVYEFV
93277076_Homo_sapien                 GAYGRDIGLPTLLWKLVEV-GAMLRLGMTNPPEHLEEMAKILNHPVYAFL
118373032_Tetrahymena_thermo         GAYGRDIGISQLLRLIVEV-DVMLRVGMTNPPEHLQNMSTILRHPVFSFL
116056662_Ostreococcus_tauri         GAYGIDLGVAALFRAITAVGSVMLRLGMTNPPAHLDAVAEAMRHPVYAWM
18409989_Arabidopsis_thalia          GAYGRDIGLPILLNAIVKEQSTMLRIGMTNPPEHLKEIAAVLRHPVYTFL
121522286_Methanococcus_maripa       ACYGLDNNLPNLINDISEIEKFAMRIGMMHAKPILDELIESFKSKVVKFL
18978284_Pyrococcus_furios           GCYGFDIGLAKLLDEITAIGEFRIRVGMMNPNKFLDELIEAYKDEVYKFL
15920732_Sulfolobus_tokoda           AAYGLDINLVELLKEILEIGNYMIRIGMMTPEKQIDEILEIIKDKVYKFL
119871859_Pyrobaculum_island         ITYGFDAGLPDLLDRILKEGEYRVRIGMSEPWKFVDQLLDIIKRDVYRYF


24213838_Leptospira_interr           ESPLQHVSSKILKVMNRTGESSYFKDLFSLAREVKPGLEIRTSFIIGYPG
111225093_Frankia_alni               DLSFQHASPAVLRRMRRFGGSEHFLDLLDRGRGLLPGLGARSNVIVGFPG
15835786_Chlamydophila_pneumo        DIPLQHINDRILKQMRRTTSREQILGFLEKLRAKVPQVYIRSSVIVGFPG
76258351_Chloroflexus_aurant         DMPLQHAHPATLRRMRRPPDTDRTLRIIAELRAAMPDIAIRSTFIVGYPG
116060729_Ostreococcus_tauri         -----------------------XXXXXXKLRDRIPGLALRTTFISGFPG
CM3493_Cyanidioschyzon_merolae       DIPLQHASDAVLQRMRRPL-MSHTSALLNKLRQRIPNLKLRSTFISGTPG
94417985_Pseudomonas_aerugi          DIPFQHASPKVLKAMKRPAFEDKTLARFKHWREICPELTIRSTFIVGFPG
94984483_Deinococcus_geothe          DVPLQHASPAVLKRMRRPGAGKQ-LDTIRRWREICPELVIRSTFIVGFPG
119886282_Thermotoga_petrop          DVPVQHGSDKILKLMGRTKSSEELKKMLSSIRERFPDAVLRTSIIVGFPG
116620691_Solibacter_usitat          DMPLQHASANVLKRMKRGASGDIFLKLIERIRRTIPGVAIRTSFIVGFPG
118443956_Clostridium_novyi          DIPIQHISNTVLKRMNRKSSKELITDNIKKMRKEIDGLCLRTSIIVGFPG
32476670_Rhodopirellula_baltic       DMPLQHASDKMLKRMARKTTRSLQTDIVQKLRSRIDSLVMRTTMITGFPG
108760706_Myxococcus_xanthu          DMPVQHVSDKLLLSMKRGRNSEFLKGLLTKLRERVPGLVMRTSLIVGLPG
51892771_Symbiobacterium_therm       DLPLQHGSNRVLRIMNRPANAEGYLRLVQKLRERVPDICLRSTFIAGHPG
33862448_Prochlorococcus_marin       DLPLQHSHPEVLRAMNRPWQTDVNERLLDRIREQLPDAVLRTTLIVGFPG
16331757_Synechocystis_sp.           DLPLQHSHPDILRAMNRPWQGQVNDDIITRLKTALPDAVLRTTFIVGFPG
15606200_Aquifex_aeolic              DVPLQHVSDRVLKDMRRGYDGKFVRNLIENIRKKIENAVFRTTFIVGFPT
21674219_Chlorobium_tepidu           DMPLQHINDRILKSMQRGIGRKATEQLIDDIRQKNPDIRLRTTMIAGYPG
34540013_Porphyromonas_gingiv        DMALQHISDPMLGRMRRRITKAETYELIERIRTEVPGIHLRTTLMTGHPG
126662084_Flavobacteria_bacter       DIPLQHISDNILKSMKRGTTKEKTTKLLQEFRERVPGMAIRTTLIVGYPG
110637073_Cytophaga_hutchi           DMPLQHGSSDMLKLMRRGIDRPKTEDLIKTIRDKVPGIAFRTTMIIGHPG
119885033_Thermotoga_petrop          HISVQSGSDDVLKRMGRKYRISDFMRVVDKLRSIDPDFSITTDIIVGFPG
116622276_Solibacter_usitat          HAPLQSGSDRVLRRMHRKYRPRHYADRVLKARALMPDGAIGADVMVGFPG
34580504_Rickettsia_sibiri           HISLQAGDDMILKRMKRRHNRANVIEFCRKLRAIRPEVSFGADIIAGFPT
15836008_Chlamydophila_pneumo        HLVLQSGSNSILKRMNRKYSRGDFLDCVEKFRASDPRYAFTTDVIVGFPG
24213088_Leptospira_interr           HIPLQSGNSEILKKMKRTYTPETFRKRVEIAKEKIPNLFLGTDIIVGFPG
66576257_Chlorobium_tepidu           HLPLQSGSDTVLRAMRRHYDTAFYRERLMKALSLIRGCAIGADVMVGYPG
19704549_Fusobacterium_nuclea        HISLQSCDDTVLKNMRRNYGSSLIKKSLLKLKSKVKDMEFTADVIVGFPK
34541783_Porphyromonas_gingiv        HMPLQSGSDDVLKLMRRRYDTALFRERVEHIRRALPHAFIGIDVIVGTRG
126663485_Flavobacteria_bacter       HIPLQSGSNDILKKMKRRYLRELYVDRVNKIREVMPHACIGVDVIVGFPG
110638137_Cytophaga_hutchi           HIPLQSGNDKILKLMRRRYKRELYAERVEAIKKVMPHACIGVDVIVGFPG
32477342_Rhodopirellula_baltic       HLCLQSGSDSVLRRMRRRWGTKMFLDRCRLLRESLDRPAITTDIIAGFPG
16079597_Bacillus_subtil             HIPIQSGSNTVLKRMRRKYTMEFFADRLNKLKKALPGLAVTSDVIVGFPG
118444753_Clostridium_novyi          HLSLQSGCNETLKRMNRKYTTEEFENVVKLLRKYIKDISITTDIIVGFPG
15605955_Aquifex_aeolic              HLSLQSGSNRILELMDRGYTREEYEEVVNFIVENRPISSIGTDVIVGFPT
32475869_Rhodopirellula_baltic       HVPAQSGSDAVLKRMKRGYTIADYMEMFERIETVLPEASVSSDFIVGFCG
76261232_Chloroflexus_aurant         NLPVQAGSDRVLKLMRRGYTVARYKTLIARIRAAIPDISLTTDIIVGHPG
94985469_Deinococcus_geothe          HLPVQSGSNRVLRRMAREYTREKYLSHIAEIRRHLPDVVLATDIIVGFPG
116625754_Solibacter_usitat          HLPVQSGSSKVLAAMDRLYTRDEYLRRIDWIKSAKRRYSLTTDIIIGFPG
```

```
66807387_Dictyostelium_discoi        HIPAQSGSSKVLESMRRGYTRESYIELIDTIKRVLPGCAISSDFISGFCG
118402077_Tetrahymena_thermo         HMPAQSGNTEMLTRMRRNYSRENYINLVDHIKQTIPGITLSSDFICGFCG
116061814_Ostreococcus_tauri         HMPAQSGSTSTLERMRRGYTREAYLELIERAREIIPGVAISSDFISGFCG
CM1685_Cyanidioschyzon_merolae       HVPMQSGSSEVLRRMRRGYTREEYLALVARIRDTVADVALSTDIISGFCG
24213805_Leptospira_interr           HLPLQAGNTRVLEEMKRSYSKEEFLDVVKEIRNIVPDVGITTDIIVGFPN
Jakaba_bahamensis                    HLPAQSGSTAVLHRMRRFYSREAYIELAERIRELIPGVALSSDFIAGFCG
28872782_Homo_sapien                 HLPAQSGSSRVLEAMRRGYSREAYVELVHHIRESIPGVSLSSDFIAGFCG
21674804_Chlorobium_tepidu           HLPVQSGSSRMLDLMKRGHTREEYLDRIAMIRSYIPEVAITTDLIAGFCT
30690642_Arabidopsis_thalia          HLPAQSGNSRILEQMRRGYTREAYLDLVKKIRSIIPDVAITSDFITGFCG
34540755_Porphyromonas_gingiv        HLPAQSGSDKMLRVMKRGYTRRWYLDRVAAIRRAIPDCAISSDLFCGFHS
126663275_Flavobacteria_bacter       HLPVQSGSTRILKEMNRQHTREEYMALIDKIYSIIPDISLSQDMIAGFPT
110638221_Cytophaga_hutchi           HLPAQSGNTRILELMNRTYTREWYMERITKIRQILDDCGISSDTIAGFCS
119885210_Thermotoga_petrop          HLPVQSGSNRILKLMNRSYTKEEYLALLERIRSKVPDVAISSDIIVGFPT
108759982_Myxococcus_xanthu          HLPVQCGSDRILKMMRRDYTVVQYLERLAKLREARPGIAVTTDIIVGFPG
34581161_Rickettsia_sibiri           HLPVQSGSNKILKAMNRKHDREYYFDIIHRLREARPDIVLSSDFIVGFPG
94417083_Pseudomonas_aerugi          HLPVQSGSDRVLAAMKRNHTVLEYKSRIRKLKAAVPDICISSDFIVGFPG
CM4402_Cyanidioschyzon_merolae       HIPFQAGDDEVLKAMGRGYTAKRYRRIIEMIREYLPDAAITADAIVGFPG
33862596_Prochlorococcus_marin       HIPFQSGDNDVLKAMARGYTVERYRRIVNRIRELMPDAAISTDVIVAFPG
16329745_Synechocystis_sp.           HIPFQSGDNDILKAMKRGYTREKYLQIIEKIRRYMPDAAISADVIVGFPG
111225071_Frankia_alni               HMPLQSGSDDVLRRMRRSYRRDRFLGIVERVRAAMPDAAITTDIIVGFPG
19703810_Fusobacterium_nuclea        HLPLQSGSSQILKKMRRGYTKEKYLALVDKIKSKIPGVALTADIIVGFPG
15605821_Aquifex_aeolic              HLPFQAGSNRILALMDRGYTKEEYLEKIEKLKEKVKDIAMSTDVIVGFPT
118443818_Clostridium_novyi          HFALQSGSTEILQKMNRKYTREDYLTLVKKLRKAMPNVGISTDIIIGYPG
51892897_Symbiobacterium_therm       HLPVQSGSDSVLRRMKRSYNRKQYLRLVGWIRELIPDAVITTDIIVGFPG
16078764_Bacillus_subtil             HLPVQSGSSEVLKLMARKYDRERYMELVRKIKEAMPNASLTTDIIVGFPN
71075619_Giardia_lambli              HLPVQSGSDRILTLMRRHYDIETFLRSCSKLQSAVPDLCLDTDIICGFPG
13542169_Thermoplasma_volcan         HLPVQSGDDYVLEVMNREYTVSDFIRITGKFREAFPDSTLSTDLIIGYYA
71029160_Theileria_parva             HIPVQSCSDSVLEKMNREYKLEDFLHIVSVIKEKVPNCTIATDIICGFPT
71661088_Trypanosoma_cruzi           HIPIQSGSNRILNAMQREYTLEEFYECVHRIRSVVPNVTLATDIICAFPG
93277076_Homo_sapien                 HIPVQSASDSVLMEMKREYCVADFKRVVDFLKEKVPGITIATDIICGFPG
118373032_Tetrahymena_thermo         HIPVQAANNTVLENMNREYTCEEFEQVCDYLLKNVPNMTIATDIICGFPG
116056662_Ostreococcus_tauri         HIPVQSGSNAVLEAMKREYTVEEFRTVCDTLLEAVPGMVIATDVICGFPG
18409989_Arabidopsis_thalia          HVPVQSGSDSVLTAMNREYTASEFRTVVDTLTELVPGMQIATDIICGFPG
121522286_Methanococcus_maripa       HLPIQSGDDQVLKDMNRNYTVDEYISVLNEFKSKIKNLNFTTDVIVGFPT
18978284_Pyrococcus_furios           HLPVQSGDNEILRRMGRTYTVEEFEEIVNAFRREFPDLNLHTDIIVGFPG
15920732_Sulfolobus_tokoda           HLPVQSGDDRVLKLMNRKYTVDEYRELVKEIRDKIPIVNITTDIIIGHPG
119871859_Pyrobaculum_island         HLPVQSGSDKVLVAMGRKYTVEEYRGLIRRIRRELDNVFIATDIIVGFPG
                                                                                :  .

24213838_Leptospira_interr           EEPEDVDQILRFIEDTRPEKVNLFSYSPQEGTKGAQVSEKEKSKRINLIR
111225093_Frankia_alni               ETEADVDILAEFLEAAELDAVGVFGYSDEEGTEAVSIAEEEIERRRVQIT
15835786_Chlamydophila_pneumo        ETQEEFQELADFIGEGWIDNLGIFLYSQEANTPAAEIPEKVKESRLKILS
76258351_Chloroflexus_aurant         ETTAEFHALLEFLQTAQLDRVGAFRYSREPGTPAAEVRPQVIERRWHELM
116060729_Ostreococcus_tauri         ETEEEHNELMQFCRDFKFERLGAFAYSEEDGTPAMEVPEDVRAFRRDQLV
CM3493_Cyanidioschyzon_merolae       ETARDHQQLVRFIQEMRFARAGFFSYSAEERTPMAEVPPQVQERRRDQLF
94417985_Pseudomonas_aerugi          ETEEDFQYLLDWLTEAQLDRVGCFQYSPVEGAPANEVPDEVKQDRWERFM
94984483_Deinococcus_geothe          ETEEDFQLLLDFLEEARLDRVGAFTYSDVEEADANAIPEEVKQERLARFM
119886282_Thermotoga_petrop          ETEEDFEELKQFVEEIQFDKLGAFVYSDEEGTVAFNVDPEMAKRRQEELL
116620691_Solibacter_usitat          ETAADFDELCAFVEAAKFDNLGVFTYSDEDTSASYAVDGRTIQNRKRRLM
118443956_Clostridium_novyi          ETEDEFNELKEFVEEIKFDNLGVFKYSQEEDTAAARVSEDLKEERLATIM
32476670_Rhodopirellula_baltic       ETEEDFVELMDFVQESRFENLGVFTYSIEEDTPAARVDPEVAARRRDDLM
108760706_Myxococcus_xanthu          ETEEDFEMLKEFVKTQRFERLGVFQYSDEEGTAAYDVPQKLIERRWREVM
51892771_Symbiobacterium_therm       ETEEDFELLLDFLRACEFDHVGVFAYSQEEGTKAGQLPEEVRLARRDRAM
33862448_Prochlorococcus_marin       ETEDHFNHLAAFIERQRFDHVGVFTFSPEDGTAAADVDPSIAAARKDRLM
16331757_Synechocystis_sp.           ETEEHFGHLLDFVQRHQFDHVGVFTFSPEEGTAAFDVPEEVMGDRRDRLM
15606200_Aquifex_aeolic              ESEEDFKELKKFVEEGHFHWLGVFTYSPEEGTHAYPIPREVKEERREELM
21674219_Chlorobium_tepidu           ETRAEFEELLDFIRQTRFDRLGCFPYRHEEHASAYAVSDEEKEKRVGELM
34540013_Porphyromonas_gingiv        ETERDFEELLQFVRDIRFERLGAFTYSHESGTYCDKIPESVKQERLGELM
126662084_Flavobacteria_bacter       ETQEDFEILRDWVQEMKFERLGCFTYSHEENTGAFVVPQEVKQARAAEIM
110637073_Cytophaga_hutchi           ETEKDFDELCSFVEEQRFDRLGAFTYSHEEHTHSYSIPQEEKEERQATIM
119885033_Thermotoga_petrop          ETDDDFQKTLDLVEKVGFSRVHIFRFSPRPGTPASRVPESKKKERLDVLK
116622276_Solibacter_usitat          ETDAEFEESRRFIEQLPFTYLHVFTYSERPGTPAAEVPMPVRKERNRVLR
34580504_Rickettsia_sibiri           ETPEMFENTRKLISEAELQYLHVFPYSEREGTPAARVPKNIRKERAAILR
15836008_Chlamydophila_pneumo        ESDQDFEDTLRIIEDVGFIKVHSFPFSARRRTKAYTIPNQVIYERKKYLA
24213088_Leptospira_interr           ETEEMFQDSVSMIRDLGFAKIHTFPFSVRRNTLAETVSKEIKKERVHTLN
66576257_Chlorobium_tepidu           ESERDFEEMCRFIEELPVAYLHVFTCSPRPGTKLFARIPSAESSSRAARL
19704549_Fusobacterium_nuclea        EDEIMFQNTYDVIKEIEFSGLHIFQYSDREGTIASNIDVKTKKQRADRLD
34541783_Porphyromonas_gingiv        EKPEFFEDCYSFLQDKSFSRLHVFSYSERPGTQALQVDAREKQRRSRRLL
126663485_Flavobacteria_bacter       ETDEHFLETYHFLNDLDISYLHVFTYSERDNTEAAEVPANVRSKRSKMLR
110638137_Cytophaga_hutchi           ETDEDFNVTYNFLNELDISYLHVFSYSERANTLAATRPNRDRSERSTMLR
32477342_Rhodopirellula_baltic       ETEEEFEQTLRTCREAGFSKIHAFPYSARRGTPAAELDKGLISERVDRLG
16079597_Bacillus_subtil             ETEEEFMETYNFIKEHKFSELHVFPYSKRTGTPAARVDENVKNERVHRLI
118444753_Clostridium_novyi          ETIEEFDETYEYLKRIELSKMHIFKYSPRTGTRAEKVDGNIKEERSKALI
```

```
15605955_Aquifex_aeolic          ESEEDFQETYEFLKRIPISYMHIFPYSDRPFTKASKLPERIKKERVRILK
32475869_Rhodopirellula_baltic   ETDEDFQKSVKLIERCRFKNSFIFQYSVREGTKAAAVPREVKAARNNELL
76261232_Chloroflexus_aurant     ETEDDFRQTMDLCAEIGFDKVHIAAFSARPGTRAAEVPPAVKEERRRRLE
94985469_Deinococcus_geothe      ETEEDFQETLSLYDEVGYDAAYMFIYSARPGTPSYKLPREVKTERLQRLI
116625754_Solibacter_usitat      ETDADFEQTLDLLDEVQYDSLFSFKYSPRPNTSA-LIPEEEKQRRLLTLQ
66807387_Dictyostelium_discoi    ESDQDHNETISLMEYVGYENAFMFMYSLREKTHAHRVPTTLKNSRLTQVV
118402077_Tetrahymena_thermo     ETDQEFQDTLTLLEYVKYENAFLFAYSLREKTHAHRVPEEVKKQRLQQMI
116061814_Ostreococcus_tauri     ESEDEHAETLTLLETVRYEHARTR-----------RRPEEVKKRRLAEVI
CM1685_Cyanidioschyzon_merolae   ETEAEHAETISLMEQVRFDQAFMFAYSERERTIAARIPKEVKLRRLGEII
24213805_Leptospira_interr       ETEEEFEDTLAVVREVQFDMAFMFKYSEREGTMAQRVPEEVKSARLTKLV
Jakaba_bahamensis                ETEAEHRDTVSLIERIRYEHAFLFAYSMREKTHAHRVLEEVKIRRLNELV
28872782_Homo_sapien             ETEEDHVQTVSLLREVQYNMGFLFAYSMRQKTRAYHVPEEVKLRRLEELI
21674804_Chlorobium_tepidu       ETEKDHRETLSLMEAVGYDTAFMFHYSVRPGTWAARVPDTVKKARLQEII
30690642_Arabidopsis_thalia      ETEEEHQETLSLVRAVGYDMAYMFAYSMREKTHAHRVPEEVKQRRLTELI
34540755_Porphyromonas_gingiv    ETEEDFEATLSLMEEVRYDSAFMFKYSERPGTYAARVPEEVKLSRLDRMI
126663275_Flavobacteria_bacter   ETEEDHQDTLSLMEYVKYDFGFMFAYSERPGTLAARVPEEVKKRRLTEIV
110638221_Cytophaga_hutchi       ETEEEHQDTLSLMEFARFDFGYQFVYSERPGTPAAKVPEDVKQRRLSEII
119885210_Thermotoga_petrop      ETEEDFMETIDLVEKAQFERLNLAIYSPRKGTVAWKVPYEEKVRRMQFLM
108759982_Myxococcus_xanthu      ETEEEFEMTMQLTEQVRYDNQFSFVYSPRPKTGAALVPHEVKIARLERLQ
34581161_Rickettsia_sibiri       ETDEDFEGTLDLVRRVKYGQCYSFKYSPRPGTPGATIPEHIKSERLTILQ
94417083_Pseudomonas_aerugi      ETEKDFEQTMKLVEEVGFDFSFSFIYSARPGTPAADLPEEVKKRRLQILQ
CM4402_Cyanidioschyzon_merolae   ETEEQFQRTLELMEALQLDNVNTAAYSPRPFTPAASVPEAVKMERLHRIN
33862596_Prochlorococcus_marin   ETDAQFQNTLNLLEEVGFDQVNTAAYSPRPNTPAATLPEAVKVERLKQLN
16329745_Synechocystis_sp.       ETEAQFENTLNLIEEVGFDLLNTAAYSPRPGTPAAFLSEEVKGDRLQRLN
111225071_Frankia_alni           ETEADFADTLDVVRQARFAGAFTFQYSPRPGTPAATIDRTTVAERYARLV
19703810_Fusobacterium_nuclea    ETEEDFLDTIDVVQKVSFDNSYMFMYSIRKGTKAATIEESVKKERLQRLM
15605821_Aquifex_aeolic          ETEEDFEHTLDVLKKVRFEQVFSFKFSPRPGTPAAEIPDDVKTERMRRLL
118443818_Clostridium_novyi      ETEKDFEDTLSIVKEIEFDSAFTFIYSKREGTPAAKVPEDVKHTRFNKLV
51892897_Symbiobacterium_therm   ETEEEFQETLSLVEEVQYDAAFMFMYSERAGTPAAQLSVPEKKERLQRLM
16078764_Bacillus_subtil         ETDEQFEETLSLYREVEFDSAYTFIYSPREGTPAAKVPMRVKKERLQRLN
71075619_Giardia_lambli          ETDEDHAQSLNLFRNLRFQVVNITQYYARKNTPAASVPASKKRERTREMS
13542169_Thermoplasma_volcan     ENDDSFEKTVQLIEKIRPEIINVTKFSPRELT--ADKPTNILKEQDRMIA
71029160_Theileria_parva         ETHEDHQATVDTLRDLKLSVINISQYYSRKGTVSSGLDNNVKKNRSREVT
71661088_Trypanosoma_cruzi       EGEAEWRETMELCDHVRFPVLNITRFYPRRNTPAASIPTDVAKRRTSELT
93277076_Homo_sapien             ETDQDFQETVKLVEEYKFPSLFINQFYPRPGTPAAKVPAQVKKQRTKDLS
118373032_Tetrahymena_thermo     ETNAQFDDTLKLVDKYKFPILNISQFYPRPGTAAMKVPSQDVKMRSKKIT
116056662_Ostreococcus_tauri     ETDEQWRETMQLIEEYKFPEVHISQFYPRPGTPAARVPTQIVKGRSRELT
18409989_Arabidopsis_thalia      ETDEDFSQTVELIKDYKFPQVHISQFYPRPGTPAAKVQSKIVKQRSRELT
121522286_Methanococcus_maripa   ETEEAFENTLEIVKKIKPDFTHAAKYSQRKYTKAAIVDTKIRKERSEILN
18978284_Pyrococcus_furios       ESEEAFQRSLELIKRIKPDKVNVSRYSPRPGTIAAKLPGWVVKERSRIMH
15920732_Sulfolobus_tokoda       EDDEAFQNTINLIKEIKFERIHLAMYSIRPNTRSASVPDSIKKERMKIAN
119871859_Pyrobaculum_island     ETEEDFQETVKLVEELQFDKIHVARFSPRPFTEAAVVPDAEKKRRSKILS
                                 *

24213838_Leptospira_interr       DSHLEILEEIHESRIGRTYDAIVDGVRRFQDAPEMPGRIGKVRIDSFYEY
111225093_Frankia_alni           DLVEQLTATRAQERIGSRVQVLVEEGCAGHQQPDAVGDLIEARVVATEGV
15835786_Chlamydophila_pneumo    QIQKRNVDKHNQKLIGEKIEAVIDNARFYGQAPEVFGERCFIEITGTAGY
76258351_Chloroflexus_aurant     RLQQTISYTRNQRWVGRTIKVLIEGGRSFRDAPEIAGTMIPVQVTQATAY
116060729_Ostreococcus_tauri     SQQQEISEDFAMSRVGKDVDVLIDSGRTTLEAPDIPGQMRKCNIIGSSLF
CM3493_Cyanidioschyzon_merolae   SLQQTIQEQIAESLIGTEIDILIDRGRTFMDAPDIPGSLVRARVVSTSGW
94417985_Pseudomonas_aerugi      AHQQAISAARLQLKVGKEIEVLIDEGRSWADAPEIPGDKVRVRITDADEY
94984483_Deinococcus_geothe      EVAQRISREKLAEKVGRVLDVIIDEGRTKGDAPGIIGDIVQARIEDSDEY
119886282_Thermotoga_petrop      LLQAEISNSRLDRFVGKKLKFLVEGGRTWTEAPEVIGDFLEVVIKEHDEY
116620691_Solibacter_usitat      AIQRKIARARNRGLVGKEVPVLVSGARMSTQAPEIAGEIRRLRITEAHDY
118443956_Clostridium_novyi      SIQQNVSSKINKNKLEKVYKVLVEGGRNYQMVPEIVGEFVYVKITDTLEY
32476670_Rhodopirellula_baltic   ELQQQIAFDWNDSRVGGTEEVLIDAGRTRSEAPDVVGQIRPCEIVASQGY
108760706_Myxococcus_xanthu      AIQKRINREQNKKLVGKRLEVLVEGGRHQGQAPDIPGEIVTVEVTEAHDY
51892771_Symbiobacterium_therm   EVQQEIARRRNQLQVGRELEVLVEGGRCYGQSPGIPGDMVQVRITGVQDY
33862448_Prochlorococcus_marin   ALQQPISAERNQRWVGRTIDVLIEQGRCDRFAPEVPGTMVPVFITGSDVY
16331757_Synechocystis_sp.       ALQQPISAQKNAACLGQTLDVLIEQGRSTRFAPEVLNEIVPVVITATDDY
15606200_Aquifex_aeolic          AIQRGITRKKNEEFLGKEIEVLIDGGRAYFQAPEVSGDILKVKVTQVADY
21674219_Chlorobium_tepidu       ELQEGISASLNRKLEGQTLKVLIDRARTEYDAPEVEGDFRQVMIEDSTAY
34540013_Porphyromonas_gingiv    AVQERISAAHNEAKIGSRLHVVIDRGRTEYDSPEVPGRFYMAEVTGAEPF
126662084_Flavobacteria_bacter   DLQSQISWDLNQEKIGQTFKCVIDRGRTEFDSPDVTGDFVNLKITDATEF
110637073_Cytophaga_hutchi       SIQEGISAELNEKKIGNTYKVLFDRGRTEHDSPEVIGDFANVKINDAAEF
119885033_Thermotoga_petrop      EKAKDVSIRYRKRIIGKERKVLAEWGYDEYYVKHEVGEFHNVRVKSLSEE
116622276_Solibacter_usitat      ELAAAKNLAFRESMIGRTLSAVTLA--TENYLKVEPGALVDLHIGGLASA
34580504_Rickettsia_sibiri       QEGQNQLTEFFKKHIGQKVELLVENAHTENFIPVKIGQIFKAKLVAIEEN
15836008_Chlamydophila_pneumo    EVAKRVGQKEMMKRLGETTEVLVEKGHSPYFEKVSINTLVSVRLDRVEEE
24213088_Leptospira_interr       SLSRELHQKYALSEIEKVREAILEQ--TDNYLKVKVGQFLNVKLLQYEPA
66576257_Chlorobium_tepidu       GVIGERIERRFAEAFIGSTLKVLFEGYSEHYLRVSRGQVREVIIDGFGEL
19704549_Fusobacterium_nuclea    SLKQEMIVDSRKKYLEKSLEVLVEEGYSQNYLRVKVNNLINVKVKCVEND
34541783_Porphyromonas_gingiv    DLSDLRLAEFYRSQEGTHRRVLWEDGFTENYIRVAVGKVEEIVLGKTDP-
```

```
126663485_Flavobacteria_bacter      GLSVKKRRAFYESQIGTKRTVLFENGFTENYVKVKVNTLHEINLTKIDEG
110638137_Cytophaga_hutchi          TLSEKKRRFFYEQHITETADVLFEAGFTNNYVKVVVNELKKVKLKTINTG
32477342_Rhodopirellula_baltic      EVEAELRQQYYKTLVGSNLELLVEEGTTCRYALGSENDLITARVVSVQDD
16079597_Bacillus_subtil            ALSDQLAKEYASQYENEVLEIIPEEGYTDNYMKVVIGKIVKVKILKAGYP
118444753_Clostridium_novyi         KLNEINEKKFIDKFIGEDMEVLYEQGYTPNYIKVISGKIVNTKLIETKDE
15605955_Aquifex_aeolic             ELDQKKRQEFYEKNKGKELRALVIE----------TENYIDIKREGYKE-
32475869_Rhodopirellula_baltic      AVQDRISKEDNQKLIGDTVEVLVEGGRTICDRIVVAGQLMDIQIDDVSSH
76261232_Chloroflexus_aurant        QLQEQIATERMARFLGQTVEVLVEGGRTPGNRLVFTGQLVPVKITATSPW
94985469_Deinococcus_geothe         AKQKEWSARKNAAKVGTIQEVLLRGGHTRGNHPTVGPGIYRARIEHATPH
116625754_Solibacter_usitat         EKQRAIQIRRNAEMIGSIQEVLVEGGRTTCNRTLNVGKYLPVRVTRSGPN
66807387_Dictyostelium_discoi       DTFYKRLKEKNQLEIGNHHLVLVDGGRTDTNKKVLVGEYYIVEIIKGDSV
118402077_Tetrahymena_thermo        DVFHKNQELVNKQEVGRIHLVLVEGGRTDTNKRVINGDFVLARVDSVSVR
116061814_Ostreococcus_tauri        ETFRRCAADVNAREVGCTHLVLIEGGRSDTGKRVVSGDYVVAKIVEAGAS
CM1685_Cyanidioschyzon_merolae      STQLKISAERARAMVGTEQRVLVEEARTESNRRVWIGDYVEVSITGVSGN
24213805_Leptospira_interr          DLQTSISHEQNRARIGRVYSILIENGRTPCGRMTVIGSTVSVQIESATSA
Jakaba_bahamensis                   TAFHAGALERNRAEIAREHLVLVEGGADESSIVVVRGEYVHVRVTDCTSA
28872782_Homo_sapien                TIFREEATKANQTSVGCTQLVLVEGGRNDGNLKVIPGDYVLVKITSASSQ
21674804_Chlorobium_tepidu          ELQNAISREIFQREIGKTVEVLAEAGRTPENRVVVPSDTLLVKITGATSA
30690642_Arabidopsis_thalia         DAFRETTGPCYDSQVGSTQLVLVEGGKTDKGHRVSIGDFVEVQIEKSTRA
34540755_Porphyromonas_gingiv       ALQNRLSEESNKRDIGKTFEVLIEGGRTQQNKVVIVGQYIYVRIKDASSA
126663275_Flavobacteria_bacter      DLQQKHGLERTQRFIGQEVEVLIEKGRNSQNTVVVVGEFVMVKITDCTAA
110638221_Cytophaga_hutchi          TVQRQLSLEHNQAQIGTIQKILIEGGRNSRNMMAIKGQYVDVLITGCSGG
119885210_Thermotoga_petrop         NLQKRINRKLNERYKGKTVRVIVEAGRDIRNKIIAIGRFADVKIEKITAG
108759982_Myxococcus_xanthu         KLQRRISGEITAALVGSEVEVMVEGGRTPENRTVNAGSFVTVKVERATPN
34581161_Rickettsia_sibiri          QELMAQQLAFNTSCVGSTMKVLFDRGKTPYMQSVYLGKIIDVKITKASLN
94417083_Pseudomonas_aerugi         GRIHQQGYEISRRMVGSTQRILVTDGRTENNRIVNIGQFAQVHIDDALPH
CM4402_Cyanidioschyzon_merolae      ELNMRIVDARSQRYLGRIEEVLVEEGRTRTNKKVFRGRMANVRITQARAY
33862596_Prochlorococcus_marin      ALVERIALQRNSRYSGKVEQVLAEGGRTRTNRLTFPGDLVDIQINSVRAF
16329745_Synechocystis_sp.          HLVSTQAMERSQRYLGRVEEVLVEGGRTRGNRLTFLGKTVPVKITEARAF
111225071_Frankia_alni              ALQDEVSWAQNRELVGRRVELLVAEGRARDGRLVHPGDVVETVVTRAAPH
19703810_Fusobacterium_nuclea       EVQNKCSFYESSKYKGRIVKVLVEGGRTSTNKIVLKGQFINVKINECKTW
15605821_Aquifex_aeolic             ELQKSILSEIAKKYEGTVQEVLVEEGRTTTNKWASLGKIVKIKVIKSSPF
118443818_Clostridium_novyi         EAVNEIMARKNKEFEGKTVEVLVEGGRTRSGKLVNVGKLVNIKITKANSF
51892897_Symbiobacterium_therm      EVQNRIARAKNEARVGKVYDILVEGGWTRGNILVTRGRIVPVRITRAGTW
16078764_Bacillus_subtil            ALVNEISAKKMKEYEGKVVEVLVEGGYTEKSKLVNIGKIVRVKIQQAKTW
71075619_Giardia_lambli             EVVK--NSFRRDQYHGQVHDIMVLEGKTYNNLTVALGSYVRVRITGSTRV
13542169_Thermoplasma_volcan        DMHKEILQEKFSNLIGKTEFVLITEGRDHAYRPIVKFEFHQVEITGFENT
71029160_Theileria_parva            NVFM--SYENNSKFLNEQFKAYFNHGYNKYYIKLILGTTQHVKVINTHKW
71661088_Trypanosoma_cruzi          AFFN--SYRTLDHMVGEVHSVALLEGHTKAYVQVLLGETVTVVIMSTTKY
93277076_Homo_sapien                RVFH--SYSPYDHKIGERQQVLVTEAHNQFYEQVLMGKMVEVDIYESGKH
118373032_Tetrahymena_thermo        ELFD--TFKRWDHLLGTTQRIWINDGHTKQYAKVMLGKSVIIKVTKVLTW
116056662_Ostreococcus_tauri        VLFE--SYQPHQHLVGKTERVWVSDAHTKNYTQILMGRSAMVEIYESSRW
18409989_Arabidopsis_thalia         SVFE--AFAPYTGMECREERIWITEGHTKGYVQVLLGTSAMARITSVGRW
121522286_Methanococcus_maripa      ELRRELSYENNTRHIGETFEILVTKGVTDNCKNVIIGEFRRVKVTGAKTF
18978284_Pyrococcus_furios          RVRLQISYEINQRYVGRKVDILVHGAVTMNYKHIIKGEFARAKVNGATST
15920732_Sulfolobus_tokoda          EVYEEVAYSVHSEYLGSNALVLTTEGRTINYIPVVLGEWINVKITEASFF
119871859_Pyrobaculum_island        EVAMRIAHLRNGAQVGKRDVVLIDEGRASDYRQVVLGEFVNVRIAAASPV


24213838_Leptospira_interr          DMNGT
111225093_Frankia_alni              DLVAE
15835786_Chlamydophila_pneumo       DLVGR
76258351_Chloroflexus_aurant        DLWGE
116060729_Ostreococcus_tauri        DLEAQ
CM3493_Cyanidioschyzon_merolae      DLLCV
94417985_Pseudomonas_aerugi         DLWAE
94984483_Deinococcus_geothe         DLYGE
119886282_Thermotoga_petrop         DMWGS
116620691_Solibacter_usitat         DVVGT
118443956_Clostridium_novyi         DLIGV
32476670_Rhodopirellula_baltic      DLVAA
108760706_Myxococcus_xanthu         DLVAR
51892771_Symbiobacterium_therm      DLLGE
33862448_Prochlorococcus_marin      DLTGQ
16331757_Synechocystis_sp.          DLYGM
15606200_Aquifex_aeolic             DLAGR
21674219_Chlorobium_tepidu          ELFGR
34540013_Porphyromonas_gingiv       DLYAR
126662084_Flavobacteria_bacter      DLYAE
110637073_Cytophaga_hutchi          DLFGE
119885033_Thermotoga_petrop         GVISC
116622276_Solibacter_usitat         GLREQ
34580504_Rickettsia_sibiri          YMKCM
```

15836008_Chlamydophila_pneumo	GLIGE
24213088_Leptospira_interr	DKEGT
66576257_Chlorobium_tepidu	QLHGR
19704549_Fusobacterium_nuclea	ILIAE
34541783_Porphyromonas_gingiv	--SGE
126663485_Flavobacteria_bacter	SVRLQ
110638137_Cytophaga_hutchi	LVEIE
32477342_Rhodopirellula_baltic	RLELA
16079597_Bacillus_subtil	YNEGQ
118444753_Clostridium_novyi	YAVGK
15605955_Aquifex_aeolic	--VGK
32475869_Rhodopirellula_baltic	TLIGR
76261232_Chloroflexus_aurant	SLQGV
94985469_Deinococcus_geothe	MLYGR
116625754_Solibacter_usitat	SLVGE
66807387_Dictyostelium_discoi	TLKGK
118402077_Tetrahymena_thermo	SLFCT
116061814_Ostreococcus_tauri	TLVAE
CM1685_Cyanidioschyzon_merolae	RLIGE
24213805_Leptospira_interr	TLKGR
Jakaba_bahamensis	TLRAE
28872782_Homo_sapien	TLRGH
21674804_Chlorobium_tepidu	TLSGE
30690642_Arabidopsis_thalia	SLFGE
34540755_Porphyromonas_gingiv	TLFGE
126663275_Flavobacteria_bacter	TLIGE
110638221_Cytophaga_hutchi	TLFGE
119885210_Thermotoga_petrop	PLYGK
108759982_Myxococcus_xanthu	QLAGK
34581161_Rickettsia_sibiri	SLTGE
94417083_Pseudomonas_aerugi	SLRGT
CM4402_Cyanidioschyzon_merolae	SLTGE
33862596_Prochlorococcus_marin	SLSGT
16329745_Synechocystis_sp.	SLTGE
111225071_Frankia_alni	HLTAD
19703810_Fusobacterium_nuclea	TLYGE
15605821_Aquifex_aeolic	SLEGE
118443818_Clostridium_novyi	SLTGE
51892897_Symbiobacterium_therm	TLEGE
16078764_Bacillus_subtil	SLDGE
71075619_Giardia_lambli	ALIGS
13542169_Thermoplasma_volcan	SLIGR
71029160_Theileria_parva	HLECE
71661088_Trypanosoma_cruzi	SVVGR
93277076_Homo_sapien	FMKGQ
118373032_Tetrahymena_thermo	HIEGE
116056662_Ostreococcus_tauri	SCKAR
18409989_Arabidopsis_thalia	SVFGE
121522286_Methanococcus_maripa	GLSGK
18978284_Pyrococcus_furios	YLLGE
15920732_Sulfolobus_tokoda	DLRGK
119871859_Pyrobaculum_island	YLYGE

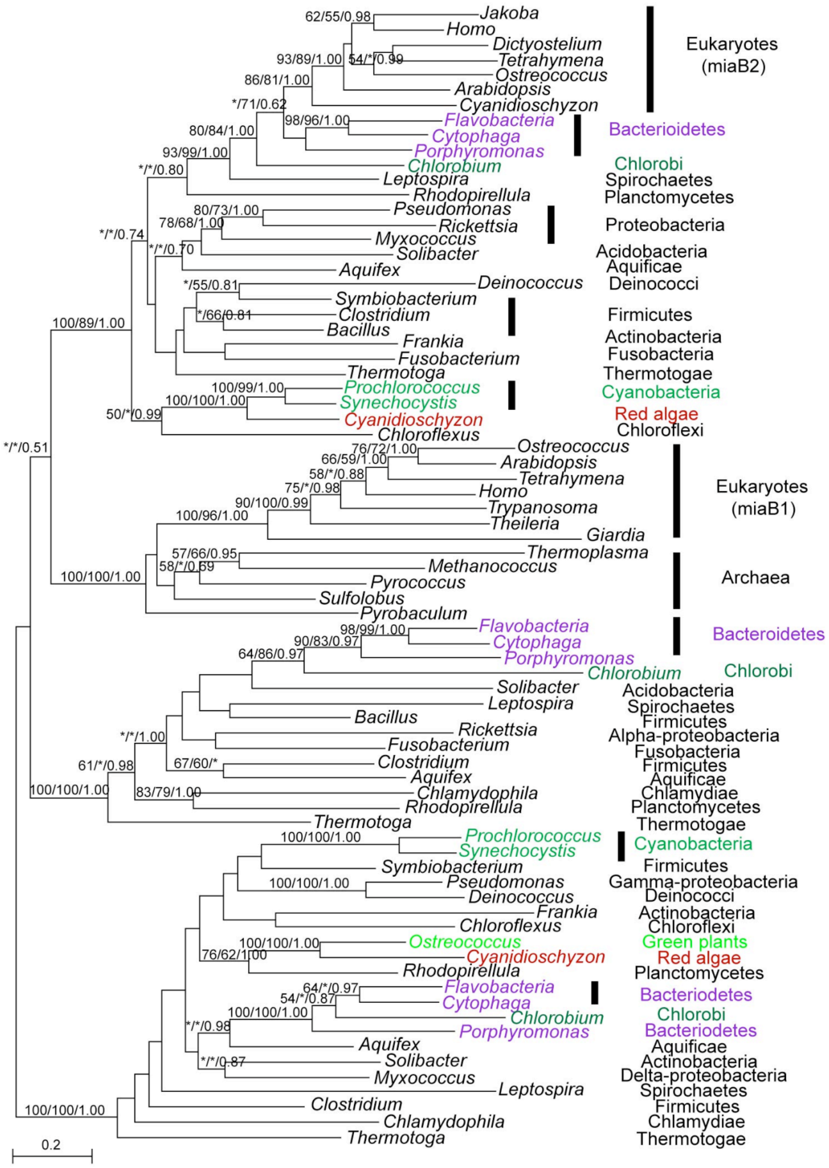

Figure 9. Molecular phylogeny of 2-methylthioadenine synthetase. P-value = 0.983 from AU test for the presented tree. See text for a more detailed discussion. AU tests were also performed on alternative topologies, including (**A**) *miB1* and *miaB2* forming a monophyly, (**B**) *miaB1* and *miaB2* forming a monophyly that in turn groups with archaeal sequences, and (**C**) *miaB2* forming a monophyly with proteobacterial sequences from the top part of the tree. These tests investigate if (a) *miaB1* and *miaB2* have the same origin, (b) *miaB1* and *miaB2* have a eukaryotic origin and are related to archaeal homologs, and (c) miaB2 has a mitochondrial origin. P-values < 0.001 from AU tests for these alternative topologies.

---

11. Uroporphyrinogen III synthase (D)

```
CLUSTAL X (1.83.1) multiple sequence alignment


15606833_Aquifex_aeolic                    VILTRELEDIKKDKPLFEREGFKVLELPLIQTKVLEFNLEEFDYIVFQSK
10175667_Bacillus_halodu                   VLITRAKEQASSLSRLIEEAGGVPIEVPLLSYEEVGASIQSYEWIVFTSA
CM1920_Cyanidioschyzon_merolae             IALTRERGKNSKLATLVRRREARIYELPCIADRIVQLLADSIDWILITSP
145352552_Ostreococcus_lucima              VVCTRERGKNGALMNLLAARGARCVELPLVEAATFPDVLESWDWVCVTSP
20196944_Arabidopsis_thalia                VVVTRERGKNNQIIKALEKNGISSLELPLIQFDRLASVLKSFDWIIITSP
15807683_Deinococcus_radiod                VAVTRTRQTGSELAALLSGRGAQVSEIPLLRPRTLVGALNGFDWVLLTSE
6458497_Deinococcus_radiod                 VAVTRPDGGGGRLGELLRAQGAEVLSVPLIRPRALWSGLRGVDWLLVTSP
48895608_Trichodesmium_erythr              ILVTRSAGQSSKFSELLKKQGAKVIEMPALVWHDLDLGINSFDWLILTST
45510631_Anabaena_variab                   ILVTRSSGQSSQFSDRLTTLGATVIEMPALEWEELDQAISQFEWLILTST
48845542_Geobacter_metall                  ILVTRAADQAGEFGAMLGALGARVLECPTIAWDNLDAAIDRFHWAVFTSV
46579147_Desulfovibrio_vulgar              VVVTRAREQASGLAAALRELGADVIQFPTIDYAPVHEAIDTYDWVVFTSV
12655814_Selenomonas_rumina                ILVTRARSQASKLTAKLEKLGAEVLEAPAISYAALDKAIADYHWLIFTSA
108804799_Rubrobacter_xylanoph             VVVTRARAQAGELSRRLEELGAAVVEFPTIEFGPLDEAIDSFDWLVFTSV
153813575_Ruminococcus_obeum               VLVTRPKGRSSRTAEELRRRGAEVLELPSIRQSTLVHAFSSYQWIVFTSP
2127342_Clostridium_josui                  ILVTRPKESSGTLVEKLRQLGAEPVEYPCIENEKLYHACREYGWILLTSK
                                           :   **             .          . * :     .          :  . *

15606833_Aquifex_aeolic                    KAVKIFLGKVNKKIVAVGKKTAEELEKLGYKADFIPENESAKGLIELFKR
10175667_Bacillus_halodu                   NAVRFFFNTWPPSIAAVGSSTQRELSRYGISVDLMPNEYVAESLVETMVK
CM1920_Cyanidioschyzon_merolae             TAAEVFAQVVRPPIASVGAATNTALLKYSLQANFIPSRAIGACLGDELPV
145352552_Ostreococcus_lucima              EAAKVFLEAYEVRVAVVGAGTGKVLAKAEMDKQFTPSKATAATMAAELPC
20196944_Arabidopsis_thalia                EAGSVFLEAWKVQIGVVGAGTARVFEEAMLHVAFTPSKATGKVLASELPE
15807683_Deinococcus_radiod                QAVRALARTLLARIAAVGSGTAWTLREYGLAPDFVPTRSGSRHLGAELPA
6458497_Deinococcus_radiod                 QGGRLLGEALATRLAAVGEATARELRARGLPVDFLPTTATALSLGRKLPA
48895608_Trichodesmium_erythr              NGVDYFFERLAVKIAVVGKKTAESLKTCGLVPDFIPPNFVADSLVANFPE
45510631_Anabaena_variab                   NGVDYFFERLNVKIAVVGEKTSQSLKQRGIQADFIPPNFVADSLVEHFPE
48845542_Geobacter_metall                  NAVRFFFERLACRVCAVGPKTAAALAPFGIRPDLIPADYKGEGVVEVFRS
46579147_Desulfovibrio_vulgar              NGVKHFWNQLARKVAAIGPATADALRDKGIAPDFIPEKYVAEGVVEGMLA
12655814_Selenomonas_rumina                NGVGRFFARLFAKIAAIGLATAEKLKQYGLVADVIPQEYRAEGVLEALKG
108804799_Rubrobacter_xylanoph             NGVEAFLERLAARIAAIGPATAGRVREAGLRVDVVPREYRAEALLEEISG
153813575_Ruminococcus_obeum               TGVEIFFDELKAQIAAIGQGTAKVLEDRGILVDLIPEVYDGESLGEALAV
2127342_Clostridium_josui                  NGIQIFFDYLNTKIGTVGSQTAKALKEVGLISDFTPEIFDGRHLALGIAE
                                            .    :         :  :*   *    .          . *     .   :    :

15606833_Aquifex_aeolic                    KGKVLIPRSKIGRTELIKFLKETSFEVFALDVYTTEYVFYTREELLEK-D
10175667_Bacillus_halodu                   PCRVLFPKGNLARPTVKVGLETNGFIVDEMTVYETKRPLEAKVMLCEAIE
CM1920_Cyanidioschyzon_merolae             KVRILYPTSASASDEIEKRLRSRAVEFIRIDMYDTIEAKWTTEDIDTAVD
145352552_Ostreococcus_lucima              GSLILYPASKKAATTLQDGLEARGATVVRLNTYSTERVERLSPEDVAAAD
20196944_Arabidopsis_thalia                RSSVLYPASLKAGNDIVEGLSKRGFEVVRLNTYTTVPVQSVDTVLLQQAP
15807683_Deinococcus_radiod                GEVALHVGSQDTDAVLEMALSARGIEYVAAEAYRSELSELTPAQREQLAQ
6458497_Deinococcus_radiod                 GQAALHLTSQLSENTLRGELAARGVTYRRLELYHTAPAVLNAAERVALVS
48895608_Trichodesmium_erythr              GKKILFPRVETGREVLVKELNSQGAEVIEVAAYESSCPENILPEALLAID
45510631_Anabaena_variab                   NKKILFPRVESGREILVKELSAKGAEVIEVAAYQSCCPSSIPPAAELAID
48845542_Geobacter_metall                  GKRIIFPKGDRARDVIPQGLAELGGEVTAPVAYRNVTPDSLPAGVIAEIT
46579147_Desulfovibrio_vulgar              GKRVLLPRALEAREVLPEELRKAGATVDVLPVYVTVPSAARRDDVLARVH
12655814_Selenomonas_rumina                HAKILIPRAEEAREVLPDTLREMGAEVEVAPAYRTICGQVDGEALAAEID
108804799_Rubrobacter_xylanoph             GRRVLIPRAARAREVLPERLREAGAEVSVPPAYETVPSAEGREELARGVD
153813575_Ruminococcus_obeum               GEKILLPRARRGNQKLVKILKEQGGQVSDIPTYDTVYEKSSLIHIDREID
2127342_Clostridium_josui                  NEKVLICDAAIASDDIVNILRSNNIKFDRVPLYNTNYINENSNKVKKSLK
                                                :            :    *               * .

15606833_Aquifex_aeolic                    FIIFYSPSAVKAFFANLQSLKFVAIGKTTKEELQKHSVKNVITSERPSTE
10175667_Bacillus_halodu                   IITFTSPSAVDHFVTLLDHLLYAYIGPIAAARAKQRGLPVHIVAKTYTGE
CM1920_Cyanidioschyzon_merolae             LVTLASPSAVKVWAQRVGKQPAVCIGKTSADAAKEVGFSEVYAPSDPGLE
145352552_Ostreococcus_lucima              VVTFGSPSAVRAWVELCGQPAYVCIGETSAEACANCELPDVFYPELPGIE
20196944_Arabidopsis_thalia                VLSVASPSAVRAWLHLIQSNYVACIGETTASAARRLGLKNVYYPEKPGLE
15807683_Deinococcus_radiod                VVTLASAVGARALAQVAGSFTAAVIGPQTELAAREAGFTRLILAEQPTLA
6458497_Deinococcus_radiod                 AVTLASGSAAQGLAALAGRLPVAAIGEQTAAAAWTLGFVSVTTAPQPSLE
48895608_Trichodesmium_erythr              VITFASSKTVKNFCYLIGNICIASIGPQTSQSCHSLLGRVDVEAQEYTLD
45510631_Anabaena_variab                   IITFASSKTVQFFHQLVDGICIASIGPQTSKTCHTLLGRVDVEAEEYTLD
48845542_Geobacter_metall                  CVTFTSSSTVENLAAILGGVTIAAIGPITARTCRELGLEVHVEPSKYTLA
46579147_Desulfovibrio_vulgar              CVTFGSSSTVENFFSLVPEVKLACIGPVTKKTLEGFGFTCHIQPDDYTIP
12655814_Selenomonas_rumina                LVTFTSSSTVKNLVNIIGGVKTACIGPVTADTAKSLGIEPDIIAKEYTID
108804799_Rubrobacter_xylanoph             CVTFTASSTVENFVAAFGGTRVACIGPITAAAARERGLRVDVEAGEYTIE
153813575_Ruminococcus_obeum               CVVFTSASTVKGFVESTGKVTAACIGKQTKAAADDYGMQ-TYMSEKATID
2127342_Clostridium_josui                  YITFTSASTVEGFIASMKSLTAVCIGNKTAEAAKKYNLR-YVVAEKSTID
                                            : . :    .               . **   :                 .

15606833_Aquifex_aeolic                    DIVKKLKE
```

```
10175667_Bacillus_halodu          GLVNAICD
CM1920_Cyanidioschyzon_merolae    AWVDTLVR
145352552_Ostreococcus_lucima     GWAETVFL
20196944_Arabidopsis_thalia       GWVESIME
15807683_Deinococcus_radiod       SLSDAVQR
6458497_Deinococcus_radiod        GLVAAAIK
48895608_Trichodesmium_erythr     GLTKAIIE
45510631_Anabaena_variab          GLTQSIIN
48845542_Geobacter_metall         AMTDALVD
46579147_Desulfovibrio_vulgar     ALVDELTR
12655814_Selenomonas_rumina       GLVEAICK
108804799_Rubrobacter_xylanoph    GLVRAVAG
153813575_Ruminococcus_obeum      SLVELVVK
2127342_Clostridium_josui         SMIDKLLE
```

Note: This sequence has identifiable homologs only in green plants and bacteria (using *Cyanidioschyzon* and *Arabidopsis* GI 20196944 as queries). The top hits in GenBank searches are from firmicute and *Deinococcus* homologs. The *Arabidopsis* sequence (GenBank accession number NP_565625 and TAIR locus AT2G26540) is annotated as a chloroplast precursor, but appears not likely to be plastid-derived. Cyanobacterial sequences have a 1-aa insertion not shared by any other sequences.

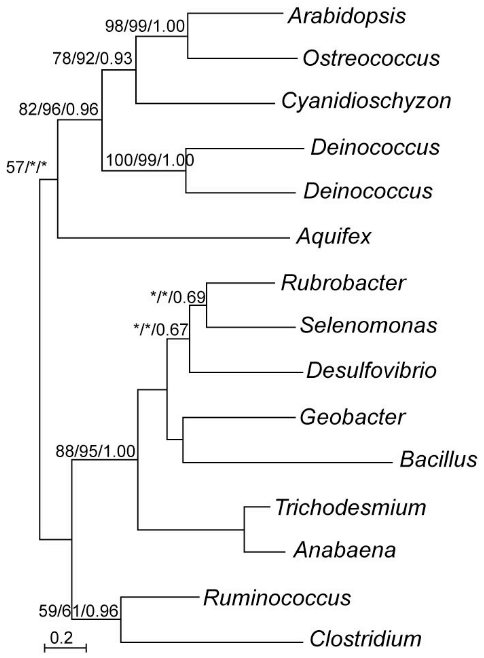

Figure 11. Molecular phylogeny of Uroporphyrinogen-III synthase. P-value = 0.959 from the AU test on the presented tree. AU tests were also performed on alternative topologies including (**A**) monophyly of red algal, green plant and cyanobacterial sequences, and (**B**) red algal, green plant and *Deinococcus* sequences forming a monophyly that in turn groups with cyanobacterial homologs. These tests investigate if a) red algae and green plants acquired this genes from plastids, and b) red algae and green plants acquired the genes from plastids and subsequently spread to *Deinococcus* by secondary HGT. P-value < 0.001 from AU test for topology **A** and p-value = 0.04 for topology **B**.

12. ACT-domain containing protein (N)

CLUSTAL X (1.83.1) multiple sequence alignment

```
113648684_Oryza_sativa                     VLIDQDSDRDATIVQLSFGDRLGALLDTMKALKDLGLDVTKGSV-STESA
9758449_Arabidopsis_thalia                 VMIDQDADPEATIVQLSFGNRLGALIDTMRALKDLGLDVIKGTV-STEGS
CM2077_Cyanidioschyzon_merolae             ----------ATRLVVTCRDRKGLLSDLTDALKSIGLQIRRAVA-RTKDG
88857979_Pseudoalteromonas_tun             IMVSEEPMHGGTQVFVYSQDEINLFARLVNALGSKKAHIHYAQVMSTKDG
46133622_Haemophilus_influe                VKISNRFSLGGTEVFIYCQDQPHLFNKVVSTIGTKKFSIHDAQIITTQDG
46143705_Actinobacillus_pleuro             VLVSNEYARGATEIFIYCEDQAQLFLRIAQILSQKKVSIHDAQIITSQNG
71546701_Syntrophobacter_fumar             WEVESSGDTA--EITIVSWEKPGLLSRCAGLLTLHSMNILGAQVFTMHNG
46449059_Desulfovibrio_vulgar              VVMEGRPVHGLWEVTILARDQQGLFATLAGVVALHGLNVYAADAFVWRDG
67985038_Kineococcus_radiot                ------------VVTVVTPDRTGVFADLAGLLAGHRF-LVRSALVRTLDG
58002716_Protein-PII]_uridyl               VEAYPIPERGVTELTVLCADHPGLFSQIAGALAVSGASIVDARIHTLSDG
103488264_Sphingopyxis_alaske              IAAVPDDDRGATLVMVLAADHPGLFYRIAGGIHLAGGNIIDARIHTTRDG
23012593_Magnetospirillum_magn             TTYETDPVRGVTELTVYSPDHPRLLAIITGACATTGGNIVDAQIFTTTDG
78697304_Bradyrhizobium_sp.                INVGFDPARGVTELTIFAMDHPWLLSIIAGACASAGANIVDAQIYTTTDG
86747159_Rhodopseudomonas_palu             VNVGFDEARGVTELTILAVDHPWLLSIIAGACASAGANIVDAQIYTTTDG
                                                           : :    :.    :                :  .        ..

113648684_Oryza_sativa                     VTQTKFHIMLEKIRLTVINNLLQYHVVTHVIVEDDGPKRSMLYIETADRP
9758449_Arabidopsis_thalia                 IKQTKFSITLEQIRLTIINNLLKYHIATHIHVKEDGPKRSLLVIETADRP
CM2077_Cyanidioschyzon_merolae             IASDEFFVTLDAVEQALQPVMGTSGRGVHVYVDNHASQHTTITVNAPDRP
88857979_Pseudoalteromonas_tun             YAIDNFVVLINSIRRSIEQAIKESGIKPQVIVRPHGRKDTLIEIQAVDIP
46133622_Haemophilus_influe                YVFDSFIITRRELEQALTVALQSEKVQTDVRFLHENKKETEMELVALDKP
46143705_Actinobacillus_pleuro             LVLDSFIVTCEQIKQSLEKVLNTSERQTKVRFLADSQQNTAFELFTLDRE
71546701_Syntrophobacter_fumar             LILLIFQCRWNAVRLDVERLLLGKMAPSQVLVDNQSSAMTILEVYTVDRV
46449059_Desulfovibrio_vulgar              TALDVFHVTWGKVRSSVQYAMTGKLRPAEVRVDNGLSDFTVIDVFAPDRP
67985038_Kineococcus_radiot                VAVDSWWVEAVLLRQGLERIVGGDVAHPRIVILPGASERTVLEVRAADRP
58002716_Protein-PII]_uridyl               MALDTFWVQLGRLNHLVEQALSGRLVPPRVVIDNTASDRTVIEVNGRDRP
103488264_Sphingopyxis_alaske              LALDNFLVQIARLTRAIEDALANRQVAPNVFVDNKASNRTVIEVNAQDRP
23012593_Magnetospirillum_magn             FALDSIFISAGRIATAIERALKGEIVPPDVSIDNALSSRTVVEITGLDRP
78697304_Bradyrhizobium_sp.                RALDTIAISATRIGETIEQVLEGKLVEPEVSINNQWSELTVIEVSGLDRP
86747159_Rhodopseudomonas_palu             RALDTISISATRIGEMIEEVLEGKLVEPEVSINNNWSDRTVIEVSGLDRP
                                                          :    :    :         : .         : . :    *

113648684_Oryza_sativa                     GLLLEIVKIITDVNVDVESAEIDTEGLVAKDKFHVS-YRGAKLNSSLSVL
9758449_Arabidopsis_thalia                 GLVVEMIKVMADVNIDVESAEIDTEGLVAKDKFHVS-YQGQALNRSLSVL
CM2077_Cyanidioschyzon_merolae             NLLNEIIDVLHELELNITFACLSTYADENKDIFHVTTMSGEQVDAVLREI
88857979_Pseudoalteromonas_tun             GLLTKIAEVFHSMALNIHAARITTVGERAEDFFVVSNNEFLALNDNEQSI
46133622_Haemophilus_influe                GLLAQVSQIFTELNLNLLNAKITTVGEKAEDFFILTNQFGQALDSQQRIL
46143705_Actinobacillus_pleuro             GLLARVSSVFNQLGLNLINAKITTIGERVEDFFVVTTQQHQALDDKAQAL
71546701_Syntrophobacter_fumar             GLLYTIGRTLFELQIRISVAKITTKIDQVADVFYVRTHQGEKVSDPEQEL
46449059_Desulfovibrio_vulgar              ALLYDVARTLQSLHLDVLFAKVSTLGNRTADTFSVRTAQGQKLTDEEHEV
67985038_Kineococcus_radiot                GLLHALGRALAEEGIDIRSAHVATYAAQAVDVLYLAEASGERLSPP-RVA
58002716_Protein-PII]_uridyl               GLLHDVTSALSSASLQISSAHITTYGMRAVDVFYVRDLLGMKITDPVRRL
103488264_Sphingopyxis_alaske              ALLNQLAYALFQSKVTVHSAHVATYGERAVDTFYVTDLIGDKIDSPARTL
23012593_Magnetospirillum_magn             GLLYELTTALNRLSLNITSAHVATFGERAVDVFYVTDLTGTRVMQPDRMI
78697304_Bradyrhizobium_sp.                GLLYELTTAISKLNLNIASAHVATFGERARDVFYVTDLLGAQINAPTRAI
86747159_Rhodopseudomonas_palu             GLLYQLTTAISKLNLNIASAHVATFGERARDVFYVTDLLGAQITAPTRAI
                                             *:   :   :     :  :  *  : *        * :  :        :

113648684_Oryza_sativa                     VNCLRYYL
9758449_Arabidopsis_thalia                 VNCLRYFL
CM2077_Cyanidioschyzon_merolae             MNTLYFLL
88857979_Pseudoalteromonas_tun             QNALIKRL
46133622_Haemophilus_influe                RNVLYRNI
46143705_Actinobacillus_pleuro             KSALLDEL
71546701_Syntrophobacter_fumar             KRALLFWL
46449059_Desulfovibrio_vulgar              RAALLHAV
67985038_Kineococcus_radiot                IRVLSDAA
58002716_Protein-PII]_uridyl               RETLLASL
103488264_Sphingopyxis_alaske              EKRLLEAA
23012593_Magnetospirillum_magn             RAAVMEVF
78697304_Bradyrhizobium_sp.                KSALLHLL
86747159_Rhodopseudomonas_palu             KRALVHLL
                                               :
```

Note: Identifiable homologs of this gene (using *Cyanidioschyzon* sequence and *Arabidopsis* GI 9758449 as queries) are restricted to red algae, green plants, proteobacteria and firmicutes with low sequence similarities (Evalue e-4 and lower). The annotation of the

Arabidopsis sequence (TAIR locus AT5G04740) indicates that the protein is located on the chloroplast thylakoid membrane.

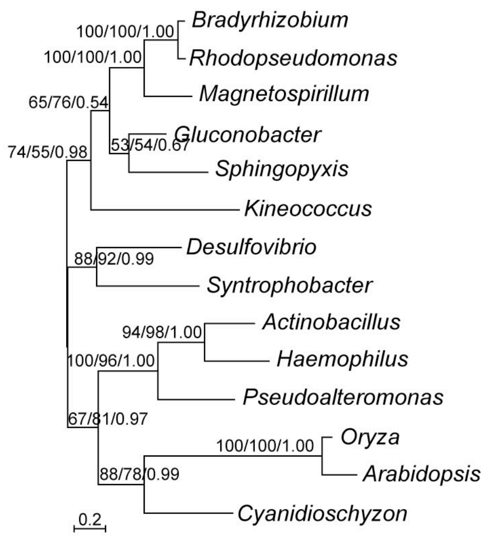

Figure 12. Molecular phylogeny of ACT-domain containing protein. Since no other eukaryotic or cyanobacterial homologs were identified, no statistical test on alternative topologies was given.

---

13. Queuine tRNA-ribosyltransferase (D)

```
CLUSTAL X (1.83.1) multiple sequence alignment


42520578_Wolbachia_endosy          SGSARVGTIKTPNGSVETPAFIFCATKAAIKAADIERISGTQIILSNTYH
58613533_Heterocapsa_trique        --------------------------------------------------
45656892_Leptospira_interr         LTKARTGILNLNGIELKTPVFMPVGTRGVVKTLSADDLEEYSLILGNTYH
19068923_Encephalitozoon_cunic     TTNARVSTLELNGTSLELPIFMPVGTYGAMKGIRVSDLE--SMILTNTYH
3881825_Caenorhabditis_elegan      AGFARRGNLHLPHSIVETPVFMPVGTQGTMKGIVPEQLVDCRILLCNTYH
56473322_Entamoeba_histol          DGRARQCTLTLPHGEVETPVFMPVGTKASVKSLTYEQVKGCQILLANTYH
46229743_Cryptosporidium_parvu     CGRARYGVLNLPHGRVDTPIFMPVATHGSIKGLSSLQVENVPILLGNAYH
TPO00008041_Trimastix_pyriform     --------------------------------------------------
12597314_Homo_sapien               RSRARAGELWLPHGTVATPVFMPVGTQATMKGITTEQLDGCRICLGNTYH
60463331_Dictyostelium_discoi      WGKARAAKLTLPHHQCSTPMFMPVGTQGTVKGLTSQQLVNCGVVLGNTYH
62360604_Trypanosoma_brucei        ---------------------MPVATQATLKGVTVEQLEDVEIILGNTYH
50900348_Oryza_sativa              FNRARAARLTLPHFTCQTPLFMPVGTQGTIKGLTTDQLEGCQIILGNTYH
JBO00061016_Jakoba_bahamensis      --------------------------------------------------
28850382_Dictyostelium_discoi      KSNARVTKITTPHGIIMQPNFVPVGTVGTIKFLDPISTKKSQLMFVNTYH
116059209_Ostreococcus_tauri       RSRARVGEIRTPHGVVRTPGFVSVGTNAALKAVHHETLDGLDLMFANTYH
CM2163_Cyanidioschyzon_merolae     RSSARIGRLHTPHGTVETPAFVAVGTNAALKMVDQRRADGLELLFCNTYH
46446428_Parachlamydia_sp.         KSRARVGRIHTPHGIIDTPNFVAVGTNGTVKALNNTMLHGLQLMFCNTYH
76788915_Chlamydia_trachomat       KSRARVGRIETAHGYIDTPAFVPVATNGALKGVLDH---NIPLMFCNTYH
29840339_Chlamydophila_caviae      KSRARVGKIETAHGIIDTPAFVPVATNGALKGVIDH---NIPLMFCNTYH
11499080_Archaeoglobus_fulgid      LDRMRVGKVKTRHGSFETPVFIPVATLAAIRGLDNRDLKGVEVILANTYH
21674218_Chlorobium_tepidu         HSAARCGVLSTSHGDIPTPIFMPVGTRASVKSVEPNELKDAKIILANTYH
48855206_Cytophaga_hutchi          GTSARAGVITTDHGTFETPIFMPVGTAGTVKAMHQRELEDAPIILGNTYH
53713592_Bacteroides_fragil        KSNARAGLITTDHGQIQTPIFMPVGTIGSVKGVHQTELKQAQIILGNTYH
76258051_Chloroflexus_auranti      ESRARAGLLQTAHGTVATPVFMPVGTRATVKSLTPHELRGASIILGNTYH
15644309_Thermotoga_mariti         FGKARLGVMKLHHGAVETPVFMPVGTNASVKLLTPRDLEGAEIILSNTFH
6460406_Deinococcus_radiod         DGRARTATFQTPRGAVTTPMFMPVGTQGTVKGISPQELLGSQMILANTYH
48891338_Trichodesmium_erythr      YTKARAGIFITPHGSINTPRFMPVGTLANVKTLTPAQLEGAQMILANTYH
56751072_Synechococcus_elonga      QTRGRACSFHTPHGIVETPRFMPVGTLATVKTVTPAQLRGAQMVLSNTYH
```

```
20455294_Nostoc_sp                 QTKARSGIFLTPHGIVETPRFMPVGTLANVKTVTPAQLKGAQMVLSNTYH
46106898_Rubrobacter_xylano        CGAARAGLLRTPHGAVETPTFMPVGTKGAVKGLSPRDLAGAGIVLGNTYH
42523694_Bdellovibrio_bacter       DGHARRATLMTAHGPVQTPVFMAVGTKATVKAMTPEELKGTQVVLGNTYH
15611336_Helicobacter_pylori       DDNARAGVLNLAHSQVETPVFMPVGTQGCIKSLDAMDAQGAKLILANTYH
15606515_Aquifex_aeolic            DGKARRGRIYTPHGVIETPVFMPVGTQGTVKAMLHKLLDGTQIILGNTYH
32397050_Rhodopirellula_baltic     DGGARRGVFHTPRGPVRTPGFMPVGTLGTVKGLTIDQVAGADMILGNTYH
39996277_Geobacter_sulfur          KTSARRGSLTTPHGTIETPIFMPVGTHAAMKAMTPSQVKGAQIILSNTYH
23473888_Desulfovibrio_desulf      DGKARTGVLQTAHGPVRTPIFMPVGTVGSVKGVAPDDLDGAEIILGNTYH
39997713_Geobacter_sulfur          DGAARLGSLTTPHGLIETPIFMPVGTQATVKAMTPEELDGARIILANTYH
52006768_Thiobacillus_denitr       DGGARRGQLHLAHGVVQTPVFMPVGTYGTVKAMSPTEIAGFEMLLSNTFH
53732897_Haemophilus_influe        SGSARRGRLVFPQGTVETPAFMPVGTYGTVKGMTPEEVRGAEILLGNTFH
48730213_Pseudomonas_fluore        DGKARRGRLTFPRGTVETPAFMPVGTYGTVKGMLPRDIVGAEIILGNTFH
19715156_Fusobacterium_nuclea      DGKARAGVITTLHGEIETPVFMPVGTQATVKTMSKEELLGSEIILGNTYH
56963323_Bacillus_clausi           QSGARLGRVHTPHGTIETPMFMPVGTLATVKTMSPEDLKDAQIILSNTYH
16800633_Listeria_innocu           QTGARLGKIHTPHGTFDTPMFMPVGTLATVKTMSPEELKGAGIILSNTYH
39935672_Rhodopseudomonas_palu     DGAARTGRLTTPHGVVRTPAFMPVGTAGAMKGLHWREVRGADIVLGNTYH
52011430_Silicibacter_sp.          DGKARTGVIHTPRGEIRTPAFMPVGTAATVKAMMPESVRGADILLGNTYH
15604558_Rickettsia_prowaz         YKKARSGVITTAHGEIRTPAFMPVGTRGAVKAMLTEAVVGADILLGNTYH


42520578_Wolbachia_endosy          LMLQPGENTVGLRKMIGWNGPMLTDSGGYQIFSINEDGAIFRSYINGKIY
58613533_Heterocapsa_trique        --------------------------------------------------
45656892_Leptospira_interr         LYLRPGTSVLGLKKFSTWKKALLTDSGGYQVFSYEQDGVRFQSHIDGSRH
19068923_Encephalitozoon_cunic     L---------NIKEFMKYNRGMLTDSGGFQIGSVTEDGVRFG------DS
3881825_Caenorhabditis_elegan      LGHRPGHERVGLHKMMNWNRSILTDSGGFQMVSVDENGVNFESPHTGEMM
56473322_Entamoeba_histol          MFLHPGVDVLGLHQFAKWDGNILTDSGGFQMVSVNEQGVIFQSIVDHKPI
46229743_Cryptosporidium_parvu     LGSRPGDEIIGLHNFMRWNRNILTDSGGFQMVSITEEGVEFRHPYTNANL
TPO00008041_Trimastix_pyriform     --------------------------------------------------
12597314_Homo_sapien               LGLRPGPELIGLHGFMNWPHNLLTDSGGFQMVSVTEEGVRFRSPYDGNET
60463331_Dictyostelium_discoi      LGHRPGPEVMGLHKFMNYPRAMLTDSGGFQMVSITEQGVQFQSPHDGSTM
62360604_Trypanosoma_brucei        LGLRPGEDILGIHFLQGWKRNILTDSGGFQMVSITEEGVRFQSTHGGGSL
50900348_Oryza_sativa              LELRPGSQLIGLHKFMNWKRALLTDSGGFQMVSITEEGVTFQSPVDGKPM
JBO00061016_Jakoba_bahamensis      -------------------------------------EGVRFQSPHDGSEM
28850382_Dictyostelium_discoi      MVVNSDPKLIGLHKFINYQNPIITDSGGFQVFSIKDSGVKFLSYKNGDTI
116059209_Ostreococcus_tauri       LMLQPGAETVGVHEMMGRRRPTITDSGGFQVFSVSEEGAMFRSYRDGTKM
CM2163_Cyanidioschyzon_merolae     LLLQPGPEIVGLHRFMQRSRPIMTDSGGFQVFSVSENGPVFRSYRDGRKI
46446428_Parachlamydia_sp.         MMLQPGTDIVGLHSFIQRKLPIITDSGGFQVFSITEEGVLFRSYRDGSKV
76788915_Chlamydia_trachomat       LIVHPGAEAIGLHQFIGRNAPIITDSGGFQIFSVNDDGVHFKSYRDGRKL
29840339_Chlamydophila_caviae      LLVHPGTEAIGLHKFMNRNAPIITDSGGFQIFSVNDEGVWFKSYRDGHKL
11499080_Archaeoglobus_fulgid      LHLRPGDELIGLHKFMNFDGVIVTDSGGFQAFSVTDRGVRFKDPKSGRIV
21674218_Chlorobium_tepidu         LYLKPGNDILGVHRFMNWDGPLLTDSGGYQVYSISEEGVMFKSHLDGSKL
48855206_Cytophaga_hutchi          LYLRPGLNIIGLHKFIGWKRSILTDSGGFQVYSINEEGVKFKSHIDGSAH
53713592_Bacteroides_fragil        LYLRPGLDVLGLHKFNGFDRPMLTDSGGFQVFSLHEEGAEFRSHIDGSKH
76258051_Chloroflexus_auranti      LYLQPGHELIGLHSFMGWNGPILTDSGGFQVFSVTEEAVIFKSYLDGSRH
15644309_Thermotoga_mariti         LMLKPGVEIIGLHNFMGWKRPILTDSGGFQVFSIDDEGVVFRSPIDGSKV
6460406_Deinococcus_radiod         LMLRPGEQLVGLPGFTAYPGPFLTDSGGFQVMSISEEGVVFKNHLDGSRV
48891338_Trichodesmium_erythr      LHLQPGESIVGLHKFMAWNRPILTDSGGFQVFSISEAGVKFRSPHDGSMI
56751072_Synechococcus_elonga      LHLQPGEEIVGLHRFMNWSGPMLTDSGGFQVFSIEERGVTFRSPRDGAVI
20455294_Nostoc_sp                 LHLQPGEAIVGLHKFMGWNGPMLTDSGGFQVFSITEEGVTFRSPRDGQII
46106898_Rubrobacter_xylano        LYLRPGAEVVGLHEFTGWSGPMLTDSGGYQVFSLSEEGVRFTSVYDGSVH
42523694_Bdellovibrio_bacter       LHLRPGEKTIGLHKFMNWHGPILTDSGGFQVFSMTEEGVEFRSHLDGAKH
15611336_Helicobacter_pylori       MYLRPGEKVVGLHRFAQFQGSFLTDSGGFQAFSLQEDGIVFKSHIDGSKH
15606515_Aquifex_aeolic            LYLRPGTEIIGLHRFISWNKPILTDSGGYQVFSVSDEGVEFQDHLQGDKH
32397050_Rhodopirellula_baltic     LRLRPGHETVGLHKMCGWDGPILTDSGGFQVFSVNEHAATFRSHIDGAKI
39996277_Geobacter_sulfur          LHLRPGEDLIGLHRFMGWDGPILTDSGGFQVFSITDEGVFFRHEVSGEEV
23473888_Desulfovibrio_desulf      LYLRPGDELVGLHEFNAWRKPILTDSGGFQVFSISEEGVEFRSHLDGSKH
39997713_Geobacter_sulfur          LYIRPGHELIGLHRFMHWDRPILTDSGGFQVFSISEEGVRFQSHLDGSYH
52006768_Thiobacillus_denitr       LWLRPGLEVIGLHRFMGWDKPILTDSGGFQVFSITEEGVKFASPTNGDKL
53732897_Haemophilus_influe        LWLRPGQEIMDLHDFMQWHRPILTDSGGFQVFSITEEGVKFQNPINGERI
48730213_Pseudomonas_fluore        LWLRPGTEVIDLHDFMQWKGPILTDSGGFQVFSIKEEGVTFASPVDGSKV
19715156_Fusobacterium_nuclea      LYLRPNDELIGLHKFMNWDRPILTDSGGFQVFSIKEEGVYFSSHIDGSKH
56963323_Bacillus_clausi           LWLRPGEEIIGLHQFMNWDKPILTDSGGFQVFSITEEGVHFRNHLSGEKL
16800633_Listeria_innocu           LWLRPGEELIGLHKFMNWDQPILTDSGGFQVFSIKEEGVHFRNHLNGDKL
39935672_Rhodopseudomonas_palu     LMLRPGAERIGLQRFTTWNGPMLTDSGGFQVMSVNEQAVTFRSHIDGAAI
52011430_Silicibacter_sp.          LMLRPTAERIGLHKFMNWDRPILTDSGGFQVMSLTEKGVTFKSHIDGSRH
15604558_Rickettsia_prowaz         LMLQPSAERIGLHKFMNWDKPILTDSGGFQVMSITEEGVSFRSHINGNKY


42520578_Wolbachia_endosy          CLTPEKSVQIQQKLGADLILVLDECTPFHVSKEYTAKSMLMSHRWAERSL
58613533_Heterocapsa_trique        --SPESSIVIQRELGADLILVLDECTPFHVDKAYTARAMRRSHRWAVRSL
45656892_Leptospira_interr         YFTPNSVIDIQRSIGSDIMMVLDDCAPFDSGPERLKQSLDRTHRWAEMSV
19068923_Encephalitozoon_cunic     LFTPEDSMNIQMMLGADIIMQLDDVVNPCDTREKIEIAMRRSIRWMDRCI
3881825_Caenorhabditis_elegan      ALPPEKSIEIQQALGADIMMQLDHVIHVLTTGDIVKEAMHRSIRWLDRCK
```

```
56473322_Entamoeba_histol           NLPPEESIHIQQAIGSDIMMQLDDVVSSLTTGPRVEEAMERSVRWLARCK
46229743_Cryptosporidium_parvu      LFTPEKSIQVQNAIGADIIMQLDDVITAKSTDQRFDEAVDRTTRWLDRCI
TPO00008041_Trimastix_pyriform      ----------------------------------PRVEEAMHRTIRWLDRCY
12597314_Homo_sapien                LLSPEKSVQIQNALGSDIIMQLDDVVSSTVTGPRVEEAMYRSIRWLDRCI
60463331_Dictyostelium_discoi       VLTPELSMGIQNSIGADIMMALDDVVSSTTVGPRVEQAMYRTLRWIDRCI
62360604_Trypanosoma_brucei         LLKPEDSIRIQNAIGGDIMMQLDDVVHVLTTGPRVEEAMRRTIRWLDRSL
50900348_Oryza_sativa               LLTPEESIHIQNNIGADIIMALDDVVKTTITGPRIEEAMYRTLRWIDRCI
JBO00061016_Jakoba_bahamensis       LLRPEDSMAIQNRLGADIMMALDDVVSSTTSGPRVEEATQRTLRWIDRCI
28850382_Dictyostelium_discoi       ELGPVSSIKYQKQLGADIIIPFDELPPYHMDEKKILRSLHRTHHWEATSL
116059209_Ostreococcus_tauri        TLTPESSVAAQKAIGADIIIPLDELPPYDIDDNALRESVARSHRWMTRSL
CM2163_Cyanidioschyzon_merolae      ELTPEKSVQAQKMLGADIIIPLDELPPYHIDRDALYQSVLLSHRWEARSL
46446428_Parachlamydia_sp.          LLTPESSIKAQKDLGADIIIPFDELPPYHIARDALKKSLDRTHRWEKRSL
76788915_Chlamydia_trachomat        FLSPEISVQAQKDLGADIILPLDELLPFHADPTYFHQSSQRTYVWEKRSL
29840339_Chlamydophila_caviae       FLSPEVSVQAQKDLGADIIIPLDELLPFHSDEKYFLSSCSRTYVWEKRSL
11499080_Archaeoglobus_fulgid       ELTPKKSMEIQSNLGSDIIFAFDECTSPLSDRDYTEKALERTHRWAEECL
21674218_Chlorobium_tepidu          HFTPENVVDTQRIIGSDIMMPLDECPPWPAEKEYVQKSGELTLRWAERAR
48855206_Cytophaga_hutchi           FFTPESVMDIQRTIGADIVMAFDECTPYPCEYEYARKSMEMTHRWLGRCV
53713592_Bacteroides_fragil         IFTPEKVMDIERIIGADIMMAFDECPPGDSDYAYAKKSLGLTHRWLDRCI
76258051_Chloroflexus_auranti       VFTPERSIEVQHHLGADIIVCFDELPPFRAGYDYTAQSMARTHRWAERCL
15644309_Thermotoga_mariti          FLNPEISMEVQIALGSDICMVFDHCPVPDADYEEVKEATERTYRWALRSK
6460406_Deinococcus_radiod          ELTPERSIQVQEALGADVIMAFDECPPYPAERPYIEASLDRTVRWLERCH
48891338_Trichodesmium_erythr       DLTPERSIQIQNELGGDVIMAFDECPPYPASWEEVELATNRTYRWLQRCI
56751072_Synechococcus_elonga       EFTPERSIRIQNALGADVIMAFDECPPYPAERKDVEAAVARTYRWLERCI
20455294_Nostoc_sp                  KLTPERSIEIQNILGADVIMAFDECPPYPANRQEVEAATERTYRWLERCI
46106898_Rubrobacter_xylano         AFTPELAVRVQEALGADVAMVLDECPPAEADRAYHVSSLRRTARWAARCK
42523694_Bdellovibrio_bacter        FISPEKSMEIQMDLGSDIIMAFDECLKYPATDEEIEKSMALTYRWLLRSQ
15611336_Helicobacter_pylori        LFTPIKVLDIQYSLNSDIMMVLDDLVGLPAPLKRLEESIKRSAKWANMSL
15606515_Aquifex_aeolic             FFTPEKVVEIQEIFGSDIMMPLDECVEYPVDKNYAEKALKRTINWLERSI
32397050_Rhodopirellula_baltic      ELTPEHSIEIQQALGSDVAMVLDHVIALPAPMTQVEDALARSIRWAARCR
39996277_Geobacter_sulfur           FLDPARATAIQEALGADIIMAFDECIPYPCDKKYAAASTRKTIRWAEACK
23473888_Desulfovibrio_desulf       LFTPEKVVSIQRNLNSDIMMVLDECVPYGADKDYTARSLKMTTRWAQRCR
39997713_Geobacter_sulfur           TIRPEDAVAIQEALGSDIAMCFDECTPYPATHDYARRSMELTTRWARRCK
52006768_Thiobacillus_denitr        FLTPETSMQIQRTLNSDIVMIFDECTPYPATERQAADSMRMSLRWAARSK
53732897_Haemophilus_influe         FLSPEKSMEIQYDLGSDIVMIFDECTPYPATFDYAKKSMEMSLRWAKRSR
48730213_Pseudomonas_fluore         FMGPEESMQVQRDLGSDIVMIFDECTPYPADEDVARVSMELSLRWAQRSK
19715156_Fusobacterium_nuclea       FISPEKSIQIQNNLGSDIVMLFDECPPGLSTREYIIPSIERTTRWAKRCV
56963323_Bacillus_clausi            FLSPEKAMQIQHVLGSDIMMAFDECPPYPADHSYMKASVERTSRWAERCL
16800633_Listeria_innocu            FLSPEKAIQIQNALGSDIMMSFDECPPYPASHEYMKKSVERTSRWAERGL
39935672_Rhodopseudomonas_palu      ELSPERAIEVQRLLGSDIAMQLDECVRLPADRADIERAMQLSLRWAERCK
52011430_Silicibacter_sp.           ELTPERSMEIQRLLGSDIVMCFDECPALPADRDRIAESMRLSMRWAERSR
15604558_Rickettsia_prowaz          MLTPEYSTEIQYLLGSTITMALDECTPYPSTFEKAKTSMHLTTRWANRSR
                                                                           :   :  *

42520578_Wolbachia_endosy           NEFEKQALYGISQGGVYQDLRRESCDFINDLPFFGQAIGG-SLGQSKEQM
58613533_Heterocapsa_trique         TEFAAQALYGIIQGGVHEDLRAESVAFVNRQPFFGTAIGG-SLGADRATM
45656892_Leptospira_interr          QYWEKQHLFGIFQGGIDLDFRLESLNTITSLPFDGIAIGGLSVGEPRKDF
19068923_Encephalitozoon_cunic      MAVNDQILFPIIQGGLDEGLRAESIAEILKRSPKGLAIGGLSGGEEKSEF
3881825_Caenorhabditis_elegan       VAHTRQAMFPILQGGLNLELRKECAKEMAKRAKVGIAIGGLSGGEEKDHF
56473322_Entamoeba_histol           NEYLKQNLFGIIQGGLQPNLRERCLEGMVKVDTPGYAIGGLSGGEAKDDF
46229743_Cryptosporidium_parvu      KAHKKQNLFAIVQGGIFKDLRERSLKSLKERNTPGYAIGGLSGGESKDSF
TPO00008041_Trimastix_pyriform      AAHKRQNLFAIVQGGLDPRLRTMCMAEMKKRDIPGYAIGGLSGGEDKNLF
12597314_Homo_sapien                AAHQRQNLFAIIQGGLDADLRATCLEEMTKRDVPGFAIGGLSGGESKSQF
60463331_Dictyostelium_discoi       KAHKKQNIFAIVQGGLDSRLRDICMEGLMAREFPGYAIGGLSGGESKDMF
62360604_Trypanosoma_brucei         AANANQGIFGIVQGGLDSGLRSICMEEMVKRKCKGYAIGGLSGGEAKEQF
50900348_Oryza_sativa               AAHKKQNLFGIVQGGLDPVLRDICVKGLVERNLPGYAIGGLAGGEDKDSF
JBO00061016_Jakoba_bahamensis       QAHKRQNLFGIVQGGLDAELRRYCAEELVRRDLPGYAIGGLSGGEAKDDF
28850382_Dictyostelium_discoi       LEHIKQAIYSVIHGGVNIEMRKQSIDYLTSLPFDGIALGG-SLGKDRNEM
116059209_Ostreococcus_tauri        RAHLSQAMYGVVHGGVDRDLRRASVEYLSALPFDGMAIGG-SLGRDSTEL
CM2163_Cyanidioschyzon_merolae      REHLHQAMYAVIHGGIDLELRRKSVEYLTALPFDGIAIGG-SLGKDREEL
46446428_Parachlamydia_sp.          EAHLKQAMYAVVHGGIDPDLRKESCAILTELPFDGFAVGG-SMGKTKDEM
76788915_Chlamydia_trachomat        DYHLKQSMYGVIHGGTFPDQRKLGCKFVEDLPFDGSAIGG-SLGKNLQDI
29840339_Chlamydophila_caviae       DYHKKQSMYGVIHGGIDPEQRKIGCQFVEDHPFDGFAIGG-SLGRNLQEM
11499080_Archaeoglobus_fulgid       QHYDRQALFGVVQGGEYRDLREKSARFMAERDFAGYGIGG-SLGKSKQDM
21674218_Chlorobium_tepidu          KHFLSQFQFGITQGGTFDDLRAHSSRALVDMDFDGYAVGGMAVGEPAEEM
48855206_Cytophaga_hutchi           TRMAEQHLFPIVQGSVYKDLRLKSAEVIAEAGCVGNAIGGLSVGEPAEEM
53713592_Bacteroides_fragil         QRFNEQSLFPIVQGCVYPDLRKQSAEYIASKDADGNAIGGLAVGEPVDKM
76258051_Chloroflexus_auranti       IAHQRQLLFGIVHGGIFPDLRRASACYISDMAFDGLCIGG-SLGANKTQM
15644309_Thermotoga_mariti          KAFKTQALFGIVQGGIYPDLRRESALQLTSIGFDGYAIGGLSIGEERSLT
6460406_Deinococcus_radiod          AVKTKQALFAIVQGGVHEDLRLKSLEATLPFATPGFAVGGLAVGESKEEM
48891338_Trichodesmium_erythr       EAHQRQALFGIVQGGVYPELRSIAARQLVDLDLPGYAIGGVSVGEPGELI
56751072_Synechococcus_elonga       NAHERQALFGIVQGGVYLDLRQQAARDLVQLDLPGYAIGGVSVGEPSEEI
20455294_Nostoc_sp                  TAHQRQALFGIVQGGVYLDLRAKAANTLTELDLPGYAIGGVSVGEPPEMM
46106898_Rubrobacter_xylano         KAHSRQALFGIVQGGLYPDLREESLRRTLELDFDGYAVGGLSVGEPRERM
```

```
42523694_Bdellovibrio_bacter        AAMTRSLLFGIVQGGLSLKHRLKSMEQICSVDLPGYALGGFSVGEPMHLM
15611336_Helicobacter_pylori        EYHKENNLFAIIQGGTHLKMRSLSV-GLTHEGFDGYAIGGLAVGESVDEM
15606515_Aquifex_aeolic             KAKKRQVLFGIVQGAFWKDLRKKAVEETLKFDLFGYSIGGLSVGEPKEIM
32397050_Rhodopirellula_baltic      IAADRQALFAIVQGGLDRTLRQQCASELAAMSFEGYAVGGLSVGEPPEDM
39996277_Geobacter_sulfur           KAHTRQALFGIVQGSVFEDLRAQCARELVQLDFPGYAVGGVSVGEGLELL
23473888_Desulfovibrio_desulf       DAYPANLMFGITQGGFFKDLREESIGELTRIDFDGFALGGLSVGESKTEM
39997713_Geobacter_sulfur           DARRRQALFGIVQGGMYRDLREQSAAELVEIGFDGYAVGGLSVGEEKEHM
52006768_Thiobacillus_denitr        AAHAGNALYGIVQGGMYEALRDESARELIGMDFDGYAIGGLSVGEPKDDM
53732897_Haemophilus_influe         DRFDENALFGIIQGGVFEELRKVSLEGLVNIGFDGYAVGGLAVGEPKEDM
48730213_Pseudomonas_fluore         NAHGDAALFGIVQGGMHQDLRMRSLEGLDKIGFDGLAIGGLSVGEPKHEM
19715156_Fusobacterium_nuclea       EAHQKQGLFAIVQGGIYEDLRQKSLDELSEMDFSGYAIGGLAVGEPREDM
56963323_Bacillus_clausi            AEHKRQGLFGIIQGGEYEDLRKQSANDLTALDFPGYAVGGLSVGEPKDVM
16800633_Listeria_innocu            KAHERQGLFGIVQGGAYEDLRAQSAKDLVSLDFPGYSIGGLSVGEPKDVM
39935672_Rhodopseudomonas_palu      RAFETYMLFGIVQGGDVPELRHASAQGLIDIGFHGYAIGGLAVGEPQDVM
52011430_Silicibacter_sp.           EAFGDHALFGIQQGGLEQDFREESAEALTKIGFDGYAVGGLAVGEGQEAM
15604558_Rickettsia_prowaz          DAFVKYAQFGIIQGSVYKELREQSVKDLVKCDFEGYAIGGLAVGEGQELM
                                            : : :*      *             *  :** : *

42520578_Wolbachia_endosy           HDVVSFTMDHLKKDRPTHLLGIGGIVDIFRGVELGIDTFDCVHPTRLARH
58613533_Heterocapsa_trique         HAVVGYTAARCRPDRPIHLLGIGGIRDIFHGVRCGIDTFDCVHPTRLGRH
45656892_Leptospira_interr          IRILDGISAHTDRNRPLYLMGVGTVPDILDGVKNGVDMFDCVLPTRNARN
19068923_Encephalitozoon_cunic      ARIVHFCTKSLPPDIPRYLMGVGYPEDIVVCVALGSDMSDCVYPTRTARF
3881825_Caenorhabditis_elegan       WRVVAACCAALPPHLPRYVMGVGFPVDLVICSFLGADMFDCVYPTRTARF
56473322_Entamoeba_histol           WKMVDISAQGLPENKPRYLMGVGYPVEMLLCVLFGVDMFDCVYPTRTARF
46229743_Cryptosporidium_parvu      WQIIELCTRSLPENKPRYVMGVGYPIDILVCVALGADMFDCVYPCRTARF
TPO00008041_Trimastix_pyriform      WRVVHQCTSELPQNKPRYLMGVGYPLDLLVCSALGVDMFDCVYPCRTARF
12597314_Homo_sapien                WRMVALSTSRLPKDKPRYLMGVGYATDLVVCVALGCDMFDCVFPTRTARF
60463331_Dictyostelium_discoi       WRVVHQCTSKLPENKPRYLMGVGYALDLVVCSALGVDMFDCVFPSRTARF
62360604_Trypanosoma_brucei         WAIVAQCCKTLPNDKPRYCMGVGYPEDIIVCIALGVDMFDCVYACRTARF
50900348_Oryza_sativa               WRVVAQCTAGLPEDKPRYVMGVGYPLDIVVCSALGADMYDCVYPTRTARF
JBO00061016_Jakoba_bahamensis       WRMVSVSTDVLPRNKPRYCMGVGYPLDLVVCVALGVDMFDCVYPTRTARF
28850382_Dictyostelium_discoi       KELLKTIIPFIPKNKPNHLLGIGDLESITSIIPLGIDSFDSSYPTRSARH
116059209_Ostreococcus_tauri        GSLLEFLMPLMPKDMPNHLLGISDVENVDRAVMLGVDTFDSAYTTQVARH
CM2163_Cyanidioschyzon_merolae      LALLAFLDTLLPKDRPVHLLGIADEESIRRAVPYGIDTFDSCYPTRAGRH
46446428_Parachlamydia_sp.          HTLLSLTLPLLPEDKPNHLLGIGDLPSIERSVPLGIDTFDSSYPTRAARH
76788915_Chlamydia_trachomat        VEVVGVTAANLSAERPRHLLGIGDLPSIWATVGFGIDSFDSSYPTKAARH
29840339_Chlamydophila_caviae       LPVVDVTTSYLSKDRPVHLLGIGDLPSIHATVGLGIDSFDSSYPTKAARH
11499080_Archaeoglobus_fulgid       LNILDWVIPLLPEEKPRHLLGIGAIEDLFNCTEKGVDMYDCVAPARWARR
21674218_Chlorobium_tepidu          YRMLELSHTILPESKPRYLMGVGTPANILNAIERGIDMFDCVIPTREGRN
48855206_Cytophaga_hutchi           YAMSELVCSVLPKDKPRYLMGVGTPENLLENIALGIDMFDCVMPTRNARN
53713592_Bacteroides_fragil         YEMIELVNEILPKDKPRYLMGVGTPVNILEGIERGVDMFDCVMPTRNGRN
76258051_Chloroflexus_auranti       YEVVDMTVPYLPDGMARHLLGVGDVDDLLEGVARGIDMFDCVSPTRLGRH
15644309_Thermotoga_mariti          LEMTEVTEVFLPEDKPRYFMGGGSPELILELVDRGVDMFDSVFPTRIARH
6460406_Deinococcus_radiod          YPAVAFTAGRLPENKPRYLMGVGHPEDLVAGVALGIDMFDCVYPTRTGRF
48891338_Trichodesmium_erythr       DHIVKVTTPFLPEDKPRYLMGVGTYREMVRAIAEGIDLFDCVIPTRLGRH
56751072_Synechococcus_elonga       HRIVEATAPLLPAHKPRYLMGVGTYREMVQAIAAGIDLFDCVIPTRLARH
20455294_Nostoc_sp                  AQIVQATAPLLPAHKPRYLMGVGTYREMVIAIASGIDLFDCVIPTRWARH
46106898_Rubrobacter_xylano         LEILSLLTPRLPEDRPRYFMGIGDPVGILEVIALGVDMFDCVLPTRLARH
42523694_Bdellovibrio_bacter        HALLPDVAPRMPANKPRYLMGVGTPTDLIIAIDSGVDMFDCVMPTRVARN
15611336_Helicobacter_pylori        LETIAHTAPLLPKDKPRYLMGVGTPENILDAISLGVDMFDCVMPTRNARN
15606515_Aquifex_aeolic             YGMTEVVCELLPEKKPRYLMGVGKPEDILEAVERGVDMFDCVVPTRNART
32397050_Rhodopirellula_baltic      YTTTGFTTPHLPADKPRYLMGVGTPRDLLENIARGIDLFDCVMPTRNGRN
39996277_Geobacter_sulfur           KKVVEYTAPFLPENKPRYLMGVGLPEDILESVERGMDMFDCVIPTRYARS
23473888_Desulfovibrio_desulf       MDILYHTAPMLPAHKPRYLMGVGTPLDIINGINAGIDMFDCVLPTRNARN
39997713_Geobacter_sulfur           YGVMEWTAPLLPVDQPRYVMGIGTPEDLVEAVWRGFDMFDCVMPTRNARN
52006768_Thiobacillus_denitr        TRILAHTAPQLPADKPRYLMGVGTPSDLVAAVAAGIDQFDCVLPTRNARH
53732897_Haemophilus_influe         HRILEYICPQIPADKPRYLMGVGKPEDLVEGVRRGIDMFDCVMPTRNARN
48730213_Pseudomonas_fluore         IKVLDYLPGMMPADKPRYLMGVGKPEDLVEGVRRGVDMFDCVMPTRNARN
19715156_Fusobacterium_nuclea       YRILDYIVEKCPEEKPRYLMGVGEPVDMLNAVESGIDMMDCVQPTRLARH
56963323_Bacillus_clausi            NHVLEFTTPLMPEDKPRYLMGVGSPDSLIDGAIRGIDMFDCVLPTRIARN
16800633_Listeria_innocu            NRVLEHTTPLLPANKPRYLMGVGSPDSLIDGVIRGVDMFDCVLPTRIARN
39935672_Rhodopseudomonas_palu      LAMIEEAAPILPAERPRYLMGVGTPEDMLEAVARSIDMFDCVMPTRNGRH
52011430_Silicibacter_sp.           FGCLDYAPDMLPVDKPRYLMGVGKPDDIVGAVSRGIDMMDCVLPSRSGRT
15604558_Rickettsia_prowaz          FRVLDYVPDFLPQNKPRYLMGVGKPSDIIGAVSRGIDMFDCVIPTRSGRN
                                                   . : :* .    :      . *  *.  . : .*

42520578_Wolbachia_endosy           GGALHINLRNQRFELDNNPIESDCLCFTCRKHSRAYIHHLLKAKELLTLV
58613533_Heterocapsa_trique         GGALHIHLEKARFRDDIRPIDSTCGCYTCRNFSRGYLHHLLRAQETLTLA
45656892_Leptospira_interr          GQVFKINLRNEKWKSSDTPMDPNCTCKVCKRYSIGYIRHLHHVGEITSLS
19068923_Encephalitozoon_cunic      GKGLDVCF-TSKLKSDKRKIDETCGCYTCSKYSRAFLFALKGTTNF-MLL
3881825_Caenorhabditis_elegan       GTAMLMQLNQKRYKEDFLPIDKKCECNTCKNYTRAYIHSIVG-KETVHLV
56473322_Entamoeba_histol           GTAFLVTLKINKYKDDFNVIDESCDCLCCKGNTKSALHFMFTNGAVDQYL
46229743_Cryptosporidium_parvu      GTAMLLKLKLSKYKDDARPIDSRCKCYCCKHYSRAALYRIVLKDSLAQLM
```

```
TPO00008041_Trimastix_pyriform      GTALVIHLRSDKFALSKEPIDAQRHCMVCHEYTMGRLHQLACHE--GELI
12597314_Homo_sapien                GSALNLQLRKKVFEKDFGPIDPECTCPTCQKHSRAFLHALLHSDNTAHHL
60463331_Dictyostelium_discoi       GTALSLNLKSSEYAFDFTPIDSECTCMVCKNYTKAYLHIVAGKEAIGQLL
62360604_Trypanosoma_brucei         GSALKIQVSKKVYANDMGPLDPECNCMTCKTFTRAYL-HIVAAKESITLL
50900348_Oryza_sativa               GTALVLKLKQNAMATDERPIDPTCPCMVCKNYTRAYLHCLVTKDAMGQLL
JBO00061016_Jakoba_bahamensis       GTALQLDLKNPRFADDATPLCVEFSWHG-RHYSRSYLHRLMLEKEEAA--
28850382_Dictyostelium_discoi       GQIILIKNQENRIKID-EPLDKSCDCHTCKNYSKAYIHHLFKSHEQITLA
116059209_Ostreococcus_tauri        GTLFRINFRRAEFRRSREPACHECDCTLCSKHSLGYLHHLDRANEPLTLA
CM2163_Cyanidioschyzon_merolae      GTLLRIQIKSRKWERVFEPVDTTCEGFVSQNHTLAYLHHLWRAHEPLALL
46446428_Parachlamydia_sp.          GILLPLNITKSEYATNFNSIEKGCLCPACHHFSLAYIHHLFKARELTSLA
76788915_Chlamydia_trachomat        GMILPLKINNQRYSSDLNPIEPGCSCLACQGITRAYLRHLFKVHEPNIWA
29840339_Chlamydophila_caviae       GLILPIKIANQAYANDLSSLDPECTCATCSNISRAYLRHLFKVHEPNIWA
11499080_Archaeoglobus_fulgid       GHLYRIHIKNAAFRIDNRPVDRTCDCLVCQNYSRAYLRHLYKANELLRLA
21674218_Chlorobium_tepidu          GRVYTINLRAGKYADDFRPIDEGFDNHVCRNYSRAYIRHLLNVGEILKLC
48855206_Cytophaga_hutchi           GMLFIINIRNKKWENDFTPLDQGLDIYLNDYYTKAYVRHLVHNNEMLQIA
53713592_Bacteroides_fragil         GMLFIMNMRNKKWEADFSPIEADGASYVDTLYSKAYLRHLFHAQELLQIA
76258051_Chloroflexus_auranti       GAALRLNVTNAALRDDRQPLQEGCPCYTCRHFSRAYIHHLYRSKELLRLV
15644309_Thermotoga_mariti          GTALKLNLKASYNKRSLEPVDERCGCYTCKNFTRSYIHHLFDRGEVLILL
6460406_Deinococcus_radiod          GYALRLNLNSSAPRTQLQPIDAECDCYACRHYTRAYLAHLLRAEEMLRML
48891338_Trichodesmium_erythr       GAALRWNLKNARFKEDFRPLDEFCPCYCCQNFSRAYLSHLVRAKEALTLL
56751072_Synechococcus_elonga       GAALRWNLKNAQFREDFQPLDEDCNCYCCQNFSRAYLNHLIRSREILTLL
20455294_Nostoc_sp                  GTAMRWNLKNAKFREDFAPIDETCPCYACQNFSRAYISHLVRSQEILTLL
46106898_Rubrobacter_xylano         GTALRLNLKNARYRRDFGPLDPGCPCEACAGFSRAYLAHLLRENELLRLV
42523694_Bdellovibrio_bacter        GTIYKVSIKRSEYKEDPSALDPECDCYTCKNYSKSYLRHMFMSGEILRLN
15611336_Helicobacter_pylori        ATLFKISIKNAPYKLDDTPIEENCACYACKRYSKAYLHHLFRAKELTRLA
15606515_Aquifex_aeolic             GTLYVVDIRHSKWKEDFSPLDPECDCYTCRNFSKAYLRHLFVAEEISVLN
32397050_Rhodopirellula_baltic      ALAFPLKLRNAVHKLDTRPLMEDCPCLACRH-SRGYLRHLFVAGEMLILL
39996277_Geobacter_sulfur           ATLFKIRLTHRNYRRDFYPVDPNCTCYTCRNFTRAYLHHLFNANEILTLA
23473888_Desulfovibrio_desulf       GTLYKINIKRREFAEDDGPLDPNCSCYTCRTFSRAYLRHLFHAQEILRLN
39997713_Geobacter_sulfur           GMLFRLNIKGAAYAEDQGPVDPACGCYVCRNYSRAYLRHLYRSGEILRLN
52006768_Thiobacillus_denitr        GILFEIRIRNARWKLDTAPIDEECDCYACRHFTRAYVHHLIRAGEILRLT
53732897_Haemophilus_influe         GHLFIVKIRNAKYRDDTNPLDPECDCYTCKNYTKAYLYHLDKCGEILRLN
48730213_Pseudomonas_fluore         GHLFVLKIRNAFHRHDDSPLDPTCDCYTCQNFSRAYLHHLDKCGEMLMLN
19715156_Fusobacterium_nuclea       GTVFRLVIKSERYKEDTKPLDEECDCYVCKNYSRAYIRHLIKVQEVLRLT
56963323_Bacillus_clausi            GTCMRLVVRNAKYARDFSPLDEKCDCHVCRTYSRAYIRHLIKCEETFRLT
16800633_Listeria_innocu            GTCMRLVIKNAKFTHDFRPIDENCDCYTCKNYSRAYIRHLIRCEETFRLT
39935672_Rhodopseudomonas_palu      GLAFPINLRNAKHADDPRPLDEESDWPSARTYSRAYLHHLVRSSETLMLL
52011430_Silicibacter_sp.           GQAFVVNIKNARHQDDPRPLDENCSCPACSNYSRAYLHHVFRSNEMIMLL
15604558_Rickettsia_prowaz          GQAFTVNIRNSKYADDKEPLEHDCKCPACTNYTKAYLHHLVRIREILMLM
                                    .                              : . :  :

42520578_Wolbachia_endosy           TIHNIFFMNKLMASIRQAILDDRL
58613533_Heterocapsa_trique         SLHNVYFVNRMMAAIRVAIRNGTL
45656892_Leptospira_interr          TYHNLHFMKNFLTEIQNSIQKGEF
19068923_Encephalitozoon_cunic      TIHNLYYMRSLTRRIRESITEDRY
3881825_Caenorhabditis_elegan       SVHNIKHQLDLMRDVRQAIQSNSV
56473322_Entamoeba_histol           THHNISYLFNLMRKYRVAIREGKS
46229743_Cryptosporidium_parvu      TIHNISFMMEFCNDMRNAIKNQTF
TPO00008041_Trimastix_pyriform      TMHNIAYQMRHMQRIRDAIKGGTF
12597314_Homo_sapien                TVHNIAYQLQLMSAVRTSIVEKRF
60463331_Dictyostelium_discoi       TFHNIHYQMSLMSQIRQSIIDQTF
62360604_Trypanosoma_brucei         SYHNLAYLINLTRGAREAILSGTF
50900348_Oryza_sativa               SYHNLSFMMRLSRDLHMSILEGRF
JBO00061016_Jakoba_bahamensis       ------------------------
28850382_Dictyostelium_discoi       TQHNLRAMSRLMENYRNKILQNEV
116059209_Ostreococcus_tauri        SEHNLFYMGQKMARVRERILNDEI
CM2163_Cyanidioschyzon_merolae      TLHNIKYMCDVMREIRTRILADEL
46446428_Parachlamydia_sp.          TVHNLYFMVQLMEKYRKQIQEDLI
76788915_Chlamydia_trachomat        SIHNMHHMQKVMREIREGILNDRI
29840339_Chlamydophila_caviae       SIHNLHYMQEVMKNIREQILNDEI
11499080_Archaeoglobus_fulgid       TYHNIYFVVKLMERIRESIADGSF
21674218_Chlorobium_tepidu          TMQNLSFYLWLTRTAREHIAAGDF
48855206_Cytophaga_hutchi           TIQNISFYLWLMREARKQIVAGTF
53713592_Bacteroides_fragil         SIHNLAFYLWLVGEARKHIIAGDF
76258051_Chloroflexus_auranti       SLHNVAFLLNLMADIRSALAAGRF
15644309_Thermotoga_mariti          TIHNINFMISLMKEVRRSIESGTF
6460406_Deinococcus_radiod          SLHNLRYLHRLVERMRVAINGQQF
48891338_Trichodesmium_erythr       SLHNVTELISFTQRIRDAILKDRF
56751072_Synechococcus_elonga       SIHNITELVRFTTRIREAILSDRF
20455294_Nostoc_sp                  SIHNITELIRFTQKIREAILSDRF
46106898_Rubrobacter_xylano         SLHNVRFVTELCRSARREILAGTY
42523694_Bdellovibrio_bacter        TIHNIHFYMKVMEKAREAIAQGRW
15611336_Helicobacter_pylori        SLHNLHFYLELVKNARNAILEKRF
```

```
15606515_Aquifex_aeolic            TIHNLRFYLKMMEEVRKAIEEKRF
32397050_Rhodopirellula_baltic     SHHNLMYYGRLMQATRDAIEAGEF
39996277_Geobacter_sulfur          SIHNVHFYLNMMAEIRAAIEEERF
23473888_Desulfovibrio_desulf      SIHNLTYFLDLVRGAREAIAQGTF
39997713_Geobacter_sulfur          TYHNLAYYLDLMAQIRTAIAEERF
52006768_Thiobacillus_denitr       TLHNLHYYHRLMAEVRAAIDAQRF
53732897_Haemophilus_influe        TIHNLRYYQRLMAEIRQAIEDDRF
48730213_Pseudomonas_fluore        TIHNLRHYQVLMAGLREAIQQGTL
19715156_Fusobacterium_nuclea      SYHNLYFLIKLMKDAREAIKEKRF
56963323_Bacillus_clausi           TYHNLYFLLNLMKQVRQAIMDDCL
16800633_Listeria_innocu           TYHNLHFLLNLMKQVRSAIMEDRL
39935672_Rhodopseudomonas_palu     SEINIAYYQRLMRDIRAAIAVGQF
52011430_Silicibacter_sp.          TWHNLHYFQDIMAGMRESIAAGTF
15604558_Rickettsia_prowaz         TWHNLTYFQNLMSRIRTYIKLGKD
```

Note: This is an intriguing case of ancient HGT. The *Dictyostelium* sequence forms a group with homologs of green and red algae as well as chlamydiae. This specific affiliation of *Dictyostelium* and plant sequences has been observed in multiple cases (Huang, unpublished data), likely resulting from plant-*Dictyostelium* transfer. A plausible explanation is that primary photosynthetic eukaryotes acquired this gene from chlamydiae and then further spread to *Dictyostelium* via secondary gene transfer. *Heterocapsa* sequence is a chloroplast precursor based on the original GenBank annotation. *Heterocapsa* and *Wolbachia* sequences also share indels and many conserved residues. The remaining eukaryotic sequences are much more similar to bacterial than to archaeal homologs, and they are likely of bacterial origin. One possible explanation is that they are derived from mitochondria. Nevertheless, most of these eukaryotic sequences lack a N-terminal extension. Sequences of *Trimastix* and *Jakoba* were obtained from TBestDB.

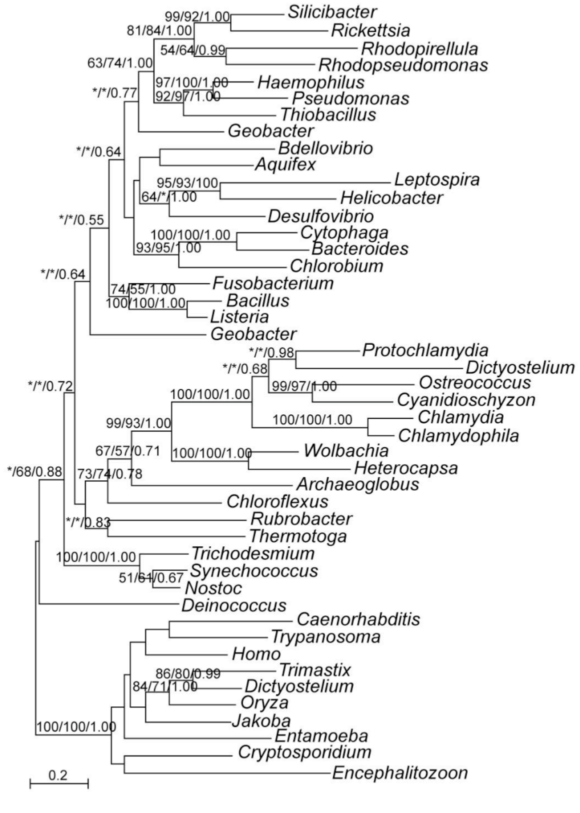

Figure 13. Molecular phylogeny of queuine tRNA-ribosyltransferase. P-value = 0.369 from the AU test for the presented gene tree. AU tests were also performed on alternative topologies including (**A**) monophyly of all eukaryotic sequences, and (**B**) monophyly of red algal, green

plant, *Dictyostelium*, and cyanobacterial sequences. These tests investigate if all eukaryotic sequences have the same origin (mitochondrial or eukaryotic) and if red algal and green plant sequences are likely derived from plastids. P-values < 0.001 from AU tests for both alternative topologies.

---

14. SAM-dependent methyltransferase (N)

CLUSTAL X (1.83.1) multiple sequence alignment

```
23509703_Plasmodium_falcip          INGFPWLYSKDIEYNENIGVGIYNRNSIISSRLLEFFNIRIRKAYEKRLE
16080887_Bacillus_subtil            KKGYPLIEKEALSGGEFLARGYYGLQNKGVGWTLAFFLSKLTKAAQARAT
29375834_Enterococcus_faecal        KKGYPLIQKEDLSKGQRLAEGYLGEQNKGIGWLLSFFQQLFEISREKRTT
54294134_Legionella_pneumo          LRGHPWIFPKAISEGESLGFGAYNEHSLYRVRVLSLITHRIKQASLVRRT
42571865_Arabidopsis_thalia         KDGSPMVYSGAVGTENPIGWGLYNSVSMFCVRLMKLLQTRIAEAVQLRKT
CM2295_Cyanidioschyzon_merolae      RQVRPLIFGRAITPGPVLGYGFYNPDSMYRVRLIRVLTRLFTAAWRLREH
42522494_Bdellovibrio_bacter        KQGHRWLFANCFYKGEALALGIWQGDTQLRFRVLLYFENQWRKAVEIRKT
14600479_Aeropyrum_pernix           ARGSSIIYRKWVRNGSEIACGLWEENTPVAVRIIEALERALEAAYKARVF
42524664_Bdellovibrio_bacter        RSGHPWVFSNELSKGQFLARGYGNPHSLISFRALDFLHEKIFNSWRVRKR
39998234_Geobacter_sulfur           QLGHPWIIADAYASGRFLATALLDPGERIVARVLRWLAQRLEDALELRRT
34763641_Fusobacterium_nuclea       LNFYPNIYKDEISDMKFLARGYITEGTSAFVRILKFIFERIKNAYEKRKT
6457710_Deinococcus_radiod          RDGHPWVYESSVRRDRFLAIGLYDPFSPLRLRVLAWWAARLDAALARRAT
32444786_Rhodopirellula_baltic      LSRHPWVMANALYDGNWIARGLINPASRLRIRLYSMWAERIEQAVARRRQ
21110809_Xanthomonas_axonop         RSSHPWIFQKLVMDGEWIGRGFYNGHSRIAVRILGWFSRKIAAAVSLRRS
15837009_Xylella_fastid             RSSHPWLFQKLLVDGEWVGRGFYNGHSRIAVRILAWFVRRIAEAVGLRRS
53797508_Chloroflexus_aurant        QQGHPWVYRNHLRCGGLTVYGLYDEQSPIGLRIYSWFRERVWEAWELRAT
22960169_Rhodobacter_sphaer         RHGFPWVYADELAERRALGLVTVNVSSKIIARMLGWFAERLTRALALREQ
56679104_Silicibacter_pomero        RHGAPWVFDNEVDNRAPLGLVAVNPGSRILARMLAWFEARLTRALALREA
15605998_Aquifex_aeolic             KHFYPWVFRDEVSEGNFLALGTYSPYSRIAFRVLEFFKRRLRESELLRRS
34556476_Wolinella_succin           RTLPAWIYGSQLPKQEFLALGYINPKSQIALRILEELKKRLQKAILKRET
46446677_Parachlamydia_sp.          RNYHHWIFSGAVSSHQLLGYGYFNRRSGIVGRMVETLKKRLISAWKFRQT
53712322_Bacteroides_fragil         KRFHPWIFSGAISKKEFIAKGHFQIGS-IAVRVLDFWKHKLEVAYDMRRN
48854326_Cytophaga_hutchi           NNRHPWIFSGAVFENKKLGYGFLSHKSQIVCRMFNYWAVKLKNAFALRQT
20094503_Methanopyrus_kandle        RSGALSVFAPAVATGDFLGYAFAQTSDRIVARIFEIVLERIERRYRFRKR
14591657_Pyrococcus_horiko          GKGAMIVFKKGVRGGKFLGKGFANPNSNIMVRIVDLFKRRIKKANEYRKT
15607037_Aquifex_aeolic             RGFFPWVYRSEIVNGKFLGYGYINPEVNIAIRILELIRKRIKQAYEYRKS
53796614_Chloroflexus_aurant        IQRHPWIFSGAIKDGSWLARGFWSSTSQLRVRLATWLRETIARAVAGREA
26246990_Escherichia_coli           LRRHPWVFSGAVHQGKWLARGAYSPASQIRARVWAFFTRRLQQAQKWRDL
29142267_Salmonella_enteri          LRRHPWVFSGAVHQGKWLARGAWSPASQIRARVWAFFTRRLRQAQQWRDL
47573089_Rubrivivax_gelati          LRRHPWVFEGSIHDGSFLAWGAYSPASQIRVRAWSLFARRVERAVALRAS
48770381_Ralstonia_metall           LRRHPWVYATGIADGRFLARGAYSPESQIRARVWAMFKRRVSAALAHRKT
48786339_Burkholderia_fungor        LRRHPWVYANAIHDGRFLARAAYSPHSQIRARVWAFFKRRVQRALAHRQT
21674038_Chlorobium_tepidu          VSGHLWVFSNELHDGRLLGAGFFNPQSLIAFRLLDFFRRKLLEALKLRET
15644445_Thermotoga_mariti          SGGHLWIFYNEIPDGSFFGKGYINDNSKIRVRILNFIKNRIESALKRKKT
39997720_Geobacter_sulfur           RGGHPWVFSNEITGGGFLGTGYYNPRSLIAARLLDFFLERIGRALALRRM
46199218_Thermus_thermo             LSRHLWVFRRDVGR-RFLALALYNPHTDLAVRAYAALLENLAQALARREG
48857855_Clostridium_thermo         KDGHPWIYGEEIAGNAFMGTGFYNSVSKITVRLIRFWRRRVEYAVRYRKF
37523934_Gloeobacter_violac         RRTHPWIFASEVAQGRFCGRGFLNRQSQIAVRYLAWWIERFGKALEHRHV
51894272_Symbiobacterium_therm      QAGHPWIFQGEVFRGRLLGKGYINPASQILVRMLEFFRRRLERAWAYRQT
                                          :                                        :

23509703_Plasmodium_falcip          NFFRVVNAESDFLPGIIIDKYNKLVCVQINASGMDIIIIIKNDNIEVQEN
16080887_Bacillus_subtil            TAFRLFNGEGDGVGGVTIDYYDGYLLIQWYSKGIYTAIYEKKRFIIIQEN
29375834_Enterococcus_faecal        TAYRLFNGEGDGIGGLIIDRYADYAVFSWYNETLYQGAYEKIRFLLVTEN
54294134_Legionella_pneumo          NAYRLFNSEADGLSGLTIDCFNDYCVIASSAYWVELHIIWLPQNTQVLEE
42571865_Arabidopsis_thalia         NAYRLVNSEGDRLSGLIVDVFGDIAVVASSAAWLEKHINWRPSLSMVVEN
CM2295_Cyanidioschyzon_merolae      AACRLINGEGDRVSGLCIDLYARYLVVQSSARWVEHELVWRSNSTWITEH
42522494_Bdellovibrio_bacter        NSFRLINGEGDGLPGLIVDVYNDTAVIKHDHPIMEKCVYLKRRNVQFLEN
14600479_Aeropyrum_pernix           DSYRLINSDGDRLSGLIADVYKDVAVVQSSSPAIDRHVYEKSVQVVIEEG
42524664_Bdellovibrio_bacter        GSFRLAFGESDFIPGLVLDYYAQLFAAQLVTAGMNEAVVIRNDVIVLNAA
39998234_Geobacter_sulfur           DAYRLVNGEGDGLPGITVDRYGDYLMVQLYCGGWRPGIYEKTRPLPVREN
34763641_Fusobacterium_nuclea       NSVRAFYSEADFIPGLIIDKFDKYVSIQFRNSGVEVGIYERSDVTIMVDN
6457710_Deinococcus_radiod          DGYRVLNGESDGFPGLVVDRYAGVLVMKLYTAAWFPVVLRLSRNVVFRES
32444786_Rhodopirellula_baltic      GGERLIFSESDRMSGLIVDRYADCLSIQITGGALIPKVLVRMDDVWYQHN
21110809_Xanthomonas_axonop         DAWRVVHSEGDGLSGLVVDRYGDLVVVEFFAAGMFRRFHSFADEAVITEH
15837009_Xylella_fastid             DAWRVVHSEGDGLSGLVVDRYGDLLVVQFFAAGMFRRFHSFADEVVITEH
53797508_Chloroflexus_aurant        TAYRWIYGEGDNLPGLVVDRYGDYAVIQTYADSVQTGVIQRPRQLVVQEH
```

```
22960169_Rhodobacter_sphaer                PFYRLVHAEADGLPGVVIDRFGEAAVIQPNAAWAETTVVKNGTGVPVPMN
56679104_Silicibacter_pomero               PFYRLVHAEADGFPGVIIDRFGEACVIQPNAAWAEATVLKNASGVPVLMN
15605998_Aquifex_aeolic                    NAYRLAFSESDLLSGLIIDRYGDAFVIQVRSYPMEIFVYERSDFLIIEER
34556476_Wolinella_succin                  NGARLIHSESDELPGLIVDSYANHLSLQINTAGMERSIIDKSDEILLQEE
46446677_Parachlamydia_sp.                 NAYRFVHGEGDAIPGLTIDVYQDVFVLQSSTKGIDESIFEKSILISFFEN
53712322_Bacteroides_fragil                NTYRLVHGEGDNLPGLVIDVYARTAVMQAHSAGMHVNIYYKSETIA-QEY
48854326_Cytophaga_hutchi                  NVYRLLHAEGDFFPGIIADVYADVVVLQVLIRGVEKHIYIKAKTVIVKEN
20094503_Methanopyrus_kandle               DHMRVVFSEADDVPGLILDKFNDIAVFQTTCPAIERTLYEKNDSTVVEEY
14591657_Pyrococcus_horiko                 NVYRMVYGEADYLPGLIVDRFNDIASLQISSAGMERTVFEKNTGTIIQEG
15607037_Aquifex_aeolic                    NAYRLVHSEGDLLPGLIVDVYGSYLAVEFTTYGMNQGIYEKVNEVIIWEH
53796614_Chloroflexus_aurant               IACRLVFSESDGLPGLIVDRYGCYLVIQLLTQAMAVGIYERSDAITALDQ
26246990_Escherichia_coli                  DSYRLIAGESDGLPGITIDRFGNFLVLQLLSAGAEYAIYDRSDVLPIEEH
29142267_Salmonella_enteri                 DSYRLIAGESDGLPGVTIDRFGHFLVLQLLSAGAEYAIYDRSDVLPIEEH
47573089_Rubrivivax_gelati                 DAQRLVHGEADGLPGLIVDRYGDTLSAQFLGAGVERRLYERSDALTIREH
48770381_Ralstonia_metall                  NAVRLIFGESDRLPGLIVDYYRGQLVCQFNAAGVEQNVYERSDALSVTEH
48786339_Burkholderia_fungor               GAVRLIFGEADGLPGLIVDHYRSQLVCQFMAAGVEANVYERSDVLIVSEN
21674038_Chlorobium_tepidu                 NAWRLVHGESDGLPGLVIDRFDRAFVLQSFSAGIDQAIVVRNESQEIRDS
15644445_Thermotoga_mariti                 TAFRVVHSEGDFLPGLIVDLFGDYLVFQITTLGMEKGIYEKSEGIEFEMN
39997720_Geobacter_sulfur                  ETFRLVHGEGDFLPGLVIDKYGEWLSIQLLTAGMDAGIVARNDVLEIEEH
46199218_Thermus_thermo                    GGYRLVHAEGDLLPGLVVDYYAGHAVVQATAHAWEGSVLAKNDAVQVREG
48857855_Clostridium_thermo                ACCRLIHGEADHMPGLTVDRYGSLLSVQITCLGMELGIYERNDITEICEN
37523934_Gloeobacter_violac                KTFRWIHGEADGLPGLTVDRYGDFVVVQLLALGLEPGIFERSDAVEIEEG
51894272_Symbiobacterium_therm             GACRVVFGEADFLPGLIVDKFGDILVVQTLALGIDKGIYERNDVITIVEN
                                              *   .:.* . *:  * :

23509703_Plasmodium_falcip                 GTFFVDILKGQKTGWYYDQRYNRTMLISYCKNVLDLFSYVGSFGITLAYY
16080887_Bacillus_subtil                   GQYAVDLNEGAMTGIFLDQRHVRKAIRDRYKTVLNTFSYTGAFSVAAALG
29375834_Enterococcus_faecal               GQFATYLNEGLMTGIFLDQKEVRGRLVDGFKTVLNMFSYTGAFSVAAAMG
54294134_Legionella_pneumo                 GLYHVEFAQTQKTGLFLDQRENHKRIAKLSKRVLDLYTFTGGFALHAAKA
42571865_Arabidopsis_thalia                GSYAISL-EGQKTGFYTDQRENRHFISTISKRVLDLCCYSGGFALNAARG
CM2295_Cyanidioschyzon_merolae             GMYEVDIMHGQKTGHYCDQRDNRLFIRHLVQRVLDLCTYTGGFALNAVLG
42522494_Bdellovibrio_bacter               GLFASNIRDAAKTGFFLDQRDNRKMIQHFAKTVLNLFSYTGGFSIFAAKG
14600479_Aeropyrum_pernix                  QRFIVDVERGQKTGFFLDQRVNRLELARVADSVLDVFSYTGGFGIHALLS
42524664_Bdellovibrio_bacter               GTMSCDLVEGQKTGFFLDQTHNKNWAKTQQIRVLDLCCYVGHWSTQITRG
39998234_Geobacter_sulfur                  GTFLVELERGLNTGLFLDQRANRRQLMARTKRVLNLFAYTGAFSVAAAAG
34763641_Fusobacterium_nuclea              GKYNIDIVDGQKTGFFLDQRDSRKFIAKYITRFLDVFSSSGGFSMAALKN
6457710_Deinococcus_radiod                 GRFEAEVRQGQKTGFFLDQRENRRRVEGLSRRVLNAFSFSGGFSLYAARG
32444786_Rhodopirellula_baltic             GEMAIDLQDGQKTGGYLDQQLNHAAAASYMRRVLDICTYTGGFALAAARA
21110809_Xanthomonas_axonop                GKFRADPAGAHKTGFFADQRDNRQWLSEQVKSVLDLCCNTGGFAVYAAAR
15837009_Xylella_fastid                    GKFLVDPMGAHKTGFFADQRENRQWLSQGVKTVLDLCCNTGGFAVYAALR
53797508_Chloroflexus_aurant               GFFQVDILYGQKTGLFLDQRDNRRTLEGLVARVLNCFAYTGGFSLYALRG
22960169_Rhodobacter_sphaer                GTYMADLTGGQKTGFFLDQRPNHAFAARLGGSVLDVFTHVGGFALAALAG
56679104_Silicibacter_pomero               GTYMADLTGGQKTGLFYDQRPNHAFAARLATRVLDVFSHVGGFALAMLAG
15605998_Aquifex_aeolic                    EKFLVDVVEGLKTGFYLDQRDNREYVRNLVDRVLDLFCYSGGFSVYCANR
34556476_Wolinella_succin                  GRFGINLLEGQKTGYYLDQRQNRLLLAEMAKRVLDLFCNGGGFGIHALLK
46446677_Parachlamydia_sp.                 GKFTTNLHKSQKTGFFLDHREMRQWIKTLSKKVLNAFSYTGGFSVYAMAG
53712322_Bacteroides_fragil                GKFHVDWLKGQKTGFFVDQRENRALLERYARSVLNMFCYTGGFSFYAMRG
48854326_Cytophaga_hutchi                  DLFEVDFIDGQKTGFFVDQRENRELLRSYSKKVLNTFCYTGGFSVYAASG
20094503_Methanopyrus_kandle               GRFAVNVEEGQKTGFYIDQVENRAKVQELVGRVLDVFTYVGGFAIHAAVG
14591657_Pyrococcus_horiko                 RKFIVDM-RGQKTGFFLDQRENRLALEKWVDRVLDVFTYTGGFAIHAAIA
15607037_Aquifex_aeolic                    DKYYINIPQGQKTGFFLDQRNARKFVRNLVDRCLDVFCHTGGFALNMKRA
53796614_Chloroflexus_aurant               PRMFADLQTGQKTGIYLDQAINHLRVGAYCADTLDCFSYAGGFALAAALA
26246990_Escherichia_coli                  GKLLVDIQHGHKTGYYLDQRDSRLATRRYVKRVLNCFSYTGGFAVSALMG
29142267_Salmonella_enteri                 GKLLVDIQGGHKTGYYLDQRDSRLVTRRYVQRVLNCFSYTGGFAVSALMG
47573089_Rubrivivax_gelati                 GQLKVDVAEGHKTGYYLDQRDNRLRFMQLVRRVLNCYSYTGGFSVAALAG
48770381_Ralstonia_metall                  GRYHVDVRNGHKTGFYVDQRDNRKLVGDLAREVLNCFCYTGGFSLAALRG
48786339_Burkholderia_fungor               DRYHVDVRNGHKTGFYVDQRDNRPLVQQLARDMLNCFCYTGGFSLAALKG
21674038_Chlorobium_tepidu                 GSYRVNILEGQKTGFFLDQRENRRHIRKYAADVLDVYTNDGGFALNAMHA
15644445_Thermotoga_mariti                 GKFLAD-TRGQKTGFFLDQRENARMVMDLAKVCLDVFSYTGNFAVHLLKG
39997720_Geobacter_sulfur                  GRFRVDIMGGQKTGHFLDQKENHLLLKGISKRVLDCFSYSGSWGIHAAAF
46199218_Thermus_thermo                    RRYLVDLRAGQKTGAYLDQRENRLYMERF-ERALDVFSYAGGFALHLAL-
48857855_Clostridium_thermo                GKYLVDVENGQKTGFFLDQKYNRAAVARIAKRVLDCFTHTGSFGLNAALG
37523934_Gloeobacter_violac                SRLLVDIQQGQKTGLFLDQRFNRQHAARFARNVLNAFGYTGAFGVQCALA
51894272_Symbiobacterium_therm             GEIAVDVAEGQKTGYYLDQRENRHAVRRYAGRVLDCFCNVGGFALNAAAA
                                                       ** : *:              *:     * :.

23509703_Plasmodium_falcip                 GAKEVTCVDSSYSAIQNGLHAAQYNNVKTYDVILIDPPPLARNNYCLPSA
16080887_Bacillus_subtil                   GAEKTTSVDVANRSLAKTIEQFSVNKLLRFDLIILDPPSFARTKKRTFSA
29375834_Enterococcus_faecal               GAVATTSVDLAKRSLPKTTEQFEVNHLLSYDMIILDPPSFARNKKKVFSV
54294134_Legionella_pneumo                 GALHVTAVDSSAQAIEQAKNNAVLNHLGEYDVVILDPPKLVPSQKHLQQA
42571865_Arabidopsis_thalia                GATSVIGIDSSLPALELARENVILNNMETWDIVILDPPKLAPRKKVLHNA
CM2295_Cyanidioschyzon_merolae             GALHVTAVDSSGQALMQAHRNAERNQVQRYDLVVLDPPKLAPTVESLPRA
42522494_Bdellovibrio_bacter               GAKEVTSVDIAKVAIQAVARNFEINDLKKYDIVITDPPSFAPNEKSVEQA
```

```
14600479_Aeropyrum_pernix          GASKAVFIEEDAHAVKLLRKNLRLNGIESFDIVTVDPPAFIPGQEAKKRG
42524664_Bdellovibrio_bacter       FEVEVSLADISKTALAFAKKNAEREGATHYDIVIADPPAFIKSKKDIPIG
39998234_Geobacter_sulfur          GASRVTSVDASPTYTDWAKAHFEANRLERFDIVLMDPPSFSTTSKSRFTT
34763641_Fusobacterium_nuclea      GAKEVVAMDKDSHALELCYENYKLNEFKKFDIITLDPPSLIKKKTEIYKG
6457710_Deinococcus_radiod         GASEVTSLDISAHALRSAERNFALNPEADYDLVILDPPSLARREAEREGA
32444786_Rhodopirellula_baltic     GAKEVVAVDSSERALDIAKRNAERNQLEQFDAIILDPPRFAGSRNQTNAA
21110809_Xanthomonas_axonop        GAAEVLGVDIDEDVIAIAKGNAKLNNVEQFDVVILDPAKMTRDREQVIPA
15837009_Xylella_fastid            GAADVVGVDIDQDVIQIAKANAKLNSVDQYDVVILDPAKMTRDRDQIINA
53797508_Chloroflexus_aurant       HAAEVVSCDIGRGLAEATATNIALNRLQTFDVVILDPPSFARTRTSQHAA
22960169_Rhodobacter_sphaer        GAERALAVDASGPALALAEAGAKLAGLARFDLVICDPPAFAPAKPALEAG
56679104_Silicibacter_pomero       GAGSALAVDGSAPALALAEQGALASGLETFDVVICDPPAFAPSKQALDAG
15605998_Aquifex_aeolic            GAK-VVGVDINKRAVELARENAKLNSVEEWDLIIADPPAIAKTKKEKESI
34556476_Wolinella_succin          GAKEALFIDSSEMACEQVKKNLSLNQLEKFDLIILDPPSFAKTKEQQKGA
46446677_Parachlamydia_sp.         GAKRVDSVDISQEAVNACQKHFVLNELLDYDLVILDPPAFAKKQKDVIAA
53712322_Bacteroides_fragil        GAKQVHSVDSSAKAIDLTNKNVELNFPDQYDLIILDPPAFAKHKDALRNA
48854326_Cytophaga_hutchi          GAEVVDSVDISKEAVALAGKNMELNYPDYYDIIVLDPPAFAKSAKAVANA
20094503_Methanopyrus_kandle       GAEEVVGIDKFDRVVQAAYRNADLNDVEEFDVVVLDPPAFVTSKEHLNRG
14591657_Pyrococcus_horiko         GADEVIGIDKSPRAIETAKENAKLNGVEKFDIVVLDPPAFVQHEKDLKAG
15607037_Aquifex_aeolic            GAGEVIGVDISELALKEAEKNARLNGFEKFDVIIIDPPSFAKNRAAVPNA
53796614_Chloroflexus_aurant       GARQLTLVDASAEALGLAADNLRLNNLRQFDVVILDPPKFVHQQAHLQRA
26246990_Escherichia_coli          GCSQVVSVDTSHEALDIARQNVELNKLEKFDVIVMDPPKFVENKSQLMGA
29142267_Salmonella_enteri         GCRQVVSVDTSQDALDIARQNVELNQLEKFDVIIMDPPKFVENKSQLMGA
47573089_Rubrivivax_gelati         GAQQVISVDSSGPALARASEHVQLNGFASFDAIVLDPPKFAPTAAHAERA
48770381_Ralstonia_metall          GATSVTSIDSSGEALKIAASNVTLNGFRQFDLIVLDPPKFAPSAQHIDRA
48786339_Burkholderia_fungor       GAKRVVSIDSSGDALAIAQQNVAANGFERFDLVVLDPPKFAPSREHVDRA
21674038_Chlorobium_tepidu         GAKSTTMVDISQEALQRAEQNARTNGFHTFDLVILDPPSFTKSRKTVPTA
15644445_Thermotoga_mariti         GAKHVTLVDYSERALEVAREILKLNGFRKYDLVVLDPPSFAKSSSNLESA
39997720_Geobacter_sulfur          GAAETLCLDVSERAAALVMANARLNGLRRFGVVVLDPPAFVKSKKALKEA
46199218_Thermus_thermo            GFREVVAVDSSAEALRRAEENARLNGLERFDLVVLDPPAFAKGKKDVERA
48857855_Clostridium_thermo        GAEHVTCVDISQSAIDMAKANAVRNGLHDYDYIILDPPAFTKSRKTVQSA
37523934_Gloeobacter_violac        GASSVLNVDIAEAATAIAEQNAQLNGVRRFEMIILDPPAFAKSRSVLEGA
51894272_Symbiobacterium_therm     GAREVVAVDASAEAIAAAEENAARNGLERFDVIVLDPPAFAKNKAALEGA
                                           :                    :  :  **. :

23509703_Plasmodium_falcip         LKIYEKLLYVSFRIIQKPGYVFVSSCNRLIGYDKLLLCIKRALKWSKCEG
16080887_Bacillus_subtil           AKDYKNLLKETIAITADKGVIVASTNSSAFGMKKFKGFIDAAFKETNERY
29375834_Enterococcus_faecal       AKNYGELVKDSIDILTDKGTLIASTNAANLSLAKYQKMVITALQEKNVRY
54294134_Legionella_pneumo         KNYYRFLHREAFKYMKPGSLLMTCNCSSALSSQDFCSLVSAQAVAMGKQA
42571865_Arabidopsis_thalia        SGMYRNLNSLAMRLTRSGGLMMTCSCSGAMTQSKFLGILQSAAAMSGRKI
CM2295_Cyanidioschyzon_merolae     RHRYRKMNAAALRLVRSSGFLFTCTCSASMTQSQFSAVLMEAALDAGRRI
42522494_Bdellovibrio_bacter       KAAYTKVFSNSIKLVNPEGLFAASSCSSHISTKEFMDICQEAFSRARKKA
14600479_Aeropyrum_pernix          FRAYKSSYLFALERAAGNSIVYLSSCSYFLSREEFITLVTEVASALEKEY
42524664_Bdellovibrio_bacter       KHAYLKMNTHAFRLVKKNGFVASCSCSGLLEEEEFRDAIRKASLRNYAEL
39998234_Geobacter_sulfur          RGGTSDLVAASLPLLAEGGLLITSSNHQKVDMADYLKELRRGALQAGSTV
34763641_Fusobacterium_nuclea      RDFFLDLCDKSFKLLENGGILGVITCAYHIGLQDLIEVTRMSASKNNKLL
6457710_Deinococcus_radiod         IRAYGKLAEGGLTRLAPGGILVSASCSAHVSAEEFEEAVMSAVRRSGRRW
32444786_Rhodopirellula_baltic     LRAYTRLNASAVDLLPPGGILVTCSCSGRVSRADFLNMLVDVGRKRGRDL
21110809_Xanthomonas_axonop        LKKYLDMNKLALGVVKPGGLFATFSCTGLVAEQEFLDMLRRASYFSGRTI
15837009_Xylella_fastid            LKKYLDMNKLALGVVKKGGLFATFSCTGLISEEQFLEMLRRAAFYSGRTI
53797508_Chloroflexus_aurant       MRAYVRLNMLGLRCVTPGGLLVSASCTAQIGPEQFRSLLGEAAAQAHRRL
22960169_Rhodobacter_sphaer        LRAYERLARLAAPLVAPGGYLVLCSCSHAADLSAFRNASARGIGRGGRRG
56679104_Silicibacter_pomero       LRAYERVARLAAALVRPGGYLGLCSCSHAADLSRFREASVRGIGRAGRSA
15605998_Aquifex_aeolic            LWAIWKLAYYSFQKLKKGGSLFICSCTYQISSEEMIRQVRLASTDVKRRI
34556476_Wolinella_succin          MNGWKYLIIGAIKVAAPKARIALYSCSYHMGLNELAQVALEASRDSYTRL
46446677_Parachlamydia_sp.         CRGYKDINRLAMQKMPRRSLLLTCSCSHHVNELLFQKVVFQAAAEANRNV
53712322_Bacteroides_fragil        LQGYRKLNAKAFEKIKPGGILFTFSCSQVVTKDNFRTAVFTAAAMSGRSV
48854326_Cytophaga_hutchi          CRGYKQINLKAFRKIKKGGLLFTYSCSQNIDKDLFQKIVFGAAADSYRNV
20094503_Methanopyrus_kandle       RRAYFDVNYKALGLVRDGGLFVTCSCSHFLEPSDFVRLVNEAAARRSVRL
14591657_Pyrococcus_horiko         LRAYFNVNFAGLNLVKDGGILVTCSCSQHVDLQMFKDMIIAAGAKAGKFL
15607037_Aquifex_aeolic            LRGYKELCVRGLKIAKSGGYLAIFSCSFHITEQHLLDVLLQASYDVRRQV
53796614_Chloroflexus_aurant       TRGYKDINLQALHLIRRGGILATFSCSGLVSADLFQKIIFGAAVDAGRDV
26246990_Escherichia_coli          CRGYKDINMLAIQLLNEGGILLTFSCSGLMTSDLFQKIIADAAIDAGRDV
29142267_Salmonella_enteri         CRGYKDINMLAIQLLNPGGILLTFSCSGLMTSDLFQKIIADAAIDAGRDV
47573089_Rubrivivax_gelati         SRAYKDINRLALKLLVPGGLLFTFSCSGGVDAALFHKIVAGAGLDAGVDG
48770381_Ralstonia_metall          ARAYKEINLVGMQLLRPGGLLFTYSCSGAISMELFQKIVAGAATDARSDA
48786339_Burkholderia_fungor       SRAYKDINLTGLKLLRPGGLLFTYSCSGAIDSELFQKIISGAAADARVDA
21674038_Chlorobium_tepidu         LKAYTKLNRLGLQLVRNEGYLATASCSHHVSEEDFLAAIHLGAMQAGKHL
15644445_Thermotoga_mariti         RRGYKEINLRAMRILKKPGVLVTSSCTQIVSEELFREILFDASFDTKTSL
39997720_Geobacter_sulfur          EKGYLTVNRRGMELLEEGGYLITCSCSYHMGREPLRELLAQAARQAGRHM
46199218_Thermus_thermo            YRAYKEVNLRAIKLLKEGGILATASCSHHMTEPLFYAMVAEAAQDAHRLL
48857855_Clostridium_thermo        ARGYKEINLKAMKLLPRGGYLATCSCSHFMTDDLFRKTLASAAKDASVSL
37523934_Gloeobacter_violac        LRGYKEINLRAMKLLTPGGILVTCSCSSHLSMEMFRDVIRDAAQDTGRTA
51894272_Symbiobacterium_therm     ARGYKEINLRALRMLTEGGFLVTCSCSYHMTPDLFKAVVAEAAQDARRRL
```

```
                                                      . .   .
23509703_Plasmodium_falcip          TIIGEGRISSDHPIHISLPETKYLTSILLM
16080887_Bacillus_subtil            TIIEEFTLPEDFKTISAFPEGNYLKVVLLQ
29375834_Enterococcus_faecal        KITDTYQLPADFQVNPNFPEGNYLKVLFIE
54294134_Legionella_pneumo          RILGVYGPASCHPTLAAFPEGNYLTAVLLA
42571865_Arabidopsis_thalia         TVVREAGAASDHPLDPSYPQGQYLSNLLLR
CM2295_Cyanidioschyzon_merolae      TILAERGAALDHVVSPAAPEGRYLTAMLLA
42522494_Bdellovibrio_bacter        TLVYIGGQPVDHPYPLAMEELRYLKFALFR
14600479_Aeropyrum_pernix           RILGDRGASPDHVYRGE-EYLSYLKGAFIH
42524664_Bdellovibrio_bacter        RSVLRGGHAADHPTLMQFPEGFYLKMYVHY
39998234_Geobacter_sulfur           RVISLAGQPEDFPYPVTFPEGRYLKYVVSV
34763641_Fusobacterium_nuclea       SVLGINYQPEDHPWILHIPETLYLKALWVK
6457710_Deinococcus_radiod          RKLLSSRHAPDH--HASFAEAEYLKAVFLQ
32444786_Rhodopirellula_baltic      IVLESRGPSPDHVFAVSCPESDYLKCIVVQ
21110809_Xanthomonas_axonop         QILKVAGAGPDHPFMAHVQESRYLKAVFCR
15837009_Xylella_fastid             QILKVAGAGPDHPFMAHVQESRYLKATFCR
53797508_Chloroflexus_aurant        QIIHEAGHPLDHPVPAGFPEGRYLKYIIAR
22960169_Rhodobacter_sphaer         QLIHTGFAGPDHPILPQLAESGYLKSLFFR
56679104_Silicibacter_pomero        QLIHTGFAGPDHPQLPQLAESGYLKAVFFR
15605998_Aquifex_aeolic             IIKSLNLQPIDHPYLPTFPESLYLKCLHAI
34556476_Wolinella_succin           QILQYHLQDIDHPAILNMPHSLYLRGIVFE
46446677_Parachlamydia_sp.          RLIGRHQLACDHPINLFHPESDYLKSFLLY
53712322_Bacteroides_fragil         RILHQLTQPADHPVNIYHPEGEYLKGLVLY
48854326_Cytophaga_hutchi           RIIHQMHQPADHPINIYHPEGEYLKGLVLY
20094503_Methanopyrus_kandle        RMLGPRGQPPCHPIVPGNPDTRYLKAMFCA
14591657_Pyrococcus_horiko          KMLEPRTQAPDHPILMASKDTEYLKCLFLY
15607037_Aquifex_aeolic             RVIAKTFQDLDHPWILQMPNTLYLKGVWVE
53796614_Chloroflexus_aurant        QILERLTQAPDHPVLLSFPEAEYLKGLICR
26246990_Escherichia_coli           QFIEQFRQAADHPVIATYPEGLYLKGFACR
29142267_Salmonella_enteri          QFIEQFRQAADHPVIATYPEGLYLKGFACR
47573089_Rubrivivax_gelati          YLLERLEAAPDHPTTVNFPEGEYLKGLAIL
48770381_Ralstonia_metall           RILRRLSAGTDHPMSAAFPEGEYLKGLLLE
48786339_Burkholderia_fungor        RILKRLGAGMDHPLLTAFPEGEYLKGLLLQ
21674038_Chlorobium_tepidu          RLISRAAQPPDHPVLLAMPETSYLKFACFY
15644445_Thermotoga_mariti          TVLRRGGQPPDHPVLMNVPETQYLKFYILQ
39997720_Geobacter_sulfur           RVVGVRSQAPDHPVLLAVPETEYLKCFILQ
46199218_Thermus_thermo             RVVEKRGQPFDHPVLLNHPETHYLKFAVFQ
48857855_Clostridium_thermo         RQIEARQQAPDHPILWNVPETDYLKFYIFQ
37523934_Gloeobacter_violac         CLVEQRGQAPDHPILLHIPETEYLKYLLIS
51894272_Symbiobacterium_therm      RLVEERAQAVDHPIVVGYDESHYLKCLIYE
                                               .          **
```

Note: This gene has identifiable homologs only in prokaryotes and plastid-containing eukaryotes (red algae, green plants, and apicomplexan *Plasmodium*) in our GenBank and TBestDB searches (using *Cyanidioschyzon* and *Arabidopsis* sequences as queries). Homologs of the gene are rarely found in cyanobacteria. All top hits are from gamma and beta-proteobacteria.

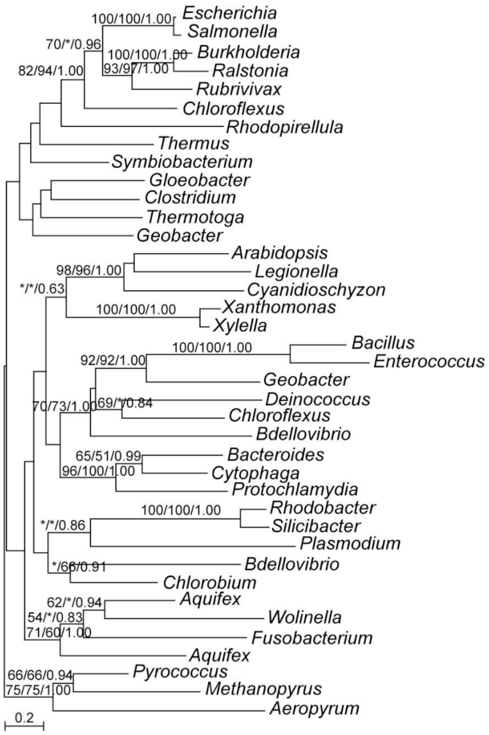

Figure 14. Molecular phylogeny of SAM-dependent methyltransferase. P-value = 0.921 from AU test for the presented gene tree. AU tests were also performed on alternative topologies including (**A**) monophyly of red algal, green plant and *Plasmodium* sequences, (**B**) monophyly of red algal, green plant, *Plasmodium* and cyanobacterial sequences, and (**C**) monophyly of red algal, green plant, *Plasmodium*, and archaeal sequences. Topology **A** investigates if red algal, green plant, and *Plasmodium* (which also has a relict plastid) sequences have the same origin. Topology **B** investigates if the three plastid-containing groups acquired the genes from plastids. Topology **C** investigates if these plastid-containing eukaryotic sequences have an archaeal or eukaryotic origin. P-value = 0.082 from AU test for topology **A** whereas p-values < 0.001 for topologies **B** and **C**.

---

15. Semialdehyde dehydrogenase (D)

```
CLUSTAL X (1.83.1) multiple sequence alignment


NUO00054019_Capsaspora_owczar       ------------------------GTRKDRK-------------------
60466242_Dictyostelium_discoi       RVGLIGARGFTGGNLVRLIDGHPNSSTNFGKPITTEFPQLKKPENIIDGW
32408531_Neurospora_crassa          RVALIGARGYTGQELIRLIDSHPNSRELAGKKLEGYNKQEVSPEDVIDCW
5155_Saccharomyces_cerevi           RVALIGARGYTGKNLVSLINGHPYSRELKGQKLQDYTKSEIQIQDIVDFW
24214878_Leptospira_interr          EISILGAGGLTGKELLLLFSRQKESDKLAGKTISEVFPEVSVFKKHKSLV
21673935_Chlorobium_tepidu          TVSVIGASGYSGAELVKLLMKHPGAHTQAGKRFTELYPSIPTFQTYSDVY
27360910_Vibrio_vulnif              KTTIIGASGYTGAELALMVEKHPENSTDAGKCISQLHGKLAVLPLTSDVI
53711823_Bacteroides_fragil         KAGIIGGAGYTAGELIRLLINHPESTSNAGNKITDVHEGLYTFTDEIDVL
34558370_Wolinella_succin           PVGIVGVSGYTGLELLKMLLEHPYANTEGGVRVGALHSSLQSVEKACELL
47529651_Bacillus_anthra            KVAIIGATGYGGIELIRLLEQHPYSFSQVGECITNVYPHFQTLQEIAEIV
38233757_Corynebacterium_dipht      KVAIVGASGYAGGEILRLLLQHPLGSSTVGQTVSELMPHLPVVEPTHDVV
29610427_Streptomyces_avermi        RVAVAGASGYAGGEALRLLLAHPEGNSNAGQKLGALQPHLLVLQETHDVV
```

```
41407459_Mycobacterium_avium        RVAVAGASGYAGGEILRLLLGHPAAAASAGSPLGEHHPHLTVLEPTHDVV
15606911_Aquifex_aeolic             RVSVFGATGYTGIELLRSLLTHPHSQSYKGKKVREVLPFFSEFLEE-ELA
32397402_Rhodopirellula_baltic      RVALVGSTGYTALEVARLLLTHPGSRQDEGKPLSEIHPMLATLQPLADVA
11499653_Archaeoglobus_fulgid       KVGIIGASGYTGSELLRILANHPESRYEGQKIWKVHRFLKEFCSPCDLV
16331759_Synechocystis_sp.          RVGIIGASGYGGIQLVRLLLEHPQGHSSAGKPYSDLYPHLTTIEPICDAV
46135514_Anabaena_variab            PVGIVGASGYGGVQLVRLLMDHPEGESSVGKSFASLYPHLASIEEVCEVV
57168350_Campylobacter_coli         KVGILGASGYAGNELVRILLNHPKSSSSVGQSYTKLYPNTPNFEDKLDVL
15895656_Clostridium_acetob         QAGIIGATGYAGEVLTWLLNNHPNSHSHGNSSFNSTYGNFTCIDDNIDIL
23473460_Desulfovibrio_desulf       RAGLVGATGYTGMELIRLLLGHHSSRAEAGKRLDDIYPFTQAITEPCDIA
53796023_Chloroflexus_aurant        RVGIYGATGYTGIELIKLLSRHPEARSAAGQRLSEVFPILSRLTAVVDLV
4468762_Thermotoga_neapol           RVGIVGATGYTGIELFRLLKKHPSSRTYAGKKMMEVYPSTLVLEDFCDVV
22956652_Rhodobacter_sphaer         NIAILGASGYTGAELVRLIATHPAGDRKAGMAMSEVFPFLRRLQKIVDLA
CM2432_Cyanidioschyzon_merolae      AVAILGAAGYTGSELVRLLLRHPHTERTRGRPYGQVYPQYAPLCLTVDTV
48765484_Rhodospirillum_rubrum      NVAILGASGYTGAELVRLLARHPRANRKAGQAFASVFPHLGVLSTIIDFV
50428699_Oryza_sativa               RIAVLGASGYTGAEIVRLLANHPQADRKAGEQFGSVFPHLINLVAVVDAV
19699208_Arabidopsis_thalia         RIGLLGASGYTGAEIVRLLANHPHADRKAGQSMESVFPHLRTLVSVVDAV
39997965_Geobacter_sulfur           KVAVVGASGYTGVELLRILHCHPESEQSAGKPVSDLFPSLRVLENLADII
7379404_Neisseria_mening            KVGIVGATGYTGVELLRLLAAHPDSRSEAGTAVADYFPSLRAFQTPCDIV
30249453_Nitrosomonas_europa        NAGIVGGTGYTGVELLRILVQHPKSRQEAGTGVDELFSSLRKFSDPCDVV
53613186_Azotobacter_vinela         KVGIVGGTGYTGVELLRLLAQHPQSRSEAGVKVADMYPNLRAFSVPCDVV
15595859_Pseudomonas_aerugi         KVGIVGGTGYTGVELLRLLAQHPQSRSEAGVKVADMYPNLRQFSVPCDVV
24211481_Deinococcus_rediod         TVAIVGGSGYAGGEFLRLALGHPHSERSAKLPVSMVHPNLRKFRKAADII
55232034_Haloarcula_marism          TASVVGGSGFTGGELLRLLDGHPESRSKENKTIGHSHPNLRRFSSPVDVL
48853180_Ferroplasma_acidar         KVGIVGASGYVGGELMRLLVRHPESTRHSGEKVSRIHPDLYKFEDIVDLI
18313659_Pyrobaculum_aeroph         KVCIIGASGFVGGELLRILLQHSGSRRFKGEYVYRVHPNLRKFVEPADVV


NUO00054019_Capsaspora_owczar       -----------------IVTVDLSGDNRFYGLPERFRERIASASFISNPG
60466242_Dictyostelium_discoi       FMALPDKISSPYIQTLEPVLVDLSSDHRFYGQPETNRAAIKESKLIANPG
32408531_Neurospora_crassa          VMALPNGVCKPFVEAVWSVIIDLSADYRFYGLPELQRSNIIQATQIANPG
5155_Saccharomyces_cerevi           VMALPNKVCEPFVETIQSKIIDLSADHRFYGLPELDRAKIANAAKIANPG
24214878_Leptospira_interr          VLAVPNEVSVESAPKFLHKVIDLSGVYRLFGIPEIFRDQLKNADFVSNPG
21673935_Chlorobium_tepidu          LLALPHGEALQLVPGIVKKVIDLSGDFRLYGMPELFRDEIAGSTAISNPG
27360910_Vibrio_vulnif              FLATAHEVSHDLAPLFLCQVFDLSGAFRVYGLAEWNEEAIKASQLVAVAG
53711823_Bacteroides_fragil         FFCTAHGDTKKFMESHNLKIIDLSMDYRIYGLPELNRRATCTAKHVANPG
34558370_Wolinella_succin           FLALPHQSAMEFAKEVLKKVVDLSADYRLYGLIEWAREEIVQARLIANPG
47529651_Bacillus_anthra            FLATPAGVSAELTPKLLLKVIDLSGDFRMYGLSEWKRSEIQKANLIANPG
38233757_Corynebacterium_dipht      FLALPHGFSAEIAQQLPITVIDCAADFRLYGIPEMHRDLIASSKRIAVPG
29610427_Streptomyces_avermi        FLALPHGQSAAVAEQLGVLVVDMGADFRLYGLPELGRAALEGSKRIAVPG
41407459_Mycobacterium_avium        FLALPHGHSAALADQLGTLIVDCGADFRLYGLPELARERLRGARRIAVPG
15606911_Aquifex_aeolic             FLCLPHEVSYEIVPKLLKKVVDLSGAYRIYGLPEVFREEIKNSDLVANPG
32397402_Rhodopirellula_baltic      MCCLPHGASAESVKQLAMRVIDFSADFRLYGMPEFFADEIRSADIVANPG
11499653_Archaeoglobus_fulgid       FTAVPHGEAMKYVPQLLIKVVDISADYRLYGLTELHREEIKKANLVANPG
16331759_Synechocystis_sp.          FLGLPNGLACDMAPALLCKVLDLSADYRFYGLPELYRDAIRDAQLIGCPG
46135514_Anabaena_variab            FLSMPNGLACQIVPTLLCKVLDLSADYRFYGLPELYRDRISEAQLVGCPG
57168350_Campylobacter_coli         FTATPHEFSAKLFSESLMKIIDLSADFRLYGLCELYQEEIKNANLIANPG
15895656_Clostridium_acetob         FTALPSGKTFSIVKKAVVKVIDLGADYRLYGLSEIYREKIKTSNLIANPG
23473460_Desulfovibrio_desulf       FLAVPHGAAMEMAAQLHLKVVDLSADFRLYGLPELYAEQTRNATLVANPG
53796023_Chloroflexus_aurant        FTCLPHNTPIPIVQRALARVVDFSDTFRIYGLPELHRERIRQARLVANTG
4468762_Thermotoga_neapol           FTALPAGVSYRLAMELQVKIIDLGADFRFYGLPELYRDSIKNARIVGNPG
22956652_Rhodobacter_sphaer         FCALPHATSQAVIAGLPLKVVDLSADFRLYGLTEFYRDEIRGARLVAGTG
CM2432_Cyanidioschyzon_merolae      FCCLPHATTQSIVTSILPRVIDLSADFRLYGLTEFARADIRSARLVANPG
48765484_Rhodospirillum_rubrum      FCALPHGTTQTIIGDLLLRIADLSADFRLYGLTEINRAAIATARLVAVPG
50428699_Oryza_sativa               FCCLPHGTTQEIIKGLPLKIVDLSADFRLYGLTEVLRNEIRNARLVANPG
19699208_Arabidopsis_thalia         FCCLPHGTTQEIIKELPLKIVDLSADFRLYGLTEILREDIKKARLVANPG
39997965_Geobacter_sulfur           FTALPHKAAMEVVPTFLKRVIDLSADYRFYGLPEVRRAAVKGAKLVANPG
7379404_Neisseria_mening            FFATPNGIAMKDAPRLIVRVIDLSADFRIYGLSELNREAVAQARLVANPG
30249453_Nitrosomonas_europa        FFATPNGIAMQQAKALLIKVIDLAADFRIYGLPEVNREKIRDARLIANPG
53613186_Azotobacter_vinela         FFATPHGVAHALAGELLTRVIDLSADFRLYGLPEVNRERIRSARLIAVPG
15595859_Pseudomonas_aerugi         FFATPHGVAHALAGELLTRVIDLSADFRLYGLPEVNREKIRQARLIAVPG
24211481_Deinococcus_rediod         VLALPHNSAAKRITEFEKVIVDLSADFRLYGNPELHREDLRGATRIACAG
55232034_Haloarcula_marism          FAATPHGVSMEQIDAFQGTVVDLSADFRLYALPELNRDNLEGADLIASGG
48853180_Ferroplasma_acidar         FLALPHGTSIKYVPGLVVKIIDMSADFRLYGMPELHREQIKNATYIATPG
18313659_Pyrobaculum_aeroph         FLALPHGESVKWVPKLYVAVFDLSADFRLYGQPELHRSELVGAKLVAVPG
                                                         * .   *.:.  *        :  :.  *

NUO00054019_Capsaspora_owczar       CYATGAQLSILPLAPAVFGISGYSGAGTTPSEKNDVNYLKDNIVPYSLVN
60466242_Dictyostelium_discoi       CYATGMFLTLKPFVPSCFGISGYSGAGSKPSEKNDPTRLSDNILPYKLVQ
32408531_Neurospora_crassa          CYATAAQLGISPLVPHVFGVSGYSGAGTKPSPKNDVENLTNNIIPYSLTG
5155_Saccharomyces_cerevi           CYATGSQLTISPLTPTVFGVSGYSGAGTKPSPKNDPKFLNNNLIPYALSD
24214878_Leptospira_interr          CFSTSVILPIFLLGIIVDSKSGVSGAGRTEDSGYSYTSVYENFRAYKILS
21673935_Chlorobium_tepidu          CYATSIILGLAPLFVNVTAVSGISGAGRSAKLELSFSEMSGNMRAYKVGK
27360910_Vibrio_vulnif              CYTTAAQLAIKPLLPVINATSGVSGAGRKASMTNSFCEV--SLQPYGIFN
```

```
53711823_Bacteroides_fragil        CFATCIQLGLLPLAVMVNAITGSTGAGVKPGATSHFSWRNNNMSVYKAFE
34558370_Wolinella_succin          CYPTATLLGILPFILFVDAKSGVSGAGKKLTHNTHYPTINENLFAYSPLS
47529651_Bacillus_anthra           CFATAALLAILPLVIIIDAKSGVSGAGKTPTTMTHFPELYDNLRIYKVNE
38233757_Corynebacterium_dipht     CFPTGATLALLPAIVSVVSITGVSGAGKKASVAMLGAETMGSLKAYNVAG
29610427_Streptomyces_avermi       CYPTAVSLALFPAYAVIVAASGTSGAGKAAKPHLLGSEVMGSMSPYGVGG
41407459_Mycobacterium_avium       CYPTAALLALLPAMVTVVAVSGTSGAGRAAKTDLLGSEVIGSARAYNIAG
15606911_Aquifex_aeolic            CYPTATLLAIYPFLVIVHALSGVSGAGRKPKQQFHFPEMTENFFNYAVEK
32397402_Rhodopirellula_baltic     CYPTSAIMPLAPLVIIVDSKSGVSGAGRSPKLGTLYCETNESISAYAVGT
11499653_Archaeoglobus_fulgid      CYPTGTILAASPLAVVFDCKSGITGAGESPSAFTHYPNLHESIVPYKITE
16331759_Synechocystis_sp.         CYPTASLLALSPLLAIIDAKSGTSGGGREAKVNMLLAEAEGSLGAYGVAK
46135514_Anabaena_variab           SYPTASLLALSPLLAIVDAKSGTSGGGREAKTYLLLAEADNSLAPYSVVR
57168350_Campylobacter_coli        CYTTCSILSLYPLFVIIDAKSGVSGAGRSAKVENLFCEVNENIKAYGLAS
15895656_Clostridium_acetob        CYPTASSLALIPLLIIIDAKSGVSGAGRKASTNNLFIECNDSIKAYGVAS
23473460_Desulfovibrio_desulf      CYPTSVILGLYPALIVIDAKSGATGAGRKASVPSLFCEVHDSFRAYGIGT
53796023_Chloroflexus_aurant       CYPLTAILPLVPLVVIVDSKSGVSGAGRSLSLKTHFGETHETFSAYNVGR
4468762_Thermotoga_neapol          CYPTSVILALTPALIVVDSKSGVSGAGRKETLDYTFSEVNENLRPYNVAK
22956652_Rhodobacter_sphaer        CNAATGQYAIRPLIILIDLKAGVSGAGRSLKENLLHAELSEGTHAYSAGG
CM2432_Cyanidioschyzon_merolae     CYPTGAQLALVPLIVIIDAKSGVTGAGRSAKEANLFCEVTEGMHAYAVAN
48765484_Rhodospirillum_rubrum     CYPTSAQLPLIPLLIIIDAKSGASGAGRDAKEGSLHCEVSEGIHAYGVGT
50428699_Oryza_sativa              CYPTSIQLPLVPLIIIIDAKSGVSGAGRGAKEANLYTEIAEGIHAYGIKG
19699208_Arabidopsis_thalia        CYPTTIQLPLVPLLIIIDAKSGVSGAGRGAKEANLYSEIAEGISSYGVTR
39997965_Geobacter_sulfur          CYPTSVILGLQPLLIISDSASGVSGAGRGAKVDNLYCEVNEGFKAYGVGG
7379404_Neisseria_mening           CYPTCVSLPLVPLLLIADCKSGVSGAGRKGNVGSLLCEVGDNFKAYGIAG
30249453_Nitrosomonas_europa       CYPTAVQLGFIPLILIADTKSGVSGAGRKAEIHTLYAEASDNFKSYAVPG
53613186_Azotobacter_vinela        CYPTATQLGFLPLLLIADCKSGVSGAGRGAKVGSLFCEASESMMAYAVKG
15595859_Pseudomonas_aerugi        CYPTATQLGLIPLLLIADCKSGVSGAGRGAKVGSLFCEAGESMMAYAVKG
24211481_Deinococcus_rediod        CFATSVILALYPLLIIATGLVGSSAAGASASESSHHPERAGSLRVYKPVG
55232034_Haloarcula_marism         CNATATILGLLPLFIVVDVKVGSSEGGAGGGEASSHPERSGVVRPYAPTG
48853180_Ferroplasma_acidar        CIASSSIYSIAPFLINVDAKVGSSGSGSGLDESKVFSERFDSIRAYKPVN
18313659_Pyrobaculum_aeroph        CMATASILMLAPLAPVIDAKIGSSGAGAEGSVVDLHSFRTYVVRPYEPVH
                                   . .                  * : .*                  *

NUO00054019_Capsaspora_owczar      HLHEREVSRQVGSKVNFMPHVAPFFQGITLTTDIRLVVQRFQDMFGKEPL
60466242_Dictyostelium_discoi      HTHELEVSHQLGSPIYFMPHVGQFFQGITLTISMELVVERYQKFYQNEPL
32408531_Neurospora_crassa         HIHEREVSSQLGAEIAFMPHVAVWFRGIHHTISIPLIRQLYQDRYAGEKL
5155_Saccharomyces_cerevi          HIHEREISARIGHNVAFMPHVGQWFQGISLTVSIPIIRKLYRNFYEDEKL
24214878_Leptospira_interr         HQHEPEIREYVDPEVIFTPHLLPVYRGILSTIVLEFLISILENSSLNEPF
21673935_Chlorobium_tepidu         HQHTPEIMQTLSFRFVFTPMIAPYVRGIYSVLNVRLVRELYAGFYANAPF
27360910_Vibrio_vulnif             HRHQPEIVHHLGCDVIFTPHLGNFKRGILATITMKLVAHAFAQAYDRKPA
53711823_Bacteroides_fragil        HQHVPEIKQSLDAEIDFIPYRGDFPRGIFATLVVKTIVRMYEEYYAKDSF
34558370_Wolinella_succin          HRHEPEIAEKIAKKVNFIPHLTPLTRGMLVSTFARLAMAILRERYANEPF
47529651_Bacillus_anthra           HQHIPEIEQMLTKPITFSTHLIPISRGIMVTLYAKVLQQLYEEAYEQSAF
38233757_Corynebacterium_dipht     HRHTPEISQNLKIVVSFTPVLAPLPRGILTTATAKLIREIYLNAYENEPF
29610427_Streptomyces_avermi       HRHTPEMIQNLPVSVSFTPTLAPMPRGILATCTAKAVRAAYEKAFADEPF
41407459_Mycobacterium_avium       HRHTPEIAQGLDVTVSFTPVLIPTSRGILATCTARTLRAAYEKAYDAEPF
15606911_Aquifex_aeolic            HRHTPEMEDVIEVKVRFTPTVVPTSRGMISTVYLKSVKELFKEVYEDEIF
32397402_Rhodopirellula_baltic     HRHAPEIADLVPIEVMFTPHLTPMDRGILSTIYVKPMMSLLRDTYSDQPC
11499653_Archaeoglobus_fulgid      HRHYYEMVQELDIKISFTPQVYPGSRGILTNAHVFLLYRIYEEFYEDSFF
16331759_Synechocystis_sp.         HRHTPEIEQIAELRVQFTPHLIPMVRGILATVYATLLITIYSAFYRASPF
46135514_Anabaena_variab           HRHTPEIEQICEVTVQFTPHLIPIVRGTLATVYATLLTTIYTAFYRNSPW
57168350_Campylobacter_coli        HRHTPEIEEHLKITLQFTPHLVPMQRGILTTAYANLIREIYQKYYQDRKF
15895656_Clostridium_acetob        HRHTPEIEQTLKITLTFTPHLVPMNRGILSVCYSNLVYDIYKKFYKNEPF
23473460_Desulfovibrio_desulf      HRHTPEIEQELPLTVSFNPHLLPVNRGILSTIYTTLVQAIYENAYADKPW
53796023_Chloroflexus_aurant       HRHLAEMEQETGLHIIFAPHLLPVFRGILSTIYVNLLAAVYADAYAAEPF
4468762_Thermotoga_neapol          HRHVPEMEQELDVKVIFTPHLVPMTRGILSTIYVKTIHRIYQEFYAGEPF
22956652_Rhodobacter_sphaer        HRHLGEFDQEFPVQVRFTPHLTPMNRGILANVYVKGVHRALTERYLTETF
CM2432_Cyanidioschyzon_merolae     HRHAPEIEQGLGVVVNFTPHLVPMARGILETIYVRCLRACLEQAYGEEKF
48765484_Rhodospirillum_rubrum     HRHGPEIEQGLPVAVTFTPHLMPMNRGILSTIYLRALRQALSAAYADEAF
50428699_Oryza_sativa              HRHVPEIEQGLKVTISFTPNLICMKRGMQSTMFVEMLYQHLKSTYEGEEF
19699208_Arabidopsis_thalia        HRHVPEIEQGLKVTVSFTPHLMPMIRGMQSTIYVEMLHQQLKTSYEDEEF
39997965_Geobacter_sulfur          HRHTPEMEQELRITITFTPHLAPMDRGILSTIYGRLLAGLYAEFYGGEPF
7379404_Neisseria_mening           HRHLPEIRQTIAEGFVFTPHLAPMIRGMHATVYLHL---ILRDYYRDSLF
30249453_Nitrosomonas_europa       HRHLPEIRQGLPIGLTFVPHLTPMIRGIHATLYARLLQTLYENRYANEPF
53613186_Azotobacter_vinela        HRHLPEISQGLEVGLTFVPHLTPMIRGIHATLYASVLQALYEKRYASEPF
15595859_Pseudomonas_aerugi        HRHLPEISQGLDVGLTFVPHLTPMIRGIHATLYAHVLQALFEKRYADEPF
24211481_Deinococcus_rediod        HRHTAEAQQELKFPLHLTAISTPRVRGILTTIQAWVVWSAYREVYGQEPF
55232034_Haloarcula_marism         HRHEAEIQQFLGIDVSFTVHAVDMIRGASATCHVFPLWGAYRGEYEDEPF
48853180_Ferroplasma_acidar        HRHTPEIEQELKVKIALSAHSVNMVRGILTTSSIYSLWSLLRKFYGNEKF
18313659_Pyrobaculum_aeroph        HRHIAEIEQELRVKVAFTPHAVDMVRGIFATGHVFVMWKMYRSMYGDSKF
                                   * *  *        . :        :*

NUO00054019_Capsaspora_owczar      IEIVVPVVKDNAQKHFVRLFAVDPATNRVVVVATLDNLLKGAATQALQNI
60466242_Dictyostelium_discoi      IKIDIPEVKSNSGKHTVTIFAVN--GNHLVVVTTLDNLLKGAATQALQNM
```

```
32408531_Neurospora_crassa          VKVVAPSVKNIGGKHGVEIFEVDKSGRRVVICATIDNLLKGAATQCLQNM
5155_Saccharomyces_cerevi           VHVIIPLVKDIEGTHGVVIFKLNDAEDRVVVCATIDNLLKGAATQCLQNI
24214878_Leptospira_interr          IRIL-VELKKVQHTNFLDI-SLRKRENTLVVVSALDNLVKGAAGQALQNI
21673935_Chlorobium_tepidu          VRLRVTEVSHVAYTNFCDILAFES-DGSLVIITAIDNLVKGAAGQAVQNM
27360910_Vibrio_vulnif              VRLKIPRIQDVEFTPFCDLWRVQ--GQHIIVISAIDNLLKGASSQAMQCL
53711823_Bacteroides_fragil         VHIVNIDLKQVVNTNKCLI-HLEKHGDKLLIISCIDNLLKGASGQAVHNM
34558370_Wolinella_succin           IRLRPVEIKAVAGTHFCDI-YAMSQGRDLWVNSAIDNLLRGASSQAIANA
47529651_Bacillus_anthra            IRIRFPSPKEVRGSNYCDMIAYDERTGRVTIVSVIDNMMKGAAGQAIQNA
38233757_Corynebacterium_dipht      VHILQPQTQAVVGSNMCHIVDADDNSKKLVVTSAIDNLTKGTAGAAVQCM
29610427_Streptomyces_avermi        VHLLWPATASVYGSNAVQVVAYDAAAQRIIAISAIDNLTKGTAGGALQSM
41407459_Mycobacterium_avium        IYLMLPRTGAVIGSNAAHIVAVDEAAGVLVAIAAIDNLVKGTAGAAVQSM
15606911_Aquifex_aeolic             VKVVPPQTKWVLGTNYCFIPHYDERTGYYVIISAIDNLGKGASLQAVQNA
32397402_Rhodopirellula_baltic      VHVVLPATKYVAGTNHVQI-SVRPSGKRAVIVCAIDNLTKGASGAAVQNM
11499653_Archaeoglobus_fulgid       VRIQ-VSLSQVRGSNFCDI-SIHPGEDRVVVVSAIDNLVKGASGQAVQNM
16331759_Synechocystis_sp.          VKILYPQTKWAWGTNLCYIIEVDPRTGRVIVLSAIDNLVKGQAGQAVQCL
46135514_Anabaena_variab            IKVCYPQTKWAAGSNLCYIVEVDPRTGRVIVMSAIDNLIKGQAGQAIQCL
57168350_Campylobacter_coli         IRLLFPQTRWVKSSNFADIFSIDERTNRIIVLGAIDNLIKGAAGQAVQNM
15895656_Clostridium_acetob         IRIILPETRWVKGSNYCDIIRIDKRTNKIIVLSAIDNLMKGAASQAVQNM
23473460_Desulfovibrio_desulf       VRVLLPALRNVRGTMYCDMLVVDPRTHRLIIVSCIDNLCRGASGQALANA
53796023_Chloroflexus_aurant        VHVLLPELRHVQHTNHVVIYQMVE-PGRAILVAVIDNLVKGAAGQAVQNM
4468762_Thermotoga_neapol           VHVLFPSTKWCYGSNHVFIMQLEERTGTPILMSAIDNLVKGASGQAIQNM
22956652_Rhodobacter_sphaer         LEVLLPSTRDIRGSNYVHIVIGDRVPGCAMVVAVLDNLCKGSSGQAIQNA
CM2432_Cyanidioschyzon_merolae      VHVLVPQTRYVRGSNHVVMVFPDRLQGRAILVVAFDNIVKGASGQAVQNM
48765484_Rhodospirillum_rubrum      VRVVSPHTRHVRGSNFVLIVHADRVPGRVIVTCVEDNLVKGASGQAIQDM
50428699_Oryza_sativa               VKLLVPHTRHVVGSNYCFMVFEDRIPGRAIIISVIDNLVKGASGQAVQNL
19699208_Arabidopsis_thalia         VKVLVPRTHNVRGSNYCHMVFPDRIPGRAIIISVIDNLVKGASGQALQNL
39997965_Geobacter_sulfur           VRVLVPSTAHVRGSNFCDIVVVDQRTGRVIVVSAIDNLVKGASGQAVQNM
7379404_Neisseria_mening            VDILTPETRSVRGANLCRIIQQAAQSDVWVVLSVIDNLVKGAAGQAVQNM
30249453_Nitrosomonas_europa        VDVLHPETRSVRGSNFCRIVHRPGNGDTAVILSVTDNLVKGAAGQAVQNM
53613186_Azotobacter_vinela         VDVMHPETRSVRGANVCRIVHRPQGGDLVVVLSVIDNLVKGASGQAVQNM
15595859_Pseudomonas_aerugi         VDVMHPETRSVRGANVCRIVHRPQGGDLVVVLSVIDNLVKGASGQALQNM
24211481_Deinococcus_rediod         IRIVYPDPMLLDGTNFCDIFEMDVDTGRVVLMSAIDNLVKGTAGHAIQSL
55232034_Haloarcula_marism          VELVYPEPKSVAGTNRAEVFELDPGNKRLVVFSAIDNMMKGSAGQAVHAA
48853180_Ferroplasma_acidar         IRLIYPDPKTVIGSNFADIFAIDKYVKRVVSFGAIDNLIKGAAGNAVQSM
18313659_Pyrobaculum_aeroph         IRIVYPNVKYVLGSNFVDIFELDQRLNRLVTFSAIDNLVRGAAGQAVQAF
                                    : :               :                **: :* :  .:

NUO00054019_Capsaspora_owczar       NIALGLRELDGI
60466242_Dictyostelium_discoi       NICLGLDELASI
32408531_Neurospora_crassa          NLALGYAEYEGI
5155_Saccharomyces_cerevi           NLAMGYGEYAGI
24214878_Leptospira_interr          NLMTGAKETLGL
21673935_Chlorobium_tepidu          NLMLGFGETTAL
27360910_Vibrio_vulnif              NIHYGFSPLTAL
53711823_Bacteroides_fragil         NLMFNLEETVGL
34558370_Wolinella_succin           NLMLGLPEGAGL
47529651_Bacillus_anthra            NIVAGLEETTGL
38233757_Corynebacterium_dipht      NLSLGVSETSGL
29610427_Streptomyces_avermi        NIALGLPEELGL
41407459_Mycobacterium_avium        NLALGWPETQGL
15606911_Aquifex_aeolic             NLMFGLAEDDGL
32397402_Rhodopirellula_baltic      NVMFGLPETAGL
11499653_Archaeoglobus_fulgid       NLMMGFDETLGL
16331759_Synechocystis_sp.          NLMMGWEESLGL
46135514_Anabaena_variab            NIMMGWDETLGL
57168350_Campylobacter_coli         NLMFDFDEDEGL
15895656_Clostridium_acetob         NIMYGLAEKTGI
23473460_Desulfovibrio_desulf       NLMLGLPVNTGL
53796023_Chloroflexus_aurant        NLLLGFAETVGL
4468762_Thermotoga_neapol           NIMFDLKETEGL
22956652_Rhodobacter_sphaer         NLMLGLDEAAGL
CM2432_Cyanidioschyzon_merolae      NVMMGIPEHTAL
48765484_Rhodospirillum_rubrum      NVMLGFPETLGL
50428699_Oryza_sativa               NLMMGLPENTGL
19699208_Arabidopsis_thalia         NIMLGYPETTGL
39997965_Geobacter_sulfur           NIMCGLPEGLGL
7379404_Neisseria_mening            NIMFGLKETHGL
30249453_Nitrosomonas_europa        NIMYGLPETTGI
53613186_Azotobacter_vinela         NILFGLDERLGL
15595859_Pseudomonas_aerugi         NILFGLDERLGL
24211481_Deinococcus_rediod         NVAQGWDERAGL
55232034_Haloarcula_marism          NVALGIEETAGL
48853180_Ferroplasma_acidar         NIMNGFNEYEGL
```

```
18313659_Pyrobaculum_aeroph         NVAMGFPEDEGL
                                    *:  .     .:
```

Note: This gene is found in green plants, red algae, fungi, *Capsaspora* and prokaryotes. All top hits in GenBank searches are from alpha-proteobacteria. Phylogenetic analyses also support an alpha-proteobacterial origin of the gene in red algae and green plants. Fungal and *Capsaspora* sequences are apparently different (with an about 500 aa N-terminal extension and many conserved residues shared between them) from red algal, green plant and prokaryotic sequences. The *Neurospora* sequence (GenBank accession number P54898) is experimentally determined to be a mitochondrial precursor. Red algal and green plant sequences share many conserved residues with alpha-proteobacterial homologs. The *Arabidopsis* sequences (GenBank accession numbers and TAIR loci NP_565461, AT2G19940 and NP_849993, AT2G19940 respectively) are annotated to be located in cytoplasm. Sequence of *Capsaspora* sequence was obtained from TBestDB.

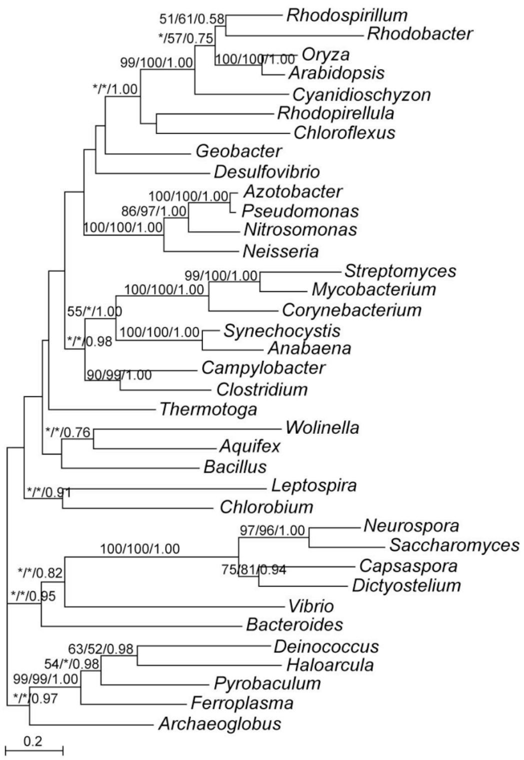

Figure 15. Molecular phylogeny of semialdehyde dehydrogenase. P-value = 0.981 from AU test for the presented tree. AU tests were also performed on alternative topologies including (**A**) monophyly of all eukaryotic sequences, and (**B**) monophyly of cyanobacterial, red algal, green plant, and alpha-proteobacterial sequences. Topology **A** investigates if all eukaryotic sequences have the same origin (mitochondrial or eukaryotic) whereas topology **B** investigates if red algae and green plants acquired this gene from plastids and further spread to alpha-proteobacteria. P-value = 0.045 from AU test for topology **A** and p-value < 0.001 for topology **B.**

---

16. Dihydrodipicolinate reductase (dapB) (D)

```
CLUSTAL X (1.83.1) multiple sequence alignment


116055387_Ostreococcus_tauri]       TPVMVCGVGGKMGRATASAVVRRDGFELIPTALDGEAVLKKAMIVVDYTL
18406430_Arabidopsis_thalia         ISIMVNGCSGKMGKAVIKAA-DSAGVNIVPISFEREKVLSSVFIVVDYTI
42572737_Arabidopsis_thalia         ISIMVNGCSGKMGKAVIKAA-DSAGVNIVPTSFEREKVLSSVFIVVDYTI
CM2591_Cyanidioschyzon_merolae      LALVSNGLPGRMALEVAQAALRRG-LELVPFSLERAQLATKLKICVDFTQ
ISO00167059_Isochrysis_galbana      TRVSLYD--PNARDALAQVVRETYPV-------DGEAIL----ICVDYTH
121542607_Candidatus_Desulf         IPIMINGLPGNVAKMIVSHVLRDSRFALVPFSLVRAARIGDIKISVDYTH
123966140_Prochlorococcus_mari      IPVLVSGALGKMGREVVNSVLNASDCELVAIDIDLEGTLCSISVLVDFTH
73748738_Dehalococcoides_sp.        IKVVVHGASGKMGQEVLKTLCQENNFHPVGVDIDLSSILSQTKVMVDFTV
123216321_Mycobacterium_tuberc      MRVGVLGAKGKVGATMVRAVAAADDLTLSALDADPLSLLTDGNVVIDFTH
145593931_Salinispora_tropic        LRVGVLGARGRMGIEVCKAVDAAPDMELVAVDQDWLFNASDAGVVVDFTT
121535277_Thermosinus_carbox        IRVMVCGAYGKMGREVLKAVHRDEQLSIVGVDIDLQTVINETKVMVDFTN
29376120_Enterococcus_faecal        IKIIVAGFKGRMGSTATQMVLETADFELVGYDPRLEEVLA-VKVWIDFTV
116873342_Listeria_welshi           MKVAVSGFKGRMGHEVVKTVLREEDLELVALDHNLSEMLEEAKCVVDFTT
125973685_Clostridium_thermo        IRVCLVGL-GKTGKEIAKVILEQENMKLVSVCSNLEQVIFKTKVVVDFST
20094858_Methanopyrus_kandle        IGVVVLGATGRMGRRICRMVIEDEELELVGIASALPNIAKDADVAIDFTV
118070755_Shewanella_woodyi         VRVAITGGSGRMGRTLIEAARQQPMIYLGAIERSLDLAVDDFDVLIDFTS
89899565_Rhodoferax_ferrir          HRVAIAGASGRMGQMLIEAIKSAPDCELTGLDVDLAVGLKQARTLIDFTR
120613186_Acidovorax_avenae         IRVAVAGASGRMGRMLIEALRSGSELVLAGLDVDLHEGLAGCDVLIDFTR
20093259_Methanosarcina_acetiv      INAAVLGACGRMGSLIIENITCSTDMQLVAFDIDLETVLKKTQVLIDFTA
116754136_Methanosaeta_thermo       TDVALTGAKGRMGSLIIDEIRSSPDLKLVAIDIDVQRVLRESRVLIDFTV
                                          .   .                                  :*::

116055387_Ostreococcus_tauri]       PESVNANGELYVKCGVPFVMGTTG---GDRDALLREVKASGNVIAPQMGK
18406430_Arabidopsis_thalia         PSAVNDNAELYSKVGVPFVMGTTG---GDRNKLYETVEEAKIVISPQMGK
42572737_Arabidopsis_thalia         PSAVNDNAELYGKVGVPFVMGTTG---GDRTRLYKTVEESKIVISPQMGK
CM2591_Cyanidioschyzon_merolae      PQAVIPNAAWYVENGFDFVMGTTG---GDTSALYQMVARSARVIAPNMAK
ISO00167059_Isochrysis_galbana      PSAVNGNAEWYAANKFPFVMGTTG---GDRESLLDAVTGVTSVIAPNMAK
121542607_Candidatus_Desulf         PTAVNGNCEFYCAHGLPFVMGTTG---GDRDRLDPAVRASSIVIAPNMAK
123966140_Prochlorococcus_mari      PDSVFENTRSAIAYGISPIIGTTGLSPSQINDLSIFAQKAEVAIIPNFSV
73748738_Dehalococcoides_sp.        AKASMPAIRIAAAHKVNLVIGTTGFSPEEISEIEQLAKTNDIILAPNFAL
123216321_Mycobacterium_tuberc      PDVVMGNLEFLIDNGIHAVVGTTGFTAERFQQVESLVAKPNTLIAPNFAI
145593931_Salinispora_tropic        PDAVMDNLHWCIDQGISAVVGTTGFTEARLERMRGLARKPGVVVAPNFGI
121535277_Thermosinus_carbox        PDTVMNNIRIAIKNGVCPVVGTTGLSEADVSEVRELCKQSRVIISPNFSV
29376120_Enterococcus_faecal        PEAAYPNTRFALEHGMAPVVGTTGFTEEQINELTNLSREKAILIAPNFAI
116873342_Listeria_welshi           PKVGYSNTKTILEHGVRAVVGTTGFTPEQIESLREIAETKKILIAPNFAV
125973685_Clostridium_thermo        PDATIRNAKIFSKMKVNIVVGTTGFTDFALKKLFVLTRKYHNCYAPNITL
20094858_Methanopyrus_kandle        REATLENAPKAARAGLDLVIGTTGFSDEDLRVLEHEIEEAGVVISPNMSL
118070755_Shewanella_woodyi         PEASLVHTDWCAKHGKAIVIGTTGFNHSQKEQISAYAEKTPVVMAPNMAV
89899565_Rhodoferax_ferrir          PEGTLAHVRMCRELGVNVVIGTTGFSDAQKAEITAATRDIAIVMAPNMSV
120613186_Acidovorax_avenae         PEGTLAHLAVCAERGVKAVIGTTGFTEAQKAEIEAFAQRTAVVMAPNMSV
20093259_Methanosarcina_acetiv      AGATIVNAPIAAGCGVNLIIGTTGLTPEQRKVIDETIQKNKVVISPNYSV
116754136_Methanosaeta_thermo       PSAALENIHAAADTGVALVVGTTGFSEEQLSIIEEMIKSAGIVISPNFSL
                                                      ::****        :            *:

116055387_Ostreococcus_tauri]       QVVAFQAAMKLMAEQFPGLTVTESHQSSKKDT-SGTAKAIVASFND-LGC
18406430_Arabidopsis_thalia         QVVAFLAAMEIMAEQFPGLDVMESHQASKLDA-SGTAKAVISCFQE-LGV
42572737_Arabidopsis_thalia         QVVAFLAAMEIMSEQFPGLEVMESHQASKLDA-SGTAKAVISCFQK-LGV
CM2591_Cyanidioschyzon_merolae      QIVAFQALVELLGREFPNLEVIESHQASKLDT-SGTAKAVVASMLQSWGI
ISO00167059_Isochrysis_galbana      Q-------------------------------------------------
121542607_Candidatus_Desulf         QVVGFQAMLAHAADTFPGMTVRESHQKGKADT-SGTARAVVHSFNR---L
123966140_Prochlorococcus_mari      GMVLLQQAASVAAKFYDNIELIEMHHNQKADSPSGTCIKTAEMIEEYPKQ
73748738_Dehalococcoides_sp.        GAIIMVHLAQVASRFLSSAEVIELHHDKKLDSPSGTALVTVASMLEARGE
123216321_Mycobacterium_tuberc      GAVLSMHFAKQAARFFDSAEVIELHHPHKADAPSGTAARTAKLIAEARKG
145593931_Salinispora_tropic        GAVLMMQFAERAASHFESAEIIEQHHPRKLDAPSGTATHTARLIAAARAA
121535277_Thermosinus_carbox        GAVLMMQLAQEAAKYFPHVEIIELHHDQKLDAPSGTALRTAELIMQNRGS
29376120_Enterococcus_faecal        GAVLMMQFAQKAAQYFPDVEIIELHHDNKLDAPSGTAIKTAEMIQEVRPA
116873342_Listeria_welshi           GAVLMMQFAQKAAKYFPNVEIIELHHDNKLDAPSGTAVKTAEMMAENRTF
125973685_Clostridium_thermo        GVNVLMLLTNLAASILNNFQITEIHHKRKVDVPSGTAIKIAGEIKKGLDS
20094858_Methanopyrus_kandle        GVNLLFELTRQLARVLGDFEIVEIHHRHKVDAPSGTALELAAIIEEELGK
118070755_Shewanella_woodyi         GVNLLWKLLEVAAEVMGDIEIIEGHHRYKKDAPSGTALKMGEVIAETLGR
89899565_Rhodoferax_ferrir          GVNVTLKLLEMAAKALATIEIIEAHHRHKVDAPSGTALKMGEVVAGALGR
120613186_Acidovorax_avenae         GVNVTFKLLEMAARALSTIEIIEAHHRHKVDAPSGTALKMGEVIAQALGR
20093259_Methanosarcina_acetiv      GVNVFFKIVREAAKYLSDIEIIEAHHNQKKDAPSGTALRAADVISEALGG
116754136_Methanosaeta_thermo       GVNVFWKLVEMAARSLSDVEVIEAHHRKKKDAPSGTAMRTVEILSRSLGI


116055387_Ostreococcus_tauri]       DFDVSQIELGVPEEHLLGHAFHTYR-LTSADGTVSFEFQHNVCGRSIYAE
18406430_Arabidopsis_thalia         SYDMDQIQLGVPEEHISGHAFHLYH-LTSPDETVSFEFQHNVCGRSIYAE
42572737_Arabidopsis_thalia         SYDMDQIQLGVPEEHVSGHAFHLYH-LTSPDKTVSFEFQHNVCGRSIYAE
```

```
CM2591_Cyanidioschyzon_merolae      TGFGGEQDIGVPESCLQGHAFHTYR-LTSPDGSVRFVFQHNVCGRRVYAE
ISO00167059_Isochrysis_galbana      --------------------------------------------------
121542607_Candidatus_Desulf         GVHFSEDQIGIPEEHLGGHGWHTYT-LTSPDRTVTFEFKHNINGRDIYAD
123966140_Prochlorococcus_mari      YNEGLVKETGLNIHSIRLPGLLAHQVVMMGSPGETYTIRHDTIDRKAYMP
73748738_Dehalococcoides_sp.        AFNKPPKENGIRVHSVRLPGLLAHQEVIFGAAGQTLTIRHDAFSRECYMP
123216321_Mycobacterium_tuberc      LPPNPDATSGIPVHAVRLAGLVAHQEVLFGTEGETLTIRHDSLDRTSFVP
145593931_Salinispora_tropic        AGQGPAPDAGVRVHSVRATGLVAHQEVLFGATGETLTIRHDSYDRASFMP
121535277_Thermosinus_carbox        MQQGHPAEVGIRIHSVRLPGYVAHQEVIFGGLGQTLTIRHDSISRESFMP
29376120_Enterococcus_faecal        KKQGNPQEVGLRIHSVRLPGLVAHQQVQFGSVGEGLTIRHDSYDRRSFMT
116873342_Listeria_welshi           VKQGAEDEVGMRIHSVRLPGLVAHQEVIFGAEGQGLTIRHDSYDRISFMS
125973685_Clostridium_thermo        AGVSIKEKV-IPINAVRAGGVVGRHEVMIVGEDDKIEISHESFSRRAFAL
20094858_Methanopyrus_kandle        GEKVFGREGEIGVLAVRGGEVVGDHTVMALGEYERIELTHRALSRDAFAK
118070755_Shewanella_woodyi         DLDKCAVYGTIGFSTIRAGDIVGEHTALFADIGERIEITHKASSRMTFAN
89899565_Rhodoferax_ferrir          DLKDCAVYASIGFATIRGGDIVGDHTVLFAGIGERIEISHKSSSRATYAQ
120613186_Acidovorax_avenae         DLKDCAVYGTIGFAAVRGGDIVGDHTVLFAGTGERIEISHKSSSRSGYAQ
20093259_Methanosarcina_acetiv      KEYVCGREG-IGIHAVRGGDITGDHTVLFVGNSERIEIRHMAHSRQIFAK
116754136_Methanosaeta_thermo       EDVRHGREG-IGVHAVRAGDIVGDHTVLFAGPGERIEIKHQAHSRSAFAR

116055387_Ostreococcus_tauri]       GTVDAVGFLKRKTLYDMIDVL
18406430_Arabidopsis_thalia         GTVDAVLFLAKQRIYNMIDVL
42572737_Arabidopsis_thalia         GTVDAVLFLAKKRIYNMIDVL
CM2591_Cyanidioschyzon_merolae      GTIDAVCFLDRQKIYDMIDVL
ISO00167059_Isochrysis_galbana      ---------------------
121542607_Candidatus_Desulf         GTLDAVVFLHQGTVFSMIDVL
123966140_Prochlorococcus_mari      GVLQAIRKIGKSLIYGLEKLI
73748738_Dehalococcoides_sp.        GVLLAIKEIVHGFVFGLDKLL
123216321_Mycobacterium_tuberc      GVLLAVRRIAEGLTVGLEPLL
145593931_Salinispora_tropic        GVLLAVRKVRTGLTVGLDALL
121535277_Thermosinus_carbox        GVLLACKRVLTGLVYGLENIM
29376120_Enterococcus_faecal        GVALACRQVVQELLYGLEQML
116873342_Listeria_welshi           GVALSIRKTKETLIYGLENIL
125973685_Clostridium_thermo        GAIKAIEFIHDVGYYEMSDVL
20094858_Methanopyrus_kandle        GALVAAKFVVEPGIYSMRDVL
118070755_Shewanella_woodyi         GAMRAASWLVEAGLYDMQQVL
89899565_Rhodoferax_ferrir          GSLRALRFLSQAGLYDMADVL
120613186_Acidovorax_avenae         GSLRAARFLAATGLFDMFDVL
20093259_Methanosarcina_acetiv      GAVRAAEWICKPGIYSMDDVL
116754136_Methanosaeta_thermo       GALRAARWVVKPGIHSMEEVL
```

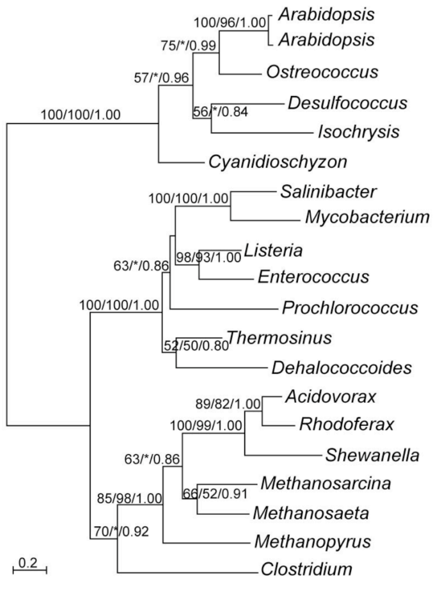

Figure 16. Molecular phylogeny of dihydrodipicolinate reductase (dapB). P-value = 0.995 from AU test for the presented tree. AU test was also performed on an alternative topology that enforces a monophyly of

Desulfococcus, cyanobacterial (i.e., *Prochlorococcus* since this gene is very rarely identified in cyanobacteria in our similarity searches), and photosynthetic eukaryotic sequences. P-value < 0.001 from AU test for the alternative topology. See text for more detailed explanation.

---

17. Leucyl-tRNA synthetase (D)

```
CLUSTAL X (1.83.1) multiple sequence alignment


60463472_Dictyostelium_discoi       FYSLSQFPYPSGA-LHMGHVRVYTISDCIARLKRMQGYDVIHPMGWDAFG
19683964_Homo_sapien                FYVLSMFPYPSGK-LHMGHVRVYTISDTIARFQKMRGMQVINPMGWDAFG
89306112_Tetrahymena_thermoph       YYVLSQFPYPSGN-LHMGHARVYYISDVISRFEKMKGKNVINPMGWDAFG
15791045_Halobacterium_sp.          -YVLAMFPYTSGQ-LHMGHVRNYAITDAFARYRRMDGEDVLHPMGWDSFG
11595633_Neurospora_crassa          -YVLPMFPYPSGH-LHLGHLRVYTIADVIARFQTLQGHKVLLPMGWDAFG
53715865_Bacteroides_fragil         FYVLNMFPYPSGAGLHVGHPLGYIASDIYARYKRLQGFNVLNPMGYDAYG
15826893_Mycobacterium_leprae       LFVQDMFPYPSGDGLHVGHPLGYIATDVYARYFRMTGHNVLHAMGFDAFG
106884629_Clostridium_phytofer      YYVLDMFPYPSGVGLHAGHPEGYTATDIVSRMKRMQGYNVLHPMGYDSFG
76788931_Chlamydia_trachoma         YYVLDMFPYPSGAGLHVGHLIGYTATDIVARYKRAQGFSVLHPMGWDSFG
33235997_Chlamydophila_pneumo       YYVLDMFPYPSGAGLHVGHLIGYTATDIVARYKRARGFSVLHPMGWDSFG
46446681_Parachlamydia_sp.          YYVLDMFPYPSGAGLHVGHVVGYTATDIIARYMRTKGYNVMHPMGWDSFG
PLO00010890_Pavlova_lutheri         --------------------------------------------------
21674468_Chlorobium_tepidu          YYVLDMFPYPSGSGLHVGHLEGYTATDIMARYKRCQGHNVLHPMGWDAFG
42527840_Treponema_dentic           LYILDMFPYPSGDGLHVGHPEGYTATDIYSRFLRMSGYNVLHPMGFDSFG
CM3072_Cyanidioschyzon_merolae      YYILDMFPYPSGAGLHVGHPEGYTATDILARYKRMRGYNVLHPMGWDAFG
87311335_Blastopirellula_marin      MYVLDMFPYPSGDGLHVGHPEGYTATDIVCRYARMQGKSVLHPMGFDAFG
83816700_Salinibacter_ruber         FYVLDMFPYPSGSGLHVGHPEGYTATDIVARYKRKQGFNVLHPMGWDAFG
150385918_Victivallis_vadensis      YYVLDMFPYPSGAGLHVGHPEGYTATDIICRYRRMKGDNVLHPMGWDAFG
10175904_Bacillus_halodu            FYALDMFPYPSGAGLHVGHPEGYTATDILSRMKRMQGYNVLHPMGWDAFG
116058126_Ostreococcus_tauri        FYALDMFPYPSGAGLHVGHPEGYTATDIVARYKRMTGHNVLHPMGWDAFG
7267192_Arabidopsis_thalia          FYVLDMFPYPSGAGLHVGHPLGYTATDILARLRRMQGYNVLHPMGWDAFG
2314727_Helicobacter_pylori         KYILSMLPYPSGE-IHMGHVRNYTIGDALARYYRLHHYNVLHPMGFDSFG
6459971_Deinococcus_radiod          FYALTMFPYPSGN-LHIGHWYANVAPDARARWLRMRGYNVLFPMAFDAFG
83367833_Rhodobacter_sphaeroi       YYVLEMFPYPSGR-IHMGHVRNYTMGDVVARQKAAAGYSVLHPMGWDAFG
48766678_Rhodospirillum_rubrum      YYVLEMFPYPSGR-IHMGHVRNYTLGDVVARFKRAQGFNVLHPMGWDAFG
15889997_Agrobacterium_tumefa       YYVLEMFPYPSGR-IHMGHVRNYAMGDVVARYKRARGFNVLHPMGWDAFG
19712730_Fusobacterium_nuclea       YYVLSMLPYPSGK-LHVGHARNYTIGDVISRYKRMKGYNVLQPMGWDSFG
106888735_Roseiflexus_sp            RYILEMFPYPSGD-LHIGHLKNYVIGDALTRYYVIRGYDVLHPFGWDAFG
46130187_Synechococcus_elonga       FYALSMFPYPSGN-LHMGHVRNYTITDAIARVKRRQGFRVLHPMGWDAFG
53763953_Anabaena_variab            FYALSMFPYPSGS-LHMGHVRNYTITDVIARLKRMQGYRVLHPMGWDAFG
23129999_Nostoc_puncti              FYALSMFPYPSGS-LHMGHVRNYTITDVIARFKRMQGYRVIHPMGWDAFG
16128625_Escherichia_coli           YYCLSMLPYPSGR-LHMGHVRNYTIGDVIARYQRMLGKNVLQPIGWDAFG
30249126_Nitrosomonas_europa        YYCLSMFPYPSGK-LHMGHVRNYTIGDVLSRYRRMQGYNVMQPMGWDAFG
53762629_Ralstonia_eutrop           FYACSMLPYPSGK-LHMGHVRNYTINDVMARYLRMNGNNVLMPMGWDAFG
126358263_Pseudomonas_putida        YYCLSMFPYPSGK-LHMGHVRNYTIGDVIARYQRMLGKNVLQPMGWDAFG
7227154_Neisseria_mening            YYCLSMFPYPSGK-LHMGHVRNYTIGDVLSRFKLLNGFNVMQPMGWDAFG
15642942_Thermotoga_mariti          YYALVMFPYPSGT-LHVGHVKNYVIGDIVARYKRMRGYNVLHPFGYDAFG
48846878_Geobacter_metall           YYLLEMFPYPSGR-IHMGHVRNYSIGDVVGRFKRLRGFNVLHPMGWDAFG


60463472_Dictyostelium_discoi       LPAENAAIDKQVSPSEWTNLNISSMRDQLKLLNFQFDWDRELSTCNKEYY
19683964_Homo_sapien                LPAENAAVERNLHPQSWTQSNIKHMRKQLDRLGLCFSWDREITTCLPDYY
89306112_Tetrahymena_thermoph       LPAENAAIQR----------NIVILQI------------QKLFTCREDYY
15791045_Halobacterium_sp.          LPAENAANERDTNPREWTERCIDQMRDQFEALGFGYDWEREITTCDPDYY
11595633_Neurospora_crassa          LPAENAAIERGINPATWTKANIAKMKEQLGHMNGSWDWNCELATCDPDFY
53715865_Bacteroides_fragil         LPAEQYAIQTGQHPAITTVNNINRYREQLDKIGFSFDWNREIRTCDPEYY
15826893_Mycobacterium_leprae       LPAEQYAMQTGTHPRILTEANVVNFRHQLGRLGLGHDSRRTFSTTDVEFY
106884629_Clostridium_phytofer      LPAEQYAVSTGNHPNGFTEKNIESFSKQLKELGFDYDWSKMIATSDPKFY
76788931_Chlamydia_trachoma         LPAEQYAIRTGTHPRETTEKNIANFKKQLTAMGFSYDESREFATSDPEYY
33235997_Chlamydophila_pneumo       LPAEQYAIRTGTHPKVTTQKNIANFKKQLSAMGFSYDEGREFATSDPDYY
46446681_Parachlamydia_sp.          LPAEQYAIRTGTHPAESTQENINNYRRQLRALGFSYDWNRELATSDPNYY
PLO00010890_Pavlova_lutheri         --------------------------------------------------
21674468_Chlorobium_tepidu          LPAEQFAIKTGTHPRLTTEKNVASFRETLKSMGFSYDWSREINTTDPNYF
42527840_Treponema_dentic           LPAENYAIKTGIHPLITTRKNMETFRKQIKSIGLSYDWDREISTSEESYY
CM3072_Cyanidioschyzon_merolae      LPAEQFALDTGIHPAVATERNITRFRKQLKSLGFSYDWKREINTTSPEYY
87311335_Blastopirellula_marin      LPAEEHAIKTGTPPRVQTEKNIANFTRQLKMLGFSYDWERQIATTDIEYV
83816700_Salinibacter_ruber         LPAEQYALKTNTHPRETTEKNIAQFKRQLKRLGFSYDWQREINTTDPDYY
150385918_Victivallis_vadensis      LPAEQYAVETGTHPEVTTQKNVDNFRRQIKSLGFSYDWDREINTTDPGYF
10175904_Bacillus_halodu            LPAEQYAIDTGNSPAEFTEKNINTFRRQIKSLGFSYDWDREVNTTDPDYY
```

```
116058126_Ostreococcus_tauri        LPAEQYAIETGTHPSQTTVKNVGRFREQLQSLGFSYDWDREVATCDAKYY
7267192_Arabidopsis_thalia          LPAEQYAIETGTHPKTTTLKNIDRFRLQLKSLGFSYDWDRELSTTEPDYY
2314727_Helicobacter_pylori         MPAENAAIKHGIHPKTWTYENIENMQKEFEALGFSFSKNREFATSDPDYT
6459971_Deinococcus_radiod          LPAENAAIKNNTNPATWTYANIERMTGQFSRMGTMIDWSRKFATCDPEYY
83367833_Rhodobacter_sphaeroi       MPAENAAMERGGHPKDWTYGNIADMRAQMKPLGLSIDWSREFATCDPEYY
48766678_Rhodospirillum_rubrum      LPAENAAIQRGVHPATWTRQNIAIMREQFKPMGLSIDWSREVSTCEPAYY
15889997_Agrobacterium_tumefa       MPAENAAMQNKVHPKDWTYQNIATMRGQLKSMGLSLDWTREFATCDVEYY
19712730_Fusobacterium_nuclea       LPAENAAIQNGTHPAIWTKSNIENMRRQLKLMGFSYDWEREIASYTPEYY
106888735_Roseiflexus_sp            LPAENAAIKYGRHPREWTYSNIAESKKSLEIAGIMYDWSREVTTCNPDYY
46130187_Synechococcus_elonga       LPAENAAIDRGVQPADWTYQNVAQMREQLKQLGLSYDWDREVTTCSPDYY
53763953_Anabaena_variab            LPAENAAIDRGVPPANWTYQNITQMRQQLQRLGLSIDWDSEVATCSPDYY
23129999_Nostoc_puncti              LPAENAAIDRGVPPAKWTYQNITQMRQQLQRLGLSIDWECELATCSPDYY
16128625_Escherichia_coli           LPAEGAAVKNNTAPAPWTYDNIAYMKNQLKMLGFGYDWSRELATCTPEYY
30249126_Nitrosomonas_europa        LPAENAAIQKGVPPAKWTYDNIAYMRSQLQSLGFAIDWQRELATCDPQYY
53762629_Ralstonia_eutrop           MPAENAALNNGVAPAAWTYDNIAYMKKQMQSMGLAIDWSREVATCSPDYY
126358263_Pseudomonas_putida        MPAENAAMKNNVAPAKWTYENIDYMKTQLKSLGLAIDWAREVTTCKPDYY
7227154_Neisseria_mening            MPAENAAMKNNVAPAAWTYDNIEYMKTQLKSLGFAIDWARETATCKPEYY
15642942_Thermotoga_mariti          LPAENAAIEKGIHPEEWTRKNIATIRQQVKKLGISYDWSREIATCDEEYY
48846878_Geobacter_metall           MPAENAAIQHKSHPAKWTYENIAYMRSQLKKMGLSYDWDRELATCDLDYY


60463472_Dictyostelium_discoi       RWTQEIFLRLLLAYRKSATVNWDPIDQTVLANEQVDQGRSWRSNAIVEKK
19683964_Homo_sapien                KWTQYLFIKLYLAYQKEALVNWDPVDQTVLANEQVDHGCSWRSGAKVEQK
89306112_Tetrahymena_thermoph       KWTQWIFIQLLLAYKKEAEVNWDPIDQTVLANEQVDQGRSWRSGAKVEKK
15791045_Halobacterium_sp.          KWNQWLFTEFRLVDRRAATVNWCPSCETVLADEQVEDDECWRCDTPVTER
11595633_Neurospora_crassa          KHTQKIFLALHLAYQAEAEVNYDPVDKTVLANEQVDNGCSWRSGARVEKR
53715865_Bacteroides_fragil         HWTQWAFIKMFIAYLGNTMVNWCPALGTVLANDEVVDGVSERGGYPVIQK
15826893_Mycobacterium_leprae       KWTQWIFLQIYLVYRADSMVNWCPGLGTVLANEEVTDGRSDRGNFPVFRK
106884629_Clostridium_phytofer      KWTQWIFKQLYYAKYIDMPVNWCEELGTVLSNDEVIDGKSERGGYPVIRK
76788931_Chlamydia_trachoma         KWTQKLFLILYLAYMADMAVNYCPELGTVLSNEEIENGFSVDGGYPVERR
33235997_Chlamydophila_pneumo       HWTQKLFLFLYLAYMADMAVNYCPELGTVLSNEEVENGFSIEGGYPVERK
46446681_Parachlamydia_sp.          KWTQWIFTKLYLAYEAEMLVNYCPALGTVLANEEIENGKTKDGGHPVERR
PLO00010890_Pavlova_lutheri         --------------------------------------------------
21674468_Chlorobium_tepidu          KWTQWIFLKLYLAYISEVDVNWCEELKVVLANEEVDKIA---DGYTVVRR
42527840_Treponema_dentic           KWTQWIFLQLFLAYEKEAPINWCPSCLTGLANEEVKDGKCERCGAQIQRK
CM3072_Cyanidioschyzon_merolae      RWTQWIFLQLYLAYQAEIPVNWCPALGTVLANEEVIDGRSERGGHPVIRK
87311335_Blastopirellula_marin      RWTQWIFLQVYLAYLNEAPVNWCPALGTVLANEEVIDGKSERGGHPVQRI
83816700_Salinibacter_ruber         KWTQWIFLQLYLAYQSEEPVWWCEELGTVLANEEVIDGKSERGGYPCERV
150385918_Victivallis_vadensis      KWTQWIFMQLHLAYLDEVAVNWCPALGTVLANEEVIDGRSERGNHPVERR
10175904_Bacillus_halodu            KWTQWIFIQLYLAYIDEVAVNWCPALGTVLANEEVIDGKSERGGHPVERR
116058126_Ostreococcus_tauri        KWTQWIFLRLLLAYQAEVPVNWCPALGTVLANEEVIDGLSERGNHPVIRK
7267192_Arabidopsis_thalia          KWTQWIFLQLYLAYQAEVPVNWCPALGTVLANEEVVDGVSERGGHPVIRK
2314727_Helicobacter_pylori         KFEQRFFIDLWLIYRKKAMLNWCPNDKTVLANEQVIDGRCWRCDTEVIQK
6459971_Deinococcus_radiod          RWNQWFFIEFWLAYKKGGLVNWCPKDQTVLANEQVVNGHCERCGTAVERR
83367833_Rhodobacter_sphaeroi       GQQQAMFIDMLLIYRKNAVVNWDPVDMTVLANEQVIDGKGWRSNAPVERR
48766678_Rhodospirillum_rubrum      RHEQKMFLDFLLAYRRESWVNWDPVEHTVLANEQVIDGKGWRSGAPVERR
15889997_Agrobacterium_tumefa       HRQQALFVDFMLVYRKQSKVNWDPVDHTVLANEQVIDGRGWRSGALVEQR
19712730_Fusobacterium_nuclea       KWNQWLFKRMYLIYKKKSLVNWCPDCQTVLANEQVEDGMCWRSKTHVIQK
106888735_Roseiflexus_sp            RWNQWLFLLLYLAYRAKATVNWDPVDQTVLANEQVDEGRSWRSGAKVEKR
46130187_Synechococcus_elonga       RWTQWLFLQFFLAYQKEATVNWDPIDQTVLANEQVDEGRSWRSGAKVERR
53763953_Anabaena_variab            KWTQWIFLQFLLAYQKEAAVNWDPIDQTVLANEQVDEGRSWRSGAIVERK
23129999_Nostoc_puncti              KWTQWIFLQFLLAYQKEAAVNWDPIDQTVLANEQVDEGRSWRSGAIVERK
16128625_Escherichia_coli           RWEQKFFTELYLVYKKTSAVNWCPNDQTVLANEQVIDGCCWRCDTKVERK
30249126_Nitrosomonas_europa        RWNQWLFLRMLIAYQKTQVVNWDPVDQTVLANEQVIDGCGWRTGAVVEKR
53762629_Ralstonia_eutrop           RWNQWLFLKMLIAYRKTGTVNWDPVDQTVLANEQVIDGRGWRSGAIVEKR
126358263_Pseudomonas_putida        RWEQWLFTRLFIIYRKNGTVNWDPADQTVLANEQVIDGRGWRSGALIEKR
7227154_Neisseria_mening            RWEQWLFTKLFIVYRKNGTVNWDPVDQTVLANEQVIDGRGWRSGALIEKR
15642942_Thermotoga_mariti          KWTQWIFLQLYLAYKKKAAVNWCPKCKTVLANEQVKDGKCERCGTSVTIR
48846878_Geobacter_metall           KWEQKVFLEMYLAYKKTSYVNWCPKCETVLANEQVEDGACWRCDSEVTQK


60463472_Dictyostelium_discoi       EMKQWFYKITSMADRLTDDLDQLWSDEIKNMQKEWIGRSYGHLIEFQITV
19683964_Homo_sapien                YLRQWFIKTTAYAKAMQDALADLW-YGIKGMQAHWIGDCVGCHLDFTLTA
89306112_Tetrahymena_thermoph       MLNQWFIKTTEYGKELFQDLSDLWPQAVQEMQRGWIGFSEGTQIKFEIEV
15791045_Halobacterium_sp.          DLDQWFFETTAYADELLDGLDELWPSNVRDMQRNWVGRTDGVEVPFTVVA
11595633_Neurospora_crassa          KLKQWFLKISEFRESLLKDLETLWPERVLAMQKNWLGKSKGATVKFPIEV
53715865_Bacteroides_fragil         VMRQWCLRVSAYAQRLLDGLETVWTDSLKETQRNWIGRSEGAEMNFKFTI
15826893_Mycobacterium_leprae       RLRQWMMRITAYADRLLDDLDLLWPEQVKTMQRNWIGRSSGATVLFSVEV
106884629_Clostridium_phytofer      NMKQWVINQPAFAEKLLEGLEEIWPESTKDMQRHWIGKSEGVEVDLEFSI
76788931_Chlamydia_trachoma         MLRQWVLRITAFADQLLEGLDELWPESVKQLQKNWIGKSSGASVNFAIEV
33235997_Chlamydophila_pneumo       MLRQWILKITAYADKLLEGLDALWPENVKQLQKNWIGKSEGALVTFHLEA
46446681_Parachlamydia_sp.          PLKQWILKITAYADRLLQDLDLLWPESLKKLQINWIGKSEGAYVQFIFSV
PLO00010890_Pavlova_lutheri         --------------------------------------------------
```

```
21674468_Chlorobium_tepidu         PLRQWVLKITAYAERLLKDLDEVWPENVKQMQRNWIGRSEGMEIDFELRV
42527840_Treponema_dentic          NLRQWILKITEYAERLLEDLDELWPESIKIMQKNWIGKSTGAEVDFAIKV
CM3072_Cyanidioschyzon_merolae     PMRQWVLRITAYAERLLKDLDLLWPENVKEMQRNWIGRSEGVEVTFPITV
87311335_Blastopirellula_marin     PLKQWMMRITDYADRLESDLEGLWSEGIKARQRNWIGRSTGAEVDFYLRV
83816700_Salinibacter_ruber        PMRQWVLKITEYADRLLEGLEDLWPESTKEMQRNWIGRSEGANVYFDLEV
150385918_Victivallis_vadensis     PMKQWMLRITEYAERLLQDLDELWPEGVKEMQRNWIGKSVGAEVIFKVTV
10175904_Bacillus_halodu           PMKQWMLKITEYADRLLEDLEELWPESIKDMQRNWIGRSEGAEVTFSITV
116058126_Ostreococcus_tauri       PMKQWMLKITEYADRLLEDLDELWPDSIKEMQRNWIGKSEGAQLGFSLEV
7267192_Arabidopsis_thalia         PMRQWMLKITAYADRLLEDLDELWPESIKEMQRNWIGRSEGAELNFSITV
2314727_Helicobacter_pylori        ELYQYYLKITNYAEELLKDLEALWPSQVLIMQKNWIGKSSGLQFGFKIEV
6459971_Deinococcus_radiod         NLSQWYLKITDYAEELL-DFSATMPEKVRAMQTNWIGKSVGAEVTFD-TV
83367833_Rhodobacter_sphaeroi      ELTQWFFRISDYAGELLEALDRLWPEKVRLMQANWIGQSRGLQFAFSLEV
48766678_Rhodospirillum_rubrum     KLSQWFLRITRYAEDLLAAIGDLWPDKVRLMQTNWIGRSEGARVRFGLEV
15889997_Agrobacterium_tumefa      ELTQWFFRITDFSQDLLDELDELWPEKVRLMQKNWIGRSEGLSLRWQITV
19712730_Fusobacterium_nuclea      ELEQWFFKITDYADELLEGHEEIWPEKVLTMQKNWIGKSFGTELKLKLPI
106888735_Roseiflexus_sp           ELEQWFFRITAYAERLLNDLDKLWPENVKTMQRNWIGRSEGAEVTFLLVV
46130187_Synechococcus_elonga      QLKQWFLKITDYAEELLQDLDQLWPERVRLMQANWIGKSTGAYLEFPVKV
53763953_Anabaena_variab           LLRQWFLKITDYAEELLNDLDKLWPERVKLMQANWIGKSVGAYLEFPIAV
23129999_Nostoc_puncti             LLRQWFFKITDYAEELLNDLDKLWPERVKLMQANWIGKSTGAYLEFPIAV
16128625_Escherichia_coli          EIPQWFIKITAYADELLNDLDKLWPDTVKTMQRNWIGRSEGVEITFNLTV
30249126_Nitrosomonas_europa       EIPGYYLAITRYADELLADLEKLWPERVKTMQANWIGKSFGVDITFPLKV
53762629_Ralstonia_eutrop          EIPMYYLRITDYAEELLGDLDGLWPERVKIMQQNWIGKSVGVRFAFKLYV
126358263_Pseudomonas_putida       EIPMYYFRITDYADELLESLDELWPEQVKTMQRNWIGKSRGMEVQFPLKV
7227154_Neisseria_mening           EIPMYYFKITDYAEELLNDLDKLWPEQVKTMQRNWIGKSRGMTVRFALQV
15642942_Thermotoga_mariti         HLEQWFFKITDYAERLLNDLDKLWPEHVKTMQRNWIGKSTGAEIDFPIRV
48846878_Geobacter_metall          ELEQWFFRITDYAEELLEYTEKLWPERVLTMQRNWIGKSYGCEIDFPIKV


60463472_Dictyostelium_discoi      FTTRAETIYGVSFLAISPHHSEIEKRLELDQYLKEIQENLKTFNTGLTFY
19683964_Homo_sapien               YTATPEAIYGTSHVAISPSHRLLHGHSSLKEALRMALVPGKDCLTPVMAV
89306112_Tetrahymena_thermoph      YTTRPDTIYGVTFLALAYDHQLAQKEEDLADY--EKMVNKIGKVKDYYAV
15791045_Halobacterium_sp.         FTTRVDTIHGATYFALAPDHPLADRDDDVAHFVEEVADEPQGVETEFTAT
11595633_Neurospora_crassa         FTSRPDTLFGVQYIALAATHPSVKSDPELQAFLSTLPPHIRANPLAYHEE
53715865_Bacteroides_fragil        FTTRADTVFGVTFMVLAPESELVEQKAEVDAYLDRTKRSVSGVFSGSYAI
15826893_Mycobacterium_leprae      FTTRPDTMFGVTYLVLAPEHNLVTPGAAVAAYRRAIVKEKTGVFLGRYAT
106884629_Clostridium_phytofer     YTTCIETIYGITFMVLAPDGQIVENKEEVEAYINETLKTKSGILKGIYAI
76788931_Chlamydia_trachoma        FTTRPDTLIGVSFLALAPEHPLVEQKAVVAQYIKETQKTKSGVFTGSYAK
33235997_Chlamydophila_pneumo      FTTRLDTLLGVSFLVIAPEHPDLEQRDEVTAYVQESLKTKTGVFTGNYAK
46446681_Parachlamydia_sp.         FTTRPDTLFGVSYLVLAPEHPLVSQQQAVRAYQAQIARDKTGVFTGAYAV
PLO00010890_Pavlova_lutheri        --------------------------------------------------
21674468_Chlorobium_tepidu         YTTRPDTLFGATYLVISPEHPMAQQLVAVKKYIEQAKKEKTGVFTGSYAI
42527840_Treponema_dentic          YTTRPDTIFGATYMVLAPEHELVNQEKAVAAYIEEAAKNKTGVFTGAYAI
CM3072_Cyanidioschyzon_merolae     FTTRPETISGATYLVVAPEYPILAQREQVQRYVEQALKLKSGVFTGRYAQ
87311335_Blastopirellula_marin     YTTRPDTLFGATYMVIAPEHPLVEQSAAVKAYCDKAAKEKTGVFSGSYAI
83816700_Salinibacter_ruber        YTTRPDTLFGATYMVLAPEHELLEHREDVDEYCRQAL-DKTGVFTGGYAV
150385918_Victivallis_vadensis     YTTRPDTLFGATYMVLSPEHPLVECRAAVEAYQAEAATEKTGAFTGAYAI
10175904_Bacillus_halodu           FTTRPDTLFGATYMVLAPEHKLVEQKQAVESYKKEVAKEKTGVFTGAYAI
116058126_Ostreococcus_tauri       YTTRPDTLFGATYLVVAPEHPMISQKDEVVAYCETASKVKTGVFTGSYAT
7267192_Arabidopsis_thalia         YTTRPDTLFGATYMVVAPEHQLLEQKQQVEEYKDFASKDKTGVFTGCYAK
2314727_Helicobacter_pylori        FTTRADTIYGVTYIAIAPEHPLVQVSQEVSKMIKAIL-EKKGAFLGIYAI
6459971_Deinococcus_radiod         FTTRPDTLMGATFMVLAPEHAKVEQRAEVEAYVAAAG-EKTGVFTGSYAT
83367833_Rhodobacter_sphaeroi      YTTRPDTLLGASFAAISPDHPLARHDAGVAEFVAECRAEKKGFDTGIRVR
48766678_Rhodospirillum_rubrum     YTTRPDTLFGASFCAISANHPLAATNPDLAAFIAECGAEKKGFDTGLKVR
15889997_Agrobacterium_tumefa      YTTRPDTLFGASFLAIAADHPLAATNPAIAEFCDECRAEKKGIDTGVKVV
19712730_Fusobacterium_nuclea      FTTRIDTIYGVSYAVVAPEHPIVKDNPSIKDKVTEMKREKNGIDSGWHIE
106888735_Roseiflexus_sp           FTTRPDTLWGATFMVLAPEHPLAERRAEVEAYIARARREKSGVFLGSYAI
46130187_Synechococcus_elonga      FTTRPDTVYGVSYVVLAPEHPLVEQQTAVAAFAAEVSRPKRGVPTGGFVT
53763953_Anabaena_variab           YTTRPDTVYGVSYVVLAPEHPLTTQQAVVDTFIQEVTKPKRGVATGGKAI
23129999_Nostoc_puncti             YTTRPDTVYGVSYLVLAPEHPLTEQQAAVEVFIKEVSKPKRGIPTGGVAI
16128625_Escherichia_coli          YTTRPDTFMGCTYLAVAAGHPLAENNPELAAFIDECRMEKKGVDTGFKAV
30249126_Nitrosomonas_europa       FTTRADTLMGVTYVAVAAEHPVAHNQPDLVAFIESCRQEKKGMATGLYVL
53762629_Ralstonia_eutrop          FTTRADTIMGVTFCAVAAEHPLAANNPELAAFIDECKMEKKGMPTGLQVT
126358263_Pseudomonas_putida       FTTRPDTLMGATYVAVAAEHPLAQGNPALQAFIDECKQEKKGMATSLLVE
7227154_Neisseria_mening           YTTRPDTLMGATYVAVAAEHPLAADKPELQAFIAECKMEKKGVPTGRYVV
15642942_Thermotoga_mariti         FTTRPDTLWGVTFMALAPESPLVEKKEELQEFLERVKAEKEGFFLGRYAI
48846878_Geobacter_metall          FTTRQDTLYGATFMSLAPEHPMADRRAEVEAFIDKVKFEKEGVFTGSYCI


60463472_Dictyostelium_discoi      QPITKKYIPLILSNFVHADYGTGAVMGVPSHNRSDYQVAKQQNLKLLPVL
19683964_Homo_sapien               NMLTQQEVPVVILAKADLEGSLDSKIGIPSTSSEDTILAQTLGLAYSEVI
89306112_Tetrahymena_thermoph      NPLSGEQVPIYLVDYVLKDYGTGAVMGVPAHDDRDQEFAKKYQIPIKEVI
15791045_Halobacterium_sp.         NPATGAEIPVVVADFVLSDVGTGALMAVPAHDDRDHEFAQAHDLPVRQVV
11595633_Neurospora_crassa         TPEDTKSLPIYVAPYVLGDYGEGAVMGVPGHDLRDHAFEHHYDAPVRFVL
```

```
53715865_Bacteroides_fragil         NPLTNEPIPVWISDYVLAGYGTGAIMAVPAHDSRDYAFAKHFNLEIRPLI
15826893_Mycobacterium_leprae       NPATGKPVPIFVADYVLVGYGTGAVMAVPGHDPRDWDFAHKFHLPIVEVI
106884629_Clostridium_phytofer      NPVNGKEVPLFIGDFVLASYGTGAVMAVPSHDQRDFEYAMVHNIPMIQVI
76788931_Chlamydia_trachoma         HPVTHELIPIWIADYVLIGFGSGAVMGVPAHDERDLLFAEQFNLPVVSVL
33235997_Chlamydophila_pneumo       HPITGNLLPVWISDYVVLGYGTGVVMGVPAHDERDREFAEMFSLPIHEVI
46446681_Parachlamydia_sp.          NPVNHKEIPIWISDYVLMNVGTGAIMAVPAHDERDFEFAKTFKLPIIPVY
PLO00010890_Pavlova_lutheri         --------------------------------------------------
21674468_Chlorobium_tepidu          NPANGEALPVWISDFVLTSYGTGAIMSVPAHDSRDWEFAKKFGLPIREVI
42527840_Treponema_dentic           NPLTEQKIPVWISDYILISYGTGAIMAVPAHDERDFEFAAQFNLPKIKVV
CM3072_Cyanidioschyzon_merolae      NPVTGEHIPIWVADYVLGSYGTGAIMAVPGHDARDRAFAETFALPIRIVI
87311335_Blastopirellula_marin      NPVNDEKTPIWIADYVLISYGTGAIMAVPAHDERDFEFAQTFDIPVIAVV
83816700_Salinibacter_ruber         NPVNGEEIPIWVADYVLVSYGTGAIMAVPAHDERDHAFANKYDLPIREVV
150385918_Victivallis_vadensis      NPVNGVKIPIWIADYVLISYGTGAIMAVPAHDTRDFEFAQKFDLPIKCII
10175904_Bacillus_halodu            NPVNGEKVPIWIADYVLVSYGTGAIMAVPAHDERDYEFAKTFDLPIKEVV
116058126_Ostreococcus_tauri        HPLTGDKVPIWVADYVLGSYGTGSIMAVPAHDERDYDFAKAFDLPITEVV
7267192_Arabidopsis_thalia          NPANGDAIPIWVADYVLASYGTGAIMAVPAHDTRDNEFALKYNIPIKWVV
2314727_Helicobacter_pylori         HPLTKQKIPVWVANFALANYGSGALMGVPACDERDFEFANLYHIPIKVIT
6459971_Deinococcus_radiod          HPISGHQLPIWVADYVLVTYGTGSIMAVPAHDERDFAFAKKFDLPIREVI
83367833_Rhodobacter_sphaeroi       HPFDTAELPVYIANFILMDYGTGAIFGCPAHDQRDFEFASKYGLPIPPVF
48766678_Rhodospirillum_rubrum      HPFDPAELPVYVANFVLMEYGTGAIFGCPAHDQRDMDFARKYGLPVRAVV
15889997_Agrobacterium_tumefa       HPLDPSELPVYVANFVLMDYGTGAIFGCPSGDQRDLDFARKYGLPVVAVV
19712730_Fusobacterium_nuclea       NPVNKEIVPLWIADYVLMNYGTGAVMGVPAHDERDFVFAGKYNLPVKQVI
106888735_Roseiflexus_sp            NPVNDERIPIWIADYVLMGYGTGAIMAVPAHDQRDFEFARQFGLPVRVVV
46130187_Synechococcus_elonga       NPFTGQAVPIWIADYVLVEYGTGAVMGVPAHDSRDFAFAQRYGLPVQPVI
53763953_Anabaena_variab            NPFTGEEVPIWIADYVLYEYGTGAVMGVPAHDVRDFKFAQRYDLPIDFVI
23129999_Nostoc_puncti              NPFTGEEVPIWIADYVLYEYGTGAVMGVPAHDVRDFKFAKNYDLPINFVI
16128625_Escherichia_coli           HPLTGEEIPVWAANFVLMEYGTGAVMAVPGHDQRDYEFASKYGLNIKPVI
30249126_Nitrosomonas_europa        HPLTGERLPVWVANYVLMSYGEGAVMAVPAHDERDFDFARQHSLPIKPVI
53762629_Ralstonia_eutrop           HPLTGEQVDVWVGNYVLMSYGDGAVMGVPAHDERDFAFANKYKLPIRQVI
126358263_Pseudomonas_putida        HPLTGEKLPVWVANYVLMHYGDGAVMAVPAHDERDFEFAHKYNLPVKAVV
7227154_Neisseria_mening            NPLNGDKLEVWIANYVLWGYGDGAVMAVPAHDERDFEFATKYNLPKKQVI
15642942_Thermotoga_mariti          NPVTGERIPIYVANYILMEYGTGAIMGVPAHDQRDFSFAKKYGIPIKVVI
48846878_Geobacter_metall           NPVTNRRMPVFLANFVLLDYGTGAVMAVPTHDQRDFEFARKYDLPLQVVI
```

```
60463472_Dictyostelium_discoi       GKRQTNYRIHDWLISRQRYWGTPIPIIVCGDVPVPSDQLPVELPIDIQFT
19683964_Homo_sapien                EGDVTSDKLKDWLISRQRYWGTPIPIVHCGPTPVPLEDLPVTLPNIASFT
89306112_Tetrahymena_thermoph       K--------HDWLVSRQRYWGVPIPVIECGVLPVPEDQLPVILPEKLGNL
15791045_Halobacterium_sp.          AASAVQYQLRDWGVSRQRYWGTPIPIVHCGPVSVPDDDLPVELPEFV--H
11595633_Neurospora_crassa          AKETEKWRLRDWLISRQRYWGTPIPIVHCGTVPVPDEQLPVELPEVDEHG
53715865_Bacteroides_fragil         ERVKVNFRLRDAIFSRQRYWGEPFPVYYGMPYMIDESCLPLELPEVAKFT
15826893_Mycobacterium_leprae       AHARIEFKLRDWLFARQRYWGEPFPIIYGRPHALDEAALPVELPDVPYYD
106884629_Clostridium_phytofer      DRKSVNYHFREWIFARQRYWGEPVPVVYDEIHVLSDNELPLILPVLEDYG
76788931_Chlamydia_trachoma         NAAKIAYKLRDWLFSRQRYWGEPIPIIHGSCRPLRDYELPLLPPEIQDYE
33235997_Chlamydophila_pneumo       DRAKTMYRLRDWLFSRQRYWGEPIPIIHGTHRPLEDDELPLLPPNIDDYE
46446681_Parachlamydia_sp.          DKSATTYKLRDWLFSRQRYWGEPFPLLKGSVRLLDDEDELPLCPPAITNYT
PLO00010890_Pavlova_lutheri         -TR-----------------------------------------------
21674468_Chlorobium_tepidu          KKRKVNYKLRDWVFSRQRYWGEPIPIKHGTMRP--ETNLPLTLPEVEAYT
42527840_Treponema_dentic           AKRAVNYKLRDWIFSRQRYWGEPIPLVHCGIVPLNEHDLPLTLPQVESYT
CM3072_Cyanidioschyzon_merolae      DERKVNYKLRDWLFSRQRYWGEPFPIVFGEPLPVDEQALPVELPTTESIS
87311335_Blastopirellula_marin      DRTAVNYKLRDWLFSRQRFWGEPFPILKGKILPVPVDQLPVNLPHLEDFL
83816700_Salinibacter_ruber         EERTVNYQLQDWLFSRQRYWGEPFPIVFGEDKPVPEEELPVTLPDLDVFS
150385918_Victivallis_vadensis      AREAVKYKLRDWLFSRQRYWGEPFPIIHGSIELVNDSELPVNLPEMEDFS
10175904_Bacillus_halodu            STKKVTYRLRDWLFSRQRYWGEPIPVIHGTMSTVPEDELPLELPKMSEIS
116058126_Ostreococcus_tauri        SRKQTNYKLRDWLFARQRYWGEPFPVVFNLAVPVPESELPLILPETDNFS
7267192_Arabidopsis_thalia          RKKKVNYKLRDWLFARQRYWGEPIPILIGETIAISESELPLTLPELNDFT
2314727_Helicobacter_pylori         QKRVINYRLQDWGVSRQRYWGAPIPMIHCGIVP--ETQLPVTLPEDIVID
6459971_Deinococcus_radiod          RKAKTTYRLRDWLFARQRYWGTPIPFVHCGMQPVPEDQLPVKLPENVAFT
83367833_Rhodobacter_sphaeroi       VRGVTNYRLRDWGISRQRYWGCPIPVIHCGVVPEAKENLPVRLPDDVTFD
48766678_Rhodospirillum_rubrum      AEGVVQFRLRDWGVSRQRYWGCPIPIIHCGPVPVPEDQLPVLLPEDVTFD
15889997_Agrobacterium_tumefa       AERKVNFRLRDWGISRQRYWGCPIPVIHCGVVPVPKKDLPVKLPDDVTFD
19712730_Fusobacterium_nuclea       TKRTYKYRLKDWGISRQRYWGTPIPALYCGEVLEKDENLPVLLPDDIEFS
106888735_Roseiflexus_sp            QKGTVTYRLRDWLISRQRYWGTPIPMLHGTIKPVPEDQLPVLLPEVQDYL
46130187_Synechococcus_elonga       QQAHVQYRLRDWLISRQRYWGCPIPIVHCG--PVAAADLPVQLPDSVQFS
53763953_Anabaena_variab            AKERIQYRLRDWLISRQRYWGAPIPVIHCGIVPVPDKDLPVILPEEVEFT
23129999_Nostoc_puncti              AKVRVQYRLRDWLISRQRYWGAPIPVIHCGIVPVPDKDLPVQLPEEVEFT
16128625_Escherichia_coli           LERKVNYRLRDWGVSRQRYWGAPIPMVTGTVMPTPDDQLPVILPEDVVMD
30249126_Nitrosomonas_europa        REKRVRYRLRDWGISRQRYWGCPIPLIHCGVVPVADDQLPVVLPEDLVPD
53762629_Ralstonia_eutrop           DEKKITWRLRDWGISRQRYWGTPIPLIHCGVVPVPEQDLPVVLPEDLVPD
126358263_Pseudomonas_putida        RKSRTQFRLRDWGISRQRYWGCPIPIIHCGDVPVPEDQLPVTLPENVVPD
7227154_Neisseria_mening            AEPKTQYRLRDWGISRQRYWGCPIPIVHCGDVPVPADQLPVVLPENVVPD
15642942_Thermotoga_mariti          KKRSVQYKLRDWLISRQRYWGAPIPIIYCGVVPVPEEDLPVRLPKDVEFL
48846878_Geobacter_metall           QTKTVNFRLRDWGISRQRYWGNPIPVIYCGVVPVPEKDLPVVLPMDVEFT
```

```
60463472_Dictyostelium_discoi      GKGNLLNQLDETDTMDTFVDSSWYFLRFLDSSQSIFSSELVNRFMPIDVY
19683964_Homo_sapien               GKGPPLAMASETDTMDTFVDSAWYYFRYTDPPHSPFNTAVADYWMPVDLY
89306112_Tetrahymena_thermoph      QKNNPLAHIKETDTLDTFVDSSWYFLRFPDSSLQIFDSNLIKYWAPVDLY
15791045_Halobacterium_sp.         TTGNPLDAAEETDTMDTFLDSSWYFLRFASPDDAPFDTQRANDWLPVDEY
11595633_Neurospora_crassa         KKTNPLESQTDTDTMDTFVDSSWYYMRFIDAKEAPFSPEKAKVLTPVDLY
53715865_Bacteroides_fragil        ETGPPLGHATELNTMPGFAGSSAYYLRYMDPHEALVSPAVDQYWKNVDLY
15826893_Mycobacterium_leprae      SEPPPLAKATDTNVMPQWAGSSWYELRYTDPSERLCAKENEAYWMGVDLY
106884629_Clostridium_phytofer     KNGAPLENATETSTMPGSAGSSWYFMRYIDPQEEFANQELLKHWLPVDLY
76788931_Chlamydia_trachoma        GVGGPLAKVRETHTMPQWAGSCWYYLRFCDASAAPWAKEKEQYWMPVDLY
33235997_Chlamydophila_pneumo      GFGGPLAKAQETYTMPQWAGSCWYYLRFCDASQLPWSKEKESYWMPVDLY
46446681_Parachlamydia_sp.         GDGGPLTQIKDTNIMPQWAGSCWYYLRFCDPTEKAFSPEKEKYWLPVDLY
PLO00010890_Pavlova_lutheri        ------------------AGSCWYYLRFMDPTKAAWSKEAADAWLPVDLY
21674468_Chlorobium_tepidu         STGSPLANIEETNTMPQWAGSCWYYLRFIDPSDALVDPSLEQYWMNVDLY
42527840_Treponema_dentic          GTGSPLAAIDETNTMPQWAGSCWYYLRFIDPNEAFADKEKCDYWMPVDLY
CM3072_Cyanidioschyzon_merolae     GDGSPLAKIVETNTMPQWAGSCWYYLRFIDNAERLVDPVKERYWMPVDLY
87311335_Blastopirellula_marin     GRPPPLEKASETNTMPQWAGSCWYYLRFIDPDQVLIDREKEKAWMPVDLY
83816700_Salinibacter_ruber        GTPGPLATIEETNTMPQWAGSCWYYLRFIDPDEQLVDPEKEEYWMPVDLY
150385918_Victivallis_vadensis     GTGPPLAKAKETNTMPQWAGSCWYYLRYIDPPDQAWDPAKEKYWMPVDLY
10175904_Bacillus_halodu           GTGSPLANATETNTMPQWAGSCWYYLRYIDPERMIADPEKLKKWLPVDIY
116058126_Ostreococcus_tauri       GSGGPLANCEETNTMPQWAGSCWYYLRFIDPEDALIDGSLEKYWLPVDLY
7267192_Arabidopsis_thalia         GTGPPLSKAVETSTMPQWAGSCWYYLRFMDPPEALVDKEKEKYWSPVDVY
2314727_Helicobacter_pylori        GEGNPLEKHAETDTMDTFIQSSWYFLRYTTPENQAFDQNYLKYFMPVDTY
6459971_Deinococcus_radiod         PTGSPLKLDEETDTMDTFVDSSWYMYRYLSPDGGPFDPSKAG-LLPVDLY
83367833_Rhodobacter_sphaeroi      VPGNPLDRHPETDTMDTFVDSSWYYARFTAPATTPTDPEEAEYWMNVDQY
48766678_Rhodospirillum_rubrum     KPGNPLDHHPETDTFDTFFESSWYFARFCAPTDRAFEREAVDYWLPVDQY
15889997_Agrobacterium_tumefa      VPGNPLDRHSETDTMDTFVDSSWYYTRFTAPEDEPTDPQVANHWLPVDQY
19712730_Fusobacterium_nuclea      GNGNPLETSNDTDTMDTFVDSSWYFLRYCDPLNLPFSKEIVDKWTPVDQY
106888735_Roseiflexus_sp           PKGSPLAAAEDTDTMDTFVDSSWYFLRFCDADREIFSREAARQWMPVDQY
46130187_Synechococcus_elonga      GRGSPLAQLEETDTMDTFMCSSWYYLRYSDAPEIAFTKDKVNDWLPVDQY
53763953_Anabaena_variab           GRGSPLAQLEETDTMDTFIDSSWYFLRFTDAEAQVFESAKTNDWMPVDQY
23129999_Nostoc_puncti             GRCSPLTQLEETDTMDTFIDSSWYFLRFPDAEQQVFDSSKVNDWMPVNQY
16128625_Escherichia_coli          GITSPIKADPETDTFDTFMESSWYYARYTCPKEGMLDSEAANYWLPVDIY
30249126_Nitrosomonas_europa       GSGNPLAKTPETDTMDTFVDSSWYFIRYACPSAAMTDQR-ANYWLPVDQY
53762629_Ralstonia_eutrop          GTGNPLNKDPETDTMDTFIDSCWYYMRYTCPDAATMVDARNDYWMPMDQY
126358263_Pseudomonas_putida       GAGSPLARMPETDTMDTFVESSWYFARYASPDKGLVDPKAANHWLPVDQY
7227154_Neisseria_mening           GMGSPLAKMPETDTMDTFMESSWYFFRYMSPSDGMVDPAAAKYWGAVDQY
15642942_Thermotoga_mariti         PTGSPLSFHEETDTMDTFVDSSWYFLRYVNPEDKPFEPDDVNYWLPVDQY
48846878_Geobacter_metall          GEGSPLKKLDETDTMDTFVQSSWYFLRYCCPAAGPIDKARAEYWMSVDQY
                                                       *. *  *:                  :: *

60463472_Dictyostelium_discoi      VGGIEHAILHLLYSRFITKFLKDQQLIDHSEPFKVLLAQGLVKSEKMSKS
19683964_Homo_sapien               IGGKEHAVMHLFYARFFSHFCHDQKMVKHREPFHKLLAQGLIKGEKMSKS
89306112_Tetrahymena_thermoph      IGGMEHAILHLLYARFIHKFLHKQQFVPLHEPFKQLIVQGLVKSEKMSKS
15791045_Halobacterium_sp.         VGGDEHAVMHLLYSRFVTKAFADLDMLEHREPFAGLTTQGMVLGTKMSKS
11595633_Neurospora_crassa         IGGVEHAILHLLYSRFIYKFLMTSSFASSEEPFKRLITQGMVHGEKMSKS
53715865_Bacteroides_fragil        VGGTEHATGHLIYSRFWNKFLHDWGISVAEEPFQKLVNQGMIQGEKMSKS
15826893_Mycobacterium_leprae      VGGAEHAVLHLLYARFWHKVLYDLGHVSSREPYRRLINQGYIQAGKIGKS
106884629_Clostridium_phytofer     IGGPEHAVGHLMYSRIWNRYLYDKGLSPVKEPFKKLVHQGMILGIKMGKR
76788931_Chlamydia_trachoma        IGGAEHAVLHLLYARFWHQIFYEAGIVSTPEPFKKLVNQGLVLAEKMSKS
33235997_Chlamydophila_pneumo      IGGAEHAVLHLLYSRFWHRVFYDAGLVSTPEPFKKLINQGLVLAEKMSKS
46446681_Parachlamydia_sp.         IGGVEHAVLHLLYARFWHKVLYDCGYVHTLEPFQTLRNQGLVVADKMSKS
PLO00010890_Pavlova_lutheri        VGGAEHAVLHLLYARFWHKALFDAGLVAAPEPFARLVHQGMILGEKMSKS
21674468_Chlorobium_tepidu         IGGAEHAVLHLLYSRFWHKVLYDLGVVSTKEPFQRLFNQGMILGEKMSKS
42527840_Treponema_dentic          VGGTEHAVLHLLYARFWHKVLYDLGLVSTKEPFTRLINQGMITSAKMSKS
CM3072_Cyanidioschyzon_merolae     VGGVEHAVLHLLYARFWHKVLYDLGVVSTPEPFLRLVNQGMILGHKMSKS
87311335_Blastopirellula_marin     IGGAEHAVLHLLYARFWHKVLFDRGVVGTAEPFQKLVNQGMILGMKMSKS
83816700_Salinibacter_ruber        VGGSEHAVLHLLYARFWHKVLYDAGVVSTKEPFQTLVHQGMILGHKMSKS
150385918_Victivallis_vadensis     VGGAEHAVLHLLYSRFWHKVLFDLGLVSTKEPFRKLVNQGMILGAKMSKS
10175904_Bacillus_halodu           IGGAEHAVLHLLYARFWHKVLYDLGVVPTKEPFQKLYNQGMILGEKMSKS
116058126_Ostreococcus_tauri       VGGAEHAVLHLLYARFWHKVLYDIGVVSTKEPFQRLVSQGMILGHKMSKS
7267192_Arabidopsis_thalia         VGGAEHAVLHLLYSRFWHKVLYDIGVVSTKEPFKCVINQGIILGYKMSKS
2314727_Helicobacter_pylori        IGGIEHAILHLLYARFFTKALRDLGYLHLDEPFKQLITQGMVLKAKMSKS
6459971_Deinococcus_radiod         TGGIEHAILHLLYSRFWTKVMRDMGLTTQSEPFARLRNQGMVLGEKMSKS
83367833_Rhodobacter_sphaeroi      IGGIEHAILHLLYSRFFARAMQKTGHLPAKEPFNALFTQGMVTHTKMSKS
48766678_Rhodospirillum_rubrum     VGGVEHAVLHLLYSRFFTRALRDCGYLGVSEPFTGLFTQGMICHEKMSKS
15889997_Agrobacterium_tumefa      IGGIEHAILHLLYSRFFTRAMRETGHVGVKEPFKGLFTQGMVVHEKMSKS
19712730_Fusobacterium_nuclea      IGGVEHAVMHLLYARFFHKVLRDLGLLSSNEPFKRLLTQGMVLGEKMSKS
106888735_Roseiflexus_sp           IGGVEHAILHLLYARFITKVLYDEGLVPEDEPFKALFTQGMVQRVTMSKS
46130187_Synechococcus_elonga      VGGIEHAILHLLYSRFFTKVLRDRGLLSFDEPFKRLLTQGMVQGEKMSKS
53763953_Anabaena_variab           VGGIEHAILHLLYSRFFTKVLRDRGLLNFDEPFERLLTQGMVQGATMSKS
23129999_Nostoc_puncti             VGGIEHAILHLLYSRFFTKVLRDRGLLNFDEPFQRLLTQGMVQGATMSKS
```

```
16128625_Escherichia_coli          IGGIEHAIMHLLYFRFFHKLMRDAGMVNSDEPAKQLLCQGMVLASKMSKS
30249126_Nitrosomonas_europa       IGGIEHAILHLLYSRFWSKVMRDLGLVSFDEPFANLLTQGMVLNGTMSKS
53762629_Ralstonia_eutrop          IGGIEHAILHLLYARFWTKVMRDLGLVKFDEPFTNLLTQGMVLNEKMSKS
126358263_Pseudomonas_putida       IGGIEHAILHLLYARFFHKLMRDEGLVTSNEPFKNLLTQGMVVAEKMSKS
7227154_Neisseria_mening           IGGIEHAILHLLYARFFTKLMRDEGLVNVDEPFERLLTQGMVVCEKMSKS
15642942_Thermotoga_mariti         IGGVEHAVLHLLYSRFVTKVLHDLGYLNFDEPFTNLFTQGMIYKAKMSKS
48846878_Geobacter_metall          IGGIEHAVLHLLYARFFTKALRDLGYVTVDEPFTNLLTQGMVIKAKMSKS
                                    ** ***  **:* *:  :           **    :  ** :   .:.*

60463472_Dictyostelium_discoi      KLNGIDPKEIIDKYGSDTLKTYILFKAPPEKSLDWDTQGIEGCKKWLTRI
19683964_Homo_sapien               KHNGVDPEEVVEQYGIDTIRLYILFAAPPEKDILWDTDALPGVLRWQQRL
89306112_Tetrahymena_thermoph      KNNGVNPLEEIEKFGADCLRMTLLFYGPNEKDIAWDGNLQKTLYSYLNKV
15791045_Halobacterium_sp.         KDNGVAPERIVDEYGADTARLFTLRAARPSKAFPWSEEGVRSSHTFLERL
11595633_Neurospora_crassa         KHNGVDPTTFIAQYGADATRAHILFQAPVSEILDWDESKITGVTRWLSRV
53715865_Bacteroides_fragil        MFNVVNPDMIVEKYGADTLRMYEMFLGPVEQSKPWDTNGIDGVHRFIKKF
15826893_Mycobacterium_leprae      LKNSVSPDEICDEYGADTLRVYEMSMGPLEASRPWATKDVVGAYRFLQRV
106884629_Clostridium_phytofer     FPEVVNPSDIIRDYGADTLRLYEMFMGPLEVSKPWNQQGVEGARKFINRV
76788931_Chlamydia_trachoma        KLNGVDPQILIDEFGADAVRMYAMFSGPLDKNKLWSNQGVAGCRRFLNRF
33235997_Chlamydophila_pneumo      KLNGVDPQVLIEEYGADALRMYAMFSGPLDKNKTWSNEGVGGCRRFLNRF
46446681_Parachlamydia_sp.         KLNGESPDEIIQEYGADALRLYEMFMGPLEKEKVWNTDAVSGTRRFLNRF
PLO00010890_Pavlova_lutheri        RGNVVNPDDIVGEYGADAMRMYEMFMGPLDAVKPWQTSQISGVVRFRDRV
21674468_Chlorobium_tepidu         RGNVIPADHVLSTYGADALRLYEMFLGPLDQVKPWNTHGIEGISRFLNKV
42527840_Treponema_dentic          LKNVINPDDIIKEYGADTLRLYEMFLGPLEVSKPWNTSGIMGVFRFLEKI
CM3072_Cyanidioschyzon_merolae     RGNVVNPDDIVEQYGADALRCYLMFMGPLDQVKPWSTQGVNGMRRFLDRA
87311335_Blastopirellula_marin     RGNVVNPDIVVKEYGADSLRLYEMFMGPLEATKPWSMAGVSGVRNFLDRV
83816700_Salinibacter_ruber        RGNVINPDDVVDEYGADTLRLYEMFMGPLEQDKPWSTDDMEGVHRFLNRI
150385918_Victivallis_vadensis     LRNVVNPDDVIREYGADSMRMYEMFMGPLEATKPWSTKGVEGVFRFLKRA
10175904_Bacillus_halodu           KGNVVNPDDIIDSHGADTLRLYEMFMGPLDASIAWSTTGLDGARRFLDRV
116058126_Ostreococcus_tauri       RGNVVNPDDIVSAYGADSLRLYEMFMGPLRETKVWQTKGVEGCFRFLART
7267192_Arabidopsis_thalia         RGNVVNPDDVVLEYGADSLRLYEMFMGPFRDSKTWNTSGIEGVHRFLART
2314727_Helicobacter_pylori        KGNVVSPKEILKKYGADAARLFILFAAPPAKELEWNDSALEGAHRFIKRL
6459971_Deinococcus_radiod         RGNVVDPDDLVREYGADTVRAFLMFIAPWELGGPWDPQGINGPSKWLSRV
83367833_Rhodobacter_sphaeroi      KKNVVDPMNIISQFGADTARWFVMSDSPPERDVEWTASGAEAAFKHLGRV
48766678_Rhodospirillum_rubrum     KMNTVDPTAILESYGADAARLFMLSDSPPDRDMDWTDSGIEGAWRYIGRL
15889997_Agrobacterium_tumefa      KKNVVDPDDIIASYGADTARFFVLSDSPPDRDVIWSEAGVEGANRFVQRV
19712730_Fusobacterium_nuclea      KNNGVDPEEMLDKYGADTTRLFIMFAAPPEKELEWNENGLAGAYRFLTRV
106888735_Roseiflexus_sp           AGNGIPVGPFVRQYGSDVARIVVLFAAPPENSMEWTDEGVAGAQRFLNRI
46130187_Synechococcus_elonga      KYNGVDPARVLDRYGADTARMFILFKAPPEKDLEWDDADVEGQFRFLNRV
53763953_Anabaena_variab           KGNGVAPEDVIAKYGVDTARMFILFKAPPEKDLEWDEADVEGQFRFLNRV
23129999_Nostoc_puncti             KGNGVAPEDVISKYGIDTARMFILFKAPPEKDLEWDEADVEGQFRFLNRV
16128625_Escherichia_coli          KNNGIDPQVMVERYGADTVRLFMMFASPADMTLEWQESGVEGANRFLKRV
30249126_Nitrosomonas_europa       KNNGIDPQEIIEQYGADTARLFMMFASPPTQTLEWSDAGVEGAFRFLKRL
53762629_Ralstonia_eutrop          KNNGIDPQALIDQHGADTARLFVMFAAPPEQQLEWSGSGVEGASRFLRRV
126358263_Pseudomonas_putida       KNNGVDPQSMIEAYGADTCRLFMMFASPPDMSLEWSDSGVEGASRFLRRV
7227154_Neisseria_mening           KNNGVDPQELINAYGADTARLFMMFAAPPEQSLEWSDSGVEGAHRFLRRL
15642942_Thermotoga_mariti         KGNVVSPDEMIEKYGADTLRMYILFMAPPEKDAEWSDAGIEGVHRFVKRL
48846878_Geobacter_metall          KGNVVDPDALINRYGADTARLFSLFAAPPEKDLDWSDQGVDGSYRFLSRV
                                     :          .* *  :   :  .       *             :

60463472_Dictyostelium_discoi      ILFETHLTMNKVTESIDFNTGIAALMELSNTLLRALTLMLFPFSPIFSQI
19683964_Homo_sapien               LWEYKNSVISQVTTHFTLNSAISQLMGLSNALLCALMVMAAPLAPHVTSE
89306112_Tetrahymena_thermoph      TFKKVYGYVENIQNALEFHVAIARQMELYNFLYEYLINSLYPFAPHTMSE
15791045_Halobacterium_sp.         VARETAATVQAATTHFDFNRAVQAVDELVSLLVTAAVKLLAPIAPHVAEE
11595633_Neurospora_crassa         IWREVQRTISSVTASYNLNTVISDLMSLTNVIVSALIRMMAPVAPAFAEE
53715865_Bacteroides_fragil        ELKALHKLIKKVTGDIEYNTSVSAFMICVNELLEQLIVVLAPFAPHVCEE
15826893_Mycobacterium_leprae      TLRTLHRTIAGVSQDYANNTATAKLIEYTNHLVEPLVLMLAPLAPHLAEE
106884629_Clostridium_phytofer     LTKVYHQTVKKVTSDFEFNTAISQMMIFVNSVAEGFIKMYSCICPHVGEE
76788931_Chlamydia_trachoma        GLSLAHKLVQRVTDAIELNTIPSSFMEFINDFVEMAVRALAPIAPHISEE
33235997_Chlamydophila_pneumo      GLVLAHKLVFRITEHIELNTIPSSFMEFLNDFLSMAVRVLEPIAPHISEE
46446681_Parachlamydia_sp.         GLKLGHRLVHSVMKDMEFNTAIAKMMEFINEFIQMATQVLAPFAPHLAEE
PLO00010890_Pavlova_lutheri        TERLMHLTIKKVTQNVDFNTAISAMMVFSNHLAEVLAKLVSPFAPHLGEE
21674468_Chlorobium_tepidu         ILKRMHKAIKKVTEDTEFNTAISEMMVLVNELTETLLVLLSPFAPHITEE
42527840_Treponema_dentic          LTVLLNKTIKKVTEDTAFNTAISQMMIFINEVWYNFVKLLNPYAPHLAEE
CM3072_Cyanidioschyzon_merolae     QLRQLHSLIKRVTEDTEFNTAIAAMIEFVNDALGPFVLLLSPYAPHVAEE
87311335_Blastopirellula_marin     QNKVIHRTIEAVTRDLSFNTPIARMMEFTNFFMKSLVLMLSAYAPHIAEE
83816700_Salinibacter_ruber        QLRTLHRTIKTVTEDIEFNTAIAAMMEFVNAAATPFVLLLSPFAPHLAEE
150385918_Victivallis_vadensis     QLRVLHATIRKVSQDLDFNTAISQLMIFLNEFAEKFVLLLSPFAPHIGEE
10175904_Bacillus_halodu           LKRAYHQTVKKVTEDFEFNVGISQLMVYVNEAAEGFVKLLSPVAPHLAEE
116058126_Ostreococcus_tauri       GLKALNKCIAKVTEETEFNTAISAMMELTNACLEPFSLLLSPYAPHLSEE
7267192_Arabidopsis_thalia         QLRTLHKCIAKVTEEIEFNTGISGMMEFVNAAIEPFVLLLSPYAPHMAEE
2314727_Helicobacter_pylori        GRKKVYEALKKSHEIFNFNTLIASCMEALNALYFVLLQILEPIIPHTAWE
6459971_Deinococcus_radiod         VRFAVHSALKKVNDDFEFNTIISTLMELTNALLDIFNRMLAPVVPHIAEE
83367833_Rhodobacter_sphaeroi      LDRATAKAIAEVTQGIEFNKAIAKLYEFTNTLMRTMAQLMSPMVPHLAEE
```

```
48766678_Rhodospirillum_rubrum      LVRHVHKAVAGVTDDLEFNAAVARLRELTNAILETAARLAAPMIPHIAEE
15889997_Agrobacterium_tumefa       ASKAAHKTLKAVQEDLDFNKAIARIYELVNALVEILIRIIAPMTPHLAEE
19712730_Fusobacterium_nuclea       LLIKLNQTIKKVTDAIEFNTAIAANMELINEVLKKIIIMLSPFVPHFCDE
106888735_Roseiflexus_sp            LYRKLHETIRKVTLDTEFNTAIAALMELLNEAAATFARLLSPFAPHLAEE
46130187_Synechococcus_elonga       LRRAVHTAIQAVTEDLELNTAIAELMKLTNALVQTLVLLLAPFAPHIAEE
53763953_Anabaena_variab            LRRAIHSAIQSVTEDLEFNTAISELMKLSNALIHTLVVLLAPFAPHIAEE
23129999_Nostoc_puncti              LRRAIHTAIQAVTEDVEFNTAISELMKLSNALIRTLVILIAPFAPHIADE
16128625_Escherichia_coli           LRRDVHKTIAKVTDDIGFNTAIAAIMELMNKLLLAVVRMLNPFTPHICFT
30249126_Nitrosomonas_europa        LRCQLHQTIVKVTDDLEFNTAIAAIMELMNELLENIVLLLSPIVPHICHV
53762629_Ralstonia_eutrop           LRREIHGVLKQANYDYQYNTVVSATMKMLNALFGILLRVLYPVVPHITHG
126358263_Pseudomonas_putida        IRRAIHAAIKQASTDVGFNTAIAQVMTVMNVLLEAVTLLLAPITPHISHE
7227154_Neisseria_mening            LRHKLHATTAKVSDDYGFNTAIAAVMELLNQYLETAVRLLWPIVPHICET
15642942_Thermotoga_mariti          LRRKLHSIIKKITEDIEFNTAISGLMELVNHLVEKLTLALSPFAPHLAEE
48846878_Geobacter_metall           LRRAVHKTIRKVTDDIDFNTAIAAIMELVNAIIESVVIMLSPFVPHVTEE
                                                      :          .              *

60463472_Dictyostelium_discoi       HWKSLVFNQRWPKPTPSALVRDFNSLVIQ
19683964_Homo_sapien                IWAGLVLLQAWPAVDPEFLQQEVVQMAVL
89306112_Tetrahymena_thermoph       IAENLEYQFVWPDL-----------LAIK
15791045_Halobacterium_sp.          CWTALVAEAAWPTPDRDVSDHDRATSLIE
11595633_Neurospora_crassa          CWHVLAARFPVPDGTEGLLKSRKQTCAVQ
53715865_Bacteroides_fragil         LWDTLVCDAQWPTFNEQYLVEDTVNYTIS
15826893_Mycobacterium_leprae       LWLRLLANGPFPQADPAYLVDDTVEYPVQ
106884629_Clostridium_phytofer      MWNVLLANESWPTYDEDAIKEDTITIGVQ
76788931_Chlamydia_trachoma         LWVLLVQKSGWPSVLPEYLEGQTVTIVVQ
33235997_Chlamydophila_pneumo       LWVILIDQAAWPQIDESYLVAQTVTFVVQ
46446681_Parachlamydia_sp.          VWEHLLSFTPYPQVEEKYLQENVITYVVQ
PLO00010890_Pavlova_lutheri         MWQRLLAYEPWPQYDEA------------
21674468_Chlorobium_tepidu          LWQALISGAVWPVFDAKLATDDVLTIAVQ
42527840_Treponema_dentic           LWQKMIAYSHWPMFVEKFCVDQTCTVVVQ
CM3072_Cyanidioschyzon_merolae      MWQEILAYEPWPDYDPQYLVQETVVYAVQ
87311335_Blastopirellula_marin      LWAVLLAYHPWPQFDEKFTKDDEIEIPVQ
83816700_Salinibacter_ruber         LWARLLAYADWPAYDDELIRREVVEMPVQ
150385918_Victivallis_vadensis      LWSTLLAYEPWPVWDEKVLEVNEVEILVQ
10175904_Bacillus_halodu            LWSKLIAYEPWPTYDEAFLVEDEVEIVVQ
116058126_Ostreococcus_tauri        MWERLNSKTSWPVADDSFLVEDTVDIGVQ
7267192_Arabidopsis_thalia          LWSRLLAYESFPKANPDYLKNTTIVLPVQ
2314727_Helicobacter_pylori         LSERLRENFKPIAIDEDALMEDFMTLGLT
6459971_Deinococcus_radiod          IWHERVHTAQWPQVDEAAAVRDTVTIGVQ
83367833_Rhodobacter_sphaeroi       VWSMLVAQAPWPKADPALLVDDMVTLPIQ
48766678_Rhodospirillum_rubrum      MWSHLLAETPWPTWDPLMLIDDQVTIAVQ
15889997_Agrobacterium_tumefa       CWSALVAETPWPTFVASLVEENDVVMPVQ
19712730_Fusobacterium_nuclea       IWEELLFNEKWPEYDEKMLSSDETTIAVQ
106888735_Roseiflexus_sp            VHSWCVYDAGWPEWDESALVLDEVEIVLQ
46130187_Synechococcus_elonga       LWQQLVHLEGWPVLDESALIVDEIPLVIQ
53763953_Anabaena_variab            LWQLLVHTQTWPAFDPAALVADEITLVIQ
23129999_Nostoc_puncti              LWHLLIHTQTWPSFDPAALVADEITLVIQ
16128625_Escherichia_coli           LWQELIDNAPWPVADEKAMVEDSTLVVVQ
30249126_Nitrosomonas_europa        LWRELLLDQPWPQADDQALIQDEVEIVVQ
53762629_Ralstonia_eutrop           LWDALLLDAPWPQVDEGALVRTEIEMVLQ
126358263_Pseudomonas_putida        LWQQLVIDAAWPSVDEQALVQDTITLVVQ
7227154_Neisseria_mening            LWSELLWEAGWPTVDEAALVKSEIEVMVQ
15642942_Thermotoga_mariti          FWHDLVVQQSWPSYDPKALEVEEVEIAIQ
48846878_Geobacter_metall           LWEALVEAAGWPSFDPSAAVDEEFLIVVQ
```

Note: All top hits in GenBank searches (using the *Cyanidioschyzon* sequence and *Arabidopsis* NP_192344 as queries) are from firmicutes, CFB bacteria and lentisphaerae. These sequences also differ from others in sharing several unique residues and a 1-bp insertion. Sequences of *Neurospora* and *Homo* are mitochondrial precursors according to GenBank annotations. In *Arabidopsis* (GenBank accession number NP_192344 and TAIR locus AT4G04350), the protein product is targeted to both chloroplasts and mitochondria. The plant sequences are distantly related to other eukaryotic homologs based on both sequence similarity and phylogenetic analyses. Sequence of *Pavlova* was obtained from TBestDB.

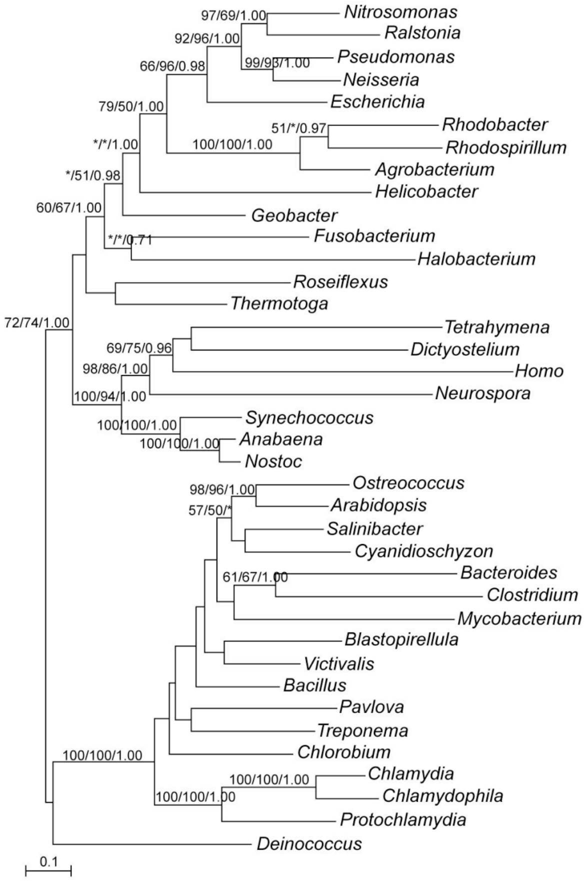

Figure 17. Molecular phylogeny of leucyl-tRNA synthetase. P-value = 0.235 from AU test for the presented tree. AU tests were also performed on alternative topologies including (**A**) monophyly of all eukaryotic sequences, and (**B**) monophyly of cyanobacterial and photosynthetic eukaryotic sequences. Topology **A** investigates if all eukaryotic sequences have the same origin (e.g. mitochondrial) whereas topology **B** investigates if photosynthetic eukaryotes acquired this gene from plastids. P-values < 0.001 from AU tests for both alternative topologies.

---

18. Ribosomal protein L11 methyltransferase (D)

CLUSTAL X (1.83.1) multiple sequence alignment

```
24214091_Leptospira_interr         EYEEAYKEFYKPFIISYRVIPTWFPLLVNPGLAFGTGHHETTRLVLGRMG
91206171_Rickettsia_bellii         DWVALYQNQLVPIQTRFFICTTLTLILIEASRAFGTGTHETTSGCIEALE
67940209_Chlorobium_phaeob         NWNAEWEANLKPVEIRIMIVQQNILIEINPKMSFGTGYHATTRLMLRQIE
34540441_Porphyromonas_gingiv      NWNEQWEKNFFPIRIKCLVRAPFLELIISPQMAFGTGHHETTSLMMSYLL
108866182_Aedes_aegypti            NWNEEWEKNFQPINVKVLIRAEFEEIIIQPKMSFGTGHHATTYLMIQQMM
RAO00324465_Reclinomonas_ameri     NWNALWESSFEPVIVFAAVRAHFQEIIITPRMSFGTGHHATTWMMIRSME
78777868_Thiomicrospira_denitr     DWVKVYQDSIEPLRIKFYIHPTWINIAIDPALAFGTGHHPTTASALRAIA
33240884_Prochlorococcus_marin     DWSSSWKKFWKADPVKILILPSWIVIKLDPGSAFGTGSHPTTRLCLEDLE
77464128_Rhodobacter_sphaer        DWVAKVRRELSPVEARFFVYGSHIALQIEATVAFGTGHHGTTLGCLRALD
```

```
113875614_Sinorhizobium_medica      DWIAKSLEGLAPVRARFVIHGSHIAIEIDAGQAFGTGHHGTTAGCLEMLA
34762532_Fusobacterium_nuclea       DYQNSWKKYLFVEKVKFVVKPTWLVIELDPGRAFGTGSHPTTSLLLKLME
37521705_Gloeobacter_violac         DWQAAWREQWRPIFVRLVIWPVWLVIPLDPGMAFGTGEHATTRLCLRALE
38605120_Thermotoga_neapo           TTPKDWMVELEPFEIDVVVDPTEIVVKLSPGVAFGTGLHPTTQMSVLFLK
116057515_Ostreococcus_tauri        DWVEAVKESFSPIEVGLNIVPNWVNIMLEPGIAFGTGEHPTTRLCLRWLK
15238878_Arabidopsis_thalia         DWITKNQDLFQPVEIRLWIVPEWVNIILNPGFAFGTGEHPTTKLCLLLLQ
CM3418_Cyanidioschyzon_merolae      DWVRAVRESFQPLLIRLLVRFPWVQLTIEPGAAFGTGEHPTTQLCARWLE
33151297_Haemophilus_ducrey         DWEREWMDNFHPMQFRLWICPSWVNIMLDPGLAFGTGTHPTTALCLQWLD
75828283_Vibrio_choler              DWEREWMDNFHPMQFRLWICPSWVNVMLDPGLAFGTGTHPTTALCLEWLD
15677697_Neisseria_mening           DWVRLTQSQFDPIRIRLWITPSWVNLRLDPGLAFGTGSHPTTRLCLKWLD
67546225_Burkholderia_vietna        DWVRLTQSQFEPIPIRIWVVPSWLILELDPGLAFGTGSHPTTRLCMEWLE
83746797_Ralstonia_solana           DWVRLTQSQFEPIHIHIWVVPSWVVLELDPGLAFGTGSHPTTRLCMEWLE
15607057_Aquifex_aeolic             DWEN-WKEKFKPIEVDFVIIPSWKPVIVKPGLAFGTGLHPTTQLCIKALK
46198605_Thermus_thermo             DWLEAWRRDLKPA-LPFVVLAPWIPLVIEPGMAFGTGHHETTRLALKALA
94984946_Deinococcus_geothe         DWLAEFKRTLRPVRARVTIVPPWLPLVIEPGMAFGTGHHATTRLAVEALS
15924568_Staphylococcus_aureus      DWENEWKNYFHPFRAKFTIVPSWLCIELDPGMAFGTGDHPTTSMCLKAIE
67873260_Clostridium_thermo         DWSNSWKKYYKPLHLRLIVKPSWIIIEMNPGMAFGTGTHETTKMCAVLLD
30022391_Bacillus_cereus            DWATAWKKYYHPVQITFTIVPTWKIIELDPGMAFGTGTHPTTTMCIRALE
76258363_Chloroflexus_aurant        DWANAWKQFYPVLRVRTVIVPSWVVLLLDPGMAFGTGLHPTTRLCLHLLE
88936160_Geobacter_uraniu           DWANNWKEYFKPVRIRLVIKPTWIVLKLDPGMAFGTGAHPTTKMCLEVLE
                                                      :      : : .  :**** * **      :

24214091_Leptospira_interr          DLNIADVGTGSGILSLAAAKSGLAIDVDPNSVRSASFNRDENEILLIANI
91206171_Rickettsia_bellii          YLKILDIGTGSGILSFIAEKLWLACDIDNASVEIAKENASFNNSLVISNI
67940209_Chlorobium_phaeob          NIDILDIGTGTGVLAIACRKIGLAIDNDDWAVENARENVSVNNVLILANI
34540441_Porphyromonas_gingiv       DMDVLDMGCGTGILAILARKLGTAIDIDDWCIRNTGENAALNDILIIANI
108866182_Aedes_aegypti             DMDVLDMGCGTSVLAIYAKKKGLGIDIDEWAVENSRENAERNNTIILANI
RAO00324465_Reclinomonas_ameri      ELDVLDFGTGTGVLAILAEKLGTAIDIDEWSIR-----------------
78777868_Thiomicrospira_denitr      KYVVLDVGCGSGILGVGAIKLGDACDTDIACIENSQLNSELNNVVVVANI
33240884_Prochlorococcus_marin      RNPVVDIGCGSGVLGIAAIKLGRAIDIDSLAVRATSENIVLNNLLICNT
77464128_Rhodobacter_sphaer         RLVVADVGCGTAVLAMAAARVFLASDIDEVAVEVAEANVAINGLLVFANI
113875614_Sinorhizobium_medica      SVAALDLGTGSGVLAIAAWKLLLATDIDPVATFVASENARRNGVLIIANI
34762532_Fusobacterium_nuclea       EQDVIDIGTGSGILMIAGKLLGYGTDIDEFSMEVAKENLILNNIIVVCNI
37521705_Gloeobacter_violac         SVPFADVGCGSGVLTVAALKLGWAVDTDDLAVVSTRKNLEISGLGVVSNI
38605120_Thermotoga_neapo           KYLVVDVGCGTGILAIVAKKLGLAVDVDEQAVEVAKENVQKNSVLVVSNI
116057515_Ostreococcus_tauri        KTIVVDFGCGSGVLAIGALLLGVGVDLARQAVQSSMDNAKLNGVVVIANI
15238878_Arabidopsis_thalia         SLIFLDYGTGSGILAIAALKFGVGVDIDPLAINSAIHNAALNNIVVIANI
CM3418_Cyanidioschyzon_merolae      LHVVLDYGSGSGILVMYACKLGVGIDIDEEAIAVAKRDAVRNQLLLVSNI
33151297_Haemophilus_ducrey         GLDVIDFGCGSGILAISALKLGIGIDIDPQAILASQNNAAANGVVVVANI
75828283_Vibrio_choler              NLDVIDFGCGSGILAIAAIKLGIGIDIDPQALLASKDNAARNGVVVVANI
15677697_Neisseria_mening           TQLVLDYGCGSGILTIAALKLGVGVDIDEQAVRAGKDNAAQNNVVVVANI
67546225_Burkholderia_vietna        QTVVLDYGCGSGILAILARKCGIGIDIDPQAVESARQNSERNHAIVVANI
83746797_Ralstonia_solana           QHVALDYGCGSGILAIVAKKLGLGIDIDPNAVEASRYNAERNRVLVVANI
15607057_Aquifex_aeolic             KYLVLDVGTGSGILAIVSALLGVGIDIDEKAVNECRENAELNKVLVVANL
46198605_Thermus_thermo             RHLVLDLGTGSGVLAIAAEKLGLGVDIDPMVLPQAEANAKRNGVLLVANL
94984946_Deinococcus_geothe         ALNVLDVGTGSGVLAIAAALLGLGVDIDPITIPIARENAQINGIVVVANL
15924568_Staphylococcus_aureus      TYVVIDVGTGSGILSIASHLIGKALDIDEMAVSVAKENFRRNHCIVIANI
67873260_Clostridium_thermo         KYVVIDVGCGTGILSIIASKLGTAVDIDEVAVKVAKENLELNKVIVVANI
30022391_Bacillus_cereus            KTVIIDVGTGSGVLSIAAAKLGQAYDLDPVAVESAEMNVRLNKTLIVANL
76258363_Chloroflexus_aurant        RLVVLDLGTGSGILAIAAAKLGLALDNDPIAVRVAHENVERNQVLIAANL
88936160_Geobacter_uraniu           MIFVLDVGTGSGVLSIAAAKLGTAIDIDADAVSVAEENLALNDAIVLANI
                                         * * *:.:* .       . *

24214091_Leptospira_interr          TFAVLKANIQKIASI--KTDHFLFSGVITERKEEFLELL
91206171_Rickettsia_bellii          LALPLIELSTQISNLMNKNGYLVLSGFLDNQLEDVRNAY
67940209_Chlorobium_phaeob          NRNVLDRILPSIKEK-APTSRVLLSGIMIYDEPWLKKLL
34540441_Porphyromonas_gingiv       NRNILLDDMSAYRSRLGNGGTLLLSGFYTEDIPILTECA
108866182_Aedes_aegypti             NRNILISDIPRYVEVLEPGGSLLLSGLCFFDVDDILQVC
RAO00324465_Reclinomonas_ameri      ---------------------------------------
78777868_Thiomicrospira_denitr      VADVLIFIANDLKSVVKEDGVLVLSGILEKYESKVLKFY
33240884_Prochlorococcus_marin      LSPVIKELAPYFFKLTHSYSRLCLSGLLVAQVEDITNFL
77464128_Rhodobacter_sphaer         LKGPLIELAPAMAAHVAPGGGLILSGLLVVQADSITEAY
113875614_Sinorhizobium_medica      LARPLMKMAPQLAANLAPGGSVILSGILAEQRWKVIAAY
34762532_Fusobacterium_nuclea       LADVLVKLLDEIKYILKENSIVLFSGIIEDKLNEVISKA
37521705_Gloeobacter_violac         LAEVIANLAPEFRRLVHPGGWGIFSGLLLTQAPRVVEAL
38605120_Thermotoga_neapo           LAEIHLRLLEDVSRVTHEKSILILSGIVDTKEDMVREKA
116057515_Ostreococcus_tauri        LIQPVLELEPLFARYCKTGGEVALSGVLHEQSASVVAKY
15238878_Arabidopsis_thalia         LLNPVMNLADHILSFVKPGATIGISGILSEQLPNVRERY
CM3418_Cyanidioschyzon_merolae      LAPTLQELAPRFCSLVRTGGRIALSGMLAAHTAAVWASY
33151297_Haemophilus_ducrey         LAGPLKELAPNIMTLVKPQGYLGLSGILATQAQSVCQAY
75828283_Vibrio_choler              LAGPLRELSPTIKGLLKPGGQLAMSGILDTQAESVAEFY
15677697_Neisseria_mening           LANPLRMLGEMLAARTKQGGRIVLSGLLDEQAEELGGIY
67546225_Burkholderia_vietna        LSNPLKLMASMLASKVKPGGRIALSGVLARQADEVAAVY
```

```
83746797_Ralstonia_solana         LSNPLKLMAAMLCARVRPGGRLVLSGVLERQAEEVAAAY
15607057_Aquifex_aeolic           EIHIFERVLKDILPKFKKIG--IFSGLYKEKDLKRFEEL
46198605_Thermus_thermo           YAELHAALALRYREALVPGGRALLTGILKDRAPLVREAM
94984946_Deinococcus_geothe       YAELHDLLAGEYAAHLVPGGALILTGILTVKLPLVHAAL
15924568_Staphylococcus_aureus    LAHIIDEMIEDAYNTLNEGGYFITSGIIKEKYEGIQSHM
67873260_Clostridium_thermo       IANVIMDISSRVPYYLKKDGLFIASGIIKERKQEVLDEC
30022391_Bacillus_cereus          LAEIILLFPEDAARVVKSGGLFITSGIIAAKEKVISEAL
76258363_Chloroflexus_aurant      IAKVLVLLAPDLATALKPGGLLISSGIIDVKEAEVVAAF
88936160_Geobacter_uraniu         LAEELVRLAPELVDRTAPGGYLVLSGILTEKEEFVINGF
```

Note: The identifiable homologs of this gene (using *Cyanidioschyzon* and *Arabidopsis* sequences as queries) are found only in bacteria and eukaryotes, with all top hits being from beta and gamma-proteobacteria. This disjunct distribution suggests that the eukaryotic sequences are likely of bacterial origin. Phylogenetic analyses show a common ancestry of sequences from red algae, green plants and gamma, beta-proteobacteria. The gene was likely transferred independently to two groups of eukaryotes, one to the ancestor of red algae and green plants, another to the bacteriotrophic *Reclinomonas*. *Reclinomonas* and *Aedes* share several conserved residues and their grouping together likely resulted from eukaryote-to-eukaryote gene transfer. The *Arabidopsis* gene product is localized in cytoplasm according to GenBank annotation. Sequence of *Reclinomonas* was obtained from TBestDB.

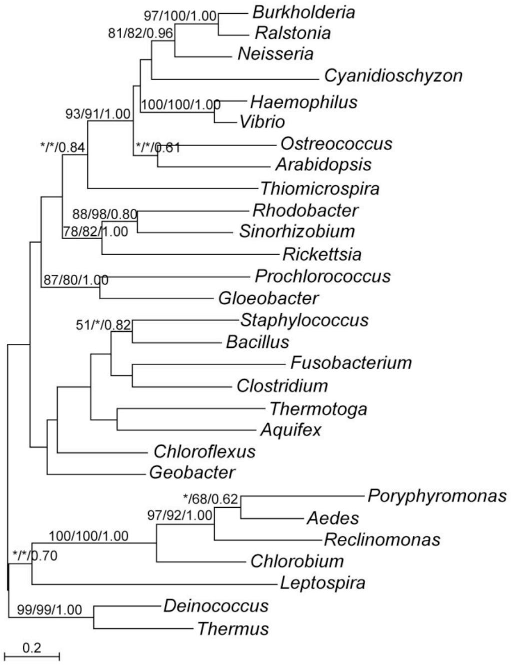

Figure 18. Molecular phylogeny of ribosomal protein L11 methyltransferase. P-value = 0.959 from AU test for the presented gene tree. AU tests were also performed on alternative topologies including (**A**) monophyly of all eukaryotic sequences, and (**B**) monophyly of red algal, green plant, and cyanobacterial sequences. P-value < 0.001 from AU tests for topology **A** and p-value = 0.041 for topology **B**.

---

19. GTP binding protein typA (D)

CLUSTAL X (1.83.1) multiple sequence alignment

```
34762648_Fusobacterium_nuclea        IKNIAIIAHVDHGKTTLVDCLLRQGGVFKTHEERVMDSDDIERERGITIF
28210762_Clostridium_tetani          IRNIAIIAHVDHGKTTLVDAMLKESHVFRENEERVMDSNDLERERGITIL
6458935_Deinococcus_radiod           YRNIAIIAHVDHGKTTLVDGLLKQTLELKHGEERAMDSNDLERERGITIL
51892172_Symbiobacterium_therm       LRNVAIIAHVDHGKTTLVDGLLRQSGTFRANEERVMDSFDLERERGITIL
67920868_Crocosphaera_watsonii       IRNVAIIAHVDHGKTTLVDSLLKQSGIFREGEDCVMDSNTLERERGITIL
16329356_Synechocystis_sp.           IRNVAIIAHVDHGKTTLVDALLKQSGIFREGEVCVMDSNDLERERGITIL
33861319_Prochlorococcus_marin       IRNVAIIAHVDHGKTTLVDALLSQSGIFRDNETCVMDSNDLERERGITIL
69247248_Enterococcus_faecium        IRNVAIIAHVDHGKTTLVDELLKQSDTLDAHTERAMDSNALEKERGITIL
16078541_Bacillus_subtil             LRNIAIIAHVDHGKTTLVDQLLHQAGTFRANEERAMDSNDLERERGITIL
21674245_Chlorobium_tepidu           IRNIAIIAHVDHGKTTLVDSIFKQTGAFRENQVRVMDSNPQERERGITIF
57169035_Campylobacter_coli          IRNIAVIAHVDHGKTTMVDELLKQSGTFSEREERVMDSNDIEKERGITIL
15611499_Helicobacter_pylori         IRNIAVIAHVDHGKTTLVDGLLSQSGTFSEREERVMDSNDLEKERGITIL
45658203_Leptospira_interr           IRNIAIIAHVDHGKTTLLDGILRQTGAVTAKEERIMDSNDLEKEKGITIK
39995607_Geobacter_sulfur            IRNIAIIAHVDHGKTTLVDAMLKHAGVFRENEERVMDSNDLEKERGITIL
24375888_Shewanella_oneide           LRNIAIIAHVDHGKTTLVDKLLSQSGTLATRGERVMDSNDLEKERGITIL
16272804_Haemophilus_influe          LRNIAIIAHVDHGKTTLVDKLLQQSGTFESARERVMDSNDLEKERGITIL
59801217_Neisseria_gonorr            IRNIAIIAHVDHGKTTLVDQLLRQSGTFRANQERVMDSNDLEKERGITIL
30250473_Nitrosomonas_europa         LRNIAIIAHVDHGKTTMVDKLLHQAGTFAAHQERVMDSDDIERERGITIF
32474280_Rhodopirellula_baltic       IRNVVIIAHVDHGKTTLVDCLLRQSGQYRDAEERILDSNDIEKERGITIL
53715742_Bacteroides_fragil          IRNIAIIAHVDHGKTTLVDKMLLAGNLFRGNQELILDNNDLERERGITIL
48854524_Cytophaga_hutchi            IRNIAIIAHVDHGKTTLVDKIIHASKIFRENQDLILDNNDQERERGITIL
91201932_Candidatus_Kuenenia         VRNVAIIAHVDHGKTTLVDQLLKQSGTFRVNQERIMDSNDLERERGITIL
30684514_Arabidopsis_thalia          VRNIAIVAHVDHGKTTLVDSMLRQAKVFRDNQERIMDSNDLERERGITIL
30684509_Arabidopsis_thalia          VRNIAIVAHVDHGKTTLVDSMLRQAKVFRDNQERIMDSNDLERERGITIL
50906979_Oryza_sativa                VRNIAIVAHVDHGKTTLVDSMLRQAKVFRDNQERIMDSNDLERERGITIL
CM3614_Cyanidioschyzon_merolae       VINIAICAHVDHGKTTLVDAILHHAKVFRENQSLVMDSNELERERGITIL
106888453_Roseiflexus_sp.            IRNIAIIAHVDHGKTTLVDGLLRQGRIFRDNQERVLDSNDLERERGITIM
53795725_Chloroflexus_aurant         LRNIAIIAHVDHGKTTLVDAMLKQSRIFRQNQERVLDSNALERERGITIL
113940020_Herpetosiphon_aurant       LRNIAIIAHVDHGKTTLVDAMLKQSRIFRDNQERVMDSNAIERERGITIM
15608305_Mycobacterium_tuberc        FRNVAIVAHVDHGKTTLVDAMLRQSGALRERGERVMDTGDLEREKGITIL
94971430_Acidobacteria_bacteri       LRNIAIIAHVDHGKTTLVDAMLKQSGTFRANEDRVMDSNELERERGITIL
46446750_Protochlamydia_amoebo       IRNIAIIAHIDHGKTTLLDALLKQSKIFRDNQERVMDSYDQEQERGITIF
89284086_Tetrahymena_thermoph        FRNVAIIAHVDHGKTTLVDTLLKTSG------DKSMDSNQLEQEKESNNR
42733852_Dictyostelium_discoi        IRNVAIIAHVDHGKTSLVDQLLRQSGAIKAT-ERVMDSNALEKERGITIM
34909598_Oryza_sativa                IRNVAVIAHVDHGKTTLMDRLLRQCGAD----ERAMDSISLERERGITIA
79323586_Arabidopsis_thalia          LRNVAVIAHVDHGKTTLMDRLLRQCGAD----ERAMDSINLERERGITIS
15889919_Agrobacterium_tumefa        LRNIAIIAHVDHGKTTLVDELLKQSGSFRDNQERVMDSNDLEKERGITIL
51473460_Rickettsia_typhi            IRNIAIIAHVDHGKTTLVDNMLKQSGTFRANQERAMDSNELERERGITIL
56696900_Silicibacter_pomeroy        LRNIAIIAHVDHGKTTLVDELLKQSGAFRENQERAMDSNDLERERGITIL
23612528_Plasmodium_falcip           IRNVAIIAHVDHGKTTLVDKLLRQGGEITNN-DRIMDHNDLEKERGITIM
                                       *:.: **:*****:::* ::             :*    *:*:  .

34762648_Fusobacterium_nuclea        SKNASVRYKDYKINIVDTPGHADFGGEVQRIMKMVDSVLLLVDAFEGPMP
28210762_Clostridium_tetani          SKNTSVMYDGVKINIIDTPGHADFGGEVERVLKMVDSVLLVVDSYEGPMP
6458935_Deinococcus_radiod           AKNTAVEYNGVKINIVDTPGHADFGGEVERVLGMVDGALVLVDAAEGPMP
51892172_Symbiobacterium_therm       SKNTGITYKGVRINIVDTPGHADFSGEVERIMTMVDGCLLIVDAAEGPMA
67920868_Crocosphaera_watsonii       SKNTAVRYNDTLINIVDTPGHADFGGEVERVLGMVDGCILIVDANEGPMP
16329356_Synechocystis_sp.           SKNTAVRYQDTLINIVDTPGHADFGGEVERVLGMVDGCVLIVDANEGPMP
33861319_Prochlorococcus_marin       SKNTAVNYKNTRINIIDTPGHADFGGEVERVLGMVDGCLLIVDANEGPMP
69247248_Enterococcus_faecium        AKNTAVDYKGIRVNIMDTPGHADFGGEVERIMKMVDGVVLVVDAYEGTMP
16078541_Bacillus_subtil             AKNTAINYKDTRINILDTPGHADFGGEVERIMKMVDGVVLVVDAYEGCMP
21674245_Chlorobium_tepidu           SKNAAVQHKGCKINIVDTPGHADFGGEVERILKMVDGVLLLVDAFEGPMP
57169035_Campylobacter_coli          SKNTAINYKGTKINIIDTPGHADFGGEVERVLKMIDGVLLLVDAQEGVMP
15611499_Helicobacter_pylori         SKNTAIYYKDTKINIIDTPGHADFGGEVERVLKMVDGVLLLVDAQEGVMP
45658203_Leptospira_interr           AKNTAVVYKGTRINVVDTPGHADFGGEVERVLATADSCLLLVDAFDGPMP
39995607_Geobacter_sulfur            AKNLSVHHGRYKINIVDTPGHADFGGEVERVLKMVDSVLLLVDALDGPMP
24375888_Shewanella_oneide           AKNTAIKWNDYRINIVDTPGHADFGGEVERVLSMVDSVLLLVDAVDGPMP
16272804_Haemophilus_influe          AKNTAINWNDYRINIVDTPGHADFGGEVERVLSMVDSVLLVVDAFDGPMP
59801217_Neisseria_gonorr            AKNTAIDYEGCHINIVDTPGHADFGGEVERVLGMVDCVVLLVDAQEGPMP
30250473_Nitrosomonas_europa         SKNCAIDYAGVHINIVDTPGHADFGGEVERVLSMVDGVLLLVDAVEGPMP
32474280_Rhodopirellula_baltic       SKNIAVHYRGVKINLIDTPGHADFGGEVERVVQMADGALVLVDAAEGPMP
53715742_Bacteroides_fragil          SKNVSINYNGTKINIIDTPGHSDFGGEVERVLNMADGCILLVDAFEGPMP
48854524_Cytophaga_hutchi            AKNVSIRYKEHKINIIDTPGHADFGGEVERVLKMADGVVLLVDAFEGAMP
91201932_Candidatus_Kuenenia         AKNTAINYEGVKINIIDTPGHADFGGEVERVLKMADGVLLLVDAAEGVMP
30684514_Arabidopsis_thalia          SKNTSITYKNTKVNIIDTPGHSDFGGEVERVLNMVDGVLLVVDSVEGPMP
30684509_Arabidopsis_thalia          SKNTSITYKNTKVNIIDTPGHSDFGGEVERVLNMVDGVLLVVDSVEGPMP
50906979_Oryza_sativa                SKNTSITYKGTKINIIDTPGHSDFGGEVERVLNMVEGVLLVVDSVEGPMP
CM3614_Cyanidioschyzon_merolae       SKNTAVVYRGVRINVIDTPGHADFGGEVQRVLSMADAMLLVVDSVEGPRP
106888453_Roseiflexus_sp.            AKNTAVTYRGVKINIVDTPGHADFGGEVERVMNMVDGVLLLVDAVDGPMP
53795725_Chloroflexus_aurant         AKNTAVNYRGVKINIVDTPGHADFGGEVERVMNMVDGVLLLVDAVDGPMP
113940020_Herpetosiphon_aurant       AKNTAVEYNGVRINIVDTPGHADFGGEVERVLNMVDGVLLLVDAVEGPMP
15608305_Mycobacterium_tuberc        AKNTAVHRHHPDINVIDTPGHADFGGEVERGLSMVDGVLLLVDASEGPLP
94971430_Acidobacteria_bacteri       AKNTAVFYHDIKINIVDTPGHSDFGGEVERALKMVDGVMLLVDASEGPLP
```

```
46446750_Protochlamydia_amoebo      AKHTSVYFDDFKINIIDTPGHADFSGEVERILGMVDSVLLLVDAQEGPMP
89284086_Tetrahymena_thermoph       SKVTGVTFKDYKINIVDTPGHHDFGGEVERIMSMVDGVILLVCATEGPMT
42733852_Dictyostelium_discoi       AKCTSFDYKNHKLNIVDTPGHGDFGGEVERVLSMVDGVVLLVDATEGPMA
34909598_Oryza_sativa               SKVTSVSWKENELNMVDTPGHADFGGEVERVVGMVEGAVLVVDAGEGPLA
79323586_Arabidopsis_thalia         SKVTSIFWKDNELNMVDTPGHADFGGEVERVVGMVEGAILVVDAGEGPLA
15889919_Agrobacterium_tumefa       AKATSVEWKGVRINIVDTPGHADFGGEVERILSMVDGAIVLVDSSEGPMP
51473460_Rickettsia_typhi           AKCTALMWNEIRINIVDTPGHADFGGEVERILSMVDGVVLLVDASEGPMP
56696900_Silicibacter_pomeroy       AKATSVEWRGTRVNIVDTPGHADFGGEVERILSMVDGVVLLVDAAEGPMP
23612528_Plasmodium_falcip          SKVTRIKYDDYYFNIVDTPGHSDFGGEVERVLNLIDGVCLIIDVVEGPKN
                                    :*   .      .*::***** **.***:* :   :   :::   :*

34762648_Fusobacterium_nuclea       QTKYVLKKALEQGHRPIVVVNKVDKPNARPEDVLYMVYDLFIELNANEYQ
28210762_Clostridium_tetani         QTKFVLKKALELDLKPIVVINKIDRPDARAEDVLDEIFDLFVELGASDDQ
6458935_Deinococcus_radiod          QTRFVLKKAIELGLKPIVVVNKIDRNDARPEEVVNLTFDLMAELGANDDQ
51892172_Symbiobacterium_therm      QTKFVLRKAMEAGLKPVVVINKIDRPDRRIAEVEDEVLDLFIELGADEDQ
67920868_Crocosphaera_watsonii      QTRFVLKKALEKGLRPIVVVNKIDRPQAEPDQAVDKVFDLFIELGADDDQ
16329356_Synechocystis_sp.          QTRFVLKKALEKGLRPLVVVNKIDRPRADPNTAVDKVFDLFVELGADDDQ
33861319_Prochlorococcus_marin      QTRFVLKKALEKGLRPIVFVNKIDRPRVVPEIAVDKVLDLFLELGADDDQ
69247248_Enterococcus_faecium       QTRFVLKKALEQHITPIVVVNKIDKPSARPEHVVDEVLELFIELGADDDQ
16078541_Bacillus_subtil            QTRFVLKKALEQNLNPVVVVNKIDRDFARPEEVIDEVLDLFIELDANEEQ
21674245_Chlorobium_tepidu          QTKFVLRKALELHLKPIVVINKIDRPQADPEKVHDQVLDLFIALGADEDQ
57169035_Campylobacter_coli         QTKFVVKKALSLGLKPIVVINKIDKPAADPERVINEIFDLFVALDANDEQ
15611499_Helicobacter_pylori        QTKFVVKKALSFGICPIVVVNKIDKPAAEPDRVVDEVFDLFVAMGASDKQ
45658203_Leptospira_interr          QTRFVLGKSLQLGHRPILVINKIDRPGARPEAVVDMAFDLFSDLGATDEQ
39995607_Geobacter_sulfur           QTRFVLKKSLDLGLKPIVVINKIDRPGSRPDEVVDMVFDLFCELNATDEQ
24375888_Shewanella_oneide          QTRFVTKKAFAQGLKPIVVINKIDRPGARPDWVIDQVFDLFDNLGATDEQ
16272804_Haemophilus_influe         QTRFVTQKAFAHGLKPIVVINKVDRPGARPDWVVDQVFDLFVNLGASDEQ
59801217_Neisseria_gonorr           QTRFVTKKALALGLKPIVVINKIDKPSARPSWVIDQTFELFDNLGATDEQ
30250473_Nitrosomonas_europa        QTRFVTRKALASGLRPIVVVNKIDRPGARPDWVVNQTFDLFDKLNATEEQ
32474280_Rhodopirellula_baltic      QTRFVLEKALQAGVKPIVVVNKVDRPDGRPPEALDEALELLADLGGEEQL
53715742_Bacteroides_fragil         QTRFVLQKALEIGLKPIVVINKVDKPNCRPDEVHEMVFDLMFSLDATEEQ
48854524_Cytophaga_hutchi           QTRFVLSKALGLGLKPILIVNKVDKENCRPDEVHEEVFDLMFNLGANEHQ
91201932_Candidatus_Kuenenia        QTRFVLRKALGYNLNPLVVINKIDRPDARCQDVINEVFDLFVDLDANDEQ
30684514_Arabidopsis_thalia         QTRFVLKKALEFGHAVVVVVNKIDRPSARPEFVVNSTFELFIELNATDEQ
30684509_Arabidopsis_thalia         QTRFVLKKALEFGHAVVVVVNKIDRPSARPEFVVNSTFELFIELNATDEQ
50906979_Oryza_sativa               QTRFVLKKALEFGHAVVVVVNKIDRPTARPEFVVNSTFELFIELNATDEQ
CM3614_Cyanidioschyzon_merolae      QTRYVLREALRLGQRVLVVVNKIDRPAARPVFVVNKTFDLFVELGASDEQ
106888453_Roseiflexus_sp.           QTRFVLRKALQAGHRAIVVVNKIDRPNARPNHVVNETFDLFIELGATDEQ
53795725_Chloroflexus_aurant        QTKFVLRKALQAGHHAIVVVNKIDRPQARPNHVVNETFDLFVDLGATDEQ
113940020_Herpetosiphon_aurant      QTKFVLRKALEQGHRAIVVVNKVDRPSARPNYVVNETFDLFIDLGATEEQ
15608305_Mycobacterium_tuberc       QTRFVLRKALAAHLPVILVVNKTDRPDARIAEVVDASHDLLLDVAAAEHA
94971430_Acidobacteria_bacteri      QTRYVLGKALEANLPPIVVINKIDRPDARAQEVLNEIYDLFIDLDAKEEQ
46446750_Protochlamydia_amoebo      QTRFVLSKSLKMGIKPIVVLNKIDRPHANPDRVLDLTFDLFSELGATDEQ
89284086_Tetrahymena_thermoph       QTKFVLKKALKQGLKPIVIINKVDRPTARVKEVESEVLDMFIEMEVNEDL
42733852_Dictyostelium_discoi       QTKFVLSKALSQGLRPIVVINKLDRSTARVDEVENEIFDLFATLGASDEQ
34909598_Oryza_sativa               QTKFVLSKALKYGLRPILLLNKVDRPEETCNEVESLVFDLFANLGATEEQ
79323586_Arabidopsis_thalia         QTKFVLAKALKYGLRPILLLNKVDRPEERCDEVESLVFDLFANCGATEEQ
15889919_Agrobacterium_tumefa       QTKFVVSKALKVGLRPIVAINKIDRPDGRHEEVINEVFDLFANLDATDEQ
51473460_Rickettsia_typhi           QTKFVLSKALNLGLKPIVVINKIDRDDQRIKEVIDEVFELFVALEANNDQ
56696900_Silicibacter_pomeroy       QTKFVTSKALALGLRPIVVLNKVDKPDAEPDRALDECFDLFANLGANDEQ
23612528_Plasmodium_falcip          QTKFVLKKSLLNPCKIIVIMNKFDKPMKKKEEIENEIFDLFVDLNAPEES
                                    **::*  :::      :: :** *:             :::      :

34762648_Fusobacterium_nuclea       LEFPVIYASGKSGFARKELDMQPLFETILEHVQDPDVTKPTQFLITNIAY
28210762_Clostridium_tetani         LDFPVVYCSARSGIAKKQLNMIPLFDTIIKNVKAPYIDKPFQILITTIDS
6458935_Deinococcus_radiod          LDFPVLYAIAREGKAFKDLDFKELFDMVLEHIPAPDLDSPFQMLVTNLDY
51892172_Symbiobacterium_therm      LDYPVLYASARKGVATYRLDLQPIFDTILERIPCPDPDAPLQAMVTSLDY
67920868_Crocosphaera_watsonii      CDFTTLYASGLSGFAKDNLDMKPLFEAILHHVPPPDVSKPLQLQVTTLDY
16329356_Synechocystis_sp.          CDFTTLFASGLGGFAKESLDMKPLFEAILHHVPPPDPNKPLQLQVTTLDY
33861319_Prochlorococcus_marin      CDFPYLFGSGLSGFAKEEMNMMPLFEAILRHVPPPDLNKPLQLQITTLDY
69247248_Enterococcus_faecium       LDFPVIYASALNGTSSLSDTMAPIFDTIIEKIPAPNSDEPLQFQVSLLDY
16078541_Bacillus_subtil            LEFPVVYASAINGTASLDPNMEALYETIIKHVPAPNAEEPLQFQVALLDY
21674245_Chlorobium_tepidu          LDFPYIFASAKNGIAKCNMDMSLLLDMIVKEIPAPDDDAGFQMLVTSLDY
57169035_Campylobacter_coli         LDFAIVYAAAKNGYAKLDLNMEPLFKTILERVPAPTNDNPLQLQVFTLGY
15611499_Helicobacter_pylori        LDFPVVYAAARDGYAMKSLNLEPLFETILEHVPSPSVDEPLQMQIFTLDY
45658203_Leptospira_interr          LDFPIVYASAKQGWAVHNLNLDPLLDTVLKHVPPVDTEAPLQFQVTSLDY
39995607_Geobacter_sulfur           LDFPIVYTSAKLGFAKLDLSMEPLFAVIESNVHPPDSKAPFQLLVTNIDY
24375888_Shewanella_oneide          LDFPIVYASALNGFATLDPDMTPLFQTIVEKVSSPDADGPFQMQISQLDY
16272804_Haemophilus_influe         LDFPIIYASALNGVAGLEHDMTPLFEAIVKHVEPPELDAPFQMQISQLDY
59801217_Neisseria_gonorr           LDFPIVYASGLSGFAKLEEDMRPLFDTILKYTPAPSADEPLQLQISQLDY
30250473_Nitrosomonas_europa        LDFPVVYASALNGYATLDADMRPLFDMILKHVPAPDPDQPLQLQISALDY
32474280_Rhodopirellula_baltic      DSAAYVFASAKEGYATDDPDMRPLLDLLVDHLPGPDTKSDFQMMVTTLDW
53715742_Bacteroides_fragil         LDFPTIYGSAKNNWMSTDWSIVPLLDCIVENIP-AQLEGTPQMLITSLDY
```

```
48854524_Cytophaga_hutchi             LDFVTLYGSGRGGWMSTDWDIIPLLDAIVDHIP-PVHEGIPQMQITSLDY
91201932_Candidatus_Kuenenia          LDFKIVYASGRNGYAKLSLDIRPLLDAIIQFVPKPDAEFPLQMQIASLDY
30684514_Arabidopsis_thalia           CDFQAIYASGIKGKAGLSPDLGPLFEAIIRCVPGPEKDGALQMLATNIEY
30684509_Arabidopsis_thalia           CDFQAIYASGIKGKAGLSPDLGPLFEAIIRCVPGPEKDGALQMLATNIEY
50906979_Oryza_sativa                 CDFQTVYASGIKGKAGLSPDLGPLFEAILRCIPEPEKDGALQLLVSNTEY
CM3614_Cyanidioschyzon_merolae        ADFPVVYASALKRVAGYAPDMGPLFEEILR-LPRPALDAPLQLLVSNISS
106888453_Roseiflexus_sp.             AEFATIYTNALKGVAGRSPSLEPLFDAILDQIPAPDPDGPAQLLVTNTVY
53795725_Chloroflexus_aurant          AEFATIYTNALLGHAGRSPSLEPLFECILDRIPPPDVEGPVQFLVTTSSY
113940020_Herpetosiphon_aurant        AEFPIVYASGIAGTAGHDPSLTPLFETILQYVPAPDAHGPPQLLVTSTMY
15608305_Mycobacterium_tuberc         LGLPTLYASGRAGVASTTANLDPLFEVLEKHVPPPEPDAPLQALVTNLDA
94971430_Acidobacteria_bacteri        LEFPVLYTNAKIGTASTDADLQPLFEAIVKTIPPPKTDGPLQVLVANLDY
46446750_Protochlamydia_amoebo        LDFRYCYASGLSGFAMHHMDMRPLFELITYAVQQPSLENPFLMHVSTITY
89284086_Tetrahymena_thermoph         LDYPVYYASGREGWAVPSIGVACVMDAIVSHVPYPQVEGDFQMLISQTES
42733852_Dictyostelium_discoi         LNYPTLYASGRQGWAIRSRDILPLLDTIVEYVECPKPEEPFTMLVTNLES
34909598_Oryza_sativa                 LDFPVLYASAKEGWASLEFNMSALLDSILQHVPSPDLEAPFQMLVSMMER
79323586_Arabidopsis_thalia           LDFPVLYASAKEGWASSTYNMADLLDAVVRHVQPPNLDEPFLMLVSMMEK
15889919_Agrobacterium_tumefa         LDFPILYGSGRDGWMNVNPGLAPLLDLVLEHVPEPE-EGPFRLIGTILEA
51473460_Rickettsia_typhi             LDFPIVYASGRAGTASLNFNLSPLFNLIVTHVPTPDNKAPFSMLVTTREY
56696900_Silicibacter_pomeroy         LDFPHLYASGRSGWADTDLDLTALFELVVRHVPAPRKDEPFRMLATTLGS
23612528_Plasmodium_falcip            MNYPILYASAKNGWCTDNFDVLVIFKKIIEYIDSPILKEPFCMLVSLIDH
                                            :  .          .  :   :

34762648_Fusobacterium_nuclea         DNYVGKLAVGRIHNGTLKRNQDVMLIKRDGKQKVSVLYGYEGLKRVEIEE
28210762_Clostridium_tetani           NEYVGRIGVGKVERGVVKRNQQAALMRKDGKIKISKLYTYSGLQREETEE
6458935_Deinococcus_radiod            SEYLGRIVLGRVARGTVKKGEFVQLMHKDGTMRVVQPFTHLGLKRIEVDE
51892172_Symbiobacterium_therm        DEYVGRVAIARVRQGTVRAGQQVAVAKLDGSVRAAQLFGFHGLKRVPVEV
67920868_Crocosphaera_watsonii        SEYLGRIMIGRIHNGTLKAGQQAALMKEDGTIKISKLLGFEGLQRVELEE
16329356_Synechocystis_sp.            SDYLGRIIIGRIHNGTVKAGQQAALVKEDGSIKVSKLLGFEGLNRIELPE
33861319_Prochlorococcus_marin        SDFLGRIVIGKIHNGTIKNGQQASLIKESGKTKVSKLLGFEGLQRIDINE
69247248_Enterococcus_faecium         NDYVGRIGIGRVFRGTIKVGDQVALIKLDGTVRVTKLFGFFGLKRLEIQE
16078541_Bacillus_subtil              NDYVGRIGIGRVFRGTMKVGQQVSLMKLDGTARVTKIFGFQGLKRVEIEE
21674245_Chlorobium_tepidu            SDYIGKIAIGRIQRGKVAPGNQLTLVTQDGVVTVTKLFLFDRTQRVEAME
57169035_Campylobacter_coli           DNFVGKIGIARIFNGVVKKNQSVMLAKADGTKRISKLIGFMGLEKMDIEE
15611499_Helicobacter_pylori          DNYVGKIGIARVFNGSVKKNESVLLMKSDGSKRITKLIGFLGLARTEIEN
45658203_Leptospira_interr            NDYVGRIAVGKIYAGKMALGMNVIQLAAKKTERITKLYNFEGLKRNEVNT
39995607_Geobacter_sulfur             NDYIGRIATGRIFNGRVKAGETVALVRRDGSIRITKLLGYEGLKQVEIPE
24375888_Shewanella_oneide            NSYVGVIGVGRINRGSIKTNQQVSVIGADGKVKMGQVLGYMGLERTEVDV
16272804_Haemophilus_influe           NNYVGVIGIGRIKRGSIKPNQPVTIINSEGKTRIGQVLGHLGLQRYEEDV
59801217_Neisseria_gonorr             DNYTGRLGIGRILNGRIKPGQTVAVMNHEQQIRINQLLGFKGLERVPLEE
30250473_Nitrosomonas_europa          SSFVGRLGIGRINRGRLKPGQEVMVLTGERAPRVNQVSGFRGLDRIQLSE
32474280_Rhodopirellula_baltic        SEYVGRIAVGRVNAGTIQTGQAVDVHTIDSSGKASGLYVFDKLGRVPAES
53715742_Bacteroides_fragil           SSYTGRIAVGRVHRGTLKEGMNVSLAKRDGSIKIKEVHVFEGLGRVKTTE
48854524_Cytophaga_hutchi             STFTGRIAIGRVFRGELKEGGQYSLTKMDGSIKIKELHSFEGLGRVRVES
91201932_Candidatus_Kuenenia          NDYVGRIGIGRIFSGTITTGQQVVRIDKTGKQKITDLFAFVGLSREKTLL
30684514_Arabidopsis_thalia           DEHKGRIAIGRLHAGVLRKGMDVRVCTSEDSCRVSELFVYEKFYRVPTDS
30684509_Arabidopsis_thalia           DEHKGRIAIGRLHAGVLRKGMDVRVCTSEDSCRVSELFVYEKFYRVPTDS
50906979_Oryza_sativa                 DEHKGRIAIGRLHAGELQRGMEVKVCTPDDACKISELFVYQNFSRVPVDN
CM3614_Cyanidioschyzon_merolae        DDYKGILGIGRIHAGTLRKGATVRVGTPAKDERVDEVFLFENLGRVSAES
106888453_Roseiflexus_sp.             DDYKGKILIGRLRRGVLRKGQMAARIARDGSVKIGQLFVFNGLERREVEE
53795725_Chloroflexus_aurant          DEYKGKILTGRLVRGTIRKGQPLVRIARDGTIKVSQIFVYNGLERQEVEQ
113940020_Herpetosiphon_aurant        DDYKGKIIIGRLNSGTIRKGQAVAHIAAGKDPKVVQVFTHYGMERVEVDE
15608305_Mycobacterium_tuberc         STFLGRLALIRIYNGRIRKGQQVAWIRQVDGQKITELLATEGVERKPTDA
94971430_Acidobacteria_bacteri        SDYLGRIAIARVFNGTLFTGEDVV-IAKLDGSKITKLFSFSGLKRVDITE
46446750_Protochlamydia_amoebo        DDYVGRQACGKILEGTVKKGQQLIHIDENGVETITRIEGHLGIEKVEMEE
89284086_Tetrahymena_thermoph         NQYHGKMVIGKILRGQLNVGDKLTSVDSSGNLKVMKIVRRYGMKQMEINT
42733852_Dictyostelium_discoi         DPFVGRIVTGKIYSGKAKVGQPLRVVDMKGNVKITKIFCRRAMERIVLEE
34909598_Oryza_sativa                 DFYLGRILTGRVRSGVVRVGDKVHGLRSTDDGQVVKLMKKKGTSMVIVEA
79323586_Arabidopsis_thalia           DFYLGRILTGRVTSGVVRVGDRVNGLRKTDSG-VVKLMKKKGTTIVSIDA
15889919_Agrobacterium_tumefa         NPFLGRIITGRIASGSIKPNQAVKVLGQDGKLRISKILAFRGIERTAIEE
51473460_Rickettsia_typhi             NPFFGRVLTGRIQSGTVKINQNVKVLNHENKVRITKILAFRGLERIAIDH
56696900_Silicibacter_pomeroy         DPFIGRILTGRVESGTLKAGETLKALSRDGTQRASKILAFRGLGQQPIDV
23612528_Plasmodium_falcip            QEGIGVTITGKIFKGVIKKGDSIVVKTEENKLIIKNIFYMKGLKIENVPF
                                      .   *     ::  *    .

34762648_Fusobacterium_nuclea         AEAGDIVCVAGIDDIDIGETLADINEPVALPLIDIDEPTLAMTFMVNDSP
28210762_Clostridium_tetani           AKIGDIVAVAGIDDINIGETLADIQNPEALPFVEIDEPTLTMNFMVNDSP
6458935_Deinococcus_radiod            VGAGDIVALAGIEDAQIGETIADLAEPEALPIITVDEPTVSMTFQPNTSP
51892172_Symbiobacterium_therm        ATVGDIIAMTGLEDVNIGETITDPENPQPLPPIKVDEPTLQMTFRTNDSP
67920868_Crocosphaera_watsonii        SNAGNIVAVAGFADANIGETLTCPDEPQALPLIKVDEPTLQMTFSVNDSP
16329356_Synechocystis_sp.            ASAGYIVAIAGFADANIGETLTCPDEPQALPLIKVDEPTLQMTFSVNDSP
33861319_Prochlorococcus_marin        AFAGDIVAVSGFDDVNIGETIACPDSPHPLPLIKVDEPTLNMTFVVNDSP
69247248_Enterococcus_faecium         AKAGDLIAVSGMEDIFVGETVTPVDHQDALPILHIDEPTLQMTFLVNNSP
16078541_Bacillus_subtil              AKAGDLVAVSGMEDINVGETVCPVDHQDPLPVLRIDEPTLQMTFVVNNSP
```

```
21674245_Chlorobium_tepidu          ATAGDIVALAGIAAANVGETLTTPDQPEPIESFEISKPTLSMLFSVNDSP
57169035_Campylobacter_coli         AGSGDIVAIAGFEALDVGDSVVDPNNPMPLDPLHIEEPTLSIVFSVNDGP
15611499_Helicobacter_pylori        AYAGDIVALAGFNAMDVGDSVVDPTNPMPLDPMHLEEPTMSVYFAVNDSP
45658203_Leptospira_interr          AEAGDIVAIAGLPDVFIGDTICEPGKPAPRPAIEVEEPTVSMFFMVNNSP
39995607_Geobacter_sulfur           ACTGDIVTVAGFDDVGIGETLAAADNPVALPYVSIDEPTISMNFIVNSSP
24375888_Shewanella_oneide          ANAGDIVAITGLGELKISDTVCAAGNVEAMPPLSVDEPTLTMTFQVNTSP
16272804_Haemophilus_influe         AYAGDIVAITGLGELNISDTICDINTVEALPSLTVDEPTVTMFFCVNTSP
59801217_Neisseria_gonorr           AEAGDIVIISGIEDIGIGVTITDKDNPKGLPMLSVDEPTLTMDFMVNTSP
30250473_Nitrosomonas_europa        AAAGDIVLISGIEELGIGTTLADIDHPEALPMPVVDEPTLSMNFQVNTSP
32474280_Rhodopirellula_baltic      AGAGDLIALEGLDDVEIGDTITAPETGKALPRLKVDEPTLEMVFSVNSSP
53715742_Bacteroides_fragil         VSSGDICALVGIDGFEIGDTICDYENPEALPPIAIDEPTMSMLFAINDSP
48854524_Cytophaga_hutchi           VLAGDICAVVGIEDFEIGDTITDLEKPEALPRIAVDEPTMSMLFTINNSP
91201932_Candidatus_Kuenenia        AMAGDIIALTGIENIDISDTITSVENPQAMPLIHIDEPTLSMVFSVNNSP
30684514_Arabidopsis_thalia         VEAGDICAVCGIDNIQIGETIADKVHGKPLPTIKVEEPTVKMSFSVNTSP
30684509_Arabidopsis_thalia         VEAGDICAVCGIDNIQIGETIADKVHGKPLPTIKVEEPTVKMSFSVNTSP
50906979_Oryza_sativa               VSAGDICAVCGINDIMIGETIADKVSGTPLPTIKIEEPTVRMSFSINTSP
CM3614_Cyanidioschyzon_merolae      AHAGDIVMVAGVDDIRIGETICDAADPRLLPPVQIEEPTVRMSLAINTTP
106888453_Roseiflexus_sp.           VTAGDIIAIAGIGDAGIGDTISDAHHPEPLPPIAVEEPTVRMTFSVNTSP
53795725_Chloroflexus_aurant        AQAGDIIAIAGISDAAIGDTIADALAPEALPIKVEEPTVRMTFGVNTSP
113940020_Herpetosiphon_aurant      ITAGDIVAMTGINDAGIGDTIADKDNPVALPPIKVEEPTVQMTFGVNTSP
15608305_Mycobacterium_tuberc       AVAGDIVAVAGLPEIMIGDTLAASANPVALPRITVDEPAISVTIGTNTSP
94971430_Acidobacteria_bacteri      TELGDIVAVAGVEGITIGETITDADNPKPLPHIAIDEPTIAMQFMVNTSP
46446750_Protochlamydia_amoebo      AGVGDIVILSGIPEVTIGDTICDPKKIVRLPRIKLDEPTVSVDMTVNNSP
89284086_Tetrahymena_thermoph       AYAGDIVSVAGFSNSTVAYTLNKYGCLDAVPSIPIDPPIMSVSIATNTSP
42733852_Dictyostelium_discoi       GEAGDIVGIAGFSQATVTNSLIAEGVKEPIKAIPIDPPVLAMYFEANNSP
34909598_Oryza_sativa               AGAGDIISMAGLAAPAIGHTVSNSDILTALPTIELDPPTISMTFGVNDSP
79323586_Arabidopsis_thalia         AGAGDIICMAGLTAPSIGHTVASAEVTTALPTVELDPPTISMTFGVNDSP
15889919_Agrobacterium_tumefa       AHAGDIVAIAGLSKGTVADTFCDPSVTEAMTAQPIDPPTVTMSFIVNDSP
51473460_Rickettsia_typhi           ATAGDIIALAGVENANVADTICAPEVTEAIPSLPIDPPTLSMTFSINDSP
56696900_Silicibacter_pomeroy       AEAGDIVTIAGMSKATVADSLVNPQVSEALPAQPIDPPTISVTFGINDSP
23612528_Plasmodium_falcip          ASAGDIVNITIAKGVTVNDTITSDENMESLKTRPIDPPVLCLKISQNTSP
                                       * :  :       :  :.             :. * : : :  *  *

34762648_Fusobacterium_nuclea       FVGKEGKFVTSRHIWDRLQKEIQTNVSMRVEATFIVKGRGELQLSILLEN
28210762_Clostridium_tetani         FAGKEGDYLTSRHLRDRLLKELETNVSLKVEETFKVSGRGELHLSVLIET
6458935_Deinococcus_radiod          FAGKEGKYVTSRHLNDRLKREVMTNVSLKVEEVFVVSGRGELHLSILLET
51892172_Symbiobacterium_therm      FAGQEGKYVTSRHLRARLFKELERNVALRVEETFIVSGRGELHLSILIET
67920868_Crocosphaera_watsonii      FAGQEGKFVTSRQIRDRLMRELETNVALRVEPGFSVSGRGELHLGILIET
16329356_Synechocystis_sp.          FAGQEGKFVTSRQIRDRLNRELETNVALRVEDGFLVSGRGELHLGILIET
33861319_Prochlorococcus_marin      FAGKEGKFVTSRQLKNRLERELLTNVALRVEETFSVSGRGELHLGILIET
69247248_Enterococcus_faecium       FAGREGKFVTARKIEERLMAELQTDVSLRVEPTWTVSGRGELHLSILIEN
16078541_Bacillus_subtil            FAGREGKYVTARKIEERLQSQLQTDVSLRVEPTWVVSGRGELHLSILIEN
21674245_Chlorobium_tepidu          FAGQEGKEVTSRKIRERLMKEIMTNVALNVEETFRVSGRGELHLSVLIET
57169035_Campylobacter_coli         LAGTEGKHVTSNKIAERLEAEMKTNIAMKYESTFKVSGRGELQITILAEN
15611499_Helicobacter_pylori        LAGLEGKHVTANKLKDRLLKEMQTNIAMKCEEMFKVSGRGELQITILAEN
45658203_Leptospira_interr          FAGKEGKFVTTRNIRERLDRELETNVAMRLEETFKVLGRGELHLSVLIET
39995607_Geobacter_sulfur           FAGREGKFVTSRNIRERLDRELRTNVSLRVEDTFKVSGRGELHLSILIEN
24375888_Shewanella_oneide          FAGKEGKYVTSRNILERLQQELVHNVALRVEETFAVSGRGELHLSILIEN
16272804_Haemophilus_influe         FAGQEGKYVTSRQILERLNKELVHNVALRVEETFRVSGRGELHLSVLIEN
59801217_Neisseria_gonorr           LAGTEGKFVTSRQIRDRLQKELLTNVALRVEDTFRVSGRGELHLTILLEN
30250473_Nitrosomonas_europa        FAGKEGKFVTSRQLRERLEKELLTNVALRLEETFLVSGRGELHLTILLEN
32474280_Rhodopirellula_baltic      LAGREGKYVTTRQIKARLEKELERNVALRVSMIYAVAGRGVLHLAVLIET
53715742_Bacteroides_fragil         FYGKDGKFVTSRHIHDRLTKELDKNLALRVRKSWVVSGRGVLHLSVLIET
48854524_Cytophaga_hutchi           FFGKEGKFVTSRHLRDRLMKELEKNLALRIQPGFLVFGRGILHLSVLIET
91201932_Candidatus_Kuenenia        FCGKDGKFVTSRNLRERLFRELRANVALKVDETFCISGRGLLHLSILIEN
30684514_Arabidopsis_thalia         FSGREGKYVTSRNLRDRLNRELERNLAMKVEDGFIVSGRGTLHITILIEN
30684509_Arabidopsis_thalia         FSGREGKYVTSRNLRDRLNRELERNLAMKVEDGFIVSGRGTLHITILIEN
50906979_Oryza_sativa               FVGKEGKYVTSRNLRDRLYRELERNLAMKVEDGFLVSGRGTLHLTILIEN
CM3614_Cyanidioschyzon_merolae      FAGREGRFLTSRQLRERLERETRTNVALRIQDGFTICGRGALHLTILIES
106888453_Roseiflexus_sp.           FAGREGTHVTSRKLRERLYQELERDVALRVADTFTVSGRGELHLTILIET
53795725_Chloroflexus_aurant        FAGREGTYVTSRKLRERLFQEMERDVALRVEETFIVAGRGELHLGILIET
113940020_Herpetosiphon_aurant      FSGREGKLVTSRKIRERLYGETERDVALKVEDTFRVSGRGELHLAILIEN
15608305_Mycobacterium_tuberc       LAGKVGHKLTARMVRSRLDAELVGNVSIRVVDIWEVQGRGELALAVLVEQ
94971430_Acidobacteria_bacteri      FAGKDGQYVTSRNLRERLAKELLTNVSLRVEDSFKVAGRGELQLSILIEM
46446750_Protochlamydia_amoebo      FVGRSGKHVTMNKIRDRLEKEKRANISLRIEDEITVSGRGELHLAVLVEA
89284086_Tetrahymena_thermoph       LAGKDGQKLTYSQIRQRLQEESENDVALKVQGLLIVEGRGDLHLGILFEN
42733852_Dictyostelium_discoi       FAGKEGTHCTTLKIKSRLLKEIESNVSLQVNVTFEVKGRGELQLGILLEN
34909598_Oryza_sativa               LSGRDGTHLTGAKIGNRLMAEAETNLAINVLPGYEVQGRGELQLGILIEN
79323586_Arabidopsis_thalia         LAGQDGTHLTGGRIGDRLMAEAETNLAINVIPGYEVQGRGELQLGILIEN
15889919_Agrobacterium_tumefa       LAGTEGDKVTSRVIRDRLFKEAEGNVALKIEEAFFVSGRGELQLAVLIET
51473460_Rickettsia_typhi           LAGSEGTKITSSLIGARLMRELESNVALKVTETFQVAGRGELQLGILIET
56696900_Silicibacter_pomeroy       LAGRDGKKVQSRVIRDRLMKEAESNVAIRVTDTFEVAGRGELQMGVLIEN
23612528_Plasmodium_falcip          LTGKDGKHITLSSIGNRLKKEAAINIAIEISESYEVKGRGELQLGILIEK
```

```
                                    : *  *       :  **  :   ::::.      : *** * : :* *

34762648_Fusobacterium_nuclea       MRRESFEVQVSKPRVLFKEKGKKLEPIELALIDVDDSYTGVVIEKMGVRK
28210762_Clostridium_tetani         MRRQGYEFQVSKPSVIFKEEGKKLEPIEYLTIDVPEEYMGTVMEKLGPRK
6458935_Deinococcus_radiod          MRREGYEVQVGSPRVIIREIGEKHEPIEHLVIDVPEQHASTVIGVLGARK
51892172_Symbiobacterium_therm      MRREGYELAVSKPKVIYRYDGNRLEPLEELIIDVPEEYMGAVMEALGIRK
67920868_Crocosphaera_watsonii      MRREGYEFQVAQPQVIYREVGQPYEPFEYLVLDVPEEAVGSCIERLGQRK
16329356_Synechocystis_sp.          MRREGYEFQVAQPQVIYREVGQPCEPVEYLVLDVPEAAVGACIERLGQRR
33861319_Prochlorococcus_marin      MRREGFEFQISQPQVIFREIDVQCEPIETLVLDVPEVAVGSCIEKLGSRK
69247248_Enterococcus_faecium       MRREGYELQVSRPEVIEKEIGVKCEPFERVQIDTPEEYMGSVIESLSLRK
16078541_Bacillus_subtil            MRREGYELQVSKPEVIIKEIGVRCEPVERVQIDVPEEHTGSVMESMGARK
21674245_Chlorobium_tepidu          MRREGYELAISRPEVILREEGVTMEPVEHVTIDVPEEYTGVVIEKMGRRK
57169035_Campylobacter_coli         MRREGFEFCMGRPEVIVKVEGVKTEPFEHLVIDVPEEFSGAVIEKLGKRK
15611499_Helicobacter_pylori        LRREGFEFSISRPEVIIKEEGVKCEPFEHLVIDTPQDFSGAIIERLGKRK
45658203_Leptospira_interr          MRREGFEIQVSRPEVILKTNGQKLEPYEYLVMDVPDQFTGAIIAELNRRK
39995607_Geobacter_sulfur           MRREGFEMAVSKPEVILRVIGTKMEPMEYLVVDVPSEFQGAIIEKMGPRK
24375888_Shewanella_oneide          MRREGYELAVSRPEVILKTIGELCEPFETLTVDVEEEHQGTVIEKLGIRK
16272804_Haemophilus_influe         MRREGYELAVSRPKVIYRDIGKKQEPYEQVTIDVEEQHQGSVMEALGIRK
59801217_Neisseria_gonorr           MRREGYELAVGKPRVVYRDIGQKCEPYENLTVDVPDDNQGAVMEELGRRR
30250473_Nitrosomonas_europa        MRREGYELAVSRPKVVIREIGEKCEPFEVLTVDIDEANQGAVMEALGTRR
32474280_Rhodopirellula_baltic      MRREGYELSVGKPRVVFKEIGKKHEPFETLRVEVPTEVMGPVMELVGLRR
53715742_Bacteroides_fragil         MRREGYELQVGQPQVIYKEIGVKCEPIEELTINVPEEYSSKIIDMVTRRK
48854524_Cytophaga_hutchi           MRREGYELQVGQPQVLYKEIGKRCEPVEVLVVDVPQETSGKVIELATQRK
91201932_Candidatus_Kuenenia        MRREGYELQVSKPKVIFKEIGKRMEPMEYVVIDVPKEMEGQAISLLGARK
30684514_Arabidopsis_thalia         MRREGYEFMVGPPKVINKRVDKLLEPYEIATVEVPEAHMGPVVELLGKRR
30684509_Arabidopsis_thalia         MRREGYEFMVGPPKVINKRVDKLLEPYEIATVEVPEAHMGPVVELLGKRR
50906979_Oryza_sativa               MRREGFEFMIGPPKVINKTVGKLQEPYEIAAVEVPEEYMGSVVELLGKRR
CM3614_Cyanidioschyzon_merolae      MRREGFEMIVGAPEVIFKMDNTPLEPFDHVEVQIPEEYLGAVVDALAKRR
106888453_Roseiflexus_sp.           MRREGYEFQVSRPEVIFKEIGVKYEPIEQVELEVAEQYQGAVIELMGQRR
53795725_Chloroflexus_aurant        MRREGYEFQVSKPEVIFREAGTRLEPMELVEIEVASEYQGVVVELLGKRQ
113940020_Herpetosiphon_aurant      MRREGYEFQVSKAEVIYKDIGTRHEPYELVEIEVDSDYQGSVVELMGLRR
15608305_Mycobacterium_tuberc       MRREGFELTVGKPQVVTKTIGTLHEPFESMTVDCPEEYIGAVTQLMAARK
94971430_Acidobacteria_bacteri      MRREGYELMVGKPEIVTKHIGKLVEPIEKLTVDIPETFIGVVIEKIGTRK
46446750_Protochlamydia_amoebo      MRREQYEFSISKPRVIIKEVGEKYEPIERVHIEVPQDYSGTVIEELSRRK
89284086_Tetrahymena_thermoph       MRREGFEMSLTPPQILFKEIGKQHEPIEKVTIELDPVYSANVIEKLSQRK
42733852_Dictyostelium_discoi       MRREGYEVAVSQPRVVFTKDGNLMEPQEEVTIDVDTEYSGAIIEKLSKRN
34909598_Oryza_sativa               MRREGFELSVSPPKVMYRTDGERLEPIEEVTVEVDEEHVGFVMETLTHRK
79323586_Arabidopsis_thalia         MRREGFELSVSPPKVMYKTEGQKLEPIEEVTIEINDEHVGLVMEALSHRR
15889919_Agrobacterium_tumefa       MRREGFELAVSRPRVVMHKDGTLLEPIEEVVIDVDEEHSGVVVQKMSERK
51473460_Rickettsia_typhi           MRREGFELSISRPEVLYHTDGNKQEPIEEIQVDVDDDYIGVVVKSLALRK
56696900_Silicibacter_pomeroy       MRREGFELSISRPQVLFR-EGQRLEPVEEVTIDVDDEYTGAVIEKVTPRR
23612528_Plasmodium_falcip          LRREGYEMTISSPNVIYTKDGNLLEPIEEYHITIPTSMTSNVIEKLNTRK
                                    :**: :*. :  . ::    .   ** :   :       .        *.

34762648_Fusobacterium_nuclea       AEMVSMVPGYTRLEFKVPARGLIGFRNEFLTDTKGTGILNHSFFDYEEYK
28210762_Clostridium_tetani         GDMVNMTSAYSRLEFRIPARGLIGFRSEFMTDTRGNGIMNHIFDGYEPYK
6458935_Deinococcus_radiod          GQMVNMEPQRVRVEFKIPARALFGFRTQFLSMTQGEGIMSHIFDGYAPWA
51892172_Symbiobacterium_therm      AEMTNMVNHNVRLEFIIPARGLVGFRSEFLTMTRGYGVMHHMFHGYGPYR
67920868_Crocosphaera_watsonii      GEMQDMQTGRTQLEFVVPARGLLGFRGDFIRITRGEGIMNHSFLEYRPLS
16329356_Synechocystis_sp.          GEMQDMQTSRTQLEFVIPARGLLGFRGDFIRITRGEGIMNHSFLEYRPMS
33861319_Prochlorococcus_marin      AEMKNMQTSRTQLEFLVPSRGLIGFRGEFVRITRGEGIMSHSFYEYKPKA
69247248_Enterococcus_faecium       GEMQDMVHTQIRLTFLTPARGLIGYSTEFLSMTRGYGIMNHTFDQYLPML
16078541_Bacillus_subtil            GEMVDMINNQVRLIFTVPSRGLIGYSTEFLSLTRGFGILNHTFDSYQPMQ
21674245_Chlorobium_tepidu          AEMTNMSTLMNRLEFEIPTRGLIGYNLEFTTDTKGEGMMSHVFHNYQPYK
57169035_Campylobacter_coli         AEMKTMAPTQTRLEFEIPARGLIGFRSQFLTDTKGEGVMNHSFLEFRPFS
15611499_Helicobacter_pylori        AEMKAMNPMYTRLEFEIPARGLIGYRSEFLTDTKGEGVMNHSFLEFRPFS
45658203_Leptospira_interr          GELQLMDAHMTRVEFVIPTRGIIGFRGFFISETRGEGVMSSRFLRFDVYK
39995607_Geobacter_sulfur           GEMTSLQPMTVRLEFVVPARGLIGLRGELLTETRGTAVMTHTFHDYAPYK
24375888_Shewanella_oneide          AEMKDMQLDRVRVDFVIPSRGLIGFQTEFLTATSGTGLIYHSFDHYGPHK
16272804_Haemophilus_influe         GEVRDMLPDRVRLEYIIPSRGLIGFRGDFMTMTSGTGLLYSSFSHYDEIK
59801217_Neisseria_gonorr           GELTNMESDRTRLEYHIPARGLIGFQGEFMTLTRGVGLMSHVFDDYAPVK
30250473_Nitrosomonas_europa        GDLLDMVTDRVRLDYRIPARGLIGFQSEFMTLTRGTGIMSHVFDEYAPMR
32474280_Rhodopirellula_baltic      GQLEEMKQRYSLLRFIVPSRGLIGLRTRLLNATRGTAIIHHRFESYRMVE
53715742_Bacteroides_fragil         GEMTMMENTRINLEFDMPSRGIIGLRTNVLTASAGEAIMAHRFKEYQPFK
48854524_Cytophaga_hutchi           GELLIMEPKLQHLEFKIPSRGIIGLRTNVLTATFGEAIMTHRFDGYEPYK
91201932_Candidatus_Kuenenia        GEIVSMKTEYTHFEFKAPSRGLIGLRNRLLSSTRGEAVMHHNFYDYEPYK
30684514_Arabidopsis_thalia         GQMFDMQGVTTFLRYKIPTRGLLGLRNAILTASRGTAILNTVFDSYGPWA
30684509_Arabidopsis_thalia         GQMFDMQGVTTFLRYKIPTRGLLGLRNAILTASRGTAILNTVFDSYGPWA
50906979_Oryza_sativa               GQMVDMQPSTTLLKYKIPTRGLIGLRNAVLTASRGTAILNTIFDSYGPWA
CM3614_Cyanidioschyzon_merolae      GEMLNLTHSYSIVEYIVPTRGLFGLNNALLTLTRGTAIIHTSFAAYRPYV
106888453_Roseiflexus_sp.           GQMRDMSIRLVHMIFHVPTRGLLGFRQTFLTATRGEGVMNSLFIGYEPLA
53795725_Chloroflexus_aurant        GQMRDMRISSVHYVYLVPTRGLLGFRQLFLTATRGTGIMHSIFFGYEPYA
113940020_Herpetosiphon_aurant      GTMRDMSVSSVQFVYHVPTRGLLGFRQQLLTATRGTGIMNSLFAGYQPYA
```

```
15608305_Mycobacterium_tuberc       GRMVEMANHWVRMDFVVPSRGLIGWRTDFLTETRGSGVGHAVFDGYRPWA
94971430_Acidobacteria_bacteri      GQMEKMTNHRVRMEFKVPSRGLIGLRSELLTDTRGTIVMNSLFAGYTEWQ
46446750_Protochlamydia_amoebo      GELQLLDTDITAIDFLIPTRGLMGYRNDFLTATRGLGILTSVFENFSPWK
89284086_Tetrahymena_thermoph       GVYENCEELLHKLSFTVPTRGMIGFRSELLNDTKGTAVLESCFLEYQEHR
42733852_Dictyostelium_discoi       GDITDMKQSKSRLVFLVPSRGLIGLRSEIINDTKGTGVMNHLFHSYVPHK
34909598_Oryza_sativa               GEVMDMGPVRTRIFLTCPSRGLVGFKGIFSSFTRGTGFMHRAFQAYAKYR
79323586_Arabidopsis_thalia         AEVIDMGPVRTRLSLTCPSRGLVGYRCVFSSDTRGTGFMHRAFLTYEKYR
15889919_Agrobacterium_tumefa       AEMAELRPSRVRLKFYAPTRGLIGYQSELLTDTRGTAIMNRLFHDYQPFK
51473460_Rickettsia_typhi           AEMTNMRPSKSRITFIGPSRGLIGYYNQFLTETRGTGIINRIFYGYTDYK
56696900_Silicibacter_pomeroy       GELVEMKPAKTRIIAHVPSRGLIGYHGEFLTDTRGTGVLNRVFHGWTPHK
23612528_Plasmodium_falcip          AEIVDIINDNTFIKCICPSRNFFGMRSYLRDISKGTSIINSELKEYKKKQ
                                    .                *:* :.*    .   : *  .    :  :

34762648_Fusobacterium_nuclea       GDIPTRNKGVLIATEPGVTVPYALNNLQDRGTLFLDPGVPVYEGMIVGEH
28210762_Clostridium_tetani         GEMPSKPRGSLVVFETGTAITYGLYNAQERGELFIGPGTEVYQGMIAGEY
6458935_Deinococcus_radiod          GELKTRQNGSLVSMEDGTSFAYSIWKLQDRGNFFIDAGQEVYVGMIVGEN
51892172_Symbiobacterium_therm      GEIPTRQRGSLVASETGTATQYALYQIQERGTLFIEPGTEVYVGMVVGEN
67920868_Crocosphaera_watsonii      GELETRYNGVIIAFEEGTSTFYAMKNAEDRGVFFIIPGTKVYKGMIIGEN
16329356_Synechocystis_sp.          GDLETRYNGVMVAFEEGVATFYAMKNAEDRGVFFITPGTKVYKGMIIGEH
33861319_Prochlorococcus_marin      GDFETRRNGVLISFEEGVATFYALKNAEDRGVYFIKPGVKVYKGMIIGEN
69247248_Enterococcus_faecium       GQIGGRHQGALVSIDTGKATTYSIMSIEERGTVFVEPGTEVYEGMIIGEN
16078541_Bacillus_subtil            GQVGGRRQGVLVSMENGKATSYGIQGIEDRGVIFVEPGTEVYEGMIVGEH
21674245_Chlorobium_tepidu          GKLPSRETGALVSAETGVAVAYAISSLEDRGTFFIGPNAKVYEGMVVGES
57169035_Campylobacter_coli         GAVEKRNNGALISMENGVALGYSLFNLQERGVLFIEPQTKVYTGMIIGEH
15611499_Helicobacter_pylori        GSVESRKNGALISMENGEATAFSLFNIQERGALFINPQTKVYVGMVIGEH
45658203_Leptospira_interr          GDIPGRKNGALISMDSGESTAYALWKIQERGELVIGPNTSVYPGMIIGIH
39995607_Geobacter_sulfur           GDIPGRKNGVLIAMELGETTAYALDALQPRGILFIGPGVEVYGGMIIGQH
24375888_Shewanella_oneide          GDIGQRANGVLISNATGKALTFALFGLQDRGRLFIGHAAEVYEGQVVGIH
16272804_Haemophilus_influe         GEIGQRKNGVLISNATGKALGYALFGLQERGKLMIDANIEVYEGQIIGIH
59801217_Neisseria_gonorr           PDMPGRHNGVLVSQEQGEAVAYALWNLEDRGRMFVSPNDKIYEGMIIGIH
30250473_Nitrosomonas_europa        PDMASRKNGVLISAEMGEAVAYALWKLQERGRMFVSPGEPLYEGMVIGIH
32474280_Rhodopirellula_baltic      GDVPRRANGVLISMVGGKTMPFALFALQDRAELFVPPSTEVYEGMIVGEN
53715742_Bacteroides_fragil         GDIERRTNGSIIAMESGTAFAYAIDKLQDRGKFFIFPQEEVYAGQVVGEH
48854524_Cytophaga_hutchi           GAIPERVSGSIISMDSGDVTAYALDKLQDRGKFFIEPGDVIYQGMVIGEH
91201932_Candidatus_Kuenenia        GDIVHRTQGVLVSMAAGEATAYALEGLHDRGIMFVKPGEEVYEGMIVGEH
30684514_Arabidopsis_thalia         GDISTRDLGSLVAFEDGTSTSYALASAQERGQMFVGSGVDVYKGQIVGIH
30684509_Arabidopsis_thalia         GDISTRDLGSLVAFEDGTSTSYALASAQERGQMFVGSGVDVYKGQIVGIH
50906979_Oryza_sativa               GDLSSRDQGSLVAFEDGSTTSYALLNAQERGILFVSPGQDVYKGQIVGIH
CM3614_Cyanidioschyzon_merolae      GQVRLREVGSLLAHETGRVTTYGVEAAQERGQLFVAPGAEVYENQIVGEN
106888453_Roseiflexus_sp.           GEIVTRTNGSLIASESGVATAYALNQAQERGTLFITPGAEVYEGMIVGQH
53795725_Chloroflexus_aurant        GEIEMRANGSLIAAESGVATTYAIHALQDRGVFFITPGQEVYEGMVVGQH
113940020_Herpetosiphon_aurant      GDVQTRQFGSLIAWEQGVTSSYGLSSAQERGQLFVGSGVDVYEGMIIGQH
15608305_Mycobacterium_tuberc       GEIRARHTGSLVSDRAGAITPFALLQLADRGQFFVEPGQQTYEGMVVGIN
94971430_Acidobacteria_bacteri      GDIPHRPTGALVADRNGLTTAYALFGLQERGELFLGPGVDVYEGMVIGEN
46446750_Protochlamydia_amoebo      GTIPGRTRGVLLSNGPGKTSGYACFNLQDRGVMFVAPGDEVYEGMVVGEN
89284086_Tetrahymena_thermoph       GALKKNNKGPLICTSEGIVTAYALKTCEKFGQLFIEPGSKVYPGQVIGEN
42733852_Dictyostelium_discoi       GPMDNKEKGALISMSEGVATAFSLSALEDRGVLFIGPQTQVYSGMIIGEN
34909598_Oryza_sativa               GQLGNVRKGVLVSVGKGLITSHALMSLEARGILFVSPGMEAYEGMIVGEH
79323586_Arabidopsis_thalia         GPLGNVRKGVLVSMARGTITAHSLMSLEARGILFVSPGLDSYDGMIIGEH
15889919_Agrobacterium_tumefa       GQIAGRVNGVLLSNGSGEAVAYAMFNLEDRGPMIIEPGEKVYAGMIIGIH
51473460_Rickettsia_typhi           GPIEGRRKGVLISNCDGAAVAYALWNLEERGKMFINPGDKVYRGMIIGEH
56696900_Silicibacter_pomeroy       GAIPGRRAGVLISMENGQSVAYALWNLEERGRMFIGPQTDVYEGMIIGEH
23612528_Plasmodium_falcip          PSYKRDRNGVIISSSSGTTTAFSLDPIQQKGNLFVNENYPTYEGMIIGEH
                                            * ::    *    ..       .  .:      * . : *

34762648_Fusobacterium_nuclea       NRENDLVVNVCKTKKLTNMRAAGSDDAVKLATPRKFTLEQALDYIAEDEL
28210762_Clostridium_tetani         SRAGDIEVNVCKKKHLSNTRSSGADDALKLTPITPMSLEECLEFIAADEL
6458935_Deinococcus_radiod          AREQDMNVNVCKNKKLTNVRSSGADEALTLIPPKRLSLEDALEYIADDEL
51892172_Symbiobacterium_therm      SRGQDMDVNVCKTKHLTNIRAAGADEKLLLDPPRKLTLEEALEQIADDEL
67920868_Crocosphaera_watsonii      NRPQDLELNVCKTKQLTNHRAASGEELVQLQAPQEMSLERALEYIGSDEL
16329356_Synechocystis_sp.          NRPQDIELNVCKTKQLTNHRSATGDELVQLQAPEDMNLERALEYIGPDEL
33861319_Prochlorococcus_marin      NRSQDLELNICKTKQLTNMRSAGAEELDTLQSPVDITLERALEYIGPDEM
69247248_Enterococcus_faecium       SRDNDLTVNITKAKQMTNVRSATKDQTSVIKKPKQLTLEESLEFLNDDEY
16078541_Bacillus_subtil            NRDNDLVVNVSKMKQQTNVRSATKDQTTTIKKARIMSLEESLEYLNEDEY
21674245_Chlorobium_tepidu          TRDLDITVNICKTKKLTNMRASGSDDSIRLTPPKRLSLEQALEFINDDEL
57169035_Campylobacter_coli         SRPNDLDVNPIKGKNLTNVRASGSDDAIKLVPPRKLSLERALEWIEEDEL
15611499_Helicobacter_pylori        SRDNDLDVNPIKSKHLTNMRASGSDDAIKLTPPRTMVLERALEWIEEDEI
45658203_Leptospira_interr          SRDNDLEVNPVKEKKLSNVRSSGADEAIRLVPPRKFSLEQNIEFLDDDEL
39995607_Geobacter_sulfur           AKDNDLDVNPCKGKKLTNVRASGSDDAIKLTPPRILTLEQALEFIDDDEL
24375888_Shewanella_oneide          ARSNDLTVNCLKGKQLTNMRASGTDEAQVLTTPITLTLEQALEFIDDDEL
16272804_Haemophilus_influe         SRSNDLTVNCLQGKKLTNMRASGKDDAIVLTTPVKFSLEQAIEFIDDDEL
59801217_Neisseria_gonorr           SRDNDLVVNPLKGKKLTNIRASGTDEAVRLTTPIKLTLEGAVEFIDDDEL
30250473_Nitrosomonas_europa        SRENDLVVNPIKGKQLTNIRASGHDEAVVLTPPIQLTLESAIEFIADDEL
```

```
32474280_Rhodopirellula_baltic      ARENDMTVNPCREKKLTNMRASGSDENVILKPPRDMSLEAALEYIEDDEL
53715742_Bacteroides_fragil         AHEKDLVVNVTKSKKLTNMRASGSDEKARLIPPVQFSLEEALEYIKEDEY
48854524_Cytophaga_hutchi           NRQNDITVNVQREKKLTNMRASGTDDNAKIAPKINFSLEEMMEYIQKDEY
91201932_Candidatus_Kuenenia        CEQNDIVVNVIREKKATNIRQATGEKSVKLSPHREFSLEMALEYIEDDEL
30684514_Arabidopsis_thalia         QRPGDLGLNICKKKAATNIRSN-KDVTVILDTPLTYSLDDCIEYIEEDEL
30684509_Arabidopsis_thalia         QRPGDLGLNICKKKAATNIRSN-KDVTVILDTPLTYSLDDCIEYIEEDEL
50906979_Oryza_sativa               QRPGDLAINVCKKKAATNVRSN-KETTVVLDEALSYSLDDCIEFIQEDEL
CM3614_Cyanidioschyzon_merolae      KYPEDLRVNVCRVKQLTNHRSATKEVVKTLQGIRTLTLDDALEYISENEF
106888453_Roseiflexus_sp.           IRERDLEVNVCRRKHLTNIRSSTAEEGIRLETPRILSLDDAIEYISDDEL
53795725_Chloroflexus_aurant        IRDNDLEVNVCKEKHLTNMRNNRGAETIRLDAPRQLSLDDAIEYISDDEL
113940020_Herpetosiphon_aurant      IRDEDLEVNACKKKQLTNMRSSGADDALRLDVPRNMSLDDCIEYLADDEL
15608305_Mycobacterium_tuberc       PRPEDLDINVTREKKLTNMRSSTADVIETLAKPLQLDLERAMELCAPDEC
94971430_Acidobacteria_bacteri      SRDNDLDVNCVREKKLTNMRASTSDDAIRLVPFKNLNLEQAIDFIADDEL
46446750_Protochlamydia_amoebo      SRDNDLVVNVTKGKQLTNVRASGSDENIILIPARRFTLEQAIDYIQDDEL
89284086_Tetrahymena_thermoph       QKESEVELNPTKKKELNNIRTKSHEEKIVLQPHRVFSIEEAICYIRDDEI
42733852_Dictyostelium_discoi       SKLTDLDVNPVKSKQLTNIRTTVKEEGIRLSPPKLLKLEEAIACVKEDEL
34909598_Oryza_sativa               SRDSDLDINPVRTKELTNIRAPGKDENVRLSPPRLMSLEEAIGYVAADEL
79323586_Arabidopsis_thalia         SRETDLDLNPVKAKELTNIRSAGKDENVKLSPPRLMTLEEAIGYVASDEL
15889919_Agrobacterium_tumefa       TRDNDLEVNVLKGKQLTNIRAAGKDEAVKLTPPIRMTLDRALSWIQEDEL
51473460_Rickettsia_typhi           NRDNDLDVNPLKAKQLSNVRAVGKDEAIRLTPPMLLTLEQAISYIQDDEL
56696900_Silicibacter_pomeroy       SRDNDLEVNPLKGKKLTNVRASGTDDAVRLTPHIQFSLEEAIAYIDDDEL
23612528_Plasmodium_falcip          FLSNDIEMNAIKVKPVQHLRNKGHEDTIRIN-HKNITIEYALSFIQDDEE
                                        :: :*  : *   : *         :       ::  :     :*

34762648_Fusobacterium_nuclea       VEVTPTNIRLRK
28210762_Clostridium_tetani         VEVTPISIRMRK
6458935_Deinococcus_radiod          VELTPQSIRLRK
51892172_Symbiobacterium_therm      VEITPKSIRLRK
67920868_Crocosphaera_watsonii      VEVTPESIRLRK
16329356_Synechocystis_sp.          VEITPESIRLRK
33861319_Prochlorococcus_marin      LEVTPDSIRMRK
69247248_Enterococcus_faecium       CEVTPESIRLRK
16078541_Bacillus_subtil            CEVTPESIRLRK
21674245_Chlorobium_tepidu          LEVTPENIRIRK
57169035_Campylobacter_coli         VEVTPQNVRVRK
15611499_Helicobacter_pylori        LEVTPLNLRIRK
45658203_Leptospira_interr          LEVTPTSLRLRK
39995607_Geobacter_sulfur           VEVTPQSIRLRK
24375888_Shewanella_oneide          VEVTPKSIRVRK
16272804_Haemophilus_influe         VEVTPESIRIRK
59801217_Neisseria_gonorr           VEITPQSIRLRK
30250473_Nitrosomonas_europa        VEITPKSIRIRK
32474280_Rhodopirellula_baltic      VEVTPESIRLRK
53715742_Bacteroides_fragil         VEVTPKAMRMRK
48854524_Cytophaga_hutchi           IEVTPKSLRMRK
91201932_Candidatus_Kuenenia        VEITPKTFRLRK
30684514_Arabidopsis_thalia         VEVTPSSIRMCK
30684509_Arabidopsis_thalia         VEVTPSSIRMCK
50906979_Oryza_sativa               VEVTPASIRMCK
CM3614_Cyanidioschyzon_merolae      VEVTPQSIRMFK
106888453_Roseiflexus_sp.           VEVTPKSIRLRK
53795725_Chloroflexus_aurant        VEVTPKGWRIRK
113940020_Herpetosiphon_aurant      LEVTPLHFRLRK
15608305_Mycobacterium_tuberc       VEVTPEIVRIRK
94971430_Acidobacteria_bacteri      CEVTPKTLRLRK
46446750_Protochlamydia_amoebo      IEVTPDSIRMRK
89284086_Tetrahymena_thermoph       VEVTPKEIRIRK
42733852_Dictyostelium_discoi       IEITPKSIRLRK
34909598_Oryza_sativa               IEASFLYLLLMI
79323586_Arabidopsis_thalia         IEVTPKTIRLRK
15889919_Agrobacterium_tumefa       MEVTPKSIRLRK
51473460_Rickettsia_typhi           VEVTPKSIRLRK
56696900_Silicibacter_pomeroy       VEVTPNTIRLRK
23612528_Plasmodium_falcip          IEVTPKRIVMRK
                                     * :     :
```

Note: This gene is distributed in bacteria and various eukaryotes, including several protists. Sequences from TBestDB are too short for phylogenetic analyses. There are multiple versions of this gene in photosynthetic eukaryotes, one of which forms a monophyletic group with homologs GNS bacterial sequences with strong support (middle part of the tree). The other eukaryotic sequences group with alpha-proteobacterial homologs, likely resulting from intracellular gene transfer from mitochondria to the nucleus. Protein products of GNS bacteria-related genes (GIs: 30684514 and 30684509) are targeted to chloroplasts and intracellular components in *Arabidopsis*.

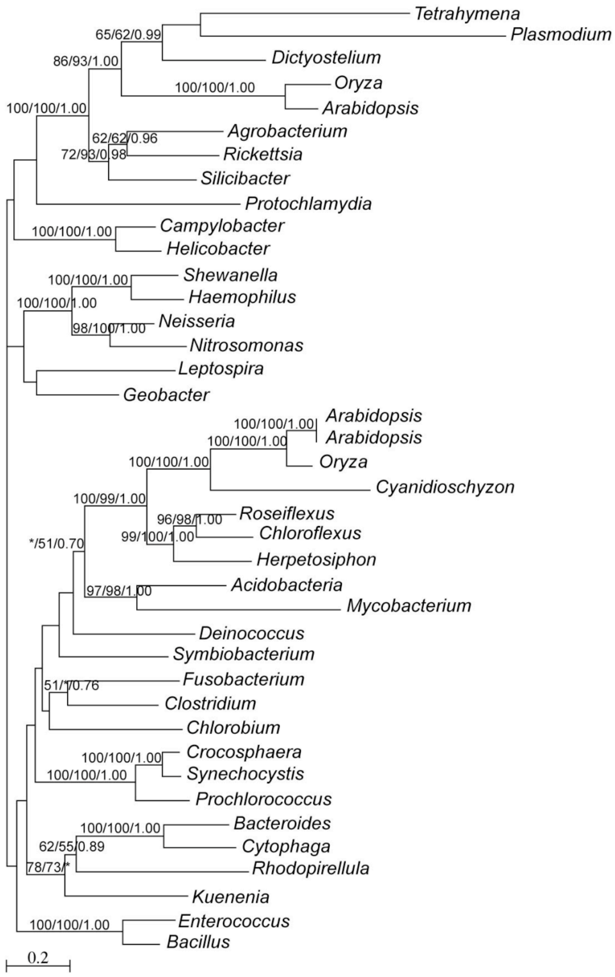

Figure 19. Molecular phylogeny of GTP binding protein, typA. P-value = 0.811 from AU test for the presented gene tree. AU tests were performed on alternative topologies including (**A**) monophyly of all eukaryotic sequences, (**B**) monophyly of red algal, green plant and cyanobacterial sequences, and (**C**) monophyly of red algal, green plant, cyanobacterial, and chloroflexi sequences. Topologies **A** and **B** investigate if red algae and green plants acquired the gene from mitochondria or plastids respectively, without invoking scenarios of secondary HGT. Topology **C** investigates if red algae and green plants acquired their genes from plastids and subsequently spread to chloroflexi through secondary HGT events. P-values < 0.001 from AU tests for topologies **A** and **B** whereas p-value = 0.195 for topology **C.** Therefore, the scenario of a plastidic origin in red algae and green plants and secondary HGT, although less parsimonious, cannot be confidently rejected.

---

20. Histidinol-phosphate transaminase (D)

CLUSTAL X (1.83.1) multiple sequence alignment

```
14601796_Aeropyrum_pernix          ALDLSSPSNPLGPPRYPDYEYLGFREAVAGFIDLERVIPLNGAAEILPLA
37522992_Gloeobacter_violac        LVDFSASINPLGPPHYPAPHGSDLQSALAARIEPERIVCGNGAAELLTWA
16330049_Synechocystis_sp.         LLDFSASINPLGPPHYPDPQYIHLRQAIADHLSMDWVLPGNGSAELLTLA
45507495_Anabaena_variab           IVDFSASISPLGPPHYPDPDYSELRLALGHFLPLEWILPGNGSAELLTLA
45655703_Leptospira_interr         ILDFSANINPLGFPSYPDPNYTSLKKKIHSKICTEQIVLGNGASELILQI
34762988_Fusobacterium_nuclea      ILDYSSNINPLGVPNYPDPYYIELRKKIAEFLNMDNIIVGNGATEILFLY
118725567_Clostridium_cellulo      LIDFSANINPLGLPNYPDPLCRELKKEISAYVPEEYLFCGNGAADVIYRI
13474816_Mesorhizobium_loti        WVKLNTNENPFPLPLYPEDDNISLREAAANAVSMDQVIAGNGSSELLGLV
57234598_Dehalococcoides_ethen     VMDFSVSSNPYPAPRYPDSDSAELKEYLAGRLKPENLIMGNGSMEIIRLV
15643798_Thermotoga_mariti         KTYLALNENPFPFPIYYDSPDEELIEKILSYLSKNNVSVGNGADEIIYVM
15807448_Deinococcus_radiod        PYKLDQNENPYDFPRYPDLHADTLRAAIAGYWDAAGVVITPGSNVLIKIL
53764865_Anabaena_variab           VIKLNSNENPYPPSRYPEPLGGEFREAASKVVPSDWLIVGNGSDEILSIV
16330453_Synechocystis_sp.         FIKLNTNENPYDPPLYPDPVSTQLRQAAADLVDLNQVLAGNGSDDILNIV
27381336_Bradyrhizobium_japoni     VVKLNTNENPYPPSLYPDPRATRLREAIAAYVAAEQVFVGNGSDEVLAHT
15599643_Pseudomonas_aerugi        LVKLNTNENPYGPSLYPDPNGERLKQAVAAHVQANQVFVGNGSDEVLAHI
27376508_Bradyrhizobium_japoni     VFKLSANETPFGPSDYPEGTSRVLREAIGRTLDPNRIICGAGSDEILNLL
45358779_Methanococcus_maripa      IIKLGSNENPWGCSQYPQPINPELMDEISKFMPVENIVGGDGADEVIDNI
118727390_Clostridium_cellulo      KIKLDANESPFTLPLYPDTDSVQLRQALGEYVDKENIIVGTGSDQLIQII
29609816_Streptomyces_avermi       PVRLNTNENPYPLPRYPDRDAVELRTELATYVGIENVWAANGSNEVIQQL
21674079_Chlorobium_tepidu         EVKLNQNENPFDLPRYPDILPYRGMAAYASFVKPELVIMSNGSNEMLYTI
37520892_Gloeobacter_violac        ADKLDANELPHDLPRYPEGDPLALKAAIAEYVTSEMVCVGNGSDELIRSL
45508256_Anabaena_variab           LDRLDTNESPCDLPRYPDGGHEELKDAIAQYITAANISVGNGSDELIRSL
15607044_Aquifex_aeolic            SVRLSSNEFPYDFPKYPDPEAKELKAVLADFVKEENLVLGNGSDELIYYL
48767779_Ralstonia_metall          LVKLDAMENPYRLPRYPVPSSEALRAKLKTVVPAGMVLLGNGSDEIISML
57234394_Dehalococcoides_ethen     IIKLDANENLYGAAIYPDATQFEIRRLLAEYVNMEQIICGAGSDQLIDLL
CM4361_Cyanidioschyzon_merolae     IIKLDANENPYGVCIYPDPESRELREALAAFVPASRLLVGHGADELIDLI
CPO00006602_Cyanophora_paradox     --------------------------------------------------
118046429_Chloroflexus_aggreg      IVKLDANENPYGPAIYPDPEQTALRSALASYRPIEEILCGAGADELIDLV
145342157_Ostreococcus_lucima      IVKLDANENPYGPPIYPDPESRRLRAALAAEVPTENLLVGCGADELIDLL
15217609_Arabidopsis_thalia        IVKLDANENPYGPPVYPDPQSRRLRDALAQDLESEYILVGCGADELIDLI
18416245_Arabidopsis_thalia        IVKLDANENPYGPPVYPDPQSRRLRDALAQDLESEYILVGCGADELIDLI
79327596_Arabidopsis_thalia        IVKLDANENPYGPPVYPDPQSRRLRDALAQDLESEYILVGCGADELIDLI
145334365_Arabidopsis_thalia       IVKLDANENPYGPPVYPDPQSRRLRDALAQDLESEYILVGCGADELIDLI
48855138_Cytophaga_hutchi          GIFLDANENPYGSARYPDPLQRDVKTALSALVSAEHIFFGNGSDEPIDLL
53714475_Bacteroides_fragil        SVFLDANENPYNLPRYPDPMQRDLKLELSKIVAPAHIFLGNGSDEAIDLV
32411469_Neurospora_crassa         NILLDANENAYGPPRYPDPHQRDLKQLLCSLLTPENLCVGVGSDEAIDAL
57223533_Cryptococcus_neofor       GILLDANENAIGPSRYPSPTHDELKREIAKLVPDENVFLGVGSDEVIDML
57506033_Campylobacter_upsali      VIKLASNENPLGVSLYPDDSMSELRNALALKLKMQNVIIGAGSDQIIELA
45658350_Leptospira_interr         IIKLGSNENPFGCAFYPDDSYLDLKTAIASKLTTDRIIPGNGSDQVLDFV
29607917_Streptomyces_avermi       AYKLSSNENPYPPLRYPDMACSGLMSELSERVPLSHLATGTGSVGVAQQL
13475919_Mesorhizobium_loti        IAKLGSNENPLGPSLYPDPAGRELRQAIAAKFGEDQIILGNGSEDLLSVI
42522616_Bdellovibrio_bacter       VYKLASNENPLGPSLYPDPSHYELLQTLSKEFPTKQLAIGNGSDELIDLL
16079319_Bacillus_subtil           VVKLASNENPYGCSLYPDGYSAALRTRLSKHVSETSLIFGNGSDEIIQII
118046536_Chloroflexus_aggreg      PIRMAANENPLGPSRYPDAGGAALRYALATRLSPEHVMLGNGSDELIMLI
15598361_Pseudomonas_aerugi        IVKLASNENPLGASRYPDGNGFELKRKLAERVDAAQVTLGNGSNDILDLV
48771086_Ralstonia_metall          IVKLASNENPLGMPRYPDSNGFELKAKLSEKVPAEWLTLGNGSNDILELA

14601796_Aeropyrum_pernix          AIVVVEPNFGDHTILALSAGIPCHSVFSLEGSLIVLSNPNNPTGSAAARG
37522992_Gloeobacter_violac        GTVLPIPAFGDYRRALAAFDAPYISVFSLPGDAVMLANPHNPSGYLYSPT
16330049_Synechocystis_sp.         GTYVVGPAFGDYGRSWRGAGAELKTIQPWPHHGLILNNPHNPSGTLLARE
45507495_Anabaena_variab           GTVLITPAFGDYYRTLRAYHARVLEFRVLGNYALLLNNPHNPTGKLFTRE
45655703_Leptospira_interr         PALIAVPCYSGYKEAISLLKIPCIEVFRLDKALVFLGHPNNPTGVTLDKI
34762988_Fusobacterium_nuclea      IVLILAPCFAEYERALKSVSAKIEYFFYPNYDLLLFCNPNNPTGQFIKLE
118725567_Clostridium_cellulo      ATLLTAPTFSEYEEAVKVFDSNITYHFNIDIRLMFLCNPNNPTGILTEKE
13474816_Mesorhizobium_loti        YVAMMSPGFSFNRKLATLQGAQFVEIHSLPAKFILLANPNNPTGTFVPVA
57234598_Dehalococcoides_ethen     AVLILKPTFGEYELAAEVAGADIIEQFKFDPKAVFICNPNNPTGVYLSKA
15643798_Thermotoga_mariti         MSVFFPPTYSCYRIFAKAVGAKFLEVLRIPGDVVFIPNPNNPTGHVFERE
15807448_Deinococcus_radiod        TVLTTDPTFSVYTLEAAMLGAELVLTFSLPPGVFYVTQPHAPTGHSDRPE
53764865_Anabaena_variab           IVVYPMPTYVLYRTLTQMQAADILEINVLPGAVTFIASPNSPSGHIVPND
16330453_Synechocystis_sp.         VVAFLDLTYSLYETIASVHGAKVQKIFDLTAKLIFLASPNPPKGKHLNRE
27381336_Bradyrhizobium_japoni     FLLFPDVTYSFYPVYCRLYGVAHEEVMRIESSAILLCNPNAPTGIALPRD
15599643_Pseudomonas_aerugi        FLLFPDVTYSFYPVYCGLYGIAHEKIFRIRGGIIF-PNPNAPTGCLLPLD
27376508_Bradyrhizobium_japoni     AAISTTHGFLVYPIATMAVGARNVIALTCDTKLVWLANPNNPTGTYIPFD
45358779_Methanococcus_maripa      MVIIPIPTFTQYAISAKIHGANIKWAFKLDTKAIFLCTPNNPTGNVIPTE
118727390_Clostridium_cellulo      AVLYPTPSFGMYKDSCVIAGGRAVDYFSYSPKVIYICNPNNPTGNLMPQD
29609816_Streptomyces_avermi       LAIGFEPSYSMHALIARGTGTAWISGFTIDPDVVFITTPNNPTGTAVPPE
21674079_Chlorobium_tepidu         FVLIPEPSFSLYDKLARLQQAGVVEVLSFDVDFIVLSTPNNPTSKSLSHD
37520892_Gloeobacter_violac        IVASAEPTFSMYRILAETLAVPYIGVYGVDVRVLFLANPNSPTGNLLSDE
45508256_Anabaena_variab           LILVANPTFSMYGILAQTLGIPVVTVFEIDIRVVFVVHPNSPTANPLTTN
15607044_Aquifex_aeolic            SVYIPVPTFPMYEISAKVLGRPLVKVFDIDPVLGYFAYPNNPTGNLFSRG
48767779_Ralstonia_metall          SVMAPVPGFVMYAMSAQFAGLEFVGVFTLDPAIIYLAYPNNPTGNLFDAD
57234394_Dehalococcoides_ethen     LVINCPPTFAMYKFYTDLNRGTVVNVYDVNTKLIFIAAPNNPTGTAISKE
```

```
CM4361_Cyanidioschyzon_merolae      FVVNAPPTFGMYQFDADLNAAKVVSVFRFPPRIVFVTCPNNPDGSRISDS
CPO00006602_Cyanophora_paradox      --------------------------------------------------
118046429_Chloroflexus_aggreg       LIIDCPPTFGMYRFDAGICGGRVISVFSLDAKAIFLTAPNNPTGNPLPRT
145342157_Ostreococcus_lucima       LIINTPPTFGMYSFDCNINAGDVIDIFKIDPKLLFLTSPNNPDGSLIEED
15217609_Arabidopsis_thalia         MIIDCPPTFSMYVFDAAVNGAGVIKVFSLNPKCIFLTSPNNPDGSIISED
18416245_Arabidopsis_thalia         MIIDCPPTFSMYVFDAAVNGAGVIKVFSLNPKCIFLTSPNNPDGSIISED
79327596_Arabidopsis_thalia         MIIDCPPTFSMYVFDAAVNGAGVIKVFSLNPKCIFLTSPNNPDGSIISED
145334365_Arabidopsis_thalia        MIIDCPPTFSMYVFDAAVNGAGVIKVFSLNPKCIFLTSPNNPDGSIISED
48855138_Cytophaga_hutchi           IILILPPTYGMYEVSAGINDVDIISVFDLDTKIIFICSPNNPTGNLMSED
53714475_Bacteroides_fragil         FVVAIDPTYGMYQVCADVNDVEYRKVFQFSTKMIFLCSPNNPTGNDLLRS
32411469_Neurospora_crassa          LILVCPPTYGMYSVSAQVNDVGLVKVFAIDIKIIYLCSPGNPTGSLLAKE
57223533_Cryptococcus_neofor        YVITCPPTYGMYKVTANVNDIGVLEVFQLDVKLLFICSPGNPTGTLIPLD
57506033_Campylobacter_upsali       TFLQCGVSFAMYEIYAKQVGAKAYKTITHDIKILYLCLPNNPLGECLDAF
45658350_Leptospira_interr          CVLINRITFAMYKIYALQCGAKIHSTAFLDPKIIFLCTPSNPAGDALSKS
29607917_Streptomyces_avermi        LVIYAWRSFEAYPIITQVSGATSVQVDVHDTRLIFVCNPNNPTGTVVRRA
13475919_Mesorhizobium_loti         SVVTLYPSFPLHEDYATLMGASVKRVLTINPRMLLFSNPMNPVGSWLSAG
42522616_Bdellovibrio_bacter        CVLTSAAAFNAYEVSAPANRAVIHKVYRFDIRLIFVSNPNNPTGTYATKA
16079319_Bacillus_subtil            CTVTAAPTFPQYKHNAVIEGAEVREIGSHDTQVVWICSPNNPTGTYTSEG
118046536_Chloroflexus_aggreg       CAVLAQGSFVSYARRIQAQGAIARQIMTHDTRLMFVCNPNNPTGTTIGAA
15598361_Pseudomonas_aerugi         AAVFSEHAFAVYPIATQAVGAEGRAVWGHDTRVVFVANPNNPTGTWFGAD
48771086_Ralstonia_metall           AIVYAEYSFAVYALATQEVGARAIEVYGHDTKLVFIANPNNPTGTFVSAA


14601796_Aeropyrum_pernix           DLRELGALAVVVDESFADLS--GVIVARSLTKTLALQGLRIGFAYDDAAL
37522992_Gloeobacter_violac         ALAALRFKLLIFDEAFIDFLHPGLIVIRSLTKFYSLPGLRLGYAVLPVAM
16330049_Synechocystis_sp.          EILALHYQWVLLDEAFMDFLHPNLIILRSLTKFYRLPGLRLGYALAHPDL
45507495_Anabaena_variab            AILPLQFALVVVDEAFMDFVYPNLVILRSLTKFYSLPGLRLGYAIAHPDR
45655703_Leptospira_interr          EVLKIQNSVFVIDESFIHFCTENMILIQSMTKILALPGLRIGICYASPLI
34762988_Fusobacterium_nuclea       DIKEIKNTRIFIDEAFIEFI-KNIFIMRAFTKFFAIPGLRLGYGVFDDEI
118725567_Clostridium_cellulo       MVLRITNTILVVDECFMEFLYDNIIVLKAFTKIYAMAGLRLGYGISDENI
13474816_Mesorhizobium_loti         DIERLSDRLIVLDEAYVDFAYSNLLVLRTFSKSYAAAGVRVGFGFGHPEI
57234598_Dehalococcoides_ethen      DIEKVTDTLLVLDEAYIAFA-GNIIVIRSMTKDCALAGLRLGYGMASAEI
15643798_Thermotoga_mariti          EIERITGAFVALDEAYYEFHYENLAVIRTFSKAFSLAAQRVGYVVASEKF
15807448_Deinococcus_radiod         DVRRVLGWVTVIDEAYSQYAGKHVLSLRTFSKAWGLAGVRAGYLLTNPEL
53764865_Anabaena_variab            DLRKLLSGVLVIDEAYVDFAYENVILIRTLSKGYSLAGLRLGFGVGNPKL
16330453_Synechocystis_sp.          FLWQTAEGVVVIDEAYGDFSFDNVIISRTLSKSYSLAGMRVGLAIAAPAL
27381336_Bradyrhizobium_japoni      AVAALPDRLVVVDEAYVDFGHDNLLVIQTFSKSRALAGLRVGFAIGQRPL
15599643_Pseudomonas_aerugi         AIEAMPDSVVVVDEAYVDFGYPNLLVTQTLSKSRSLAGLRVGLAVGHADL
27376508_Bradyrhizobium_japoni      EVKRLSHVLLVLDAAYCDYVTENTVVHTHTFSKIHGLAALRIGWMFGPEHI
45358779_Methanococcus_maripa       DIKKITDALVMIDHAYIEYSYDNVLVLRTFSKVFGLAGQRVGYGVTSKKV
118727390_Clostridium_cellulo       EILRVTKSVVVVDEAYAEFSYENLLILRTFSKAFGLAGIRCGYSIASEKL
29609816_Streptomyces_avermi        TVLALKPSMVVVDEAYIEFSRPHLVVSRTMSKAFGAAGLRLGYLAAHPAV
21674079_Chlorobium_tepidu          EIERIADAIVLVDEAYVEFSYPNLIVLRTMSKALALAGMRIGFAIANPEL
37520892_Gloeobacter_violac         IIERLLPVLVVLDEAYYEFSRPNLVILRTFSKAFRLANFRVGYALANPEI
45508256_Anabaena_variab            ELRWLEQILVVVDEAYFEFSRPNWVILRTFSKAFRLAAMRVGYCVAHPEA
15607044_Aquifex_aeolic             KIEEIRGVFCVIDEAYYHYSREDTVVLRTLSKI-GMASLRVGILIGKGEI
48767779_Ralstonia_metall           DMAAICQSLVVVDEAYQPFAFGNLLVMRTVSK-LGLAGIRLGYVAGAPEW
57234394_Dehalococcoides_ethen      DIRQILGVPTVIDEAYYEFTYPNLMILRTFSKWAGLAGLRVGYGLFPPVI
CM4361_Cyanidioschyzon_merolae      DLRRLLPTLVVLDEAYIEFAYQNLIVLRTFSKWAALAGLRVGYGAFPEAL
CPO00006602_Cyanophora_paradox      --------------------HENLVVLRTFSKMAGLAGMRVGYGAFPLHV
118046429_Chloroflexus_aggreg       DLLRILPILVVVDEAYVEFAYPNLVILRTFSKWAGLAGLRVGYGLFPRWL
145342157_Ostreococcus_lucima       DLLRLLPILVVLDEAYIEFARKNLIVLRTFSKRAALAGLRVGYGAFPSDL
15217609_Arabidopsis_thalia         DLLKIMPILVVLDEAYIEFSYENLIVLRTFSKRAGLAGLRVGYGAFPLSI
18416245_Arabidopsis_thalia         DLLKIMPILVVLDEAYIEFSYENLIVLRTFSKRAGLAGLRVGYGAFPLSI
79327596_Arabidopsis_thalia         DLLKIMPILVVLDEAYIEFSYENLIVLRTFSKRAGLAGLRVGYGAFPLSI
145334365_Arabidopsis_thalia        DLLKIMPILVVLDEAYIEFSYENLIVLRTFSKRAGLAGLRVGYGAFPLSI
48855138_Cytophaga_hutchi           KVKRIFSGIVVVDEAYIDFAYPNMVVLQTFSKAWGMAALRLGTAFASKEI
53714475_Bacteroides_fragil         EIIKVFEGLVILDEAYNDFSYPNLIIILQTFSKAFGCAAIRLGMAFASEGI
32411469_Neurospora_crassa          DIQKVWNGVVVVDEAYIDFAWPNLVVMQTLSKAFGLAGIRLGAAFTSPPI
57223533_Cryptococcus_neofor        VIKRIFKGVVVVDEAYVDFSYANICVSQTLSKSFGLAAIRLGYLLAPPPL
57506033_Campylobacter_upsali       SVEEFEECLIVIDAAYNEFAFPNVLYLGTFSKLYGLGGLRVGYGMADEEL
45658350_Leptospira_interr          DVYEFLDTLVVIDAAYMEFGFPNVFYTGTFSKVYGLGGMRIGYGIGNQEL
29607917_Streptomyces_avermi        ELERFSDVLVVLDEAYREFIRPNVCVLRTFSKAYGLAGLRVGFAIAHEPV
13475919_Mesorhizobium_loti         DLSKVDETLIVVDEAYAEYADQPWIVLRTFSKAFGLAGLRIGFGIGDPEL
42522616_Bdellovibrio_bacter        EVEAFDDVMIIFDEAYNEFVYKNLIVLRTFSKIYGLAGFRLGAMIAPPEV
16079319_Bacillus_subtil            ELLAFSRVLVVLDEAYYEYVYSNLMILRTFSKAYGLAALRVGYGIADENL
118046536_Chloroflexus_aggreg       EMAAFDDVLVVVDEAYIEFVRDNLLLLRTFAKIHGLAGLRLGYAFGAPDL
15598361_Pseudomonas_aerugi         ALERFAEVLVVLDEAYIEYAHPNLLVSRTFSKAYGLASLRVGYALSSKAV
48771086_Ralstonia_metall           EIEAFPHVVVVLDEAYNEYLYPNLMVSRTFSKAYGLAGLRVGYGVAQPQL
                                                                 :.:*       * *

14601796_Aeropyrum_pernix           AARLDMARQPWNIAALLVERLLGDMEVKGHIARAIEAIEGLKPRLYSAVA
37522992_Gloeobacter_violac         AARWQAWRDPWPVNALAQHLGVAALADLDFERRTRAWLGPARSRLAAGLL
```

```
16330049_Synechocystis_sp.         LAQWQAWRDPWPVNVLAEAAAIASLEAEEFRQLVWQWLPPARSALQQDLL
45507495_Anabaena_variab           LQKWQLWRDPWPVNTLAAAAAIAALEDREFQVQTWQWLPPAREELFQGLL
45655703_Leptospira_interr         CSNISKRLPTWNVNSIAASVYEKAISDEDYIENSKQNIKIWKEKLIYDLL
34762988_Fusobacterium_nuclea      LKKMWEEKEPWTVNSFANLAGLVMLDDKEYIEKSEKWILEEKKFMYKELI
118725567_Clostridium_cellulo      IEDLHRAGQPWNVSVAAQKCGMAALKEIDYVNRTRKLIKYNRIFLQNSLF
13474816_Mesorhizobium_loti        IGRLRNIQNVFNMNVIGQAVGISVLAHRAAYADNHRHIRHERRRVTLALF
57234598_Dehalococcoides_ethen     ITNLKKVCPPWNVNSAAQKAGLVCLCHPSYLAESEKKIKASKEYLRQGFF
15643798_Thermotoga_mariti         IDAYNRVRLPFNVSYVSQMFAKVALDHREIFEERTKFIVEERERMKSALY
15807448_Deinococcus_radiod        AGHLQKLVSAFTINFLTQAVIETALEHPEYMRERVAEAIAERGRIYAAVC
53764865_Anabaena_variab           LDGLFKVKDSYNIDAIACKVAAVAITDQAYKNSCVAKVKASRTQLTKDLF
16330453_Synechocystis_sp.         IEEMDKVRDSYNLDRLAQVLGTAALRNQAEFVPLWEKVRHTRTRLMEQLF
27381336_Bradyrhizobium_japoni     IEALERVKDSFNVDCLAIAGAVAAIEDEAWFLESRTRIIASRDVLTRDLF
15599643_Pseudomonas_aerugi        VEALERIKNSFNLDRLAIAGAAAAFEDDAYFRRTCQAVIDSREALSASLF
27376508_Bradyrhizobium_japoni     IDAVNRIRGPFNVSTPAMYAAVAAIEDTAHQAMSKQFTETWRNWLTEEIL
45358779_Methanococcus_maripa      VDYMMRIKPIFSLTRASQVSAITALQDKEFFEKCLKEGIESREEIYNGLL
118727390_Clostridium_cellulo      TRAVNLTRPPYNISSLSQFTAALILSYKEEIQNNIQYLVEERERVLSKLL
29609816_Streptomyces_avermi       VDAVQLVRLPYHLSAITQATALAALEHTDTLLGYVEQLKAERDRLVAELY
21674079_Chlorobium_tepidu         LAEISKPKIPFASSRLAEITLMAVLENYSLVTDAVQYILAERGRIEAELI
37520892_Gloeobacter_violac        AAVLEKVRLPYNLPGLSQLAAFAALEHRDVLLAAIPEILAERRRIERFLL
45508256_Anabaena_variab           IAILEKVRLPYNLPSFSITSALVALQNRAILLESIPQILNERAKLITALL
15607044_Aquifex_aeolic            VSEINKVRLPFNVTYPSQVMAKVLLEGREFLMEKIQEVVKERERMYDEMV
48767779_Ralstonia_metall          LSQLDKVRPPYNVNVLTEATALFALEHVAVLDDQAAKLRAERSKLVEAMV
57234394_Dehalococcoides_ethen     ADYLSRIKDPYSVNIAADAAVRQTMLQREYMLETVKKIVDERKRLYTELL
CM4361_Cyanidioschyzon_merolae     LPHLWKIKQPYNVNVAGQVAALEALCQRDLLFQQVSWMKAERQRLYQILL
CPO00006602_Cyanophora_paradox     AEHLWKIKQPYNVTVASQLAAVASLQDLDLLLQNLSRMVAAREQLAAGLL
118046429_Chloroflexus_aggreg      SEQLWKIKQPYNVSVAAQAAAVASLNAMTELRRRVQAIVAERERLFARLL
145342157_Ostreococcus_lucima      IEYIWRVKQPYNVSAIAETAALAALSNPSYLQEVRDKIVFERARMFGLLL
15217609_Arabidopsis_thalia        IEYLWRAKQPYNVSVAGEVAALAALSNGKYLEDVRDALVRERERLFGLLL
18416245_Arabidopsis_thalia        IEYLWRAKQPYNVSVAGEVAALAALSNGKYLEDVRDALVRERERLFGLLL
79327596_Arabidopsis_thalia        IEYLWRAKQPYNVSVAGEVAALAALSNGKYLEDVRDALVRERERLFGLLL
145334365_Arabidopsis_thalia       IEYLWRAKQPYNVSVAGEVAALAALSNGKYLEDVRDALVRERERLFGLLL
48855138_Cytophaga_hutchi          VSVLNKIKPPYNINLLTQEAALEALQNVSVKDEMVYKILKQRDWLREELL
53714475_Bacteroides_fragil        IGVLNKIKYPYNVNQLTQQQAIEMLHKYYEIERWVKTLKEERGYLEEAFL
32411469_Neurospora_crassa         ARLLNSLKAPYNISSPTSALASYALDGLKVMRANKERLIKQRERLVAELG
57223533_Cryptococcus_neofor       VQILSNTKAPYNVSLPTASIALKAVEGVAAMSRSVATLNKNRQTLMDDLG
57506033_Campylobacter_upsali      INALYKLRAPFNVNILALKAATAALDDEEFVKNSLENNFSQMQEFEKFAL
45658350_Leptospira_interr         ISNLYKMRPPFSVTNLSALAATEALKNESYVESYLENNWNEMKRYEKFAI
29607917_Streptomyces_avermi       AAALRKTAVPFGVSQLAQDAAVASLRAEDELLGRVGALVCERQRVVASLW
13475919_Mesorhizobium_loti        RALLDRVRTPFNANGMAQAAALAALADEEHLAKVVALAKAERTQVESLLF
42522616_Bdellovibrio_bacter       VEVFNRVRKPFNVNDLAQVAANAALQDKEFIERSQQICWKGLDYFYKKLL
16079319_Bacillus_subtil           IRQIEPAREPFNTSRLGQAAAIAALYDQAFIASCVEQNNAGLQQYYDFAL
118046536_Chloroflexus_aggreg      IAYLERARPVFNVNALAQIAGLAALDDTDHLARSLAHANASRTRLTNALL
15598361_Pseudomonas_aerugi        ADVLNRVRQPFNVNSLALAAACAALDDHDYLAQSRRLNDSGMAQLEDGFL
48771086_Ralstonia_metall          TDLLNRVRQPFNVNSLAQAAAVAALDDTAFLQRSAELNRQGKARLVEAFL
                                             :              .

14601796_Aeropyrum_pernix           KPYSSQAPFILVRHRGVYIRLTPYHSRISVAPGERLERLQEAILD
37522992_Gloeobacter_violac        TVLPGAANFLLVQSARLLIRLGERYFRVAVRGESENDLLTGALAE
16330049_Synechocystis_sp.         FPLPSQTNFLLVKTTQILIRLGEDFFRVAVRTMADNQKLTQALAT
45507495_Anabaena_variab           TPQASAANFLLVETQQILIRLGDRFFRLAVRSHTDNQRLTKALKT
45655703_Leptospira_interr         NLFSSEANFILIKILKIAVRLSKNFIRIAVRTPEENKKIIDAFSN
34762988_Fusobacterium_nuclea      KAYKTECNFILIKLTNILIRLDYHFIRLAIKDRKLNLKMLEILAE
118725567_Clostridium_cellulo      EVINSKANYVLFRTEGILIRLDNKYFRIAVKSREDNEYLVNCIKK
13474816_Mesorhizobium_loti        SVIPSHANFLLARVPKILVAGLENYIRVSIGTKEQMDAFLRAASR
57234598_Dehalococcoides_ethen     RVLPSETNFFLLKVKGLMVRGLPQYVRIAPRTQSECERLLAVAAE
15643798_Thermotoga_mariti         RITDSRGNFVFVFMENVAVRSFREGVRITIGKREENDMILRELEV
15807448_Deinococcus_radiod        TIFPSNTNFFLLKTPGIVCRGLDGCLRIAVGTPAENDALIAAILA
53764865_Anabaena_variab           HVWDSHGNFLLTKPPKILIRGLEDKLRITVGTDEQNHILVRALRD
16330453_Synechocystis_sp.         QVCPSDANFVFAAPRKILVRRITDYLRITVGTDGEIDQLLLAIAS
27381336_Bradyrhizobium_japoni     EVLPSLANFVFARHRGVLVRRIEDFLRITVGTDEQCGRLIEVLRE
15599643_Pseudomonas_aerugi        EVLPSAANFVFARHPGVIVRRIDQFLRITIGSPEQNQALLDALHF
27376508_Bradyrhizobium_japoni     KVTPSVANFVLIHFPGLVLRGLPHSLRMTIGTEEANRLVVDGLRD
45358779_Methanococcus_maripa      EVYPTEANYMLVKVKGVIVRLEPYYFRVSIGTSEENERFLKIMSE
118727390_Clostridium_cellulo      KVYSSAANFILIKVQGIFVRLLSDCMRITIGTQEQNSILLDELYA
29609816_Streptomyces_avermi       DVTESDANFVQFGRFGVLVRGVPGWLRVSAGTPEENDAFLDAVRE
21674079_Chlorobium_tepidu         HTFESDTNFLIIRVAGVLVRLMENCLRFNVGLREENDRLLELLKK
37520892_Gloeobacter_violac        ELFPSDANFLFVRPRGSLVRGAAGGLRVTVGTPEQNDRLIANVAA
45508256_Anabaena_variab           AVAESAANFIFLQLKGTLVRQISGGLRITIGTPEENIRTLNHIQT
15607044_Aquifex_aeolic            EVFPSKANFLLFRTPDVLVRGLQKCLRVSVGKPEENNKFLEALEE
48767779_Ralstonia_metall          TVFPSAANFVLVRVPKVLIKLLANCLRVTVSTPEENAQFLEAFKA
57234394_Dehalococcoides_ethen     KPYPSAANFILCKLLGILVRMMENCLRFSVGKPEDTDALLKALGE
CM4361_Cyanidioschyzon_merolae     HPYPSQSNFVLCRVRGILIRGLTDCIRISMGTPAQMDRLYEVLEA
CPO00006602_Cyanophora_paradox     EPFPSSSNFVLARVVGILIRGLADCVRFSVGTPEQISILLSALSD
```

```
118046429_Chloroflexus_aggreg       TPFPSVANFILCRVNGVLVRLLDGYIRISVGTPDQTDTLLATIAE
145342157_Ostreococcus_lucima       EPFTSESNFILCRVKGVVIREIADCIRISVGRPEDTDAVVSALAS
15217609_Arabidopsis_thalia         NPYPSYSNFILCEVTGVMVRELKGYVRVSAGKPEHTDVLMECLKQ
18416245_Arabidopsis_thalia         NPYPSYSNFILCEVTGVMVRELKGYVRVSAGKPEHTDVLMECLKQ
79327596_Arabidopsis_thalia         NPYPSYSNFILCEVTGVMVRELKGYVRVSAGKPEHTDVLMECLKQ
145334365_Arabidopsis_thalia        NPYPSYSNFILCEVTGVMVRELKGYVRVSAGKPEHTDVLMECLKQ
48855138_Cytophaga_hutchi           ELYPSDANFILMRTAIVITRLCEGCVRITVGTEAENKRLLDVLKT
53714475_Bacteroides_fragil         QVFPSNANFFLARVTGIIVRLCGNCLRVTVGTRAENAKLIGALKK
32411469_Neurospora_crassa          KLAGGESNFLLYEMLGVVVRGCEGCLRITVGTEEEVTRCLGALRE
57223533_Cryptococcus_neofor        GLLGGNANFVLCQIVGVVVRGCEGCLRITVGTKEECKQATEHIAA
57506033_Campylobacter_upsali       DFIPSYTNFITYFFKGIIVRDLNA-IRISIGKPYENTRFFEEFES
45658350_Leptospira_interr          EFIDSYANFITFFARGIILRDLNA-LRITIGRPEQNDLVLEALEK
29607917_Streptomyces_avermi        TVPETQANFVWLRLGGVVVRGV----RVTVGENEANDIFLKVTER
13475919_Mesorhizobium_loti         DVAPSRGNFLFFNCRGVIVKGFDSYVRVSIGSPAENDHFMAALSQ
42522616_Bdellovibrio_bacter        PYIPSQGNFVMFDTLGIIMRGFKTHLRLSVGRDHENEAAMVALAE
16079319_Bacillus_subtil            KCYPSQTNFVLIDFKGYIVRGFPTSLRITIGTKEQNEEILAILAE
118046536_Chloroflexus_aggreg       TVIPGETNFIAVAVHGVLVTGLPGWIRISFGTEEENDACIAALQA
15598361_Pseudomonas_aerugi         SWIPSKGNFIAVDLAGVIVRGMPTFLRVSIGLPEENDRFLQALGK
48771086_Ralstonia_metall           EYVKSSGNFVMVRVGGVIVRGLPQWLRVTIGLPEENDAFIAALEQ
                                            :.                *.
```

Note: Identifiable homologs of this gene are only found in bacteria, primary photosynthetic eukaryotes and fungi. Protein sequences of primary photosynthetic eukaryotes and chloroflexi have the highest percent identity and they also share many conserved residues. As expected, these sequences also form a well-supported group in phylogenetic analyses. Fungal sequences have the highest percent identity with those of CFB bacteria and form another group with them. It is likely that primary photosynthetic eukaryotes and fungi acquired this gene from chloroflexi and CFB bacteria, respectively. The *Arabidopsis* sequence (GenBank accession number NP_568226 and TAIR locus AT5G10330) is annotated to be a chloroplast precursor. Sequence of *Cyanophora paradoxa* was obtained from TBestDB.

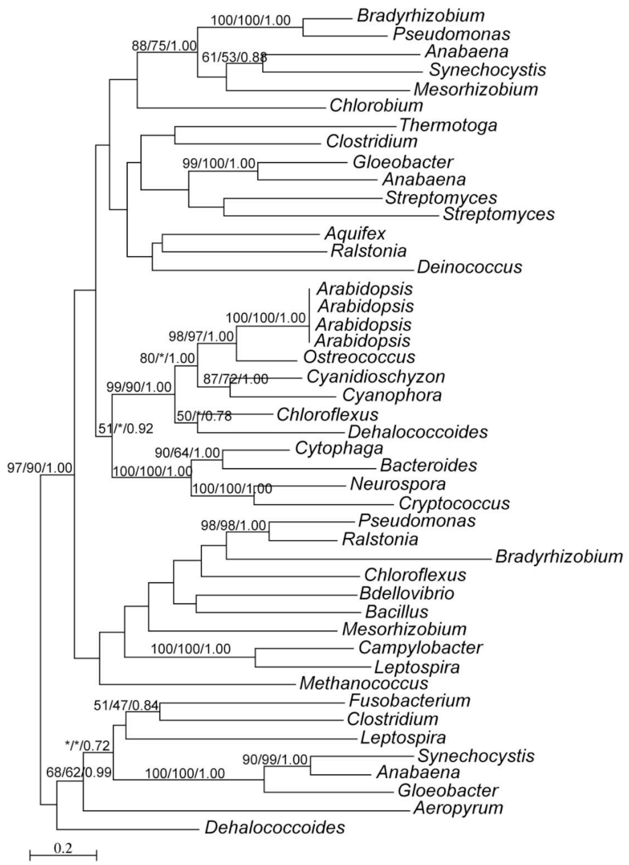

Figure 20. Molecular phylogeny of histidinol-phosphate transaminase. P-value = 0.999 from AU test for the presented tree. AU tests were also performed on alternative topologies including (**A**) monophyly of all eukaryotic sequences, and (**B**) monophyly of red algal, green plant, glaucophyte, and different versions of cyanobacterial sequences. P-values < 0.001 from AU tests for both alternative topologies.

---

21. tRNA methyltransferase (D)

```
CLUSTAL X (1.83.1) multiple sequence alignment


71030526_Theileria_parva            RVAVLVSGGVDSSLALWLMKFDVHAFYLKVVTLHVLPFSKAYSEHILSGV
34540110_Porphyromonas_gingiva      DVAALVSGGVDSSVVVHRLCYKPAIFYIRICPFEVVDLHKEYWERVVSYT
53712700_Bacteroides_fragil         DIAALLSGGVDSSVVVHLLCYKPTLFYIKILALEVVDLHREYWDNVAAYA
CM4720_Cyanidioschyzon_merolae      EVAVLLSGGVDSAVAMASLLFRVQPFYLKIVQLETLSLQEEYWSRVVQYT
116060467_Ostreococcus_tauri        RVAVLLSGGVDSSVALALLRHECRAFYLQIVELETVPLTDAYWNLVVSDS
15223816_Arabidopsis_thalia         RVAVLLSGGVDSSVALRLLHHSCTAFYLKIVPLEVVHLTDEYWERVVSYI
149197386_Lentisphaera_araneos      KIAMLLSGGVDSSVALRLLKHEITAFYLKIIPLQVVPLQKEYMDRVVSYV
2688619_Borrelia_burgdo             KIAVLLSGGVDSSVALYRIISNIKCYYLKIVPYEIINFQKEYYNKVVSYT
60464825_Dictyostelium_discoi       KVCIGMSGGVDSTITAKLLKFDVTGVFIKSIPMYKADFVKDYWNRVFVDF
116054971_Ostreococcus_tauri        SVVVGLSGGVDSAIAAWALKADVTAATTKNIPLTEIDFTREYWNDVFEPY
15612319_Helicobacter_pylori        KIAVLLSGGVDSSYSAYSLKHELVGIYLKLIPLEVLDFQKDFKSAVYDEF
15643286_Thermotoga_mariti          KVGVALSGGVDSAVALYLLLHEVKAFHMKTVEIEIVDVKEIFREKVIEPF
53711506_Bacteroides_fragil         RVLLGMSGGTDSSVAAMLLLYEVTGVTFRFIGHITYDARKVFQEQIIDYF
53715285_Bacteroides_fragil         RVLVGMSGGIDSTATCLMLQYEIVGVTMRVIEHYVADERVPFKDTIVKNF
15606458_Aquifex_aeolic             RVAVGMSGGVDSSVTALLLKHDVIRVTLRFIPHLVFSWEEIFKSKVIDYF
24214187_Leptospira_interr          KIIVAMSGGVDSAVTAGLLMYEVIGVNLRTIPFYVIKMEKVFQEKVIDRF
34763143_Fusobacterium_nuclea       KVGVAMSGGVDSSTVAYLLKYDIFGVTMKTIEHYVLDVRNEFKEKVMDYF
42526204_Treponema_dentic           KVLVGLSGGVDSAVAAKLLIYDVTGVTMQLIKHIVYDMRETFKTEIIDYF
6459532_Deinococcus_radiod          RVLCAMSGGVDSSVTASLLKYQVIGAMMRFVPFYLLDYREPFQRHIVGPF
56752503_Synechococcus_elong        RVVVGLSGGVDSSVAAALLHYAVEGLTL--VPYHVVDSRDRFQEAIVDYV
67922387_Crocosphaera_watsonii      KVVVGLSGGVDSSVAAASLQYDVIGLTL--IAHHIVDTRDLFTENIIDYL
21674668_Chlorobium_tepidu          HVIIGLSGGVDSAVAACLLIYHVTGLNIRVFPVFTLNLSAKFARDVVGYF
68230476_Frankia_sp                 RVLAAMSGGVDSAVAAARAVHDVTGVHLALIPFYVWDLAERFETEVIDEF
15827913_Mycobacterium_leprae       KVLAAMSGGVDSSVAAARMVHDVVGVHLALIPFYVWDFAEKFQEDVIDNF
15604175_Rickettsia_prowaz          TIVVAMSGGVDSSAVAAMLHHNVIGITLQLIPHYVLDYENKFKESVIDNF
39934381_Rhodopseudomonas_palu      RVVVAMSGGVDSSATAALLKYDVVGITLQLIPHYVLDYESRFRESVIDSF
53795994_Chloroflexus_aurant        -----MSGGVDSSLAAALLLHQVTGVTMHLIPFYVFNYQREFRRHVIDYF
94969297_Acidobacteria_bacteri      TIAVAMSGGVDSSTVAAMLRYNLVGLTMQLIPYYVVNQEDRFERDVVKPF
39997664_Geobacter_sulfur           -----MSGGVDSSVAAALLKHEVIGISMQVIPFYVVNFEEEFRRLVIDDF
87306814_Blastopirellula_marin      -----MSGGVDSSVAAHLLLHEVIGVFMRHIPFYALNLQREFG-QIIDYF
149175981_Planctomyces_maris        RVVLAMSGGVDSSAAAHLLLYEVIGLFMRSIPFHALNFKDAFG-RIKDYF
34762114_Fusobacterium_nuclea       KVVIGMSGGVDSSVSAYLLKYEVIGVTLNQIIHKVVNIRKDFENIVIKYF
49651723_Yarrowia_lipoly            HVYVAMSAGVDSSVTAALMAGRVTGVFMRNIPAVNLSLEKEYWLEVFEPM
8134740_Homo_sapiens                HVVCALSGGVDSAVAALLLRYQVTGVFMKNIPFHQVSYVKEYWNDVFSDF
21357477_Drosophila_melano          NVVVGVSGGVDSAVSAHLLAFKVLGVFMRNVELRQVNYVREYWTAVFSQF
RAO00086770_Reclinomonas            SVAVGMSGGVDSAVSAYLLKYRVVGVFMRNIECRQVDFVREYWNNVFSEV
62184976_Chlamydophila_abortu       TVVVAMSGGVDSSVVAYLLKYRVLGIFMKNIPYYTVSFAREYRERVFSRF
HVO00042429_Hartmanella_vermif      SVVVGLSGGVDSSVSAYLLQYKVRGLYMRNIPYDIVSLEKEYWNLVFEPA
46447434_Protochlamydia_amoebo      TVVVGMSGGVDSSVSALLMKYNVIGLFMKNIPYYAVNFVENYRKQVFTQF
106895797_Clostridium_sp            KVVIGMSGGVDSSVAALLLKYDVVGIFMKNIPYYSVNFEKEYWDRVFTYF
52141111_Bacillus_cereus            RVVIGMSGGVDSSVAALLLKYDVIGIFMKNIPYYAVNFEKQYWDKVFTYF
15597822_Pseudomonas_aerugi         RVIVGMSGGVDSSVSALLLLYQVEGLFMKNIKLHTANFAAEYWDNVFEHF
50121369_Erwinia_carotovora         KVIVGMSGGVDSSVSAYLLQYHVEGLFMKNIELHTVNFAAEYWDNVFELF
38703944_Escherichia_coli           KVIVGMSGGVDSSVSAWLLQYQVEGLFMKNIELHTVNFAAEYWDNVFELF
                                         :*.* **:                            :   :

71030526_Theileria_parva            LDDYRKGEVPNPDVLCNSRIKFGEFFDYVASGHYATLLCLSNDTLKDQTY
34540110_Porphyromonas_gingiva      VETVRRGLTPNPDMMCNKLIKFGCFFDRIATGHYATTLSTAKDPVKDQTD
53712700_Bacteroides_fragil         IEKIRKGQTPNPDVMCNKLIKFGCFFDLTATGHYATTLGTAKDPIKDQTD
CM4720_Cyanidioschyzon_merolae      IDEARRGRTPNPDIMCNSRIKFGAFYALVASGHYARVLFCSPDIVKDQTY
116060467_Ostreococcus_tauri        VEEIRRGRTPNPDVLCNSRVKFGAFFDRVASGHYAAVLVMSGDAFKDQTY
15223816_Arabidopsis_thalia         IEEYRCGRTPNPDVLCNTRIKFGAFYDYVGSGHYAKVLELSQDMVKDQTY
149197386_Lentisphaera_araneos      LAELKQGRTPSPDILCNERIKFGAFYDKVATGHYAQVLLRAPDPVKDQTY
```

```
2688619_Borrelia_burgdo             IEELKNGNTPSPDIFCNQRIKFGAFYDLVVTGHYAKILKQAKDKIKDQSY
60464825_Dictyostelium_discoi       LKDYKNGLTPNPDVWCNREIKFDLFVDYIATGHYSNLLHRAIDKNKDQTF
116054971_Ostreococcus_tauri        VKAFASGATPNPDLECNRSVKFGALGDLLATGHYARILLRGVDETKDQSY
15612319_Helicobacter_pylori        INAYEEGQTPNPCALCNPLMKFGLACEKIATGHYARVIQEALDKTKDQSY
15643286_Thermotoga_mariti          KRDLLKGLTPNPCAHCNRFVKFGYLFDAFASGHYARIIKKGVDLKKDQSY
53711506_Bacteroides_fragil         IDEYMSGHTPVPCTLCNNQLKWPLLIFYLATGHYVRKIAPAEDVDKDQSF
53715285_Bacteroides_fragil         IDEYRQGRTPNPCVMCNPLFKFRMLCDWIATGHYSRLIVAGDDDKKDQSY
15606458_Aquifex_aeolic             VEEYKRGRTPNPCALCNREVKTGFFIDKLATGHYAKIIKRPKDRKRDQTY
24214187_Leptospira_interr          IEDYQHGKTPNPCVECNTFVKFGALIDKIATGHYARIIANGIDVGKNQAY
34763143_Fusobacterium_nuclea       VNEYMNGRTPNPCMVCNRHIKFGKMASFMATGHYTKLLSVGDDSNKDQVY
42526204_Treponema_dentic           VEEYKQGRTPNPCFICNSKIKFGLFFDKIATGHYAKILRQAEDAQKDQSY
6459532_Deinococcus_radiod          LEEYARGRTPNPCVNCNTKVKFDELCRYVATGHYVKRFHRGDDPRKDQTY
56752503_Synechococcus_elong        VQGYEAGITPLPCSQCNRAVKFGPMADAIATGHYARLLLRAVDRNKDQTY
67922387_Crocosphaera_watsonii      VSGYESGVTPLPCSQCNRAVKFGPMCDRIATGHYARILLRAVDRNKDQSY
21674668_Chlorobium_tepidu          HDDYLAGRTPNPCMVCNKAIKWFGLADLVATGHYARTLLKGVDPEKDQSY
68230476_Frankia_sp                 VADYSAGRTPNPCVRCNERIKFAAVFDAVVTGHHARLLRRSVDPAKDQSY
15827913_Mycobacterium_leprae       VSAYARGETPNPCVQCNQRIKFSALFDTVATGHYARLLRRAVDRGKDQSY
15604175_Rickettsia_prowaz          VDSYLHGETPLPCVQCNKSVKFRDLADKLATGHYVRKLHTGLDTTKDQSY
39934381_Rhodopseudomonas_palu      ADSYASGETPVPCIECNRSVKFRDLASALATGHYVSSLVCAADRDRDQSY
53795994_Chloroflexus_aurant        IRAYTHGLTPNPCVECNRMIKFRALFDAVATGHYARILWRAVDLEKDQSY
94969297_Acidobacteria_bacteri      VEDYLSGRTPIPCSLCNNHLKFDQLAEKIATGHYARNLKRPADRAKDQTY
39997664_Geobacter_sulfur           VDEYFRGRTPNPCVRCNQRVKFELLADFLATGHYARRLLRGLDPAKDQSY
87306814_Blastopirellula_marin      VDEYVAGRTPNPCVMCNNWIKFGKLAEYVATGHYARLLVRGVDSGKDQTY
149175981_Planctomyces_maris        ADEYLAGRTPNPCVMCNNWLKFGKLASYISTGHYAQLLIRGLDRSKDQSY
34762114_Fusobacterium_nuclea       LDGYKTGITPSPCIICDDEIKFKILAEYVATGHYTSVLKSVHSIIKDQSY
49651723_Yarrowia_lipoly            LERYRQGLTPNPDVSCNRYIKFGSLDWWLATGHYARVLFRPKHLAKDQSY
8134740_Homo_sapiens                LNEYEKGRTPNPDIVCNKHIKFSCFADAIATGHYARTLLQAADSFKDQTF
21357477_Drosophila_melano          LDDYQMGLTPNPDILCNRHIKFDLFYDAVATGHYARNLLIPADTFKDQTF
RAO00086770_Reclinomonas            LAGYARGITPNADVLCNTHIKFRSLFDWLATGHCCRTLLRGVDPNKDQSY
62184976_Chlamydophila_abortu       LKEYSQGYTPNPDVLCNREIKFDLLGDFLATGHYCRLLLRGKDPHKDQSY
HVO00042429_Hartmanella_vermif      LLDYQRGVTPNPDVACNREIKFKHFADFVATGHYCRM---GIDL------
46447434_Protochlamydia_amoebo      IADFQKGWTPNPDILCNREIKFKVFADFLATGHYCQNLVKGIDHNKDQTY
106895797_Clostridium_sp            LNEYKAGRTPNPDVMCNKEIKFKAFADYLATGHYAQVLLRGVDTNKDQTY
52141111_Bacillus_cereus            LDEYRAGRTPNPDVMCNKEIKFKAFADYVATGHYARVMLRGVDDNKDQTY
15597822_Pseudomonas_aerugi         LAEYKAGRTPNPDILCNREIKFKAFADLIATGHYVRRLLKGLDPNKDQSY
50121369_Erwinia_carotovora         LEEYKAGRTPNPDILCNKEIKFKAFADYIATGHYVRRLLRGMDGNKDQSY
38703944_Escherichia_coli           LAEYKAGRTPNPDILCNKEIKFKAFADYIATGHYVRRLLRGLDSNKDQSY
                                          * .* .   *:  .*         :**

71030526_Theileria_parva            FLSRLTQNQMSKLIFPIGHLTKSQVREFAKTVGLPTFDKKDSFGLCFLDI
34540110_Porphyromonas_gingiva      FLAQINFAQISKLMFPIGHLLKSEVRAIANAAGLPSAKRKDSQGICFIDY
53712700_Bacteroides_fragil         FLAQIDYLQVSKLLFPIGGLMKHEVREIALRAGLPSARRKDSQGICFINY
CM4720_Cyanidioschyzon_merolae      FLSQLSQQQLLKATFPIGDLTKSAVRRLARERYLPNCDRPDSQGICFLKF
116060467_Ostreococcus_tauri        FLANLTQEQLRAVSFPVGGMPKADIRSLATSAELPNAGRKDSQGICFVKF
15223816_Arabidopsis_thalia         FLSHLSQTQLKRLLFPLGCVKKDEVRKLATQFDLPNKDRKDSQGICFIKF
149197386_Lentisphaera_araneos      FLSSLSQAQLSRALFPIGSLMKSRVRELAEEYDLPNKVRKDSQGICFISY
2688619_Borrelia_burgdo             FLSHLSQKQMSKLYFPLGTLLKSEVRQIAKNINLPNKDRKDSQGICFIKY
60464825_Dictyostelium_discoi       FLCMTKGERLKQAIFPIGGFTKENIVSFAKTIPSKITSKKSSRGICFRPL
116054971_Ostreococcus_tauri        FLASVSGDALSKACFPLGEMLKSETREAARALGLPCAERRSSAGICFPER
15612319_Helicobacter_pylori        FLYALEHEVIAKLVFPLGDLLKKDIKPLALNAMLGTETYKESQEICFKSY
15643286_Thermotoga_mariti          FLARIEPWRIERLIFPNGIYTKEEIRKIAEEAGIHVAKKQESQDVCFGSI
53711506_Bacteroides_fragil         FLWGLRQEILQRMLLPMGGMTKSEARAYAAGRGFEKSKKKDSIGVCFLDY
53715285_Bacteroides_fragil         FLWRLGQDVLRRCIFPLGNYTKQTVRDYLHEKGYEASKEGESMEVCFGDY
15606458_Aquifex_aeolic             FLSLVRREDLELLTFPLGAYTKEEVREIAKRYGLEVAQKRDSQEVCF-SP
24214187_Leptospira_interr          YLYGLSQENLKNVIFPLGEMTKPEVRQIARRMGLPVADKSESQEICFNDY
34763143_Fusobacterium_nuclea       FLSQIEKDRLSKIIFPVGDLEKTKLRELAKQMGVRVYSKKDSQEICFGKL
42526204_Treponema_dentic           FLALLTQEQLSRSIFPLGDFTKEKVRSIAEDAGLINAHRPDSQDICFDDY
6459532_Deinococcus_radiod          FLWGTPRDALPYILFPVGELEKPQVREIAAERGLLTAQKPESQNICFGKV
56752503_Synechococcus_elong        FLYDLPQSVLQSVKFPLGELTKPETRQIAAELGLRTAEKPESQDLCLGSM
67922387_Crocosphaera_watsonii      FLYDLSQDILKGTIFPLGEQTKEETRRIAAEFDLKTASKPESQDLCLGSM
21674668_Chlorobium_tepidu          FLWMLTQAELAKTLFPLGGYTKAEVRELARSFGVHAAEKKESQEICFDDY
68230476_Frankia_sp                 VLGTLRPEQLAAARFPLGDSTKAQVREEAARRGLAVADKPDSHDICFGDT
15827913_Mycobacterium_leprae       VLAVLTAQQLRHAAFPIGDTLKRQVRAEAARRGLLVADKPDSHGICFGNT
15604175_Rickettsia_prowaz          FLFTITREQLEYLSFPLGGFTKYETRKLASKFGLDIADKPDSQDICFGNY
39934381_Rhodopseudomonas_palu      FLFATTREQLQFLRFPLGDMTKPQTRELARQFGLSVADKHDSQDICFGRY
53795994_Chloroflexus_aurant        MLHMLGQAELSRLIFPIGAYTKREVREMAAARGLPSADREESQDICFGDY
94969297_Acidobacteria_bacteri      FLFGLTQEQLSRTLFPLGHKTKPEVREDAKNHGLKLFEKPDSQEICFGDY
39997664_Geobacter_sulfur           FLFTLTQDQLARTLFPLGEMTKEQVRRLAAERGLRVAEKGESQEICFDDY
87306814_Blastopirellula_marin      VLFGIGREYLRRMLLPVGQFEKSRIRELAGQIGMRVADKKDSQEICFGKH
149175981_Planctomyces_maris        VLFGINRGLLDKIIFPVGGFVKPEIREMAGEAGLRTANKPDSQEICFNDY
34762114_Fusobacterium_nuclea       MLYRLSPDKLERLIFPLKSYSKQEIREIALKIGLEVYDKKDSQGVCFEGY
49651723_Yarrowia_lipoly            YLSSVNSDSLRNVMFPLSDYLKPEIREMAHAYKLHVASKPDSQGLCFRKF
8134740_Homo_sapiens                FLSQVSQDALRRTIFPLGGLTKEFVKKIAAENRLHHLQKKESMGMCFRNF
```

```
21357477_Drosophila_melano        FLAGISRKALQRTMFPLGDFQKSQVKDLAKKIGFQRAKKKESTGICFRNF
RAO00086770_Reclinomonas          FLCAAPQRGLARTLFPVGGLKRTQVRQLA---------------------
62184976_Chlamydophila_abortu     FLCGTHPESLKNVLFPLGDMTKREVRSIAAQAGLATAQKRDSTGICFRPF
HVO00042429_Hartmanella_vermif    --------------------------------------------------
46447434_Protochlamydia_amoebo    FLYTLNQQILQQVLFPVGGLEKSQVRDIARKHQLVTAEKKDSTGICFRDF
106895797_Clostridium_sp          FLNQLNQYQLSKAMFPVGHLQKKDLRQIALDAGLATASKKDSTGICFRNF
52141111_Bacillus_cereus          FLNQLSQEQLSKTMFPLGELKKPQIREMAKEAGLATAAKKDSTGICFRNF
15597822_Pseudomonas_aerugi       FLHAVGGEQIARSLFPVGELEKPEVRAIAEKHGLATAKKKDSTGICFRRF
50121369_Erwinia_carotovora       FLYTLSHEQIAQSLFPVGELEKPQVRKIAEELELATAKKKDSTGICFRKF
38703944_Escherichia_coli         FLYTLSHEQIAQSLFPVGELEKPQVRKIAEDLGLVTAKKKDSTGICFRKF

71030526_Theileria_parva          SQYLTKSGPIVEYENKVIGTHNGLYNFTIGQKKTINSTPKYVVKKDIATN
34540110_Porphyromonas_gingiva    NDFIERYGRIIELEGKVIGRHQGYWFHTIGQRKGLGGGPWFVVKKDIKRN
53712700_Bacteroides_fragil       NDFVRRFGAVIEFEGKKIGTHRGYWFHTIGQRKGLGGGPWFVVKKDIQDN
CM4720_Cyanidioschyzon_merolae    EQFLEHYGPIMEAEGRFLGTHRGFWFYTIGQRKGLAGGPWYVVGKDLENN
116060467_Ostreococcus_tauri      SEFIAEHGDIIEVEGKVLGKHRAYWFHTIGQRSGLFGGPWYVAGKDCVNN
15223816_Arabidopsis_thalia       SDFVCRHGIILEAEGDFLGNHRGFWFYTIGQRQGLRGGPWYVVEKDTKNN
149197386_Lentisphaera_araneos    PDFVRFHGDIIEIEDEKKGEHKGVWFHTIGQRSGLGGGPWYVVKKDLATN
2688619_Borrelia_burgdo           NEFIKYHGNIIEKEGKIIGIHNGYWFFTVGQRRGIKNGPWFVIEKDLEKN
60464825_Dictyostelium_discoi     PDFLSQYGDFFDISNSFIKKHKGSVCYTMGQKANIDSERYFIVRSDIERN
116054971_Ostreococcus_tauri      GRVCGRRGRAAAQGGVVHGRS------TRENRRP--AKPWFVIGKNVEEN
15612319_Helicobacter_pylori      IDTLKKHGVVKNLQGEVIGTHKGYMQYTIGKRKGFSLEPHFVVGIDAKKN
15643286_Thermotoga_mariti        ENFLKDEGDIITPEGEVVGRHFGYPLYTIGQRKGFKGRRYYVRGKIPEKN
53711506_Bacteroides_fragil       RSFLKKCGRFLDESGNFIAWHEGYPFYTIGQRRGLGNRAVFVKEIHPETN
53715285_Bacteroides_fragil       RDFLREQGWFVSSEGVKLGQHKGFPYYTIGQRKGLEGKPAYVLKINPQKN
15606458_Aquifex_aeolic           GEYLEGIGLIKHVSGKVLGEHEGVYRYTIGQRRGLGGKPVYVIDIDAKTN
24214187_Leptospira_interr        RKFLEKKGFFKLKDGRIVGKHKGRENFTIGQRKGLGKNPLYVISIE-DDG
34763143_Fusobacterium_nuclea     KQFLIENGNIVDKNGNILGKHKGFSFYTIGQRKGLGEEPLYVLAFDKKTN
42526204_Treponema_dentic         TRVINALGKFIDTMGNEIGRHKGLQYYTIGQRRGLAGYPVYVVKKDAKTN
6459532_Deinococcus_radiod        QDFVAEHGYIREIAGEVVGEHLGTQFYTLGQKKGLGHRVRHVVHLDPDSN
56752503_Synechococcus_elong      RAFLDRYGEIVDQSGRVLGKHTGIHHYTIGQRKGLGSEPLYVIAIDPIQN
67922387_Crocosphaera_watsonii     STFLDQYGEIVDLEGKVLGTHQGVHHYTIGQRRGLGPEPLYVVKLDAVMN
21674668_Chlorobium_tepidu        CAYLANAGEIVDQAGKVIGHHRGYPFYTIGQRRGLGGEPVYVTEIDAEHN
68230476_Frankia_sp               GAWLRERGPVVDAEGETLGEHDGAYAFTVGQRRGLGGRPRYVLEISPATS
15827913_Mycobacterium_leprae     RAFLSECGSVVDADGTVLAEHDGVHGFTVGQRKGLGGRPRYVTAIDADTA
15604175_Rickettsia_prowaz        KTVINKIGKIVHINGFELGEHSGIINYTIGQRRGLGNEPLYVVKIDPSNN
39934381_Rhodopseudomonas_palu    TDVVERLGDIVDVDGRVLGQHPGIVHFTVGQRRGLGRSPLYVLRLDAAQR
53795994_Chloroflexus_aurant      RNLLRIEGPIVDLEGREIGRHRGLPLYTVGQRRGLGGEPRYVVAIDPARN
94969297_Acidobacteria_bacteri    KKFLDAYGELVTSSGEVVGHHTGIHNFTVGQRKGLGGSPLYVIELRGDKR
39997664_Geobacter_sulfur         VRFLEEEGEIVDRKGNVLGRHGGTYRYTVGQRRGLGPTPLYVVGVDAERR
87306814_Blastopirellula_marin    DEFIKSRGEIVTTAGEVVGTHDGIEKYTIGQRKGLGGEPRFVVRIDAETN
149175981_Planctomyces_maris      FGFLNRYGEMVDTSGNVVGQHTGYENYTIGQRKRLGGTPRFVVIKIEPETN
34762114_Fusobacterium_nuclea     KKFLKENGNYIDKDGNILGQHEGYQLYTIGQRRGLGSKPVFVITEIKPQTN
49651723_Yarrowia_lipoly          RDFLSDYGNVITEEGEVVGQHNGLWNATVGQKSGICRGSWFVKEKNIDTN
8134740_Homo_sapiens              EHFLLQYGHFISIENKVLGTHKGWFLYTLGQRANIGREPWYVVEKDSVKG
21357477_Drosophila_melano        KDFIQEYGPFLDIDGAVVGHHEGIHQWTVGQRCRLSLQPYFVARKEAASN
RAO00086770_Reclinomonas          --------------------------------------------------
62184976_Chlamydophila_abortu     KSFLEQFGEIIDYDQKIVGNHEGAHYYTIGQRRGLDEKPCYVVGKDMEKN
HVO00042429_Hartmanella_vermif    --------------------------------------------------
46447434_Protochlamydia_amoebo    RSFLSQYGAFQTLQGKNVGKHMGTAYYTLGQRKGLGGEAWFVVGKDHERG
106895797_Clostridium_sp          KEFLSNYGKIISLDGEVKGKHDGLMYYTLGQRKGLGGEPWFVVDKDLEKN
52141111_Bacillus_cereus          KDFLSNYGVMQTLSGEVKGKHDGLMYYTIGQRHGLGGDPWFAVGKNLKEN
15597822_Pseudomonas_aerugi       TDFLKQYGDIETTEGKVIGRHSGLMYHTIGQRQGLGDDPWYVLGKDLQRN
50121369_Erwinia_carotovora       TDFLARYGPILSVDNKPMGQHQGLMYHTLGQRKGLGEDPWYVVDKDVANN
38703944_Escherichia_coli         REFLGRYGKIITVDGDEIGEHQGLMYHTLGQRKGLGEEPWYVVDKDVENN

71030526_Theileria_parva          TLYVSVKLRHTPNYSQCVFLKHPDTGISTGQYCVFYCIGSAKM
34540110_Porphyromonas_gingiva    IILVSFKIRHTPEFTRGLESEERIQGIAPGQYCVIYCYGSGMI
53712700_Bacteroides_fragil       IIYVSFKIRHTPEFIKGIISSEKLQGIAPGQFGVIYCFGSGEI
CM4720_Cyanidioschyzon_merolae    IVLVSVKIRHGPRSVSAIALDEPESGIAPGQFAVFYCLGGGVI
116060467_Ostreococcus_tauri      VVYVTVKVRHGENIYSCVTINQDDQGLAPGQYAVFYCLGCGKI
15223816_Arabidopsis_thalia       VVFVSCKVRHGPGFYSCVHLDEDDQGLAAGQFAAFYCIGSGVI
149197386_Lentisphaera_araneos    IVYISVKLRHGPQLVEAIKLAEKDKGIAPGQSCIFYCLGRGVI
2688619_Borrelia_burgdo           IIYISIKIRHGEKKYSCISLNKKDQGISPGQFAIFYCLGGAKI
60464825_Dictyostelium_discoi     IVYVCGICRHRGDVVNLVNLDQPLRSVASGQILCLFCFGGGVI
116054971_Ostreococcus_tauri      VVYVAAKARYASPLTPVATFDAPERAVTPGQALVLYCLGGAVI
15612319_Helicobacter_pylori      ELIVGIKARYRSVPTKAVEFKEPFYGVAKGQALVVYLLGGGVI
15643286_Thermotoga_mariti        VVVVSCRVRKKSEEAPAVSFEKKVFAVTPGQIAAFYLLGGAII
53711506_Bacteroides_fragil       EVVLAVKIRYRKQENHCIRLHEPLSAIAEGQAAAFYLLGGGII
53715285_Bacteroides_fragil       TVMLGVRIRYRSRPIPCVRFLAEASAIAPGQSAVFYVLGGAFI
15606458_Aquifex_aeolic           TLIVGAQIRYRHKPVPVVKFKEDVRGITPGQVVAFYLLGGII
```

```
24214187_Leptospira_interr         SVILGVQVRYRHIPIRCVHPLEDVRGVTPGQSAVFYLLLGGII
34763143_Fusobacterium_nuclea      NIIVGAKTRSRDILHKCVKFDNKVRAITPGQGIVFYVIAGGFI
42526204_Treponema_dentic          TVTVGVKTRYRQQKKKAVEFIEPEKAVAEGQAAVFYIIGGGII
6459532_Deinococcus_radiod         TVWVGVQVRYRTAPVKALEFAEPQFAVAPGQSAVLYLLGGGLI
56752503_Synechococcus_elong       RVVVGVQVRYRSAPVPCILFADPQFSITPGQAAVWYLLGGGLI
67922387_Crocosphaera_watsonii     RVIVGVQVRYRSKAVRVLQFNEPQFGITPGQAAVFYVLGGGII
21674668_Chlorobium_tepidu         RIHVGARIRYRDRQSACVSFREPKQGVACGQAVVFYVLGGGII
68230476_Frankia_sp                TVTVGAQVRAHGGVVPAVRLAEPVRGTAAGQAVVFYVLGGGRI
15827913_Mycobacterium_leprae      TVYVGVQIRAHGETAVAVRLCEPLRGVARGQTLVLYVLGSATI
15604175_Rickettsia_prowaz         IVYVGVKIRSTRPPCHAVKFLSKEKAVAPGQACVIYVLGGGWI
39934381_Rhodopseudomonas_palu     QVVVGVRVRSTRAPQPAVELVVGEEGVSPGQACVFYVLGGGFI
53795994_Chloroflexus_aurant       ALIVGVQVRAHAEPLPAVELERPQRAVSPGQAAVFYVLGGGWI
94969297_Acidobacteria_bacteri     QVVVGAKIRHKHEPAWAVTFDDPQRAVTPGQAVVLYVVGGGWI
39997664_Geobacter_sulfur          QVIVGCKIRYRHQPVPCVRFRQPEKSVTPGQAVVFYVLGGGWI
87306814_Blastopirellula_marin     QVVIGAQIRYNSDDAPAVTFDEPRRGVAPGQAVVCYVLGGGWI
149175981_Planctomyces_maris       RVVIGAQIRYQHKEADCVTFDDPEYGVAPGQAVVLYVLGGGWI
34762114_Fusobacterium_nuclea      EIVLGARPRFSSTGFYGFKYNEENTHNAKGQHIVFFVVGGGEI
49651723_Yarrowia_lipoly           ELVIVLTIQFRSLMEPVIRTAVPVRAVSPGQNLVLYVLGCGVI
8134740_Homo_sapiens               DVFVAFRFRHQMALVPCVTAVQAVRALATGQFAVFYCLGSGKI
21357477_Drosophila_melano         TIYVAFRFQHTKPLVDCVELDAPLRAITPGQYAVFYCLGSARI
RAO00086770_Reclinomonas           -------------------------------------------
62184976_Chlamydophila_abortu      IVYIVAKVRYRSPDEECVRFTSPVKAITPGQTIAFYCLGGGII
HVO00042429_Hartmanella_vermif     -------------------------------------------
46447434_Protochlamydia_amoebo     IVFVESKVRYRQNDQFCVTFDRPQRAVTPGQSIVFYCLGGGVI
106895797_Clostridium_sp           ILYVTAKFRYRQPDQGVVMFDEPQKAITPGQAVVFYCLGGATI
52141111_Bacillus_cereus           ILYVDAKFRYRQEDNKVILCDEPIRAITPGQAVVFYCLGGATI
15597822_Pseudomonas_aerugi        VLLVGAKVRYRQSDQDCAVFDEPQRAVTPGQSVVFYCLGGGVI
50121369_Erwinia_carotovora        ILYVAVKTRYRQADISCVRFDEPFAAVTPGQSAVFYCLGGGII
38703944_Escherichia_coli          ILIVAVKTRYRQTDIPCVIFDEPVAAVTPGQSAVFYCLGGGII
```

Note: This gene appears to be restricted to bacteria and eukaryotes. All top hits of GenBank searches are from *Borrelia*, Lentisphaeria, and CFB bacteria. Protein product of the *Arabidopsis* sequence (GenBank accession number NP_175542 and TAIR locus AT1G51310) is localized in both chloroplasts and cytoplasm. The major eukaryotic sequence group contains some mitochondrial precursors (e.g. *Homo* sequence) and is likely of mitochondrial origin, although it is not particularly related to alpha-proteobacterial homologs. Please also note that the green alga *Ostreococcus* contains two versions of this gene, one of which groups with the eukaryotic mitochondrial clade while the other version with sequences of red algae, *Theileria*, CFB bacteria and spirochaetes. Sequences of *Reclinomonas* and *Hartmanella* were obtained from TBestDB; these two sequences formed a group with *Homo*, *Dictyostelium*, and other eukaryotic sequences in preliminary phylogenetic analyses, but were excluded from detailed phylogenetic analyses because of their short length. *Theileria* is an apicomplexan parasite containing a plastid derived from an algal endosymbiont.

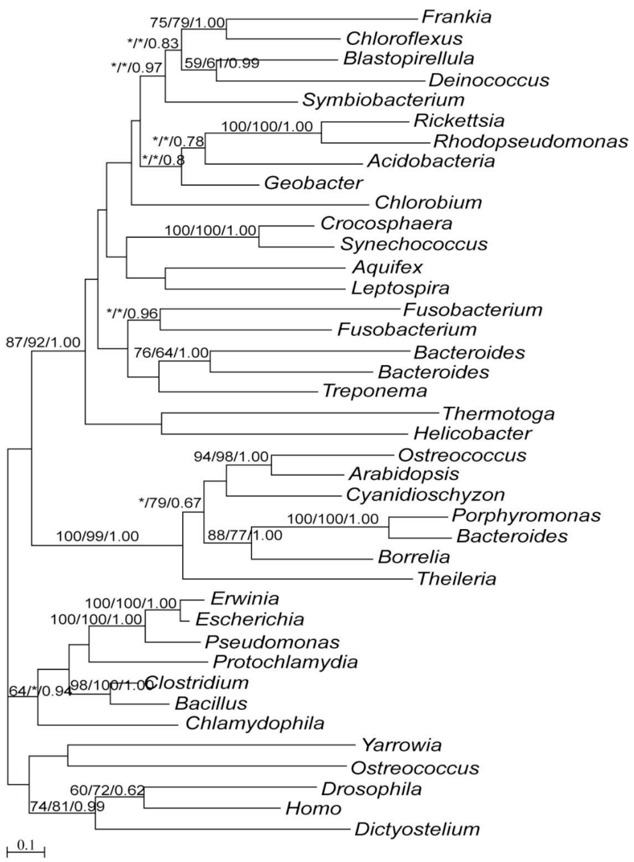

Figure 21. Molecular phylogeny of tRNA methyltransferase. P-value = 0.977 from AU test for the presented tree. AU tests were also performed on alternative topologies including (**A**) monophyly of all eukaryotic sequences, and (**B**) monophyly of cyanobacterial, red algal, and green plant sequences. These alternative topologies investigate if red algae and green plants acquired the genes from mitochondria or plastids respectively. P-values < 0.001 from AU tests for both alternative topologies.

---

22. Isolecucyl-tRNA synthetase (D)

```
CLUSTAL X (1.83.1) multiple sequence alignment


118193839_Cenarchaeum_symbio       VRSHLEGADL-DRMIVMFIEGPPTMNGPPHAGHLRGRVIKDLWYRYNTLR
6325217_Saccharomyces_mt           LVYKEQLRDFFEEFSFILHDGPPYANGELHLGHALNKILKDIINRYQLSQ
66800699_Dictyostelium_mt          LLK--DPYKLYKWQLWVFHDGPPYANGDLHMGHALNKILKDIVNRYKVLK
118751535_Marinomonas_sp.          MLKRWQDMDLYQKVRFILHDGPPYANGSIHIGHAVNKILKDIIVKSKTVS
46129096_Haemophilus_influe        MLKNWYEKNLYQKIRFILHDGPPYANGNIHIGHAVNKILKDIIIKSKTAL
75237181_Escherichia_coli          MLARWTDDDLYGIIRFILHDGPPYANGSIHIGHSVNKILKDIIIKSKGLS
47572014_Rubrivivax_gelati         WVKQWEQQGTYQRLRFVLHDGPPYANGQLHIGHALNKVLKDMIVKARQLA
1174519_Pseudomonas_fluorescen     ILQRWNSIGLYGKLRFVLHDGPPYANGTIHIGHALNKILKDMILRSKTLS
83594296_Rhodospirillum_rubrum     ILARWEKLDIYARLRFVLHDGPPYANGHLHNGHALNKILKDVITRSQQML
94968888_Acidobacteria_bacter      MLARWEEQRIYELIRYILHDGPPYANGPIHLGHALNKCLKDFVVKSKTMA
15644113_Thermotoga_maritima       FLEEWEKMDLYNYVLFVLHDGPPYANGHIHIGTALNKILKDIVVKYKTMR
15605834_Aquifex_aeolic            ILEKWK--GLYEKIQFVLHDGPPYANGHIHVGHALNKILKDVINKYNLLI
116747573_Syntrophobacter_fuma     ILEKWEAMGLYDMLRYILHDGPPYANGHIHLGTALNKILKDMIVKSQQMS
116057018_Ostreococcus_tauri       MQEWWRERGVYETLRFVLHDGPPYANGDLHIGHALNKILKDFVNRWEMMN
CM546_Cyanidioschyzon_merolae      IQRFWEENRIYEGLYFLLHDGPPYANGSLHMGHALNKILKDIINRYFILC
```

```
33239720_Prochlorococcus_marin      LQQFWKEKGIDFELGFTLHDGPPYANGNLHMGHALNKILKDIINKYQILK
22327660_Arabidopsis_cp             LQKLWEENQVFKRVSFILHDGPPYANGDLHMGHALNKILKDIINRYKLLQ
56751678_Synechococcus_elonga       IQQFWADRQIYETLSFVLHDGPPYANGALHMGHALNKTLKDIINKYQLLQ
113477079_Trichodesmium_erythr      LQKFWAQEQIYERLSFTLHDGPPYANGNLHIGHALNKILKDIINRYQILQ
67921387_Crocosphaera_watson        LQKFWADNQIYEKLSFVLHDGPPYANGSLHMGHALNKTLKDIINKYKLMR
34763705_Fusobacterium_nuclea       YIKKWTEEKIYEKGLFILHDGPPYANGNTHIGHALNKILKDIIVKYKTFR
47570310_Bacillus_cereus            MQEKWAEMNIYEKVQFVLHDGPPYANGDIHMGHALNKVLKDFIVRYKSMT
46852147_Homo_mt                    IQQKCGFSELYSWQRFCLHDGPPYANGDPHVGHALNKILKDIANRFHMMN
24665024_Drosophila_melano          RLNKIAVSYAYQEQRFVLHDGPPYANGQLHMGHAVNKILKDITLRQRVAH
149179295_Planctomyces_maris        ILKFWEVNQTFDQLRWSFIDGPMTANNPMGVHHAWGRAYKDAYQRYYAMT
18312756_Pyrobaculum_aeroph         VEEFWRRNRIFEKWKFTFLEGPPTTNGMPHVGHIRGRTYKDVVIRFYRLL
70606414_Sulfolobus_acidoc          VLKYWDDNNIYKKLKFLFIDGPPYPSSPPHIGTVWNKTIKDCILRYERLM
14600670_Aeropyrum_pernix           VKSYWEENSIYRKVKFYFLDGPPYASAKIHIGTAWNKVVKDVVLRFYRMT
84490249_Methanosphaera_stadtm      IQSFWQENDIYNKTSYSFLDGPPYCSGRIHLGTAWNKTIKDSFLRYKSMN
11498241_Archaeoglobus_fulgid       IFKFWEDNDIYRKVKFYFVDGPPYTTGRIHLGTAWNKVLKDTILRYKRMM
14521127_Pyrococcus_abyssi          VEKFWSENEIYRKVKYYFLDGPPYVSGAIHLGTAWNKIIKDMIIRFRTMQ
75401346_Pseudomonas_fluoresce      VLERWEKEKTFEQSIYVFYDGPPFATGLPHYGHILTSYIKDVIPRYQTML
102191610_Rickettsia_canade         ILKLWQDNNIFQKSIFIFYDGPPFANGLPHYGHLLTGFIKDVYARYQTIK
116670944_Arthrobacter_sp.          ILKYWDQDGTFQASIFVFYDGPPFANGLPHYGHLLTGYAKDLVGRYQTQR
15827608_Mycobacterium_leprae       VLDYWSNDDTFRASIYVFYDGPPFANGLPHYGHLLTGYVKDIVPRYRTMC
54023744_Nocardia_farcin            VLEAWAADDTFRASIFVFYDGPPFANGLPHYGHLLTGYVKDVIPRFQTMR
19074910_Encephalitozoon_cunic      VLEYWRRNKSFERSCFTFYDGPPFATGLPHYGHLLSGTIKDTVTRFFYQQ
51599084_Borrelia_garini            ILKFWNDNKIFEKSMFTFYDGPPFATGLPHFGHFVPNTIKDIIPRYQTMK
42528163_Treponema_dentic           VLKFWEKNDVFKKSVYIFFDGPPFATGLPHFGHFVPGTIKDIIPRYKTMK
46446128_Protochlamydia_amoeba      VLKFWQEGQLFERSVFTFYDGPPFATGLPHYGHILAGTIKDVVLRYKTMK
89898154_Chlamydophila_felis        ILDFWKTQNIFQKSLYSFYDGPPFATGLPHYGHLLAGTIKDVVGRFATMD
118749311_Marinomonas_sp.           VLTFWQDKQIFKRSLYVFYDGPPFATGLPHHGHLVASTIKDIIPRYFTMK
68130224_Leishmania_major           VLAMWHEKDCFRTSMFSFYDGPPFATGLPHYGHILAGTIKDMVTRFAYQT
CM4722_Cyanidioschyzon_merolae      VLSFWNDSRAFERSNFTFYDGPPFATGLPHYGHLLAGTIKDTVTRYAYQT
118380025_Tetrahymena_thermo        ILKFWDEINAFKQQLFTFYDGPPFATGLPHYGNLLAGTIKDVVCRYASQN
23619270_Plasmodium_falcip          ILKYWEDIDAFNLSNYIFYDGPPFATGLPHYGHLLAGIIKDCVTRYFYQS
71076452_Giardia_lambli             MLNYWDQIQAFETQLFNFYDGPPFATGLPHYGHLLAGTIKDVVCRYYSMN
42523714_Bdellovibrio_bacter        ILDFWDQEKIFAQSLYSFYDGPPFATGLPHYGHLLAGVLKDVVPRYWTMK
6319395_Saccharomyces_cerevi        VLSLWDEIDAFHTSLFSFFDGPPFATGTPHYGHILASTIKDIVPRYATMT
66816517_Dictyostelium_discoi       ILKYWDDIKAFETSVYSFYDGPPFATGLPHYGHILAGTIKDTITRYAHQT
24668543_Drosophila_melano          VLQKWRHENIFEKCSYTFYDGPPFATGLPHYGHILAGTIKDIVTRYAYQQ
94721239_Homo_sapien                ILEFWTEFNCFQECLFTFYDGPPFATGLPHYGHILAGTIKDIVTRYAHQS
116061521_Ostreococcus_tauri        VLKLWEEIDAFGQQLFVFYDGPPFATGLPHYGHLLAGTIKDIVTRFASTT
30681405_Arabidopsis_thalia         VLSFWTEIDAFKTQLYIFYDGPPFATGLPHYGHILAGTIKDIVTRYQTMT
67481173_Entamoeba_histol           VIEFWKKIDVFNKCNFSFYDGPPFATGLPHYGHLLAGTIKDTVCRYAIQT
57157197_Trichomonas_vagina         --------------------------GTPHYGHILAGTIKDVVTRYAYQT
48477483_Picrophilus_torrid         ILNYWKENHIDENIIFAFLEGPPTANGRPHVGHLMTRAVKDTVMRYKYMT
84029573_Thermoplasma_acidophi      ILKYWKDKNILEKILFVFLEGPPTANGRPHIGHAMTRTIKDIVLRYNTMT
60681847_Bacteroides_fragil         VLKKWDENQVFAKSMFVFFEGPPSANGMPGIHHVMARSIKDIFCRYKTMK
83815822_Salinibacter_ruber         VLDWWQDQNIFERSIFTFYEGPPTANGTPGIHHVLARAIKDIFCRYKTMQ
21673150_Chlorobium_tepidu          IREFWIERNIFRKSLYSFYEGPPTVNGKPGVHHLFSRTIKDVVCRYHTMQ
111221641_Frankia_alni              TLARWRDAKVFHRSLWVFYEGPPTANGKPGAHHVEARVFKDLFPRYRTMK
28210025_Clostridium_tetani         VLDFWNKNNIVDKSFFTFYDGPPTANGKPHVGHVLTRVIKDLIPRYKVMK
47570085_Bacillus_cereus            IRKQWNEQSIFEQSIFVFYEGPPTANGLPHVGHALGRTIKDVVARYKTMA
87311784_Blastopirellula_marin      VLDFWKSQKIYEKSLFVFYEGPPTANGMPHPGHCLTRAIKDVFPRYRTMK
94985204_Deinococcus_geothe         ILNFWQENRIFEQTQFVFYEGPPTANGRPALHHVLARSFKDLFPRYKVMQ
76261183_Chloroflexus_aurant        VIAWWEANNIVAKSLFVFYEGPPTSNGRPGLHHTISRSFKDVILRYRAMQ
                                                                           **   :

118193839_Cenarchaeum_symbio        GKRVIFNAGWDTQGLPVELQVQKELGVMGIEKLVAECKALVGKYQVQWKE
6325217_Saccharomyces_mt            GKYIFYKPGWDCHGLPIEIKALKDLSAISPLKIRSMALKHAQKAIKRQRE
66800699_Dictyostelium_mt           GFKVNYIPGWDCHGLPIEQQAFKKLKKMKASDIRKIAGDFARKEIEKQSK
118751535_Marinomonas_sp.           GFDAPYIPGWDCHGLPIEHKVEQLIGKVSYKEFRAKCREYAYTQIEEQKK
46129096_Haemophilus_influe         GFDSPYIPGWDCHGLPIELKVEGLVGKISAAEFRQKCREYASEQVEGQKK
75237181_Escherichia_coli           GYDSPYVPGWDCHGLPIELKVEQEYGKFTAAEFRAKCREYAATQVDGQRK
47572014_Rubrivivax_gelati          GYDALYVPGWDCHGLPIENQIEKLHGRLGRDEVQAKSRAYATEQIEQQRA
1174519_Pseudomonas_fluorescen      GFDAPYVPGWDCHGLPIEHKVEVTYGKLGADKTRELCRAYATEQIEGQKS
83594296_Rhodospirillum_rubrum      GKDANYVPGWDCHGLPIEWKIEERYRAIDVIEFRRECRQFAEEWIAIQRE
94968888_Acidobacteria_bacter       GFDAPYIPGWDCHGLPIEIKVDEQLGRMDPLDVRAACAKYALKYLDTQRE
15644113_Thermotoga_maritima        GYRAPYVPGWDTHGLPIEHRVSQELGEMSPAEIRKKCEEFALRFVDIQRE
15605834_Aquifex_aeolic             GKNVNFIPGWDCHGLPIERAVEKELSKLPKTEFRELCREYAKKYVNIQRE
116747573_Syntrophobacter_fuma      GKNAVYVPGWDCHGLPIEHQVDKELGKMSQVEIRRHCRKYAEKFIDIQRN
116057018_Ostreococcus_tauri        GKKVRFVPGWDCHGLPIELKVLQSMDALTPIKLRYKAKAFAEKTVKAQRE
CM546_Cyanidioschyzon_merolae       GKRVRFVPGWDCHGLPIELKAVQSARSLDPLQIRLLARKFALEAIEEQRK
33239720_Prochlorococcus_marin      GSKVRFVPGWDCHGLPIELQVLKTLDRLTPIKLRKKAAAYAHKQIALQKD
22327660_Arabidopsis_cp             NYKVQYVPGWDCHGLPIELKVLQSLDQLTPLKLRAKAAKFAKATVKTQME
56751678_Synechococcus_elonga       GRKVRYVPGWDCHGLPIELKVLQELSSLTPLTLRQKAKAYALAQVEQQSQ
113477079_Trichodesmium_erythr      GRKVRYVPGWDCHGLPIELKVIQNIKSLTPIELRRKARDFALETIDKQRE
```

```
67921387_Crocosphaera_watson        GHKVRYVPGWDCHGLPIELKVLQSMKSLTPLKLRRKASDFALKTQQEQAK
34763705_Fusobacterium_nuclea       GYKSPYVPGWDTHGLPIELQVVKEVGVMSALEIRQLCEKYARKWVGIQKE
47570310_Bacillus_cereus            GFCAPYVPGWDTHGLPIEQALTNK-GVMTVAEFRKLCAEYAYEQVERQRE
46852147_Homo_mt                    GSKIHFVPGWDCHGLPIEIKVLSELGRLSAMEIRKKARSFAKAAIEKQKS
24665024_Drosophila_melano          GQRVNYIPGWDCHGLPIELKATS----QSAQEIRQKSRAFAWEAIKSQKE
149179295_Planctomyces_maris        GHELRFQNGFDCQGLWVEVEVEKELGYHGIDKFVNECKKRVLRFAARQTE
18312756_Pyrobaculum_aeroph         GYDVWVQGGWDMQGMPVEWEVEKKLKLFGLEKFALECNSLVEEYLAYWRE
70606414_Sulfolobus_acidoc          GYSVKDQPGYDTHGLPIEVETEKRLGIVGVDNFISKCKEFAVNNSKSLTQ
14600670_Aeropyrum_pernix           GYNVWDKPGYDTHGLPIEVKIEQSLGVVGVENFIAACKRFVDENMEAMTR
84490249_Methanosphaera_stadtm      GYSLRRQAGWDTHGLPIEHKVEELLDIYGIDNFVEKCKEFAIKNKEDMTQ
11498241_Archaeoglobus_fulgid       GFAPTDTPGWDMHGLPIEVKVEQELGFFGIDKFIERCMNYALANKDAMTE
14521127_Pyrococcus_abyssi          GYNVWRQPGYDMHGLPIEVKVEQALGLIGVENFIKKCKEFALNNLKIMTE
75401346_Pseudomonas_fluoresce      GKQVPRRWGWDCHGLPVEFEVEKAMGFFGVEQFNDECRELVLKYADDWRG
102191610_Rickettsia_canade         GKKVERRFGWDCHGLPAEMQSEQELGIFSIEKFNSHCRASVMKYTGEWEQ
116670944_Arthrobacter_sp.          GRRVERRFGWDTHGLPAELEAMKQLGMMGIDKFNDACRASVMKYADEWKS
15827608_Mycobacterium_leprae       GYKVERRFGWDTHGLPAELEVERQLGIMGIAAFNEACRKSVLRYTEEWQA
54023744_Nocardia_farcin            GKRVDRRFGWDCHGLPAEIEAEKQLGIMGLAEFNAACKSSVLRYTGEWRD
19074910_Encephalitozoon_cunic      GYDVDRRFGWDCHGLPVEYEIDKKLGIMGIGKYNAECRGIVMKYSSEWEA
51599084_Borrelia_garini            GKYVKRNFGWDTHGLPVEYEVEKKLGIYGIENFNKECKKIVLRYTEEWKN
42528163_Treponema_dentic           GFRVERRFGWDCHGLPVENLIEKELGLYGIDKFNEACRASVLRYVKEWKQ
46446128_Protochlamydia_amoeba      GFCAPRRFGWDCHGLPVENEIEKTFGLFGIAKFNEECRNIVLRYTEEWKF
89898154_Chlamydophila_felis        GYYVPRRFGWDCHGVPVEYEVEKSLNLFGVAKFNEECRKIVFRYVDEWES
118749311_Marinomonas_sp.           GFHVERRFGWDCHGLPIEHEIDKSLGMLGIARYNAECRGIVQRYASEWEK
68130224_Leishmania_major           NHHVIRRFGWDCHGLPVEYEIDKMLGILGIDKYNDECKKIVTRYVGEWRK
CM4722_Cyanidioschyzon_merolae      GYQVPRRFGWDCHGLPVEYEIDQKLGILSIAAYNEECRKIVLRYSGEWER
118380025_Tetrahymena_thermo        GKYVERRFGWDCHGLPVEYEIDKKLGIMGVDKYNAECRSIVMRYAQEWRS
23619270_Plasmodium_falcip          NFYVERRFGWDCHGLPVEYEIEKENNIMGIDVYNEKCRGIVLKYSKEWVA
71076452_Giardia_lambli             RRYVNRRFGWDCHGLPVEYEVDKMLDVIGIRKYNQTCRSIVMRYSQEWEA
42523714_Bdellovibrio_bacter        GYTVPRRFGWDCHGLPVEYEINKTHKIMGVANYNDACRSIVKRYSTEWKT
6319395_Saccharomyces_cerevi        GHHVERRFGWDTHGVPIEHIIDKKLGIYGLENYNNECRSIVMTYASDWRK
66816517_Dictyostelium_discoi       GHHVERRFGWDCHGLPIEFEIDKLHGVMGIPTYNQHCRSIVMKYSHEWEI
24668543_Drosophila_melano          GYHVDRRFGWDCHGLPVEFEIDKLLNIMGIAAYNAECRKIVMRYADEWEN
94721239_Homo_sapien                GFHVDRRFGWDCHGLPVEYEIDKTLGIMGITEYNNQCRAIVMRYSAEWKS
116061521_Ostreococcus_tauri        GKHVVRRFGWDCHGLPVEHEIDKKLKILGIANYNEECRRIVLRYSAEWKK
30681405_Arabidopsis_thalia         GHHVTRRFGWDCHGLPVENEIDRKLNIMGIDKYNEECRSIVTRYVAEWEK
67481173_Entamoeba_histol           GHRVDRKFGWDTHGLPIEFEIDKLLGILGIPKYNQECRNIVMRYSEEWKK
57157197_Trichomonas_vagina         GHSVPRRFGWDTHGVPVEHEVDKMLNIMGIGVYNEKCRSIVMKHQQDWER
48477483_Picrophilus_torrid         GHDILRRTGWDCHGLPVELEAEKHFGFFGIEKFNQYCRDSVFRYIDEWNI
84029573_Thermoplasma_acidophi      DHKIYRRVGWDCHGLPVELEAEKHFGFFGVEKFNQYCRESIFRYIDEWKQ
60681847_Bacteroides_fragil         GYQVKRKAGWDTHGLPVELGVEKSLGIISVAEYNAHCRQDVMKFTKEWED
83815822_Salinibacter_ruber         GYQVDRKAGWDTHGLPVEIEVEEELGLYGIEKYNAACRGSVLEYKDLWDN
21673150_Chlorobium_tepidu          GYQVPRKAGWDTHGLPVEISVEKKLGLYGVGEFNREARALVYHHIDDWGK
111221641_Frankia_alni              GFHVPRRAGWDCHGLPVELAVEKELGFYGIAEFNARCRESVLRHVADFSA
28210025_Clostridium_tetani         GYKVLRKAGWDTHGLPVELEIEKKLGIFGVEEFIKECKDSVFTYSSMWKD
47570085_Bacillus_cereus            GYKVLRKAGWDTHGLPVELGVEKQLGIYGIEPFIKKCKESVFTYEKQWRE
87311784_Blastopirellula_marin      GYYCERKAGWDTHGLPVEVEVCKEMGIFGIEPFIHKCQASVWRYMQEWER
94985204_Deinococcus_geothe         GYFVTRKGGWDTHGLPVEISVEKKLGLEELEEFNRLCRASVWETIQEWNT
76261183_Chloroflexus_aurant        GYRIIRREGWDTHGLPVEIEIEKKLGFFGIAEFNRLCRESVWEYIQEWKA
                                            *:* :*:  *                  .

118193839_Cenarchaeum_symbio        ADRLLGVSLNQDKAYWTYRDDFIEREWQVLKKANERGILEVDFTVIAYCP
6325217_Saccharomyces_mt            TFQHFAILTDWETPYLTMDKDYEINQLNIFKEMYERGLIKRQNKPVYWGT
66800699_Dictyostelium_mt           GFQEMGILGDWENPYKTLDYGYEVEQIQTFYDMFNKGYIYRGVKPVHWSP
118751535_Marinomonas_sp.           DFIRLGVMGDWENPYLTMNFQTEANIVRALGKIAENGHLVKGFKPVYWSV
46129096_Haemophilus_influe         DFIRLGVLGDWDNPYLTMNFDTEANIIRTLGKVIENGHLYKGSKPVHWCL
75237181_Escherichia_coli           DFIRLGVLGDWSHPYLTMDFKTEANIIRALGKIIGNGHLHKGAKPVHWCV
47572014_Rubrivivax_gelati          DFKRLGVLGAWDQPYRTMDFGNEAGEIRALKRVMERGFVYRGLKPVYWCF
1174519_Pseudomonas_fluorescen      EFIRLGVLGEWDNPYKTMNFKNEAGEIRALAEIVKGGFVFKGLKPVNWCF
83594296_Rhodospirillum_rubrum      EFKRLGITGDWKRPYTTMAFAAEAQIVRELGKFLMAGDLYKGAKPVLWSV
94968888_Acidobacteria_bacter       QFKRLGVFGQWDKPYSTMTPEYESVVLRIFYDFLEQGAVYKGLRPVYWCI
15644113_Thermotoga_maritima        EFKRLGVRGDWENPYITLKPDYEVKILDVFKTLVEQGNVYRSLKPIYWCP
15605834_Aquifex_aeolic             DFVRLGVLGDWEHPYLTMDPKYEAQEIRELGKFFERGLAYRSKKPVYWCI
116747573_Syntrophobacter_fuma      EFRRLGVLGEWNNPYLTMSYDYEASIARELGRFFQEGGVIRSKKPIYWCT
116057018_Ostreococcus_tauri        QFKRYGVWADWEDPYMTLLPEYEAAQMEVFGSMFLNGHIYRGKKPVHWSP
CM546_Cyanidioschyzon_merolae       GFKRYGVWGTWNAPYLTLDHQYEAAQVRIFGEMFLKGYIYRGRKPVYWSP
33239720_Prochlorococcus_marin      GFRRWGIWADWDNPYLTLQKEYEAAQIKLFGEMAFKGYIYRGLKPVHWSP
22327660_Arabidopsis_cp             SFKRFGVWADWNNPYLTLDPEYEAAQIEVFGQMALKGYIYRGRKPVHWSP
56751678_Synechococcus_elonga       SFQRYGVWADWDAPYLTLTPEYEAAQIDVFGQMVLKGYIYRGLKPVHWSP
113477079_Trichodesmium_erythr      SFKRYGVWGDWEHPYMTLSPEYEAAQIGVFGKMVLKGYIYRGLKPVHWSP
67921387_Crocosphaera_watson        AFKRFGVWGNWEHPYLTLTPEYEAAQIGVFGEMALKGYIYRGLKPVHWSP
34763705_Fusobacterium_nuclea       QFIRLGVLGDWDNPYLTLDPRFEAKQLELFGEIYENGYIFKGLKPVYWSP
47570310_Bacillus_cereus            QFKRLGVRADWDNPYITLEPAYEAQQIKVFGDMAKKGYIYKGQKPVYWSP
46852147_Homo_mt                    AFIRWGIMADWNNCYYTFDGKYEAKQLRTFYQMYDKGLVYRSYKPVFWSP
```

```
24665024_Drosophila_melano          EFSSWGILANWDNIYMTFQPEFIVNQLEMFYNLYEKGLVYRDLKPVYWSP
149179295_Planctomyces_maris        QSVRLGYWMDWDNPYFTFSTENNETIWTFLKKCFERGKVYRGHDVMPWSG
18312756_Pyrobaculum_aeroph         GTKRLGLWLDLENAYETRQPHYLQYAWRIVKRAHELGLLTEDYRVLWFCP
70606414_Sulfolobus_acidoc          NFRNLGIFMDWENPYFTFNNDYISNSWAVIKKAYERGLLYKGVHVLHWCS
14600670_Aeropyrum_pernix           QFKEIGVFMDWENPYVTYRDEYIESGWWLVKKAWEKGLLYKGYRVLHWCP
84490249_Methanosphaera_stadtm      QFKQMGVWMDWQDPYVTYDNGYMESCWWTLKQADKRDLLTQDKRVITWCP
11498241_Archaeoglobus_fulgid       QFKSLAVWMDWENPYMTIKAEYMNAAWFAIKRAHERGLLERKKMVVNWCH
14521127_Pyrococcus_abyssi          QFKMLGIWMDWDNPYMTIKNEYIESAWFTLKRAWEKGLLEKDKRVLHWCP
75401346_Pseudomonas_fluoresce      FVNRMGRWVDFDGAYKTMDNDYMESVLWGFKTLHDKGHVYEGGKIVPYCV
102191610_Rickettsia_canade         YVTRQARWVDFKNSYKTMDTHFMESVLWAFKELYNKGLLYESMRVMPYSW
116670944_Arthrobacter_sp.          YVTRQARWVDFDNDYKTLNVEYMESVLWAFKQLHEKGLTYNGYRVLPYCW
15827608_Mycobacterium_leprae       YVTRQARWVDFDNDYKTLDLSYMESVIWAFKQLWDKGLAYESYRVLPYCW
54023744_Nocardia_farcin            YVTRQARWVDFDNDYKTLDLDFMESVMWAFKSLYDKGLIYQGFRVLPYSW
19074910_Encephalitozoon_cunic      VVERLGRWVSFRDGYRTMDMTFMESVWNIFKELFSRGLIYRGFRVMPFST
51599084_Borrelia_garini            IILRLGRWVDFEKGYKTMDISFMESVWWVFKNLYNKGLIYESYYVLPYSP
42528163_Treponema_dentic           TITRLGRWVDFENDYKTMEPAFMESIWWVMKSLWEKGLLYEGYYILPYCP
46446128_Protochlamydia_amoeba      TVNRMGRWVDFNQTYRTMDLPFMESVWWVFKQLYAKGLVYEGLKVMPFSA
89898154_Chlamydophila_felis        YVTRLGRWVDFASTWKTMDASFMETVWWVFRSLYDQGLVYEGVKVVPFST
118749311_Marinomonas_sp.           TITRLGRWVDFDNDYRTMEPWYMESVWWVFKQLWDKGLVYQGDKVIAYST
68130224_Leishmania_major           TVERVGRWIDFDDDYKTMHLTYMESVWWVFSQLWEKKMVYRGFRVMPYST
CM4722_Cyanidioschyzon_merolae      IVSRMGRWIDFKNGYRTMDTSFMESVWWVFSQLHKKGLVYRGFKVMPFST
118380025_Tetrahymena_thermo        IVNRFGRWVDFDNDYKTLDLKFMESVWWVFKQMFDKGLVYRGCKVMPYSN
23619270_Plasmodium_falcip          TVQRIGRWIDFKNDYKTMDKSFMESVWWVFSELYKNNYIYKSFKVMPYSC
71076452_Giardia_lambli             IVKRSGRWIDFKRDYKTMNLLFMESVWWVFKELYDKGLVYEGTKVMPYST
42523714_Bdellovibrio_bacter        TVRRVGRWVDMENPYFTMDVSFMQSVWWVFQQLFNKGLIYEGYKVVPYSV
6319395_Saccharomyces_cerevi        TIGRLGRWIDFDNDYKTMYPSFMESTWWAFKQLHEKGQVYRGFKVMPYST
66816517_Dictyostelium_discoi       VVNRMGRWIDMKNNYKTMDTPFMESVWWVFQELFKKDLVYQGFKVMPYSI
24668543_Drosophila_melano          VVTRVGRWIDFKNDYKTLYPWYMESIWWIFKQLFDKGLVYQGVKVMPYST
94721239_Homo_sapien                TVSRLGRWIDFDNDYKTLYPQFMESVWWVFKQLYDKGLVYRGVKVMPFST
116061521_Ostreococcus_tauri        TVTRIGRWIDFENDYKTMDPEFMESIWWVFKTLHEKNLVYKGFKVMPYST
30681405_Arabidopsis_thalia         VITRCGRWIDFKNDYKTMDLPFMESVWWVFSQLWEKNLVYRGFKVMPYST
67481173_Entamoeba_histol           VVWRCGRWIDMDHPYKTMDIDFMESVWWVFKQLYEKNLVYRGLKIMPFST
57157197_Trichomonas_vagina         VVRRCGRWIDFVNSYKSMDVTFMESVWWVFKQLWEQDNVYCGVKVMPYSY
48477483_Picrophilus_torrid         VDDLVGFWVDKENSYITLKNDYMESEWWALKTLYENNLLVKDYKIVPYCP
84029573_Thermoplasma_acidophi      VDDLIGFSIDHNGDYITLRNDYMESEWFALKTMYNSGLLYKDYTVVPYCP
60681847_Bacteroides_fragil         LTHKMGYWVDMKHPYITYDNRYIETLWWLLKQLYKKGLLYKGYTIQPYSP
83815822_Salinibacter_ruber         LTQRMGYWVDLDDPYVTFETDYIETVWWLLKQIEEEDLLYKGHKIQWYSP
21673150_Chlorobium_tepidu          LTERMGYWVDMDSPYITCDNNYIESVWWALKTIFDKGLIYKDYKIVPQDP
111221641_Frankia_alni              MTERMGYWVDLDSAYRTMDTSYIESVWWSLKQIFDKGLLVEDFRVTPYCP
28210025_Clostridium_tetani         MSEKLAFWVDMENPYVTYHNDYIESVWWALKQLWNKELLYKGHKVIPYCP
47570085_Bacillus_cereus            FTESIGYWVDMDDPYVTLENPYIESVWHILGTIHEKGLLYKGHRVSPYCP
87311784_Blastopirellula_marin      LTERLGFWLKLDEAYVTYHQSYVESVWWALKNFYDRGLLYQGHKIVWWWA
94985204_Deinococcus_geothe         FTQRLGYWVDLSDPYITYENEYVESVWNLLKRLHAKGLIAQDYKVVPLSP
76261183_Chloroflexus_aurant        FTKRIAFWLS-EDAYITYENDYIESTWWIFRQLWDRGLLFRDYKVTMHCP
                                         .        : :            .

118193839_Cenarchaeum_symbio        SCQASLSHAEVGYEEVSDPSLYYKVKLLIIWTTMPFTLVTDAMVGLHPDE
6325217_Saccharomyces_mt            ETRTALAEGELEYNEHKSIAAYVKFPLCLIWTSTPWTLLSNRAICFNQDF
66800699_Dictyostelium_mt           SSRTALADAELEYNNHTSKSIFVKFNVAIIWTTTPWTIPANQAICVNSEM
118751535_Marinomonas_sp.           VGGSALAEAEVEYQDKTSLSLDVRYAPVVIWTTTPWTLPASQAVSIHPEF
46129096_Haemophilus_influe         DCGSSLAEAEVEYEDKVSPSIYVRFPVAIIWTTTPWTMPSNRAIAVNADL
75237181_Escherichia_coli           DCRSALAEAEVEYYDKTSPSIDVAFQALVIWTTTPWTLPANRAISIAPDF
47572014_Rubrivivax_gelati          DCGSSLAEFEIEYENKSSPTVDVAFLAAVIWTTTPWTLPANQALNLNPEL
1174519_Pseudomonas_fluorescen      DCGSALAEAEVEYEDKKSSTIDVAFPIIVIWTTTPWTIPANQALNVHPEF
83594296_Rhodospirillum_rubrum      VEKTALADAEVEYQDHTSTTIWIRFPVVVIWTTTPWTMPGNRAVAYGPGM
94968888_Acidobacteria_bacter       HDKTALAEAEVEYEMHTSPSVYVRYMMAIIWTTTPWTLPASMAIAFSPNA
15644113_Thermotoga_maritima        RCRTALAEAEIEYHDHKSPSIYVKFRSIVIWTTTPWTLPANVGIALHPDY
15605834_Aquifex_aeolic             YDKTAEAEAEVEYYEKEDPSIYVKFPLAIIWTTTPWTLPANLGIMVKEDA
116747573_Syntrophobacter_fuma      SCKTALAEAEVEYHDHTSPSIYVKFPMVLIWTTTPWTLPANLAIAVHPDF
116057018_Ostreococcus_tauri        SSMTALAEAELEYPEHFSQSIYVAFPVLAIWTTTPWTMPANAAVAVNAKL
CM546_Cyanidioschyzon_merolae       SSRTALAEAELEYPDHVSRSAYVAFEVVAIWTTTPWTIPANRAVAVNPNL
33239720_Prochlorococcus_marin      SSRTALAEAELEYPEHTSPSIYVAFSVVAIWTTTPWTLPANMAVAVNENI
22327660_Arabidopsis_cp             SSRTALAEAELEYPEHISKSIYAIFKLLAVWTTTPWTMPANAAVAVNAKL
56751678_Synechococcus_elonga       SSRTALAEAELEYPDHTSRSIYVAMPILAIWTTTPWTIPANLAVAVNGEL
113477079_Trichodesmium_erythr      SSKTALAEAELEYPEHKSRSIYAAFKIVAIWTTTPWTIPGNLAVSVNPDL
67921387_Crocosphaera_watson        SSQTALAEAELEYPEHTSRSIFAAFPIVAIWTTTPWTLPGNLAVALNPDL
34763705_Fusobacterium_nuclea       ATETALAEAEIEYYDHVSPSIYVRMQAVLIWTTTPWTLPANVAICLNENF
47570310_Bacillus_cereus            TSESALAEAEIEYQDKKSASIYVAFPVFIIWTTTPWTLPANLGISVHPEL
46852147_Homo_mt                    SSRTALAEAELEYNPHVSRSIYVKFPLILVWTTQPWTIPANEAVCYMPES
24665024_Drosophila_melano          SSRTALAEAELEYDAHISPSVYVRFALALVWTTTPWTLPSNQAICYNASL
149179295_Planctomyces_maris        RGGSAYSQMEVGRKLSVHKSVFVRFPLLLIWTTTPWTLTSNVAAAINPEL
18312756_Pyrobaculum_aeroph         RCETSLSDHEVGYDEREDPSIYVKFRVLVIWTTTPWTIVDNEAVAVHPDY
70606414_Sulfolobus_acidoc          RCETTLADYEVEYRDLEDPSIYVKFRVLVIWTTTPWTLPANVFVMINKDF
```

```
14600670_Aeropyrum_pernix           RCETTLADYEVEYRELEDPSIYVKFPVLLVWTTTPWTLPANAFVMAHPDM
84490249_Methanosphaera_stadtm      HCQTALANAEIEYGDKQDPSIYVKFKLVLIWTTTPWTIPANLAVSIHPDF
11498241_Archaeoglobus_fulgid       RCETALADAEVEYWDEEDPSIYVKFPVIVIWTTTPWTLPANMAVAVHPSL
14521127_Pyrococcus_abyssi          RCETALAEHEVEYKLRKDPSIYVKFPVLLIWTTTPWTLPANLAVSAHPDY
75401346_Pseudomonas_fluoresce      RCQTVLSNFEAAFRPRRDMSAYVKFRQFLAWTTTPWTLPANVALAVAADE
102191610_Rickettsia_canade         ACETPLSHFETSYRERADKAVTVSFMLIFAWTTTPWTLPANLALAVGSDI
116670944_Arthrobacter_sp.          KDETPLSNHELVYKNRQDQTVTVTFPIALAWTTTPWTLPTNLALAVGPSI
15827608_Mycobacterium_leprae       RDETPLSNHELVYQSRQDPAVTLGFKVLLVWTTTPWTLPSNLAVAVHPDV
54023744_Nocardia_farcin            YEQTPLSNQETAYKMRQDPAVTVDMVLALIWTTTPWTLPSNLAIAVHPDV
19074910_Encephalitozoon_cunic      ACSTPLSNFESNYKDVSDPSVLIAFPLLVAWTTTPWTLPSNCGLAVNPGF
51599084_Borrelia_garini            KLATPLSNFEVEYKEVNDPSLTIKFKILLAWTTTPWTLPSNLGIAVGKEI
42528163_Treponema_dentic           RCSTVLSNHELGYKDVHDPAITVRFKTLLAWTTTPWTLPSNLGLAVGADI
46446128_Protochlamydia_amoeba      KLGTPLSNFEANYKEVDDPSLTVAFQSFLAWTTTPWTLVSNLALMVSPMI
89898154_Chlamydophila_felis        KLGTPLSNFEANYKEVDDPSVVIKFALLLVWTTTPWTLVSNMATAVGPEI
118749311_Marinomonas_sp.           ELETVLSNFEANYKEVQDPAVTVLFKLFAAWTTTPWTLPSNLALCVGPDI
68130224_Leishmania_major           ACTTPLSNFEANYKEVSDLAVTVAFQAFLAWTTTPWTLPSNLSLCVHPEL
CM4722_Cyanidioschyzon_merolae      ACSTPLSNFETNYREVQDPAITISFPVFLAWTTTPWTVPSNVALCVHPEL
118380025_Tetrahymena_thermo        GCATVLSNFETNYKEVDDPSLFIAFKTFIAWTTTPWTLPSNLALVINKDF
23619270_Plasmodium_falcip          KCNTPISNFELNYKDTPDPSIIISFILVFAWTTTPWTLPSNLALCVNEHF
71076452_Giardia_lambli             ACATPLSNFEANYKEVQDMTCIVSFPVLVAWTTTPWTLPSNLALCVNPSL
42523714_Bdellovibrio_bacter        GISTSLSNFEANYKMVQDPAITVMFKLIMAWTTTPWTLPSNLALAVGNDI
6319395_Saccharomyces_cerevi        GLTTPLSNFEANYKDVNDPAVTIGFNVLVAWTTTPWTLPSNLSLCVNADF
66816517_Dictyostelium_discoi       GCTTPLSNFEANYKDVSDPACVVSFQTILAWTTTPWTLPSNLALTVNPKM
24668543_Drosophila_melano          ACTTSLSNFEANYKEVVDPCVVVALEAFLVWTTTPWTLPSNFACCVHPTM
94721239_Homo_sapien                ACNTPLSNFESNYKDVQDPSVFVTFPLLVAWTTTPWTLPSNLAVCVNPEM
116061521_Ostreococcus_tauri        ACNTPLSNFEADYRDVSDPAVTVSFPILVAWTTTPWTLPSNMALCVNPEL
30681405_Arabidopsis_thalia         GCKTPLSNFEANYKEVPDPEIMVTFPVFVAWTTTPWTLPSNLALCVNAKF
67481173_Entamoeba_histol           GCCTPLSNFEAAYRDVPDPAVTMTFPVFLAWTTTPWTLPSNLALCVNPNM
57157197_Trichomonas_vagina         GCGTPLSNFEADYRSVSDPAIYIAFKVVIAWTTTPWALPSNLILVVNPKM
48477483_Picrophilus_torrid         RCGTSLSSHEVGYKNVDDPSVFVKFAEFIAWTTTPWTLPSNEFLVVNPDM
84029573_Thermoplasma_acidophi      RCETSLSSHEVGYKDVKDPSVYVRFKSFVAWTTTPWTLPSNEFLVVNPDM
60681847_Bacteroides_fragil         AAGTGLSSHELCYRDVKDTTVVAQFKMFLAWTTTPWTLPSNTALCVGPKI
83815822_Salinibacter_ruber         GSHTVLSSHEVGYEETQDPSVYIRFPVFLAWTTTPWTLISNTALAVGPEL
21673150_Chlorobium_tepidu          KSETVLSSHELGYKEVQDPSVYIKFRLILVWTTTPWTLISNVALAVGRDI
111221641_Frankia_alni              RDETPLSDHEVGYADVDDPSVYVRFPLLLVWTTTPWTLVSNTAVAAHPDV
28210025_Clostridium_tetani         RCGTALSSHEVGYKDVKEATAFVKFKVILAWTTTPWTLPSNVALAINKNF
47570085_Bacillus_cereus            SCQTSLSSHEVGYKTVKDLSATVKFKVFLGWTTTPWTLPANVALAVHPNM
87311784_Blastopirellula_marin      QGGTALSAGEVGYREVADPSVYVRFPLLLVWTTTPWTLPSNQFAAVHPEL
94985204_Deinococcus_geothe         RISTTLSRAELSYRMVDDPSVYVRFPVLVVWTTTPWTLPSNTLAAVNPDL
76261183_Chloroflexus_aurant        RCGTSLSDHEVGARDVDDPSVYIKFRVLVAWTTTPWTLPANVALAVKHDA
                                       :  :  *                    **: *:::  .

118193839_Cenarchaeum_symbio        DYNWIVGATRTAEFIEKSSKGSEFEGLKYVHPLHVAVSEEFVDASTGSGL
6325217_Saccharomyces_mt            SYSILVETGSIDKLTIKQFQGTHLNGLYYQNLLRPLLHGAHVTSGTGTGL
66800699_Dictyostelium_mt           DYIFIISKERLESLVILEFKGEQLKGTITKHPQSPIITGDHVIEGSGTGL
118751535_Marinomonas_sp.           NYALILAEDMVASVIVGRTVGAELAGTVLNHPFIPVILGEHVTTEAGTGC
46129096_Haemophilus_influe         EYNVILAAELVESVILGSVKGADLELSRFHHPFVPVILGDHVTTDGGTGL
75237181_Escherichia_coli           DYAVILAKDLVESVILGTVKGAELELLRFTHPFVPAILGDHVTLDAGTGA
47572014_Rubrivivax_gelati          EYALLLANALVEKCVLATTAGQALEGLEFHHPLSPVYLADYATAEDGTGI
1174519_Pseudomonas_fluorescen      TYALVLAEEMVESCVIATATGSALELINFRHPFSPVYLADYVELGSGTGI
83594296_Rhodospirillum_rubrum      DYVIAVCADLLDDVEIAHVKGADLAGTVCRHPWVPLLSGLFVTTEQGTGF
94968888_Acidobacteria_bacter       EYVYIVAGELAEATEIARFAGSKLERATFQHPFILGVLADYVTMDTGTGA
15644113_Thermotoga_maritima        EYSWVIATDLLDAFVVEKIKGKDLEGKEFVHPISRVILADYVSLETGTGC
15605834_Aquifex_aeolic             DYVWIVAKELMDKFVLETVKGKDLVGLEYTHPFWKIYPSEFVSLDTGTGL
116747573_Syntrophobacter_fuma      TYVWILARGLLESCVLRTFEAGELRGLACSHPFSVIVTGTHVTLEAGTGC
116057018_Ostreococcus_tauri        DYSLVVAEGLREAVTLGTFKGQDFENVQYSHPFPVIIGGEYITTETGTGL
CM546_Cyanidioschyzon_merolae       PYSLVVAQDLVETLNKKIVPGSELVGMTYRHPLYRVVGGDYITTESGTGL
33239720_Prochlorococcus_marin      DYILIIASDLREIVKVAIVKGKMLDGVLYKHPLPFVLGGNYITTESGTGI
22327660_Arabidopsis_cp             QYSVIVATDLVPALISKTFLGSDLENCRYTHPIPVVIGGDYITTESGTGL
56751678_Synechococcus_elonga       TYALIVAAELAESLVLATFPGSVLEHSRYRHPLPVVIGGDYITTESGTGL
113477079_Trichodesmium_erythr      NYAFIVAAEAVERLPVATVKGEDLENCTYQHPLKIVIGGDYVTTESGTGL
67921387_Crocosphaera_watson        NYALIVAADLVERLVKATLPGKALEHTIYRHPLEILIGGDYVTTESGTGL
34763705_Fusobacterium_nuclea       DYGLILAKDLAESALIKEFKGKDLEYTTYKHPFGLVILGDHVTADAGTGA
47570310_Bacillus_cereus            EYSYIIASELFETVVVKTVKGSELEYTVAKHPFSLVMLGDHVTTDAGTGC
46852147_Homo_mt                    KYAYVLAADKVASVTISTLSGVDLENGTCSHPLSPLLPANHVTMAKGTGL
24665024_Drosophila_melano          EYVYLMASALMADFIVQHLSGELLSKLSYNHPILPFFDASHVQDSKGTGL
149179295_Planctomyces_maris        DYVYYFAKQRLSREILETIKGAQMVGWEYTGPFRDFKGNPHVVAGEGTGI
18312756_Pyrobaculum_aeroph         VYAWWLAEALVPSLVVETKKGVELAGVRYIHPLHQVVTAEFVTLEQGTGL
70606414_Sulfolobus_acidoc          EYALVIAKDRVKELILRVYKGEELLGLEYEHPLKVLDGGEAVTLQEGTGL
14600670_Aeropyrum_pernix           EYVLILAKARLDHVVLEVLKGSQLEGLRYRHPLKVVMAPEAVTPHEGSGL
84490249_Methanosphaera_stadtm      DYSFIVADALIESIILKTLKGETLKGLEYIHPLHKIILGTHVTLEEGTGC
11498241_Archaeoglobus_fulgid       EYALILAKELADSVVVETYLGEDLEGLEYEHPLHQVFFADFVTAENT-GC
14521127_Pyrococcus_abyssi          DYVWILAKALVDKVVVEEFKGRELEGLRYVHILHRVILADFVTLEEGTGL
```

```
75401346_Pseudomonas_fluoresce      NYVLWLAEGCLGGLILERCTGAELAGLRYLPVVHRVVTADFVQMGDGSGI
102191610_Rickettsia_canade         DYAYIIAAASVSKYQFTIIKGSELEGLRYKPLFFKIFACDFVVEGDGTGV
116670944_Arthrobacter_sp.          TYAFLLAADLLGAYVTATYTGAELAGLAYQPLWWRFLVADYVTTTDGTGI
15827608_Mycobacterium_leprae       TYVFVLAQARLTAYVLATYRGADLLGRHYLPPFFQVLPGDFVTTDDGTGI
54023744_Nocardia_farcin            RYVYVLAAERVSHYVLAEFEGAALVGLSYRPPFHRVLAADYVTTDSGTGV
19074910_Encephalitozoon_cunic      LYGYLMHVDRIGEYILQRVSGRELEGLEYEQPFFRVLASGFVTDTDGTGV
51599084_Borrelia_garini            EYSLILGSKKINSYIIEKFKGSQLQGIEYEPIFFKVHTADYITTDDGTGI
42528163_Treponema_dentic           DYAYIMAVPRLEAYLIWTKKGAELEGLRYEPLFFRVLIGDFVTTEDGTGI
46446128_Protochlamydia_amoeba      EYAYILATERLKGYIVRKFPGSELEGQHYIPLFFKIILEDSISVEEGTGI
89898154_Chlamydophila_felis        TYVWILGQGCLERWVLESFPGTALVGKSYEPPFYKILSGSFVEESEGTGV
118749311_Marinomonas_sp.           PYVLWLAEAQLESMVLDRVLGADLSGKHYEPLFFQVFADDFVSTGSGTGI
68130224_Leishmania_major           DYVYWLAEARLGEVVVEKVKGAQLVGEKYVPLFFRVISGTYVTTDAGTGI
CM4722_Cyanidioschyzon_merolae      IYVWILAEARLGSIIIARHHGYELVGLHYEPVFFRVLGDDFVTADSGTGV
118380025_Tetrahymena_thermo        DYVYILAECRLPELILEKFKGSELVGREYEPLFFRILAGDFVTADAGTGI
23619270_Plasmodium_falcip          IYLFIFAECRLEWIIVNRFLGSELKELKYKPLFYKIIADDFVSDDAGTGI
71076452_Giardia_lambli             AYLYLVAESLVKTLVLHRLVGQDLAGLRYNPLFWKVVADDYVTSDSGVGI
42523714_Bdellovibrio_bacter        EYVLIMAQALLSSVVLQMMKGTELVGLTYEPLFFRIISSDHVTTESGTGV
6319395_Saccharomyces_cerevi        EYVFILLESLIKTLIVEKIKGSDLVGLKYEPLFFRVISDDYVTSDSGTGI
66816517_Dictyostelium_discoi       EYIFILGKNRTSILVLETMKGTDLIGKKYVPMFFVVIGGDFVTDDSGTGI
24668543_Drosophila_melano          TYVFVLAESRLSYVVKEKFVGKTLKDLHYKPLFYRVLVDEYVTEDSGTGI
94721239_Homo_sapien                QYVLILMEARLSALILERFPGAYLKGKKYRPLFFTVLVDNYVKEEEGTGV
116061521_Ostreococcus_tauri        KYVLVVAETRLESLIIKTVEGRALAGLRYEPIFFKVCADSYVTSDSGTGV
30681405_Arabidopsis_thalia         VYVYIVAESRLSALVLEKFNGASLVGKKYEPLFFRVVADDYVTDDSGTGI
67481173_Entamoeba_histol           DYVYYMMECLMPTLIIEKMKGKDLVGKTYVPLFFRVVADDYVQNDSGTGI
57157197_Trichomonas_vagina         KYAYITMKCRIPSLILEEMDGQDLVGIEYEPLFFKIYTADYVTDDSGSGI
48477483_Picrophilus_torrid         DYSYYLLSSKVESLLIKTFKGRDLEGIEYEQLMFRVVAASFVTAEDGTGI
84029573_Thermoplasma_acidophi      EYSYYVASSRAGYIEIRRMHGRDLVGKRYLQLMLKVVAGSFVTSEDGSGI
60681847_Bacteroides_fragil         DYVVVLAKALLNAHVIAEYKGPDLVGMEYEQLIFRVILGDYVTTEDGTGI
83815822_Salinibacter_ruber         TYVLILAEALLDDVVEETFSGEELIGQRYEPPYWYVLGADVVSTEEGTGV
21673150_Chlorobium_tepidu          DYVLILAESRLSVLVIDRCKGSDLEGRDYEPLFWYVVCGDFVSTGEGTGI
111221641_Frankia_alni              DYAFVVAEPLVAAAVVERFRGTALAGARYTRPFHSVVLAEYVTTTDGTGL
28210025_Clostridium_tetani         DYVLILAKELVDSVIIKEFKGEDILGLQYEQLLFRVIHGDFVTLSDGTGI
47570085_Bacillus_cereus            EYVYIVAKERVRDVVLSVHKGEELLNTSYTAPFYRVIGADFVTADSGTGL
87311784_Blastopirellula_marin      EYSLILATALVEPLTIATCLGAALLGKRYLPPFWRVVAADFVTTDSGTGV
94985204_Deinococcus_geothe         TYVVIVAADATRRLVLTSFPGRDLEGVEYEPPFHFVALADFVSAADGSGV
76261183_Chloroflexus_aurant        EYVLVMAAALVGNVVLRHFRGNDLVGLRYEPLFYRVIADEIVSLDDGTGI
                                     *                  .  :                       *

118193839_Cenarchaeum_symbio        VHLSPANGEEDIKIAIFCPIDDEVKFTPDRVIVEDLKLVRIGKLRHKYPL
6325217_Saccharomyces_mt            VHTAPGHGQDDYLIGIYSPVDHQGRYQLNKIILCKLSLYKSHEYTHSYPY
66800699_Dictyostelium_mt           VHTAPGHGVEDFQICVLSPVNDLGCFTDEEAVINDLELLHKEDYIHKYPY
118751535_Marinomonas_sp.           VHTAPDHGVDDFNVGTINLVQDNGVYSDADAVLEALNLVFESKIFHSYPH
46129096_Haemophilus_influe         VHTAPDHGLDDFIVGMAGLVSNDGKFISTPLVIEKLQLLKVEKIKHSYPH
75237181_Escherichia_coli           VHTAPGHGPDDYVIGTANPVGPDGTYLPGDIVIALLQLLHVEKMQHSYPC
47572014_Rubrivivax_gelati          VHSAPAYGVEDFNSCILNPVQGNGVYAAEPLIVQALQLMATAKLEHSYPH
1174519_Pseudomonas_fluorescen      VHCSPAYGVDDFVICIINPVQSNGVYVPSQPIIEKLRLMQTAAIQHSYMH
83594296_Rhodospirillum_rubrum      VHMAPGHGEDDYFLCPPDTVDGDGLYMDHPDIVAAMILLAQGSLTHSYPH
94968888_Acidobacteria_bacter       VHTAPAHGADDFYTGQTCNVDEAGRLRNGPVIVQLLRLLGFENIEHSYPH
15644113_Thermotoga_maritima        VHIAPGHGEEDYIYGIVSPVDEEGRFTEEVIIEDLKLVHASSITHSYPH
15605834_Aquifex_aeolic             VHMAPGHGQEDYVVGPYAPVSDEGRFVEPHLIVGVLKLVHEEKIRHSYPH
116747573_Syntrophobacter_fuma      VHTAPGHGREDYDMAVYSPVDDNGCFTDDRAINAKLELILEKSITHSYPH
116057018_Ostreococcus_tauri        VHTAPGHGQDDYISGLYSPVDNAGLFTAEVLCIEKLELLKREKYGHKYPY
CM546_Cyanidioschyzon_merolae       VHTAPGHGLEDYNVGVFAPVDDVGRFTSDARVLELLRLLLEEKYEHKYPY
33239720_Prochlorococcus_marin      VHTAPGHGVDDFNTGIICPVDEKGFFTSETKIINALKLLKEKPYIHKYPY
22327660_Arabidopsis_cp             VHTAPGHGQEDYATGLVSPVDDEGKFTEETAVVSYLDLVMEESYAHKYPY
56751678_Synechococcus_elonga       VHTAPGHGQDDFIVGVFCPVDDKGDFTAAAAVIEWLTLLKEESYAHRYPY
113477079_Trichodesmium_erythr      VHTAPGHGQEDYIVGILSPVDGNGKFTAEIAVIEALQLLKEEPYEHKYPY
67921387_Crocosphaera_watson        VHTAPGHGQEDYLVGVLSPVDAKGNFTEEEVIINELQLLKEEAYQHKYPY
34763705_Fusobacterium_nuclea       VHTAPGHGQEDYVVAVISPIDHRGYLTEEKAIIEYLTILKMQEINHSYPH
47570310_Bacillus_cereus            VHTAPGHGEDDFIVGVLCPVDDKGVLTEEKPITEKLELLKLTFITHSYPH
46852147_Homo_mt                    VHTAPAHGMEDYGVAMDCLVDEDGVFTDVDVVIKMLQLLKEEKLVHSYPY
24665024_Drosophila_melano          VHTAPAHGPEDFLVSVKCFVNEEGTYTKESLVLDRIAVVHSSKLEHSYPI
149179295_Planctomyces_maris        VHTAPGCGDVDHQLGAIAPLGEDGRFEEGELVFERLKLVSVESYPHIYPH
18312756_Pyrobaculum_aeroph         VHIAPGHGPEDFELAVTNSVEINGIYNELKEVTRDLRLVFEEKIRHEYPH
70606414_Sulfolobus_acidoc          VHSAPGHGDVDFEIGVVMLVNDKGEFTQDEEIISDLKLLHASKIVHRYPV
14600670_Aeropyrum_pernix           VHSAPGHGTIDYEIAVVSLVDERGFMTEAKAIVEDLRLFHAATIVHRYPV
84490249_Methanosphaera_stadtm      VHTAPGHGPEDYEVGPFCPVGEDGCYTSDDRITHELELLNYGTIDHRVGF
11498241_Archaeoglobus_fulgid       VHIAPGHGVEDYELGVFNPVDDRGVYTEEDDIIDDLYLLAEERIVHRYGH
14521127_Pyrococcus_abyssi          VHTAPGHGEEDFEVGVYSPLDDQGKYTE-PKIIEHLRLVKAGEIEHKYPH
75401346_Pseudomonas_fluoresce      VHIAPAFGEDDALLGAPNPVRDDGTFSDAPRILADLKLFKQEQIEHNYPH
102191610_Rickettsia_canade         VHMAPGFGEDDQILCLVCPVDNSGKFTKEDKIIIKLKWLKTEQYIHNYPH
116670944_Arthrobacter_sp.          VHQAPAYGEDDQKVCVVLSVDEGAKFLPLKPITQVLRLVRQASYEHSYPH
15827608_Mycobacterium_leprae       VHIAPAYGEDDMATAPVTPVDSNGCFDATAQIIRDLKLLRHETYEHPYPH
```

```
54023744_Nocardia_farcin            VHMAPAFGEEDMEVCLVQPLDPGGRFTSMPVIIKDLKLLRHETIEHSYPH
19074910_Encephalitozoon_cunic      VHCAPGFGECDYNAFVPCPVDENGRYTSEKAILSDIRVLMNQRIVHKYPF
51599084_Borrelia_garini            VHIAP-FGEEDYRVLIIDPLDAECKFTNRKKIIENLKLFKRENYLHRYPF
42528163_Treponema_dentic           VHTAPGFGEDDNRIFTVCPVDAECKFTHEKQIMERLKLFKKAQILHSYPH
46446128_Protochlamydia_amoeba      VQTAPAFGEIDFYACPVCPVDNNGQFTDEKDIIKRLKVIHQGTCHHRYPF
89898154_Chlamydophila_felis        VHMAPAFGEADFFVCIVCPVNNHGCFTEEKGIIKSLKVFYHGTVMHRYPF
118749311_Marinomonas_sp.           VHMAPAFGEDDNRVCSVCPLDSRGRFTHEKAIMQTLKLYRQETITHSYPF
68130224_Leishmania_major           VHQAPAFGEDDYQACFVCPVDENGMFTSEPEIIKALELFHKASIVHSYPF
CM4722_Cyanidioschyzon_merolae      VHLSPAFGEDDYRVCLPCPVDEDGKFTEPRGLIRKMKLVKHETYTHSYPF
118380025_Tetrahymena_thermo        VHCAPGFGDDDYKVSPPVPVDENGHFTNVKLIRKNLKLLVDSSFKHNYPF
23619270_Plasmodium_falcip          VHCAPSYGEDDYRVCLIDPLDWNGYFTNENVIKKKLKLMSNNTIVHSYPF
71076452_Giardia_lambli             VHIAPFFGEDDYRVGIVCPLDDSGNFLPDKPILKYLKLVSQSTCTHSYPY
42523714_Bdellovibrio_bacter        VHMAPAFGEEDYYACMVNPVDDDGMFTMEKDIIADLKLFKQDTIQHSYPF
6319395_Saccharomyces_cerevi        VHNAPAFGEEDNAACLPNAIDDLGRFTKDKLIIKYLTLLLASQIRHSYPF
66816517_Dictyostelium_discoi       VHTAPAYGEDDFNVCILNSVDANGCFTSDKQISIYLKMVNSANLVHSYPY
24668543_Drosophila_melano          VHNAPYFGEDDYRVCVLCPVDEAGRFTNEKQIMAALKLVSSGQVKHSYPF
94721239_Homo_sapien                VHQAPYFGAEDYRVCPVCPVDASGCFTTEKSIIRTLKLLVATTFTHSYPF
116061521_Ostreococcus_tauri        VHQAPAYGEDDYRVCIPDPVDANGCFMEPKLLIQEVKLVDNSRIVHSYPF
30681405_Arabidopsis_thalia         VHCAPGFGEDDYRVCLVVAVDDDGLFTERKDIIEAVKLVKTGSFTHSYPF
67481173_Entamoeba_histol           VHQAPGFGEDDYRVCLVCPIDFSGKFTAEKDIIERLKIFNVATITHSYPF
57157197_Trichomonas_vagina         VHTAPGFGEDDYGACIVCPIDDNCCFTEEAQIMDRLKLIKKETIKHEYPY
48477483_Picrophilus_torrid         VHAAPAFGADDFEIGILNPVDQDGRFNEKKSIINYLKLFKAETMKHDYPF
84029573_Thermoplasma_acidophi      VHAAPAFGADDYQIGILNPVDKNGRFADPEDIIVYLKLLKSEKYEHSYPF
60681847_Bacteroides_fragil         VHIAPTFGADDAQVAPLQLVNKKGELRPMVSICMMMKAFKIEKHVHNYPH
83815822_Salinibacter_ruber         VHMAPAFGEEDHAVALFNPIDKDGEFTDAKTITDDLKLYKHETYLHNYPH
21673150_Chlorobium_tepidu          VHIAPAFGADDYELSMLQPVARNGCFTAEKPIMQRLKLYRRETIQHTYPF
111221641_Frankia_alni              VHQSPAFGAEDLAVCVVNPIGTDGRFLADAPLTADLRLWRGLTFTHSYPH
28210025_Clostridium_tetani         VHTAPAYGEDDNIVCMINLVDKEGKFIDCSKIIEYMDLYKSEKFTHSYPH
47570085_Bacillus_cereus            VHIAPAYGEEDYRVVFLHVVDEKGEYTEAVDIVRYLALYHKEKYEHSYPH
87311784_Blastopirellula_marin      VHQAPAFGEVDYDVLLICAVGPDGKFTAEKDIAHELKLFLLDQYLHDYPF
94985204_Deinococcus_geothe         AHEAPAYGAEDLELALLFGVDDHGILQVTKGLIADLKMFHAGTIRHRYPF
76261183_Chloroflexus_aurant        VHIAPAYG--DLEVGTLFSVGLDGRVLPEPDITRNLKLLRSGRVRHYYPF
                                    .: :*  *  *         :                 :          *

118193839_Cenarchaeum_symbio        CWRSHHRIVWLARRGWFYKLGLGSEVVDAAESVEYFFPRNRFLGIVKEKH
6325217_Saccharomyces_mt            DWRSKKPVIIRATPQWFADLHVKNLALESISRVKFCPGYSRLSSFMKSRN
66800699_Dictyostelium_mt           DWRTKKPIIIRTTLQWFVGLKIQKTALQSIERVNMVPGSNRLSSMIGKRT
118751535_Marinomonas_sp.           CWRTKTPLIFRATPQWFISMTLLDSAKHAVEGVKWVPGQNRMEGMLNNSP
46129096_Haemophilus_influe         CWRHKTPIIFRATPQWFIGMELRQQALGEIKQVRWIPGQARIEKMVENRP
75237181_Escherichia_coli           CWRHKTPIIFRATPQWFVSMDLRAQSLKEIKGVQWIPGQARIESMVANRP
47572014_Rubrivivax_gelati          CWRHKTPVIYRAAAQWFVRMDLRQTALAAIDATAFYPGRARLRDMIANRP
1174519_Pseudomonas_fluorescen      CWRHKTPLIYRATAQWFIGMDLRVRSLKAIEDTKFVPGQARLHSMIANRP
83594296_Rhodospirillum_rubrum      SWRSKAPLIFRNTPQWFISMELRDKALAAIDETRWVPGRNRIRAMIESRP
94968888_Acidobacteria_bacter       CWRCHNPIIFRATEQWFIAMELRSVALDEIKKVKWDPGEERISNMIATRP
15644113_Thermotoga_maritima        CWRCKGPVIFRATEQWFISVDLRQKVLEEIDKVKWIPGRNRIRSMVEERP
15605834_Aquifex_aeolic             CWRCKNPVIFRATPQWFIGMDLRQRALEEIEKVKWIPGKNRIKSMVENRP
116747573_Syntrophobacter_fuma      CWRCKNPVIFRATEQWFISMELRGRALQWIDRVDWIPGRDRIHNMIANRP
116057018_Ostreococcus_tauri        DWRTKKPTIFRATEQWFASVEFREDALKELDKLQFIPGSKRMRPMVSGRA
CM546_Cyanidioschyzon_merolae       DWRTREPTIVRATEQWFASVRFRDKALEALQSVQWVPGENRIRGMIESRD
33239720_Prochlorococcus_marin      DWRTKKPTIFRATEQWFASVEFRNDALNSIEKVEWLPGKKRIKSMVEDRG
22327660_Arabidopsis_cp             DWRTKKPTIFRATEQWFASVEFRTATMDAINNVKWVPAVNRISAMTSSRS
56751678_Synechococcus_elonga       DWRTKKPTIFRATEQWFASVEFREQALQAIAEVDWIPGENRITSMVSERS
113477079_Trichodesmium_erythr      DWRTKKPTIFRATEQWFASVEFRSEALKAIAEVNWIPGENRITPMVNDRS
67921387_Crocosphaera_watson        DWRTKKPTIFRATEQWFASVEFRDAALTAIKTVNWVPGENRITSMVGDRS
34763705_Fusobacterium_nuclea       DWRSKTPVIFRATEQWFIRMELREKTLKAIDKINFIPGKNRIGSMMETRP
47570310_Bacillus_cereus            DWRTKKPIIFRATAQWFASIEFRKELLEAVAETKWVPGETRLHNMVRDRG
46852147_Homo_mt                    DWRTKKPVVIRASKQWFINITIKTAAKELLKKVKFIPALNGMVEMMDRRP
24665024_Drosophila_melano          DWRTKKPVIIRASEQWFINTELKTPAADALEQVEIYPSKKALLTQLQKRP
149179295_Planctomyces_maris        CWRTGDELIFRLVDEWFINMDWREEIKDVTRQIDWVPGEQHELEWLTNMR
18312756_Pyrobaculum_aeroph         CWRCGSKLILRADRQWFIAISIRDKMYAELQKVNVVPRD-RFDIFVQNAR
70606414_Sulfolobus_acidoc          CWRCKTPLILRAIEQWFIAVSLKDHLMGEIDRVRWIPGKTRIGNMVKEVR
14600670_Aeropyrum_pernix           CWRCKTPLLLRATTQWFVAVTLKNQLMREAEKVEWQPAKTRFMNMLRELR
84490249_Methanosphaera_stadtm      CWRCKTPIIYIATKQWFIKVTIKDQMLEQVDSVEWVPGESRFRNWVENAR
11498241_Archaeoglobus_fulgid       CWRCKTPIIYRATEQWFIKISLKDEMLEEIDKVMWIPGSARFKDWVSNAK
14521127_Pyrococcus_abyssi          CWRCKTPLIFRATDQWFLKISVKDKIIKENDKVTWYPKI-RFDNGVRDSG
75401346_Pseudomonas_fluoresce      CWRCDNPLIYRAVESWFIRASLREQLVENNSQVNWVPKEGRFGDWIRNAR
102191610_Rickettsia_canade         CWRTDTPLIYKAVPSWYVKVTFKDRIVELNQQINWIPKDNVFGKWLENAR
116670944_Arthrobacter_sp.          CWRCRNPLIYRAVSSWYVEVTFKDRMSELNQEINWIPKDGQFGKWLANAR
15827608_Mycobacterium_leprae       CWRCRNPLIYRAVSSWFVAVTFRDRMVELNQQITWYPKDGQFGKWLQGAR
54023744_Nocardia_farcin            SWRSGQPLIYMAVPSWFVAVTFRDRMVELNKQITWVPRDGQFGKWLEGAR
19074910_Encephalitozoon_cunic      CWRSDTPLLYKLVPNWFVKVKHVDSLLRNNEKINWVPKYKRFHNWLENAR
51599084_Borrelia_garini            CYRTNYPIIYRPISSWFVNVEIKTQLLEVNEKINWMPKKGRFGKWLENAK
42528163_Treponema_dentic           CWRCSSPLIYRAVASWFVSVTIKDKLLNANSKINWQPKTGRFGKWLEGAR
```

```
46446128_Protochlamydia_amoeba      CPRSDTPLIYKTVRTWFVAVEIKDRLLAANSQIHWTPQYGRFGKWLEGAR
89898154_Chlamydophila_felis        CWRTDTPLIYKTVNSWFISVEIKDKMLRANRKIHWVPKDGRFGKWLDGAR
118749311_Marinomonas_sp.           CPRSDTPIIYRSVPSWYIKVEMRAELQANNEQINWVPQQGRMGKWLEGAI
68130224_Leishmania_major           CWRSDTPLIYKAVESWFVNVEFRDRLIECNDKTYWVPKTRRFSNWLAEAR
CM4722_Cyanidioschyzon_merolae      CWRSDTPLIYRAVPSWFIAVEFRDRLLDCNARTYWVPREHRFANWLENTR
118380025_Tetrahymena_thermo        CWRSDTPLIYKAVHCWFIKVTLKDDLLANNKKAYWVPQEGRFNNWLQNVS
23619270_Plasmodium_falcip          CWRSDTPLIYRAIPAWFVRVSSTNKLVENNETTYWIPKEKKFHNWIKDAK
71076452_Giardia_lambli             CWRSDTPLIYRAIPSWFVRVEIKDSLIRNNLKASWVPQEKRFHNWLENAR
42523714_Bdellovibrio_bacter        CYRSDTPLIYRAVSSWFVAVEIKEELIANNKQTSWVPRDGRFGNWLEGAR
6319395_Saccharomyces_cerevi        CWRSDTPLLYRSVPAWFVRVKIVPQMLDSVMKSHWVPKEKRFANWIANAR
66816517_Dictyostelium_discoi       CWRSDTPLIYKAVGSWFVRVEIRDKLLANNDKTYWVPKEKRFANWLKNAT
24668543_Drosophila_melano          CWRSDTPLIYKAVPSWFVRVEMSKNLLDCSSQTYWVPKEKRFGNWLKEAR
94721239_Homo_sapien                CWRSDTPLIYKAVPSWFVRVEMVDQLLRNNDLCYWVPREKRFGNWLKDAR
116061521_Ostreococcus_tauri        CWRSQTPLIYRAIASFFVKVEVKERLVANNLETRWVPQEKRFHNWLESAH
30681405_Arabidopsis_thalia         CWRSDTPLIYRAVPSWFVRVELKEKLLKSNEQTEWVPKDKRFHNWLENAR
67481173_Entamoeba_histol           CWRSDTPLIYRAIPSWFVNVEFKDKLVESSQQTYWVPREGRFENWLRNAR
57157197_Trichomonas_vagina         CWRSGTPLIYRCIPSWFVRIEHRELLIENTNNTHWVPRDGRFIEWLKNAR
48477483_Picrophilus_torrid         CYRCGTRLLYYPLDTWFIKVSIRKKLLENNEKINWVPKNGRFGNFLEEAK
84029573_Thermoplasma_acidophi      CYRCDTPLLYYPLDAWFIAVSIRDKLVEYNERINWKPKHGRFGNFLGEAK
60681847_Bacteroides_fragil         CWRTDKPVLYYPLDSWFIRSTCKERMIELNKTINWKPGTGRFGKWLENLN
83815822_Salinibacter_ruber         DWRKGTPLMSYPVESWFIETTLKDRMVELNDTINWQPGEGRFGEWLENNV
21673150_Chlorobium_tepidu          SWRYDVPVIYYARESWYIRTTIAPRMVALNKTINWNPGTGRFGNWLEENK
111221641_Frankia_alni              CWRCHTPLIYYPLPSWYIRTTIREQLLAQNEATDWHPKTGRYGEWLRGNV
28210025_Clostridium_tetani         CWRCDTALLYYPTDSWFVRMTLRDKLLENNNKVNWYPRTGRFGKFLENVI
47570085_Bacillus_cereus            CWRCDSPLLYYAGESWLIRTTIKDTFLQNNDTVTWYPKHGRFGKFLENMV
87311784_Blastopirellula_marin      CWRADDPLIQYPRKSWFLRTTFKDEMVANNQQINWLPRDGRFGNFLESNV
94985204_Deinococcus_geothe         HDRTGDPILYFAKKGWYIRTNVADRMLETNEEINWVPKHGRFGHWLEGNV
76261183_Chloroflexus_aurant        CWRCSTPLLYYAKRSWYIRTTFKADLVANNQQIHWVPRDGRFGNWLENNI
                                      *       :        :

118193839_Cenarchaeum_symbio        PWCISRERFWGCPLPVWNNWLYSRQEIELHRPWIDEVKIKTKREQFVLDT
6325217_Saccharomyces_mt            EWCISRQRSWGIPILSFYEILAHAIEKNAWFDNDMKEWLPYCRSQDTMDV
66800699_Dictyostelium_mt           DWCISRQRVWGCPIPVLYESINHIKELDCWFEMSTQQLLPFVKGTDTMDV
118751535_Marinomonas_sp.           DWCVSRQRTWGVPIALFIRLIEEVAKRDAWFEMEAEELLGYSKVTDTLDV
46129096_Haemophilus_influe         DWCISRQRTWGVPMTLFVELLEEVAKRQAWWDLDEKELLGYRKVPDTLDV
75237181_Escherichia_coli           DWCISRQRTWGVPMSLFVELMEEVAKRQAWWDLDAKEILGYVKVPDTLDV
47572014_Rubrivivax_gelati          DWCISRQRNWGVPLPFFLALMDRAAALEAWSRLDPREWLGYAKSTDILDV
1174519_Pseudomonas_fluorescen      DWCISRQRNWGVPIPFFLELMEVVAQREAWFKLDAAELLGYDKISDTLDV
83594296_Rhodospirillum_rubrum      DWCVSRQRSWGVPIAIFVAVTERIAQADAWFTGDPRRFLGFEPVRDIVEV
94968888_Acidobacteria_bacter       DWCISRQRLWGVPIAVFFAVNAGVVERDTWYKHQASELLPFRKEMDIIDV
15644113_Thermotoga_maritima        DWCISRQRVWGTPIPAVKKVIEHFMKINAWFEKEVEELIPFEKMLDTLDV
15605834_Aquifex_aeolic             DWCISRQRFWGVPITVFYEVFERVAQLDVWFELTSSQLLPFTKEEDILDV
116747573_Syntrophobacter_fuma      DWCISRQRSWGVPICVFTELFEKTAALDCWFEMTSEELLPFEKEEDILDV
116057018_Ostreococcus_tauri        DWCISRQRAWGVPIPAFYAIIKHVTEINAWWELEVEDLLPYVRGTDTMDV
CM546_Cyanidioschyzon_merolae       DWCISRQRPWGVPIPVFYEIIQYVSEIDAWWTLPIEQLLPLRRGLDTMDV
33239720_Prochlorococcus_marin      DWCISRQRNWGVPIPVFYATIKHIYNLDAWWELPISELLPWEKGKDTMDV
22327660_Arabidopsis_cp             DWCISRQRTWGVPIPAFYETINHVKSIDAWWYMSVEDLLPYEKGTDTMDV
56751678_Synechococcus_elonga       DWCISRQRTWGVPIPVFYETIAHVRAIDAWWELDVADLLPYRKGTDTMDV
113477079_Trichodesmium_erythr      DWCISRQRSWGVPIPVFYETVAHVQKIDAWWEMSVEELLPYRKGTDTMDV
67921387_Crocosphaera_watson        DWCISRQRSWGLPIPVFYETIKYIQKIDAWWEMSIEELLPYRKGTDTMDV
34763705_Fusobacterium_nuclea       DWCISRQRVWGVPIPIFYEILDRICDLNIWVEKSPEELIGLRKETNIMDV
47570310_Bacillus_cereus            DWCISRQRAWGVPIPVFYETINHVADLNVWFEREAKDLLPFRKETDIMDV
46852147_Homo_mt                    YWCISRQRVWGVPIPVFHQTTEHIVKLDIWWTLPPEQLLPYVPGQDILDI
24665024_Drosophila_melano          YWCISRQRAWGVPIPVLYALIEHLCNLDFWWVKSVEELVPLVKGTDILDI
149179295_Planctomyces_maris        DWMVSKKRFWGLALPIWVEVIGSLAELTPHRPWIDAVKLKMSRIPDVGNP
18312756_Pyrobaculum_aeroph         DWNISRSRVWGTPLPVWRLVIGSLEELLVHRPWIDQVKISWVREPYVMDV
70606414_Sulfolobus_acidoc          DWVISRQRFWGTPLPIWVIVVGGVDELDLHRPWIDSVVVRARRISDVADV
14600670_Aeropyrum_pernix           DWVISRQRYWGIPLPVWRHVVGSVEELNLHRPWVDRVELKMKRVPDVLDV
84490249_Methanosphaera_stadtm      DWTISRQRYWGIPLPIWTVVIGSKEELFVHRPHVDNITIPMHRVPDVLDV
11498241_Archaeoglobus_fulgid       DWCISRQRYWGIPIPVWIKVVGSINEIDLHRPKIDAVTFSMRRVPDVFDV
14521127_Pyrococcus_abyssi          DWVISRQRYWGIPLPIWQYVVGSWRELDLHRPYVDAFIIKMRRVKDVVDV
75401346_Pseudomonas_fluoresce      DWAVSRNRFWGAPIPVWREVMGSIAQIDLHVPHIDEHRFAMSRVTGVFDC
102191610_Rickettsia_canade         DWSISRNRFWGTPLPVWKDVYGSIEELDLHRPFIDELTRAMRRIEDVFDC
116670944_Arthrobacter_sp.          DWSISRNRYWGSPIPVWQDVYGSLAEIDLHRPFIDELVRPMRRVEDVLDV
15827608_Mycobacterium_leprae       DWSISRNRYWGTPIPVWKDVYGSIDELNLHRPYIDELTRPMRRIPDVFDV
54023744_Nocardia_farcin            DWNISRNRYWGSPIPVWVDVYGSLEELDLHRPAIDQLTRPMRRVPEVLDC
19074910_Encephalitozoon_cunic      DWSISRNRFWGTPIPLWVICIGSVGELDIHREFIDGIVI-YRRVEEVLDC
51599084_Borrelia_garini            DWAISRNRFWGNPIPIWIICVGSRKELDLHKDKIDKITWPFIRTSEVLDC
42528163_Treponema_dentic           DWAISRNRYWGNPIPIWKICVGSREELDMHKHFVDKISIPMKRVSEVLDC
46446128_Protochlamydia_amoeba      DWAISRNRYWGTPIPLWRHVVGSIEELDLHRHFIDEMSF-FKRIPEVFDC
89898154_Chlamydophila_felis        DWAISRNRYWGTPIPIWKLVIGSIKELDLHCHFIDQLKV-FQRVPYVFDC
118749311_Marinomonas_sp.           DWSVSRNRYWGTPLPIWVLCLGSIEELDLHRDHVDELTFSYRRIAEVFDC
68130224_Leishmania_major           DWNVSRNRYWGTPMPVWHVCVSNAKQLDIHRQFVDNITIPLKRVPMVFDC
```

```
CM4722_Cyanidioschyzon_merolae      DWNVSRNRYWGTPLPVWTIVVGSIAELDLHRHNIDSIEIRLRRVEEVFDC
118380025_Tetrahymena_thermo        DWCFSRSRFWGNPIPIWVVCIGSVEELDLHKDFIDHLTIPLRRIDEVFDC
23619270_Plasmodium_falcip          DWCISRNRYWGTPIPIWCICVESIKHLDLHRHYIDNIEIKLKRIPEVFDC
71076452_Giardia_lambli             DWSISRNRFWGCPIPIWMKVIGSISELDIHRDYLDNMTIPMRRISPVFDC
42523714_Bdellovibrio_bacter        DWAISRNRFWGTPLPIWRMCIGSRAELDLHIEFVDKITIPLKRVDGVLDC
6319395_Saccharomyces_cerevi        DWNVSRNRYWGTPIPLWVVCVGSIKELDLHRDVIDKLTIPLKRIEEVFDC
66816517_Dictyostelium_discoi       DWAVSRNRYWGTPIPLWIVVIGSIDELDLHRESIDHITIPLRRIEDVFDC
24668543_Drosophila_melano          DWAISRNRYWGTPIPIWRVVIGSIKQLDLHRESIDHIEIPLRRIAPVFDC
94721239_Homo_sapien                DWTISRNRYWGTPIPLWVVCIGSVAELDLHRESVDHLTIPLHRISEVFDC
116061521_Ostreococcus_tauri        DWAISRNRYWGTPIPVWSLVVGSIAELDLHRHFIDDLEIPLRRVEDVFDC
30681405_Arabidopsis_thalia         DWAISRSRFWGTPLPIWIVIMDSVEKLDLHRHIDHITIPLRRVEDVFDC
67481173_Entamoeba_histol           DWAISRNRFWGTPIPIWTCVCVGSVKELDLHRESVDNLIIELHRISEVFDC
57157197_Trichomonas_vagina         DWAISRNRYWGTPIPIWTYCIGSIKELDIHCHFIDQITFKLHRIPEVFDC
48477483_Picrophilus_torrid         DWALSRDRYWGTPLPIWRLAIGSRDDLDLHRPYIDDVVLKMHRESYVIDT
84029573_Thermoplasma_acidophi      DWNLSRDRFWGTPLPAWRVFVGSRKEIDLHRPYIDEVRFKMSREPYVIDT
60681847_Bacteroides_fragil         DWNLSRSRYWGTPLPIWRKCIESVEELDLHRPYVDDIILVMKREADLIDV
83815822_Salinibacter_ruber         DWALSRRRYWGTPLPVWEEVIGSVEELDLHRPFVDELTWEMRRVPDLIDV
21673150_Chlorobium_tepidu          DWALSRERFWGTPLPVWVFAVGSVAELDLHKPFVDRI-WFFNRTPELIDV
111221641_Frankia_alni              DWALSRNRYWGTPLPIWRVCVGSVAELDPHRPFVDEVTISAHRVPEVIDV
28210025_Clostridium_tetani         DWGISRDRYWGTPLPIWEECIGSISELELHKPYIDKVKLKMKRTREVIDC
47570085_Bacillus_cereus            DWNISRNRYWGTPLNVWEFAPKSIADLELHKPYVDEVQVCMNRTPEVIDV
87311784_Blastopirellula_marin      DWALSRERYWGTPLPIWVEAVCSYAELKIHKPYIDAITYDMQRVTEVIDC
94985204_Deinococcus_geothe         DWAISRERYWGTPLPFWMQVVGSVAELDLHRPSIDDVTFTYRRVPEVLDV
76261183_Chloroflexus_aurant        DWAISRERYWGTPIPIWTVCIGSLAELDLHRPYIDEVVWEMRRIPDVADC
                                     * .*: * ** .:                                  :

118193839_Cenarchaeum_symbio        WHNSGSAPFSSLIPAPFFTEGIDQTRGWAYTLLVENVIMSGSSPYKSFLF
6325217_Saccharomyces_mt            WFDSGSSWSVIKLPSPLYLEGSDQHRGWFQSSLLTKVASSNVPPYEEVIT
66800699_Dictyostelium_mt           WFDSGTSWRGVLGRADIYLEGSDQHRGWFQSSLLTSVCVRDIEPYKNVVT
118751535_Marinomonas_sp.           WFDSGVTHYSVIFPADLYLEGSDQHRGWFQSSLKTSIAIRGVPPYKQVLT
46129096_Haemophilus_influe         WFDSGSTYSSVVQNIDMYLEGSDQHRGWFMSSLMLSTATDSKAPYKQVLT
75237181_Escherichia_coli           WFDSGSTHSSVVHAADMYLEGSDQHRGWFMSSLMISTAMKGKAPYRQVLT
47572014_Rubrivivax_gelati          WFDSGSTFFHVLPEADLYLEGHDQHRGWFHSSLLIACAIEGRAPYRGLLT
1174519_Pseudomonas_fluorescen      WFDSGTTHWHVLPRADLYLEGSDQHRGWFHSSLLTGCAIDNHAPYRELLT
83594296_Rhodospirillum_rubrum      WFDSGSTHAFVLWPADLYLEGSDQHRGWFHTSLLESCGTRGRAPYDAVLT
94968888_Acidobacteria_bacter       WFESGSSKLAVI--ADFYTEGGDQHRGWFHSSLLCHIGAQGHAPYKHVAT
15644113_Thermotoga_maritima        WIDSGASFEYITFPLDMYLEGSDQHRGWFHSSIFLAVAKRGSAPYKEVLT
15605834_Aquifex_aeolic             WFDSGCSHAAVIQKADLYLEGSDQHRGWFQASLLESVGSYLEAPYKAVLT
116747573_Syntrophobacter_fuma      WFDSGVSHAAVLSPADLYLEGSDQHRGWFHSSLLTAVGTRNAAPYKSVLT
116057018_Ostreococcus_tauri        WFDSGSSWAGVVFPADVYLEGSDQHRGWFQSSLLTGVGAMGQAPYKTILT
CM546_Cyanidioschyzon_merolae       WFDSGSSWASLSVVADLYLEGSDQHRGWFQSSLLTSVAVRGYAPFRAVLT
33239720_Prochlorococcus_marin      WFDSGSSWTSVIYPADLYLEGSDQHRGWFQSSLLTSVAVNSHAPYLKVLT
22327660_Arabidopsis_cp             WFDSGSSWAGVLFPADVYLEGTDQHRGWFQSSLLTSIATQGKAPYSAVIT
56751678_Synechococcus_elonga       WFDSGSSWAAVAYPADLYLEGSDQHRGWFQSSLLTSVACNGHAPYRRVLT
113477079_Trichodesmium_erythr      WFDSGSSWAAVAYPVDIYLEGSDQHRGWFQSSLLTSIATNGVAPYQTVLT
67921387_Crocosphaera_watson        WFDSGSSWASVAYPVDIYLEGSDQHRGWFQSSLLTSVAVNGIAPYKTVLT
34763705_Fusobacterium_nuclea       WFDSGSSHRGVLRPADLYLEGSDQHRGWFHTSLLTSVASTGDSPYKSVLT
47570310_Bacillus_cereus            WFDSGSSHQAVLRPADLYLEGSDQYRGWFNSSLSTAVAVTGKAPYKGVLS
46852147_Homo_mt                    WFDSGTSWSYVLQRADLYLEGKDQLGGWFQSSLLTSVAARKRAPYKTVIV
24665024_Drosophila_melano          WFDSGSTWSAVL--ADLYLEGYDQFTGWFQSSLLMSIAARECAPYKALFV
149179295_Planctomyces_maris        WLDAGIVPFSTMYPADLVTECFGQFRNWFYALLSMATMMDGTPPFKTLLG
18312756_Pyrobaculum_aeroph         WLDSGIAWIAAVFPYDFVTEGIDQTRGWFYSLLATSVLYTGRAPYKNVLI
70606414_Sulfolobus_acidoc          WFDSGVAFFASLGPVDLVLEGHDQLRGWFFSLLRTGVILMDKAPYEAVLV
14600670_Aeropyrum_pernix           WFDSGIAFYASLKPVDFIVEGHDQIRGWFFSLLRSGVIGFGETPYRRVLV
84490249_Methanosphaera_stadtm      WIDSGVAGWAALFPYDFITEGHDQTRGWFYSQLGLGVAAFGKAPYKKVLM
11498241_Archaeoglobus_fulgid       WFDSGVASWGSIWPADFITEGHDQTRGWFYSQLGTSVVCFDKAPYKAVLM
14521127_Pyrococcus_abyssi          WFDSGIASWASLWPADFIVEGEDQVTKWFYSQQAASIVAFDTVPYRRVAM
75401346_Pseudomonas_fluoresce      WFESGAMPFASRFPADFIVEYLAQTRGWFYTMMVISTGCFEQNPFKNAMC
102191610_Rickettsia_canade         WFESGSMPYSQAFPADFIVEYVAQTRGWFYTLMVLSTALFDRPPFLNCIC
116670944_Arthrobacter_sp.          WFDSGSMPYGQVNPADFIVEYIGQTRGWFYMLHILSTALFDRPAFRNVIS
15827608_Mycobacterium_leprae       WFDSGSMPYAQVYPGDFIVEYIGQARGWFYTLHVLATALFDRPAFKTCIA
54023744_Nocardia_farcin            WFESGSMPYAQVFPGDFIVEYNGQTRGWFYTLHVLATALFDSPAFKTVAA
19074910_Encephalitozoon_cunic      WFESGSMPYAQDFPAHFIGEGLDQTRGWFYTLHVISSLLFGQPAFLNVVV
51599084_Borrelia_garini            WFESGAMPYASNFPADFIAEGLDQTRGWFYTLTILGTSLFENTAFKNVIV
42528163_Treponema_dentic           WFESGSMPYAQQFPADFISEGLDQTRGWFYTLTILAAALFDEPAFKNCIV
46446128_Protochlamydia_amoeba      WFESGSMPYAQNFPADFIAEGLDQTRGWFYTLTVLSAALFDQPAMKNVIV
89898154_Chlamydophila_felis        WFDSGAMPYAQNFPADFIAEGLDQTRGWFYTLTVISSALFDQPAFKNAIV
118749311_Marinomonas_sp.           WFESGAMPYAQVFPAAFIAEGVDQTRGWFYTLQVLSQCLFGRPAFKNVIV
68130224_Leishmania_major           WFESGSMPYAQEFPADFVSEGLDQTRGWFYTLLVLGVALFDVSPFRNVAV
CM4722_Cyanidioschyzon_merolae      WFESGAMPYASVFPAQFIAEGLDQTRGWFYTLMVLGTALFNEPPFKNVIV
118380025_Tetrahymena_thermo        WFESGSMPYGQQFPADFIGEGIDQTRGWFYTLNVISTALRNSNPYKNLIV
23619270_Plasmodium_falcip          WFESGSMPYAKVFPADFIAEGLDQTRGWFYTLLVISTLLFNKAPFKNLIC
71076452_Giardia_lambli             WFESGSMPYAQKFPGDFIAEGLDQTRGWFYTLLILSTALFDSPPSMNCIV
```

```
42523714_Bdellovibrio_bacter        WFESGSMPYAQWFPADFIAEGLDQTRGWFYTLSIIGTALFNQAPFKNVVV
6319395_Saccharomyces_cerevi        WFESGSMPYASQVPANFISEGLDQTRGWFYTLAVLGTHLFGSVPYKNVIV
66816517_Dictyostelium_discoi       WFESGSMPYAQQFPAHFIAEGLDQTRGWFYTLLVLSTALFDKPPFQNLIV
24668543_Drosophila_melano          WFESGSMPFAQQFPADFIAEGIDQTRGWFYTLLVISTALFNKAPFKNLIA
94721239_Homo_sapien                WFESGSMPYAQVFPADFIAEGIDQTRGWFYTLLVLATALFGQPPFKNVIV
116061521_Ostreococcus_tauri        WFESGSMPYAQQFPADFVAEGLDQTRGWFYTLMVLSTALFDKPAFKNLIC
30681405_Arabidopsis_thalia         WFESGSMPYAYIFPGDFVAEGLDQTRGWFYTLMVLSTALFEKPAFKNLIC
67481173_Entamoeba_histol           WFESGSMPYGQSFPGDFIAEGIDQTRGWFYSLIVISTALFGKAPFKNCVV
57157197_Trichomonas_vagina         WFESGSMPFSQFRQADFIAEGLDQTRGWFYTMTILSSLLGHPSPFKNVIV
48477483_Picrophilus_torrid         WFDSGSASYAAMFPVDFITEAIDQTRGWFYTLHVVASLLFDENAYKNVVS
84029573_Thermoplasma_acidophi      WFDSGSATYAASVPVSFITEAIDQTRGWFYVLHVIATIMFNKNAYESALS
60681847_Bacteroides_fragil         WFDSGAMPYAQIYPADFIAEGVDQTRGWFFTLHAIATMVFDSVSYKAVIS
83815822_Salinibacter_ruber         WFDSGAMPYAQWFPADFIAEGVDQTRGWFYSLHAIATVVFDEVAYENVVV
21673150_Chlorobium_tepidu          WFDSGSMPFAQLFPADFIAEGVDQTRGWFYTLHAIATLIFDRPAYRNVVV
111221641_Frankia_alni              WYDSGAMPFAQWYPAQYICEAIDQTRGWFYTLMAIGTLVFDRSSYETVLC
28210025_Clostridium_tetani         WFDSGSMPFAQHFPAQFISEAVDQTRGWFYTLTAISTAIFDTNPFENCIV
47570085_Bacillus_cereus            WFDSGSMPFAQYFPADVIAEGIDQTRGWFYSLLAVSALYTGKVPYKRVLS
87311784_Blastopirellula_marin      WFDSGAMPFAQWFPADFISEAIDQTRGWFYSQLAISTLLFGKGPFKNCIV
94985204_Deinococcus_geothe         WFDSGAMPYAQWFPADFISEAIDQTRGWFYSLHAIATMLYDQPAYRNVIC
76261183_Chloroflexus_aurant        WFDSGSMPVAQWHPADYICEAVDQTRGWFYTLHAISTLLFDRPAFKNVIC
                                    * ::*              *   *   *               .

118193839_Cenarchaeum_symbio        QGHVLDKKGNKMSKSLGNMLEAGEMLREHPADLVRFYLMWKASIEPLSFD
6325217_Saccharomyces_mt            HGFTLDENGLKMSKSVGNTISPEAIIRALGVDGLRYLIAQSNFTTDIVAG
66800699_Dictyostelium_mt           HGFLLDESGIKMSKSIGNTIVPSTVIKPYGVDLLRTWVASSDYSKDISIG
118751535_Marinomonas_sp.           HGFTVDGDGRKMSKSLGNVLSPQKVMDTLGADIIRLWVAATDYTTEMTVS
46129096_Haemophilus_influe         HGFTVDGQGRKMSKSIGNIVTPQEVMDKFGGDILRLWVASTDYTGEMTVS
75237181_Escherichia_coli           HGFTVDGQGRKMSKSIGNTVSPQDVMNKLGADILRLWVASTDYTGEMAVS
47572014_Rubrivivax_gelati          HGFATDGQGRKMSKSLGNTVVPQSVSEKLGAEIIRLWVASTDYSGDLNID
1174519_Pseudomonas_fluorescen      HGFTVDETGRKMSKSLKNVIEPKKINDTLGADIMRLWVASTDYSGEIAVS
83594296_Rhodospirillum_rubrum      HGFLLDEKGDKLSKSKGNAESPQKVVSSVGADIMRLWVVSSDYTGDIRFG
94968888_Acidobacteria_bacter       SGWTLDPQGRAMSKSLGNVVDPVDIAKRLGAEIVRLWVASVDFREDVRAS
15644113_Thermotoga_maritima        HGFIKDEQGRKMSKSLGNVVDPMEVVEKYGAEILRLWLASSDYFNDIKIS
15605834_Aquifex_aeolic             HGFIVDEKGRKMSKSLGNVISPQEVVKEFGADILRLWVVSEDYTEDVKLG
116747573_Syntrophobacter_fuma      HGFVVDGQGYKMSKSLGNVIAPEEIIRQYGAEVLRLWVSAEDYRDDVRIS
116057018_Ostreococcus_tauri        HGFVLDEKGYKMSKSLGNVIDPRTVIEGYGADTLRLWVASTDYSGDVSIG
CM546_Cyanidioschyzon_merolae       HGFVLDEKGLKMSKSLGNVVDPSEIINALGADVLRLWVASVDYMSDVLIG
33239720_Prochlorococcus_marin      HGFALDENGRKMSKSLGNIIDPWKIINAYGADVLRLWVSSVDYSVDVPIG
22327660_Arabidopsis_cp             HGFVLDEKGMKMSKSLGNVVDPRLVIEAYGADVMRLWVSSVDYTGDVLIG
56751678_Synechococcus_elonga       HGFALDEKGRKMSKSLGNVVDPAIVINPYGADVLRLWVSSVDYSSDVPIG
113477079_Trichodesmium_erythr      HGFVLDENGRKMSKSLGNVVDPVIVIEAYGADILRLWVSSVDYSSDVPLG
67921387_Crocosphaera_watson        HGFVLDEKGYKMSKSLGNIVDPNVIINPYGADLVRLWVSSVDYSSDIRIG
34763705_Fusobacterium_nuclea       HGFVNDGEGRKMSKSLGNTVSPADVIKVYGADILRLWCGSVDYRDDVRIS
47570310_Bacillus_cereus            HGFVLDGEGRKMSKSIGNIVVPKKIMDQLGGDILRLWVSSVDYQSDVRIS
46852147_Homo_mt                    HGFTLGEKGEKMSKSLGNVIHPDVVVNPYGADVLRWWVADSNVFTEVAIG
24665024_Drosophila_melano          HGFTVDEKGYKMSKSLGNVISPKQITKKYGTDALRWWVASHGTHMSITVS
149179295_Planctomyces_maris        YRLVLNEEGKPMHKSDGTAIWFEEAAEQLGVDTMRWMYLAHNPASDLRFG
18312756_Pyrobaculum_aeroph         QGLILDKHGQKMSKSKGNVIWAKDLFEKYGADPVRLYILSKVAWEDLSFD
70606414_Sulfolobus_acidoc          HGFMLDEQGREMHKSSGNYVEPSQVVSKYGRDTLRLWLLRNTTWEDAKFS
14600670_Aeropyrum_pernix           HGFALDEQGREMHKSLGNYVDFEELIAKVPRDVVRFWVLQNTVWEDLRFS
84490249_Methanosphaera_stadtm      HGFTLDEDGKKMSKSLGNVVSPEEVIEKYGADVLRFYLLDANKWDDLKFH
11498241_Archaeoglobus_fulgid       HGFTLDEQGRKMSKSLGNVVEPEEVVGQIGVDGFRLYVLYSAPWEDLRFS
14521127_Pyrococcus_abyssi          HGYVLDEKGDKMSKSLGNIIRPEEVVERAGRDTFRFYMLWATNWENLKFS
75401346_Pseudomonas_fluoresce      HGVILAKDGRKMSKRLKNYPNPMDLMQTHGSDALRVALLASPVGEDIKFS
102191610_Rickettsia_canade         HGVILDTTGQKLSKRLNNYADPLELFDKYGSDALRITMLSSNVGQELLID
116670944_Arthrobacter_sp.          HGIVLGSDGQKMSKSLRNYPDVSEVLDRDGSDAMRWFLMSSPIGGNLVVT
15827608_Mycobacterium_leprae       HGVVLGSNGQKMSKSLRNYPDVTEIFDRDGSDAMRWFLMASPIGGNLIIT
54023744_Nocardia_farcin            HGIVLGDDGLKMSKSKGNYPDVNEVFDRDGSDAMRWFLMSSPIGGNLIVT
19074910_Encephalitozoon_cunic      NGIVLAEDGRKMSKRLRNYPDPSHIFNTYGADSLRMYLISSPVAENLKFS
51599084_Borrelia_garini            NGLVLSSDGRKMSKSFKNYTDPMEVINTFGADALRLYLIMSPVADDLKYS
42528163_Treponema_dentic           NGLVLAEDGKKMSKSLRNYTDPNEVIKQFGADALRLFLMNSNVADDLKYS
46446128_Protochlamydia_amoeba      NGLILAENGAKMSKRLKNYPDPAEVIQQYGADAIRLYMLHSPAADDLSFS
89898154_Chlamydophila_felis        NGIVLAEDGNKMSKRLNNYPSPIGIMNTYGADALRLYLLNSVVAEDLRFS
118749311_Marinomonas_sp.           NGIVMAEDGKKMSKRLKNYTPPDTLMEHYGADALRLYLINSGLAEEQRFS
68130224_Leishmania_major           SGLVLAEDGKKMSKRLKNYPEPKVVIDTYGADALRMYMISSPVAEPLRFR
CM4722_Cyanidioschyzon_merolae      NGLVLAEDGKKMSKRLKNYPDPMDMIENFGADALRLYLINSPVGEPLRFK
118380025_Tetrahymena_thermo        NGIVLAADGKKMSKSKKNYDDPLLIASKYSVDAIRLYMINSPLAEEMSFK
23619270_Plasmodium_falcip          NGLVLASDGKKMSKRLKNYPDPLYILNKYGADSLRLYLINSVAAENLKFQ
71076452_Giardia_lambli             NGLVLASDGQKMSKRIRNYPDVNHIINTYGSDALRLYLVNSPAAQELRFR
42523714_Bdellovibrio_bacter        NGLVLAEDGRKMSKSLKNYPDPMEVLNQHGADALRLYLIDSPVAQELKFS
6319395_Saccharomyces_cerevi        SGIVLAADGRKMSKSLKNYPDPSIVLNKYGADALRLYLINSPVAESLKFK
66816517_Dictyostelium_discoi       NGLVLAADGKKMSKRLKNYPDPMEVVSKVGADSLRLYLINSPVAETLKFQ
24668543_Drosophila_melano          SGLVLAADGQKMSKRKKNYPDPMEVVHKYGADALRLYLINSPVAESLRFK
```

```
94721239_Homo_sapien                 NGLVLASDGQKMSKRKKNYPDPVSIIQKYGADALRLYLINSPVAENLRFK
116061521_Ostreococcus_tauri         NGLVLAADGKKMSKSLKNYPDPNTILDKYGADALRLYLIDSPVAEPLRFK
30681405_Arabidopsis_thalia          NGLVLAEDGKKMAKKLRNYPPPLEVIDEYGADAVRLYLINSPVAEPLRFK
67481173_Entamoeba_histol            NGLVLASDGKKMSKRLKNYPDPMDMINLYGADALRLSLINSPAAETVKFQ
57157197_Trichomonas_vagina          NGLIMAADGKKMSKKEKNYTDPELIMDRFGADAVRLYLTNSPAADPFSFK
48477483_Picrophilus_torrid          ISHILDENGQKMSKSKGNFIAAIDFLNDYGADAARMFFFTGAPWNSKSVN
84029573_Thermoplasma_acidophi       INFILDAQGRKMSKSKGNSVYALDFLNEVPPDSLRLFFLYGAPWKSKNLD
60681847_Bacteroides_fragil          NGLVLDKNGNKMSKRLGNAVDPFSTIEQYGSDPLRWYMITNSSWDNLKFD
83815822_Salinibacter_ruber          NGLVLDEDGNKMSKSVGNTVEPFEVIDDYGADVVRWFMMSNAPWESIRFS
21673150_Chlorobium_tepidu           NGHILDKSGQKMSKSKGNVVDPFESMEQYGADAIRWYLMITSPWRPKLFN
111221641_Frankia_alni               LGLLLDAEGRKMSKHVGNVLDPFELFERHGADAVRWLMLAGGSWADRRVG
28210025_Clostridium_tetani          LGHVLDKHGLKMSKSKGNVVDPFDVLDSAGADASRWHFYTASAWLPTRFS
47570085_Bacillus_cereus             LGHVLDEEGQKMSKSKGNALDPVDLVEKFGADALRWALLVDSAWNAKRFS
87311784_Blastopirellula_marin       LGLMLGEDGQKMSKSKRNYREPNEIFDKYGADALRWYLFANQPWTSIRYN
94985204_Deinococcus_geothe          LGHIVDEHGAKMSKSKGNVVEPLPLFDRYGADSVRWYMFMASDGDQKRFS
76261183_Chloroflexus_aurant         LGHILDENGLKMSKSKGNVIEPEEVINAYGVDALRWYLFTAAPGNARRFS
                                             *  : *   .             :  *

118193839_Cenarchaeum_symbio         MMSRPYQVLSTLYHLHLYFKLRPPDIWILSKLQGLIRDAAGYSERCRLHE
6325217_Saccharomyces_mt             VMKHVGEALKKVRLTFRYLLLRRVDQYTLYKINELLETTREHYQKYNFSK
66800699_Dictyostelium_mt            ILIKILDGIKKIRNTLRFMLLSSLDKYALHRVFKLQESVTRHYDQFQFQK
118751535_Marinomonas_sp.            ILKRVADSYRRIRNTARFMMMIALDRWIVDRAALLQKELNTAYNEYQFHT
46129096_Haemophilus_influe          ILKRAADSYRRIRNTARFLLMISLDRWAVACALDAQNEIKDAYDNYQFHT
75237181_Escherichia_coli            ILKRAADSYRRIRNTARFLLMVVLDRWAVGCAKAAQEDILKAYEAYDFHE
47572014_Rubrivivax_gelati           ILARVVDAYRRIRNTLRFLLLLEIDRYAIDRAAQLQAEILAHYEVYEFHP
1174519_Pseudomonas_fluorescen       ILARSADAYRRIRNTARFMLMLALDRWAVDRTLLLQRELQEHYGEYRFWN
83594296_Rhodospirillum_rubrum       ILKQTTDTYRRLRNTLRFLLMPELERWVLHRLSEIDVKVRGCCDAFSFHE
94968888_Acidobacteria_bacter        LMQRVAENYKKIRNTFRYILLQPFDQYILLRLAEVIGDVRDWYDEMSFHK
15644113_Thermotoga_maritima         IVEQQTEVYRKIRNTFRFLLLLTIDRWALGRLQEIIKRATEYYDSYEFSK
15605834_Aquifex_aeolic              LLKKIADDYRKIRNTLRFIILHHFDRWIISELQNLLKKVHENYEKFLFYR
116747573_Syntrophobacter_fuma       ILKRLSEAYRRIRNTCRFLLMDELDRFALHQLQEFVRRVRQAYERFEFHR
116057018_Ostreococcus_tauri         IIKQTSEAYRKLRGTIRFAMLPVFDKYVLHRLNATVKEIESSYKAFDYSR
CM546_Cyanidioschyzon_merolae        ILKQTADAYRKIRNTLRFLALPSLDRYILRRAAAVLKDIEDAYKNFTFNR
33239720_Prochlorococcus_marin       ILSQLSDVYRKVRNTARYLLLPILDRWMLNRTADVVDEISIAFERYEFSK
22327660_Arabidopsis_cp              ILRQMSDIYRKLRGTLRYLLLPIIDQHALFQLENVVKNIQECYENYQFFK
56751678_Synechococcus_elonga        ILKQMADVYRKIRNTARFLLLPELDRYLLHRLREVILEIQDAFESFQFFR
113477079_Trichodesmium_erythr       ILKQIADIYRKIRNTARFLLMPELDRYMLHRITEVFAEVKDGFDKFQYFR
67921387_Crocosphaera_watson         ILKQLFDIYRKIRNTARFLLLPELDRYMLHRITEVFTEVTDAFDTYQFFR
34763705_Fusobacterium_nuclea        IVKQMSEAYRRIRNTARYILMLEIDKWALNKLEVLKRNVTESYDKYEFYN
47570310_Bacillus_cereus             ILKQVAEVYRKIRNTFRFLLLREVDRYMLVKLNDLITKVKEAYETYDFAA
46852147_Homo_mt                     VLNAARDDISKLRNTLRFLLMYVIDQYMLHLLQDLANKITELYKQYDFGK
24665024_Drosophila_melano           LLQQAAENLCKIRGTMRYLKKSYLNRYLLSQLVEFESEVEKLYQAYEYNR
149179295_Planctomyces_maris         ADEIRRQVLIPLWNSYAFFVRPEIDRWILSNLQALLSSAKTEIEAYNFAG
18312756_Pyrobaculum_aeroph          EVKYVIGDLNILWNVVKFADGLEEDKWILSELNIMISEFTNFVKNFEFHK
70606414_Sulfolobus_acidoc           SLDMTRRDLNIIWNVYVFANLKLEDIWLLSRYYRMLKEVIEAMKEYKVHE
14600670_Aeropyrum_pernix            -LDLMRRDFNVIWNVYAFASLEVEDRWILSRLASLKRRYLTAMEDLRIHD
84490249_Methanosphaera_stadtm       EVQNSSKLFNILWNVYYFATFREEDLWIESRINSLIKSVGEDIESLVFNR
11498241_Archaeoglobus_fulgid        EARNINRMLNVVWNAVRFAHLKIEDRWILSRLESFIKEANEAMEGYQVHR
14521127_Pyrococcus_abyssi           -VEQVRRMLNILWNVYVLASFREEDKWILSRVNSLISEVENGIESFYLTK
75401346_Pseudomonas_fluoresce       VRDVVRRYHLLFWNCLQFYKDNVLDHYLLHELAALESDIKMWMESLDFSK
102191610_Rickettsia_canade          VFDTLRLFIKPIWNAYHFFTENVLDIYILSKLKIAVQKIEKSLDNFDTQA
116670944_Arthrobacter_sp.           IRDGVRQVILPLWNVYSFFTADTLDQYLMANTGDLVRNMTAQLDGYDISG
15827608_Mycobacterium_leprae        IREGMRQVLLPLWNAYSFLASHVLDRYILAKLAVLRDDLSQAMEVCDISG
54023744_Nocardia_farcin             IREGVSHALRPLWNAWTFLQTHVLDRYILAKLAQTRDGMTEALEVYDIAG
19074910_Encephalitozoon_cunic       VKEVLKTLIIPWYNSLGFYLSLPMDGWITASFDNFAWSLTRKMRKYELSS
51599084_Borrelia_garini             VRDVLKNIIIPIWNAYSFFTSNNLDKWIISELESLKKILNKEIDKYNLTK
42528163_Treponema_dentic            VRDVLKGILIPFWNSYSFYINNPLDLWILSVTEKLVADVTEALDKYDLSQ
46446128_Protochlamydia_amoeba       VELVLRQILLPLWNAYTFFLELAIDQWIISLLNKLVHEVEQGMDDYDLSR
89898154_Chlamydophila_felis         VESILKQILLPLTNVLSFFKYSEIDKWILSDLYTVVGKVRESMNSYNLNT
118749311_Marinomonas_sp.            VQDMVRQVLLPWLNGFRFFSDNILDQWILSRLQTLTTKVNQEMQAYHLYN
68130224_Leishmania_major            VKGVVKDILLPLFNAAKFFITNEMDRWILASCQSLLRYVKAEMRLYHLYN
CM4722_Cyanidioschyzon_merolae       VKDIVRDVMLPWYNAFRFFISNPMDRWILSLLASLISFLHKEMRAYRLYT
118380025_Tetrahymena_thermo         VFAVKKDIFLPWYNAYKFLITNPTDRWIIISCQNLINYVRIEMEKYHLYN
23619270_Plasmodium_falcip           VNEIVKSFILPYYHSFRFFSDNIMDQWIFSSLQSLINSVHTEMKAYKLYN
71076452_Giardia_lambli              VKGVTKDLFVPWYNSFRFFTTSVLDMWILASFEQLVATVRREMASYQLYN
42523714_Bdellovibrio_bacter         VYDIVRKILLRWWNSYSFFAPNILDQWVLSRLNGLIANTHKEMDAYRLYN
6319395_Saccharomyces_cerevi         VKEVVSKVLLPWWNSFKFLDDNVMDRWILASMQSLVQFIHEEMGQYKLYT
66816517_Dictyostelium_discoi        VQDMIKDVFLPWFNAYRFFVENVMDKWILASCQSLIAFVRAEMAAYRLYT
24668543_Drosophila_melano           VRDIIKDVFLPWYNAYRFLLASVIDVWILSFKESLLEFFATEMKMYRLYT
94721239_Homo_sapien                 VRDVLKDVLLPWYNAYRFLIPNITDRWILSFMQSLIGFFETEMAAYRLYT
116061521_Ostreococcus_tauri         VFGVLKDVFLPWYNAYRFLVTNVLDIWITSSTNSLVKFVTEEMQDYKLYT
30681405_Arabidopsis_thalia          VLGVVKDVFLPWYNAYRFLVANILDQWIHSATQSLVRFVREEMDAYRLYT
67481173_Entamoeba_histol            LKELISTIFLPWFNTLRYLKMNDMDRWILSSLMSLVKKVREEMKMFRLYT
```

```
57157197_Trichomonas_vagina         VAMIVRQTMLPWMNSIKFWVENILDKWILSKINRLIQHVHREMGLYQLYN
48477483_Picrophilus_torrid         IGEITRKNLSTLLNVYSFFAENLLDRWMLSRLNTTIIKVRENMDNYNIHT
84029573_Thermoplasma_acidophi      IDEVSRKTLMTVLNVYSFFAGNALDRYMVSKVNSFVRSSRDAYESLDFHE
60681847_Bacteroides_fragil         IEEVRRKFFGTLYNTYSFFARPEIDRWILSVLNTLVKEVDTCYNEYEPTK
83815822_Salinibacter_ruber         LRDLRRTFFGTLENVYRFFARPELDQWIISRLHTTTQAVEAALDEYDPTT
21673150_Chlorobium_tepidu          IEEEQRKFFRAFINSYNFFVRSELDRWVLSSLNTLIAEVTSRMEQYDLTG
111221641_Frankia_alni              IEDIVRKVLLTYWNTASFFARHVLDRWALSELAATVTEVDESLAGFDSLR
28210025_Clostridium_tetani         VEETQRKFLSTLWNVYSFYVENVMDKWIVSKLNSLIKDVEDHMDSYRITQ
47570085_Bacillus_cereus            VLEAKSKFVDTLVNVYSFYVQTKLDEWVLSRLHSTTKKVRTALDDYQFTN
87311784_Blastopirellula_marin      IKDSIPEFLLRLWNVYSFFTRSELDRWVLSELNQAIATVVERMDAYDNYG
94985204_Deinococcus_geothe         VAEAQRNYVNTLWNVYAFFVRPEMDRWLLARLEETVRDVTTSLDAYDARG
76261183_Chloroflexus_aurant        VSESMRKFLLTLWNTYAFFVLQPIDRWALAALNQLVQQVTTCFEEYDVYT
                                                            :

118193839_Cenarchaeum_symbio        YARSLESFINMLSQVYYVPIVRGELWYAVISEVLQALDVMLHPISPYTTEY
6325217_Saccharomyces_mt            VLITLQYHNNELSAFYFDISKDILYQTTLVHILNAYRAILAPILPVMVQE
66800699_Dictyostelium_mt           VHTEIINFSIEISSFYFDVIKRHLYQTVLFKMLDVINIALAPITVHTSED
118751535_Marinomonas_sp.           VNQKIQNFSVDLGGFYLDVIKDRQYQTALYHVIEAFSRWIAPILSFTADE
46129096_Haemophilus_influe         VVQRLMRFSVEMGSFYLDIIKDRQYQTALWHIAEALVRWMAPILSFTADE
75237181_Escherichia_coli           VVQRLMRFSVEMGSFYLDIIKDRQYQTALYHIAEALVRWMAPILSFTADE
47572014_Rubrivivax_gelati          VVAKLQVYSEDLGAFYLDVLKDRLYQTALHRITGAMLRWMAPFLSFTAEE
1174519_Pseudomonas_fluorescen      VYSKIHNFVQELGGFYLDIIKDRQYQTALYHISEALVRWIAPILAFTADE
83594296_Rhodospirillum_rubrum      MFQELHGFAVDLSAFYLDIRKDALYRTVIDTVFDCLVKWLAPFVCFTAEE
94968888_Acidobacteria_bacter       LFMRLKDFVVDLSAVYFDVIKDRLYQTAIWTIGEALVRLLAPLMSFTAEE
15644113_Thermotoga_maritima        VYNLVVKYTTELSSLYLDVVKDRLYQTVMHEILISLMKILAPIMTFTMEE
15605834_Aquifex_aeolic             VHNHIKNFITTLSAIYLDVLKDRLYQTALWHLLIALTTSTAPYLSFTAEE
116747573_Syntrophobacter_fuma      VYHAFHNYVVDLSAFYLDILKDRLYQTAVYRILSALLRLMAPILSFTAEE
116057018_Ostreococcus_tauri        VVSAVTSFTTFLSNVYLDVSKDKLYQTVVSALVERIIAAIAPLTPHMAEE
CM546_Cyanidioschyzon_merolae       IFQSVLRFVADLSNFYLDIAKDRCYQATMWAILMDMARALAPILPHTVED
33239720_Prochlorococcus_marin      FFQLLQSFVVDLSNFYLDIAKDRLYQYVLSLIVERLAGVISPVLSHTAED
22327660_Arabidopsis_cp             IFQIIQRFIVDLSNFYFDIAKDRLYQTVLSTHLLSILRVIAPIVPHLAED
56751678_Synechococcus_elonga       FFQTVQNFVVDLSNFYLDIGKDRLYQTVLAICVEALATAIAPVLSHMAED
113477079_Trichodesmium_erythr      FFQTIQNFVVDLSNFYLDIAKDRLYQTVLVVAVENLAKAIAPVLPHMAED
67921387_Crocosphaera_watson        FFQTVQNFVVDLSNFYLDIAKDRLYQTVLAVAIENLAKAIAPVLCHMAED
34763705_Fusobacterium_nuclea       LFQGIHYFAIDMSAFYLDIIKDRLYQTVMYEVLMTLTKMVAPILSFTAEE
47570310_Bacillus_cereus            VYHAIHNFTIDLSSFYLDFAKDILYQTVLYDVLVALTKLVTPILPHTADE
46852147_Homo_mt                    VVRLLRTFTRELSNFYFSIIKDRLYQTALVEILDVIVRSFAPILPHLAEE
24665024_Drosophila_melano          VVACVQNFANQVSAVYVHLIKDRLYRQTLTHCYQQLCKSLWPIVPFLVEE
149179295_Planctomyces_maris        FLKNATAFIDDLSNWYIRRNRRRFWYQTLFEVLVTLSKALAPSIPFLSER
18312756_Pyrobaculum_aeroph         AAALWREFVETLSHRYIRLLRRRVWYAVLHDVLKKVLILGSILVPFITEY
70606414_Sulfolobus_acidoc          LANKVTAFIDDISRFYLRVTRKRAWYYVLYHVLKGSLILLSTVIPFTAEK
14600670_Aeropyrum_pernix           AARALRSFIEDVSHWYLRIIRRRVWYATLYKVLWEWLLMAAPLIPYTAEY
84490249_Methanosphaera_stadtm      ATEKITDFLEDLSRWYVRLIRGRTWYYTLYYTLKNLIMVLAPISPHVTEE
11498241_Archaeoglobus_fulgid       VVRAFFDFVEDFSRWYIQIIRPRVWYYTMFRVIDRSLRAIAPFAPLIAEW
14521127_Pyrococcus_abyssi          ATRALYNFVEDLSRWYVRLIRKRLWYYTLWKVFDVLLRLLAPFTPYIAEE
75401346_Pseudomonas_fluoresce      IYSRIEVFINVLSTWYLRLNKARIWYEVLHYALSNFARLLAPFMPFLAEA
102191610_Rickettsia_canade         AYHQVSEFFEVLNNWYIRRSRARFWYNTLYSCLETMAIAMSALVPMISEA
116670944_Arthrobacter_sp.          ACDELRSYLDMLTNWYVRRSRQRFF-DALYTALETVSRVAASLLPLVSEE
15827608_Mycobacterium_leprae       ACEQLRQFTEPLTNWYLRRSRARFW-DTLHTVLEVTARLAAPLLPLITEI
54023744_Nocardia_farcin            ACEELRTFADALTNWYVRRSRSRFW-DTLHTVLEVATRLAAPLLPLISEV
19074910_Encephalitozoon_cunic      VLTLALRFIDDLSNWYIRMYRKEI-HAVLGEILKKFSIVMGPFTPFFSEY
51599084_Borrelia_garini            SIESLLEFIDKLNNWYIRRSRRRFWYETLYYAIKTLMILLAPFIPFITEE
42528163_Treponema_dentic           AIPPMVEYIDLLNNWYIRRSRRRFWYETLYRALKKFSLVAAPVVPFITES
46446128_Protochlamydia_amoeba      SVEPFVNFVDQLTNWYIRRSRRRFWFETLYYVLIELTKISAPYVPFISEA
89898154_Chlamydophila_felis        AVSPFVSFIDDLTNWYIRRCRRRFWFATLYEVLTVFCRVIAPFIPFISED
118749311_Marinomonas_sp.           VVPALFEFIEDLTNGYIRLNRARFWYHTLYSCLDTFSRLMAPFTPFLAEH
68130224_Leishmania_major           VVPGILRFVGDLSNWYVRMNRRRMKLSTMLYLLFSVSRIVGHIAPFVAEM
CM4722_Cyanidioschyzon_merolae      VVPRLLLFIESLTNWYVRLNRPRLKLCTLGDLLSRLAVLMAPFAPFMAEW
118380025_Tetrahymena_thermo        VVPRLIHFLENLTNWYIRLNRNRLKLNVLFNVILNSTILMSPLVPFITES
23619270_Plasmodium_falcip          VLPKLLNFIENLTNWYIRLNRDRMRLNVLCRTLYLFTIIMAPFTPFISEY
71076452_Giardia_lambli             VLPELLRFIDDLTKWYIRFNRDTLKLNVLFYVLFMLCRLMAPYTPFVCDW
42523714_Bdellovibrio_bacter        VVPHLLQFIEDLTNTYIRFNRSLFWYETLHEVLVTLSRLMAPFAPFMSEV
6319395_Saccharomyces_cerevi        VVPKLLNFIDELTNWYIRFNRRRLKLNSLFDALFTFVRAMAPFTPFLSES
66816517_Dictyostelium_discoi       VVPKLVRFIEDLTNWFVRLNRKRLKLNILYEVLMTICIAMGPFTPFFTEY
24668543_Drosophila_melano          VVPRLTKFIDQLTNWYVRLNRRRIKLDTLYDVLYTMVKMMAPFTPYLTEY
94721239_Homo_sapien                VVPRLVKFVDILTNWYVRMNRRRLKLETLFSVLLSLCRLMAPYTPFLTEL
116061521_Ostreococcus_tauri        VVPKLISFIDQLTNIYVRYNRGRLKLNVLFHVLLTLCKTMAPFTPFFVEN
30681405_Arabidopsis_thalia         VVPRLLKFLDNLTNIYVRFNRKRLKLSTLFNVLLTSCKVMAPFTPFFTET
67481173_Entamoeba_histol           IITPLVDLLVTLSNWYIRLNRKRFRCSVLYHCLKIMSILMAPFTPFFSEF
57157197_Trichomonas_vagina         VLPELVKFIDQLIN------------------------------------
48477483_Picrophilus_torrid         ALRYIEDLISELSNVYLRLSRKRFWYSTLYYTLRETIKMMAPITPFFSEY
84029573_Thermoplasma_acidophi      VVRASMEFVDDLSNFYLRLSRRRFWYSTLYYALKAFSEVMAPITPFFSDF
60681847_Bacteroides_fragil         AGRLISDFNDNLSNWYVRLNRKRFWYQTLYTCLETVAKLMAPIAPFYADR
```

```
83815822_Salinibacter_ruber         AARAVEDFVEELSNWHLRRSRPRFWYQTIHECLAATAKLMSPIAPFFGEW
21673150_Chlorobium_tepidu          ACRLIGDFVDDLSNWYIRRSRKRFWYQTLSTVLETLAKLMAPFVPFIAEK
111221641_Frankia_alni              AGRRIARFIDDLSNWYVRRSRRRFW-TTLYTCLDALTRVMAPFTPFLTDW
28210025_Clostridium_tetani         AALAIEDFVDELSNWYVRRNRSRFWYVTLYKVLTTVSLIAAPFVPFITEE
47570085_Bacillus_cereus            AAREIAALVDEVSNWYVRRSRNRFWYETLHEVLVTISKLIAPFAPFVAED
87311784_Blastopirellula_marin      ACGALTNFVDALSNWYVRRSRDRFWYWTLYECLITTCKLIAPFTPFLAEG
94985204_Deinococcus_geothe         GGRALERFVHDLSNWYVRRNRSRFWYATLHEALLTVSQLTAPFTPFLAEA
76261183_Chloroflexus_aurant        AANAIEHFVDELSNWYVRRNRRRFWYQTLYTCLVTVAKLAAPFIPFVSEE
                                               .

118193839_Cenarchaeum_symbio        LYSILLEGW
6325217_Saccharomyces_mt            VWNPMRGKW
66800699_Dictyostelium_mt           VFSVFAHGW
118751535_Marinomonas_sp.           IWSVFLETW
46129096_Haemophilus_influe         IWFVFTEEF
75237181_Escherichia_coli           VWYVFTGEW
47572014_Rubrivivax_gelati          AWSIFTQTY
1174519_Pseudomonas_fluorescen      LWSVMLNTW
83594296_Rhodospirillum_rubrum      AWSVHFQSF
94968888_Acidobacteria_bacter       LWTVHAAYF
15644113_Thermotoga_maritima        VYTVQAEYW
15605834_Aquifex_aeolic             LWSVFLYEM
116747573_Syntrophobacter_fuma      AWTVHLEEF
116057018_Ostreococcus_tauri        AFSVFIAGW
CM546_Cyanidioschyzon_merolae       LWSIFQNGW
33239720_Prochlorococcus_marin      IWSVFQRYW
22327660_Arabidopsis_cp             VWFVFELKW
56751678_Synechococcus_elonga       IWSVFQAGW
113477079_Trichodesmium_erythr      IWSVFESGW
67921387_Crocosphaera_watson        IWSVFEAGW
34763705_Fusobacterium_nuclea       IWSIFLADW
47570310_Bacillus_cereus            VWSVQLTDM
46852147_Homo_mt                    VFSVFRTGW
24665024_Drosophila_melano          SWGAFHEQI
149179295_Planctomyces_maris        MYSVHLCDF
18312756_Pyrobaculum_aeroph         LWSVHLAQY
70606414_Sulfolobus_acidoc          IYSISMEKI
14600670_Aeropyrum_pernix           LYSIHMLDM
84490249_Methanosphaera_stadtm      IYSVHMLDW
11498241_Archaeoglobus_fulgid       FYSIFMEEY
14521127_Pyrococcus_abyssi          IYSVHMLDW
75401346_Pseudomonas_fluoresce      VYSVHLQDW
102191610_Rickettsia_canade         IYSVHLCNY
116670944_Arthrobacter_sp.          IWSVHLADW
15827608_Mycobacterium_leprae       IWSVHLTDW
54023744_Nocardia_farcin            IWSVHLADW
19074910_Encephalitozoon_cunic      SYSVHFQEY
51599084_Borrelia_garini            IYSIHLNDY
42528163_Treponema_dentic           IWSIHLADY
46446128_Protochlamydia_amoeba      IYSVHLCDF
89898154_Chlamydophila_felis        IYSVHLCDF
118749311_Marinomonas_sp.           LYSVHLCDY
68130224_Leishmania_major           LYSVHYLMI
CM4722_Cyanidioschyzon_merolae      TYSVHFLPL
118380025_Tetrahymena_thermo        FYSIHFLRI
23619270_Plasmodium_falcip          IYSVHFIML
71076452_Giardia_lambli             MYSIHFWAV
42523714_Bdellovibrio_bacter        TYSVHLESF
6319395_Saccharomyces_cerevi        IYSVHFLSY
66816517_Dictyostelium_discoi       MYSVHYVMF
24668543_Drosophila_melano          IFSVHYQMM
94721239_Homo_sapien                MYSIHYLML
116061521_Ostreococcus_tauri        IYSIHFCEF
30681405_Arabidopsis_thalia         LYSVHYCSI
67481173_Entamoeba_histol           CYSVHFVQI
57157197_Trichomonas_vagina         ---------
48477483_Picrophilus_torrid         LYSVHMESY
84029573_Thermoplasma_acidophi      IYSVHLEAF
60681847_Bacteroides_fragil         LYSVHLAKF
83815822_Salinibacter_ruber         LYSVHLASF
21673150_Chlorobium_tepidu          IWSVHLADW
111221641_Frankia_alni              LWSVHLAAW
28210025_Clostridium_tetani         IYSIHLCNW
```

```
47570085_Bacillus_cereus           IHSVHLEDY
87311784_Blastopirellula_marin     LWSVHLCDF
94985204_Deinococcus_geothe        LYSVHLTRW
76261183_Chloroflexus_aurant       IYSVHLAQW
```

Note: There are two eukaryotic sequence clades for this gene, each of which clusters within bacterial homologs with strong support. The minor eukaryotic sequence clade (lower part of the tree) contains chloroplast precursors from plants and mitochondrial precursors from opisthokonts. Sequences in the major eukaryotic sequence clade are cytosolic. It is likely that the major eukaryotic sequence clade resulted from an ancient HGT event prior to the split of most eukaryotic super groups. An alternative explanation is that the common ancestor of cellular organisms contained two copies of this gene, which were differentially retained among lineages.

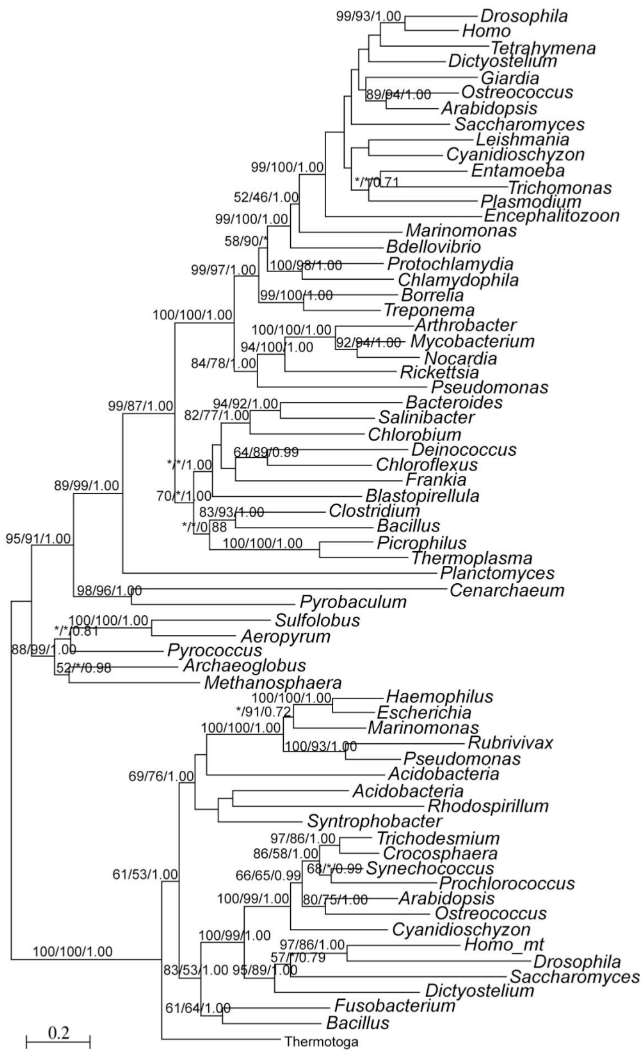

Figure 22. Molecular phylogeny of isolecucyl-tRNA synthetase. P-value = 0.235 from AU test for the presented tree. AU test was also performed on an alternative topology enforcing a sequence monophyly of archaea and the major eukaryotic group. Such an alternative topology is based on the common belief that archaea and eukaryotes are more closely related than each is to bacteria. P-value < 0.001 from AU test for the alternative topology.

23. IspD (D)

See Huang and Gogarten 2007. Did an ancient chlamydial endosymbiosis facilitate the establishment of primary plastids? Genome Biology 8:R99.

24. Polynucleotide phosphorylase (D)

See Huang and Gogarten 2007. Did an ancient chlamydial endosymbiosis facilitate the establishment of primary plastids? Genome Biology 8:R99.

25. ATP/ADP translocase (N)

See Huang and Gogarten 2007. Did an ancient chlamydial endosymbiosis facilitate the establishment of primary plastids? Genome Biology 8:R99.

26. Glycero-3-phosphate acyltransferase (N)

See Huang and Gogarten 2007. Did an ancient chlamydial endosymbiosis facilitate the establishment of primary plastids? Genome Biology 8:R99.

27. Sodium:hydrogen antiporter (N)

See Huang and Gogarten 20007. Did an ancient chlamydial endosymbiosis facilitate the establishment of primary plastids? Genome Biology 8:R99.

28. GcpE (D)

See Huang and Gogarten 2007. Did an ancient chlamydial endosymbiosis facilitate the establishment of primary plastids? Genome Biology 8:R99.

29. Beta-ketoacyl-ACP synthase (fabF) (D)

See Huang and Gogarten 2007. Did an ancient chlamydial endosymbiosis facilitate the establishment of primary plastids? Genome Biology 8:R99.

30. Aspartate aminotransferase (D)

See Huang and Gogarten 2007. Did an ancient chlamydial endosymbiosis facilitate the establishment of primary plastids? Genome Biology 8:R99.

31. tyrosyl-tRNA synthetase (D)

See Huang and Gogarten 2007. Did an ancient chlamydial endosymbiosis facilitate the establishment of primary plastids? Genome Biology 8:R99.

32. Cu-ATPase (D)

See Huang and Gogarten 2007. Did an ancient chlamydial endosymbiosis facilitate the establishment of primary plastids? Genome Biology 8:R99.

33. IspE (D)

See Huang and Gogarten 2007. Did an ancient chlamydial endosymbiosis facilitate the establishment of primary plastids? Genome Biology 8:R99.

34. Enoyl-ACP reductase (fabI) (D)

See Huang and Gogarten 2007. Did an ancient chlamydial endosymbiosis facilitate the establishment of primary plastids? Genome Biology 8:R99.

35. Florfenicol resistance protein (E)

See Huang and Gogarten 2006. Ancient horizontal gene transfer can benefit phylogenetic reconstruction. Trends in Genetics 22:361-366.

36. 23S rRNA (Uracil-5-)-methyltransferase (D)

See Huang and Gogarten 2007. Did an ancient chlamydial endosymbiosis facilitate the establishment of primary plastids? Genome Biology 8:R99.

37. Topoisomerase 6 subunit B (TOP6B) (N)

See Huang and Gogarten 2006. Ancient horizontal gene transfer can benefit phylogenetic reconstruction. Trends in Genetics 22:361-366.
